# Supplementary material for: Asymmetric benzylic C(sp3)−H acylation via dual nickel and photoredox catalysis
Source: Nat Commun. 2021 Jun 10;12:3536. doi: 10.1038/s41467-021-23887-2 (PMC8192574; doi:10.1038/s41467-021-23887-2)
Supplement: Supplementary file 1 — Supplementary Information [file 41467_2021_23887_MOESM1_ESM.pdf]

Asymmetric Benzylic C(sp^3)–H Acylation via Dual Nickel and Photoredox Catalysis

Leitao Huan, Xiaomin Shu, Weisai Zu, De Zhong, and Haohua Huo*

State Key Laboratory of Physical Chemistry of Solid Surfaces, Key Laboratory of Chemical Biology of Fujian Province, College of Chemistry and Chemical Engineering, Xiamen University, Xiamen 361005, P. R. China

Supplementary Information

Table of Contents

**I.**	**General Information**	**S–2**
**II.**	**Effect of Reaction Parameters**	**S–3**
**III.**	**Catalytic Enantioselective Benzylic C(sp^3)–H Acylation**	**S–5**
**IV.**	**Synthetic Utility: Gram Scale Synthesis and Parallel Synthesis**	**S–40**
**V.**	**Mechanistic Studies**	**S–48**
**VI.**	**Assignment of Absolute Configuration**	**S–54**
**VII.**	**^{1}H-NMR and ^{13}C-NMR Spectra; Stereoselectivity Analysis**	**S–57**
**VIII.**	**Supplementary References**	**S–216**

I. General Information

Unless otherwise noted, reactions were performed with rigorous exclusion of air and moisture. Anhydrous *i*-PrOAc (>99.6%, Sigma-Aldrich) and EtOAc (99.9%, J & K) were dried using freshly activated 4Å MS and bubbled with argon for 1 h before it was brought into the glovebox. Chiral ligands (>98%, DAICEL), $NiBr_2$·glyme (>97%, Strem), NH_4Cl (99.999%, Alfa Aesar), Na_2HPO_4 (>99.0%, Sigma-Aldrich), and DMDC (Rhawn), K_2HPO_4 (>99.9%, Sigma-Aldrich), $KHCO_3$ (>99.9%, Sigma-Aldrich), phenyl carbonochloridate (98%, Energy), and all commercially available carboxylic acids and alkylarenes (Alfa Aesar, Energy Chemical, TCI, and Sigma-Aldrich) were used as received. Alkylarenes for preparation of compounds **26**, **34**, **35**, **41**, **42**, **47**, **50**, **51**, **61**-**65**, and **53**, were synthesized according to a literature procedure,[1] and all analytical data matched that report.

NMR spectra were collected on a Bruker 400 MHz, a Bruker 500 MHz, a Bruker 600 MHz, or a Varian 500 MHz spectrometer at ambient temperature. HPLC analyses were carried out on an Agilent 1260 series system with Daicel CHIRALPAK® or Daicel CHIRALCEL® columns (4.6 × 250 mm, particle size 3 μm and 5 μm). FT-IR measurements were carried out on a Nicolet AVATER FTIR330 spectrometer. High resolution mass spectra (ESI) were recorded by the instrumentation center of Department of Chemistry, Xiamen University, on a high-resolution LC/MS instrument. Optical rotation data were obtained with an Anton Paar MCP 500 polarimeter at 589 nm and at 25 °C, using a 100 mm path-length cell in the solvent and at the concentration indicated. GC analyses were obtained on an Agilent 6890A GC. Flash column chromatography was performed using silica gel (300–400 mesh). Blue LED lamps (40 W; Kessil PR160L) were used to irradiate the reaction mixtures.

II. Effect of Reaction Parameters

General Procedure A (GP-A): In a glovebox, Ir[dF(CF_3)ppy]$_2$(dtbbpy)PF_6 (1.1 mg, 0.001 mmol, 1%), $NiBr_2$·glyme (3.1 mg, 0.010 mmol, 10%), (*S*)-**L** (4.7 mg, 0.013 mmol, 13%), NH_4Cl (5.3 mg, 0.10 mmol, 1.0 equiv), Na_2HPO_4 (21.3 mg, 0.15 mmol, 1.5 equiv), a Teflon stir bar, and anhydrous *i*-PrOAc (1.0 mL) were added sequentially to a 4-mL vial. The reaction mixture was stirred at room temperature for 30 min, after which it turned to a purple suspension. A solution of the 4-ethylbiphenyl (0.15 mL, 2.0 M in *i*-PrOAc, 0.30 mmol, 3.0 equiv) was added via a 0.25 mL syringe. The vial was sealed with a septum cap and wrapped with electrical tape, followed by the sequential addition of 3-phenylpropanoic acid (0.10 mL, 1.0 M in *i*-PrOAc, 0.10 mmol, 1.0 equiv) and DMDC (16.2 μL, 0.15 mmol, 1.5 equiv) via microsyringe. Next, the vial was transferred out of the glovebox, the vacuum grease was liberally applied to cover the entire top of septum cap. Then, the reaction mixture was stirred at 10 °C in an EtOH bath for 5 min before being irradiated with a 40 W blue LED lamp (Kessil PR160L, 427 nm). The reaction was stirred at 10 °C under irradiation for 25 hours. Next, the lamp was turned off and the resulting mixture was allowed to warm to room temperature, and then dodecane (22 μL, 0.10 mmol) was added as an internal standard. The mixture was filtered through a small plug of silica gel, which was flushed with Et_2O (~10 mL). A portion of the filtrate (~0.1 mL) was diluted with acetone (total volume: 1 mL) and analyzed via GC, and the remainder of the filtrate was concentrated via rotary evaporation, and the pure product was isolated by preparative TLC on silica gel (1:30 EtOAc/hexanes).

The results for the effect of reaction parameters were shown in Table 1 and Supplementary Table 1. **GP-A** was followed for the experiments set-up, using 3-phenylpropanoic acid (0.10 mmol) and 4-ethylbiphenyl (0.30 mmol), the yield was determined via GC analysis with dodecane as an internal standard. The ee values were determined via HPLC analysis after purification by preparative thin-layer chromatography.

Supplementary Table 1. Further study of effect of reaction parameters

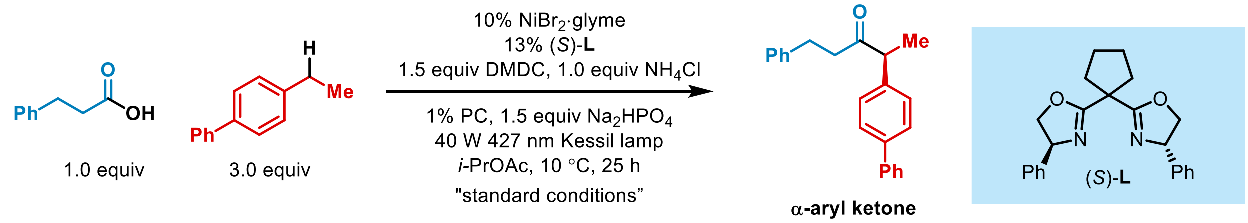

entry	variation from standard conditions	yield (%)[a]	ee (%)[b]	entry	variation from standard conditions	yield (%)[a]	ee (%)[b]
1	none	85	94	11	as entry 10, but plus 1.5 equiv NaBr	72	91
2	no (*S*)-**L**	33	-	12	Ir[dF(CF_3)ppy]$_2$(bpy)PF_6, instead of **PC**	63	94
3	no base	83	92	13	2% 4CzIPN, instead of **PC**	0	-
4	5% $NiBr_2$·glyme, 7% (*S*)-**L**	70	94	14	25% **PC1**, instead of **PC**	0	-
5	under air in a capped vial	71	93	15	25% **PC2**, instead of **PC**	0	-
6	Plus 0.2 equiv H_2O	78	93	16	Plus 25% **PC1**	77	94
7	EA, instead of *i*-PrOAc	73	93	17	Plus 25% **PC2**	81	95
8	dioxane, instead of *i*PrOAc	72	93	18	**L-S1-S8**, instead of (*S*)-**L**	as shown below	
9	Benzene, instead of *i*PrOAc	32	96	19	other carboxylic acids	as shown below	
10	$NiCl_2$·glyme, instead of $NiBr_2$·glyme	14	83	20	other alkylarenes	as shown below	

PC = Ir[dF(CF_3)ppy]$_2$(dtbbpy)PF_6. DMDC = Dimethyl dicarbonate. [a] Determined through GC analysis. [b] Determined through HPLC analysis.

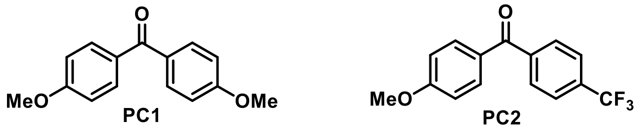

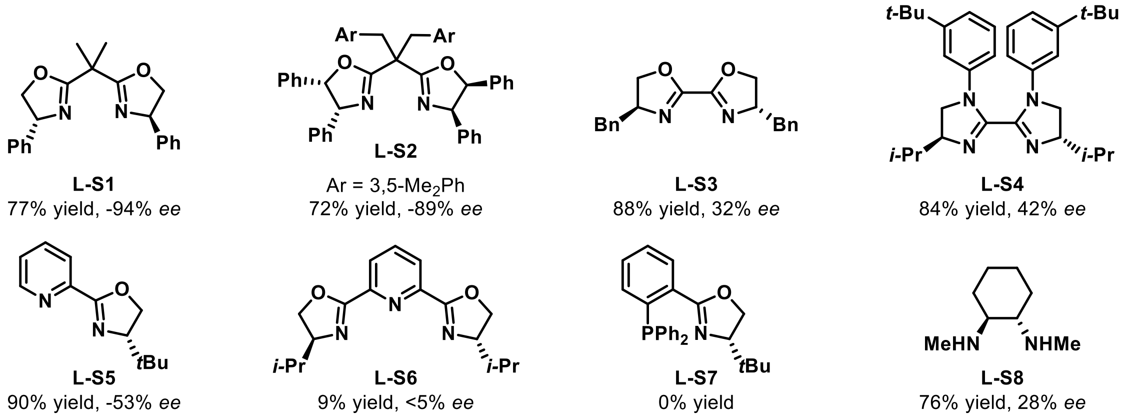

Observed limitations in both coupling partners:

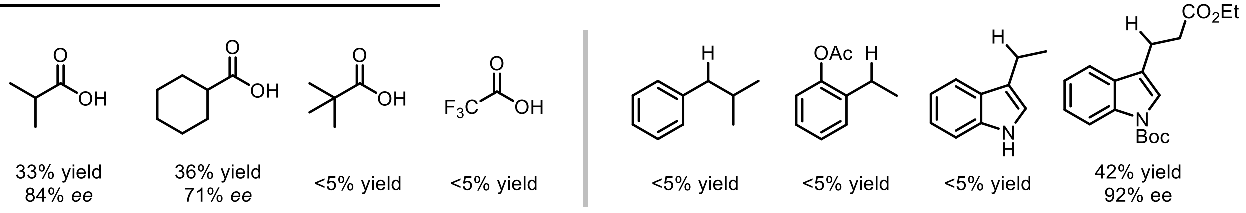

III. Catalytic Enantioselective Benzylic C(sp^3)–H Acylation

Supplementary Methods

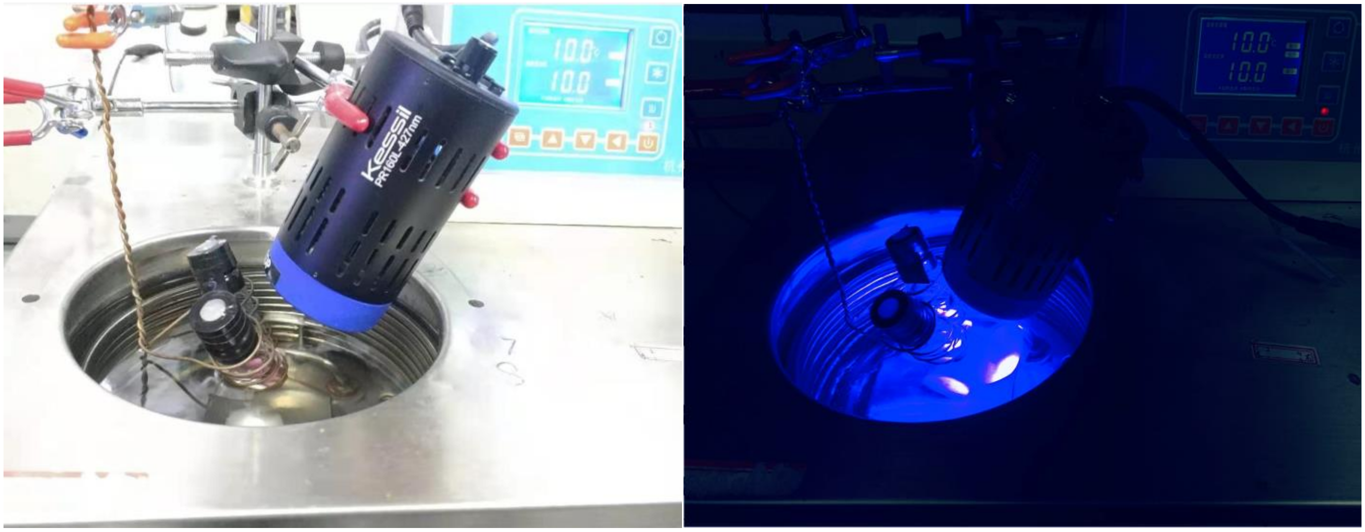

Supplementary Figure 1. Exemplary reaction setup (two runs for each substrate)

General Procedure B (GP-B): Catalytic enantioselective acylation of benzylic C(sp^3)–H bonds with alkyl carboxylic acids. In a glovebox, Ir[dF(CF$_3$)ppy]$_2$(dtbbpy)PF$_6$ (5.5 mg, 0.005 mmol, 1%), $NiBr_2$·glyme (15.5 mg, 0.05 mmol, 10%), (*S*)-**L** (23.5 mg, 0.065 mmol, 13%), NH_4Cl (26.5 mg, 0.50 mmol, 1.0 equiv), Na_2HPO_4 (106.5 mg, 0.75 mmol, 1.5 equiv), a Teflon stir bar, and anhydrous *i*-PrOAc (5.0 mL) were added sequentially to a 15 mL vial. The reaction mixture was stirred at room temperature for 30 min, after which it turned to a purple suspension. Next, a solution of the alkylarene (0.75 mL, 2.0 M solution in *i*-PrOAc, 1.50 mmol, 3.0 equiv) was added via a 1.0 mL syringe (if the carboxylic acid was a solid, it was added as solid directly at this point following the addition of alkylarene). The vial was closed with a PTFE septum cap and wrapped with electrical tape. Then, carboxylic acid (0.50 mmol, 1.0 equiv) and DMDC (80.0 μL, 0.75 mmol, 1.5 equiv) were added sequentially via microsyringe. Next, the vial was transferred out of the glovebox, and then vacuum grease was liberally applied to cover the entire top of the septum cap. Then, the reaction mixture was stirred at 10 °C in an EtOH bath for 5 min before being irradiated with a 40 W blue LED lamp (Kessil PR160L, 427 nm). The reaction was stirred under irradiation at 10 °C for 25 hours. The reaction mixture was then passed through a short pad of silica gel, with Et_2O as the eluent (~35 mL). The resulting mixture was concentrated, and the residue was purified by chromatography on silica gel.

For compounds **49** and **58**, the procedure is the same as above, but the reaction was conducted at 25°C in dioxane; For compound **56**, the procedure is the same as above, but the reaction was conducted at 25 °C in place of 10 °C; For the compound **57, i**n place of the standard conditions, 5.0 equiv of 4-ethylbiphenyl was used; For the compounds **51** and **52**, the reactions were stirred for 15 hours instead of 25 hours.

General Procedure C (GP-C): Catalytic enantioselective acylation of benzylic C(sp^3)–H bonds with artesunate. The procedure is the same as **GP-B**, except for changes in the following quantities: alkylarene (2.5 mmol, 5.0 equiv), and the reaction was stirred for 35 hours.

General Procedure D (GP-D): Catalytic enantioselective acylation of benzylic C(sp^3)–H bonds with aromatic carboxylic acids. In a glovebox, Ir[dF(CF$_3$)ppy]$_2$(dtbbpy)PF$_6$ (5.5 mg, 0.005 mmol, 1%), NiBr$_2$·glyme (15.5 mg, 0.05 mmol, 10%), (*S, R*)-**L3** (20.6 mg, 0.065 mmol, 13%), NH$_4$Cl (26.5 mg, 0.50 mmol, 1.0 equiv), K$_2$HPO$_4$ (261.3 mg, 1.50 mmol, 3.0 equiv), a Teflon stir bar, and anhydrous *i*-PrOAc (5.0 mL) were added sequentially to a 15 mL vial. The reaction mixture was stirred at room temperature for 30 min, after which it turned to a grass green suspension. Next, 4-propyl-1,1'-biphenyl (297 μL, 1.50 mmol, 3.0 equiv) was added via microsyringe, then aromatic carboxylic acids were added directly as a solid. The vial was closed with a PTFE septum cap and wrapped with electrical tape. Then, DMDC (161 μL, 1.50 mmol, 3.0 equiv) were added sequentially via a microsyringe. Next, the vial was transferred out of the glovebox, and then vacuum grease was liberally applied to cover the entire top of the septum cap. Then, the reaction mixture was stirred at 10 °C in an EtOH bath for 5 min before being irradiated with a 40 W blue LED lamp (Kessil PR160L, 427 nm). The reaction was stirred under irradiation at 10 °C for 28 hours. The reaction mixture was then passed through a short pad of silica gel, with Et$_2$O as the eluent (~35 mL). The resulting mixture was concentrated, and the residue was purified by preparative thin-layer chromatography on silica gel.

General Procedure E (GP-E): Catalytic enantioselective acylation of benzylic C(sp^3)–H bonds with phenyl chloroformate. In a glovebox, Ir[dF(CF$_3$)ppy]$_2$(dtbbpy)PF$_6$ (5.5 mg, 0.005 mmol, 1%), NiBr$_2$·glyme (15.5 mg, 0.05 mmol, 10%), (*S, R*)-**L2** (49.3 mg, 0.065 mmol, 13%), KHCO$_3$ (75.1 mg, 0.75 mmol, 1.5 equiv), a Teflon stir bar, and anhydrous EtOAc (5.0 mL) were added sequentially to a 15 mL vial. The reaction mixture was stirred at room temperature for 30 min, after which it turned to a brown red suspension. Next, alkylarene (1.50 mmol, 3.0 equiv) was added via microsyringe. The vial was closed with a PTFE septum cap and wrapped with electrical tape. Then, phenyl chloroformate (63 μL, 0.50 mmol, 1.0 equiv) were added sequentially via a microsyringe. Next, the vial was transferred out of the glovebox, and then vacuum grease was liberally applied to cover the entire top of the septum cap. Then, the reaction mixture was stirred at −30 °C in an EtOH bath for 5 min before being irradiated with a 40 W blue LED lamp (Kessil PR160L, 427 nm). The reaction was stirred under irradiation at −30 °C for 40 hours. The reaction mixture was then passed through a short pad of silica gel, with Et$_2$O as the eluent (~35 mL). The resulting mixture was concentrated, and the residue was purified by preparative thin-layer chromatography on silica gel.

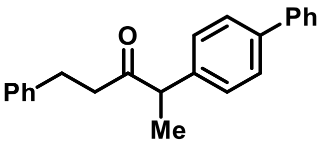

4-([1,1'-Biphenyl]-4-yl)-1-phenylpentan-3-one (Fig. 2, compound 1). The title compound was synthesized according to **GP-B** from 3-phenylpropanoic acid and 4-ethylbiphenyl. The product was purified by column chromatography on silica gel (1:80 EtOAc/Petroleum ether). White solid (mp = 84-85 °C).

(*S*)-**L**: 127 mg, 81% yield, 94% ee; (*R*)-**L**: 130 mg, 83% yield, 94% ee.

HPLC analysis: The ee was determined via HPLC on a CHIRALPAK AD-3 column (5.0% 2-PrOH in hexanes, 1.0 mL/min); retention times for compound obtained using (*S*)-**L**: 6.6 min (major), 6.9 min (minor).

^{1}H NMR (400 MHz, $CDCl_3$) δ 7.56 (d, *J* = 7.7 Hz, 2H), 7.51 (d, *J* = 8.0 Hz, 2H), 7.42 (t, *J* = 7.4 Hz, 2H), 7.33 (t, *J* = 7.4 Hz, 1H), 7.22 – 7.19 (m, 4H), 7.16 – 7.12 (m, 1H), 7.06 (d, *J* = 7.4 Hz, 2H), 3.74 (q, *J* = 6.9 Hz, 1H), 2.90 – 2.63 (m, 4H), 1.40 (d, *J* = 7.0 Hz, 3H).

^{13}C NMR (101 MHz, $CDCl_3$) δ 209.7, 141.0, 140.6, 140.0, 139.4, 128.7, 128.3, 128.25, 128.23, 127.6, 127.3, 127.0, 126.0, 52.8, 42.6, 29.9, 17.3.

FT-IR (film): 2921, 1705, 1485, 1452, 1196, 1180, 1141, 1075, 838, 759, 697 cm^{-1}.

HRMS (ESI-MS) *m/z* [M+Na]$^+$ calcd for $C_{23}H_{22}ONa$: 337.1563, found: 337.1558.

$[\alpha]^{25}_D$ = +101.2 (*c* 1.0, CH_2Cl_2); 94% ee from (*S*)-**L**.

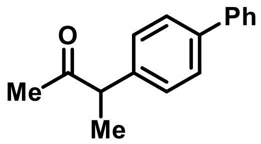

3-([1,1'-Biphenyl]-4-yl)butan-2-one (Fig. 2, compound 2). The title compound was synthesized according to **GP-B** from acetic acid and 4-ethylbiphenyl. The product was purified by column chromatography on silica gel (1:150 EtOAc/Petroleum ether). White solid (mp = 69-70 °C).

(*S*)-**L**: 98 mg, 88% yield, 95% ee; (*R*)-**L**: 99 mg, 88% yield, 95% ee.

HPLC analysis: The ee was determined via HPLC on a CHIRALCEL OJ-3 column (15.0% 2-PrOH in hexanes, 1.0 mL/min); retention times for compound obtained using (*S*)-**L**: 8.8 min (minor), 9.3 min (major).

^{1}H NMR (400 MHz, $CDCl_3$) δ 7.62 – 7.57 (m, 4H), 7.48 – 7.43 (m, 2H), 7.38 – 7.34 (m, 1H), 7.33 – 7.30 (m, 2H), 3.81 (q, *J* = 7.0 Hz, 1H), 2.11 (s, 3H), 1.46 (d, *J* = 7.0 Hz, 3H).

^{13}C NMR (101 MHz, $CDCl_3$) δ 208.6, 140.5, 140.0, 139.5, 128.7, 128.2, 127.6, 127.3, 126.9, 53.3, 28.4, 17.2.

FT-IR (film): 2977, 2931, 1705, 1581, 1450, 1353, 1165, 754, 725, 688 cm^{-1}.

HRMS (ESI-MS) *m/z* [M+Na]$^+$ calcd for $C_{16}H_{16}ONa$: 247.1093, found: 247.1090.

$[\alpha]^{25}_D$ = +212.8 (*c* 1.0, CH_2Cl_2); 95% ee from (*S*)-**L**.

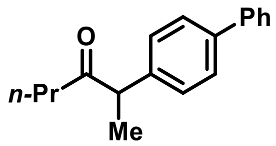

2-([1,1'-Biphenyl]-4-yl)hexan-3-one (Fig. 2, compound 3). The title compound was synthesized according to **GP-B** from butyric acid and 4-ethylbiphenyl. The product was purified by column chromatography on silica gel (1:400 EtOAc/Petroleum ether). White solid (mp = 34-36 °C).

(*S*)-**L**: 111 mg, 88% yield, 94% ee; (*R*)-**L**: 111 mg, 88% yield, 94% ee.

HPLC analysis: The ee was determined via HPLC on a CHIRALCEL OJ-3 column (15.0% 2-PrOH in hexanes, 1.0 mL/min); retention times for compound obtained using (*S*)-**L**: 7.0 min (minor), 8.8 min (major).

^{1}H NMR (400 MHz, $CDCl_3$) δ 7.61 – 7.56 (m, 4H), 7.47 – 7.43 (m, 2H), 7.37 – 7.33 (m, 1H), 7.32 – 7.29 (m, 2H), 3.81 (q, *J* = 7.0 Hz, 1H), 2.41 – 2.37 (m, 2H), 1.63-1.49 (m, 2H), 1.44 (d, *J* = 7.0 Hz, 3H), 0.84 (t, *J* = 7.4 Hz, 3H).

^{13}C NMR (101 MHz, $CDCl_3$) δ 210.8, 140.6, 140.0, 139.7, 128.7, 128.2, 127.5, 127.3, 127.0, 52.5, 43.0, 17.4, 17.2, 13.6.

FT-IR (film): 2976, 2931, 2874, 1712, 1487, 1451, 1136, 763, 726 cm^{-1}.

HRMS (ESI-MS) *m/z* $[M+Na]^+$ calcd for $C_{18}H_{20}ONa$: 275.1406, found: 275.1403.

$[\alpha]^{25}_{D}$ = –195.4 (*c* 1.0, CH_2Cl_2); 94% ee from (*R*)-**L**.

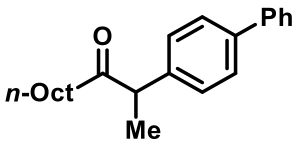

2-([1,1'-Biphenyl]-4-yl)undecan-3-one (Fig. 2, compound 4). The title compound was synthesized according to **GP-B** from nonanoic acid and 4-ethylbiphenyl. The product was purified by column chromatography on silica gel (1:400 EtOAc/Petroleum ether). White solid (mp = 62-63 °C).

(*S*)-**L**: 141 mg, 87% yield, 93% ee; (*R*)-**L**: 137 mg, 85% yield, 94% ee.

HPLC analysis: The ee was determined via HPLC on a CHIRALCEL OJ-3 column (15.0% 2-PrOH in hexanes, 1.0 mL/min); retention times for compound obtained using (*S*)-**L**: 4.5 min (minor), 5.2 min (major).

^{1}H NMR (500 MHz, $CDCl_3$) δ 7.60 – 7.55 (m, 4H), 7.46 – 7.42 (m, 2H), 7.37 – 7.33 (m, 1H), 7.31 – 7.28 (m, 2H), 3.81 (q, *J* = 7.0 Hz, 1H), 2.44 – 2.34 (m, 2H), 1.54 – 1.49 (m, 2H), 1.43 (d, *J* = 7.0 Hz, 3H), 1.27 – 1.24 (m, 2H), 1.22 – 1.20 (m, 8H), 0.86 (t, *J* = 7.0 Hz, 3H).

^{13}C NMR (126 MHz, $CDCl_3$) δ 209.4, 166.9, 134.2, 131.6, 128.5, 127.0, 58.3, 39.9, 31.8, 31.4, 29.3, 29.2, 29.1, 27.0, 23.6, 22.6, 22.5, 14.0, 13.8.

FT-IR (film): 2925, 2853, 1714, 1486, 1456, 764, 567 cm^{-1}.

HRMS (ESI-MS) *m/z* $[M+Na]^+$ calcd for $C_{23}H_{30}ONa$: 345.2189, found: 345.2184.

$[\alpha]^{25}_{D}$ = –140.9 (*c* 1.0, CH_2Cl_2); 94% ee from (*R*)-**L**.

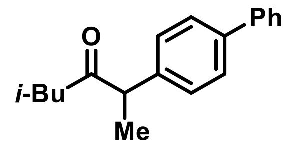

2-([1,1'-Biphenyl]-4-yl)-5-methylhexan-3-one (Fig. 2, compound 5). The title compound was synthesized according to **GP-B** from 3-methylbutanoic acid and 4-ethylbiphenyl. The product was purified by column chromatography on silica gel (1:40 EtOAc/Petroleum ether). White solid (mp = 89-90 °C).

(*S*)-**L**: 107 mg, 80% yield, 95% ee; (*R*)-**L**: 108 mg, 81% yield, 95% ee.

HPLC analysis: The ee was determined via HPLC on a CHIRALCEL OJ-3 column (5.0% 2-PrOH in hexanes, 1.0 mL/min); retention times for compound obtained using (*S*)-**L**: 8.9 min (minor), 11.3 min (major).

^{1}H NMR (500 MHz, $CDCl_3$) δ 7.61 – 7.56 (m, 4H), 7.46 – 7.43 (m, 2H), 7.37 – 7.33 (m, 1H), 7.31 – 7.28 (m, 2H), 3.78 (q, *J* = 7.0 Hz, 1H), 2.34 – 2.24 (m, 2H), 2.17 – 2.11 (m, 1H), 1.43 (d, *J* = 7.0 Hz, 3H), 0.88 (d, *J* = 6.7 Hz, 3H), 0.80 (d, *J* = 6.6 Hz, 3H).

^{13}C NMR (126 MHz, $CDCl_3$) δ 210.4, 140.6, 140.0, 139.6, 128.7, 128.3, 127.5, 127.3, 127.0, 52.9, 50.1, 24.4, 22.6, 22.3, 17.4.

FT-IR (film): 2959, 2930, 2871, 1708, 1633, 1470, 1384, 837, 763, 689 cm^{-1}.

HRMS (ESI-MS) *m/z* $[M+Na]^+$ calcd for $C_{19}H_{22}ONa$: 289.1563, found: 289.1559.

$[\alpha]^{25}_{D}$ = +164.7 (*c* 1.0, CH_2Cl_2); 95% ee from (*S*)-**L**.

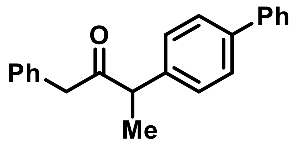

3-([1,1'-Biphenyl]-4-yl)-1-phenylbutan-2-one (Fig. 2, compound 6). The title compound was synthesized according to **GP-B** from 2-phenylacetic acid and 4-ethylbiphenyl. The product was purified by column chromatography on silica gel (1:200 EtOAc/Petroleum ether). White solid (mp = 68-69 °C).

(*S*)-**L**: 74 mg, 50% yield, 92% ee; (*R*)-**L**: 81 mg, 54% yield, 93% ee.

HPLC analysis: The ee was determined via HPLC on a CHIRALPAK AD-3 column (5.0% 2-PrOH in hexanes, 1.0 mL/min); retention times for compound obtained using (*S*)-**L**: 6.7 min (major), 7.5 min (minor).

^{1}H NMR (500 MHz, $CDCl_3$) δ 7.61 – 7.56 (m, 4H), 7.45 (t, *J* = 7.7 Hz, 2H), 7.36 (t, *J* = 7.3 Hz, 1H), 7.30 – 7.22 (m, 5H), 7.09 – 7.08 (m, 2H), 3.91 (q, *J* = 6.9 Hz, 1H), 3.68 (s, 2H), 1.41 (d, *J* = 6.9 Hz, 3H).

^{13}C NMR (126 MHz, $CDCl_3$) δ 207.9, 140.6, 140.2, 139.3, 134.3, 129.4, 128.8, 128.5, 128.4, 127.6, 127.3, 127.0, 126.8, 51.7, 48.1, 17.6.

FT-IR (film): 3028, 2922, 1713, 1486, 1453, 1031, 838, 765, 732, 697 cm^{-1}.

HRMS (ESI-MS) *m/z* $[M+Na]^+$ calcd for $C_{22}H_{20}ONa$: 323.1406, found: 323.1404.

$[\alpha]^{25}_{D}$ = +192.4 (*c* 1.0, CH_2Cl_2); 92% ee from (*S*)-**L**.

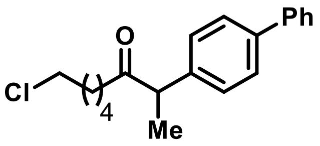

2-([1,1'-Biphenyl]-4-yl)-8-chlorooctan-3-one (Fig. 2, compound 7). The title compound was synthesized according to **GP-B** from 6-chlorohexanoic acid and 4-ethylbiphenyl. The product was purified by column chromatography on silica gel (1:20 EtOAc/Petroleum ether). Colorless oil.

(*S*)-L: 136 mg, 86% yield, 93% ee; (*R*)-**L**: 136 mg, 87% yield, 93% ee.

HPLC analysis: The ee was determined via HPLC on a CHIRALCEL OJ-3 column (15.0% 2-PrOH in hexanes, 1.0 mL/min); retention times for compound obtained using (*S*)-**L**: 9.9 min (minor), 12.5 min (major).

^{1}H NMR (500 MHz, $CDCl_3$) δ 7.61 – 7.56 (m, 4H), 7.46 – 7.43 (m, 2H), 7.37 – 7.34 (m, 1H), 7.31 – 7.28 (m, 2H), 3.80 (q, *J* = 7.0 Hz, 1H), 3.47 (t, *J* = 6.7 Hz, 2H), 2.49 – 2.37 (m, 2H), 1.72 – 1.66 (m, 2H), 1.59 – 1.52 (m, 2H), 1.44 (d, *J* = 7.0 Hz, 3H), 1.36 – 1.28 (m, 2H).

^{13}C NMR (126 MHz, $CDCl_3$) δ 210.4, 140.5, 140.0, 139.5, 128.7, 128.2, 127.5, 127.3, 126.9, 52.6, 44.7, 40.7, 32.2, 26.2, 23.0, 17.4.

FT-IR (film): 2921, 2851, 1712, 1659, 1633, 1486, 1409, 1180, 1141, 1076, 764, 697 cm^{-1}.

HRMS (ESI-MS) *m/z* $[M+Na]^+$ calcd for $C_{20}H_{23}ClONa$: 337.1330, found: 337.1327.

$[\alpha]^{25}_{D}$ = +148.4 (*c* 1.0, CH_2Cl_2); 93% ee from (*S*)-**L**.

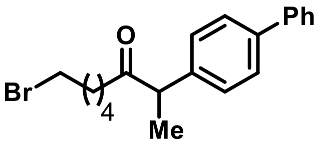

2-([1,1'-Biphenyl]-4-yl)-8-bromooctan-3-one (Fig. 2, compound 8). The title compound was synthesized according to **GP-B** from 6-bromohexanoic acid and 4-ethylbiphenyl. The product was purified by column chromatography on silica gel (1:150 EtOAc/Petroleum ether). White solid (mp = 43-45 °C).

(*S*)-**L**: 160 mg, 89% yield, 93% ee; (*R*)-**L**: 160 mg, 89% yield, 93% ee.

HPLC analysis: The ee was determined via HPLC on a CHIRALCEL OJ-3 column (15.0% 2-PrOH in hexanes, 1.0 mL/min); retention times for compound obtained using (*S*)-**L**: 10.4 min (minor), 13.2 min (major).

^{1}H NMR (600 MHz, $CDCl_3$) δ 7.62 – 7.58 (m, 4H), 7.47 – 7.44 (m, 2H), 7.37 – 7.34 (m, 1H), 7.32 – 7.30 (m, 2H), 3.81 (q, *J* = 6.9 Hz, 1H), 3.34 (t, *J* = 6.8 Hz, 2H), 2.49 – 2.38 (m, 2H), 1.80 – 1.75 (m, 2H), 1.60 – 1.52 (m, 2H), 1.45 (d, *J* = 7.0 Hz, 3H), 1.37 – 1.30 (m, 2H).

^{13}C NMR (126 MHz, $CDCl_3$) δ 210.2, 140.4, 139.9, 139.5, 128.7, 128.1, 127.5, 127.2, 126.9, 52.5, 40.5, 33.5, 32.3, 27.4, 22.7, 17.3.

FT-IR (film): 2923, 1711, 1558, 1485, 14566, 839, 763, 729, 696 cm^{-1}.

HRMS (ESI-MS) *m/z* $[M+Na]^+$ calcd for $C_{20}H_{23}BrONa$: 381.0824, found: 381.0819.

$[\alpha]^{25}_{D}$ = +128.8 (*c* 1.0, CH_2Cl_2); 93% ee from (*S*)-**L**.

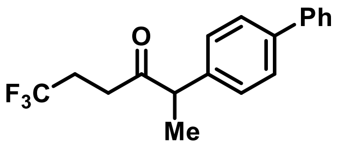

2-([1,1'-Biphenyl]-4-yl)-6,6,6-trifluorohexan-3-one (Fig. 2, compound 9). The title compound was synthesized according to **GP-B** from 4,4,4-trifluorobutanoic acid and 4-ethylbiphenyl. The product was purified by column chromatography on silica gel (1:30 EtOAc/Petroleum ether). Yellow oil.

(*S*)-**L**: 117 mg, 76% yield, 95% ee; (*R*)-**L**: 114 mg, 74% yield, 97% ee.

HPLC analysis: The ee was determined via HPLC on a CHIRALCEL OJ-3 column (15.0% 2-PrOH in hexanes, 1.0 mL/min); retention times for compound obtained using (*S*)-**L**: 8.5 min (minor), 9.5 min (major).

^{1}H NMR (500 MHz, $CDCl_3$) δ 7.61 – 7.58 (m, 4H), 7.48 – 7.44 (m, 2H), 7.39 – 7.35 (m, 1H), 7.31 – 7.29 (m, 2H), 3.83 (q, *J* = 7.0 Hz, 1H), 2.74 – 2.61 (m, 2H), 2.48 – 2.39 (m, 1H), 2.38 – 2.27 (m, 1H), 1.48 (d, *J* = 7.0 Hz, 3H).

^{19}F NMR (471 MHz, $CDCl_3$) δ −66.5 (s, 3F).

^{13}C NMR (126 MHz, $CDCl_3$) δ 207.1, 140.4, 138.9, 128.8, 128.1, 127.8, 127.4, 127.0, 126.9 (q, J_{C-F} = 276.4 Hz), 52.7, 33.3 (q, J_{C-F} = 2.5 Hz), 28.1 (q, J_{C-F} = 29.9 Hz), 17.3.

FT-IR (film): 2985, 2919, 1716, 1454, 1299, 1254, 1147, 1093, 837, 761, 726, 691 cm^{-1}.

HRMS (ESI-MS) *m/z* $[M+Na]^+$ calcd for $C_{18}H_{17}F_3ONa$: 329.1124, found: 329.1120.

$[\alpha]^{25}_{D}$ = +150.1 (*c* 1.0, CH_2Cl_2); 95% ee from (*S*)-**L**.

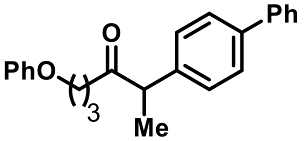

2-([1,1'-Biphenyl]-4-yl)-6-phenoxyhexan-3-one (Fig. 2, compound 10). The title compound was synthesized according to **GP-B** from 4-phenoxybutanoic acid and 4-ethylbiphenyl. The product was purified by column chromatography on silica gel (1:60 EtOAc/Petroleum ether). White solid (mp = 75-77 °C).

(*S*)-**L**: 148 mg, 86% yield, 94% ee; (*R*)-**L**: 142 mg, 83% yield, 93% ee.

HPLC analysis: The ee was determined via HPLC on a CHIRALPAK AD-3 column (5.0% 2-PrOH in hexanes, 1.0 mL/min); retention times for compound obtained using (*S*)-**L**: 8.8 min (major), 9.3 min (minor).

^{1}H NMR (400 MHz, $CDCl_3$) δ 7.60 – 7.55 (m, 4H), 7.48 – 7.44 (m, 2H), 7.39 – 7.35 (m, 1H), 7.32 – 7.30 (m, 2H), 7.27 – 7.23 (m, 2H), 6.93 (t, *J* = 7.3 Hz, 1H), 6.83 (d, *J* = 8.0 Hz, 2H), 3.95 – 3.83 (m, 3H), 2.65 (td, *J* = 7.2, 3.1 Hz, 2H), 2.10 – 1.97 (m, 2H), 1.47 (d, *J* = 7.0 Hz, 3H).

^{13}C NMR (126 MHz, $CDCl_3$) δ 210.2, 158.7, 140.5, 140.1, 139.5, 129.3, 128.7, 128.2, 127.6, 127.3, 127.0, 120.6, 114.4, 66.5, 52.7, 37.3, 23.5, 17.4.

FT-IR (film): 3028, 2921, 2850, 1713, 1600, 1586, 1497, 1245, 1039, 841, 754, 692 cm^{-1}.

HRMS (ESI-MS) *m/z* $[M+Na]^+$ calcd for $C_{24}H_{24}O_2Na$: 367.1669, found: 367.1665.

$[\alpha]^{25}_{D}$ = +110.0 (*c* 1.0, CH_2Cl_2); 94% ee from (*S*)-**L**.

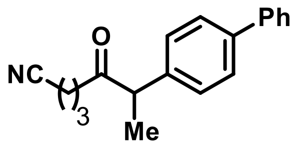

6-([1,1'-Biphenyl]-4-yl)-5-oxoheptanenitrile (Fig. 2, compound 11). The title compound was synthesized according to **GP-B** from 4-cyanobutanoic acid and 4-ethylbiphenyl. The product was purified by column chromatography on silica gel (1:10 EtOAc/Petroleum ether). White solid (mp = 83-85 °C).

(*S*)-**L**: 122 mg, 88% yield, 94% ee; (*R*)-**L**: 125 mg, 90% yield, 94% ee.

HPLC analysis: The ee was determined via HPLC on a CHIRALPAK AD-3 column (5.0% 2-PrOH in hexanes, 1.0 mL/min); retention times for compound obtained using (*S*)-**L**: 14.6 min (minor), 15.6 min (major).

^{1}H NMR (400 MHz, $CDCl_3$) δ 7.60 – 7.57 (m, 4H), 7.44 (t, *J* = 7.3 Hz, 2H), 7.37 – 7.33 (m, 1H), 7.28 (d, *J* = 8.2 Hz, 2H), 3.81 (q, *J* = 6.9 Hz, 1H), 2.66 – 2.52 (m, 2H), 2.40 – 2.23 (m, 2H), 1.93 – 1.80 (m, 2H), 1.45 (d, *J* = 7.0 Hz, 3H).

^{13}C NMR (101 MHz, $CDCl_3$) δ 209.0, 140.44, 140.36, 139.0, 128.8, 128.2, 127.8, 127.4, 127.0, 119.1, 52.7, 38.7, 19.5, 17.2, 16.3.

FT-IR (film): 2928, 1711, 1450, 1366, 836, 757, 725, 688 cm^{-1}.

HRMS (ESI-MS) *m/z* $[M+Na]^+$ calcd for $C_{19}H_{19}NONa$: 300.1359, found: 300.1355.

$[\alpha]^{25}_{D}$ = +157.7 (*c* 1.0, CH_2Cl_2); 94% ee from (*S*)-**L**.

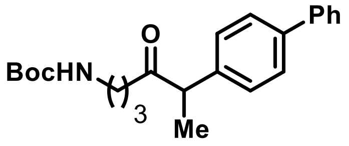

***tert*-Butyl (5-([1,1'-biphenyl]-4-yl)-4-oxohexyl)carbamate (Fig. 2, compound 12).** The title compound was synthesized according to **GP-B** from 4-((*tert*-butoxycarbonyl)amino)butanoic acid and 4-ethylbiphenyl. The product was purified by column chromatography on silica gel (1:8 EtOAc/Petroleum ether). White solid (mp = 94-95 °C).

(*S*)-**L**: 165 mg, 90% yield, 92% ee; (*R*)-**L**: 170 mg, 92% yield, 93% ee.

HPLC analysis: The ee was determined via HPLC on a CHIRALCEL OJ-3 column (15.0% 2-PrOH in hexanes, 1.0 mL/min); retention times for compound obtained using (*S*)-**L**: 11.6 min (minor), 12.7 min (major).

^{1}H NMR (500 MHz, $CDCl_3$) δ 7.58 – 7.54 (m, 4H), 7.45 – 7.41 (m, 2H), 7.36 – 7.32 (m, 1H), 7.29 – 7.26 (m, 2H), 4.51 (s, 1H), 3.80 (q, *J* = 7.0 Hz, 1H), 3.07 – 2.97 (m, 2H), 2.46 – 2.42 (m, 2H), 1.76 – 1.63 (m, 2H), 1.43 (d, *J* = 7.0 Hz, 3H), 1.40 (s, 9H).

^{13}C NMR (126 MHz, $CDCl_3$) δ 210.2, 155.9, 140.5, 140.1, 139.4, 128.7, 128.2, 127.6, 127.3, 126.9, 79.0, 52.6, 39.8, 38.0, 28.3, 24.1, 17.3.

FT-IR (film): 2973, 2929, 1710, 1515, 1487, 1449, 1365, 1249, 1168, 764, 697 cm^{-1}.

HRMS (ESI-MS) *m/z* $[M+Na]^+$ calcd for $C_{23}H_{29}NO_3Na$: 390.2040, found: 390.2035.

$[\alpha]^{25}_{D}$ = +123.3 (*c* 1.0, CH_2Cl_2); 92% ee from (*S*)-**L**.

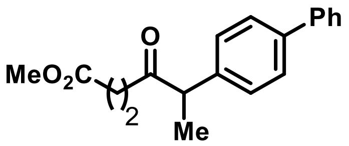

Methyl 5-([1,1'-biphenyl]-4-yl)-4-oxohexanoate (Fig. 2, compound 13). The title compound was synthesized according to **GP-B** from 4-methoxy-4-oxobutanoic acid and 4-ethylbiphenyl. The product was purified by column chromatography on silica gel (1:15 EtOAc/Petroleum ether). White solid (mp = 75-76 °C).

(*S*)-**L**: 118 mg, 80% yield, 95% ee; (*R*)-**L**: 116 mg, 78% yield, 95% ee.

HPLC analysis: The ee was determined via HPLC on a CHIRALCEL OJ-3 column (15.0% 2-PrOH in hexanes, 1.0 mL/min); retention times for compound obtained using (*S*)-**L**: 25.8 min (major), 28.0 min (minor).

^{1}H NMR (400 MHz, $CDCl_3$) δ 7.59 – 7.55 (m, 4H), 7.46 – 7.41 (m, 2H), 7.37 – 7.32 (m, 1H), 7.31 – 7.28 (m, 2H), 3.85 (q, *J* = 7.0 Hz, 1H), 3.65 (s, 3H), 2.78 – 2.56 (m, 3H), 2.51 – 2.44 (m, 1H), 1.46 (d, *J* = 7.0 Hz, 3H).

^{13}C NMR (126 MHz, $CDCl_3$) δ 208.9, 173.2, 140.6, 140.2, 139.5, 128.7, 128.3, 127.6, 127.3, 127.0, 52.6, 51.7, 35.7, 28.0, 17.4.

FT-IR (film): 2920, 1736, 1713, 1485, 1434, 1196, 1142, 1132, 1075, 1021, 843, 764, 732, 697 cm^{-1}.

HRMS (ESI-MS) *m/z* $[M+Na]^+$ calcd for $C_{19}H_{20}O_3Na$: 319.1305, found: 319.1300.

$[\alpha]^{25}_{D}$ = +165.5 (*c* 1.0, CH_2Cl_2); 95% ee from (*S*)-**L**.

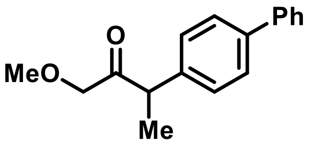

3-([1,1'-Biphenyl]-4-yl)-1-methoxybutan-2-one (Fig. 2, compound 14). The title compound was synthesized according to **GP-B** from 2-methoxyacetic acid and 4-ethylbiphenyl. The product was purified by column chromatography on silica gel (1:5 EtOAc/Petroleum ether). White solid (mp = 67-68 °C).

(*S*)-**L**: 69mg, 54% yield, 94% ee; (*R*)-**L**: 68 mg, 53% yield, 93% ee.

HPLC analysis: The ee was determined via HPLC on a CHIRALCEL OJ-3 column (15.0% 2-PrOH in hexanes, 1.0 mL/min); retention times for compound obtained using (*S*)-**L**: 11.6 min (minor), 17.1 min (major).

^{1}H NMR (400 MHz, $CDCl_3$) δ 7.59 – 7.55 (m, 4H), 7.46 – 7.42 (m, 2H), 7.37 – 7.30 (m, 3H), 4.05 (s, 2H), 3.96 (q, *J* = 7.0 Hz, 1H), 3.33 (s, 3H), 1.46 (d, *J* = 7.0 Hz, 3H)

^{13}C NMR (101 MHz, $CDCl_3$) δ 207.9, 140.5, 140.2, 138.9, 128.7, 128.2, 127.6, 127.3, 127.0, 76.2, 59.2, 48.5, 17.2.

FT-IR (film): 2980, 2931, 1725, 1408, 1204, 1125, 1111, 1044, 837, 762, 727, 690 cm^{-1}.

HRMS (ESI-MS) *m/z* $[M+Na]^+$ calcd for $C_{17}H_{18}O_2Na$: 277.1199, found: 277.1196.

$[\alpha]^{25}_{D}$ = +188.2 (*c* 1.0, CH_2Cl_2); 94% ee from (*S*)-**L**.

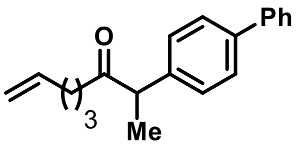

2-([1,1'-Biphenyl]-4-yl)oct-7-en-3-one (Fig. 2, compound 15). The title compound was synthesized according to **GP-B** from hex-5-enoic acid and 4-ethylbiphenyl. The product was purified by column chromatography on silica gel (1:60 EtOAc/Petroleum ether). White solid (mp = 43-45 °C).

(*S*)-**L**: 79 mg, 57% yield, 93% ee; (*R*)-**L**: 79 mg, 57% yield, 93% ee.

HPLC analysis: The ee was determined via HPLC on a CHIRALCEL OJ-3 column (5.0% 2-PrOH in hexanes, 1.0 mL/min); retention times for compound obtained using (*S*)-**L**: 13.0 min (minor), 16.8 min (major).

^{1}H NMR (400 MHz, $CDCl_3$) δ 7.62 – 7.57 (m, 4H), 7.48 – 7.43 (m, 2H), 7.38 – 7.34 (m, 1H), 7.32 – 7.29 (m, 2H), 5.76 – 5.66 (m, 1H), 4.96 – 4.94 (m, 1H), 4.93 (t, *J* = 1.5 Hz, 1H), 3.82 (q, *J* = 6.9 Hz, 1H), 2.50 – 2.37 (m, 2H), 2.06 – 1.91 (m, 2H), 1.72 – 1.59 (m, 2H), 1.45 (d, *J* = 7.0 Hz, 3H).

^{13}C NMR (101 MHz, $CDCl_3$) δ 210.5, 140.6, 140.0, 139.6, 137.9, 128.7, 128.2, 127.5, 127.3, 126.9, 115.0, 52.6, 40.2, 32.9, 22.8, 17.4.

FT-IR (film): 3028, 2973, 2928, 1713, 1639, 1486, 1195, 1180, 910, 764, 697 cm^{-1}.

HRMS (ESI-MS) *m/z* $[M+Na]^+$ calcd for $C_{20}H_{22}ONa$: 301.1563, found: 301.1559.

$[\alpha]^{25}_{D}$ = –158.8 (*c* 1.0, CH_2Cl_2); 93% ee from (*R*)-**L**.

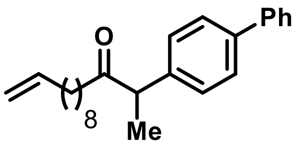

2-([1,1'-Biphenyl]-4-yl)tridec-12-en-3-one (Fig. 2, compound 16). The title compound was synthesized according to **GP-B** from undec-10-enoic acid and 4-ethylbiphenyl. The product was purified by column chromatography on silica gel (1:50 EtOAc/Petroleum ether). White solid (mp = 30-31 °C).

(*S*)-**L**: 74 mg, 42% yield, 94% ee; (*R*)-**L**: 74 mg, 43% yield, 93% ee.

HPLC analysis: The ee was determined via HPLC on a CHIRALCEL OJ-3 column (15.0% 2-PrOH in hexanes, 1.0 mL/min); retention times for compound obtained using (*S*)-**L**: 4.8 min (minor), 5.6 min (major).

^{1}H NMR (400 MHz, $CDCl_3$) δ 7.60 – 7.55 (m, 4H), 7.46 – 7.42 (m, 2H), 7.37 – 7.33 (m, 1H), 7.30 – 7.28 (m, 2H), 5.84 – 5.74 (m, 1H), 5.00 – 4.90 (m, 2H), 3.80 (q, *J* = 7.0 Hz, 1H), 2.42 – 2.37 (m, 2H), 2.04 – 1.98 (m, 2H), 1.55 – 1.49 (m, 2H), 1.43 (d, *J* = 7.0 Hz, 3H), 1.35 – 1.30 (m, 2H), 1.25 – 1.19 (m, 8H).

^{13}C NMR (126 MHz, $CDCl_3$) δ 210.9, 140.6, 140.0, 139.7, 139.1, 128.7, 128.3, 127.5, 127.3, 127.0, 114.1, 52. 6, 41.1, 33.7, 29.2, 29.0, 28.8, 23.8, 17.5.

FT-IR (film): 2925, 2853, 1713, 1486, 1452, 1007, 907, 841, 763, 696 cm^{-1}.

HRMS (ESI-MS) *m/z* $[M+Na]^+$ calcd for $C_{25}H_{32}ONa$: 371.2345, found: 371.2350.

$[\alpha]^{25}_{D}$ = +130.0 (*c* 1.0, CH_2Cl_2); 94% ee from (*S*)-**L**.

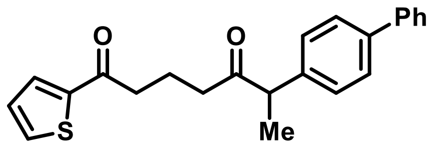

6-([1,1'-Biphenyl]-4-yl)-1-(thiophen-2-yl)heptane-1,5-dione (Fig. 2, compound 17). The title compound was synthesized according to **GP-B** from 5-oxo-5-(thiophen-2-yl)pentanoic acid and 4-ethylbiphenyl. The product was purified by column chromatography on silica gel (1:15 EtOAc/Petroleum ether). Yellow solid (mp = 102-104 °C).

(*S*)-**L**: 136mg, 75% yield, 93% ee; (*R*)-**L**: 135 mg, 74% yield, 94% ee.

HPLC analysis: The ee was determined via HPLC on a CHIRALPAK AD-3 column (10.0% 2-PrOH in hexanes, 1.0 mL/min); retention times for compound obtained using (*S*)-**L**: 13.7 min (major), 14.3 min (minor).

^{1}H NMR (400 MHz, $CDCl_3$) δ 7.62 (dd, *J* = 3.8, 1.1 Hz, 1H), 7.57 – 7.56 (m, 2H), 7.55 – 7.54 (m, 2H), 7.52 (t, *J* = 2.0 Hz, 1H), 7.45 – 7.41 (m, 2H), 7.36 – 7.32 (m, 1H), 7.29 – 7.27 (m, 2H), 7.05 (dd, *J* = 5.0, 3.8 Hz, 1H), 3.81 (q, *J* = 6.9 Hz, 1H), 2.88 – 2.73 (m, 2H), 2.55 (td, *J* = 7.1, 1.6 Hz, 2H), 2.01 – 1.93 (m, 2H), 1.44 (d, *J* = 7.0 Hz, 3H).

^{13}C NMR (126 MHz, $CDCl_3$) δ 210.2, 192.6, 144.1, 140.5, 140.0, 139.3, 133.4, 131.8, 128.7, 128.2, 128.0, 127.5, 127.3, 126.9, 52.6, 39.8, 38.0, 18.6, 17.3.

FT-IR (film): 2922, 1707, 1654, 1484, 1412, 1384, 1196, 1180, 1142, 1022, 763, 695 cm^{-1}.

HRMS (ESI-MS) *m/z* $[M+Na]^+$ calcd for $C_{23}H_{22}O_2SNa$: 385.1233, found: 385.1228.

$[\alpha]^{25}_D$ = +132.2 (*c* 1.0, CH_2Cl_2); 93% ee from (*S*)-**L**.

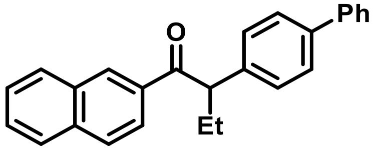

2-([1,1'-Biphenyl]-4-yl)-1-(naphthalen-2-yl)butan-1-one (Fig. 2, compound 18). The title compound was synthesized according to **GP-D** from 2-naphthoic acid and 4-propylbiphenyl. The product was purified by preparative thin-layer chromatography on silica gel (1:30 EtOAc/Petroleum ether). White solid (mp = 112-115 °C).

(*S, R*)-**L3**: 66 mg, 37% yield, 69% ee; (*R, S*)-**L3**: 54 mg, 31% yield, 71% ee.

HPLC analysis: The ee was determined via HPLC on a CHIRALCEL OD-3 column (1.0% 2-PrOH in hexanes, 1.0 mL/min); retention times for compound obtained using (*S, R*)-**L**: 12.9 min (major), 14.3 min (minor).

^{1}H NMR (400 MHz, $CDCl_3$) δ 8.56 (s, 1H), 8.08 (dd, *J* = 8.6, 1.8 Hz, 1H), 7.95 (d, *J* = 8.2 Hz, 1H), 7.87 – 7.82 (m, 2H), 7.59 – 7.51 (m, 6H), 7.48 – 7.45 (m, 2H), 7.43 – 7.39 (m, 2H), 7.34 – 7.30 (m, 1H), 4.69 (t, *J* = 7.3 Hz, 1H), 2.37 – 2.28 (m, 1H), 2.04 – 1.94 (m, 1H), 1.01 (t, *J* = 7.4 Hz, 3H).

^{13}C NMR (126 MHz, $CDCl_3$) δ 120.0, 140.6, 139.8, 138.7, 135.4, 134.3, 132.4, 130.3, 129.6, 128.7, 128.6, 128.4, 128.3, 127.65, 127.55, 127.2, 126.9, 126.6, 124.4, 55.1, 27.2, 12.4.

FT-IR (film): 2922, 2851, 1670, 1483, 1274, 756, 969 cm^{-1}.

HRMS (ESI-MS) *m/z* $[M+Na]^+$ calcd for $C_{26}H_{22}ONa$: 373.1563, found: 373.1559.

$[\alpha]^{25}_D$ = +113.3 (*c* 1.0, CH_2Cl_2); 71% ee from (*R, S*)-**L**.

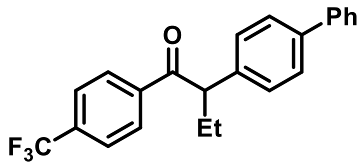

2-([1,1'-Biphenyl]-4-yl)-1-(4-(trifluoromethyl)phenyl)butan-1-one (Fig. 2, compound 19). The title compound was synthesized according to **GP-D** from 4-(trifluoromethyl)benzoic acid and 4-propylbiphenyl. The product was purified by preparative thin-layer chromatography on silica gel (1:30 EtOAc/Petroleum ether). Colorless oil.

(*S, R*)-**L3**: 78 mg, 42% yield, 76% ee; (*R, S*)-**L3**: 88 mg, 46% yield, 77% ee.

HPLC analysis: The ee was determined via HPLC on a CHIRALPAK AD-3 column (1.0% 2-PrOH in hexanes, 1.0 mL/min); retention times for compound obtained using (*S*)-**L**: 12.4 min (major), 19.0 min (minor).

^{1}H NMR (500 MHz, $CDCl_3$) δ 8.08 (d, *J* = 8.2 Hz, 2H), 7.67 (d, *J* = 8.3 Hz, 2H), 7.56 – 7.53 (m, 4H), 7.42 (t, *J* = 7.7 Hz, 2H), 7.36 – 7.31 (m, 3H), 4.47 (t, *J* = 7.2 Hz, 1H), 2.28 – 2.23 (m, 1H), 1.95 – 1.89 (m, 1H), 0.96 (t, *J* = 7.4 Hz, 3H).

^{19}F NMR (471 MHz, $CDCl_3$) δ -63.1 (s, 3F).

^{13}C NMR (101 MHz, $CDCl_3$) δ 199.0, 140.4, 140.2, 139.7, 137.9, 134.0 (q, $J_{C\text{-}F}$ = 32.8 Hz), 128.9, 128.7, 128.6, 127.7, 127.3, 127.0, 125.6 (q, $J_{C\text{-}F}$ = 3.8 Hz), 123.5 (q, $J_{C\text{-}F}$ = 273.7 Hz), 55.6, 26.9, 12.2.

FT-IR (film): 2964, 2925, 1687, 1485, 1408, 1323, 1129, 1066, 759, 696 cm^{-1}.

HRMS (ESI-MS) *m/z* $[M+Na]^+$ calcd for $C_{23}H_{19}F_3ONa$: 391.1280, found: 391.1277.

$[\alpha]^{25}_{D}$ = –72.4 (*c* 1.0, CH_2Cl_2); 77% ee from (*R, S*)-**L**.

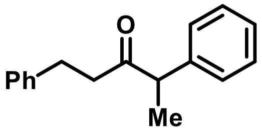

1,4-Diphenylpentan-3-one (Fig. 2, compound 20). The title compound was synthesized according to **GP-B** from 3-phenylpropanoic acid and ethylbenzene. The product was purified by column chromatography on silica gel (1:90 EtOAc/Petroleum ether). Colorless oil.

(*S*)-**L**: 105 mg, 88% yield, 93% ee; (*R*)-**L**: 100 mg, 84% yield, 93% ee.

HPLC analysis: The ee was determined via HPLC on a CHIRALCEL OD-3 column (5.0% 2-PrOH in hexanes, 1.0 mL/min); retention times for compound obtained using (*S*)-**L**: 5.7 min (major), 6.2 min (minor).

^{1}H NMR (500 MHz, $CDCl_3$) δ 7.33 – 7.29 (m, 2H), 7.27 – 7.21 (m, 3H), 7.19 – 7.15 (m, 3H), 7.09 – 7.06 (m, 2H), 3.72 (q, *J* = 7.0 Hz, 1H), 2.90 – 2.84 (m, 1H), 2.82 – 2.76 (m, 1H), 2.74 – 2.62 (m, 2H), 1.40 (d, *J* = 7.0 Hz, 3H).

^{13}C NMR (126 MHz, $CDCl_3$) δ 209.8, 141.0, 140.4, 128.9, 128.3, 128.2, 127.8, 127.1, 125.9, 53.2, 42.5, 29.9, 17.3.

FT-IR (film): 3027, 2974, 1713, 1601, 1494, 1453, 1373, 1073, 1029, 751, 699, cm^{-1}.

HRMS (ESI-MS) *m/z* $[M+Na]^+$ calcd for $C_{17}H_{18}ONa$: 261.1250, found: 261.1252.

$[\alpha]^{25}_{D}$ = –142.5 (*c* 1.0, CH_2Cl_2); 93% ee from (*R*)-**L**.

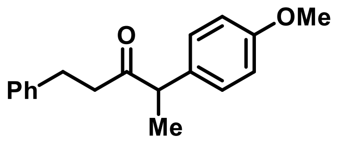

4-(4-Methoxyphenyl)-1-phenylpentan-3-one (Fig. 2, compound 21). The title compound was synthesized according to **GP-B** from 3-phenylpropanoic acid and 4-(4-methoxyphenyl)-1-phenylpentan-3-one. The product was purified by column chromatography on silica gel (1:5 EtOAc/Petroleum ether). Colorless oil.

(*S*)-**L**: 105 mg, 78% yield, 95% ee; (*R*)-**L**: 107 mg, 80% yield, 95% ee.

HPLC analysis: The ee was determined via HPLC on a CHIRALCEL OJ-3 column (15.0% 2-PrOH in hexanes, 1.0 mL/min); retention times for compound obtained using (*S*)-**L**: 13.8 min (minor), 15.4 min (major).

^{1}H NMR (500 MHz, CD_2Cl_2) δ 7.25 – 7.21 (m, 2H), 7.18 – 7.14 (m, 1H), 7.12 – 7.07 (m, 4H), 6.87 – 6.84 (m, 2H), 3.79 (s, 3H), 3.69 (q, *J* = 6.9 Hz, 1H), 2.87 – 2.63 (m, 4H), 1.35 (d, *J* = 7.0 Hz, 3H).

^{13}C NMR (126 MHz, CD_2Cl_2) δ 210.2, 159.2, 141.7, 133.0, 129.3, 128.7, 128.6, 126.3, 114.6, 55.6, 52.5, 42.7, 30.2, 17.6.

FT-IR (film): 3027, 2931, 1712, 1609, 1510, 1454, 1247, 1178, 1032, 832, 783, 699 cm^{-1}.

HRMS (ESI-MS) *m/z* $[M+Na]^+$ calcd for $C_{18}H_{20}O_2Na$: 291.1356, found: 291.1352.

$[\alpha]^{25}_{D}$ = +125.9 (*c* 1.0, CH_2Cl_2); 95% ee from (*S*)-**L**.

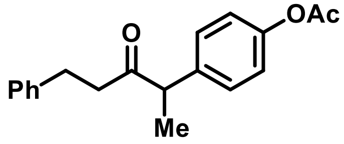

4-(3-Oxo-5-phenylpentan-2-yl)phenyl acetate (Fig. 2, compound 22). The title compound was synthesized according to **GP-B** from 3-phenylpropanoic acid and 4-ethylphenyl acetate. The product was purified by column chromatography on silica gel (1:5 EtOAc/Petroleum ether). Colorless oil.

(*S*)-**L**: 115 mg, 78% yield, 97% ee; (*R*)-**L**: 119 mg, 80% yield, 97% ee.

HPLC analysis: The ee was determined via HPLC on a CHIRALCEL OJ-3 column (15.0% 2-PrOH in hexanes, 1.0 mL/min); retention times for compound obtained using (*S*)-**L**: 23.3 min (minor), 24.1 min (major).

^{1}H NMR (500 MHz, $CDCl_3$) δ 7.25 – 7.21 (m, 2H), 7.18 – 7.14 (m, 3H), 7.09 – 7.07 (m, 2H), 7.04 – 7.01 (m, 2H), 3.71 (q, *J* = 7.0 Hz, 1H), 2.89 – 2.75 (m, 2H), 2.73 – 2.63 (m, 2H), 2.29 (s, 3H), 1.37 (d, *J* = 7.0 Hz, 3H).

^{13}C NMR (126 MHz, $CDCl_3$) δ 209.5, 169.3, 149.7, 140.9, 137.8, 128.8, 128.4, 128.2, 126.0, 121.9, 52.4, 42.6, 29.9, 21.1, 17.4.

FT-IR (film): 2929, 1757, 1711, 1516, 1452, 1368, 1197, 1076, 1016, 909, 744, 698 cm^{-1}.

HRMS (ESI-MS) *m/z* $[M+Na]^+$ calcd for $C_{19}H_{20}O_3Na$: 319.1305, found: 319.1302.

$[\alpha]^{25}_{D}$ = +98.3 (*c* 1.0, CH_2Cl_2); 97% ee from (*S*)-**L**.

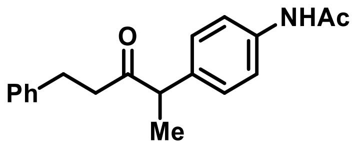

***N*-(4-(3-Oxo-5-phenylpentan-2-yl)phenyl)acetamide (Fig. 2, compound 23).** The title compound was synthesized according to **GP-B** from 3-phenylpropanoic acid and *N*-(4-ethylphenyl)acetamide. The product was purified by column chromatography on silica gel (2:3 EtOAc/Petroleum ether). Yellow oil.

(*S*)-**L**: 96 mg, 65% yield, 95% ee; (*R*)-**L**: 104 mg, 70% yield, 95% ee.

HPLC analysis: The ee was determined via HPLC on a CHIRALPAK AD-3 column (10.0% 2-PrOH in hexanes, 1.0 mL/min); retention times for compound obtained using (*S*)-**L**: 27.4 min (minor), 30.1 min (major).

^{1}H NMR (400 MHz, $CDCl_3$) δ 7.45 – 7.40 (m, 3H), 7.24 – 7.20 (m, 2H), 7.17 – 7.15 (m, 1H), 7.12 – 7.05 (m, 4H), 3.67 (q, *J* = 6.9 Hz, 1H), 2.88 – 2.71 (m, 2H), 2.69 – 2.63 (m, 2H), 2.16 (s, 3H), 1.34 (d, *J* = 6.9 Hz, 3H).

^{13}C NMR (126 MHz, $CDCl_3$) δ 209.9, 168.3, 140.9, 136.9, 136.2, 128.4, 128.2, 126.0, 120.3, 52.5, 42.5, 29.9, 24.5, 17.3.

FT-IR (film): 3027, 2972, 1709, 1666, 1602, 1534, 1411, 1316, 1267, 837, 747, 700, cm^{-1}.

HRMS (ESI-MS) *m*/*z* $[M+ Na]^+$ calcd for $C_{19}H_{21}NO_2Na$: 318.1465, found: 318.1462.

$[\alpha]^{25}_D$ = +86.9 (*c* 1.0, CH_2Cl_2); 95% ee from (*S*)-**L**.

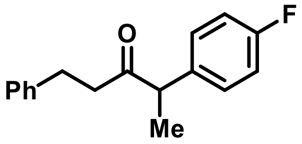

4-(4-Fluorophenyl)-1-phenylpentan-3-one (Fig. 2, compound 24). The title compound was synthesized according to **GP-B** from 3-phenylpropanoic acid and 1-ethyl-4-fluorobenzene. The product was purified by column chromatography on silica gel (1:30 EtOAc/Petroleum ether). Colorless oil.

(*S*)-**L**: 97 mg, 75% yield, 93% ee; (*R*)-**L**: 98 mg, 77% yield, 93% ee.

HPLC analysis: The ee was determined via HPLC on a CHIRALCEL OJ-3 column (15.0% 2-PrOH in hexanes, 1.0 mL/min); retention times for compound obtained using (*S*)-**L**: 7.9 min (minor), 9.4 min (major).

^{1}H NMR (500 MHz, $CDCl_3$) δ 7.25 – 7.22 (m, 2H), 7.19 – 7.16 (m, 1H), 7.13 – 7.11 (m, 2H), 7.09 – 7.07 (m, 2H), 7.00 – 6.97 (m, 2H), 3.71 (q, *J* = 7.0 Hz, 1H), 2.90 – 2.77 (m, 2H), 2.74 – 2.64 (m, 2H), 1.37 (d, *J* = 7.0 Hz, 3H).

^{19}F NMR (471 MHz, $CDCl_3$) δ −115.5 (s, 1F).

^{13}C NMR (126 MHz, $CDCl_3$) δ 209.5, 161.9 (d, J_{C-F} = 246.0 Hz), 140.9, 136.0 (d, J_{C-F} = 3.3 Hz), 129.3 (d, J_{C-F} = 8.1 Hz), 128.4, 128.2, 126.0, 115.7 (d, J_{C-F} = 21.4 Hz), 52.3, 42.5, 29.9, 17.4.

FT-IR (film): 3027, 2931, 1714, 1602, 1508, 1224, 1159, 1076, 836, 749, 699 cm^{-1}.

HRMS (ESI-MS) *m*/*z* $[M+Na]^+$ calcd for $C_{17}H_{17}FONa$: 279.1156, found: 279.1152.

$[\alpha]^{25}_D$ = +110.9 (*c* 1.0, CH_2Cl_2); 93% ee from (*S*)-**L**.

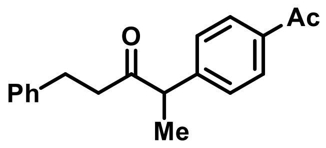

4-(4-Acetylphenyl)-1-phenylpentan-3-one (Fig. 2, compound 25). The title compound was synthesized according to **GP-B** from 3-phenylpropanoic acid and 1-(4-ethylphenyl)ethan-1-one. The product was purified by column chromatography on silica gel (1:10 EtOAc/Petroleum ether). Colorless oil.

(*S*)-**L**: 95 mg, 68% yield, 92% ee; (*R*)-**L**: 94 mg, 67% yield, 90% ee.

HPLC analysis: The ee was determined via HPLC on a CHIRALCEL OD-3 column (10.0% 2-PrOH in hexanes, 1.0 mL/min); retention times for compound obtained using (*S*)-**L**: 8.6 min (major), 10.3 min (minor).

^{1}H NMR (400 MHz, $CDCl_3$) δ 7.89 – 7.86 (m, 2H), 7.26 – 7.18 (m, 4H), 7.17 – 7.12 (m, 1H), 7.07 – 7.03 (m, 2H), 3.78 (q, *J* = 7.0 Hz, 1H), 2.89 – 2.74 (m, 2H), 2.70 – 2.66 (m, 2H), 2.58 (s, 3H), 1.39 (d, *J* = 7.0 Hz, 3H).

^{13}C NMR (126 MHz, $CDCl_3$) δ 208.8, 197.5, 145.6, 140.7, 136.0, 128.9, 128.3, 128.2, 128.0, 126.0, 53.0, 42.8, 29.8, 26.5, 17.2.

FT-IR (film): 3027, 2931, 1715, 1682, 1605, 1412, 1358, 1267, 1057, 837, 700, 598 cm^{-1}.

HRMS (ESI-MS) *m/z* $[M+Na]^+$ calcd for $C_{19}H_{20}O_2Na$: 303.1356, found: 303.1352.

$[\alpha]^{25}_D$ = +91.8 (*c* 1.0, CH_2Cl_2); 92% ee from (*S*)-**L**.

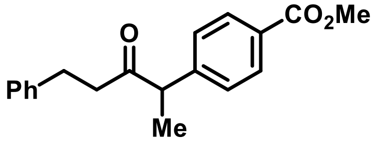

Methyl 4-(3-oxo-5-phenylpentan-2-yl)benzoate (Fig. 2, compound 26). The title compound was synthesized according to **GP-B** from 3-phenylpropanoic acid and methyl 4-ethylbenzoate. The product was purified by column chromatography on silica gel (1:10 EtOAc/Petroleum ether). Colorless oil.

(*S*)-**L**: 103 mg, 69% yield, 92% ee; (*R*)-**L**: 101 mg, 68% yield, 90% ee.

HPLC analysis: The ee was determined via HPLC on a CHIRALPAK AD-3 column (10.0% 2-PrOH in hexanes, 1.0 mL/min); retention times for compound obtained using (*S*)-**L**: 8.9 min (minor), 9.5 min (major).

^{1}H NMR (500 MHz, $CDCl_3$) δ 7.97 – 7.95 (m, 2H), 7.23 – 7.19 (m, 4H), 7.17 – 7.14 (m, 1H), 7.06 – 7.04 (m, 2H), 3.91 (s, 3H), 3.76 (q, *J* = 7.0 Hz, 1H), 2.88 – 2.75 (m, 2H), 2.68 – 2.65 (m, 2H), 1.39 (d, *J* = 7.0 Hz, 3H).

^{13}C NMR (101 MHz, $CDCl_3$) δ 208.9, 166.7, 145.4, 140.7, 130.2, 129.1, 128.4, 128.2, 127.9, 126.0, 53.1, 52.1, 42.8, 29.8, 17.2.

FT-IR (film): 3027, 2931, 1717, 1608, 1435, 1279, 1111, 1018, 858, 768, 747, 700 cm^{-1}.

HRMS (ESI-MS) *m/z* $[M+Na]^+$ calcd for $C_{19}H_{20}O_3Na$: 319.1305, found: 319.1301.

$[\alpha]^{25}_D$ = +88.9 (*c* 1.0, CH_2Cl_2); 92% ee from (*S*)-**L**.

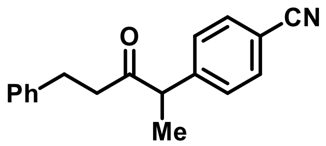

4-(3-Oxo-5-phenylpentan-2-yl)benzonitrile (Fig. 2, compound 27). The title compound was synthesized according to **GP-B** from 3-phenylpropanoic acid and 4-ethylbenzonitrile. The product was purified by column chromatography on silica gel (1:18 EtOAc/Petroleum ether). Colorless oil.

(*S*)-**L**: 81 mg, 61% yield, 88% ee; (*R*)-**L**: 81 mg, 62% yield, 88% ee.

HPLC analysis: The ee was determined via HPLC on a CHIRALPAK IC-3 column (10.0% 2-PrOH in hexanes, 1.0 mL/min); retention times for compound obtained using (*S*)-**L**: 16.6 min (major), 18.0 min (minor).

^{1}H NMR (400 MHz, $CDCl_3$) δ 7.57 – 7.55 (m, 2H), 7.24 – 7.15 (m, 5H), 7.06 – 7.04 (m, 2H), 3.77 (q, *J* = 7.0 Hz, 1H), 2.86 – 2.81 (m, 2H), 2.71 – 2.66 (m, 2H), 1.38 (d, *J* = 7.0 Hz, 3H).

^{13}C NMR (101 MHz, $CDCl_3$) δ 208.2, 145.4, 140.5, 132.5, 128.6, 128.4, 128.2, 126.1, 118.5, 111.0, 53.0, 42.9, 29.7, 17.2.

FT-IR (film): 2923, 1716, 1604, 1497, 1452, 1058, 840, 746, 694 cm^{-1}.

HRMS (ESI-MS) *m*/*z* $[M+Na]^+$ calcd for $C_{18}H_{17}NONa$: 286.1202, found: 286.1202.

$[\alpha]^{25}_{D}$ = +78.8 (*c* 1.0, CH_2Cl_2); 88% ee from (*S*)-**L**.

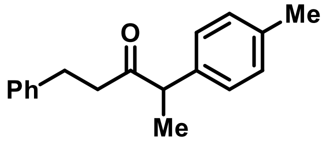

1-Phenyl-4-(*p*-tolyl)pentan-3-one (Fig. 2, compound 28). The title compound was synthesized according to **GP-B** from 3-phenylpropanoic acid and 1-ethyl-4-methylbenzene. The product was purified by column chromatography on silica gel (1:60 EtOAc/Petroleum ether). Colorless oil.

(*S*)-**L**: 84 mg, 67% yield, 93% ee; (*R*)-**L**: 78 mg, 62% yield, 95% ee.

HPLC analysis: The ee was determined via HPLC on a CHIRALCEL OJ-3 column (15.0% 2-PrOH in hexanes, 1.0 mL/min); retention times for compound obtained using (*S*)-**L**: 7.7 min (minor), 8.2 min (major).

^{1}H NMR (500 MHz, $CDCl_3$) δ 7.28 – 7.25 (m, 2H), 7.21 – 7.18 (m, 1H), 7.16 – 7.15 (m, 2H), 7.12 – 7.09 (m, 4H), 3.71 (q, *J* = 6.9 Hz, 1H), 2.93 – 2.87 (m, 1H), 2.85 – 2.77 (m, 1H), 2.76 – 2.65 (m, 2H), 2.37 (s, 3H), 1.41 (d, *J* = 7.0, Hz, 3H).

^{13}C NMR (126 MHz, $CDCl_3$) δ 209.9, 141.0, 137.4, 136.7, 129.5, 128.3, 128.2, 127.7, 125.9, 52.7, 42.4, 29.9, 21.0, 17.3.

FT-IR (film): 2974, 2928, 1712, 1513, 1453, 1372, 1060, 819, 747, 698 cm^{-1}.

HRMS (ESI-MS) *m*/*z* $[M+Na]^+$ calcd for $C_{18}H_{20}ONa$: 275.1406, found: 275.1404.

$[\alpha]^{25}_{D}$ = +125.1 (*c* 1.0, CH_2Cl_2); 93% ee from (*S*)-**L**.

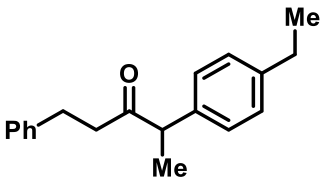

4-(4-Ethylphenyl)-1-phenylpentan-3-one (Fig. 2, compound 29). The title compound was synthesized according to **GP-B** from 3-phenylpropanoic acid and 1,4-diethylbenzene. The product was purified by column chromatography on silica gel (1:20 EtOAc/Petroleum ether). Colorless oil.

(*S*)-**L**: 80 mg, 60% yield, 95% ee; (*R*)-**L**: 77 mg, 58% yield, 94% ee.

HPLC analysis: The ee was determined via HPLC on a CHIRALCEL OJ-3 column (15.0% 2-PrOH in hexanes, 1.0 mL/min); retention times for compound obtained using (*S*)-**L**: 6.4 min (minor), 6.7 min (major).

^{1}H NMR (400 MHz, $CDCl_3$) δ 7.22 – 7.17 (m, 2H), 7.15 – 7.11 (m, 3H), 7.07 – 7.04 (m, 4H), 3.66 (q, *J* = 6.9 Hz, 1H), 2.88 – 2.70 (m, 2H), 2.69 – 2.58 (m, 4H), 1.35 (d, *J* = 7.0 Hz, 3H), 1.22 (t, *J* = 7.6 Hz, 3H).

^{13}C NMR (101 MHz, $CDCl_3$) δ 210.0, 143.0, 141.1, 137.6, 128.33, 128.29, 128.2, 127.7, 125.9, 52.7, 42.4, 29.9, 28.4, 17.3, 15.4.

FT-IR (film): 2966, 2930, 1713, 1511, 1453, 1372, 1180, 1130, 1061, 833, 748, 698 cm^{-1}.

HRMS (ESI-MS) *m/z* $[M+Na]^+$ calcd for $C_{19}H_{22}ONa$: 289.1563, found: 289.1560.

$[\alpha]^{25}_{D}$ = +117.9 (*c* 1.0, CH_2Cl_2); 95% ee from (*S*)-**L**.

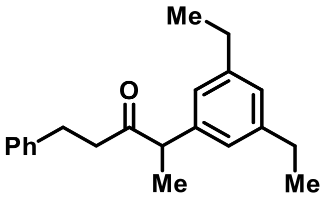

4-(3,5-Diethylphenyl)-1-phenylpentan-3-one (Fig. 2, compound 30). The title compound was synthesized according to **GP-B** from 3-phenylpropanoic acid and 1,3,5-triethylbenzene. The product was purified by column chromatography on silica gel (1:40 EtOAc/Petroleum ether). Colorless oil.

(*S*)-**L**: 95 mg, 65% yield, 93% ee; (*R*)-**L**: 100 mg, 68% yield, 93% ee.

HPLC analysis: The ee was determined via HPLC on a CHIRALPAK AD-3 column (1.0% 2-PrOH in hexanes, 1.0 mL/min); retention times for compound obtained using (*S*)-**L**: 4.4 min (major), 4.8 min (minor).

^{1}H NMR (400 MHz, $CDCl_3$) δ 7.28 – 7.23 (m, 2H), 7.20 – 7.16 (m, 1H), 7.12 – 7.09 (m, 2H), 6.97 (s, 1H), 6.85 (s, 2H), 3.70 (q, *J* = 6.9 Hz, 1H), 2.94 – 2.68 (m, 4H), 2.63 (q, *J* = 7.6 Hz, 4H), 1.41 (d, *J* = 6.9 Hz, 3H), 1.26 (t, *J* = 7.6 Hz, 6H).

^{13}C NMR (101 MHz, $CDCl_3$) δ 210.1, 144.8, 141.1, 140.4, 128.3, 128.2, 126.3, 125.9, 124.7, 53.1, 42.4, 30.0, 28.7, 17.3, 15.5.

FT-IR (film): 2965, 2931, 1713, 1600, 1454, 1372, 1076, 868, 711, 699 cm^{-1}.

HRMS (ESI-MS) *m/z* $[M+Na]^+$ calcd for $C_{21}H_{26}ONa$: 317.1876, found: 317.1872.

$[\alpha]^{25}_{D}$ = +119.6 (*c* 1.0, CH_2Cl_2); 93% ee from (*S*)-**L**.

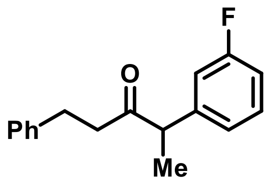

4-(3-Fluorophenyl)-1-phenylpentan-3-one (Fig. 2, compound 31). The title compound was synthesized according to **GP-B** from 3-phenylpropanoic acid and 1-ethyl-3-fluorobenzene. The product was purified by column chromatography on silica gel (1:30 EtOAc/Petroleum ether). Colorless oil.

(*S*)-**L**: 79 mg, 62% yield, 93% ee; (*R*)-**L**: 79 mg, 62% yield, 93% ee.

HPLC analysis: The ee was determined via HPLC on a CHIRALCEL OJ-3 column (15.0% 2-PrOH in hexanes, 1.0 mL/min); retention times for compound obtained using (*S*)-**L**: 7.3 min (minor), 7.4 min (major).

^{1}H NMR (400 MHz, $CDCl_3$) δ 7.33 – 7.25 (m, 3H), 7.23 – 7.19 (m, 1H), 7.14 – 7.11 (m, 2H), 7.01 – 6.96 (m, 2H), 6.94 – 6.91 (m, 1H), 3.75 (q, *J* = 7.0 Hz, 1H), 2.95 – 2.82 (m, 2H), 2.80 – 2.68 (m, 2H), 1.42 (d, *J* = 7.0 Hz, 3H).

^{19}F NMR (471 MHz, $CDCl_3$) δ -112.2 (s, 1F).

^{13}C NMR (101 MHz, $CDCl_3$) δ 209.0, 163.0 (d, J_{C-F} = 247.8 Hz), 142.7 (d, J_{C-F} = 7.4 Hz), 140.8, 130.3 (d, J_{C-F} = 8.4 Hz), 128.3, 128.2, 126.0, 123.5 (d, J_{C-F} = 3.1 Hz), 114.7 (d, J_{C-F} = 21.7 Hz), 114.0 (d, J_{C-F} = 21.1 Hz), 52.7, 42.5, 29.8, 17.2.

FT-IR (film): 3027, 2931, 1713, 1589, 1489, 1449, 1057, 907, 783, 697 cm^{-1}.

HRMS (ESI-MS) *m/z* $[M+Na]^+$ calcd for $C_{17}H_{17}FONaS$: 279.1156, found: 279.1153.

$[\alpha]^{25}_{D}$ = +104.1 (*c* 1.0, CH_2Cl_2); 93% ee from (*S*)-**L**.

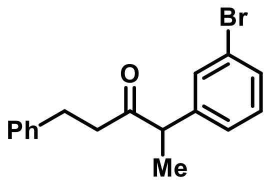

4-(3-Bromophenyl)-1-phenylpentan-3-one (Fig. 2, compound 32). The title compound was synthesized according to **GP-B** from 3-phenylpropanoic acid and 1-bromo-3-ethylbenzene. The product was purified by column chromatography on silica gel (1:20 EtOAc/Petroleum ether). Colorless oil.

(*S*)-**L**: 108 mg, 68% yield, 93% ee; (*R*)-**L**: 100 mg, 63% yield, 94% ee.

HPLC analysis: The ee was determined via HPLC on a CHIRALCEL OJ-3 column (15.0% 2-PrOH in hexanes, 1.0 mL/min); retention times for compound obtained using (*S*)-**L**: 7.7 min (minor), 8.7 min (major).

^{1}H NMR (400 MHz, $CDCl_3$) δ 7.37 – 7.34 (m, 1H), 7.31 (t, *J* = 1.7 Hz, 1H), 7.23 – 7.19 (m, 2H), 7.16 – 7.11 (m, 2H), 7.07 -7.04 (m, 3H), 3.64 (q, *J* = 6.9 Hz, 1H), 2.86 – 2.75 (m, 2H), 2.68 – 2.64 (m, 2H), 1.34 (d, *J* = 7.0 Hz, 3H).

^{13}C NMR (126 MHz, $CDCl_3$) δ 209.0, 142.6, 140.8, 130.9, 130.4, 130.3, 128.4, 128.2, 126.4, 126.1, 122.9, 52.7, 42.7, 29.8, 17.3.

FT-IR (film): 3027, 2929, 1713, 1590, 1567, 1495, 1453, 1425, 1373, 1074, 749, 696 cm^{-1}.

HRMS (ESI-MS) *m/z* $[M+Na]^+$ calcd for $C_{17}H_{17}BrONa$: 339.0355, found: 339.0354.

$[\alpha]^{25}_D$ = +80.0 (*c* 1.0, CH_2Cl_2); 93% ee from (*S*)-**L**.

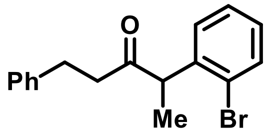

4-(2-Bromophenyl)-1-phenylpentan-3-one (Fig. 2, compound 33). The title compound was synthesized according to **GP-B** from 3-phenylpropanoic acid and 1-bromo-2-ethylbenzene. The product was purified by column chromatography on silica gel (1:20 EtOAc/Petroleum ether). Light yellow oil.

(*S*)-**L**: 58 mg, 36% yield, 91% ee; (*R*)-**L**: 59 mg, 37% yield, 90% ee.

HPLC analysis: The ee was determined via HPLC on a CHIRALCEL OJ-3 column (15.0% 2-PrOH in hexanes, 1.0 mL/min); retention times for compound obtained using (*S*)-**L**: 7.4 min (minor), 8.4 min (major).

^{1}H NMR (400 MHz, $CDCl_3$) δ 7.59 (dd, *J* = 8.0, 1.1 Hz, 1H), 7.25 – 7.21 (m, 3H), 7.19 – 7.15 (m, 1H), 7.13 – 7.08 (m, 3H), 7.05 (dd, *J* = 7.7, 1.6 Hz, 1H), 4.27 (q, *J* = 6.9 Hz, 1H), 2.93 – 2.80 (m, 2H), 2.71 – 2.67 (m, 2H), 1.36 (d, *J* = 6.9 Hz, 3H).

^{13}C NMR (101 MHz, $CDCl_3$) δ 209.1, 140.9, 140.0, 133.2, 128.60, 128.58, 128.4, 128.3, 128.0, 126.0, 124.8, 51.6, 43.0, 29.9, 16.5.

FT-IR (film): 3026, 2930, 1716, 1453, 1438, 1373, 1180, 1132, 1022, 751, 699 cm^{-1}.

HRMS (ESI-MS) *m/z* $[M+Na]^+$ calcd for $C_{17}H_{17}BrONa$: 339.0355, found: 339.0357.

$[\alpha]^{25}_D$ = +120.3 (*c* 1.0, CH_2Cl_2); 91% ee from (*S*)-**L**.

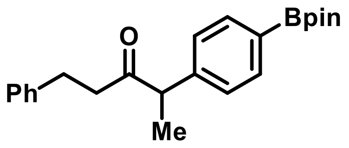

1-Phenyl-4-(4-(4,4,5,5-tetramethyl-1,3,2-dioxaborolan-2-yl)phenyl)pentan-3-one (Fig. 2, compound 34). The title compound was synthesized according to **GP-B** from 3-phenylpropanoic acid and 2-(4-ethylphenyl)-4,4,5,5-tetramethyl-1,3,2-dioxaborolane. The product was purified by column chromatography on silica gel (1:30 EtOAc/Petroleum ether). Colorless oil.

(*S*)-**L**: 130 mg, 71% yield, 94% ee; (*R*)-**L**:125 mg, 68% yield, 94% ee.

HPLC analysis: The ee was determined via HPLC on a CHIRALCEL OD-3 column (5.0% 2-PrOH in hexanes, 1.0 mL/min); retention times for compound obtained using (*S*)-**L**: 5.2 min (major), 7.7 min (minor).

^{1}H NMR (400 MHz, $CDCl_3$) δ 7.75 (d, *J* = 8.0 Hz, 2H), 7.22 – 7.13 (m, 5H), 7.06 – 7.01 (m, 2H), 3.69 (q, *J* = 6.9 Hz, 1H), 2.88 – 2.69 (m, 2H), 2.66 – 2.61 (m, 2H), 1.36 (d, *J* = 6.9 Hz, 3H), 1.33 (s, 12H).

^{13}C NMR (101 MHz, $CDCl_3$) δ 209.3, 143.5, 140.8, 135.3, 134.8, 128.3, 128.1, 127.2, 125.9, 83.7, 53.3, 42.5, 29.8, 24.7, 17.1.

FT-IR (film): 2977, 2930, 1715, 1609, 1361, 1143, 1091, 859, 748, 659 cm^{-1}.

HRMS (ESI-MS) *m*/*z* $[M+Na]^+$ calcd for $C_{23}H_{29}BO_3Na$: 387.2102, found: 387.2099.

$[\alpha]^{25}_{D}$ = +69.3 (*c* 1.0, CH_2Cl_2); 94% ee from (*S*)-**L**.

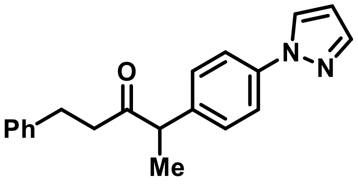

4-(4-(1*H*-Pyrazol-1-yl)phenyl)-1-phenylpentan-3-one (Fig. 2, compound 35). The title compound was synthesized according to **GP-B** from 3-phenylpropanoic acid and 1-(4-ethylphenyl)-1*H*-pyrazole. The product was purified by column chromatography on silica gel (1:5 EtOAc/Petroleum ether). Colorless oil.

(*S*)-**L**: 118 mg, 78% yield, 95% ee; (*R*)-**L**: 121 mg, 80% yield, 94% ee.

HPLC analysis: The ee was determined via HPLC on a CHIRALPAK ID column (10.0% 2-PrOH in hexanes, 1.0 mL/min); retention times for compound obtained using (*S*)-**L**: 17.8 min (major), 21.5 min (minor).

^{1}H NMR (400 MHz, $CDCl_3$) δ 7.89 (d, *J* = 2.3 Hz, 1H), 7.71 (d, *J* = 1.4 Hz, 1H), 7.63 – 7.60 (m, 2H), 7.25 – 7.19 (m, 4H), 7.16 – 7.11 (m, 1H), 7.07 – 7.05 (m, 2H), 6.45 (t, *J* = 2.0 Hz, 1H), 3.73 (q, *J* = 6.9 Hz, 1H), 2.89 – 2.63 (m, 4H), 1.39 (d, *J* = 7.0 Hz, 3H).

^{13}C NMR (101 MHz, $CDCl_3$) δ 209.4, 141.0, 140.8, 139.1, 138.5, 128.8, 128.3, 128.2, 126.6, 126.0, 119.5, 107.6, 52.5, 42.6, 29.8, 17.2.

FT-IR (film): 2971, 2929, 1712, 1524, 1394, 1333, 1046, 936, 840, 749, 699 cm^{-1}.

HRMS (ESI-MS) *m*/*z* $[M+Na]^+$ calcd for $C_{20}H_{20}N_2ONa$: 327.1468, found: 327.1465.

$[\alpha]^{25}_{D}$ = +90.7 (*c* 1.0, CH_2Cl_2); 95% ee from (*S*)-**L**.

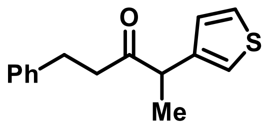

1-Phenyl-4-(thiophen-3-yl)pentan-3-one (Fig. 2, compound 36). The title compound was synthesized according to **GP-B** from 3-phenylpropanoic acid and 1-phenyl-4-(thiophen-3-yl)pentan-3-one. The product was purified by column chromatography on silica gel (1:60 EtOAc/Petroleum ether). Yellow oil.

(*S*)-**L**: 90 mg, 74% yield, 86% ee; (*R*)-**L**: 88 mg, 72% yield, 86% ee.

HPLC analysis: The ee was determined via HPLC on a CHIRALPAK AD-3 column (5.0% 2-PrOH in hexanes, 1.0 mL/min); retention times for compound obtained using (*S*)-**L**: 5.3 min (major), 5.5 min (minor).

^{1}H NMR (500 MHz, $CDCl_3$) δ 7.29 – 7.25 (m, 3H), 7.21 – 7.18 (m, 1H), 7.13 – 7.11 (m, 2H), 7.05 – 7.04 (m, 1H), 6.93 (dd, *J* = 5.0, 1.3 Hz, 1H), 3.87 (q, *J* = 7.0 Hz, 1H), 2.92 – 2.80 (m, 2H), 2.79 – 2.66 (m, 2H), 1.41 (d, *J* = 7.0 Hz, 3H).

^{13}C NMR (126 MHz, $CDCl_3$) δ 209.4, 140.9, 140.5, 128.3, 128.2, 126.9, 126.1, 125.9, 121.4, 48.3, 42.0, 29.8, 16.9.

FT-IR (film): 3026, 2931, 1713, 1453, 1371, 1113, 1058, 865, 772, 699 cm^{-1}.

HRMS (ESI-MS) m/z [M+Na]+ calcd for $C_{15}H_{16}OSNa$: 267.0814, found: 267.0814.

$[\alpha]^{25}_D$ = +81.4 (c 1.0, CH_2Cl_2); 86% ee from (*S*)-L.

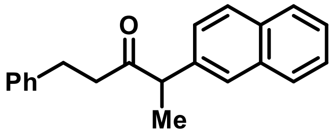

4-(Naphthalen-2-yl)-1-phenylpentan-3-one (Fig. 2, compound 37). The title compound was synthesized according to **GP-B** from 3-phenylpropanoic acid and 2-ethylnaphthalene. The product was purified by column chromatography on silica gel (1:60 EtOAc/Petroleum ether). Colorless oil.

(*S*)-**L**: 103 mg, 71% yield, 94% ee; (*R*)-**L**: 105 mg, 73% yield, 94% ee.

HPLC analysis: The ee was determined via HPLC on a CHIRALPAK AD-3 column (5.0% 2-PrOH in hexanes, 1.0 mL/min); retention times for compound obtained using (*S*)-**L**: 5.9 min (major), 6.8 min (minor).

^{1}H NMR (500 MHz, $CDCl_3$) δ 7.84 – 7.78 (m, 3H), 7.63 (s, 1H), 7.51 – 7.46 (m, 2H), 7.29 (dd, *J* = 8.4, 1.7 Hz, 1H), 7.21 – 7.18 (m, 2H), 7.15 – 7.12 (m, 1H), 7.06 – 7.04 (m, 2H), 3.89 (q, *J* = 7.0 Hz, 1H), 2.91 – 2.85 (m, 1H), 2.83 – 2.77 (m, 1H), 2.75 – 2.68 (m, 2H), 1.48 (d, *J* = 7.0 Hz, 3H).

^{13}C NMR (126 MHz, $CDCl_3$) δ 209.8, 140.9, 137.9, 133.6, 132.5, 128.7, 128.3, 128.2, 127.7, 127.6, 126.6, 126.2, 126.0, 125.9, 125.8, 53.3, 42.6, 29.9, 17.3.

FT-IR (film): 3058, 2971, 1712, 1452, 1375, 1180, 1076, 1054, 857, 820, 699 cm^{-1}.

HRMS (ESI-MS) *m/z* $[M+Na]^+$ calcd for $C_{21}H_{20}ONa$: 311.1406, found: 311.1403.

$[\alpha]^{25}_D$ = +126.4 (*c* 1.0, CH_2Cl_2); 94% ee from (*S*)-**L**.

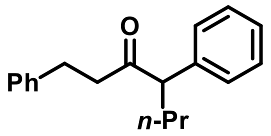

1,4-Diphenylheptan-3-one (Fig. 2, compound 38). The title compound was synthesized according to **GP-B** from 3-phenylpropanoic acid and butylbenzene. The product was purified by column chromatography on silica gel (1:40 EtOAc/Petroleum ether). Colorless oil.

(*S*)-**L**: 89 mg, 67% yield, 94% ee; (*R*)-**L**: 85 mg, 64% yield, 94% ee.

HPLC analysis: The ee was determined via HPLC on a CHIRALCEL OJ-3 column (5.0% 2-PrOH in hexanes, 1.0 mL/min); retention times for compound obtained using (*S*)-**L**: 8.0 min (minor), 8.4 min (major).

^{1}H NMR (400 MHz, $CDCl_3$) δ 7.30 – 7.25 (m, 2H), 7.24 – 7.11 (m, 6H), 7.06 – 7.04 (m, 2H), 3.57 (t, *J* = 7.4Hz, 1H), 2.88 – 2.71 (m, 2H), 2.69 – 2.59 (m, 2H), 2.03 – 1.94 (m, 1H), 1.71 – 1.61 (m, 1H), 1.22 – 1.12 (m, 2H), 0.86 (t, *J* = 7.3 Hz, 3H).

^{13}C NMR (126 MHz, $CDCl_3$) δ 209.5, 141.0, 138.9, 128.8, 128.3, 128.21, 128.19, 127.1, 125.9, 58.9, 43.3, 34.0, 29.8, 20.6, 13.9.

FT-IR (film): 2957, 2931, 1713, 1494, 1453, 1180, 1131, 1075, 750, 699 cm^{-1}.

HRMS (ESI-MS) *m*/*z* $[M+Na]^+$ calcd for $C_{19}H_{22}ONa$: 289.1568, found: 289.1571.

$[\alpha]^{25}_{D}$ = +128.2 (*c* 1.0, CH_2Cl_2); 94% ee from (*S*)-**L**.

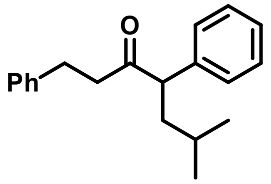

6-Methyl-1,4-diphenylheptan-3-one (Fig. 2, compound 39). The title compound was synthesized according to **GP-B** from 3-phenylpropanoic acid and isopentylbenzene. The product was purified by column chromatography on silica gel (1:40 EtOAc/Petroleum ether). Light yellow oil.

(*S*)-**L**: 75 mg, 54% yield, 93% ee; (*R*)-**L**: 70 mg, 50% yield, 93% ee.

HPLC analysis: The ee was determined via HPLC on a CHIRALCEL OD-3 column (1.0% 2-PrOH in hexanes, 1.0 mL/min); retention times for compound obtained using (*S*)-**L**: 5.6 min (major), 6.2 min (minor).

^{1}H NMR (400 MHz, $CDCl_3$) δ 7.30 – 7.26 (m, 2H), 7.24 – 7.11 (m, 6H), 7.06 – 7.03 (m, 2H), 3.67 (dd, *J* = 8.0, 7.0 Hz, 1H), 2.87 – 2.64 (m, 4H), 1.89 – 1.82 (m, 1H), 1.66 – 1.59 (m, 1H), 1.39 – 1.29 (m, 1H), 0.85 (d, *J* = 3.0 Hz, 3H), 0.83 (d, *J* = 3.0 Hz, 3H).

^{13}C NMR (101 MHz, $CDCl_3$) δ 209.5, 141.0, 139.0, 128.8, 128.33, 128.26, 128.2, 127.1, 125.9, 57.0, 43.2, 40.8, 29.9, 25.5, 23.0, 22.1.

FT-IR (film): 2955, 2930, 1713, 1494, 1466, 1453, 1367, 1069, 749, 699 cm^{-1}.

HRMS (ESI-MS) *m*/*z* $[M+Na]^+$ calcd for $C_{20}H_{24}ONa$: 303.1719, found: 303.1716.

$[\alpha]^{25}_{D}$ = +125.7 (*c* 1.0, CH_2Cl_2); 93% ee from (*S*)-**L**.

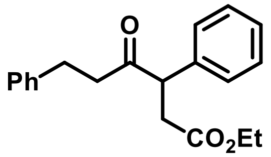

Ethyl 4-oxo-3,6-diphenylhexanoate (Fig. 2, compound 40). The title compound was synthesized according to **GP-B** from 3-phenylpropanoic acid and ethyl 3-phenylpropanoate. The product was purified by column chromatography on silica gel (1:5 EtOAc/Petroleum ether). Colorless oil.

(*S*)-**L**: 82 mg, 53% yield, 95% ee; (*R*)-**L**: 79 mg, 51% yield, 94% ee.

HPLC analysis: The ee was determined via HPLC on a CHIRALPAK IC-3 column (5.0% 2-PrOH in hexanes, 1.0 mL/min); retention times for compound obtained using (*S*)-**L**: 11.4 min (major), 12.9 min (minor).

^{1}H NMR (500 MHz, $CDCl_3$) δ 7.32 – 7.25 (m, 3H), 7.23 – 7.19 (m, 2H), 7.17 – 7.13 (m, 3H), 7.07 – 7.05 (m, 2H), 4.18 (dd, *J* = 9.9, 5.0 Hz, 1H), 4.14 – 4.08 (m, 2H), 3.23 (dd, *J* = 16.9, 9.9 Hz, 1H), 2.90 – 2.86 (m, 1H), 2.82 – 2.71 (m, 3H), 2.55 (dd, *J* = 17.0, 5.0 Hz, 1H), 1.22 (t, *J* = 7.2 Hz, 3H).

^{13}C NMR (126 MHz, $CDCl_3$) δ 207.8, 172.0, 140.8, 137.2, 129.1, 128.3, 128.19, 128.17, 127.6, 125.9, 60.6, 54.2, 43.0, 37.1, 29.6, 14.1.

FT-IR (film): 3027, 2927, 1716, 1495, 1453, 1372, 1241, 1075, 1027, 753, 699 cm^{-1}.

HRMS (ESI-MS) *m/z* $[M+Na]^+$ calcd for $C_{20}H_{22}O_3Na$: 333.1461, found: 333.1457.

$[\alpha]^{25}_{D}$ = +178.8 (*c* 1.0, CH_2Cl_2); 95% ee from (*S*)-**L**.

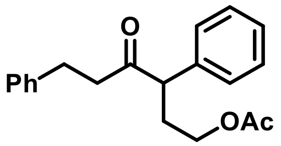

4-Oxo-3,6-diphenylhexyl acetate (Fig. 2, compound 41). The title compound was synthesized according to **GP-B** from 3-phenylpropanoic acid and 3-phenylpropyl acetate. The product was purified by column chromatography on silica gel (1:10 EtOAc/Petroleum ether). Yellow oil.

(*S*)-**L**: 101 mg, 65% yield, 95% ee; (*R*)-**L**: 102 mg, 66% yield, 95% ee.

HPLC analysis: The ee was determined via HPLC on a CHIRALPAK AD-3 column (5.0% 2-PrOH in hexanes, 1.0 mL/min); retention times for compound obtained using (*S*)-**L**: 7.8 min (major), 8.3 min (minor).

^{1}H NMR (500 MHz, $CDCl_3$) δ 7.32 – 7.28 (m, 2H), 7.27 – 7.26 (m, 1H), 7.24 – 7.20 (m, 2H), 7.17 – 7.12 (m, 3H), 7.06 – 7.04 (m, 2H), 4.03 – 3.98 (m, 1H), 3.90 – 3.85 (m, 1H), 3.71 (t, *J* = 7.2 Hz, 1H), 2.89 – 2.83 (m, 1H), 2.79 – 2.73 (m, 1H), 2.70 – 2.66 (m, 2H), 2.42 – 2.34 (m, 1H), 2.02 – 1.95 (m, 4H).

^{13}C NMR (126 MHz, $CDCl_3$) δ 208.4, 170.9, 140.8, 137.8, 129.1, 128.4, 128.25, 128.19, 127.5, 126.0, 62.3, 55.6, 43.1, 30.8, 29.8, 20.8.

FT-IR (film): 3027, 2926, 1712, 1493, 1453, 1364, 1236, 1038, 751, 699 cm^{-1}.

HRMS (ESI-MS) *m/z* $[M+Na]^+$ calcd for $C_{20}H_{22}O_3Na$: 333.1461, found: 333.1457.

$[\alpha]^{25}_{D}$ = +161.5 (*c* 1.0, CH_2Cl_2); 95% ee from (*S*)-**L**.

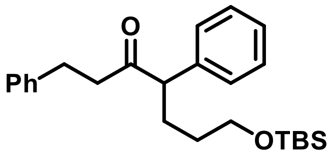

7-((*tert*-Butyldimethylsilyl)oxy)-1,4-diphenylheptan-3-one (Fig. 2, compound 42). The title compound was synthesized according to **GP-B** from 3-phenylpropanoic acid and *tert*-butyldimethyl(4-phenylbutoxy)silane. The product was purified by column chromatography on silica gel (1:40 EtOAc/Petroleum ether). Colorless oil.

(*S*)-**L**: 131 mg, 66% yield, 95% ee; (*R*)-**L**: 136 mg, 69% yield, 93% ee.

HPLC analysis: The ee was determined via HPLC on a CHIRALAPK AD-3 column (1.0% 2-PrOH in hexanes, 1.0 mL/min); retention times for compound obtained using (*S*)-**L**: 4.6 min (major), 4.9 min (minor).

^{1}H NMR (500 MHz, $CDCl_3$) δ 7.33 – 7.29 (m, 2H), 7.27 – 7.24 (m, 1H), 7.23 – 7.21 (m, 2H), 7.19 – 7.16 (m, 3H), 7.08 – 7.06 (m, 2H), 3.65 – 3.57 (m, 3H), 2.90 – 2.84 (m, 1H), 2.80 – 2.65 (m, 3H), 2.11 – 2.05 (m, 1H), 1.81 – 1.74 (m, 1H), 1.46 – 1.34 (m, 2H), 0.90 (s, 9H), 0.04 (d, *J* = 2.6 Hz, 6H).

^{13}C NMR (126 MHz, $CDCl_3$) δ 209.3, 140.9, 138.7, 128.8, 128.3, 128.2, 128.2, 127.1, 125.9, 62.9, 58.9, 43.2, 30.5, 29.8, 28.3, 25.9, 18.3, –5.3.

FT-IR (film): 2928, 2856, 1714, 1495, 1453, 1360, 1099, 835, 775, 699 cm^{-1}.

HRMS (ESI-MS) *m/z* $[M+Na]^+$ calcd for $C_{25}H_{36}O_2SiNa$: 419.2377, found: 419.2373.

$[\alpha]^{25}_{D}$ = +90.3 (*c* 1.0, CH_2Cl_2); 95% ee from (*S*)-**L**.

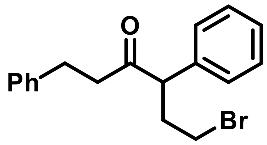

6-Bromo-1,4-diphenylhexan-3-one (Fig. 2, compound 43). The title compound was synthesized according to **GP-B** from 3-phenylpropanoic acid and (3-bromopropyl)benzene. The product was purified by column chromatography on silica gel (1:40 EtOAc/Petroleum ether). Yellow oil.

(*S*)-**L**: 83 mg, 50% yield, 80% ee; (*R*)-**L**: 82 mg, 50% yield, 83% ee.

HPLC analysis: The ee was determined via HPLC on a CHIRALCEL OJ-3 column (15.0% 2-PrOH in hexanes, 1.0 mL/min); retention times for compound obtained using (*S*)-**L**: 8.4 min (minor), 9.7 min (major).

^{1}H NMR (500 MHz, $CDCl_3$) δ 7.34 – 7.27 (m, 3H), 7.24 – 7.21 (m, 2H), 7.18 – 7.14 (m, 3H), 7.08 – 7.06 (m, 2H), 3.94 (dd, *J* = 8.1, 6.4 Hz, 1H), 3.39 – 3.35 (m, 1H), 3.18 – 3.13 (m, 1H), 2.91 – 2.85 (m, 1H), 2.81 – 2.75 (m, 1H), 2.72 – 2.69 (m, 2H), 2.56 – 2.49 (m, 1H), 2.25 – 2.18 (m, 1H).

^{13}C NMR (126 MHz, $CDCl_3$) δ 208.4, 140.7, 137.2, 129.2, 128.4, 128.4, 128.2, 127.7, 126.0, 56.6, 43.2, 34.4, 31.9, 29.8.

FT-IR (film): 2924, 2854, 1711, 1601, 1493, 1453, 1359, 1253, 1180, 1075, 1029, 753, 698 cm^{-1}.

HRMS (ESI-MS) *m/z* $[M+Na]^+$ calcd for $C_{18}H_{19}BrONa$: 353.0511, found: 353.0509.

$[\alpha]^{25}_{D}$ = +152.4 (*c* 1.0, CH_2Cl_2); 80% ee from (*S*)-**L**.

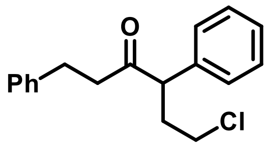

6-Chloro-1,4-diphenylhexan-3-one (Fig. 2, compound 44). The title compound was synthesized according to **GP-B** from 3-phenylpropanoic acid and (3-chloropropyl)benzene. The product was purified by column chromatography on silica gel (1:40 EtOAc/Petroleum ether). Colorless oil.

(*S*)-**L**: 85 mg, 59% yield, 94% ee; (*R*)-**L**: 88 mg, 61% yield, 95% ee.

HPLC analysis: The ee was determined via HPLC on a CHIRALCEL OJ-3 column (15.0% 2-PrOH in hexanes, 1.0 mL/min); retention times for compound obtained using (*S*)-**L**: 8.7 min (minor), 9.4 min (major).

^{1}H NMR (400 MHz, $CDCl_3$) δ 7.35 – 7.26 (m, 3H), 7.25 – 7.21 (m, 2H), 7.18 – 7.14 (m, 3H), 7.08-7.06 (m, 2H), 3.95 (dd, *J* = 8.0, 6.6 Hz, 1H), 3.53 – 3.47 (m, 1H), 3.34 – 3.28 (m, 1H), 2.93 – 2.85 (m, 1H), 2.82 -2.75 (m, 1H), 2.73 – 2.69 (m, 2H), 2.51 – 2.42 (m, 1H), 2.17 – 2.09 (m, 1H).

^{13}C NMR (126 MHz, $CDCl_3$) δ 208.5, 140.7, 137.3, 129.1, 128.4, 128.4, 128.2, 127.6, 126.0, 55.4, 43.2, 42.9, 34.3, 29.8.

FT-IR (film): 3027, 2925, 1712, 1494, 1453, 1074, 1030, 749, 699 cm^{-1}.

HRMS (ESI-MS) *m*/*z* $[M+Na]^+$ calcd for $C_{18}H_{19}ClNa$: 309.1017, found: 309.1013.

$[\alpha]^{25}_{D}$ = +197.2 (*c* 1.0, CH_2Cl_2); 94% ee from (*S*)-**L**.

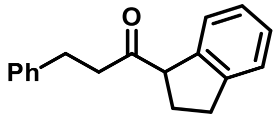

1-(2,3-Dihydro-1*H*-inden-1-yl)-3-phenylpropan-1-one (Fig. 2, compound 45). The title compound was synthesized according to **GP-B** from 3-phenylpropanoic acid and 2,3-dihydro-1*H*-indene. The product was purified by column chromatography on silica gel (1:9 EtOAc/Petroleum ether). Colorless oil.

(*S*)-**L**: 91 mg, 73% yield, 82% ee; (*R*)-**L**: 85 mg, 68% yield, 81% ee.

HPLC analysis: The ee was determined via HPLC on a CHIRALPAK AD-3 column (5.0% 2-PrOH in hexanes, 1.0 mL/min); retention times for compound obtained using (*S*)-**L**: 6.8 min (major), 7.4 min (minor).

^{1}H NMR (400 MHz, $CDCl_3$) δ 7.31 – 7.26 (m, 3H), 7.24 – 7.15 (m, 6H), 4.10 (t, *J* = 7.0 Hz, 1H), 3.12 – 3.05 (m, 1H), 2.98 – 2.90 (m, 3H), 2.89 – 2.81 (m, 2H), 2.30 (q, *J* = 6.8 Hz, 2H).

^{13}C NMR (101 MHz, $CDCl_3$) δ 209.7, 144.5, 141.1, 140.8, 128.4, 128.3, 127.5, 126.4, 126.0, 124.9, 124.7, 58.3, 42.0, 31.9, 29.8, 28.5.

FT-IR (film): 3025, 2932, 1708, 1496, 1454, 1360, 1180, 1142, 1075, 1027, 749, 699 cm^{-1}.

HRMS (ESI-MS) *m*/*z* $[M+Na]^+$ calcd for $C_{18}H_{18}ONa$: 273.1250, found: 273.1247.

$[\alpha]^{25}_{D}$ = –10.8 (*c* 1.0, CH_2Cl_2); 82% ee from (*S*)-**L**.

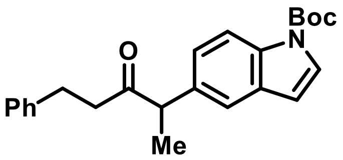

***tert*-Butyl 5-(3-oxo-5-phenylpentan-2-yl)-1*H*-indole-1-carboxylate (Fig. 2, compound 46).** The title compound was synthesized according to **GP-B** from 3-phenylpropanoic acid and *tert*-butyl 5-ethyl-1*H*-indole-1-carboxylate. The product was purified by column chromatography on silica gel (1:40 EtOAc/Petroleum ether). Colorless oil.

(*S*)-**L**: 140 mg, 74% yield, 95% ee; (*R*)-**L**: 136 mg, 72% yield, 95% ee.

HPLC analysis: The ee was determined via HPLC on a CHIRALPAK AD-3 column (5.0% 2-PrOH in hexanes, 1.0 mL/min); retention times for compound obtained using (*S*)-**L**: 6.5 min (minor), 7.3 min (major).

^{1}H NMR (400 MHz, $CDCl_3$) δ 8.08 (d, *J* = 8.6 Hz, 1H), 7.60 (d, *J* = 3.8 Hz, 1H), 7.34 (d, *J* = 2.0 Hz, 1H), 7.22 – 7.18 (m, 2H), 7.16 – 7.11 (m, 2H), 7.07 – 7.04 (m, 2H), 6.52 (d, *J* = 3.7 Hz, 1H), 3.80 (q, *J* = 7.0 Hz, 1H), 2.90 – 2.61 (m, 4H), 1.68 (s, 9H), 1.43 (d, *J* = 6.8 Hz, 3H).

^{13}C NMR (101 MHz, $CDCl_3$) δ 210.1, 149.6, 141.0, 134.9, 134.3, 131.1, 128.3, 128.2, 126.4, 125.9, 124.1, 120.0, 115.5, 107.1, 83.7, 53.0, 42.4, 30.0, 28.1, 17.6.

FT-IR (film): 2976, 1731, 1469, 1371, 1348, 1256, 1161, 1082, 1023, 728, 698 cm^{-1}.

HRMS (ESI-MS) *m/z* $[M+Na]^+$ calcd for $C_{24}H_{27}NO_3Na$: 400.1883, found: 400.1879.

$[\alpha]^{25}_{D}$ = –77.5 (*c* 1.0, CH_2Cl_2); 95% ee from (*R*)-**L**.

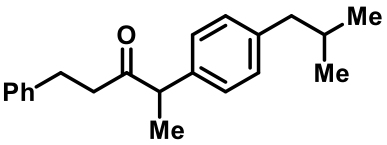

4-(4-Isobutylphenyl)-1-phenylpentan-3-one (Fig. 3, compound 47). The title compound was synthesized according to **GP-B** from 3-phenylpropanoic acid and 4-(4-isobutylphenyl)-1-phenylpentan-3-one. The product was purified by column chromatography on silica gel (1:100 EtOAc/Petroleum ether). Colorless oil.

(*S*)-**L**: 91 mg, 62% yield, 94% ee; (*R*)-**L**: 88 mg, 60% yield, 95% ee.

HPLC analysis: The ee was determined via HPLC on a CHIRALCEL OJ-3 column (5.0% 2-PrOH in hexanes, 1.0 mL/min); retention times for compound obtained using (*S*)-**L**: 7.6 min (major), 8.7 min (minor).

^{1}H NMR (500 MHz, $CDCl_3$) δ 7.21 – 7.17 (m, 2H), 7.14 – 7.10 (m, 1H), 7.07 – 7.02 (m, 6H), 3.66 (q, *J* = 7.0 Hz, 1H), 2.86 – 2.58 (m, 4H), 2.43 (d, *J* = 7.1 Hz, 2H), 1.87 – 1.79 (m, 1H), 1.35 (d, *J* = 7.0 Hz, 3H), 0.89 (d, *J* = 6.6 Hz, 6H).

^{13}C NMR (126 MHz, $CDCl_3$) δ 209.9, 141.0, 140.5, 137.6, 129.5, 128.3, 128.2, 128.2, 127.5, 125.9, 52.7, 44.9, 42.4, 30.1, 29.9, 22.3, 17.2.

FT-IR (film): 2955, 2868, 1713, 1510, 1453, 1366, 1180, 1128, 1075, 1020, 846, 748, 699 cm^{-1}.

HRMS (ESI-MS) *m/z* $[M+Na]^+$ calcd for $C_{21}H_{26}ONa$: 341.1512, found: 341.1508.

$[\alpha]^{25}_{D}$ = +99.5 (*c* 1.0, CH_2Cl_2); 94% ee from (*S*)-**L**.

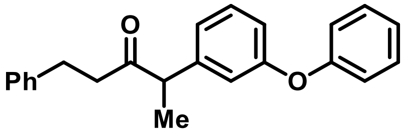

4-(3-Phenoxyphenyl)-1-phenylpentan-3-one (Fig. 3, compound 48). The title compound was synthesized according to **GP-B** from 3-phenylpropanoic acid and 1-ethyl-3-phenoxybenzene. The product was purified by column chromatography on silica gel (1:30 EtOAc/Petroleum ether). Colorless oil.

(*S*)-**L**: 121 mg, 73% yield, 93% ee; (*R*)-**L**: 127 mg, 77% yield, 94% ee.

HPLC analysis: The ee was determined via HPLC on a CHIRALPAK AD-3 column (5.0% 2-PrOH in hexanes, 1.0 mL/min); retention times for compound obtained using (*S*)-**L**: 6.2 min (major), 6.7 min (minor).

^{1}H NMR (400 MHz, $CDCl_3$) δ 7.41 – 7.35 (m, 2H), 7.33 – 7.27 (m, 3H), 7.24 – 7.19 (m, 1H), 7.18 – 7.13 (m, 3H), 7.07 – 7.04 (m, 2H), 6.97 – 6.92 (m, 3H), 3.72 (q, *J* = 7.0 Hz, 1H), 2.96 – 2.82 (m, 2H), 2.80 – 2.67 (m, 2H), 1.43 (d, *J* = 7.0 Hz, 3H).

^{13}C NMR (101 MHz, $CDCl_3$) δ 209.2, 157.6, 156.8, 142.3, 140.9, 130.0, 129.7, 1283, 128.1, 125.9, 123.3, 122.5, 118.8, 118.3, 117.2, 52.8, 42.4, 29.8, 17.1.

FT-IR (film): 3026, 2927, 1712, 1581, 1484, 1243, 1142, 1075, 924, 750, 692 cm^{-1}.

HRMS (ESI-MS) *m*/*z* $[M+Na]^+$ calcd for $C_{23}H_{22}O_2Na$: 353.1512, found: 353.1508.

$[\alpha]^{25}_{D}$ = +89.3 (*c* 1.0, CH_2Cl_2); 93% ee from (*S*)-**L**.

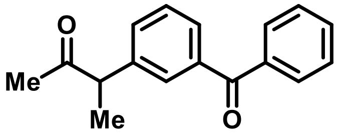

3-(3-Benzoylphenyl)butan-2-one (Fig. 3, compound 49). The title compound was synthesized according to **GP-B** from acetic acid and (3-ethylphenyl)(phenyl)methanone. The product was purified by column chromatography on silica gel (1:10 EtOAc/Petroleum ether). Colorless oil.

(*S*)-**L**: 108 mg, 85% yield, 94% ee; (*R*)-**L**: 105 mg, 83% yield, 93% ee.

HPLC analysis: The ee was determined via HPLC on a CHIRALCEL OJ-3 column (15.0% 2-PrOH in hexanes, 1.0 mL/min); retention times for compound obtained using (*S*)-**L**: 11.9 min (minor), 12.4 min (major).

^{1}H NMR (400 MHz, $CDCl_3$) δ 7.56 – 7.52 (m, 2H), 7.47 – 7.45 (m, 1H), 7.44 – 7.41 (m, 1H), 7.36 – 7.32 (m, 1H), 7.25 – 7.18 (m, 4H), 3.61 (q, *J* = 7.0 Hz, 1H), 1.85 (s, 3H), 1.19 (d, *J* = 7.0 Hz, 3H).

^{13}C NMR (101 MHz, $CDCl_3$) δ 207.9, 196.1, 140.7, 138.0, 137.2, 132.4, 131.5, 129.8, 129.2, 128.8, 128.7, 128.1, 53.2, 28.3, 17.1.

FT-IR (film): 2927, 1707, 1654, 1596, 1446, 1383, 1276, 1163, 786, 701, 634 cm^{-1}.

HRMS (ESI-MS) *m*/*z* $[M+Na]^+$ calcd for $C_{17}H_{16}O_2Na$: 275.1043, found: 275.1039.

$[\alpha]^{25}_{D}$ = +113.9 (*c* 1.0, CH_2Cl_2); 94% ee from (*S*)-**L**.

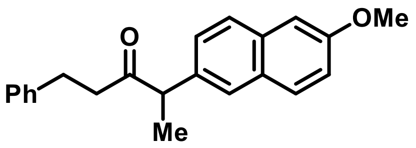

4-(6-Methoxynaphthalen-2-yl)-1-phenylpentan-3-one (Fig. 3, compound 50). The title compound was synthesized according to **GP-B** from 3-phenylpropanoic acid and 2-ethyl-6-methoxynaphthalene. The product was purified by column chromatography on silica gel (1:100 EtOAc/Petroleum ether). Colorless oil.

(*S*)-**L**: 105 mg, 66% yield, 93% ee; (*R*)-**L**: 109 mg, 68% yield, 92% ee.

HPLC analysis: The ee was determined via HPLC on a CHIRALPAK AD-3 column (5.0% 2-PrOH in hexanes, 1.0 mL/min); retention times for compound obtained using (*S*)-**L**: 10.6 min (major), 11.6 min (minor).

^{1}H NMR (500 MHz, $CDCl_3$) δ 7.64 (t, *J* = 9.3 Hz, 2H), 7.50 (s, 1H), 7.21 (dd, *J* = 8.4, 1.9 Hz, 1H), 7.17 – 7.12 (m, 3H), 7.10 – 7.07 (m, 2H), 7.01 (d, *J* = 6.9 Hz, 2H), 3.85 (s, 3H), 3.79 (q, *J* = 7.0 Hz, 1H), 2.86 – 2.79 (m, 1H), 2.77 – 2.71 (m, 1H), 2.70 – 2.61 (m, 2H), 1.43 (d, *J* = 7.0 Hz, 3H).

^{13}C NMR (126 MHz, $CDCl_3$) δ 209.8, 157.6, 140.9, 135.5, 133.5, 129.1, 129.0, 128.2, 128.1, 127.4, 126.4, 126.2, 125.8, 119.0, 105.5, 55.1, 52.9, 42.4, 29.8, 17.2.

FT-IR (film): 2927, 1707, 1604, 1389, 1261, 1030, 849, 739, 696 cm^{-1}.

HRMS (ESI-MS) *m*/*z* $[M+Na]^+$ calcd for $C_{22}H_{22}O_2Na$: 341.1512, found: 341.1508.

$[\alpha]^{25}_{D}$ = +115.9 (*c* 1.0, CH_2Cl_2); 93% ee from (*S*)-**L**.

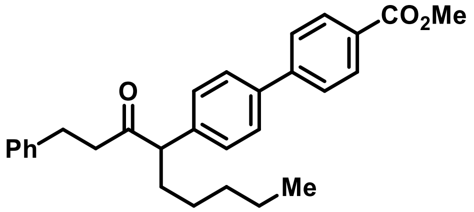

Methyl 4'-(3-oxo-1-phenylnonan-4-yl)-[1,1'-biphenyl]-4-carboxylate (Fig. 3, compound 51). The title compound was synthesized according to **GP-B** from 3-phenylpropanoic acid and methyl 4'-hexyl-[1,1'-biphenyl]-4-carboxylate. The product was purified by column chromatography on silica gel (1:10 EtOAc/Petroleum ether). White solid (mp = 67-69 °C).

(*S*)-**L**: 155 mg, 72% yield, 92% ee; (*R*)-**L**: 159 mg, 74% yield, 92% ee.

HPLC analysis: The ee was determined via HPLC on a CHIRALCEL OD-3 column (5.0% 2-PrOH in hexanes, 1.0 mL/min); retention times for compound obtained using (*S*)-**L**: 8.7 min (major), 9.9 min (minor).

^{1}H NMR (400 MHz, $CDCl_3$) δ 8.09 (d, *J* = 8.4 Hz, 2H), 7.63 (d, *J* = 8.4 Hz, 2H), 7.53 (d, *J* = 8.2 Hz, 2H)., 7.24 (d, *J* = 8.3 Hz, 2H), 7.21 – 7.17 (m, 2H), 7.15 – 7.10 (m, 1H), 7.07 – 7.05 (m, 2H), 3.91 (s, 3H), 3.62 (t, *J* = 7.4 Hz, 1H), 2.90 – 2.77 (m, 2H), 2.76 – 2.66 (m, 2H), 2.09 – 2.00 (m, 1H), 1.75 – 1.66 (m, 1H), 1.27 – 1.12 (m, 6H), 0.92 – 0.80 (t, *J* = 6.7 Hz, 3H).

^{13}C NMR (126 MHz, $CDCl_3$) δ 209.2, 166.8, 144.9, 140.8, 138.9, 138.6, 130.0, 128.8, 128.7, 128.3, 128.2, 127.5, 126.8, 125.9, 58.8, 52.0, 43.3, 31.9, 31.6, 29.7, 27.1, 22.3, 13.9.

FT-IR (film): 2927, 2856, 1718, 1607, 1434, 1278, 1110, 1005, 828, 772, 699 cm^{-1}.

HRMS (ESI-MS) *m/z* $[M+Na]^+$ calcd for $C_{29}H_{32}O_3Na$: 451.2244, found: 451.2241.

$[\alpha]^{25}_{D}$ = +70.5 (*c* 1.0, CH_2Cl_2); 92% ee from (*S*)-**L**.

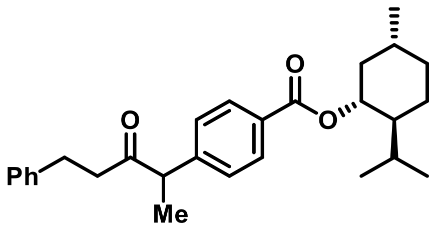

(1*R*,2*S*,5*R*)-2-Isopropyl-5-methylcyclohexyl 4-(3-oxo-5-phenylpentan-2-yl)benzoate (Fig. 3, compound 52 & 53). The title compound was synthesized according to GP-B from lithocholic acid and (1*R*,2*S*,5*R*)-2-isopropyl-5-methylcyclohexyl 4-(3-oxo-5-phenylpentan-2-yl)benzoate. The product was purified by column chromatography on silica gel (1:60 EtOAc/Petroleum ether). Colorless oil.

(*S*)-**L**: 147 mg, 70% yield, 4:96 dr;

(*R*)**-L:** 143 mg, 70% yield, 96:4 dr.

HPLC analysis: The dr was determined via HPLC on a CHIRALPAK AD-3 column (5.0% 2-PrOH in hexanes, 1.0 mL/min); retention times for compound obtained using (*S*)-L: 12.9 min (minor), 15.0 min (major).

NMR data for the product from (*S*)-**L**:

^{1}H NMR (400 MHz, $CDCl_3$) δ 7.96 (d, *J* = 7.1 Hz, 2H), 7.21 (t, *J* = 6.9 Hz, 4H), 7.17 – 7.13 (m, 1H), 7.06 (d, *J* = 7.4 Hz, 2H), 4.93 (td, *J* = 10.8, 3.8 Hz, 1H), 3.76 (q, *J* = 6.9 Hz, 1H), 2.89 – 2.75 (m, 2H), 2.67 (t, *J* = 7.5 Hz, 2H), 2.12 (d, *J* = 12.0 Hz, 1H), 1.99 – 1.91 (m, 1H), 1.73 (d, *J* = 11.0 Hz, 2H), 1.59 – 1.52 (m, 3H), 1.39 (d, *J* = 6.8 Hz, 3H), 1.18 – 1.05 (m, 2H), 0.93 (t, *J* = 4.8 Hz, 6H), 0.80 (d, *J* = 6.8 Hz, 3H).

^{13}C NMR (101 MHz, $CDCl_3$) δ 208.8, 165.6, 145.2, 140.7, 130.1, 129.7, 128.3, 128.2, 127.8, 126.0, 74.7, 53.1, 47.2, 42.7, 40.9, 34.2, 31.4, 29.8, 26.4, 23.5, 22.0, 20.7, 17.2, 16.4.

NMR data for the product from (*R*)-**L**:

^{1}H NMR (400 MHz, $CDCl_3$) δ 7.96 (d, *J* = 7.2 Hz, 2H), 7.23 – 7.19 (m, 4H), 7.17 – 7.13 (m, 1H), 7.06 (d, *J* = 7.7 Hz, 2H), 4.93 (td, *J* = 10.7, 4.1 Hz, 1H), 3.76 (q, *J* = 6.9 Hz, 1H), 2.89 – 2.76 (m, 2H), 2.67 (t, *J* = 7.3 Hz, 2H), 2.11 (d, *J* = 12.2 Hz, 1H), 2.00 – 1.92 (m, 1H), 1.73 (d, *J* = 11.1 Hz, 2H), 1.60 – 1.52 (m, 3H), 1.38 (d, *J* = 6.8 Hz, 3H), 1.19 – 1.05 (m, 2H), 0.93 (d, *J* = 6.8 Hz, 6H), 0.80 (d, *J* = 6.8 Hz, 3H).

^{13}C NMR (101 MHz, $CDCl_3$) δ 208.7, 165.6, 145.1, 140.7, 130.1, 129.6, 128.3, 128.1, 127.7, 125.9, 74.7, 53.0, 47.1, 42.6, 40.8, 34.2, 31.3, 29.7, 26.4, 23.5, 21.9, 20.7, 17.2, 16.4.

FT-IR (film): 3026, 2927, 1712, 1581, 1484, 1442, 1243, 1142, 1075, 924, 750, 692 cm^{-1}.

HRMS (ESI-MS) *m/z* $[M+Na]^+$ calcd for $C_{28}H_{36}O_3Na$: 443.2557, found: 443.2553.

$[\alpha]^{25}_{D}$ = +11.4 (*c* 1.0, CH_2Cl_2); 4:96 dr from (*S*)-**L**.

$[\alpha]^{25}_{D}$ = –119.0 (*c* 1.0, CH_2Cl_2); 96:4 dr from (*R*)**-L**.

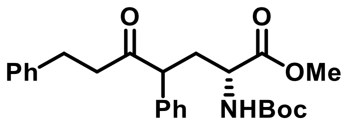

Methyl (2*R*)-2-((*tert*-butoxycarbonyl)amino)-5-oxo-4,7-diphenylheptanoate (Fig. 3, compound 54 & 55). The title compound was synthesized according to GP-B from lithocholic acid and methyl methyl (*R*)-2-((*tert*-butoxycarbonyl)amino)-4-phenylbutanoate. The product was purified by column chromatography on silica gel (1:5 EtOAc/Petroleum ether). Colorless oil.

(*S*)-**L**: 98 mg, 46% yield, 3:97 dr;

(*R*)-**L**: 93 mg, 44% yield, 99:1 dr.

HPLC analysis: The dr was determined via HPLC on a CHIRALPAK AD-3 column (10.0% 2-PrOH in hexanes, 1.0 mL/min); retention times for compound obtained using (*S*)-L: 16.6 min (minor), 18.6 min (major).

NMR data for the product from (*S*)-**L**:

^{1}H NMR (400 MHz, $CDCl_3$) δ 7.32 – 7.24 (m, 3H), 7.23 – 7.18 (m, 2H), 7.17 – 7.12 (m, 3H), 7.04 – 7.02 (m, 2H), 4.98 (d, *J* = 8.9 Hz, 1H), 4.14 (s, 1H), 3.75 (t, *J* = 7.0 Hz, 1H), 3.66 (s, 3H), 2.88 – 2.73 (m, 2H), 2.72 – 2.65 (m, 2H), 2.43 (s, 1H), 2.21 – 2.14 (m, 1H), 1.44 (s, 9H).

^{13}C NMR (101 MHz, $CDCl_3$) δ 208.3, 172.9, 155.2, 140.6, 137.5, 129.0, 128.2, 128.1, 127.5, 125.9, 79.8, 55.4, 52.1, 43.0, 34.4, 29.7, 28.2.

NMR data for the product from (*R*)-**L**:

^{1}H NMR (400 MHz, $CDCl_3$) δ 7.30 – 7.27 (m, 1H), 7.26 – 7.23 (m, 2H), 7.21 – 7.17 (m, 2H), 7.15 – 7.11 (m, 3H), 7.05 – 7.03 (m, 2H), 5.09 (s, 1H), 4.30 (s, 1H), 3.78 (dd, *J* = 8.9, 5.3 Hz, 1H), 3.65 (s, 3H), 2.91 – 2.83 (m, 1H), 2.78 – 2.61 (m, 4H), 1.79 (s, 1H), 1.45 (s, 9H).

^{13}C NMR (101 MHz, $CDCl_3$) δ 207.8, 172.7, 155.4, 140.9, 137.9, 129.0, 128.2, 128.2, 127.5, 125.8, 79.9, 54.8, 52.2, 51.9, 43.0, 35.6, 29.6, 28.2.

FT-IR (film): 2928, 1713, 1496, 1365, 1250, 1163, 1049, 748, 700 cm^{-1}.

HRMS (ESI-MS) *m*/*z* $[M+Na]^+$ calcd for $C_{25}H_{31}NO_5Na$: 448.2094, found: 448.2090.

$[\alpha]^{25}_D$ = +98.0 (*c* 1.0, CH_2Cl_2); 3:97 dr from (*S*)-**L**.

$[\alpha]^{25}_D$ = –148.7 (*c* 1.0, CH_2Cl_2); 99:1 dr from (*R*)-**L**.

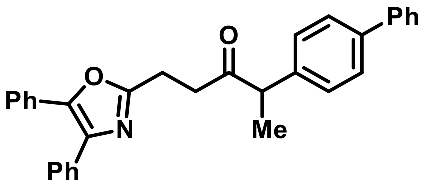

4-([1,1'-Biphenyl]-4-yl)-1-(4,5-diphenyloxazol-2-yl)pentan-3-one (Fig. 3, compound 56). The title compound was synthesized according to **GP-B** from 3-(4,5-diphenyloxazol-2-yl)propanoic acid and 4-ethyl-1,1'-biphenyl. The product was purified by column chromatography on silica gel (1:5 EtOAc/Petroleum ether). Light green oil.

(*S*)-**L**: 180 mg, 78% yield, 87% ee; (*R*)-**L**: 176 mg, 77% yield, 88% ee.

HPLC analysis: The ee was determined via HPLC on a CHIRALPAK AD-3 column (10.0% 2-PrOH in hexanes, 1.0 mL/min); retention times for compound obtained using (*S*)-**L**: 7.9 min (major), 8.9 min (minor).

^{1}H NMR (400 MHz, $CDCl_3$) δ δ 7.60 – 7.58 (m, 2H), 7.57 – 7.53 (m, 6H), 7.45 – 7.42 (m, 2H), 7.36 – 7.30 (m, 9H), 3.92 (q, *J* = 5.6 Hz, 1H), 3.18 – 3.12 (m, 1H), 3.09 – 3.06 (m, 1H), 3.05 – 3.00 (m, 2H), 1.49 (dd,*5.6* Hz, 3H).

^{13}C NMR (126 MHz, $CDCl_3$) δ 208.5, 162.3, 145.2, 140.6, 140.2, 139.3, 135.0, 132.5, 129.0, 128.7, 128.6, 128.5, 128.33, 128.28, 128.0, 127.8, 127.7, 127.3, 127.0, 126.4, 52.6, 37.6, 22.4, 17.5.

FT-IR (film): 2922, 1712, 1570, 1484, 1384, 1054, 960, 838, 761, 693 cm^{-1}.

HRMS (ESI-MS) *m/z* $[M+Na]^+$ calcd for $C_{32}H_{27}NO_2Na$: 480.1934, found: 480.1930.

$[\alpha]^{25}_D$ = +67.1 (*c* 1.0, CH_2Cl_2); 87% ee from (*S*)-**L**.

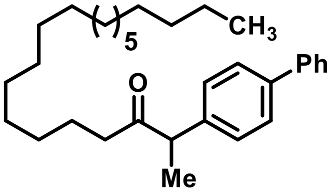

2-([1,1'-Biphenyl]-4-yl)icosan-3-one (Fig. 3, compound 57). The title compound was synthesized according to **GP-B** from stearic acid and 4-ethyl-1,1'-biphenyl. The product was purified by column chromatography on silica gel (1:600 EtOAc/Petroleum ether). White solid (mp = 63-65 °C).

(*S*)-**L**: 208mg, 93% yield, 90% ee; (*R*)-**L**: 201 mg, 90% yield, 90% ee.

HPLC analysis: The ee was determined via HPLC on a CHIRALCEL OJ-3 column (5.0% 2-PrOH in hexanes, 1.0 mL/min); retention times for compound obtained using (*S*)-**L**: 4.4 min (minor),5.1 min (major).

^{1}H NMR (400 MHz, $CDCl_3$) δ 7.60 – 7.54 (m, 4H), 7.45 – 7.42 (m, 2H), 7.36 – 7.32 (m, 1H), 7.29 (d, *J* = 8.2 Hz, 2H), 3.80 (q, *J* = 7.0 Hz, 1H), 2.41 – 2.37 (m, 2H), 1.53 – 1.46 (m, 2H), 1.43 (d, *J* = 7.0 Hz, 3H), 1.32 – 1.16 (m, 28H), 0.88 (t, *J* = 6.9 Hz, 3H).

^{13}C NMR (126 MHz, $CDCl_3$) δ 211.1, 140.6, 140.0, 139.8, 128.8, 128.3, 127.5, 127.3, 127.0, 52.6, 41.1, 31.9, 29.7, 29.6, 29.6, 29.4, 29. 4, 29.3, 29.1, 23.9, 22.7, 17.5, 14.1.

FT-IR (film): 2921, 2850, 1710, 1471, 1406, 848, 762, 724 cm^{-1}.

HRMS (ESI-MS) *m/z* $[M+Na]^+$ calcd for $C_{32}H_{48}ONa$: 471.3597, found: 471.3593.

$[\alpha]^{25}_D$ = +92.5 (*c* 1.0, CH_2Cl_2); 90% ee from (*S*)-**L**.

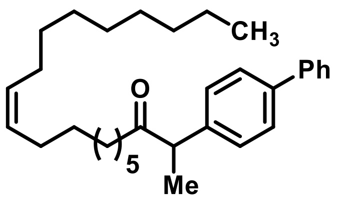

(*Z*)-2-([1,1'-Biphenyl]-4-yl)icos-11-en-3-one (Fig. 3, compound 58). The title compound was synthesized according to **GP-B** from oleic acid and 4-ethyl-1,1'-biphenyl. The product was purified by column chromatography on silica gel (1:400 EtOAc/Petroleum ether). White solid (mp = 39-41 °C).

(*S*)-**L**: 93 mg, 41% yield, 94% ee; (*R*)-**L**: 87 mg, 39% yield, 94% ee.

HPLC analysis: The ee was determined via HPLC on a CHIRALCEL OJ-3 column (10.0% 2-PrOH in hexanes, 1.0 mL/min); retention times for compound obtained using (*S*)-**L**: 3.9 min (minor), 4.3 min (major).

^{1}H NMR (500 MHz, $CDCl_3$) δ 7.59 – 7.56 (m, 3H), 7.55 (t, *J* = 1.9 Hz, 1H), 7.45 – 7.42 (m, 2H), 7.36 – 7.33 (m, 1H), 7.30 – 7.28 (m, 2H), 5.36 – 5.34 (m, 2H), 3.80 (q, *J* = 7.0 Hz, 1H), 2.41 – 2.37 (m, 2H), 2.00 – 1.90 (m, 4H), 1.56 – 1.47 (m, 2H), 1.43 (d, *J* = 7.0 Hz, 3H), 1.33 – 1.26 (m, 14H), 1.23 – 1.18 (m, 6H), 0.88 (t, *J* = 6.9 Hz, 3H).

^{13}C NMR (126 MHz, $CDCl_3$) δ 211.0, 140.6, 140.00, 139.76, 130.4, 130.2, 128.8, 128.3, 127.5, 127.3, 127.0, 52.6, 41.1, 32.6, 32.5, 31.9, 29.6, 29.5, 29.5, 29.3, 29,182, 29.176, 29.0, 28.9, 23.9, 22. 7, 17.5, 14.1.

FT-IR (film): 2924, 2852, 1715, 1486, 1456, 1180, 1131, 1075, 1007, 967, 840, 763, 731, 696 cm^{-1}.

HRMS (ESI-MS) *m/z* $[M+Na]^+$ calcd for $C_{32}H_{46}ONa$: 469.3441, found: 469.3443.

$[\alpha]^{25}_{D}$ = +101.2 (*c* 1.0, CH_2Cl_2); 94% ee from (*S*)-**L**.

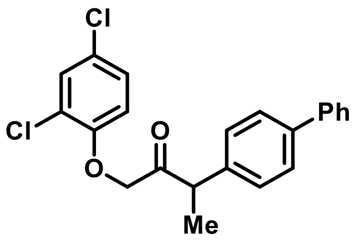

3-([1,1'-Biphenyl]-4-yl)-1-(2,4-dichlorophenoxy)butan-2-one (Fig. 3, compound 59). The title compound was synthesized according to **GP-B** from 2-(2,4-dichlorophenoxy)acetic acid and 4-ethylbiphenyl. The product was purified by column chromatography on silica gel (1:15 EtOAc/Petroleum ether). White solid (mp = 105-107 °C).

(*S*)-**L**: 73 mg, 38% yield, 87% ee; (*R*)-**L**: **77** mg, 40% yield, 86% ee.

HPLC analysis: The ee was determined via HPLC on a CHIRALPAK AD-3 column (5.0% 2-PrOH in hexanes, 1.0 mL/min); retention times for compound obtained using (*S*)-**L**: 8.0 min (major), 8.7 min (minor).

^{1}H NMR (500 MHz, $CDCl_3$) δ 7.57 – 7.54 (m, 4H), 7.45 – 7.42 (m, 2H), 7.38 (d, *J* = 2.5 Hz, 1H), 7.37 – 7.34 (m, 3H), 7.05 (dd, *J* = 8.8, 2.5 Hz, 1H), 6.49 (d, *J* = 8.8 Hz, 1H), 4.59 (s, 2H), 4.25 (q, *J* = 7.0 Hz, 1H), 1.49 (d, *J* = 7.0 Hz, 3H).

^{13}C NMR (126 MHz, $CDCl_3$) δ 205.8, 152.1, 140.4, 140.4, 138.1, 130.2, 128.8, 128.5, 127.7, 127.5, 127.4, 127.0, 126.7, 123.8, 113.9, 72.1, 48.4, 17.1.

FT-IR (film): 2971, 2900, 1718, 1476, 1384, 1242, 1196, 1180, 1075, 1052, 840, 731, 695 cm^{-1}.

HRMS (ESI-MS) *m/z* $[M+Na]^+$ calcd for $C_{22}H_{18}Cl_2O_2Na$: 407.0576, found: 407.0578.

$[\alpha]^{25}_D$ = +105.2 (*c* 1.0, CH_2Cl_2); 87% ee from (*S*)-**L**.

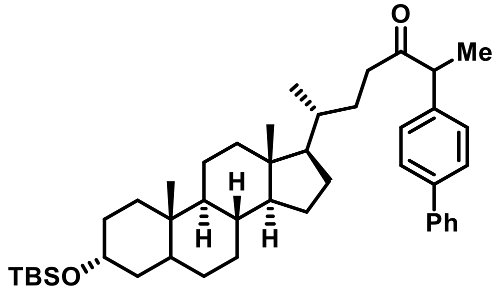

(6*R*)-2-([1,1'-Biphenyl]-4-yl)-6-((3*R*,8*R*,9*S*,10*S*,13*R*,14*S*,17*R*)-3-((*tert*-butyldimethylsilyl)oxy)-10,13-dimethylhexadecahydro-1*H*-cyclopenta[*a*]phenanthren-17-yl)heptan-3-one (Fig. 3, compound 60 & 61). The title compound was synthesized according to **GP-B** from lithocholic acid and 4-ethyl-1,1'-biphenyl. The product was purified by column chromatography on silica gel (1:400 EtOAc/Petroleum ether). White solid (mp = 101-102 °C).

(*S*)-**L**: 295 mg, 84% yield, 4:96 dr;

(*R*)-**L**: 300 mg, 85% yield, 96:4 dr.

HPLC analysis: The dr was determined via HPLC on a CHIRALCEL OD-3 column (5.0% 2-PrOH in hexanes, 1.0 mL/min); retention times for compound obtained using (*S*)-**L**: 4.0 min (minor), 4.2 min (major).

NMR data for the product from (*S*)-**L**:

^1H NMR (500 MHz, $CDCl_3$) δ 7.61 – 7.57 (m, 4H), 7.46 – 7.43 (m, 2H), 7.36 – 7.30 (m, 3H), 3.82 (q, *J* = 6.9 Hz, 1H), 3.63 – 3.57 (m, 1H), 2.48 – 2.42 (m, 1H), 2.35 – 2.28 (m, 1H), 1.92 – 1.89 (m, 1H), 1.86 – 1.79 (m, 2H), 1.77 – 1.70 (m, 3H), 1.58 – 1.50 (m, 2H), 1.45 (d, *J* = 7.0 Hz, 3H), 1.42 – 1.29 (m, 8H), 1.26 – 1.07 (m, 6H), 1.05 – 1.00 (m, 3H), 0.98 – 0.96 (m, 1H), 0.92 (s, 9H), 0.91 (s, 3H), 0.82 (d, *J* = 6.6 Hz, 3H), 0.61 (s, 3H), 0.09 (s, 6H).

^{13}C NMR (126 MHz, $CDCl_3$) δ 211.4, 140.6, 140.0, 139.7, 128.7, 128.3, 127.5, 127.2, 127.0, 72.8, 56.3, 55.9, 52.7, 42.6, 42.2, 40.2, 40.1, 37.9, 36. 9, 35.8, 35.5, 35.2, 34.5, 31.0, 30.1, 28.0, 27.3, 26.3, 25.9, 24.1, 23.3, 20.8, 18.4, 18.3, 17.5, 12.0, –4.6.

NMR data for the product from (*R*)-**L**:

^1H NMR (500 MHz, $CDCl_3$) δ 7.59 (t, *J* = 8.1 Hz, 4H), 7.44 (t, *J* = 6.2 Hz, 2H), 7.36 – 7.30 (m, 3H), 3.83 (q, *J* = 6.3 Hz, 1H), 3.63 – 3.57 (m, 1H), 2.41 – 2.36 (m, 2H), 1.90 – 1.67 (m, 7H), 1.58 – 1.34 (m, 12H), 1.29 – 1.18 (m, 5H), 1.15 – 1.07 (m, 2H), 1.04 – 1.01 (m, 3H), 0.92 (s, 9H), 0.91 (s, 3H), 0.76 (s, 3H), 0.59 (s, 3H), 0.09 (s, 6H).

^{13}C NMR (126 MHz, $CDCl_3$) δ 211.1, 140.6, 139.9, 139.7, 128.7, 128.2, 127.5, 127.2, 126.9, 72.7, 56.3, 56.0, 52.5, 42.6, 42.2, 40.1, 40.0, 38.0, 36.8, 35.8, 35.5, 35.1, 34.5, 31.0, 30.0, 28.1, 27.2, 26.3, 25.9, 24.1, 23.3, 20.7, 18.23, 18.17, 17.4, 11.9, –4.6.

FT-IR (film):2927, 2857, 1714, 1486, 1449, 1372, 1250, 1094, 1077, 835, 773, 697 cm^{-1}.

HRMS (ESI-MS) m/z $[M+Na]^+$ calcd for $C_{44}H_{66}O_2SiNa$: 677.4724, found: 677.4719.

$[\alpha]^{25}_D$ = +82.9 (c 1.0, CH_2Cl_2); 4:96 dr from (*S*)-**L**.

$[\alpha]^{25}_D$ = –43.6 (c 1.0, CH_2Cl_2); 96:4 dr from (*R*)-**L**.

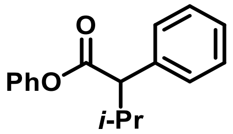

Phenyl 3-methyl-2-phenylbutanoate (Fig. 6, compound 72). The title compound was synthesized according to **GP-E** from phenyl carbonochloridate and isobutylbenzene. The product was purified by preparative thin-layer chromatography on silica gel (1:25 EtOAc/Petroleum ether). Yellow oil.

(*S, R*)-**L2**: 107 mg, 84% yield, 98% ee; (*R, S*)-**L2**: 102 mg, 80% yield, 98% ee.

HPLC analysis: The ee was determined via HPLC on a CHIRALCEL OJ-3 column (25.0% 2-PrOH in hexanes, 1.0 mL/min); retention times for compound obtained using (*S, R*)-**L**: 7.7 min (minor), 8.7 min (major).

^{1}H NMR (500 MHz, $CDCl_3$) δ 7.47 – 7.44 (m, 2H), 7.40 – 7.31 (m, 5H), 7.23 – 7.20 (m, 1H), 7.03 – 7.01 (m, 2H), 3.43 (d, J = 10.5 Hz, 1H), 2.54 – 2.46 (m, 1H), 1.22 (d, J = 6.5 Hz, 3H), 0.82 (d, J = 6.7 Hz, 3H).

^{13}C NMR (126 MHz, $CDCl_3$) δ 172.4, 150.7, 137.8, 129.3, 128.6, 128.6, 127.4, 125.7, 121.4, 60.0, 32.0, 21.5, 20.2.

FT-IR (film): 2961, 2871, 1754, 1492, 1287, 1195, 1105, 750, 698 cm^{-1}.

HRMS (ESI-MS) m/z $[M+Na]^+$ calcd for $C_{17}H_{18}O_2Na$: 277.1199, found: 277.1198.

$[\alpha]^{25}_D$ = +68.0 (c 1.0, CH_2Cl_2); 98% ee from (*S, R*)-**L**.

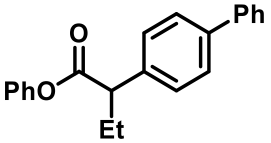

Phenyl 2-([1,1'-biphenyl]-4-yl)butanoate (Fig. 6, compound 73). The title compound was synthesized according to **GP-E** from phenyl carbonochloridate and 4-propyl-1,1'-biphenyl. The product was purified by preparative thin-layer chromatography on silica gel (1:25 EtOAc/Petroleum ether). Yellow solid (mp = 66-67 °C).

(*S, R*)-**L2**: 138 mg, 87% yield, 91% ee; (*R, S*)-**L2**: 142 mg, 90% yield, 90% ee.

HPLC analysis: The ee was determined via HPLC on a CHIRALCEL OJ-3 column (15.0% 2-PrOH in hexanes, 1.0 mL/min); retention times for compound obtained using (*S, R*)-**L**: 24.0 min (major), 29.7 min (minor).

^{1}H NMR (500 MHz, $CDCl_3$) δ 7.64 – 7.62 (m, 4H), 7.51 – 7.45 (m, 4H), 7.39 – 7.35 (m, 3H), 7.22 (t, J = 7.5 Hz, 1H), 7.04 (d, J = 7.7 Hz, 2H), 3.78 (t, J = 7.7 Hz, 1H), 2.34 – 2.25 (m, 1H), 2.02 – 1.93 (m, 1H), 1.06 (t, J = 7.4 Hz, 3H).

^{13}C NMR (126 MHz, $CDCl_3$) δ 172.5, 150.6, 140.7, 140.3, 137.6, 129.3, 128.8, 128.4, 127.4, 127.3, 127.0, 125.8, 121.4, 53.2, 26.8, 12.2.

FT-IR (film): 2965, 2929, 1754, 1592, 1486, 1193, 1139, 839, 757, 697 cm^{-1}.

HRMS (ESI-MS) *m/z* $[M+Na]^+$ calcd for $C_{22}H_{20}O_2Na$: 339.1356, found: 339.1353.

$[\alpha]^{25}_D$ = +98.4 (*c* 1.0, CH_2Cl_2); 91% ee from (*S, R*)-**L**.

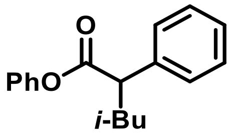

Phenyl 4-methyl-2-phenylpentanoate (Fig. 6, compound 74). The title compound was synthesized according to **GP-E** from phenyl carbonochloridate and isopentylbenzene. The product was purified by preparative thin-layer chromatography on silica gel (1:25 EtOAc/Petroleum ether). Yellow oil.

(*S, R*)-**L2**: 103 mg, 76% yield, 83% ee; (*R, S*)-**L2**: 93 mg, 69% yield, 81% ee.

HPLC analysis: The ee was determined via HPLC on a CHIRALCEL OJ-3 column (25.0% 2-PrOH in hexanes, 1.0 mL/min); retention times for compound obtained using (*S, R*)-**L**: 6.1 min (major), 7.1 min (minor).

^{1}H NMR (500 MHz, $CDCl_3$) δ 7.45 – 7.43 (m, 2H), 7.40 – 7.30 (m, 5H), 7.22 – 7.19 (m, 1H), 7.01 – 6.99 (m, 2H), 3.92 (t, *J* = 7.8 Hz, 1H), 2.15 – 2.10 (m, 1H), 1.84 – 1.78 (m, 1H), 1.66 – 1.58 (m, 1H), 1.00 (d, *J* = 6.6 Hz, 6H).

^{13}C NMR (126 MHz, $CDCl_3$) δ 172.7, 150.8, 138.9, 129.3, 128.7, 128.0, 127.4, 125.7, 121.4, 49.7, 42.4, 26.0, 22.6, 22.3.

FT-IR (film): 2956, 2928, 1755, 1592, 1492, 1455, 1195, 1114, 747, 697 cm^{-1}.

HRMS (ESI-MS) *m/z* $[M+Na]^+$ calcd for $C_{18}H_{20}O_2Na$: 291.1356, found: 291.1355.

$[\alpha]^{25}_D$ = +66.5 (*c* 1.0, CH_2Cl_2); 83% ee from (*S, R*)-**L**.

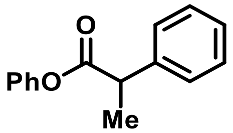

Phenyl 2-phenylpropanoate (Fig. 6, compound 75). The title compound was synthesized according to **GP-E** from phenyl carbonochloridate and ethylbenzene. The product was purified by preparative thin-layer chromatography on silica gel (1:25 EtOAc/Petroleum ether). Yellow oil.

(*S, R*)-**L2**: **6**3 mg, 56% yield, 83% ee; (*R, S*)-**L2**: **70** mg, 61% yield, 83% ee.

HPLC analysis: The ee was determined via HPLC on a CHIRALCEL OJ-3 column (25.0% 2-PrOH in hexanes, 1.0 mL/min); retention times for compound obtained using (*S, R*)-**L**: 19.0 min (minor), 20.5 min (major).

^{1}H NMR (500 MHz, $CDCl_3$) δ 7.45 – 7.29 (m, 7H), 7.23 – 7.19 (m, 1H), 7.03 – 7.00 (m, 2H), 3.99 (q, *J* = 7.1 Hz, 1H), 1.64 (d, *J* = 7.2 Hz, 3H).

^{13}C NMR (126 MHz, $CDCl_3$) δ 173.0, 150.8, 140.1, 129.3, 128.8, 127.5, 127.3, 125.7, 121.3, 45.6, 18.5.

FT-IR (film): 2979, 2919, 1754, 1591, 1491, 1195, 1138, 1071, 746, 690 cm^{-1}.

HRMS (ESI-MS) *m/z* $[M+Na]^+$ calcd for $C_{15}H_{14}O_2Na$: 249.0886, found: 249.0883.

$[\alpha]^{25}_D$ = +74.4 (*c* 1.0, $CHCl_3$); 83% ee from (*S, R*)-**L**.

IV. Synthetic Utility: Gram Scale Synthesis and Parallel Synthesis

A) Gram scale synthesis (20.0 mmol scale):

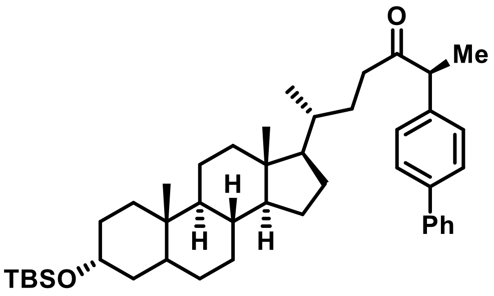

General Procedure F (GP-F): In a glovebox, a 1000 mL flask, equipped with a Teflon stir bar, was charged with Ir[dF(CF_3)ppy]$_2$(dtbbpy)PF_6 (0.22 g, 0.2 mmol, 1.0%), $NiBr_2$·glyme (0.62 g, 2.0 mmol, 10.0%), (*S*)-**L** (0.94 g, 2.6 mmol, 13.0%), NH_4Cl (1.06 g, 20.0 mmol, 1.0 equiv), Na_2HPO_4 (4.24 g, 30.0 mmol, 1.0 equiv), anhydrous *i*-PrOAc (200.0 mL), and the 4-ethylbiphenyl (10.94 g, 60.0 mmol, 3.0 equiv) was added, and followed by the addition of lithocholic acid (9.82 g, 20.0 mmol, 1.0 equiv) as a solid. Then, DMDC (3.20 mL, 30.0 mmol, 1.5 equiv) was added dropwise via a syringe. The flask was closed with a rubber stopper and wrapped with electrical tape. Next, the reaction mixture was transferred out of the glovebox, and then vacuum grease was liberally applied to cover the punctures in the septum cap. Then, the reaction mixture was stirred at 10°C in an EtOH bath for 5 min before irradiation. The reaction was stirred at 10 °C for 25 hours under blue LED irradiation with 3*40 W blue LED lamps (Kessil PR160L, 427 nm). The reaction mixture was then passed through a short pad of silica gel, with Et_2O as the eluent (~400 mL). The resulting mixture was concentrated, and the residue was purified by flash chromatography on silica gel (1:200 EtOAc/Petroleum ether). White solid.

(*S*)-**L**: 9.13 g, 70% yield, 5:95 dr.

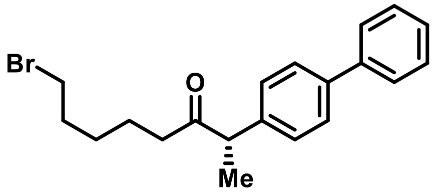

The procedure is the same as **GP-F**, except for the use of 6-bromohexanoic acid (3.90 g, 20.0 mmol, 1.0 equiv) instead of lithocholic acid.

(*S*)-**L**: 5.35 g, 75% yield, 92% ee

B) Parallel synthsis of drug analogues (>100 mg product in all cases):

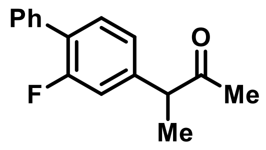

3-(2-Fluoro-[1,1'-biphenyl]-4-yl)butan-2-one (Fig. 4, compound 62). The title compound was synthesized according to **GP-B** from acetic acid and 4-ethyl-2-fluoro-1,1'-biphenyl. The product was purified by column chromatography on silica gel (1:40 EtOAc/Petroleum ether). Colorless oil.

(*S*)-**L**: 108 mg, 89% yield, 94% ee; (*R*)-**L**: 104 mg, 86% yield, 94% ee.

HPLC analysis: The ee was determined via HPLC on a CHIRALCEL OJ-3 column (15.0% 2-PrOH in hexanes, 1.0 mL/min); retention times for compound obtained using (*S*)-**L**: 10.6 min (minor), 12.6 min (major).

^{1}H NMR (400 MHz, $CDCl_3$) δ 7.56 (d, *J* = 7.7 Hz, 2H), 7.47 – 7.43 (m, 3H), 7.41 – 7.36 (m, 1H), 7.11 – 7.04 (m, 2H), 3.80 (q, *J* = 7.0 Hz, 1H), 2.13 (s, 3H), 1.45 (d, *J* = 7.0 Hz, 3H).

^{19}F NMR (471 MHz, $CDCl_3$) δ -117.3 (s, 1F).

^{13}C NMR (126 MHz, $CDCl_3$) δ 208.2, 159.8 (d, $J_{C\text{-}F}$ = 249.3 Hz), 141.8 (d, $J_{C\text{-}F}$ = 7.7 Hz), 135.3, 131.1 (d, $J_{C\text{-}F}$ = 4.1 Hz), 128.9 (d, $J_{C\text{-}F}$ = 2.7 Hz), 128.4, 127.9, 127.85, 127.7, 123.8 (d, $J_{C\text{-}F}$ = 3.2 Hz), 115.4 (d, $J_{C\text{-}F}$ = 23.2 Hz), 53.0, 28.5, 17.1.

FT-IR (film): 2977, 2931, 1716, 1622, 1483, 1417, 1355, 1267, 1173, 1130, 919, 767, 698 cm^{-1}.

HRMS (ESI-MS) *m/z* $[M+Na]^+$ calcd for $C_{16}H_{15}FONa$: 265.0999, found: 265.0997.

$[\alpha]^{25}_D$ = +171.1 (*c* 1.0, CH_2Cl_2); 94% ee from (*S*)-**L**.

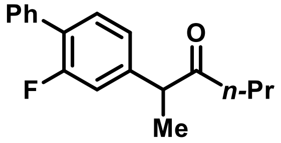

2-(2-Fluoro-[1,1'-biphenyl]-4-yl)hexan-3-one (Fig. 4, compound 63). The title compound was synthesized according to **GP-B** from butyric acid and 4-ethyl-2-fluoro-1,1'-biphenyl. The product was purified by column chromatography on silica gel (1:40 EtOAc/Petroleum ether). Colorless oil.

(*S*)-**L**: 121 mg, 90% yield, 91% ee; (*R*)-**L**: 119 mg, 88% yield, 92% ee.

HPLC analysis: The ee was determined via HPLC on a CHIRALPAK IC-3 column (5.0% 2-PrOH in hexanes, 1.0 mL/min); retention times for compound obtained using (*S*)-**L**: 5.8 min (minor), 6.2 min (major).

^{1}H NMR (400 MHz, $CDCl_3$) δ 7.55 (d, *J* = 7.5 Hz, 2H), 7.46 – 7.35 (m, 4H), 7.08 – 7.03 (m, 2H), 3.79 (q, *J* = 7.0 Hz, 1H), 2.41 (t, *J* = 7.2 Hz, 2H), 1.63 – 1.52 (m, 2H), 1.43 (d, *J* = 7.0 Hz, 3H), 0.85 (t, *J* = 7.5 Hz, 3H).

^{19}F NMR (471 MHz, $CDCl_3$) δ -117.4 (s, 1F).

^{13}C NMR (126 MHz, $CDCl_3$) δ 210.3, 159.7 (d, J_{C-F} = 249.3 Hz), 142.0 (d, J_{C-F} = 7.7 Hz), 135.3, 131.0 (d, J_{C-F} = 4.1 Hz), 128.9 (d, J_{C-F} = 2.7 Hz), 128.4, 127.70, 127.66, 127.6, 123.8 (d, J_{C-F} = 3.2 Hz), 115.5 (d, J_{C-F} = 23.2 Hz), 52.2, 43.1, 17.4, 17.1, 13.6.

FT-IR (film): 2963, 2874, 1714, 1581, 1483, 1416, 1266, 1130, 1011, 921, 875, 766, 697 cm^{-1}.

HRMS (ESI-MS) *m/z* $[M+Na]^+$ calcd for $C_{18}H_{19}FONa$: 293.1312, found: 293.1309.

$[\alpha]^{25}_{D}$ = +139.4 (*c* 1.0, CH_2Cl_2); 91% ee from (*S*)-**L**.

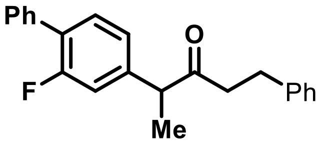

4-(3-Fluoro-[1,1'-biphenyl]-4-yl)-1-phenylpentan-3-one (Fig. 4, compound 64). The title compound was synthesized according to **GP-B** from 3-phenylpropanoic acid and 4-ethyl-3-fluoro-1,1'-biphenyl. The product was purified by column chromatography on silica gel (1:80 EtOAc/Petroleum ether). Colorless oil.

(*S*)-**L**: 133 mg, 80% yield, 91% ee; (*R*)-**L**: 129 mg, 77% yield, 92% ee.

HPLC analysis: The ee was determined via HPLC on a CHIRALPAK AD-3 column (5.0% 2-PrOH in hexanes, 1.0 mL/min); retention times for compound obtained using (*S*)-**L**: 6.5 min (major), 6.8 min (minor).

^{1}H NMR (500 MHz, $CDCl_3$) δ 7.54 – 7.51 (m, 2H), 7.46 – 7.43 (m, 2H), 7.39 – 7.34 (m, 2H), 7.26 – 7.22 (m, 2H), 7.18 – 7.15 (m, 1H), 7.10 – 7.09 (m, 2H), 7.01 – 6.94 (m, 2H), 3.74 (q, *J* = 7.0 Hz, 1H), 2.91 – 2.81 (m, 2H), 2.80 – 2.68 (m, 2H), 1.41 (d, *J* = 7.0 Hz, 3H).

^{19}F NMR (471 MHz, $CDCl_3$) δ -116.9 (s, 1F).

^{13}C NMR (126 MHz, $CDCl_3$) δ 208.8, 159.7 (d, J_{C-F} = 249.6 Hz), 141.5 (d, J_{C-F} = 7.6 Hz), 140.7, 135.2, 130.9 (d, J_{C-F} = 3.9 Hz), 128.8 (d, J_{C-F} = 2.8 Hz), 128.33, 128.27, 128.2, 127.7, 127.6, 125.9, 123.7 (d, J_{C-F} = 3.3 Hz), 115.4 (d, J_{C-F} = 23.3 Hz), 52.4, 42.5, 29.7, 17.1.

FT-IR (film): 3028, 2975, 1715, 1622, 1483, 1453, 1416, 1266, 1131, 1066, 915, 873, 766, 697 cm^{-1}.

HRMS (ESI-MS) *m/z* $[M+Na]^+$ calcd for $C_{23}H_{21}FONa$: 355.1469, found: 355.1465.

$[\alpha]^{25}_{D}$ = +73.7 (*c* 1.0, CH_2Cl_2); 91% ee from (*S*)-**L**.

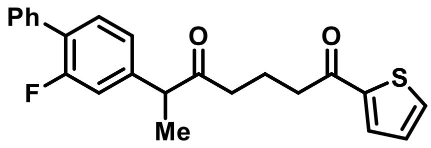

6-(2-Fluoro-[1,1'-biphenyl]-4-yl)-1-(thiophen-2-yl)heptane-1,5-dione (Fig. 4, compound 65). The title compound was synthesized according to **GP-B** from 5-oxo-5-(thiophen-2-yl)pentanoic acid and 4-ethyl-2-fluoro-1,1'-biphenyl. The product was purified by column chromatography on silica gel (1:10 EtOAc/Petroleum ether). Colorless oil.

(*S*)-**L**: 138 mg, 73% yield, 90% ee; (*R*)-**L**: 134 mg, 71% yield, 90% ee.

HPLC analysis: The ee was determined via HPLC on a CHIRALCEL OD-3 column (15.0% 2-PrOH in hexanes, 1.0 mL/min); retention times for compound obtained using (*S*)-**L**: 15.6 min (major), 16.9 min (minor).

^{1}H NMR (400 MHz, $CDCl_3$) δ 7.66 (d, *J* = 3.7 Hz, 1H), 7.58 (d, *J* = 4.9 Hz, 1H), 7.51 (d, *J* = 8.3 Hz, 2H), 7.43 (t, *J* = 7.5 Hz, 2H), 7.39 – 7.34 (m, 2H), 7.09 – 7.00 (m, 3H), 3.80 (q, *J* = 7.0 Hz, 1H), 2.92 – 2.78 (m, 2H), 2.57 (t, *J* = 6.9 Hz, 2H), 2.05 – 1.94 (m, 2H), 1.43 (d, *J* = 7.0 Hz, 3H).

^{19}F NMR (471 MHz, $CDCl_3$) δ -117.1 (s, 1F).

^{13}C NMR (101 MHz, $CDCl_3$) δ 209.6, 192.6, 159.8 (d, J_{C-F} = 311.9 Hz), 144.1, 141.7 (d, J_{C-F} = 9.7 Hz), 135.3 (d, J_{C-F} = 1.1 Hz), 133.4, 131.8, 131.0 (d, J_{C-F} = 5.1 Hz), 128.9 (d, J_{C-F} = 3.7 Hz), 128.4, 128.0, 127.9, 127.7 (d, J_{C-F} = 17.0 Hz), 123.8 (d, J_{C-F} = 4.1 Hz), 115.4 (d, J_{C-F} = 29.4 Hz), 52.3, 39.9, 37.9, 18.5, 17.3.

FT-IR (film): 2920, 2850, 1710, 1658, 1482, 1414, 1371, 1265, 1232, 1130, 766, 697 cm^{-1}.

HRMS (ESI-MS) *m/z* $[M+Na]^+$ calcd for $C_{23}H_{21}FO_2SNa$: 403.1138, found: 403.1135.

$[\alpha]^{25}_{D}$ = +97.9 (*c* 1.0, CH_2Cl_2); 90% ee from (*S*)-**L**.

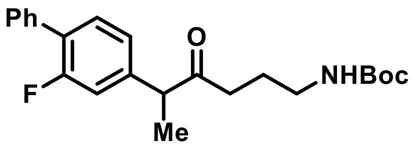

***tert*-Butyl (5-(2-fluoro-[1,1'-biphenyl]-4-yl)-4-oxohexyl)carbamate (Fig. 4, compound 66).** The title compound was synthesized according to **GP-B** from 4-((*tert*-butoxycarbonyl)amino)butanoic acid and 4-ethyl-2-fluoro-1,1'-biphenyl. The product was purified by column chromatography on silica gel (1:5 EtOAc/Petroleum ether). Colorless oil.

(*S*)-**L**: 155 mg, 80% yield, 91% ee; (*R*)-**L**: 153 mg, 80% yield, 92% ee.

HPLC analysis: The ee was determined via HPLC on a CHIRALCEL OJ-3 column (40.0% 2-PrOH in hexanes, 0.8 mL/min); retention times for compound obtained using (*S*)-**L**: 6.7 min (minor), 7.4 min (major).

^{1}H NMR (500 MHz, $CDCl_3$) δ 7.53 (d, *J* = 8.1 Hz, 2H), δ 7.44 (t, *J* = 7.6 Hz, 2H), 7.41 – 7.35 (m, 2H), 7.07 – 7.01 (m, 2H), 4.53 (s, 1H), 3.80 (q, *J* = 6.9 Hz, 1H), 3.09 – 2.99 (m, 2H), 2.46 (t, *J* = 7.2 Hz, 2H), 1.77 – 1.73 (m, 1H), 171 – 1.64(m, 1H), 1.43 (s, 3H), 1.41 (s, 9H).

^{19}F NMR (471 MHz, $CDCl_3$) δ -117.1 (s, 1F).

^{13}C NMR (126 MHz, $CDCl_3$) δ 209.8, 159.7 (d, J_{C-F} = 249.3 Hz), 155.9, 141.7 (d, J_{C-F} = 7.3 Hz), 135.2, 131.1 (d, J_{C-F} = 4.1 Hz), 128.9 (d, J_{C-F} = 2.7 Hz), 128.4, 127.8 (d, J_{C-F} = 13.7 Hz), 127.7, 123.8 (d, J_{C-F} = 3.2 Hz), 115.4 (d, J_{C-F} = 23 Hz), 79.1, 52.3, 39.7, 38.2, 28.3, 24.1, 17.3.

FT-IR (film): 2976, 2931, 1714, 1515, 1365, 1267, 1170, 767, 698 cm^{-1}.

HRMS (ESI-MS) *m/z* $[M+Na]^+$ calcd for $C_{23}H_{28}FNO_3Na$: 408.1945, found: 408.1942.

$[\alpha]^{25}_D$ = -34.2 (*c* 1.0, CH_2Cl_2); 91% ee from (*S*)-**L**.

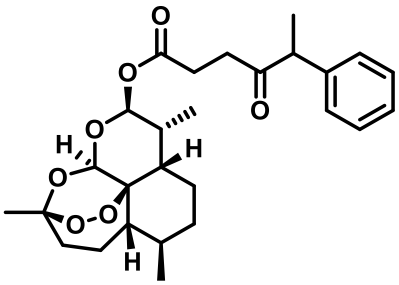

(3*R*,5a*S*,6*R*,8a*S*,9*R*,10*S*,12*R*,12a*R*)-3,6,9-Trimethyldecahydro-12*H*-3,12-epoxy[1,2]dioxepino[4,3-*i*]isochromen-10-yl 4-oxo-5-phenylhexanoate (Fig. 4, compound 67). The title compound was synthesized according to **GP-C** from artesunate and ethylbenzene. The product was purified by column chromatography on silica gel (1:4 EtOAc/Petroleum ether). Yellow oil.

(*S*)-**L**: 169 mg, 71% yield, 98:2 dr;

(*R*)-**L**: 158 mg, 67% yield, 2:98 dr.

HPLC analysis: The dr was determined via HPLC on a CHIRALCEL OJ-3 column (40.0% 2-PrOH in hexanes, 0.8 mL/min); retention times for compound obtained using (*S*)-**L**: 23.6 min (major), 31.7 min (minor).

NMR data for the product from (*S*)-**L**:

^{1}H NMR (400 MHz, $CDCl_3$) δ 8.04 (s, 1H), 7.33 (t, *J* = 7.3 Hz, 2H), 7.28 – 7.24 (m, 1H), 7.22 – 7.19 (m, 2H), 6.88 (d, *J* = 4.7 Hz, 1H), 3.77 (q, *J* = 7.0 Hz, 1H), 2.76 – 2.61 (m, 2H), 2.58 – 2.47 (m, 3H), 2.43 – 2.31 (m, 2H), 2.24 – 2.17 (m, 1H), 2.12 (s, 3H), 2.09 – 1.99 (m, 2H), 1.87 – 1.85 (m, 1H), 1.80 – 1.71(m, 2H), 1.56 – 1.46 (m, 3H), 1.41 (d, *J* = 7.0 Hz, 3H), 1.07 (d, *J* = 6.0 Hz, 3H), 1.02 (d, *J* = 6.8 Hz, 3H).

^{13}C NMR (101 MHz, $CDCl_3$) δ 212.0, 209.0, 208.5, 171.0, 159.4, 140.3, 128.9, 1278, 127.2, 91.2, 57.0, 52.7, 52.0, 41.2, 40.7, 36.3, 35.2, 34.5, 31.0, 29.8, 27.9, 20.4, 20.0, 17.3, 11.4.

NMR data for the product from (*R*)-**L**:

^{1}H NMR (400 MHz, CD_2Cl_2) δ 8.04 (s, 1H), 7.34 (t, *J* = 7.4 Hz, 2H), 7.29 – 7.26 (m, 1H), 7.25 – 7.21 (m, 2H), 6.85 (d, *J* = 4.8 Hz, 1H), 3.80 (q, *J* = 7.0 Hz, 1H), 2.79 – 2.71 (m, 1H), 2.66 – 2.54 (m, 2H), 2.52 – 2.29 (m, 4H), 2.19 – 2.13 (m, 1H), 2.08 (s, 3H), 2.06 – 1.99 (m, 2H), 1.87 – 1.85 (m, 1H), 1.79 – 1.65 (m, 2H), 1.53 – 1.45 (m, 3H), 1.39 (d, *J* = 7.0 Hz, 3H), 1.06 (d, *J* = 5.6 Hz, 3H), 1.01 (d, *J* = 6.8 Hz, 3H).

^{13}C NMR (101 MHz, CD_2Cl_2) δ 212.3, 208.8, 171.6, 159.9, 141.0, 129.2, 128.3, 127.5, 91.6, 57.3, 52.9, 52.3, 41.5, 41.0, 36.8, 35.7, 34.9, 31.3, 30.0, 28.3, 20.6, 20.4, 17.6, 11.6.

FT-IR (film): 2926, 1759, 1712, 1453, 1374, 1172, 1103, 958, 766, 702 cm^{-1}.

HRMS (ESI-MS) *m/z* $[M+Na]^+$ calcd for $C_{27}H_{36}O_7Na$: 495.2353, found: 495.2350.

$[\alpha]^{25}_{D}$ = +71.1 (*c* 1.0, CH_2Cl_2); 98:2 dr from (*S*)-**L**.

$[\alpha]^{25}_{D}$ = –71.2 (*c* 1.0, CH_2Cl_2); 1:99 dr from (*R*)-**L**.

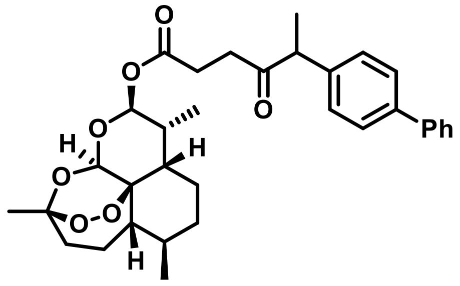

(3*R*,5a*S*,6*R*,8a*S*,9*R*,10*S*,12*R*,12a*R*)-3,6,9-Trimethyldecahydro-12*H*-3,12-epoxy[1,2]dioxepino[4,3-*i*]isochromen-10-yl 5-([1,1'-biphenyl]-4-yl)-4-oxohexanoate (Fig. 4, compound 68). The title compound was synthesized according to **GP-C** from artesunate and 4-ethyl-1,1'-biphenyl. The product was purified by column chromatography on silica gel (1:4 EtOAc/Petroleum ether). White solid (mp = 88-90 °C).

(*S*)-**L**: 202 mg, 74% yield, 97:3 dr;

(*R*)-**L**: 202 mg, 74% yield, 5:95 dr.

HPLC analysis: The dr was determined via HPLC on a CHIRALCEL OD-3 column (30.0% 2-PrOH in hexanes, 0.8 mL/min); retention times for compound obtained using (*S*)-**L**: 12.2 min (major), 25.1 min (minor).

NMR data for the product from (*S*)-**L**:

^{1}H NMR (400 MHz, $CDCl_3$) δ 8.04 (s, 1H), 7.58 – 7.54 (m, 4H), 7.42 (t, *J* = 7.6 Hz, 2H), 7.35 – 7.31 (m, 1H), 7.28 (s, 2H), 6.89 (d, *J* = 4.9 Hz, 1H), 3.82 (q, *J* = 7.0 Hz, 1H), 2.75 – 2.65 (m, 2H), 2.59 – 2.48 (m, 3H), 2.42 – 2.30 (m, 2H), 2.21 – 2.15 (m, 1H), 2.10 (s, 3H), 2.07 – 1.98 (m, 2H), 1.86 – 1.80 (m, 1H), 1.79 – 1.69 (m, 2H), 1.57 – 1.46 (m, 3H), 1.44 (d, *J* = 7.0 Hz, 3H), 1.05 (d, *J* = 6.0 Hz, 3H), 1.02 (d, *J* = 6.8 Hz, 3H).

^{13}C NMR (101 MHz, $CDCl_3$) δ 212.0, 209.0, 208.4, 171.0, 159.4, 140.4, 140.1, 139.2, 128.7, 128.2, 127.6, 127.3, 126.9, 91.2, 56.9, 52.3, 51.9, 41.1, 40.7, 36.3, 35.2, 34.5, 31.0, 29.8, 27.9, 20.4, 20.0, 17.3, 11.4.

NMR data for the product from (*R*)-**L**:

^{1}H NMR (400 MHz, CD_2Cl_2) δ 8.04 (s, 1H), 7.60 – 7.57 (m, 4H), 7.43 (t, *J* = 7.3 Hz, 2H), 7.35 (d, *J* = 7.7 Hz, 1H), 7.30 (d, *J* = 7.3 Hz, 2H), 6.86 (d, *J* = 4.2 Hz, 1H), 3.84 (q, *J* = 6.8 Hz, 1H), 2.83 – 2.75 (m, 1H), 2.72 – 2.56 (m, 2H), 2.53 – 2.38 (m, 3H), 2.36 – 2.27 (m, 1H), 2.21 – 2.13 (m, 1H), 2.07 (s, 3H), 2.03 – 1.98 (m, 2H), 1.87 – 1.84 (m, 1H), 1.77 – 1.67 (m, 2H), 1.51 (dd, *J* = 18.7, 9.9 Hz, 3H), δ 1.42 (d, *J* = 7.0 Hz, 3H), 1.05 (d, *J* = 4.5 Hz, 3H), 1.01 (d, *J* = 6.7 Hz, 3H).

^{13}C NMR (101 MHz, CD_2Cl_2) δ 212.3, 208.9, 208.8, 171.6, 160.0, 140.9, 140.4, 140.1, 129.2, 128.8, 127.9, 127.8, 127.3, 91.7, 57.4, 52.6, 52.3, 41.5, 41.0, 36.9, 35.8, 34.9, 31.4, 30.0, 28.4, 20.7, 20.5, 17.6, 11.6.

FT-IR (film): 2920, 2850, 1755, 1707, 1485, 1353, 1160, 1100, 954, 766, 698 cm^{-1}.

HRMS (ESI-MS) *m/z* $[M+Na]^+$ calcd for $C_{33}H_{40}O_7Na$: 571.2666, found: 571.2664.

$[\alpha]^{25}_{D}$ = +82.6 (*c* 1.0, CH_2Cl_2); 97:3 dr from (*S*)-**L**.

$[\alpha]^{25}_{D}$ = –67.2 (*c* 1.0, CH_2Cl_2); 5: 95 dr from (*R*)-**L**.

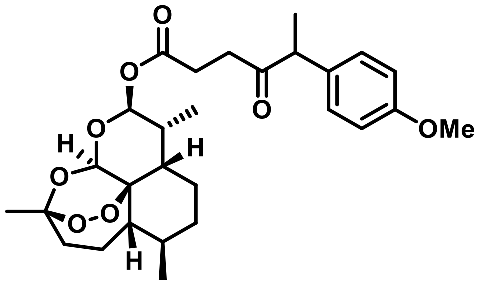

(3*R*,5a*S*,6*R*,8a*S*,9*R*,10*S*,12*R*,12a*R*)-3,6,9-Trimethyldecahydro-12*H*-3,12-epoxy[1,2]dioxepino[4,3-*i*]isochromen-10-yl 5-(4-methoxyphenyl)-4-oxohexanoate (Fig. 4, compound 69). The title compound was synthesized according to **GP-C** from artesunate and 1-ethyl-4-methoxybenzene. The product was purified by column chromatography on silica gel (1:4 EtOAc/Petroleum ether). Colorless oil.

(*S*)-**L**: 186 mg, 74% yield, 97:3 dr;

(*R*)-**L**: 184 mg, 73% yield, 2:98 dr.

HPLC analysis: The dr was determined via HPLC on a CHIRALCEL OD-3 column (30.0% 2-PrOH in hexanes, 0.8 mL/min); retention times for compound obtained using (*S*)-**L**: 10.3 min (major), 13.1 min (minor).

NMR data for the product from (*S*)-**L**:

^{1}H NMR (400 MHz, CD_2Cl_2) δ 8.05 (s, 1H), 7.13 (d, *J* = 8.7 Hz, 2H), 6.88 – 6.84 (m, 3H), 3.78 (s, 3H), 3.73 (q, *J* = 7.0 Hz, 1H), 2.70 – 2.62 (m, 2H), 2.54 – 2.46 (m, 3H), 2.43 – 2.30 (m, 2H), 2.19 – 2.14 (m, 1H), 2.08 (s, 3H), 2.06 – 1.98 (m, 2H), 1.87 – 1.84 (m, 1H), 1.76 – 1.68 (m, 2H), 1.57 – 1.44 (m, 3H), 1.35 (d, *J* = 7.0 Hz, 3H), 1.06 (d, *J* = 6.0 Hz, 3H), 1.00 (d, *J* = 6.9 Hz, 3H).

^{13}C NMR (101 MHz, CD_2Cl_2) δ 212.3, 209.1, 208.9, 171.6, 160.0, 159.3, 133.0, 129.3, 114.6, 91.6, 57.4, 55.6, 52.3, 52.1, 41.5, 41.0, 36.8, 35.5, 34.9, 31.4, 30.0, 28.4, 20.7, 20.5, 17.7, 11.6.

NMR data for the product from (*R*)-**L**:

^{1}H NMR (400 MHz, CD_2Cl_2) δ 8.03 (s, 1H), 7.12 (d, *J* = 8.6 Hz, 2H), 6.86 – 6.84 (m, 3H), 3.76 (s, 3H), 3.72 (t, *J* = 7.0 Hz, 1H), 2.76 – 2.67 (m, 1H), 2.65 – 2.53 (m, 2H), 2.51 – 2.36 (m, 3H), 2.34 – 2.28 (m, 1H), 2.18 – 2.14 (m, 1H), 2.07 (s, 3H), 2.05 – 1.97 (m, 2H), 1.86 – 1.83 (m, 1H), 1.78 – 1.67 (m, 2H), 1.53 – 1.43 (m, 3H), 1.34 (d, *J* = 7.0 Hz, 3H), 1.05 (d, *J* = 5.7 Hz, 3H), 1.00 (d, *J* = 6.9 Hz, 3H).

^{13}C NMR (101 MHz, CD_2Cl_2) δ 212.3, 209.1, 208.9, 171.6, 160.0, 159.2, 132.9, 129.2, 114.6, 91.6, 57.3, 55.6, 52.3, 52.1, 41.5, 41.0, 36.8, 35.5, 34.9, 31.3, 30.0, 28.4, 20.6, 20.4, 17.6, 11.6.

FT-IR (film): 2927, 1755, 1708, 1510, 1444, 1372, 1245, 1177, 1102, 1023, 949, 831 cm^{-1}.

HRMS (ESI-MS) *m/z* $[M+Na]^+$ calcd for $C_{28}H_{38}O_8Na$: 525.2459, found: 525.2456.

$[\alpha]^{25}_{D}$ = +79.2 (*c* 1.0, CH_2Cl_2); 97:3 dr from (*S*)-**L**.

$[\alpha]^{25}_{D}$ = –94.9 (*c* 1.0, CH_2Cl_2); 2:98 dr from (*R*)-**L**.

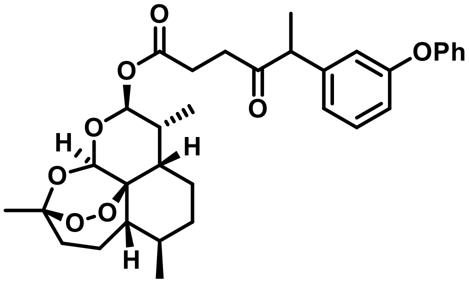

(3*R*,5a*S*,6*R*,8a*S*,9*R*,10*S*,12*R*,12a*R*)-3,6,9-Trimethyldecahydro-12*H*-3,12-epoxy[1,2]dioxepino[4,3-*i*]isochromen-10-yl 4-oxo-5-(3-phenoxyphenyl)hexanoate (Fig. 4, compound 70). The title compound was synthesized according to **GP-C** from artesunate and 1-ethyl-3-phenoxybenzene. The product was purified by column chromatography on silica gel (1:4 EtOAc/Petroleum ether). Colorless oil.

(*S*)-**L**: 214 mg, 76% yield, 98:2 dr;

(*R*)-**L**: 209 mg, 74% yield, 2:98 dr.

HPLC analysis: The dr was determined via HPLC on a CHIRALPAK AD-3 column (40.0% 2-PrOH in hexanes, 0.8 mL/min); retention times for compound obtained using (*S*)-**L**: 11.3 min (major), 12.9 min (minor).

NMR data for the product from (*S*)-**L**:

^{1}H NMR (400 MHz, CD_2Cl_2) δ 8.04 (s, 1H), 7.36 – 7.28 (m, 3H), 7.11 (t, *J* = 7.4 Hz, 1H), 7.01- 6.95 (m, 3H), 6.89 – 6.85 (m, 3H), 3.76 (q, *J* = 7.0 Hz, 1H), 2.76 – 2.67 (m, 2H), 2.53 – 2.47 (m, 3H), 2.46 – 2.37 (m, 1H), 2.36 – 2.29 (m, 1H), 2.19 – 2.15 (m, 1H), 2.07 (s, 3H), 2.05 – 1.98 (m, 2H), 1.85 – 1.81 (m, 1H), 1.77 – 1.68 (m, 2H), 1.56 – 1.42 (m, 3H), 1.36 (d, *J* = 7.0 Hz, 3H), 1.05 (d, *J* = 5.6 Hz, 3H), 1.01 (d, *J* = 6.8 Hz, 3H).

^{13}C NMR (101 MHz, CD_2Cl_2) δ 212.3, 208.9, 208.4, 171.5, 159.9, 158.1, 157.4, 143.0, 130.6, 130.2, 123.8, 123.1, 119.3, 118.8, 117.8, 91.6, 57.4, 52.8, 52.3, 41.5, 41.0, 36.9, 35.7, 34.9, 31.4, 30.0, 28.3, 20.7, 20.5, 17.5, 11.6.

NMR data for the product from (*R*)-**L**:

^{1}H NMR (400 MHz, CD_2Cl_2) δ 8.04 (s, 1H), 7.36 – 7.27 (m, 3H), 7.11 (t, *J* = 7.4 Hz, 1H), 7.00 – 6.95 (m, 3H), 6.89 – 6.85 (m, 3H), 3.77 (q, *J* = 6.8 Hz, 1H), 2.77 – 2.71 (m, 1H), 2.69 – 2.54 (m, 2H), 2.53 – 2.38 (m, 3H), 2.36 – 2.28 (m, 1H), 2.20 – 2.15 (m, *J* = 6.8 Hz, 1H), 2.07 (s, 3H), 2.03 – 1.99 (m, 2H), 1.86 – 1.84 (m, 1H), 1.79 – 1.67 (m, 2H), 1.54 – 1.44 (m, 3H), 1.37 (d, *J* = 6.9 Hz, 3H), 1.05 (d, *J* = 5.3 Hz, 3H), 1.01 (d, *J* = 6.8 Hz, 3H).

^{13}C NMR (101 MHz, CD_2Cl_2) δ 212.3, 208.9, 208.5, 171.6, 160.0, 158.1, 157.4, 143.0, 130.6, 130.2, 123.8, 123.1, 119.3, 118.7, 117.7, 91.7, 57.3, 52.8, 52.3, 41.5, 41.0, 36.9, 35.7, 34.9, 31.4, 30.0, 28.4, 20.7, 20.5, 17.5, 11.6.

FT-IR (film): 2929, 1759, 1712, 1581, 1485, 1375, 1244, 1165, 1105, 952, 757, 694 cm^{-1}.

HRMS (ESI-MS) *m*/*z* $[M+Na]^+$ calcd for $C_{33}H_{40}O_8Na$: 587.2615, found: 587.2613.

$[\alpha]^{25}_{D}$ = +68.8 (*c* 1.0, CH_2Cl_2); 98:2 dr from (*S*)-**L**.

$[\alpha]^{25}_{D}$ = –117.2 (*c* 1.0, CH_2Cl_2); 2:98 dr from (*R*)-**L**.

V. Mechanistic Studies

A. Kinetic isotope effect experiments

Parallel reactions:

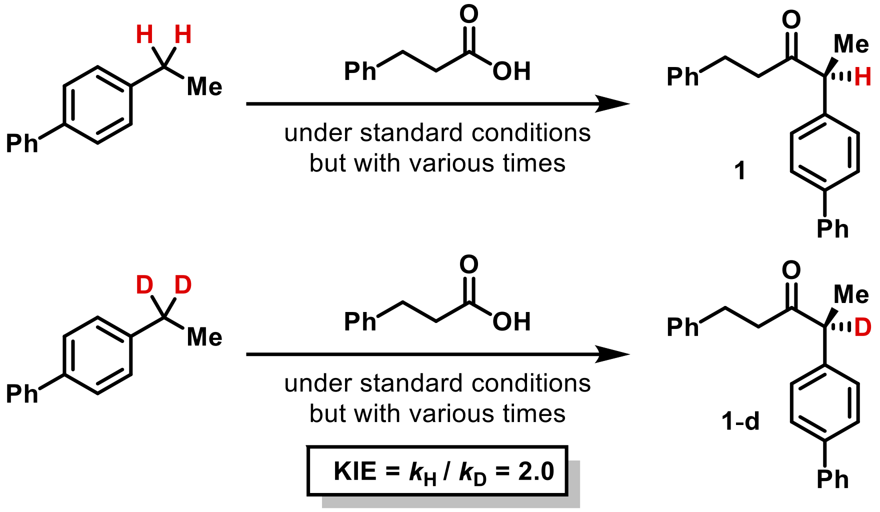

Supplementary Figure 2.

Given the reaction mixture of the enantioselective benzylic C(sp^3)–H acylation is heterogeneous, the reactions were carried out based on the standard conditions at various time independently.

The procedure is the same as **GP-B**, except for the following changes: the dodecane (110 μL, 0.50 mmol) was added as an internal standard. The reaction was stirred under blue LEDs irradiation for the indicated time. All the reactions were quenched through a small plug of silica gel, which was flushed with Et_2O. The yield was determined via GC analysis with dodecane as an internal standard (see Supplementary Figure 3 & 4). A primary kinetic isotope effect (KIE = 0.1302/0.0639 = 2.0) was obtained by parallel reactions.

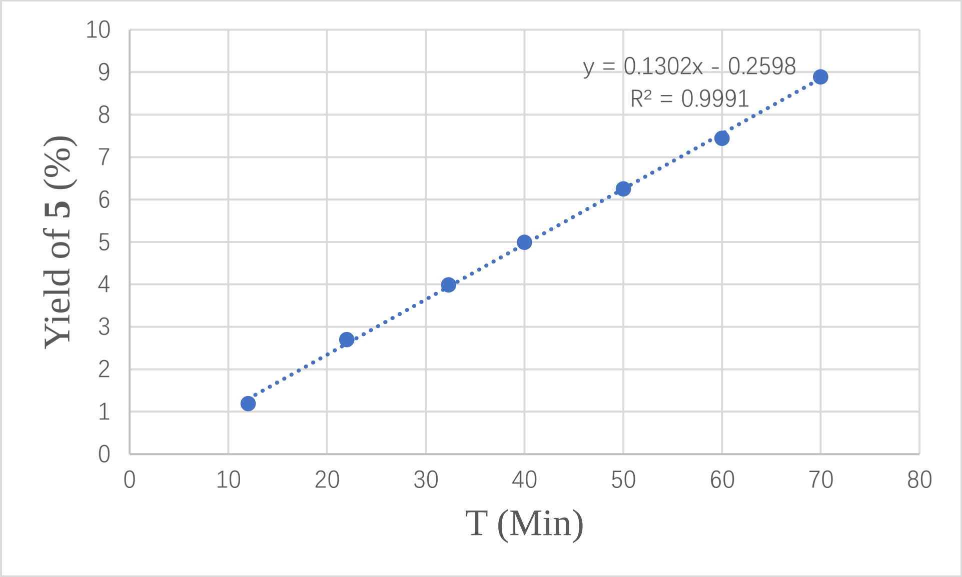

Supplementary Figure 3.

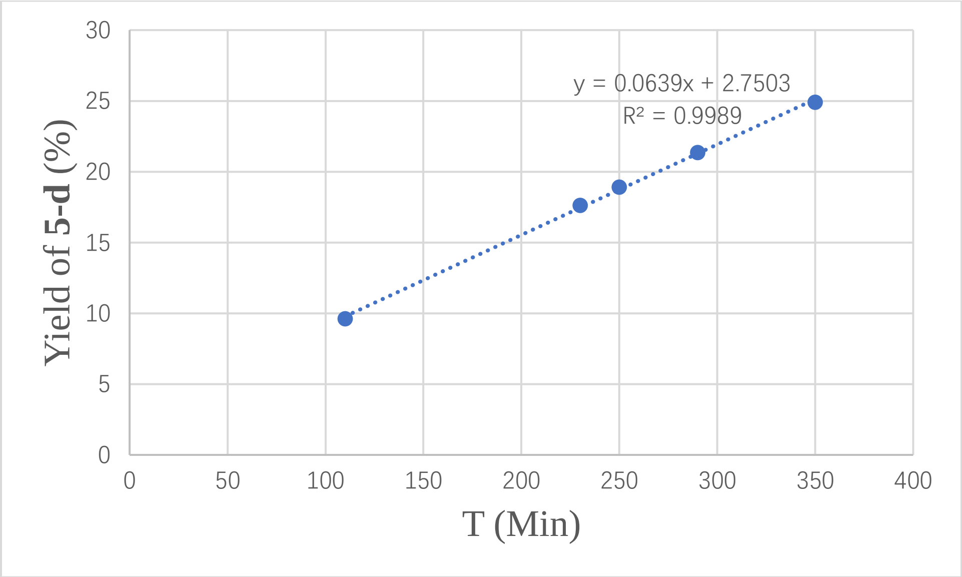

Supplementary Figure 4.

Competition reactions:

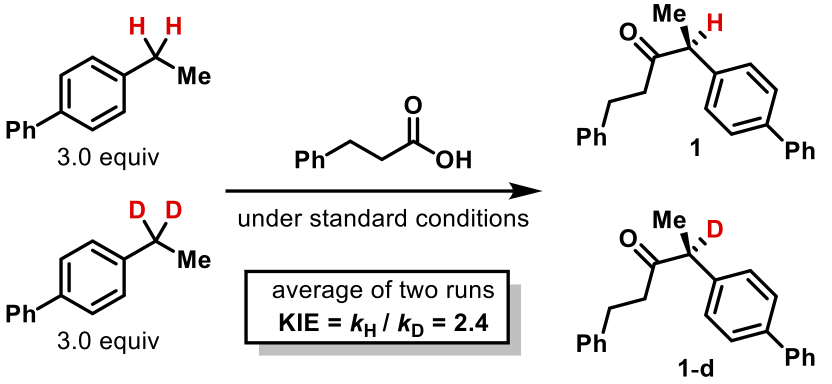

Ratio of the Product

Rxn.	**1**	**1-d**	k_H / k_D
1	0.71	0.29	2.4
2	0.70	0.30	2.3

Supplementary Figure 5.

The procedure is the same as **GP-A**, and the reactions were stirred under blue LEDs irradiation for 40 min and 200 min, respectively. All the reactions were quenched through a small plug of silica gel, which was flushed with Et_2O. The yield was determined via GC analysis with dodecane as an internal standard. The remainder of the filtrate was concentrated via rotary evaporation, and the pure product was isolated by preparative thin-layer chromatography on silica gel (1:40 EtOAc/hexanes) to give the mixture of **1** and **1-d** as a white solid.

The KIE ratio was determined by quantitative ^{1}H-NMR spectroscopy (see Supplementary Figure 6).

First run: KIE = 2.4 (6% yield); second run: KIE = 2.3 (39% yield).

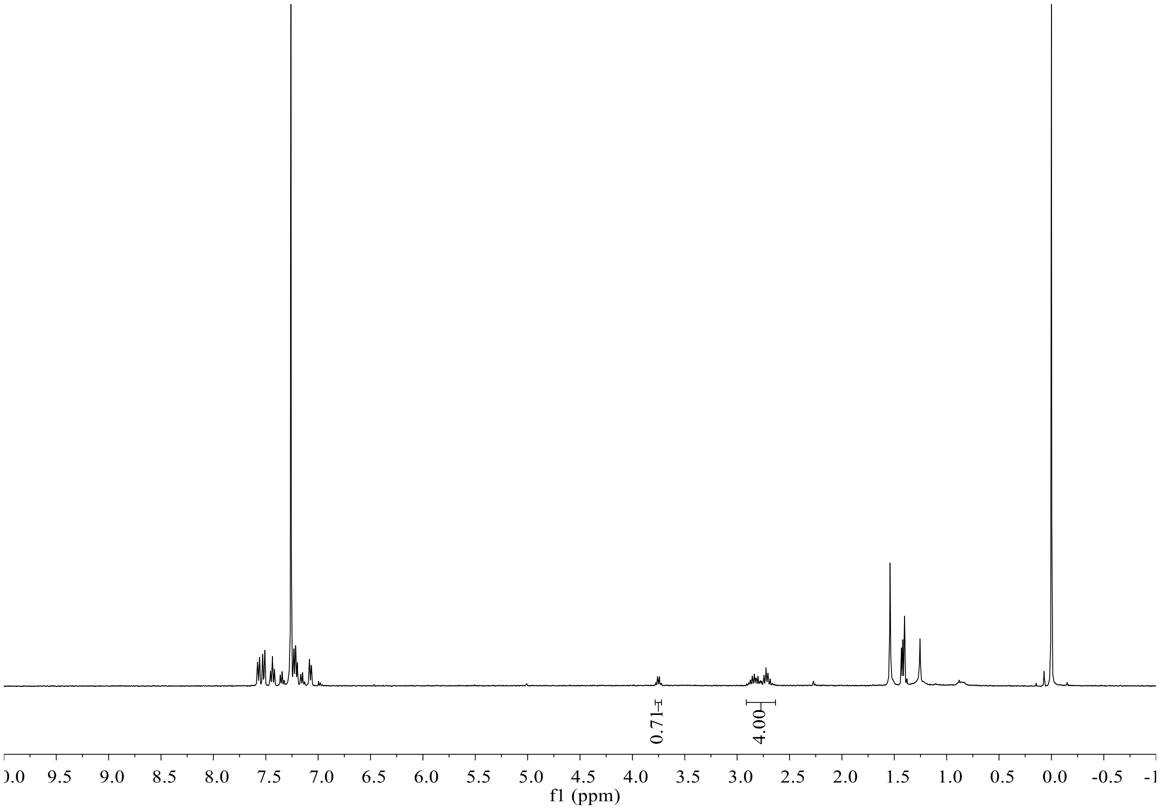

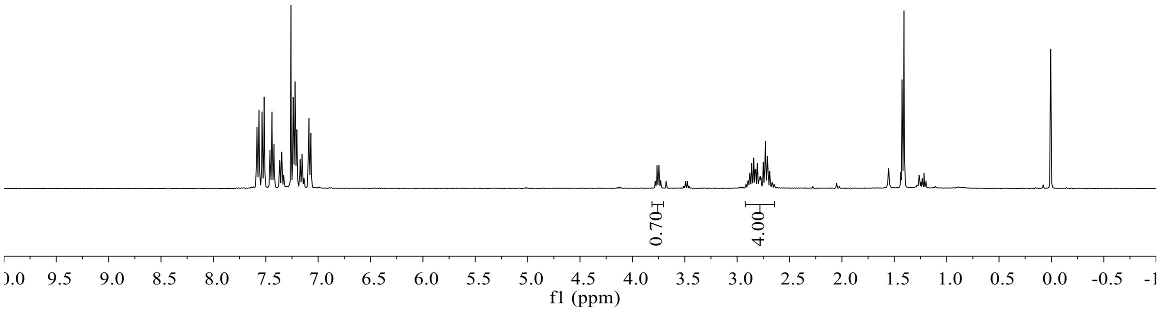

Supplementary Figure 6.

B. Benzylic radical trapping experiments

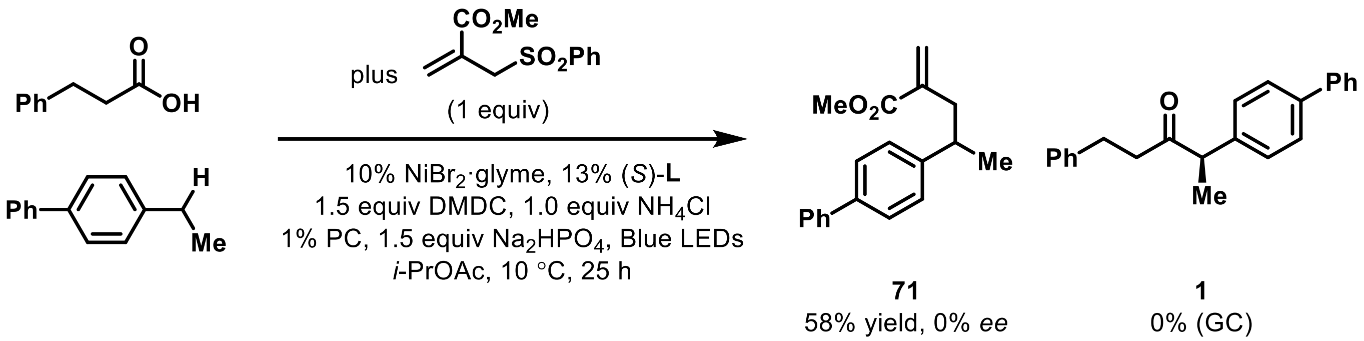

Supplementary Figure 7.

The procedure is the same as **GP-A**, except for the following changes: a solution of the methyl 2-((phenylsulfonyl)methyl)acrylate (24.0 mg, 1.0 M solution in *i*-PrOAc, 0.10 mmol, 1.0 equiv) was added via a 250 μL microsyringe.

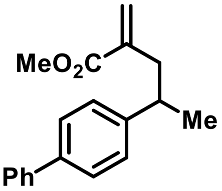

Mthyl 4-([1,1'-biphenyl]-4-yl)-2-methylenepentanoate (Fig. 5, compound 71). The title compound was synthesized according to **GP-A** from 4-ethylbiphenyl and methyl 2-((phenylsulfonyl)methyl)acrylate. The pure product was isolated by preparative thin-layer chromatography on silica gel (1: 25 EtOAc/hexanes) to give a white solid (16.1 mg, 58% yield).

^{1}H NMR (500 MHz, $CDCl_3$) δ 7.60 – 7.57 (m, 2H), 7.54 – 7.52 (m, 2H), 7.45 – 7.40 (m, 2H), 7.35 – 7.31 (m, 1H), 7.28 – 7.27 (m, 1H), 6.13 (d, *J* = 1.5 Hz, 1H), 5.42 (d, *J* = 1.4 Hz, 1H), 3.74 (s, 3H), 3.07 – 3.30 (m, 1H), 2.69 – 2.64 (m, 1H), 2.59 – 2.54 (m, 1H), 1.30 (d, *J* = 7.0 Hz, 3H).

^{13}C NMR (126 MHz, $CDCl_3$) δ 167.7, 145.6, 141.0, 139.0, 138.8, 128.7, 127.5, 127.0, 126.99, 126.96, 126.7, 51.8, 40.9, 38.4, 21.3.

FT-IR (film): 3027, 2958, 1719, 1629, 1486, 1438, 1304, 1201, 1150, 947, 838, 765, 697 cm^{-1}.

HRMS (ESI-MS) *m/z* $[M+Na]^+$ calcd for $C_{19}H_{20}O_2Na$: 303.1356, found: 303.1353.

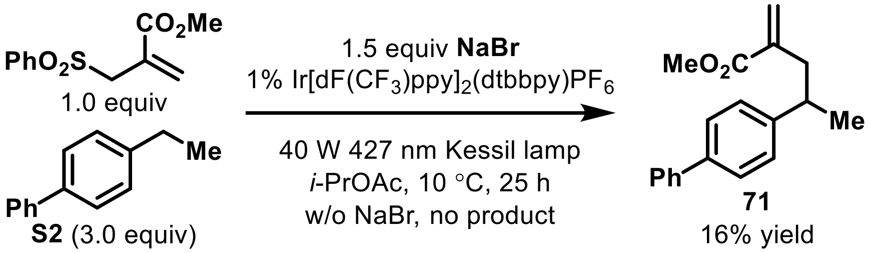

In a glovebox, Ir[dF(CF_3)ppy]$_2$(dtbbpy)PF_6 (1.1 mg, 0.001 mmol, 1%), NaBr (15.4 mg, 0.15 mmol, 1.5 equiv), Na_2HPO_4 (21.3 mg, 0.15 mmol, 1.5 equiv), a Teflon stir bar, and anhydrous *i*-PrOAc (1.0 mL) were added sequentially to a 4 mL vial. Next, a solution of the 4-ethylbiphenyl (150 µL, 2.0 M solution in *i*-PrOAc, 0.30 mmol, 3.0 equiv) was added via a 250 µL microsyringe. The vial was sealed with a septum cap and wrapped with electrical tape. Then, a solution of the methyl 2-((phenylsulfonyl)methyl)acrylate (100 µL, 24.0 mg, 1.0 M solution in *i*-PrOAc, 0.10 mmol, 1.0 equiv) was added via a 100 µL microsyringe. Next, the vial was transferred out of the glovebox, and then vacuum grease was liberally applied to cover the entire top of the septum cap. Then, the reaction mixture was stirred at 10 °C in an EtOH bath for 5 min before being irradiated with a 40 W blue LED lamp (Kessil PR160L, 427 nm). The reaction was stirred under irradiation at 10 °C for 25 hours. Next, the lamp was turned off and the resulting mixture was allowed to warm to room temperature, and then dodecane (22 µL, 0.10 mmol) was added as an internal standard. The mixture was filtered through a small plug of silica gel, which was flushed with Et_2O (~6 mL). A portion of the filtrate (~0.1 mL) was diluted with acetone (total volume: ~1 mL) and analyzed via GC, and the remainder of the filtrate was concentrated via rotary evaporation, and the pure product was isolated by preparative TLC on silica gel (1:25 EtOAc/hexanes). The yield was determined via GC analysis with dodecane as an internal standard.

VI. Assignment of Absolute Configuration

The configuration of the coupling product **59** illustrated in Figure 3 prepared with (*R*)-**L**, was determined via X-ray crystallography.

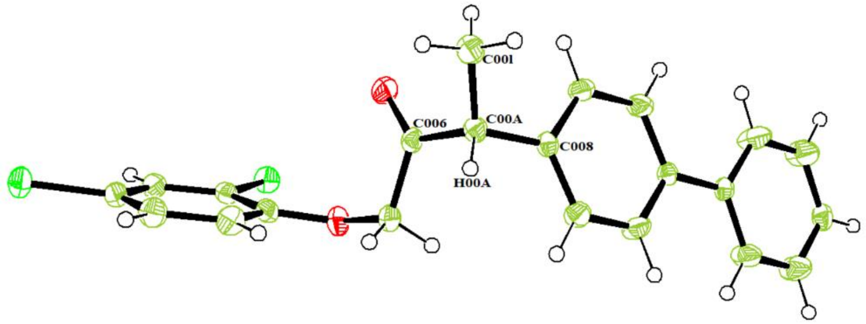

Supplementary Figure 8. Thermal ellipsoid plot at the 50% probability level.

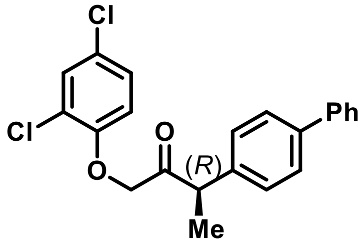

(*R*)-3-([1,1'-Biphenyl]-4-yl)-1-(2,4-dichlorophenoxy)butan-2-one. X-ray quality crystals were obtained by slow evaporation of a saturated solution in hexane and *i*-PrOH of a sample synthesized using (*R*)-**L**. All measurements were made on a 'Bruker APEX-II CCD' diffractometer with filtered Cu-Kα radiation at a temperature of 293 K. Using Olex2, the structure was solved with the ShelXT structure solution program using direct methods and refined with the ShelXL refinement package using least squares minimization.[2,3] The absolute stereochemistry was determined on the basis of the absolute structure parameter.

Supplementary Table 2. Crystal data and structure refinement for the product 59.

Identification code	**Compound 59**	
Empirical formula	$C_{22}H_{18}Cl_2O_2$	
Formula weight	385.29	
Temperature	150.00(10) K	
Wavelength	1.54184 Å	
Crystal system	orthorhombic	
Space group	$P2_12_12_1$	
Unit cell dimensions	a = 5.37670(10) Å	α= 90°
	b = 8.17780(10) Å	β= 90°
	c = 41.0081(5) Å	γ = 90°
Volume	1803.11(5) $Å^3$	
Z	4	
Density (calculated)	1.4192 g/cm^3	
Absorption coefficient	3.345 mm^{-1}	
F(000)	805.0	
Theta range for data collection	8.62 to 143.06°.	
Index ranges	$-3 \le h \le 6$, $-9 \le k \le 9$, $-49 \le l \le 49$	
Reflections collected	15998	
Independent reflections	3388 [R(int) = 0.0443, R(sigma) = 0.0320]	
Data / restraints / parameters	3388 / 0 / 236	
Goodness-of-fit on F^2	1.042	
Final R indices [I>2sigma(I)]	R1 = 0.0292, wR2 = 0.0760	
R indices (all data)	R1 = 0.0304, wR2 = 0.0767	
Absolute structure parameter [Flack]	-0.010(11)	
Largest diff. peak and hole	0.17 and -0.19 e.$Å^{-3}$	

The configuration of the coupling product **20** illustrated in **Fig. 2** was determined via comparison of optical rotation with the literature.

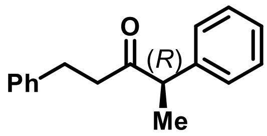

(*R*)-1,4-Diphenylpentan-3-one (20). $[\alpha]^{25}_{D} = -142.5$ (*c* 1.0, CH_2Cl_2); 93% ee from (*R*)-**L**. Lit for **R** isomer: $[\alpha]^{26}_{D} = -407.4$ (*c* 0.2, CH_2Cl_2, 90% ee).[4]

The configuration of the coupling product **75** illustrated in **Fig. 6** was determined via comparison of optical rotation with the literature.

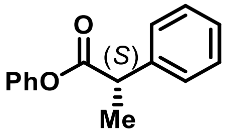

Phenyl (*S*)-2-phenylpropanoate (75). $[\alpha]^{25}_{D} = +74.4$ (*c* 1.0, $CHCl_3$); 83% ee from (*S, R*)-**L**. Lit for *R*-isomer: $[\alpha]^{20}_{D} = +85.3$ (*c* 1.4, $CHCl_3$, 92% ee).[5]

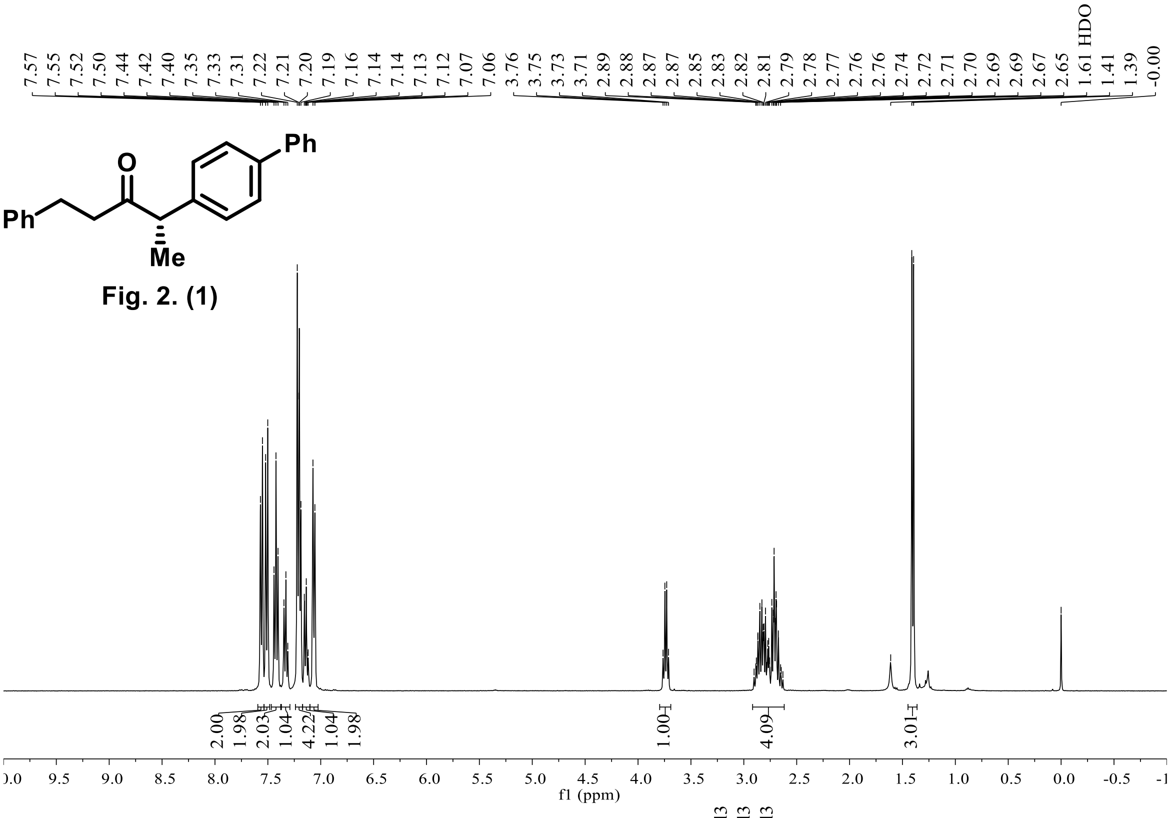

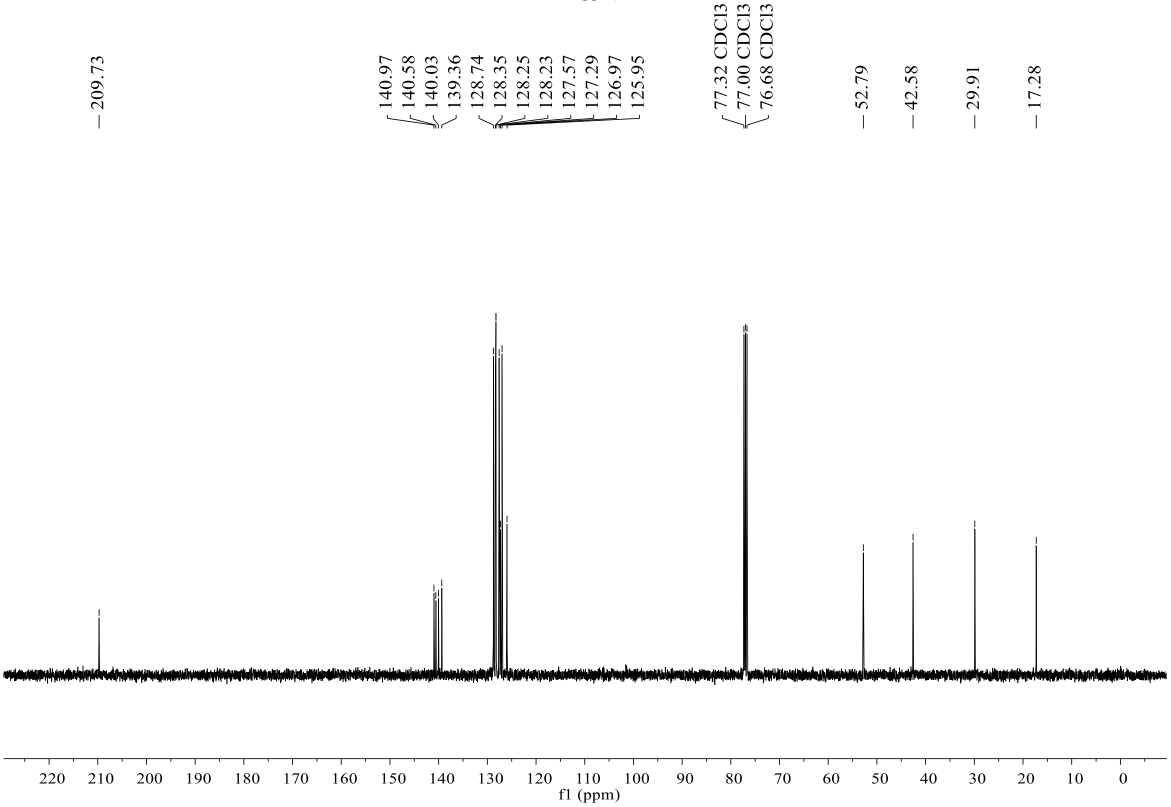

Supplementary Figure 9. ^{1}H NMR and ^{13}C NMR spectrum of **1.**

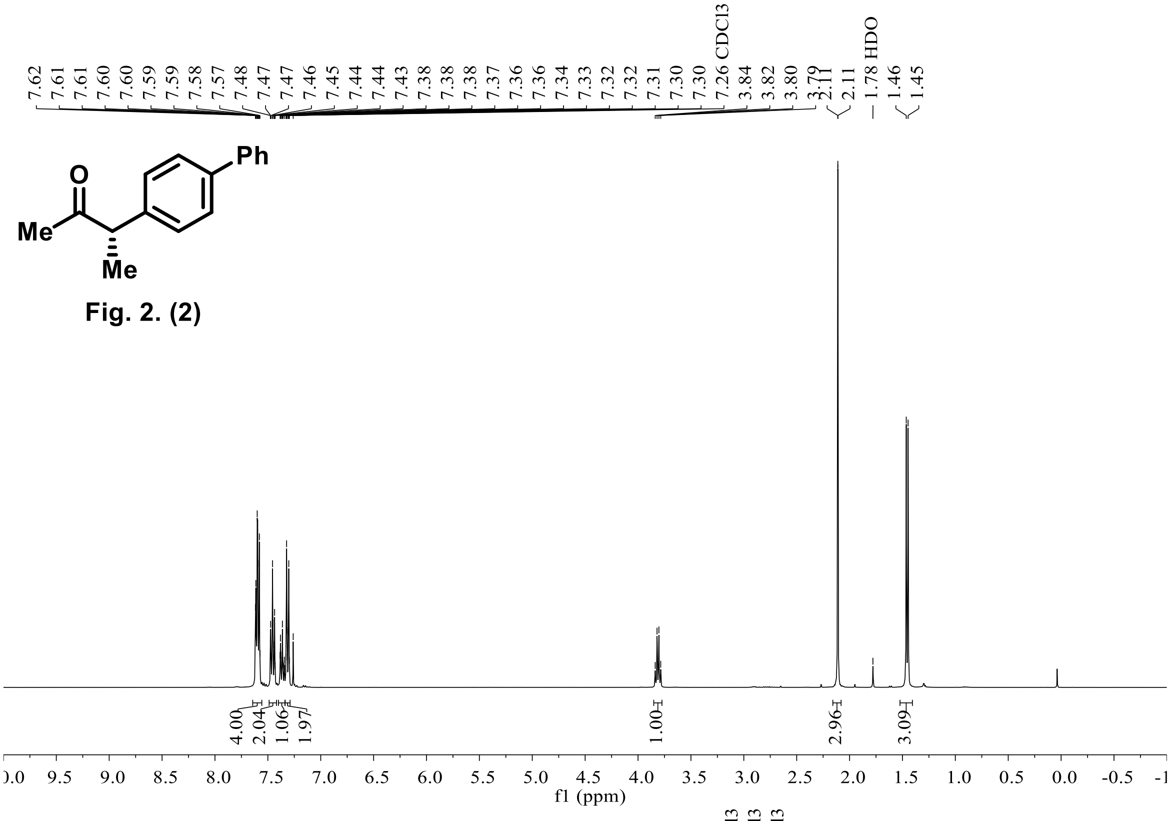

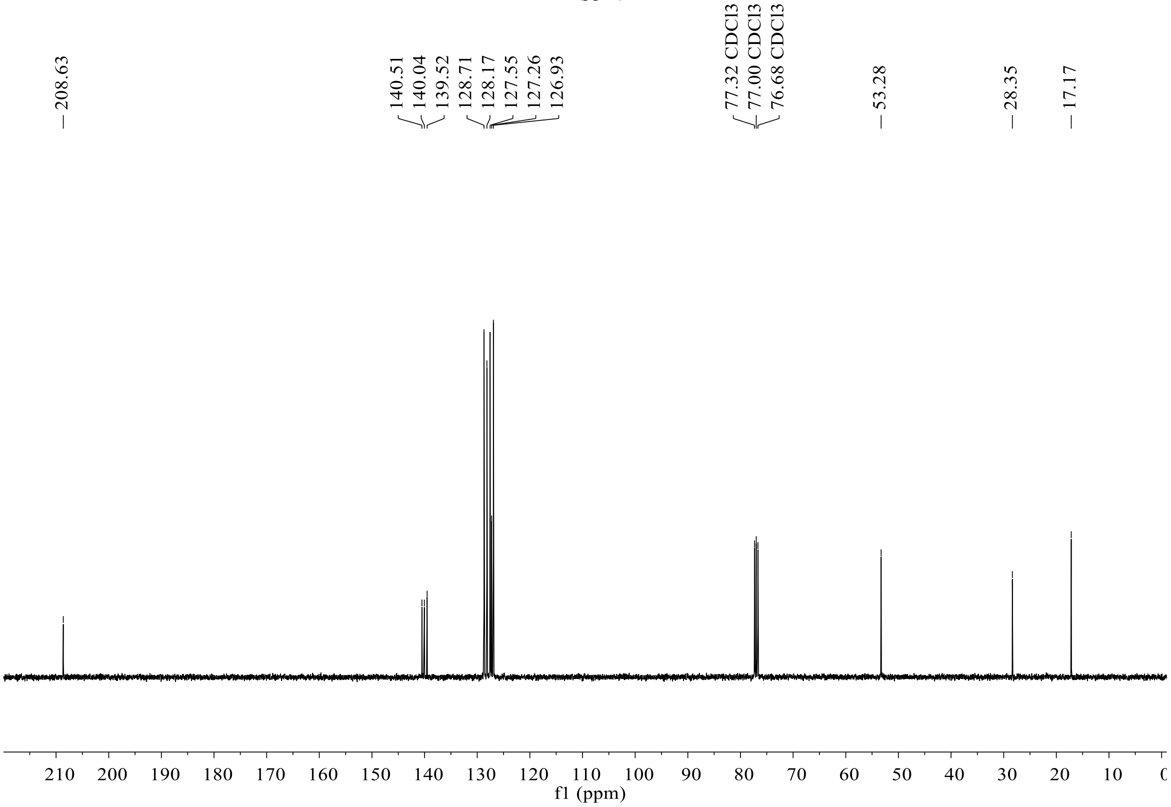

Supplementary Figure 10. ^{1}H NMR and ^{13}C NMR spectrum of **2**.

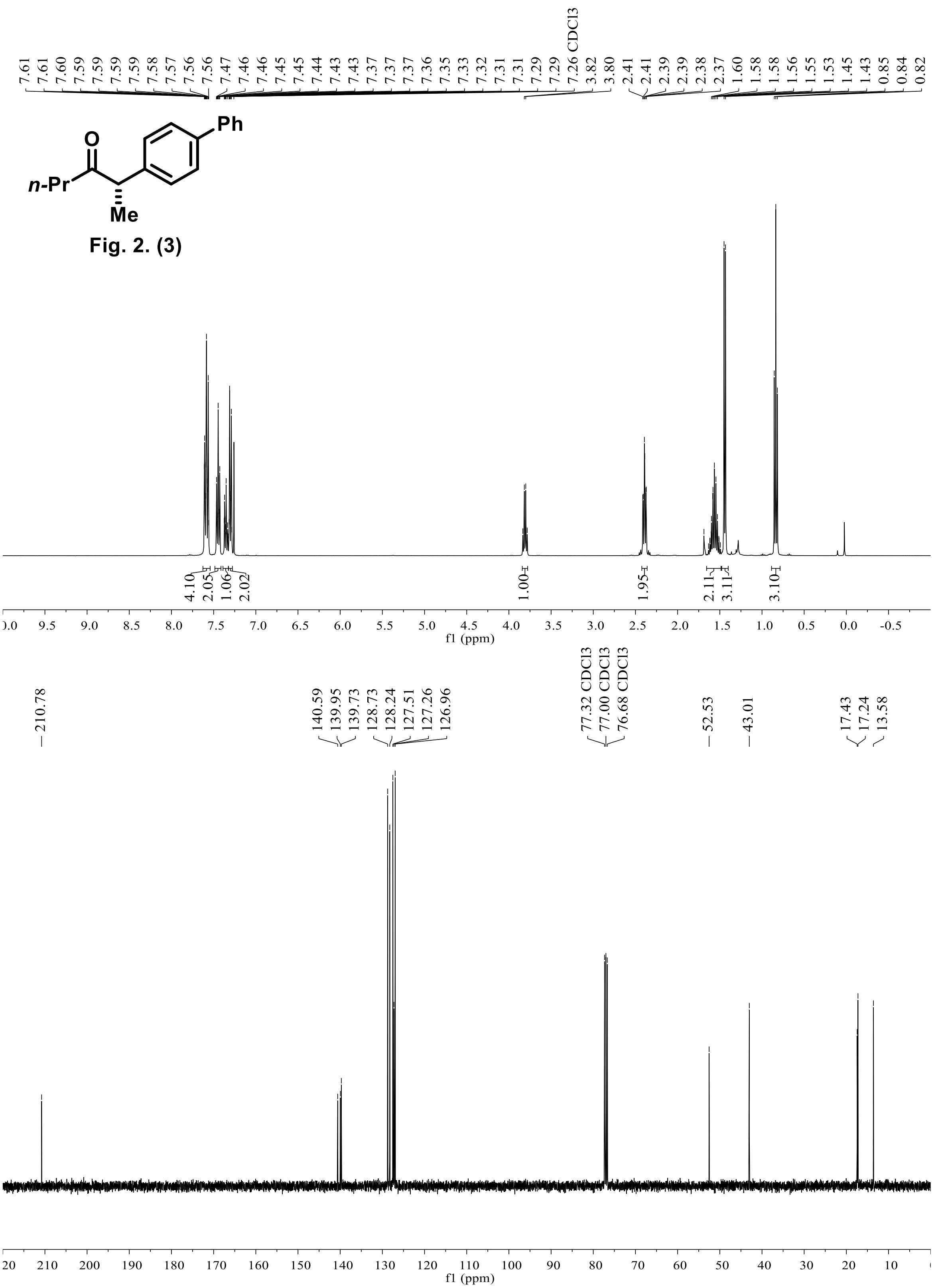

Supplementary Figure 11. ^{1}H NMR and ^{13}C NMR spectrum of **3**.

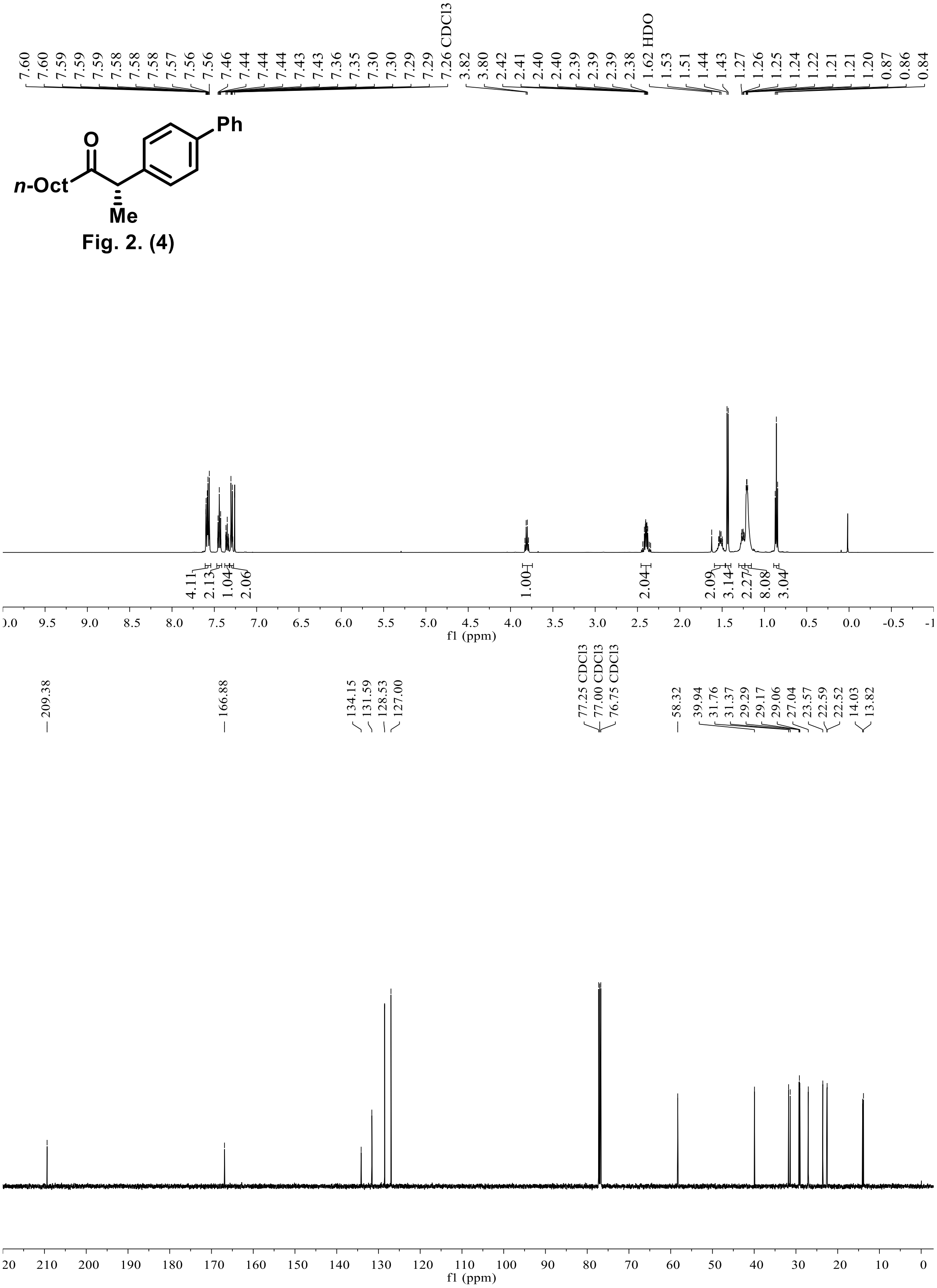

Supplementary Figure 12. ^{1}H NMR and ^{13}C NMR spectrum of **4**.

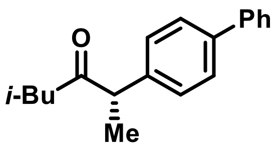

Fig. 2. (5)

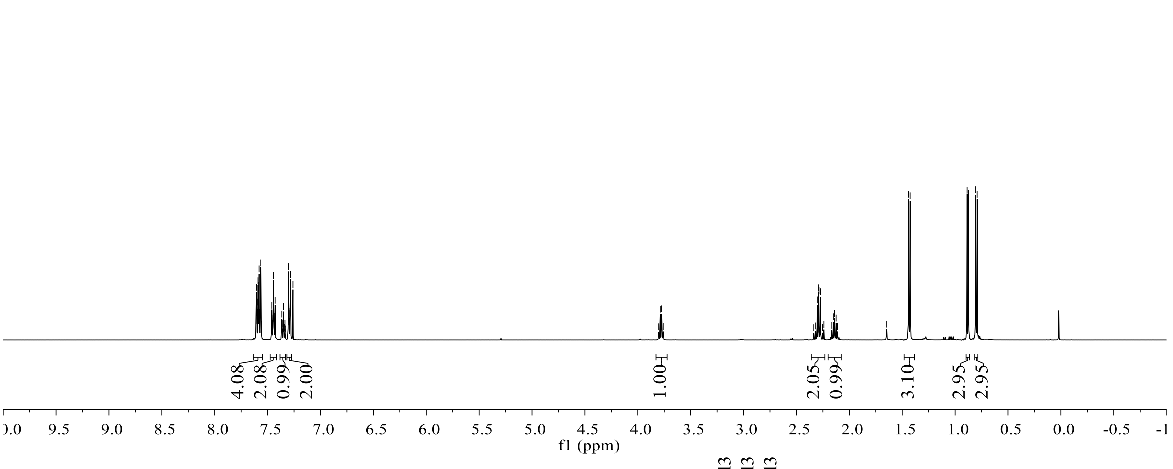

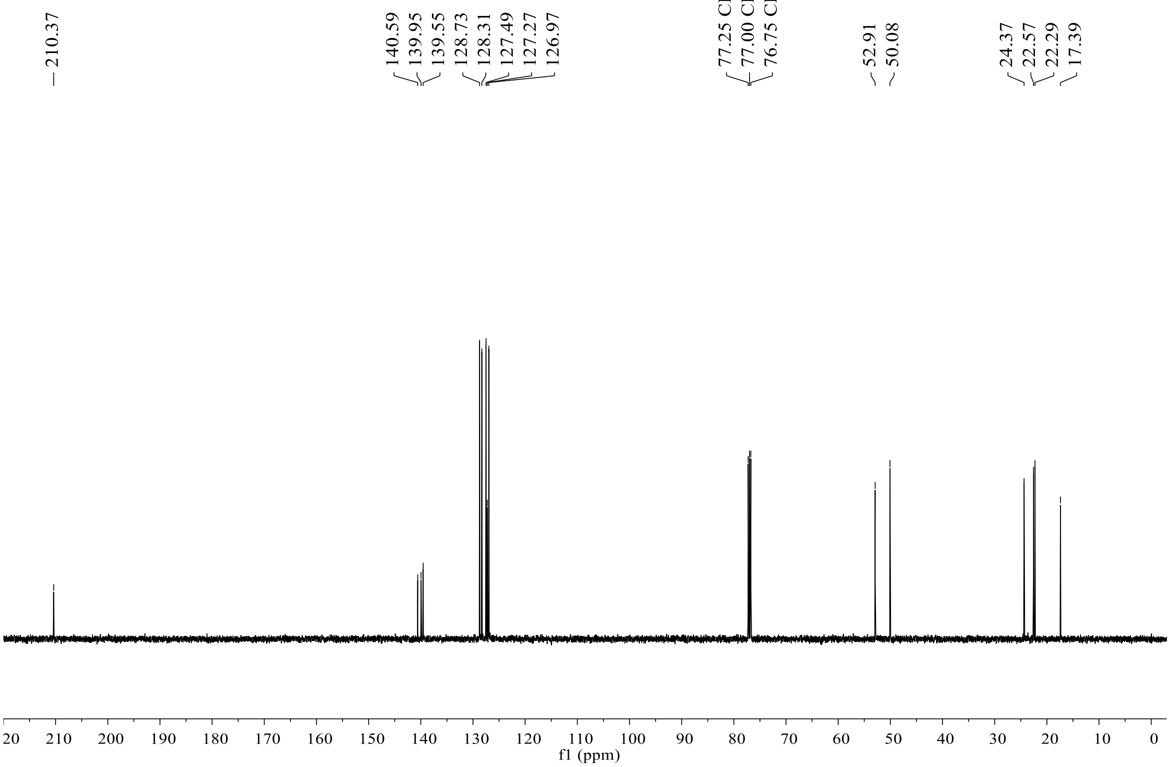

Supplementary Figure 13. ^{1}H NMR and ^{13}C NMR spectrum of **5**.

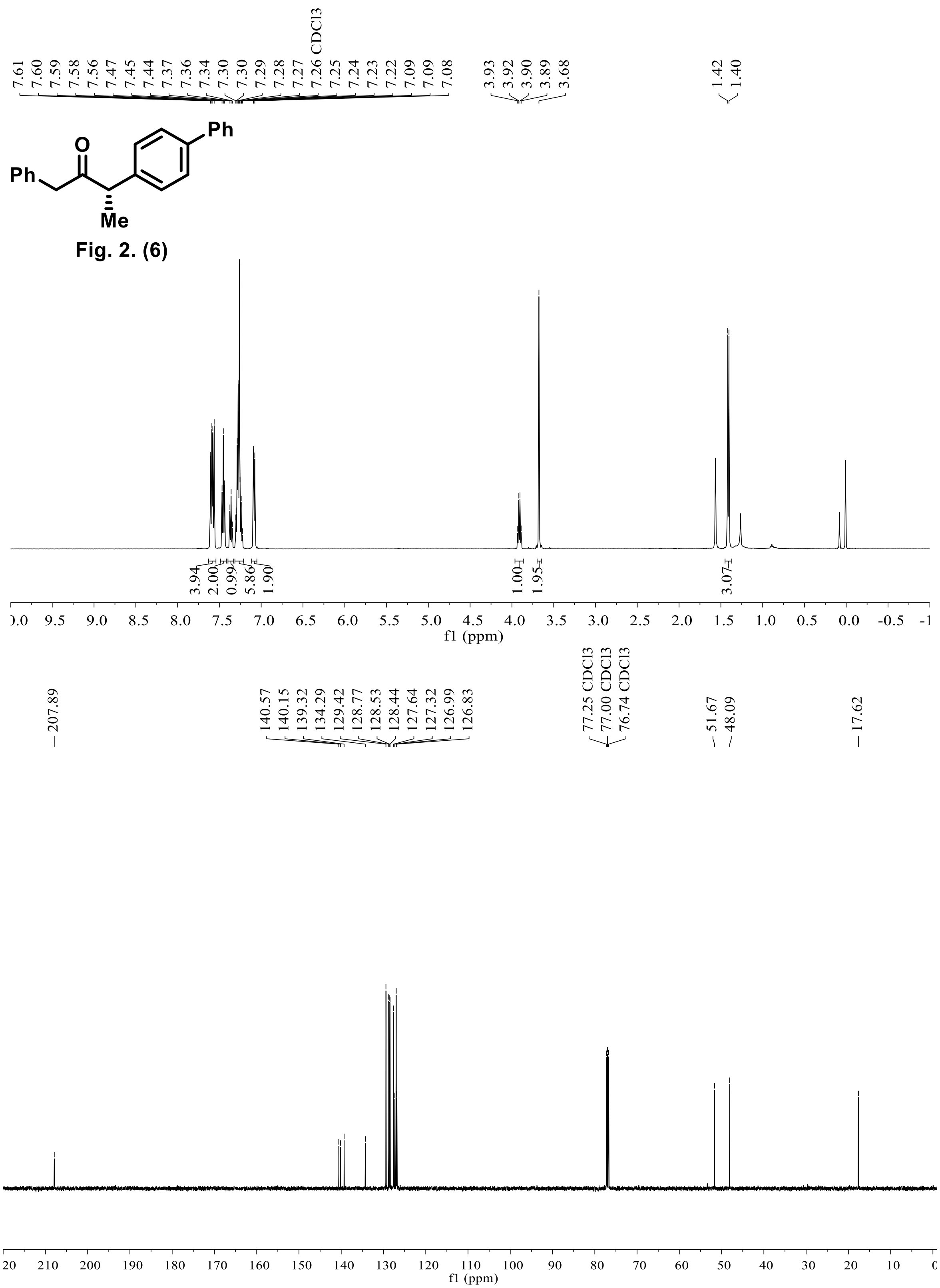

Supplementary Figure 14. ^{1}H NMR and ^{13}C NMR spectrum of **6.**

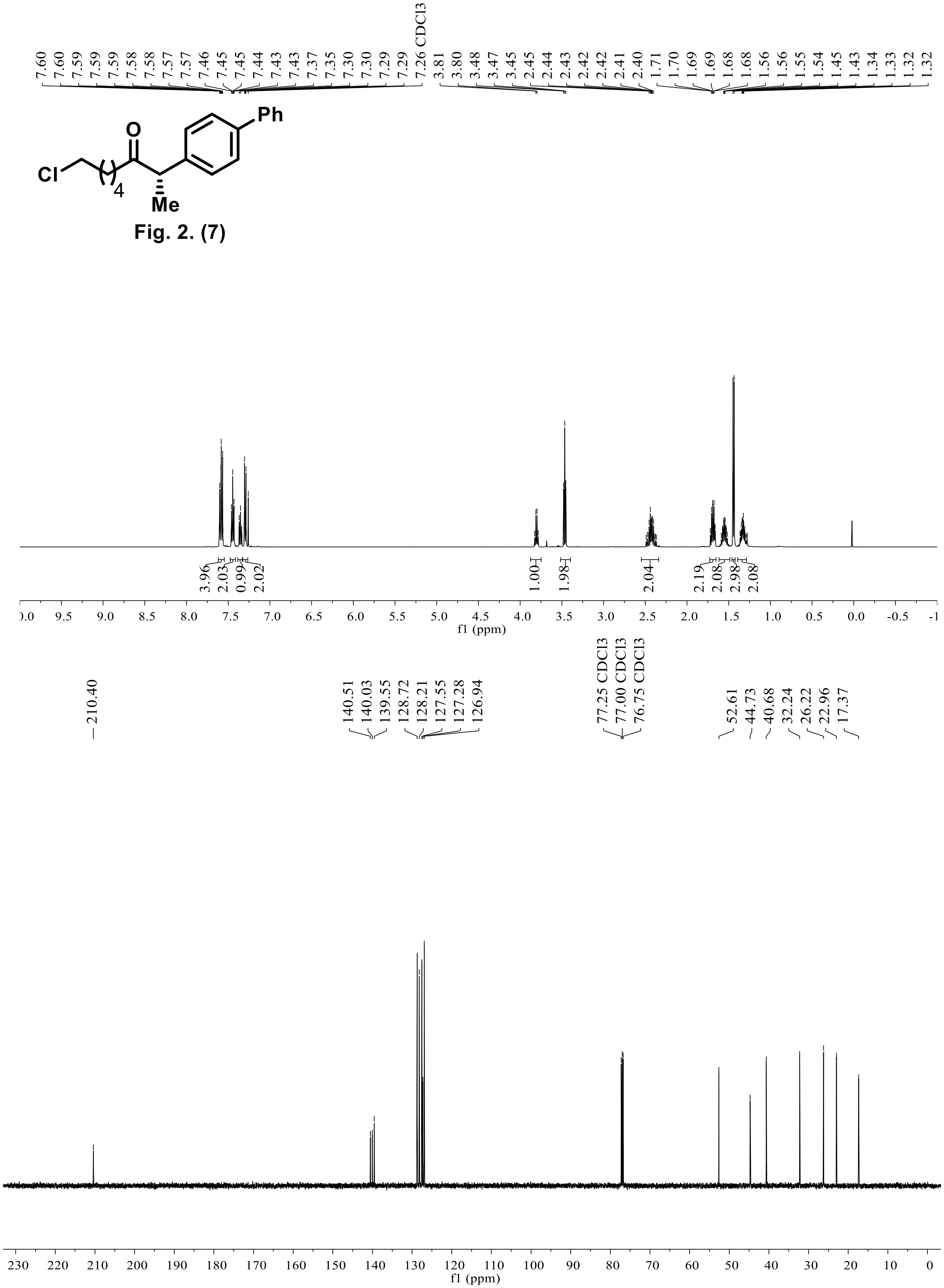

Supplementary Figure 15. ^{1}H NMR and ^{13}C NMR spectrum of **7**.

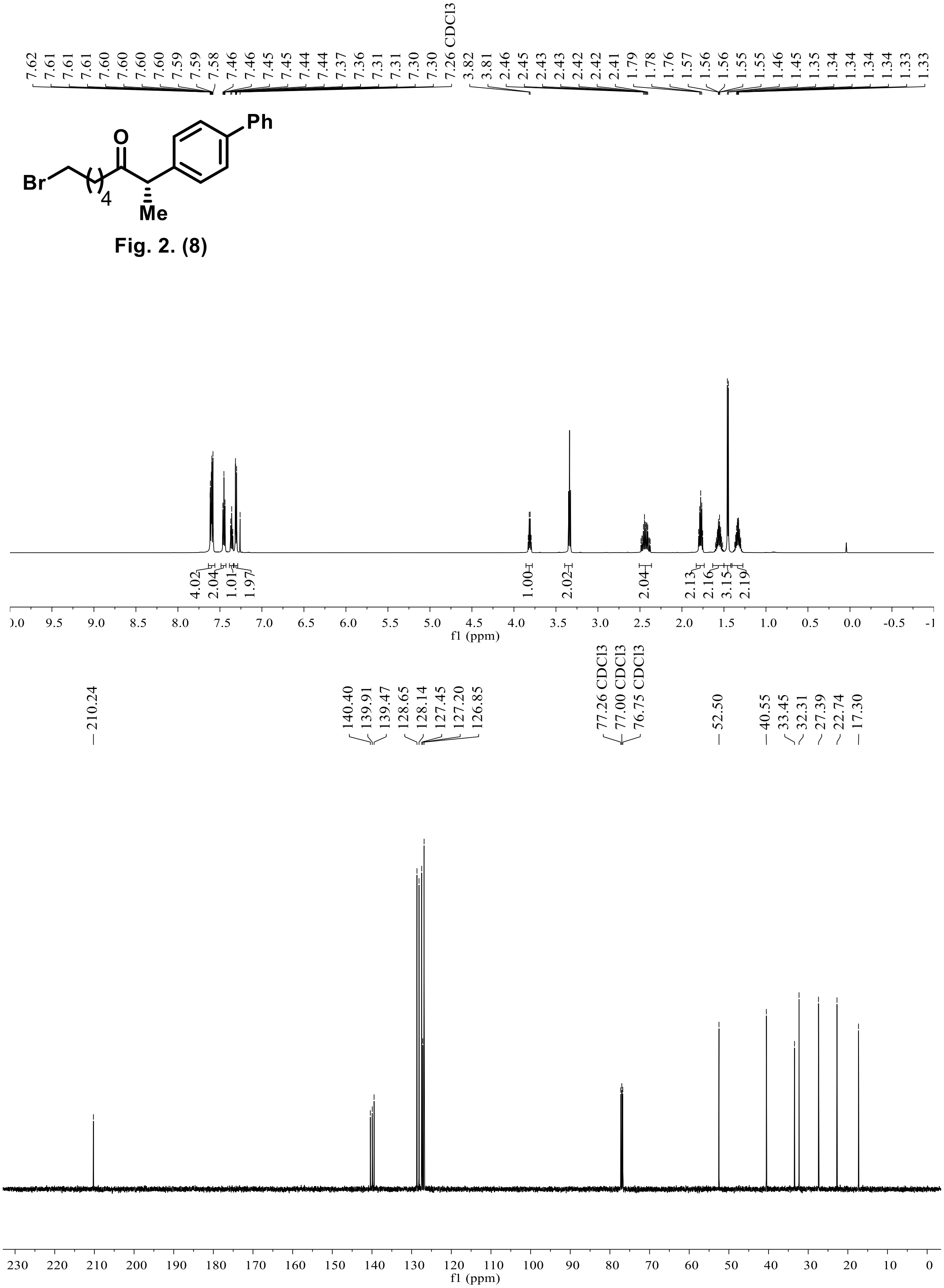

Supplementary Figure 16. ^{1}H NMR and ^{13}C NMR spectrum of **8**.

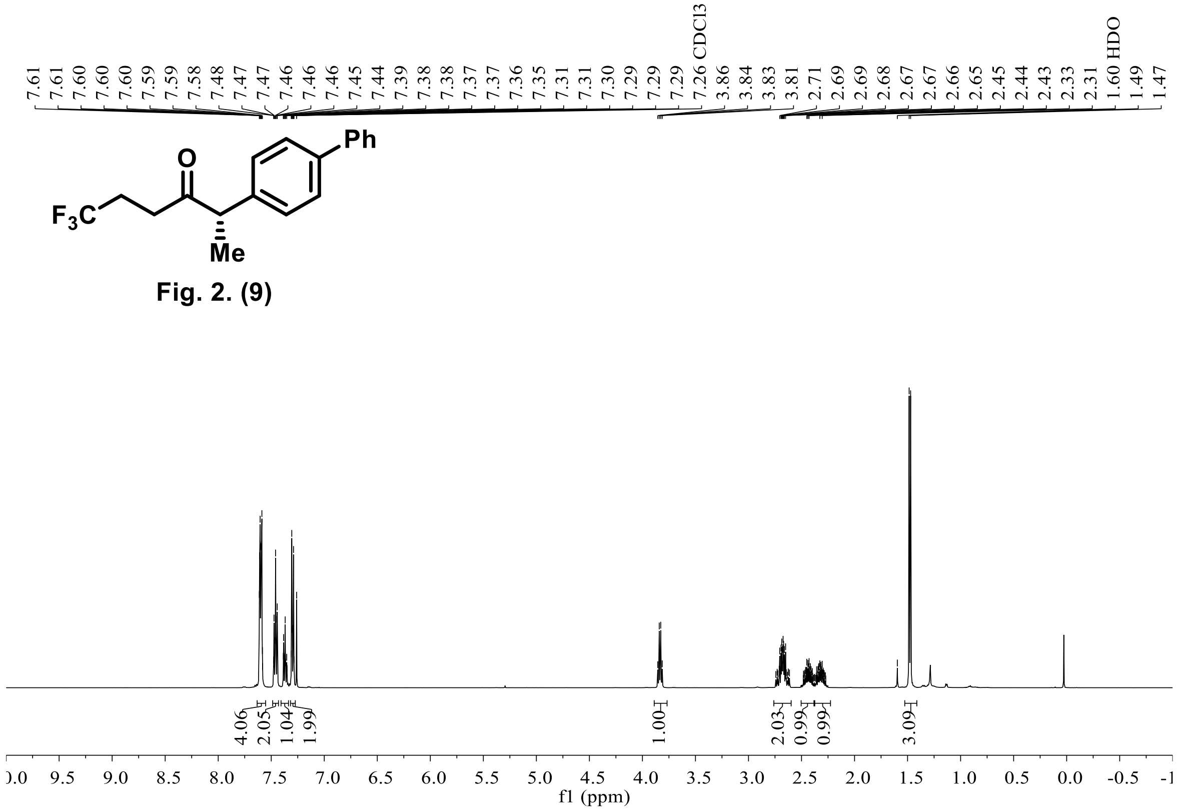

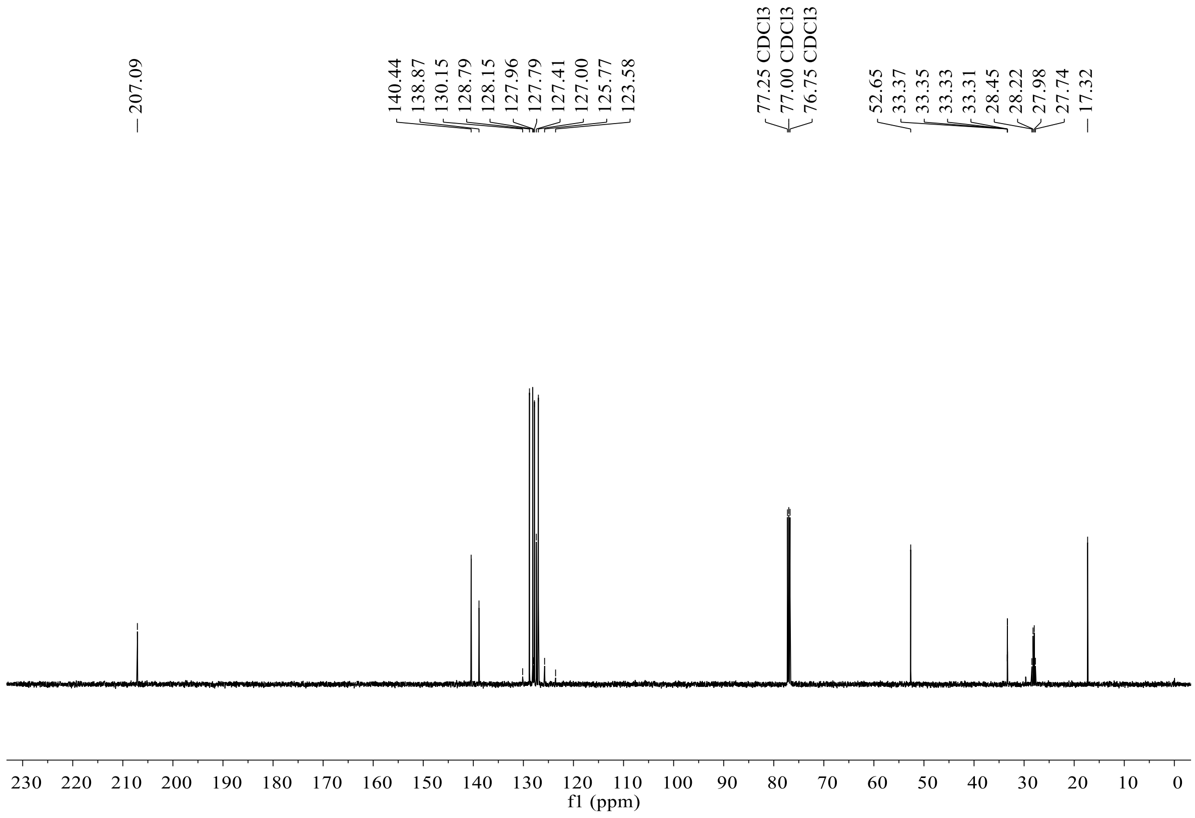

Supplementary Figure 17. ^{1}H NMR and ^{13}C NMR spectrum of **9**.

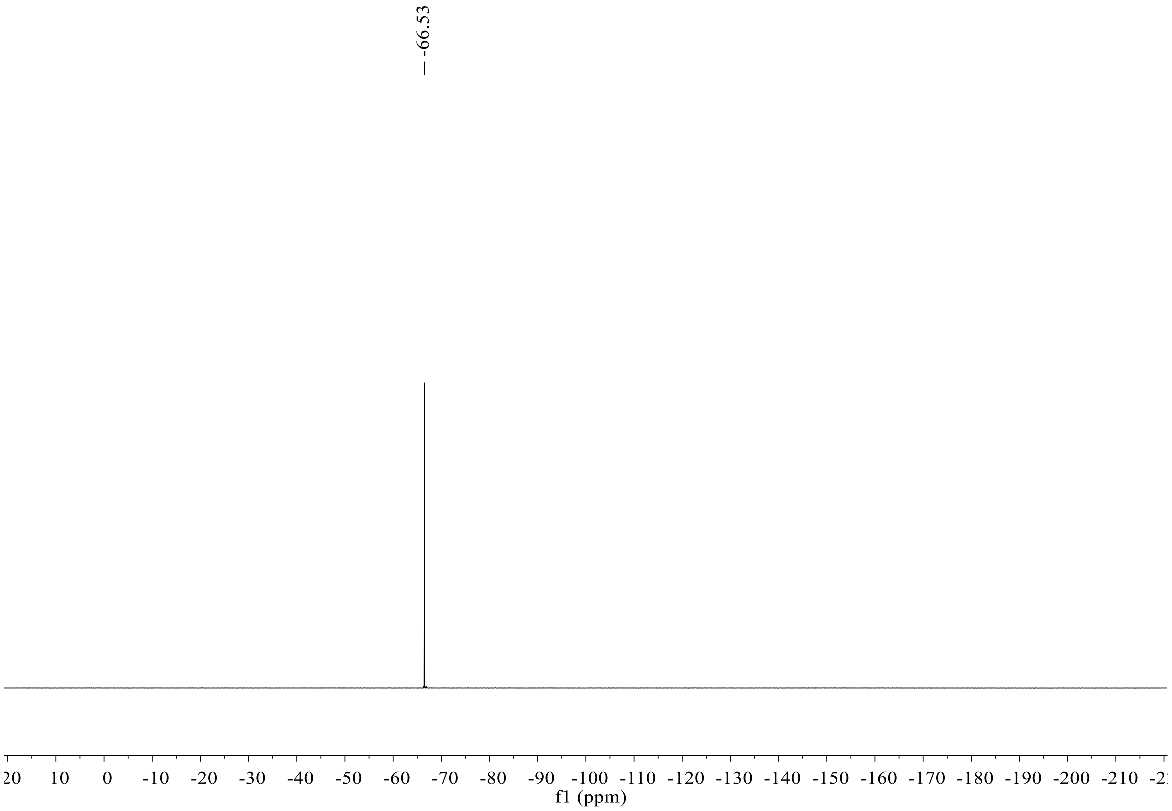

Supplementary Figure 18. ^{19}F NMR spectrum of **9**.

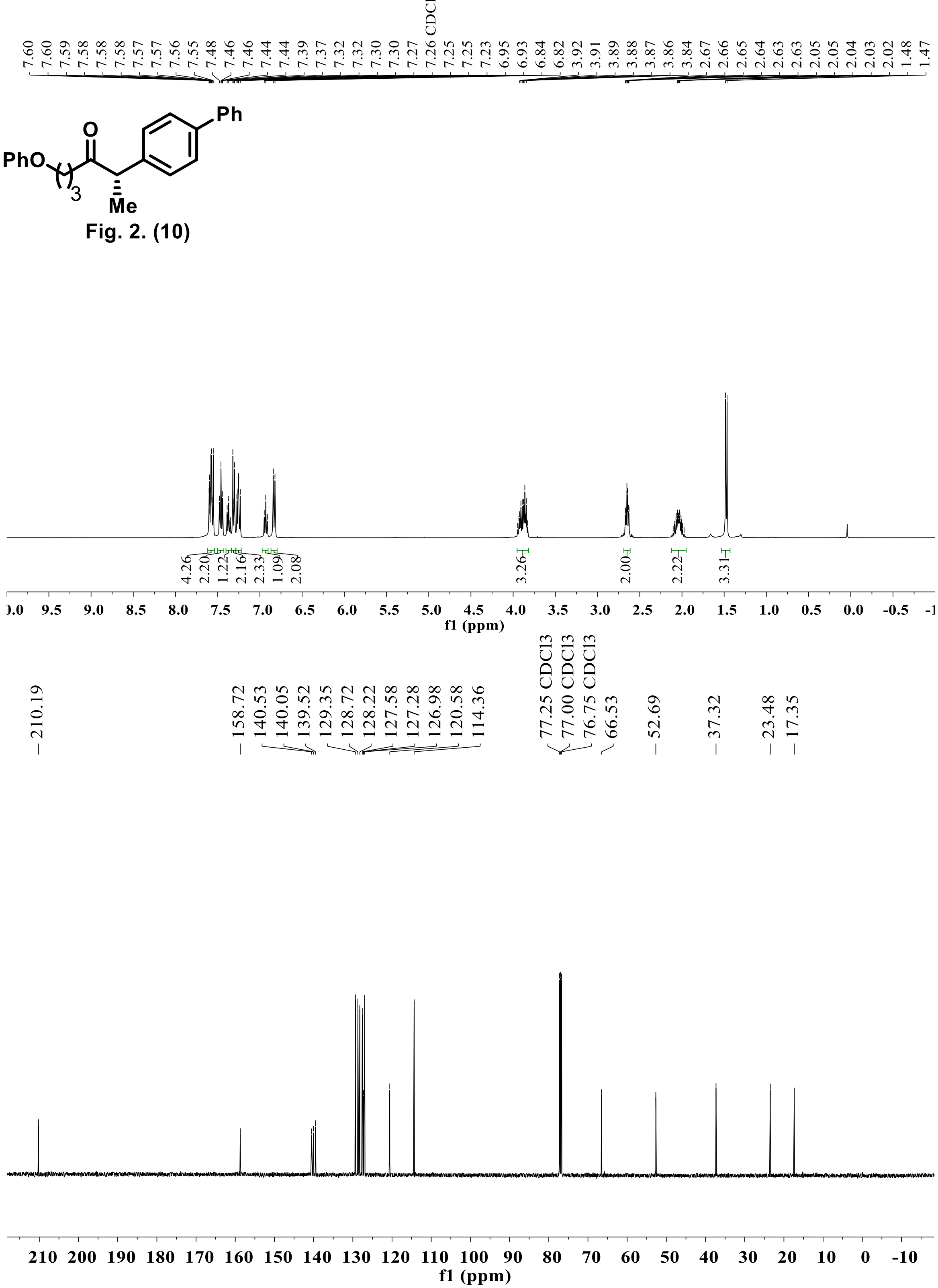

Supplementary Figure 19. ^{1}H NMR and ^{13}C NMR spectrum of **10**.

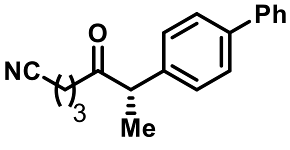

Fig. 2. (11)

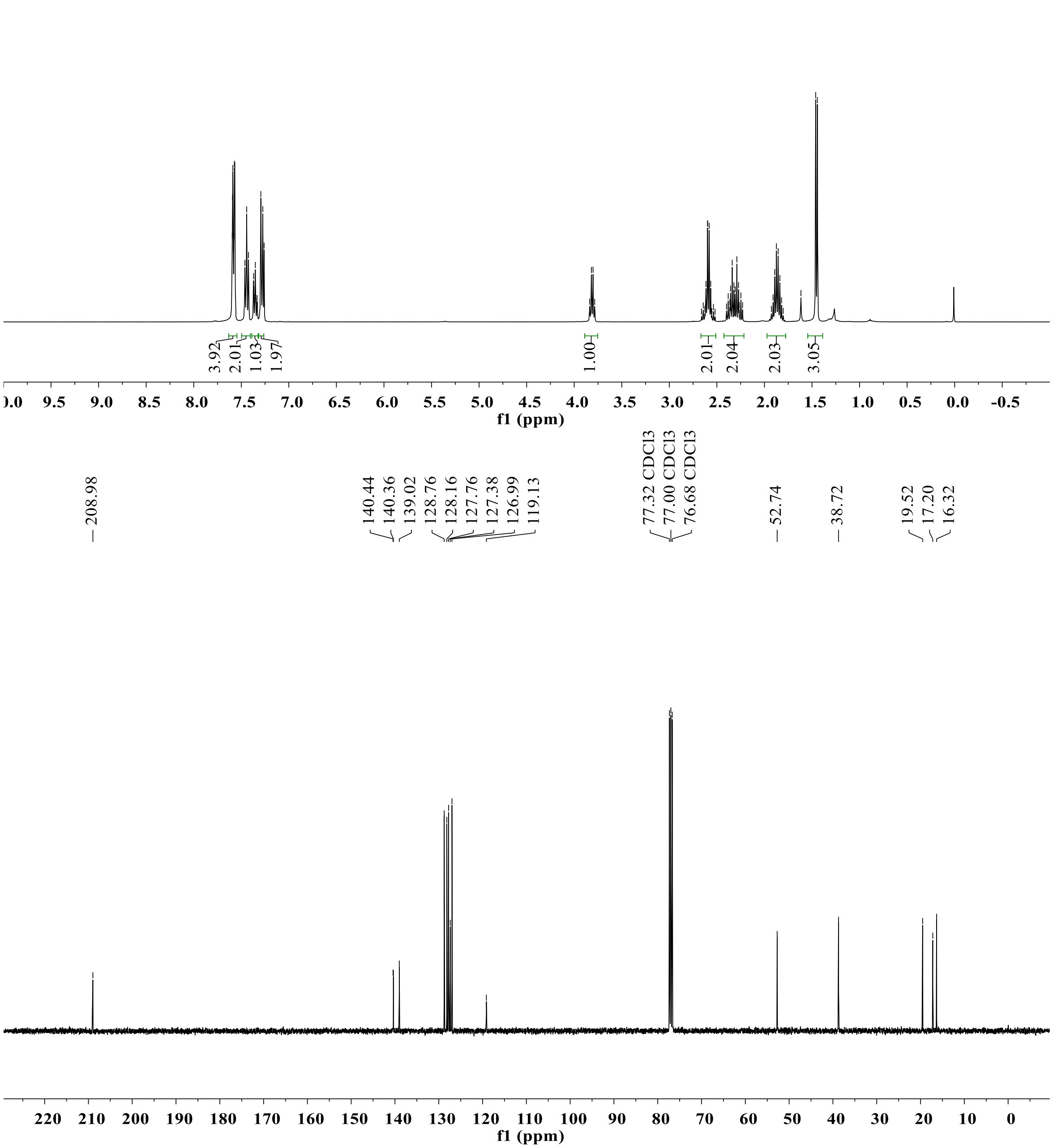

Supplementary Figure 20. ^{1}H NMR and ^{13}C NMR spectrum of **11**.

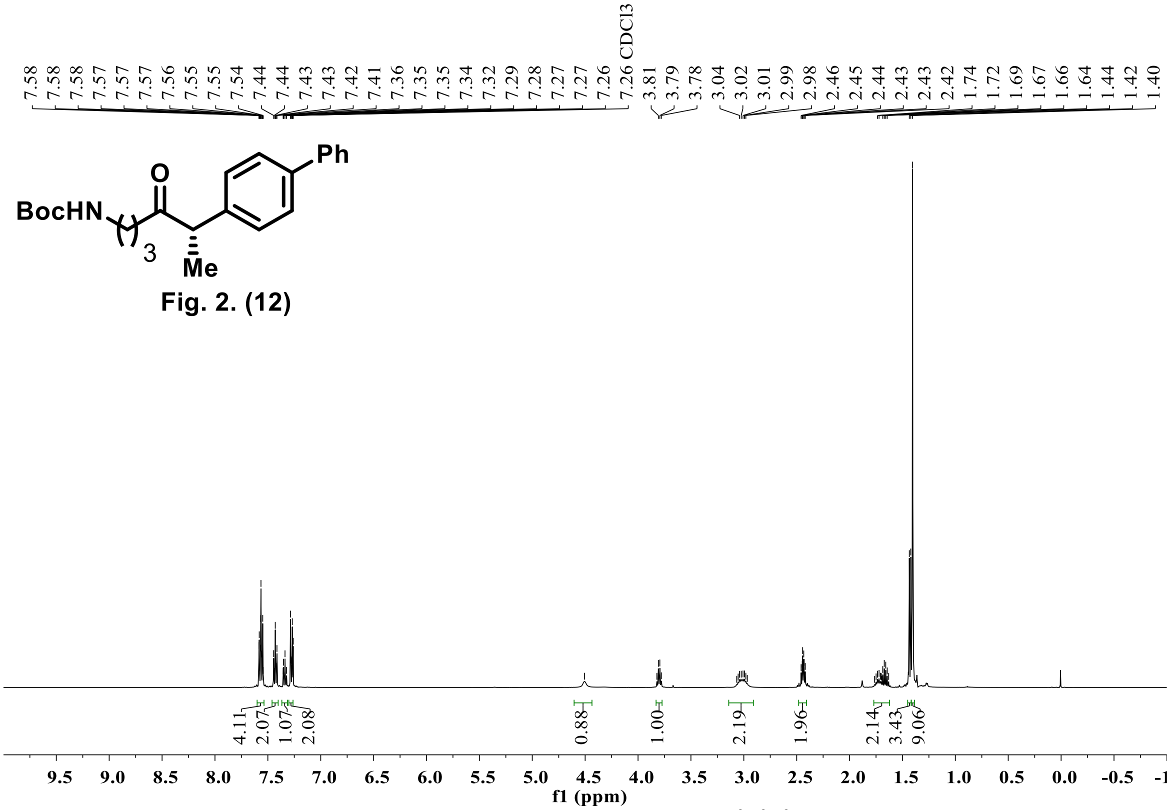

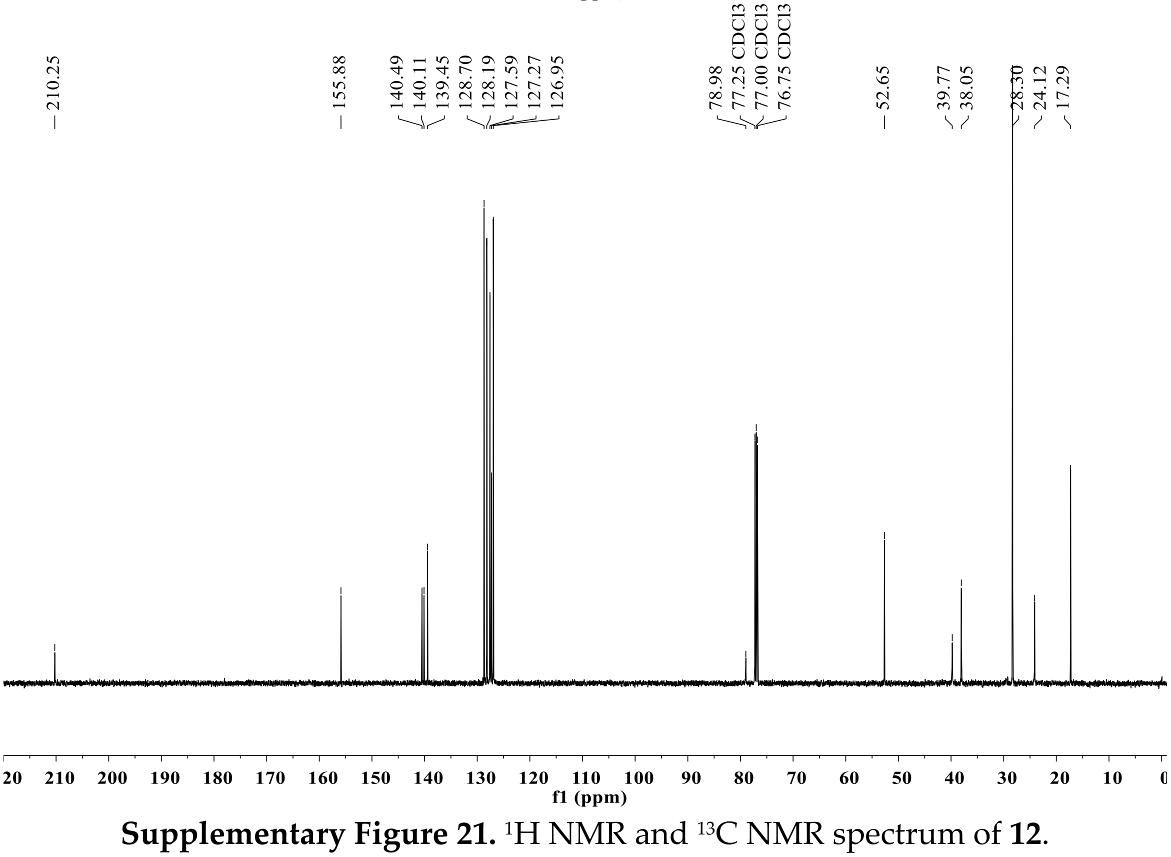

Supplementary Figure 21. ^{1}H NMR and ^{13}C NMR spectrum of **12**.

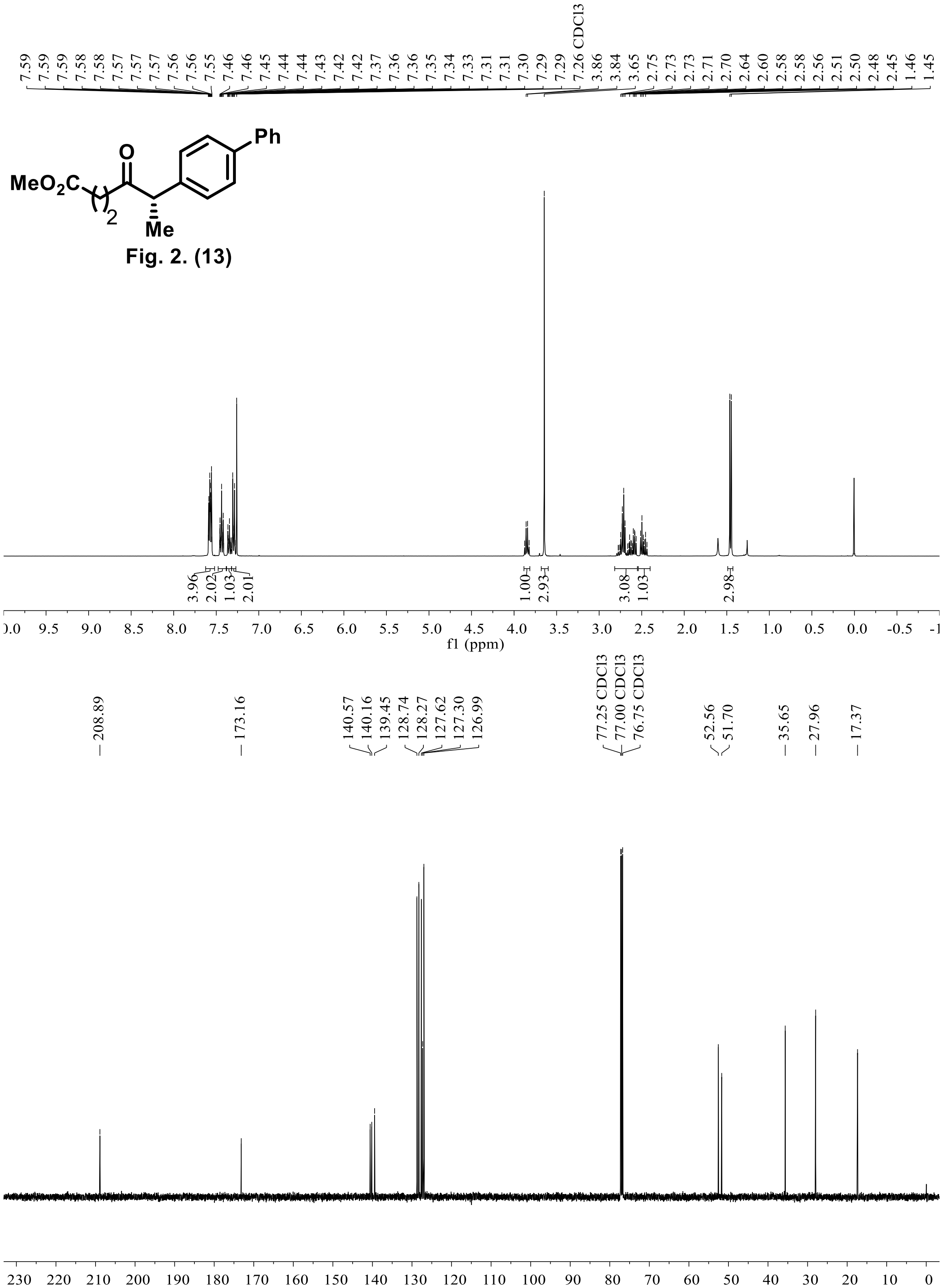

Supplementary Figure 22. ^{1}H NMR and ^{13}C NMR spectrum of **13**.

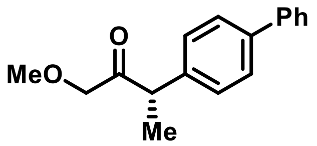

Fig. 2. (14)

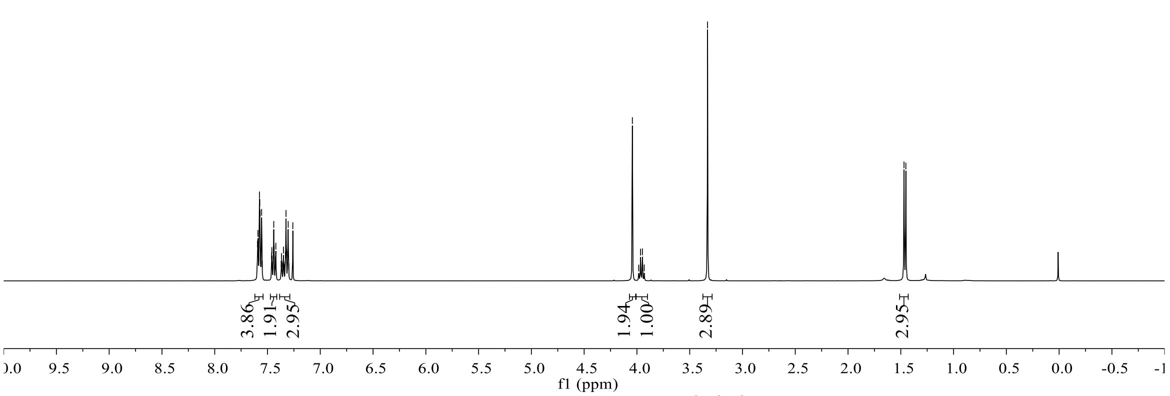

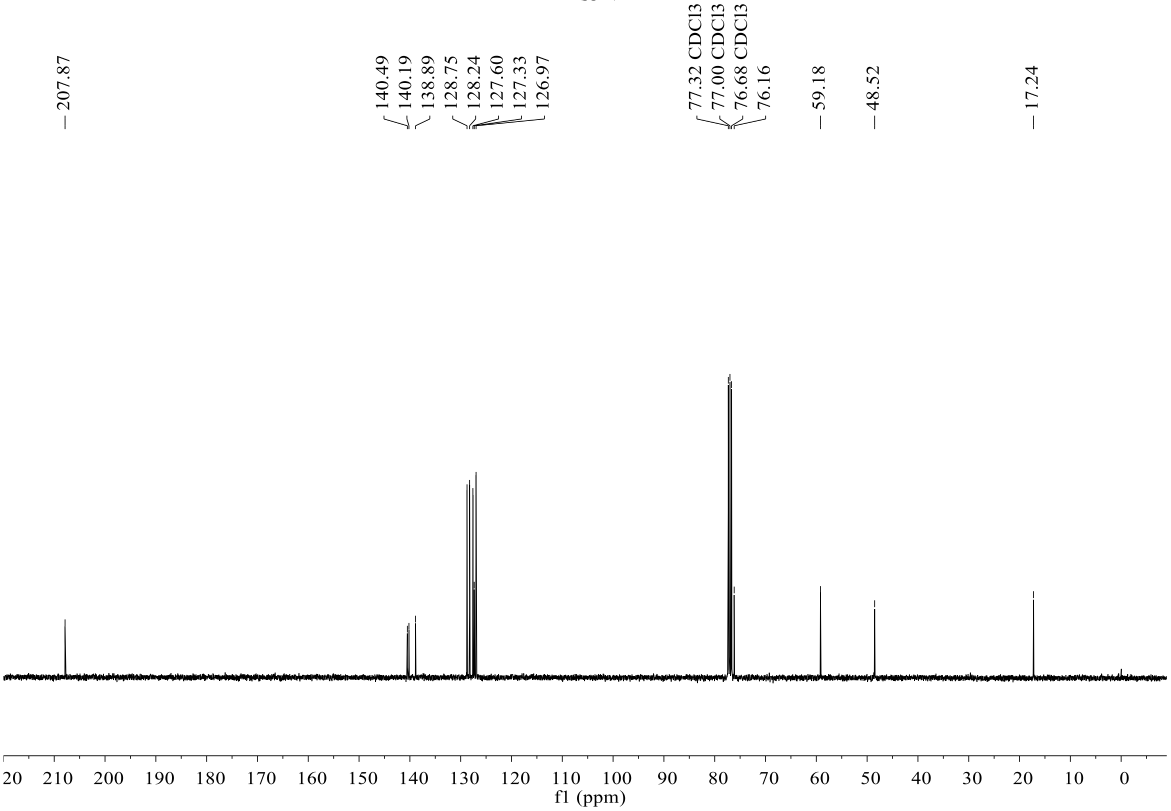

Supplementary Figure 23. ^{1}H NMR and ^{13}C NMR spectrum of **14**.

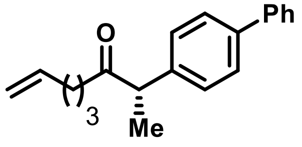

Fig. 2. (15)

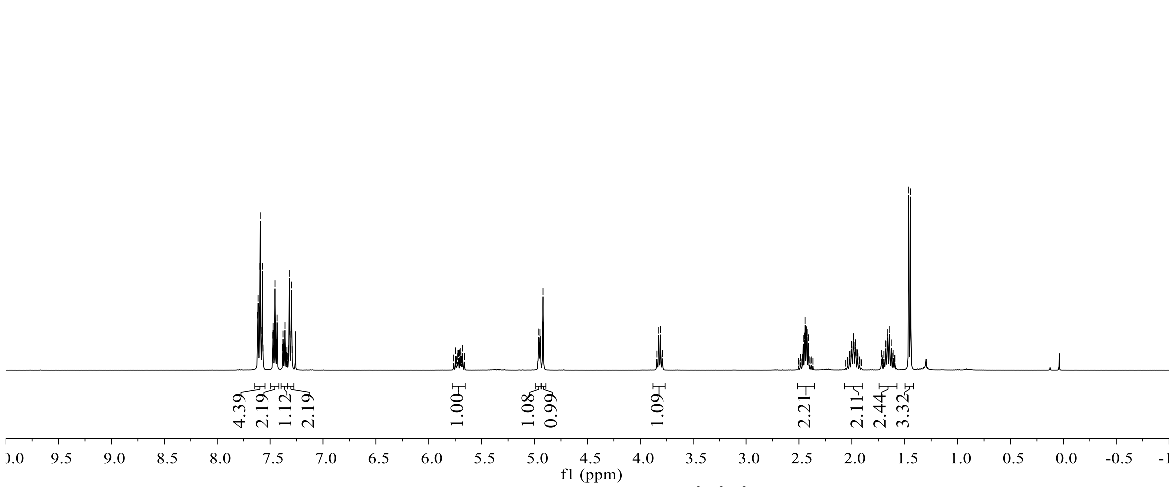

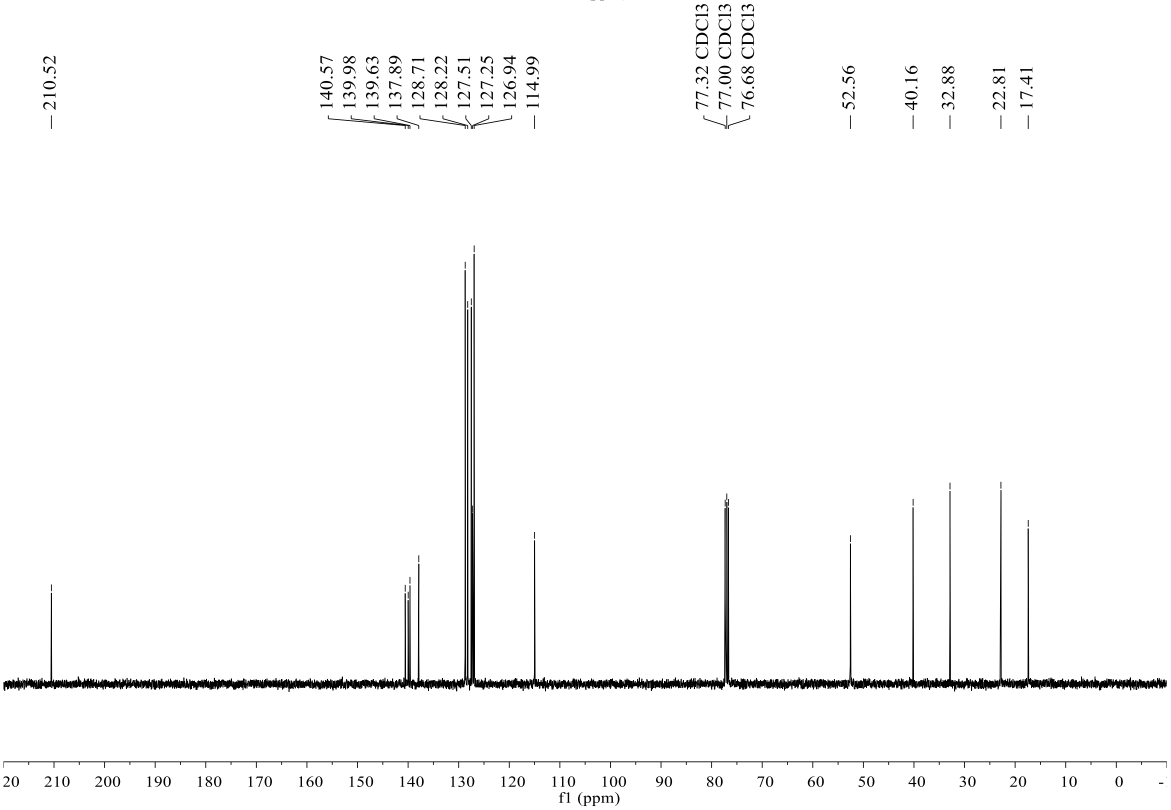

Supplementary Figure 24. ^{1}H NMR and ^{13}C NMR spectrum of **15**.

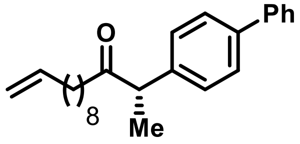

Fig. 2. (16)

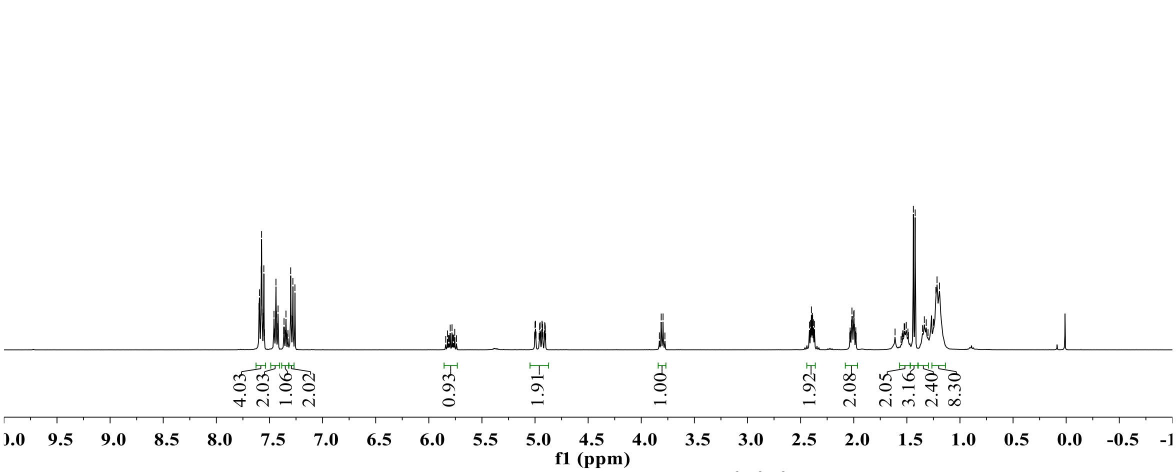

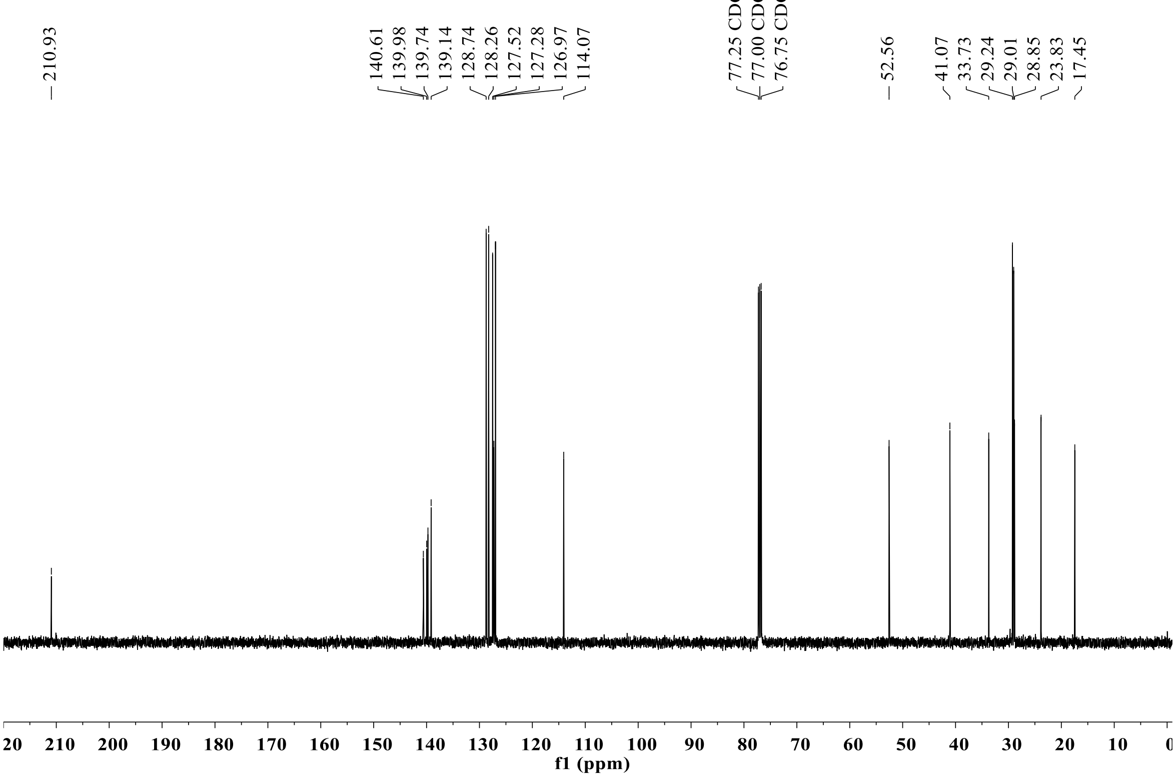

Supplementary Figure 25. ^{1}H NMR and ^{13}C NMR spectrum of **16**.

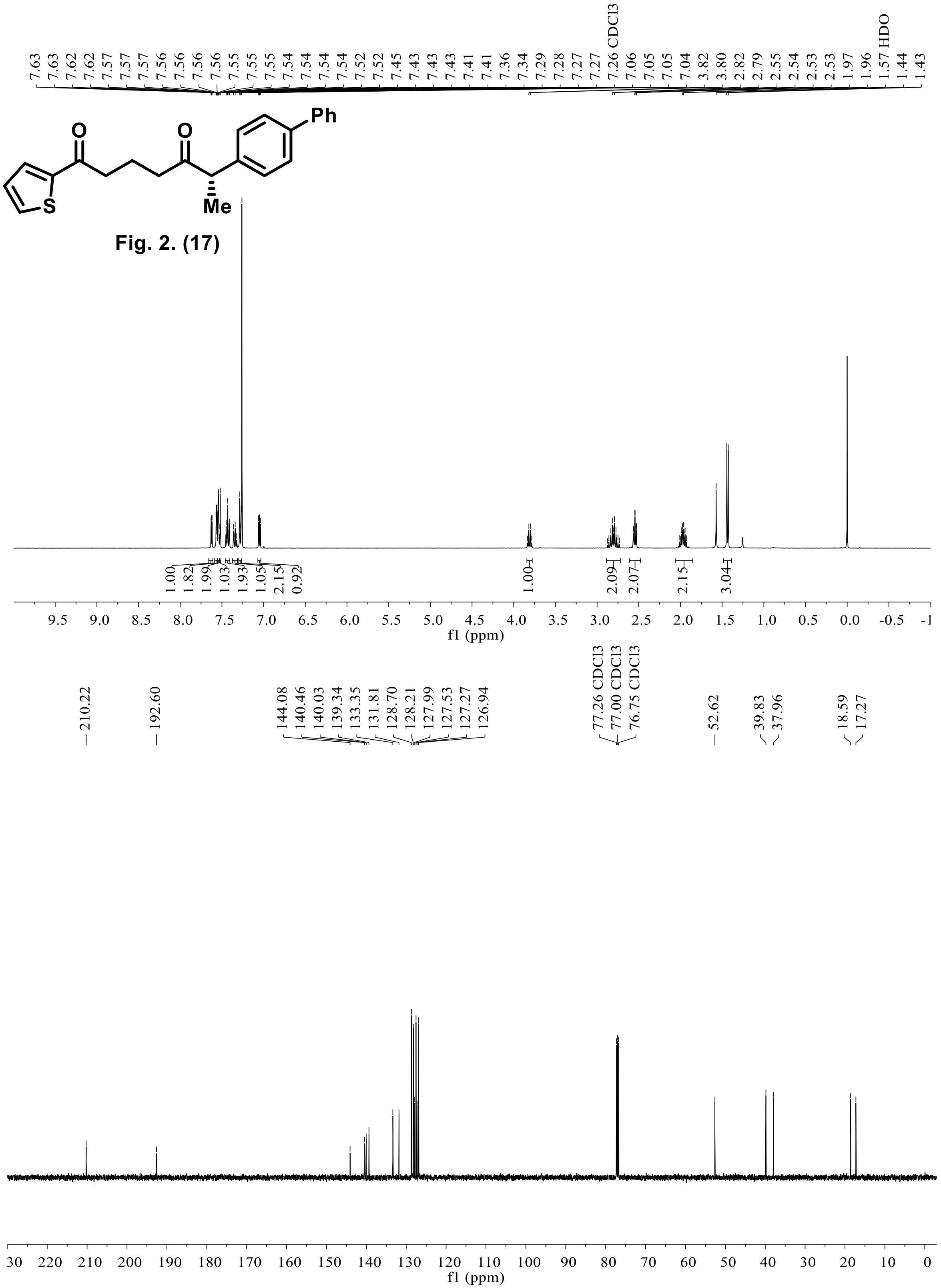

Supplementary Figure 26. ^{1}H NMR and ^{13}C NMR spectrum of **17**.

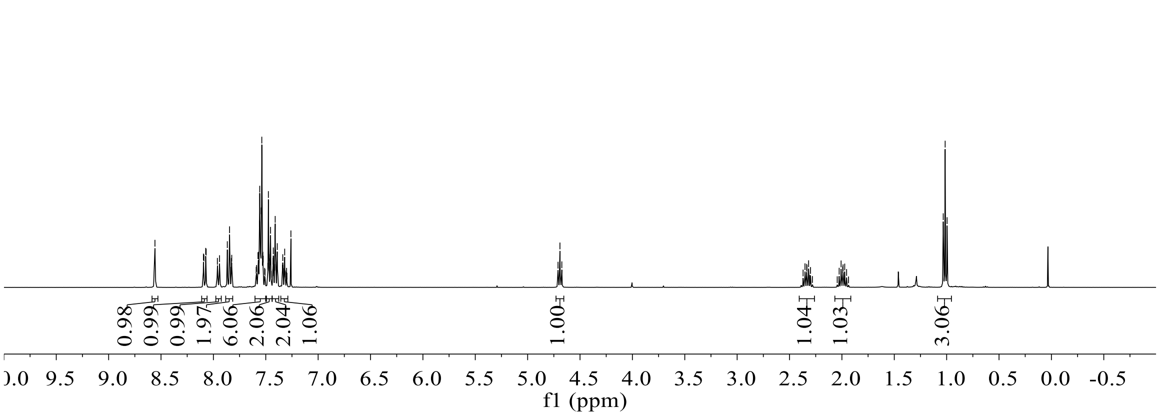

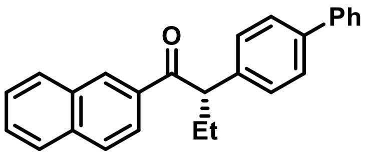

Fig. 2. (18)

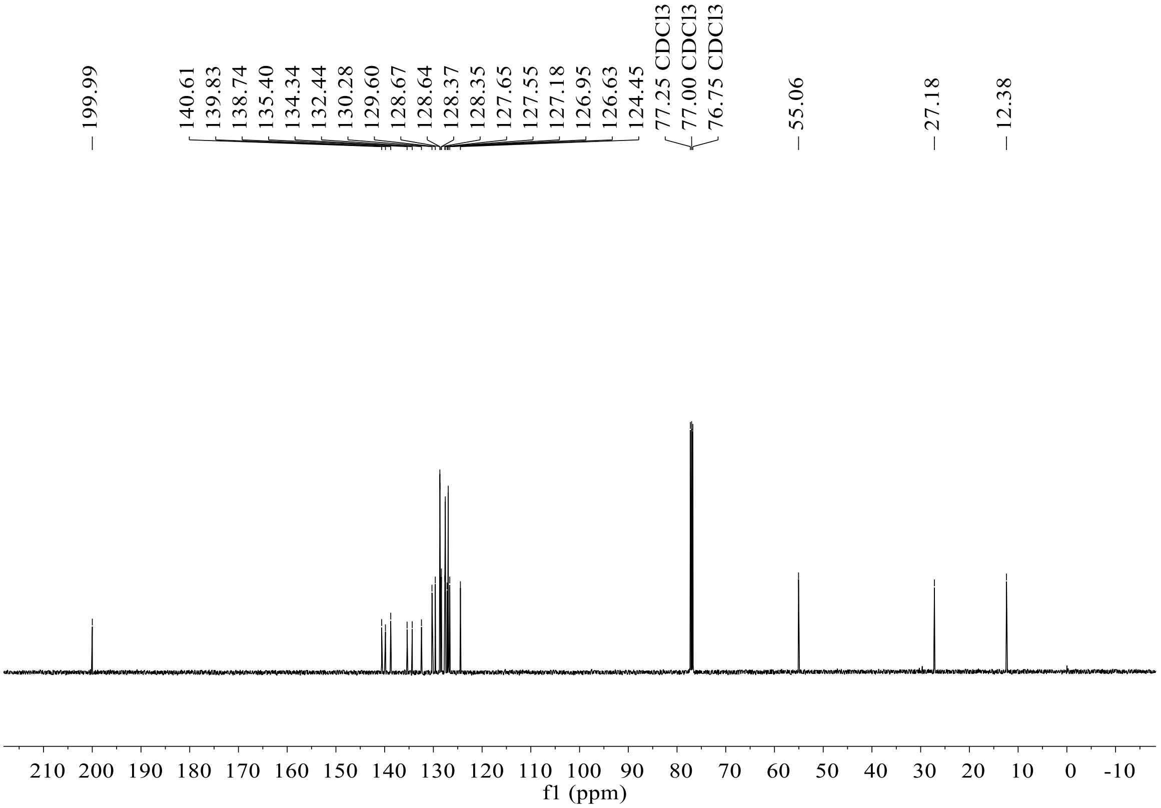

Supplementary Figure 27. ^{1}H NMR and ^{13}C NMR spectrum of **18**.

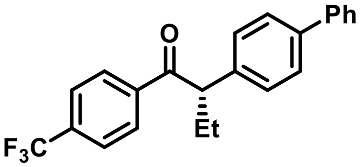

Fig. 2. (19)

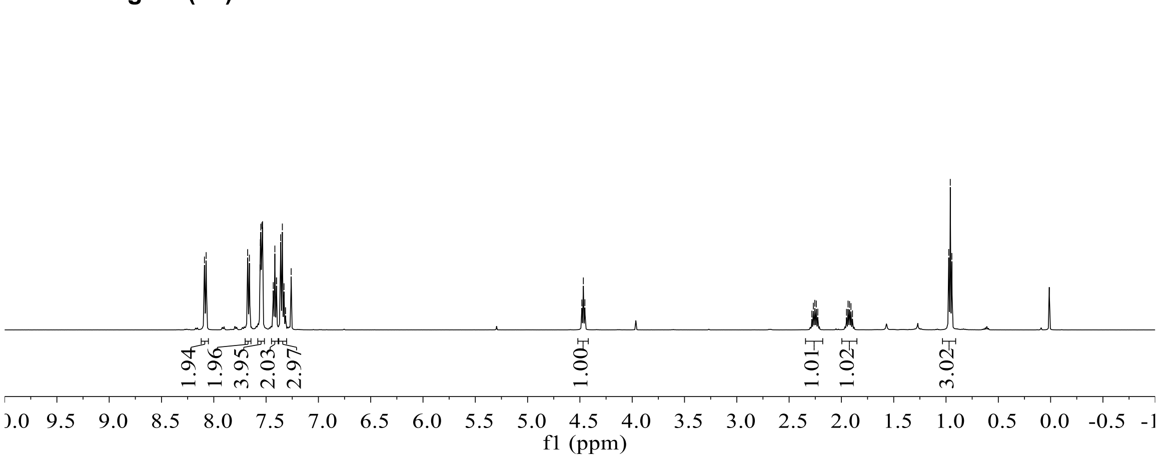

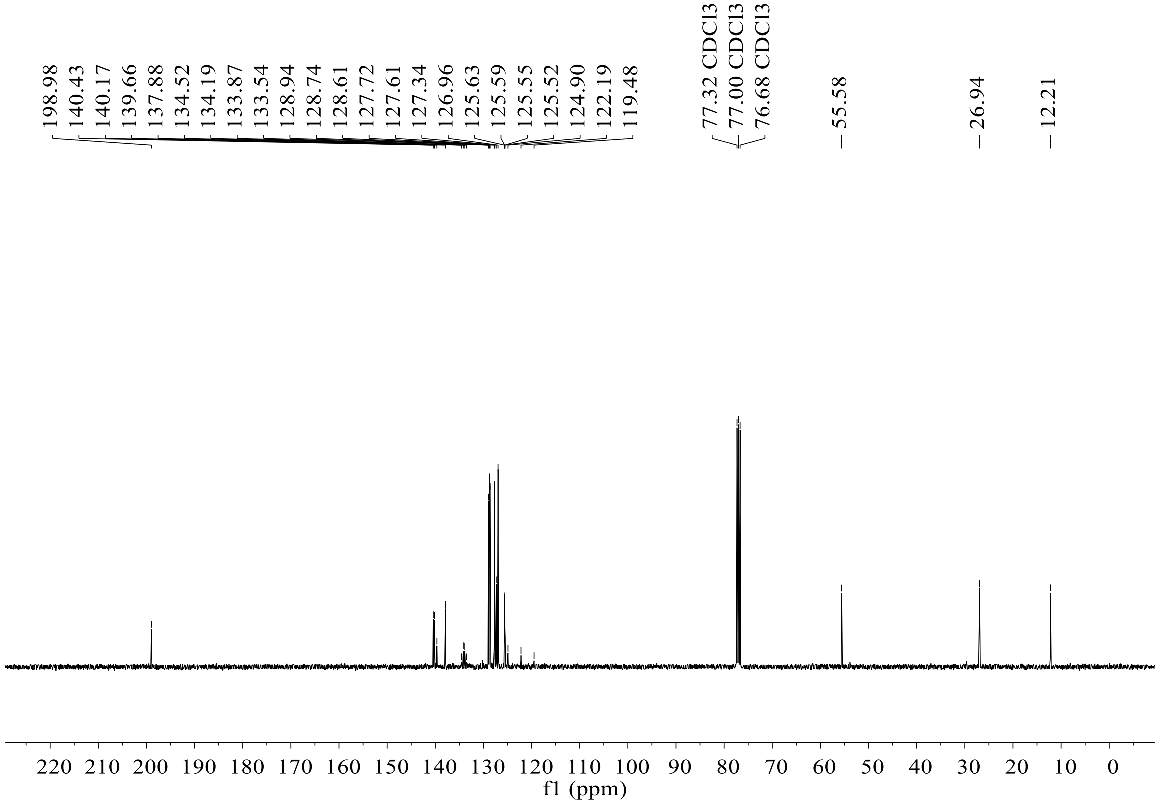

Supplementary Figure 28. ^{1}H NMR and ^{13}C NMR spectrum of **19**.

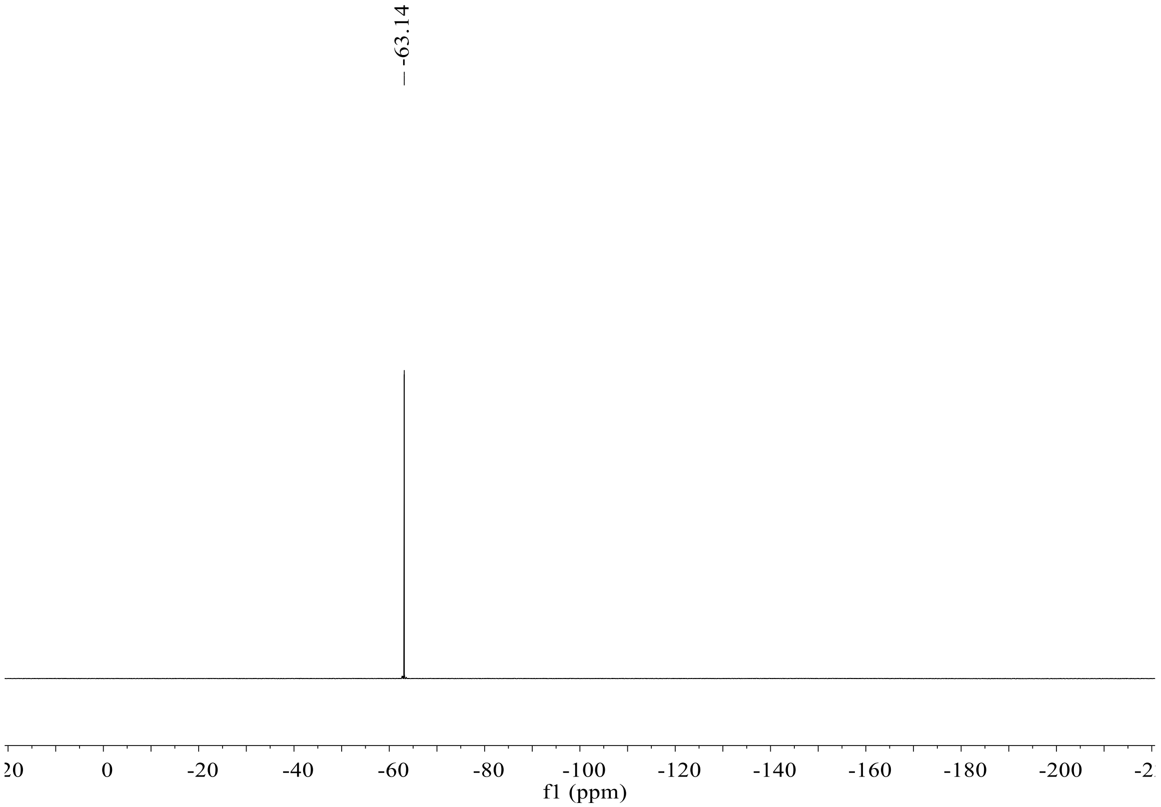

Supplementary Figure 29. ^{19}F NMR spectrum of **19**.

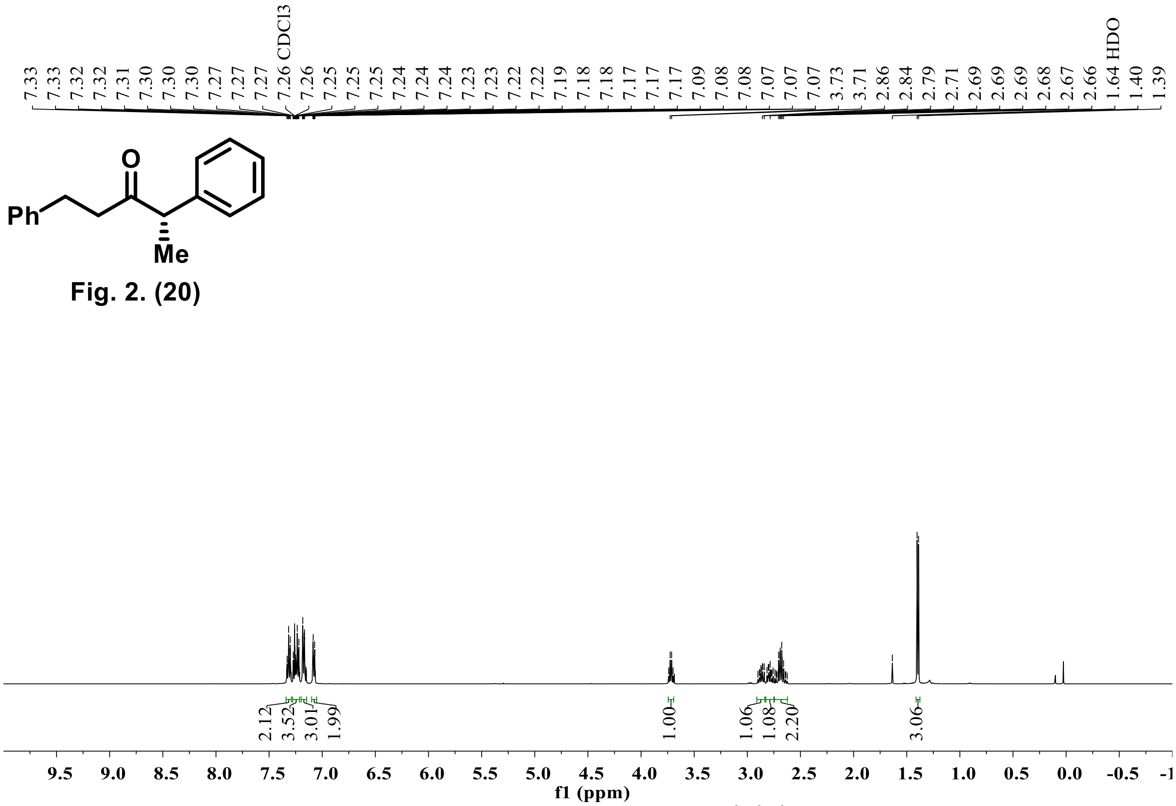

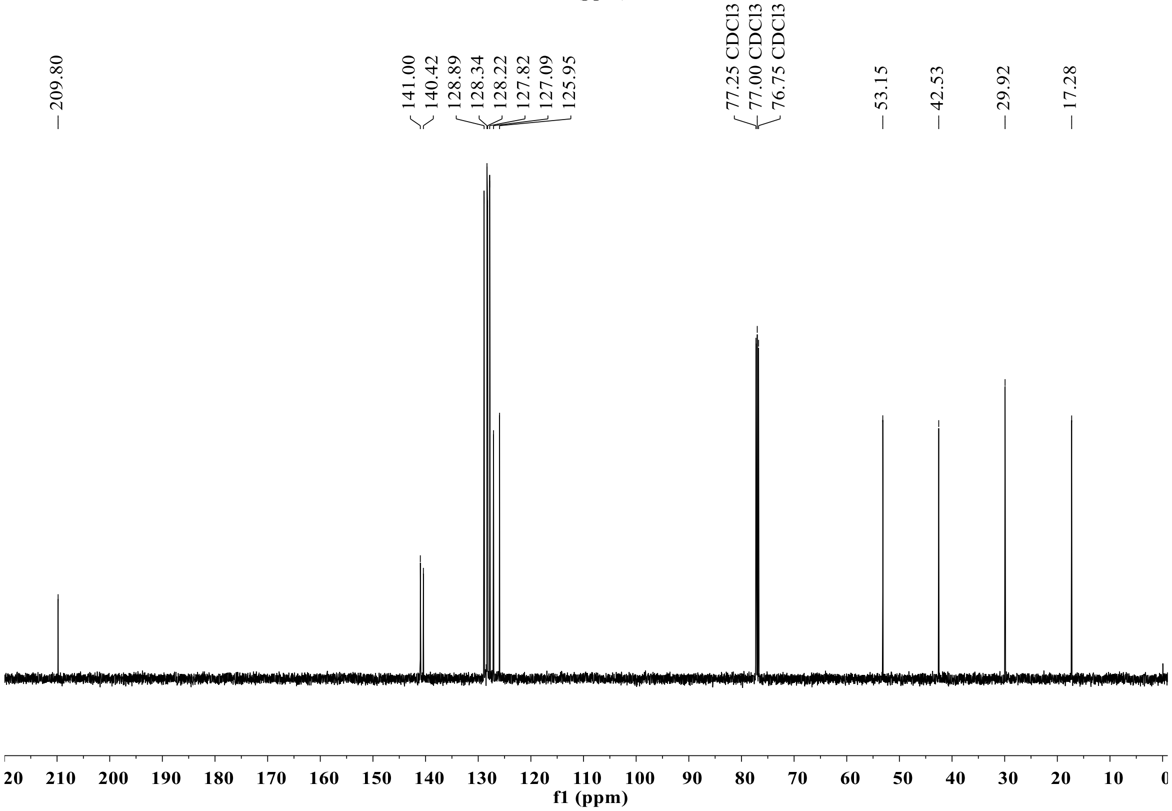

Supplementary Figure 30. ^{1}H NMR and ^{13}C NMR spectrum of **20**.

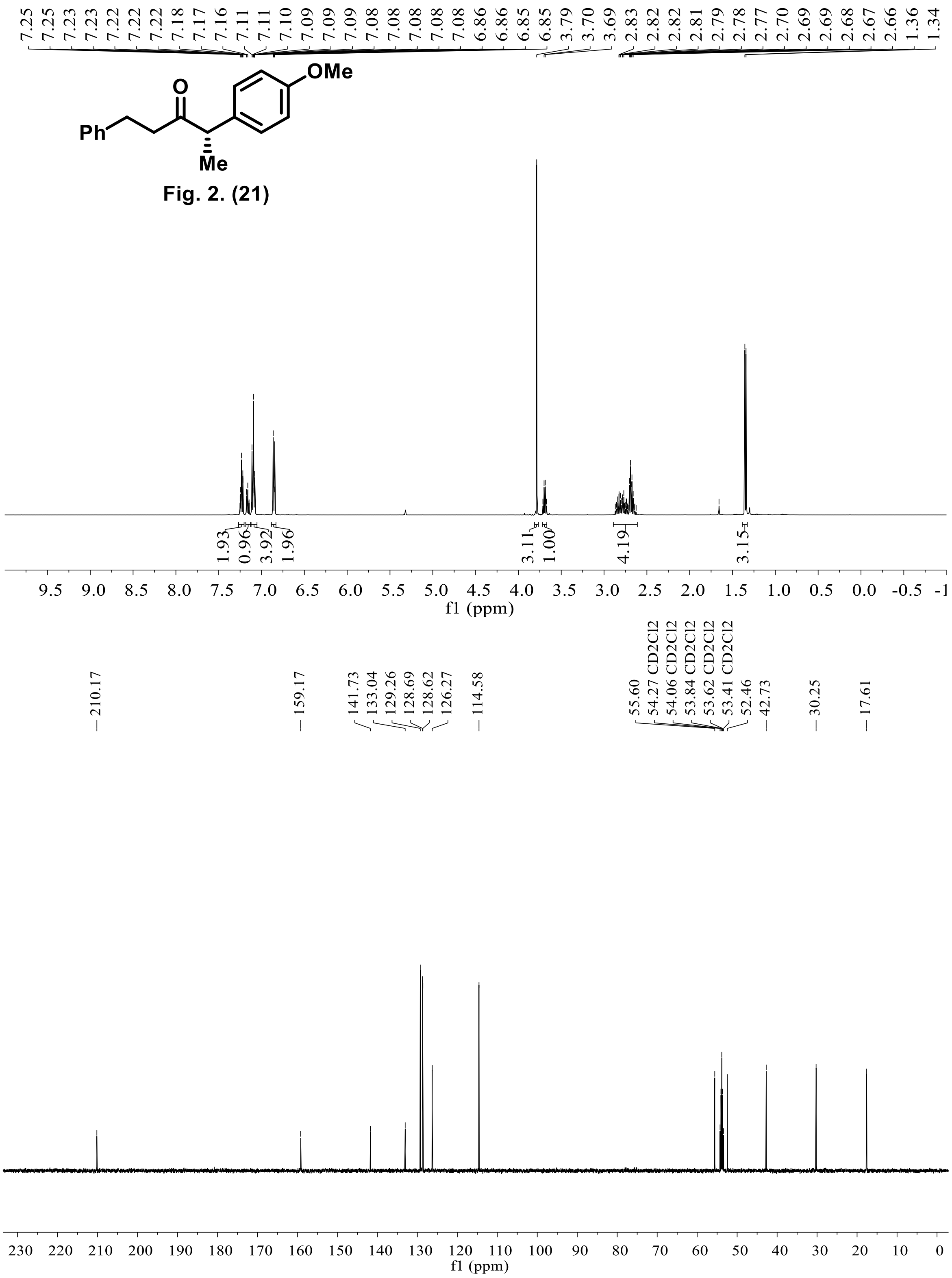

Supplementary Figure 31. ^{1}H NMR and ^{13}C NMR spectrum of **21**.

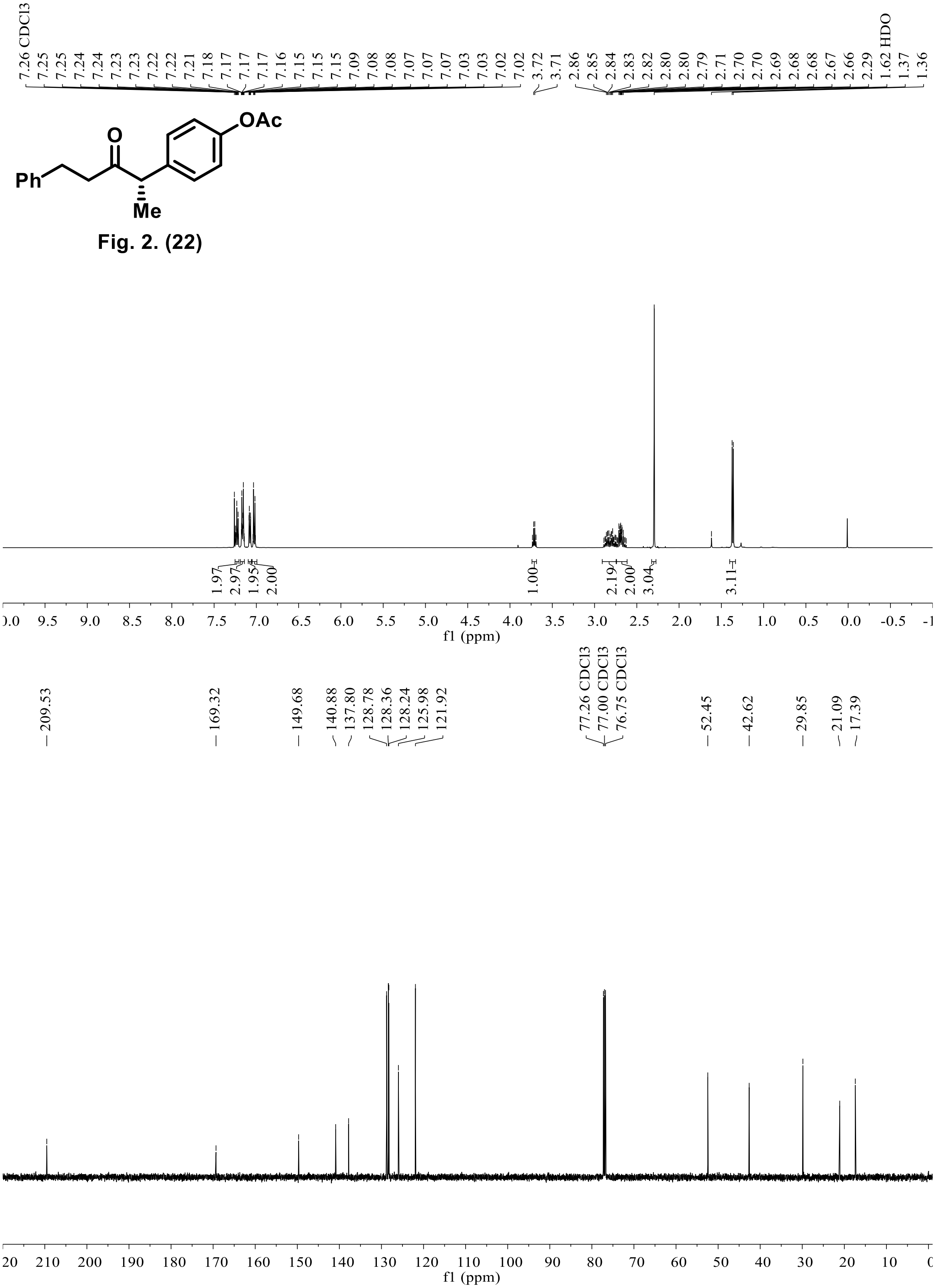

Supplementary Figure 32. ^{1}H NMR and ^{13}C NMR spectrum of **22**.

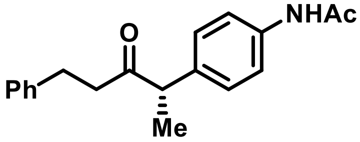

Fig. 2. (23)

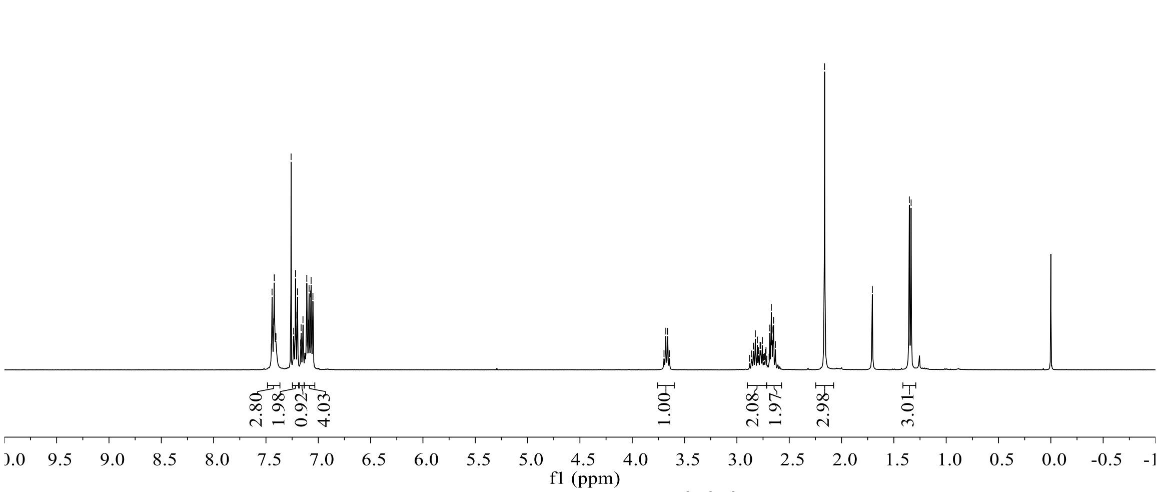

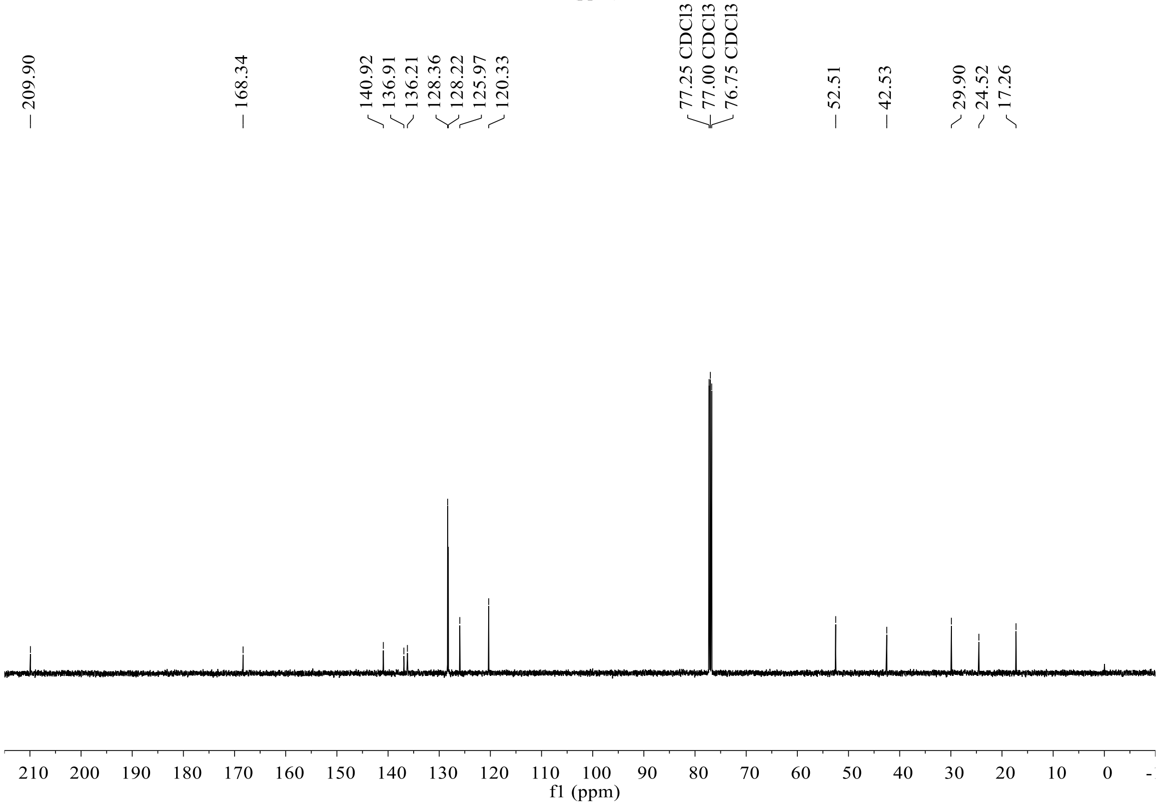

Supplementary Figure 33. ^{1}H NMR and ^{13}C NMR spectrum of **23**.

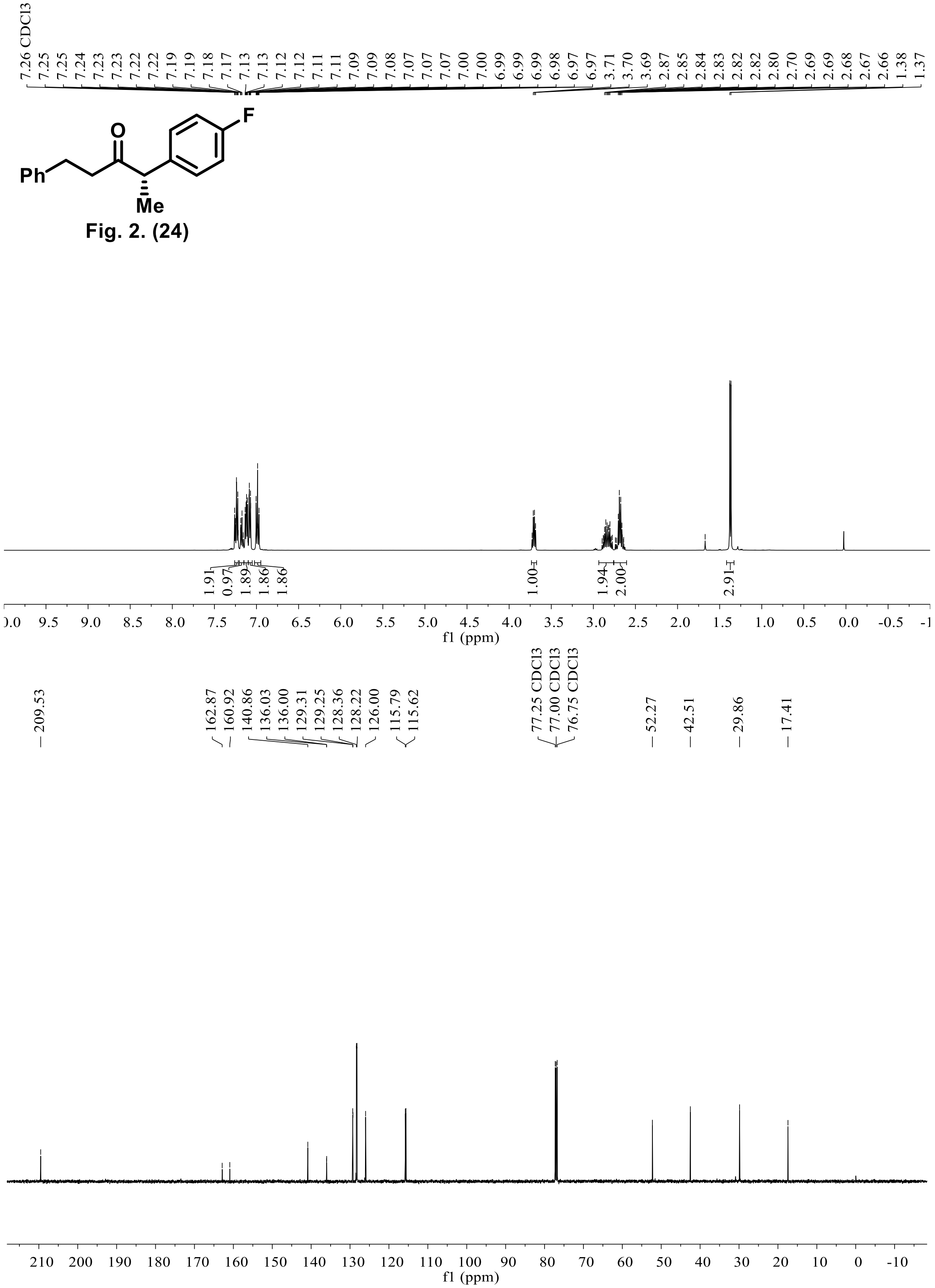

Supplementary Figure 34. ^{1}H NMR and ^{13}C NMR spectrum of **24**.

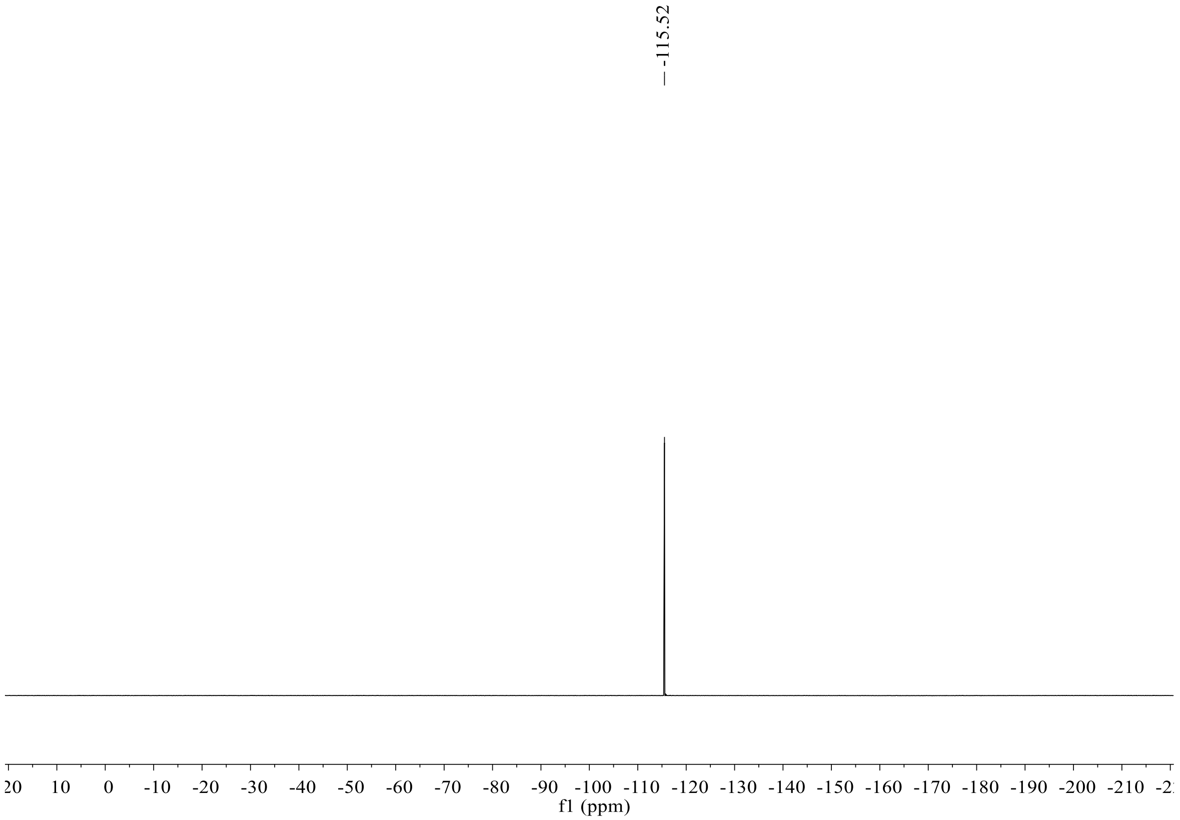

Supplementary Figure 35. ^{19}F NMR spectrum of **24**.

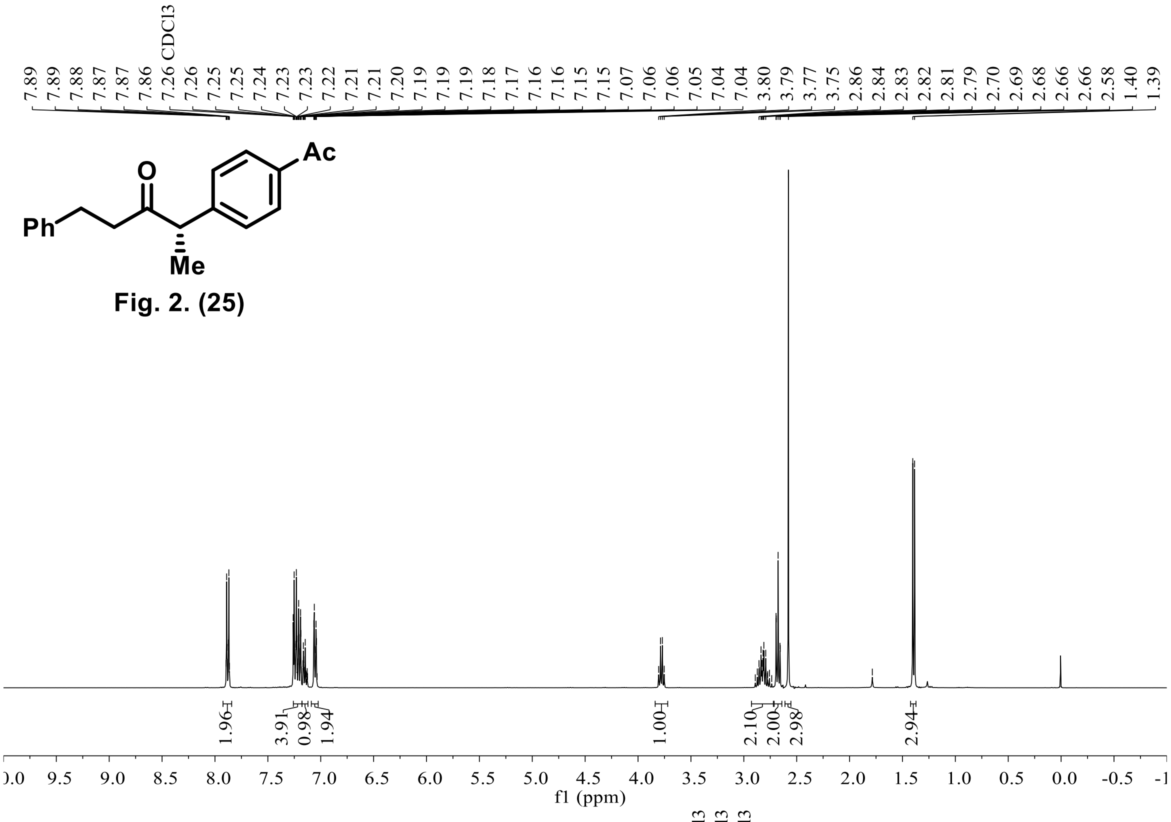

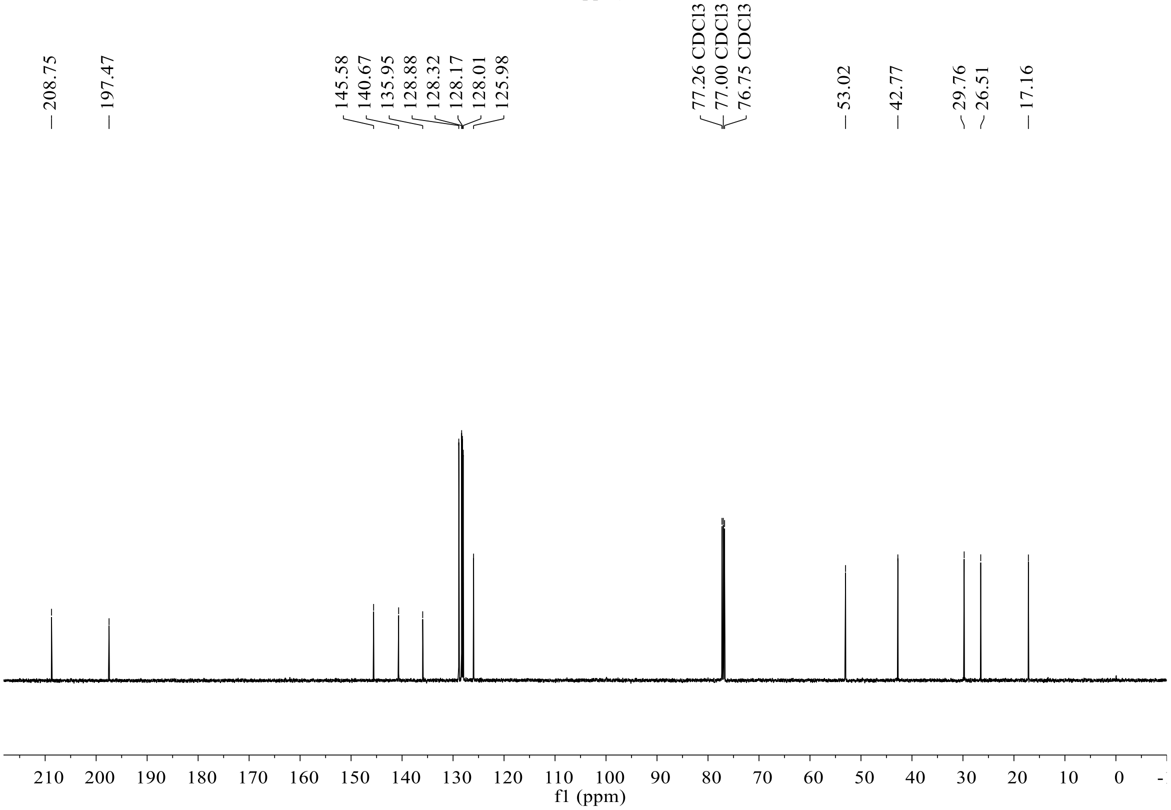

Supplementary Figure 36. ^{1}H NMR and ^{13}C NMR spectrum of **25**.

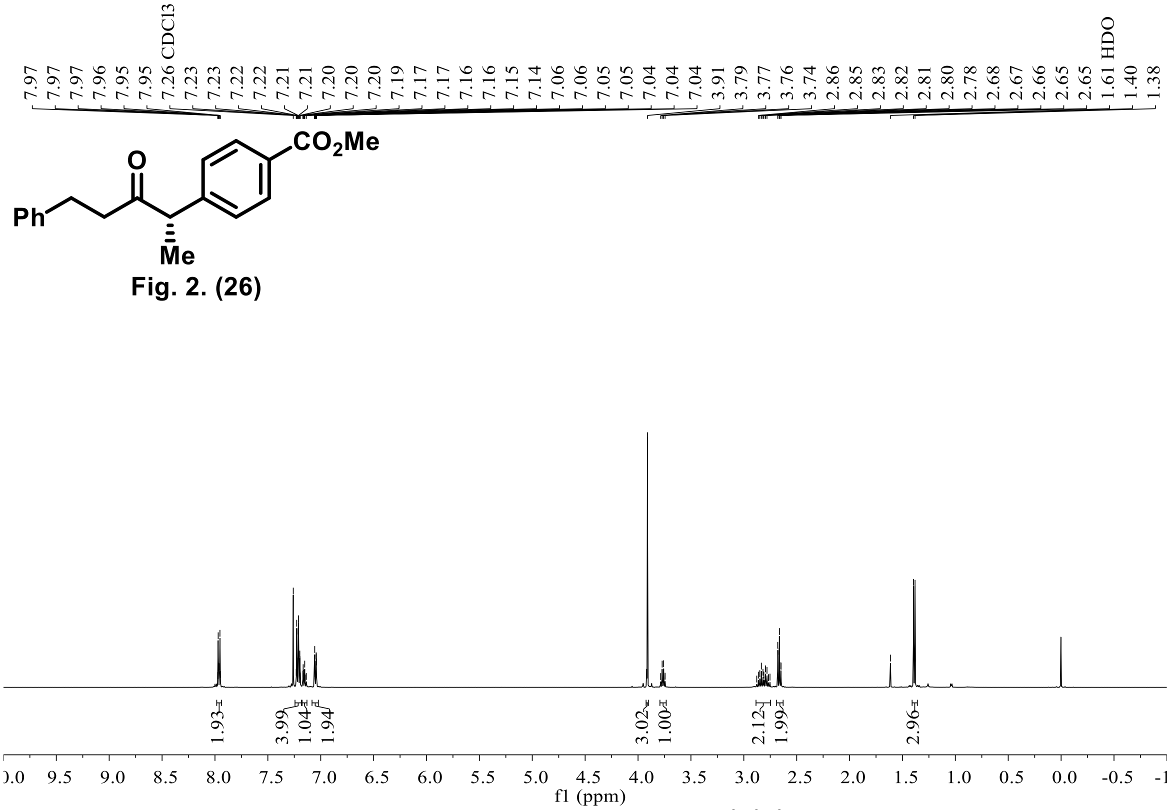

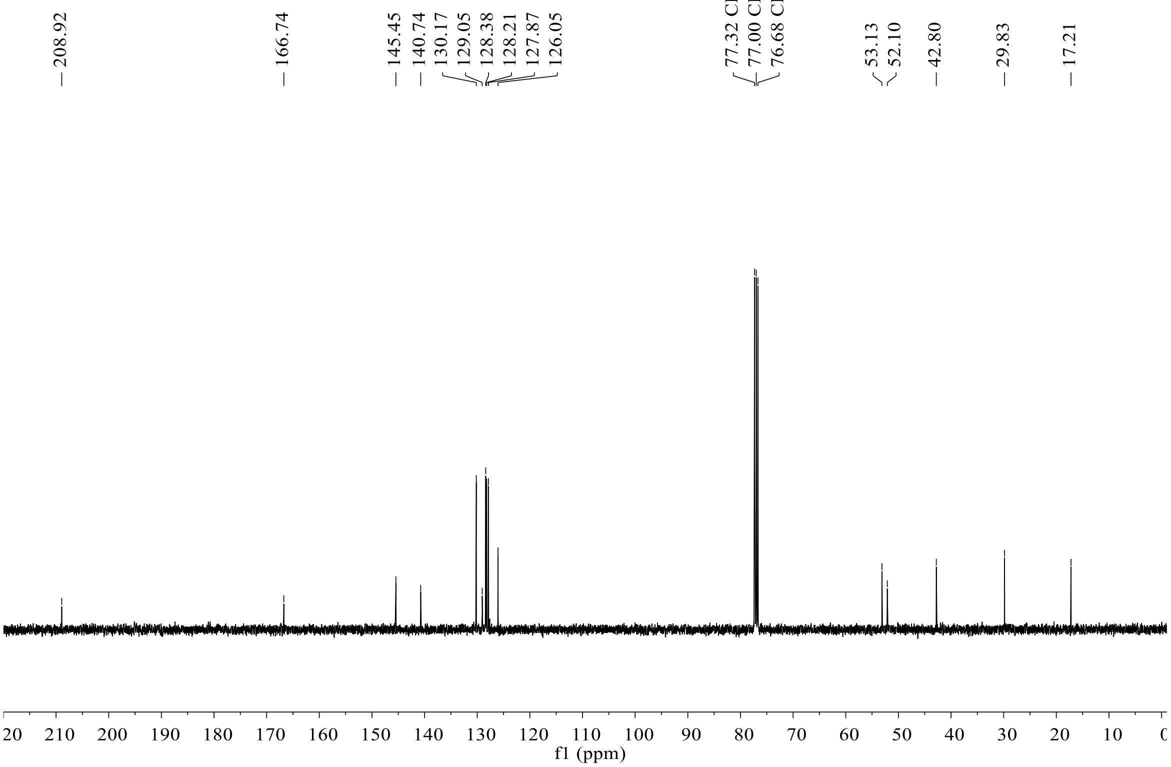

Supplementary Figure 37. ^{1}H NMR and ^{13}C NMR spectrum of **26**.

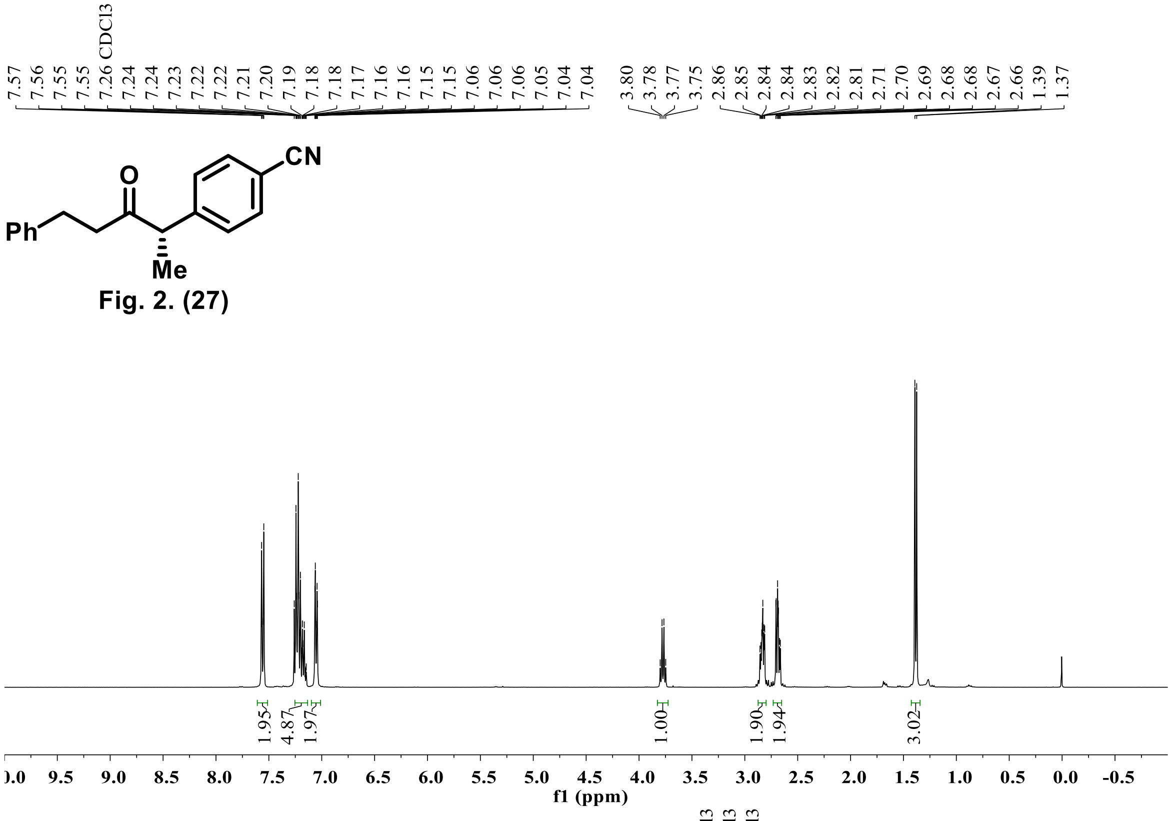

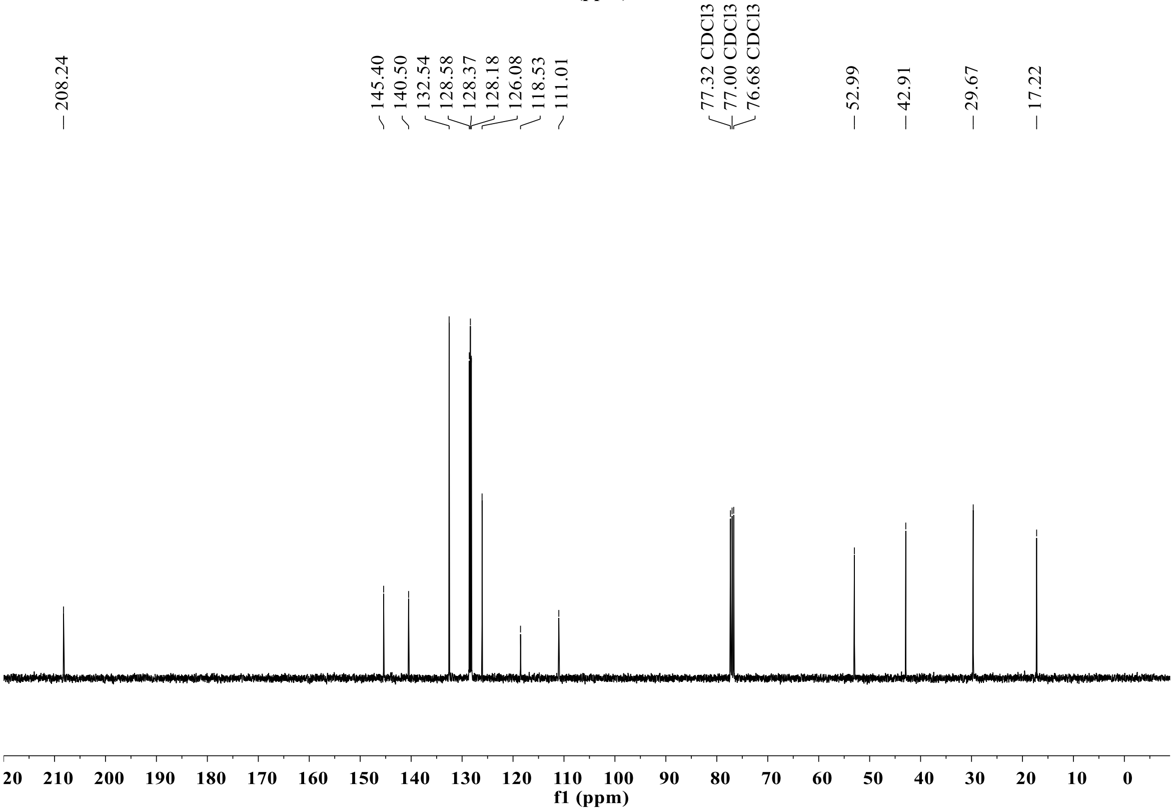

Supplementary Figure 38. ^{1}H NMR and ^{13}C NMR spectrum of **27**.

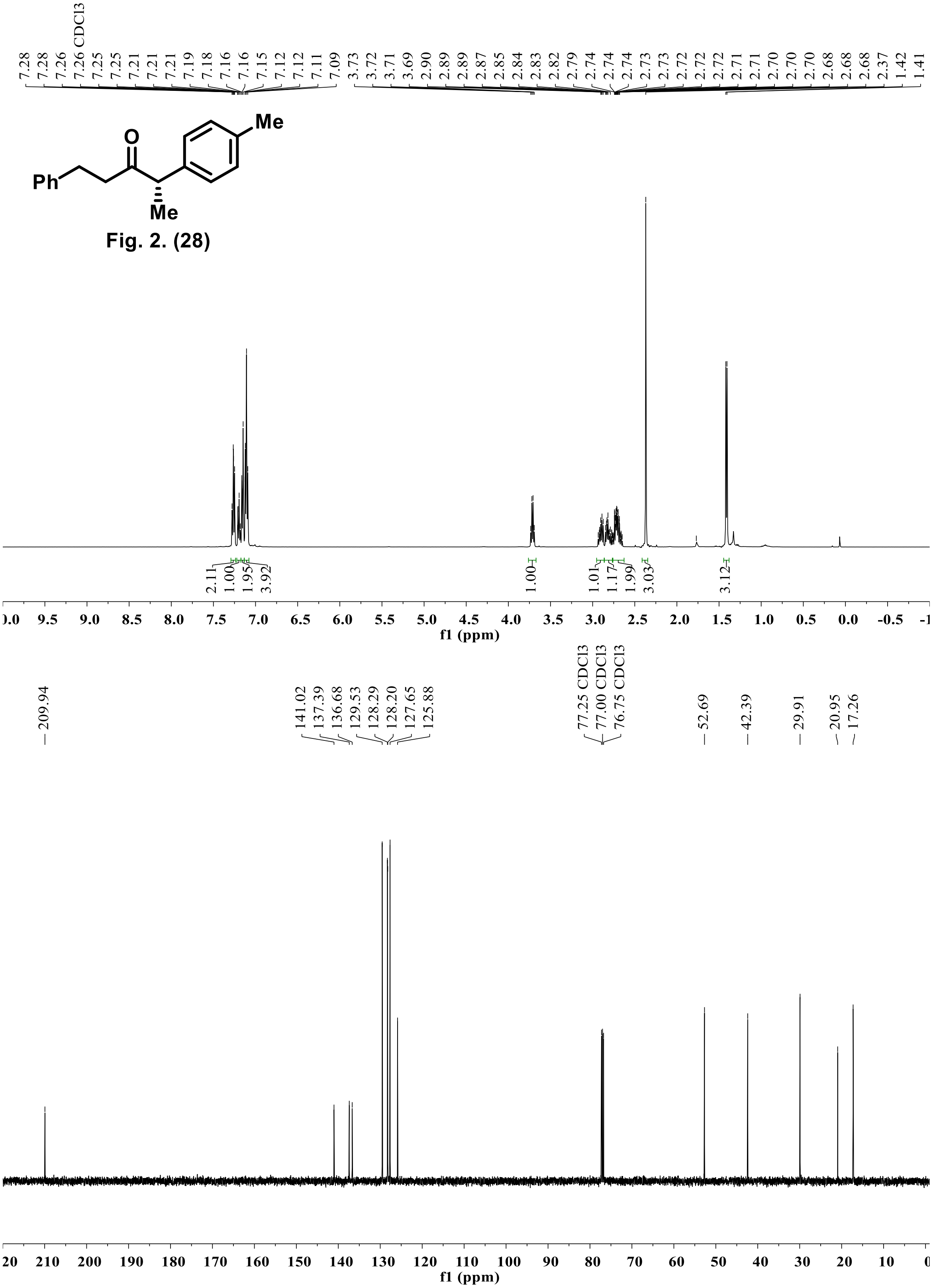

Supplementary Figure 39. ^{1}H NMR and ^{13}C NMR spectrum of **28**.

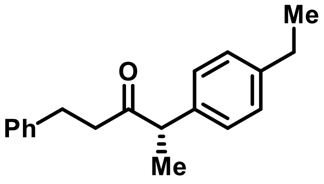

Fig. 2. (29)

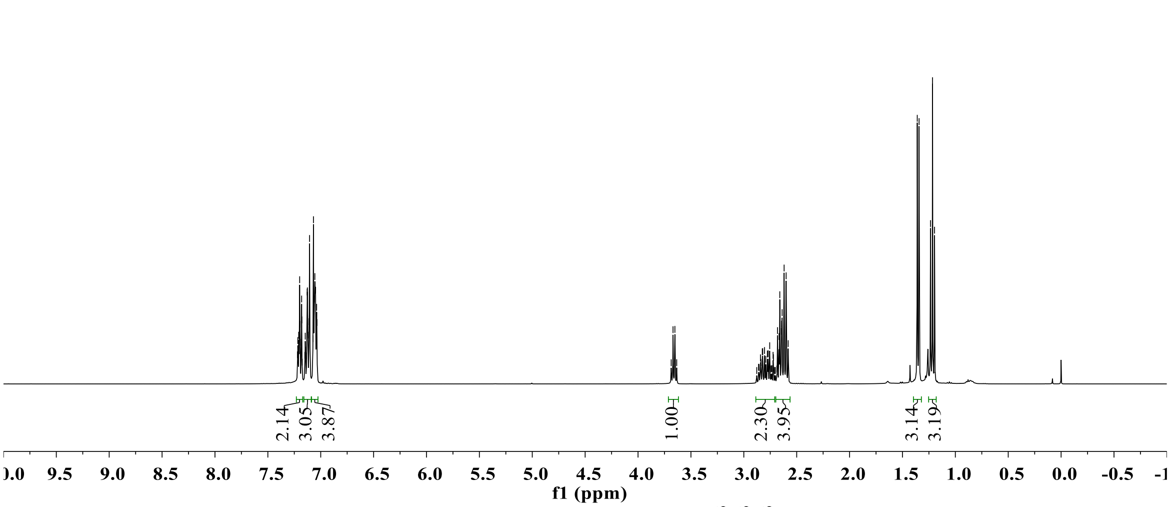

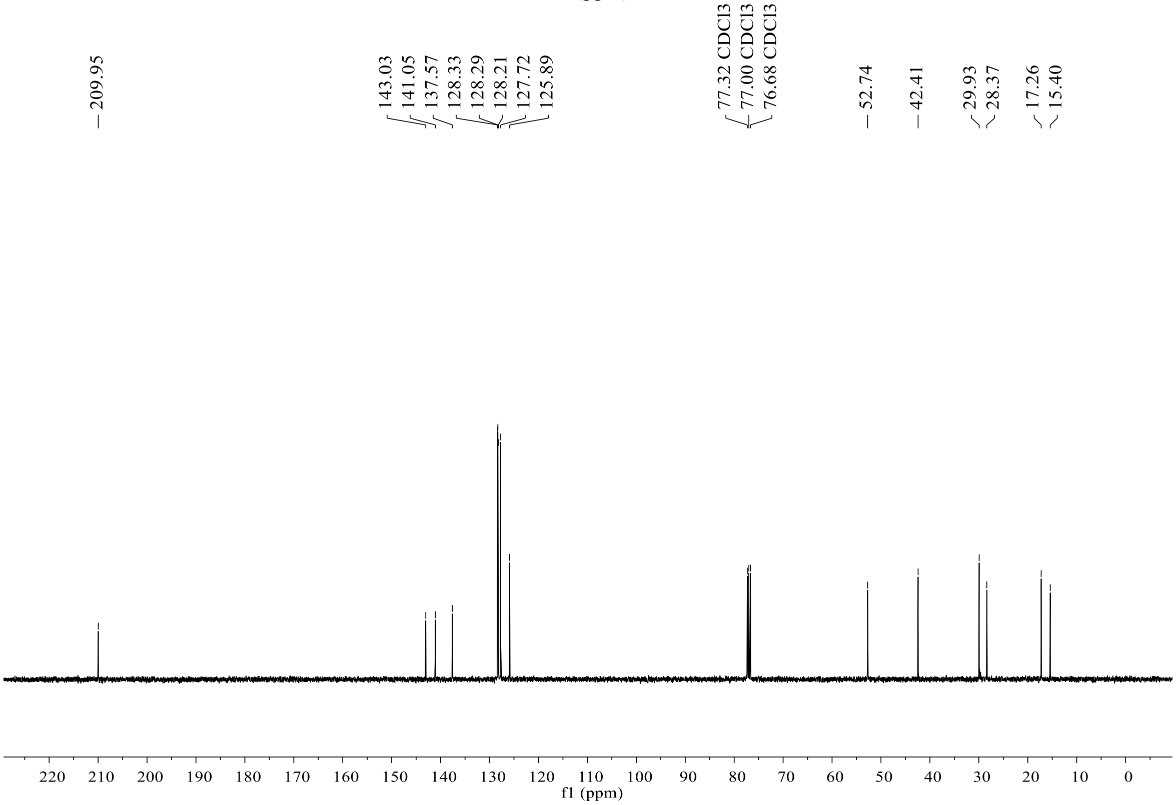

Supplementary Figure 40. ^{1}H NMR and ^{13}C NMR spectrum of **29**.

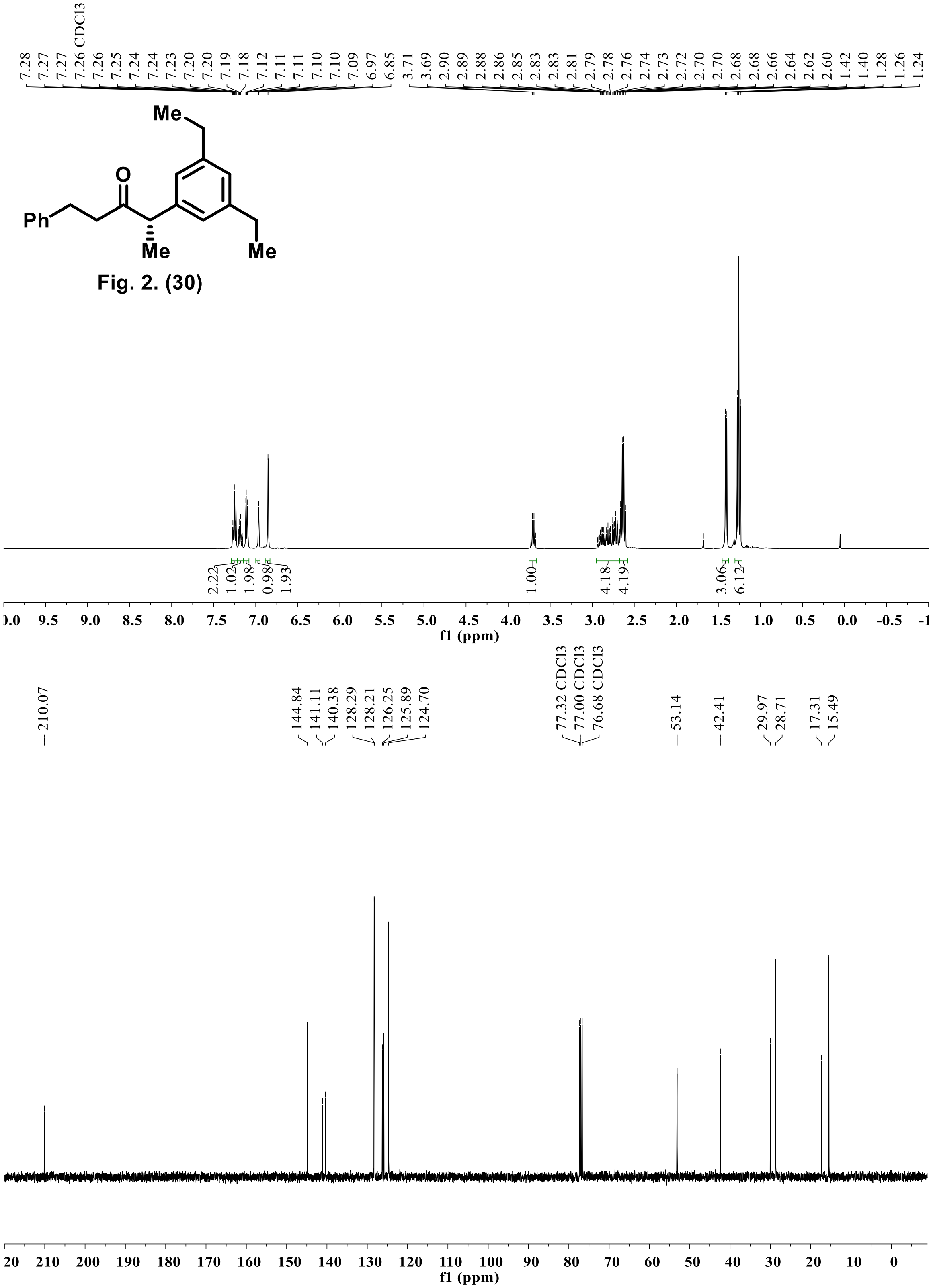

Supplementary Figure 41. ^{1}H NMR and ^{13}C NMR spectrum of **30**.

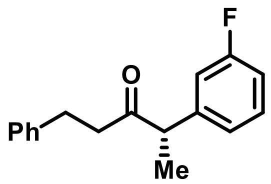

Fig. 2. (31)

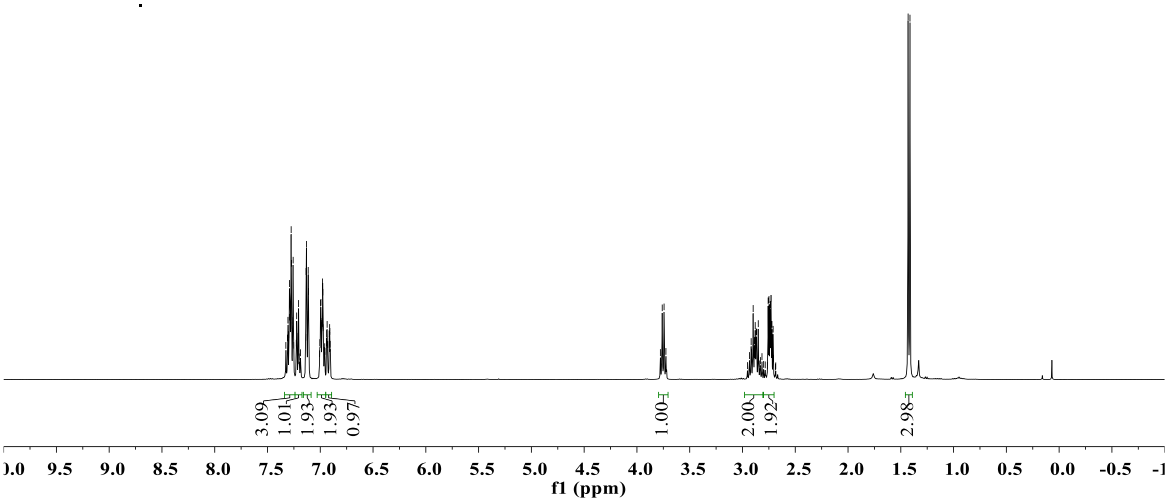

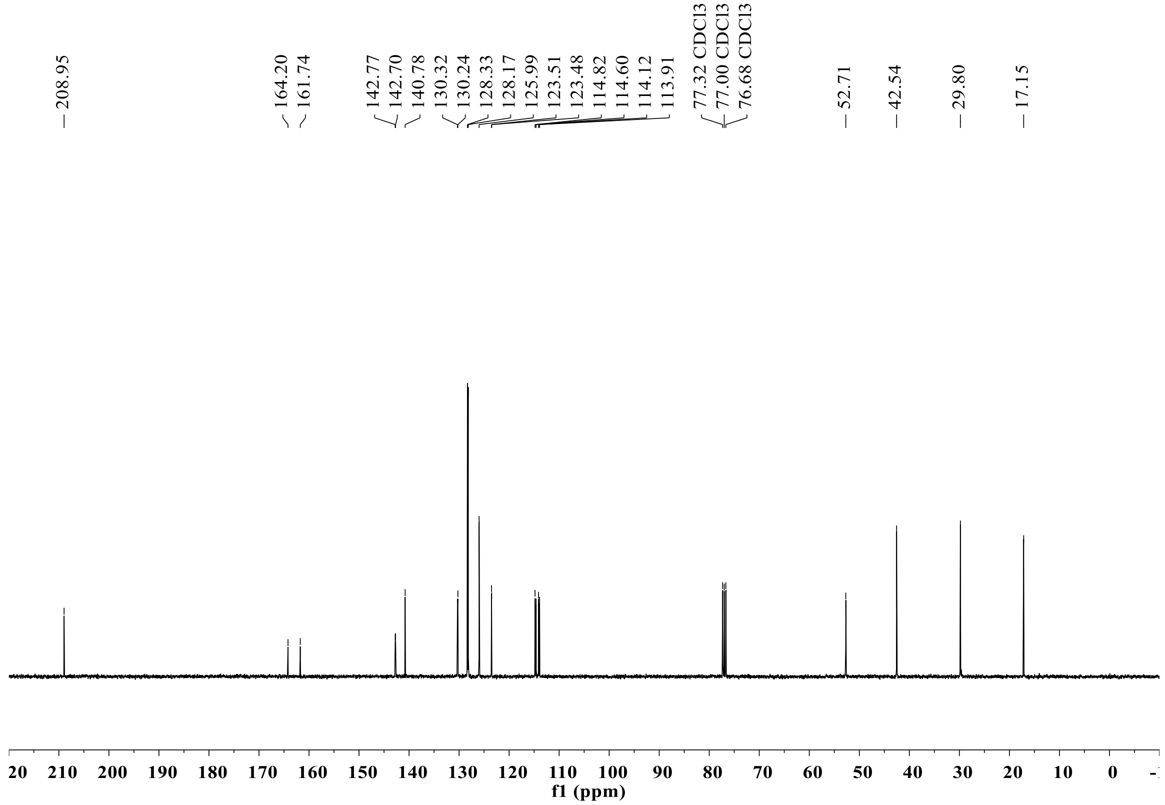

Supplementary Figure 42. ^{1}H NMR and ^{13}C NMR spectrum of **31.**

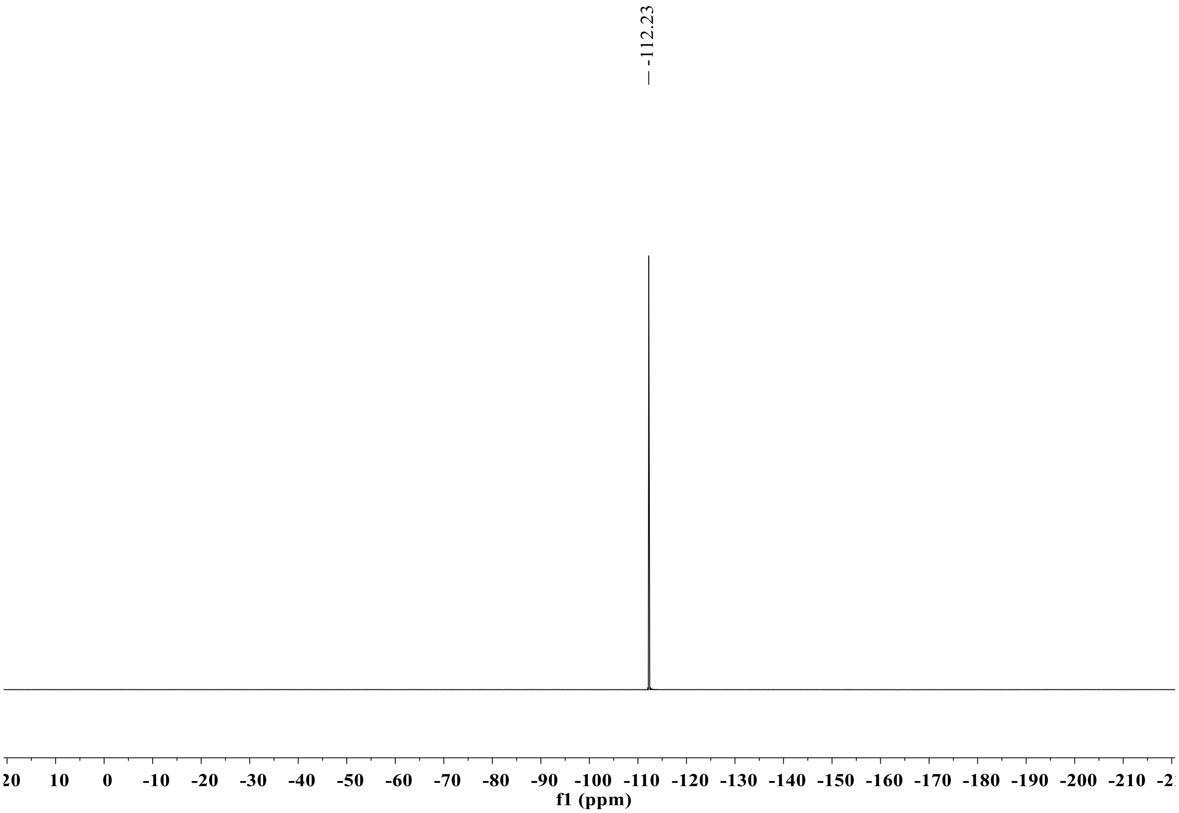

Supplementary Figure 43. ^{19}F NMR spectrum of **31.**

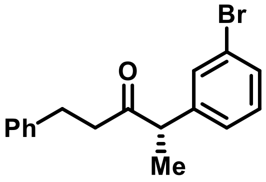

Fig. 2. (32)

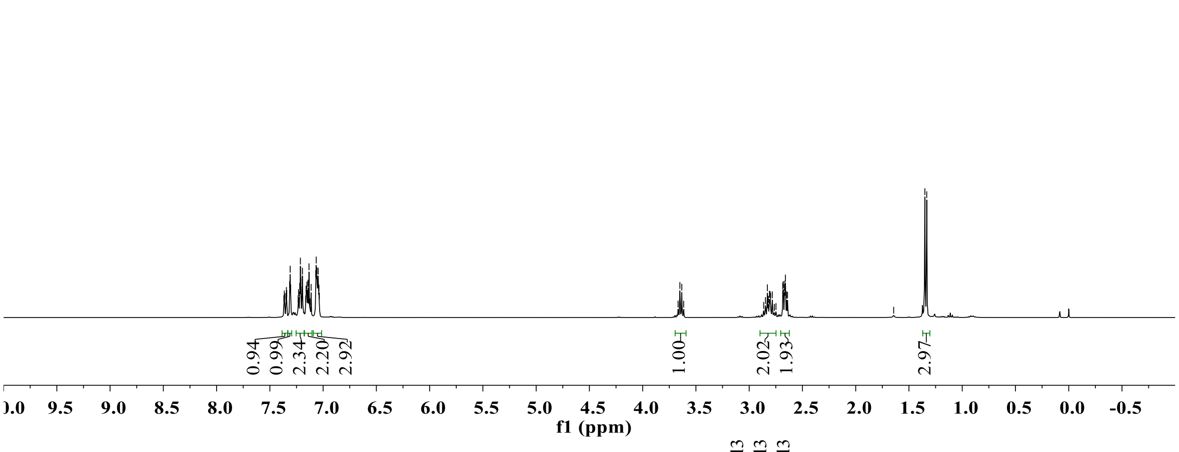

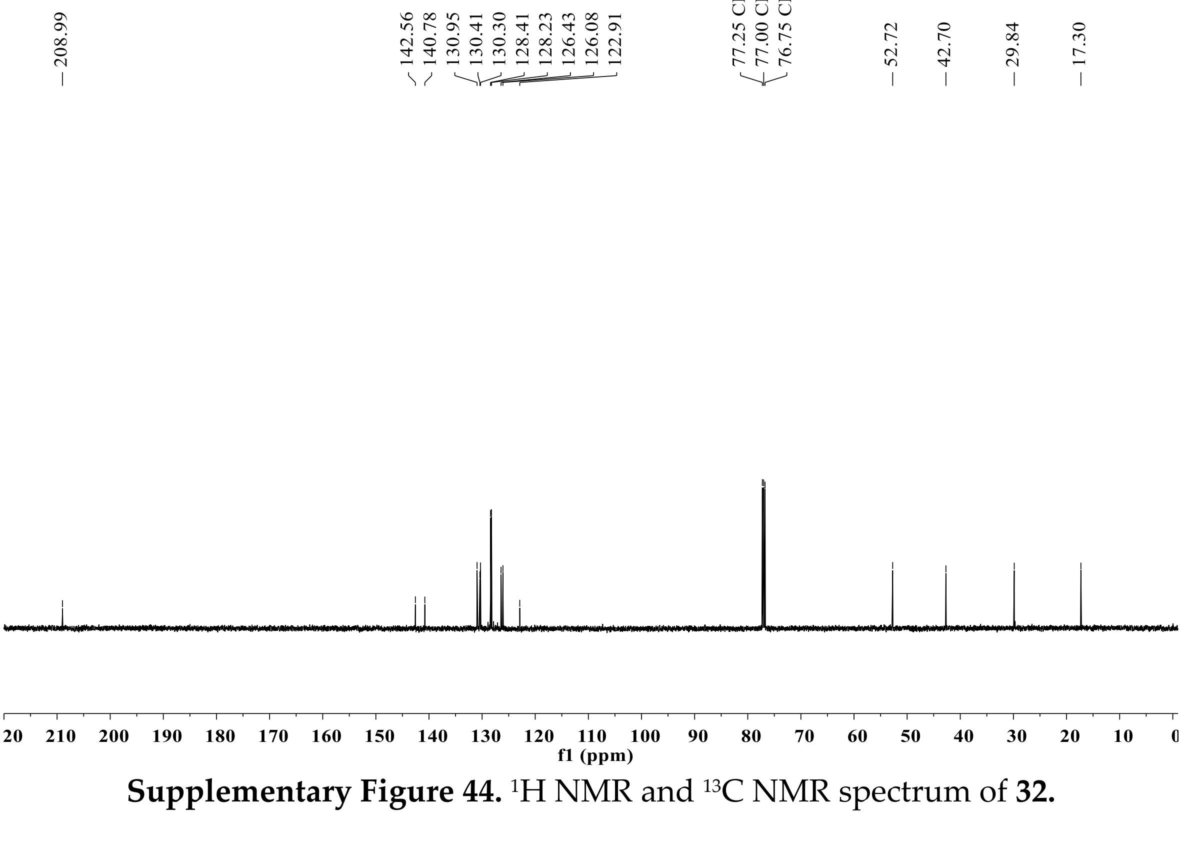

Supplementary Figure 44. ^{1}H NMR and ^{13}C NMR spectrum of **32.**

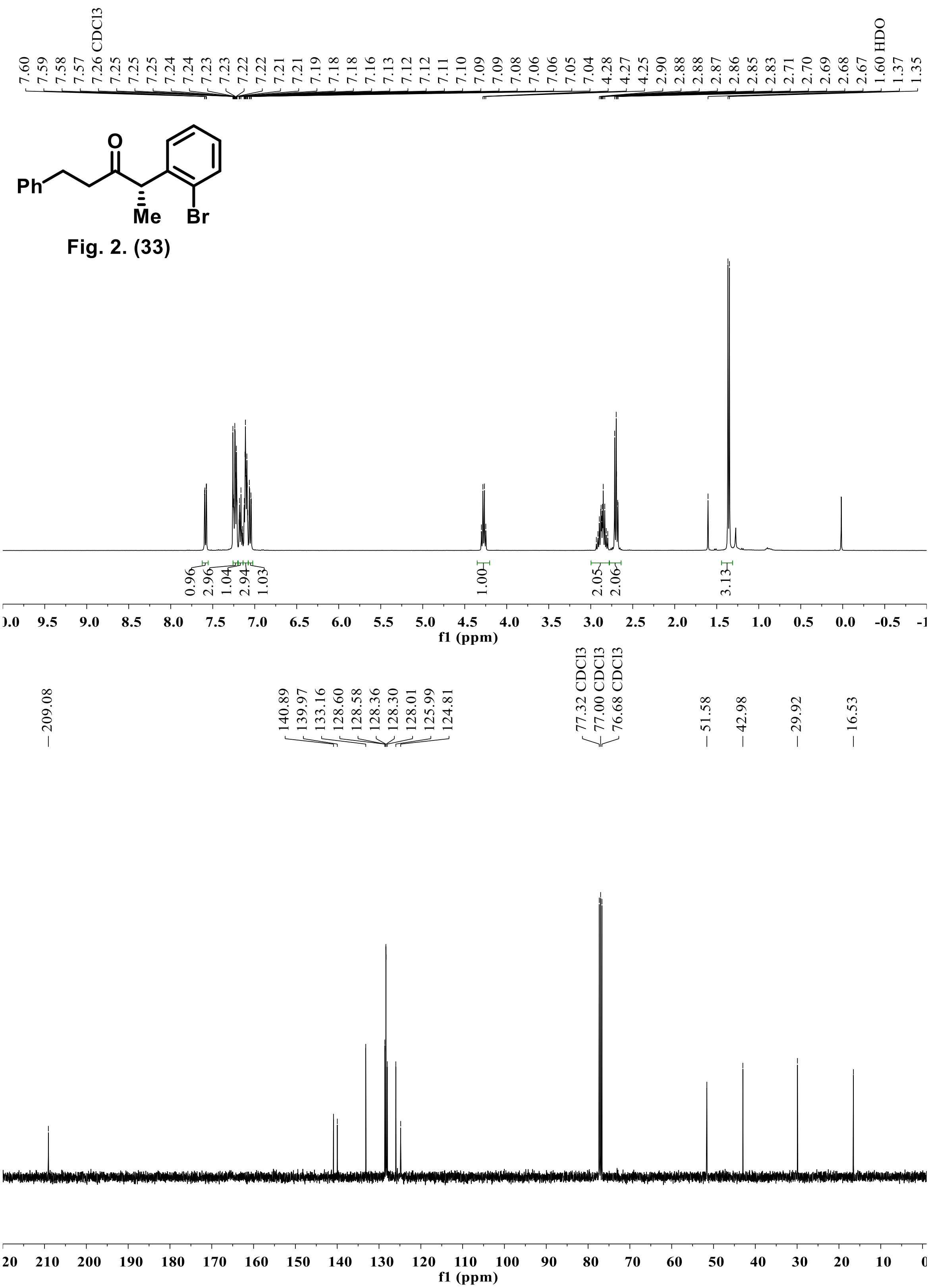

Supplementary Figure 45. ^{1}H NMR and ^{13}C NMR spectrum of **33.**

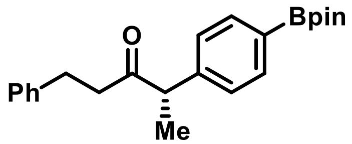

Fig. 2. (34)

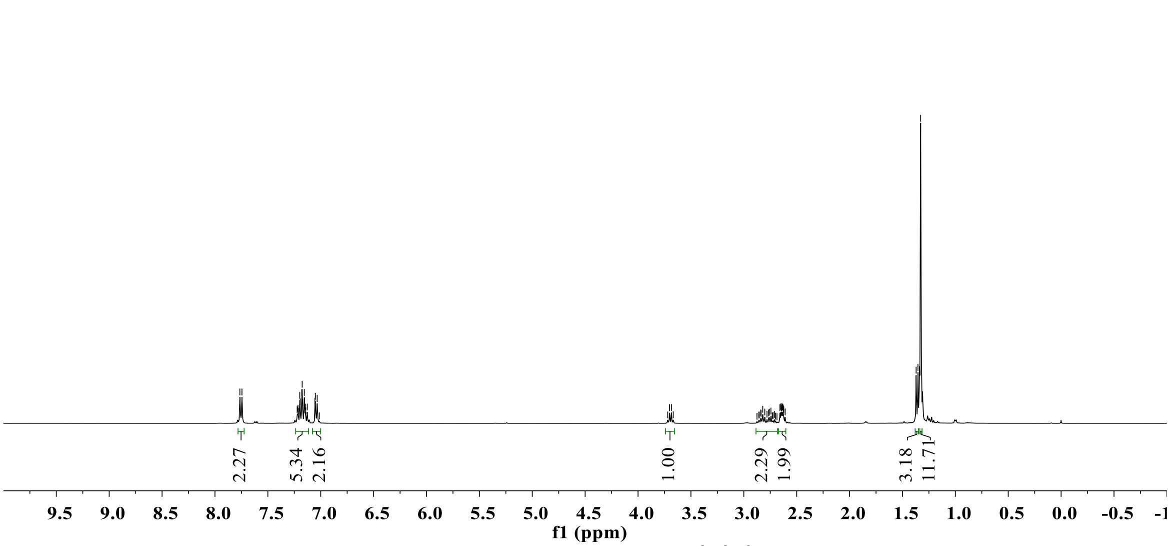

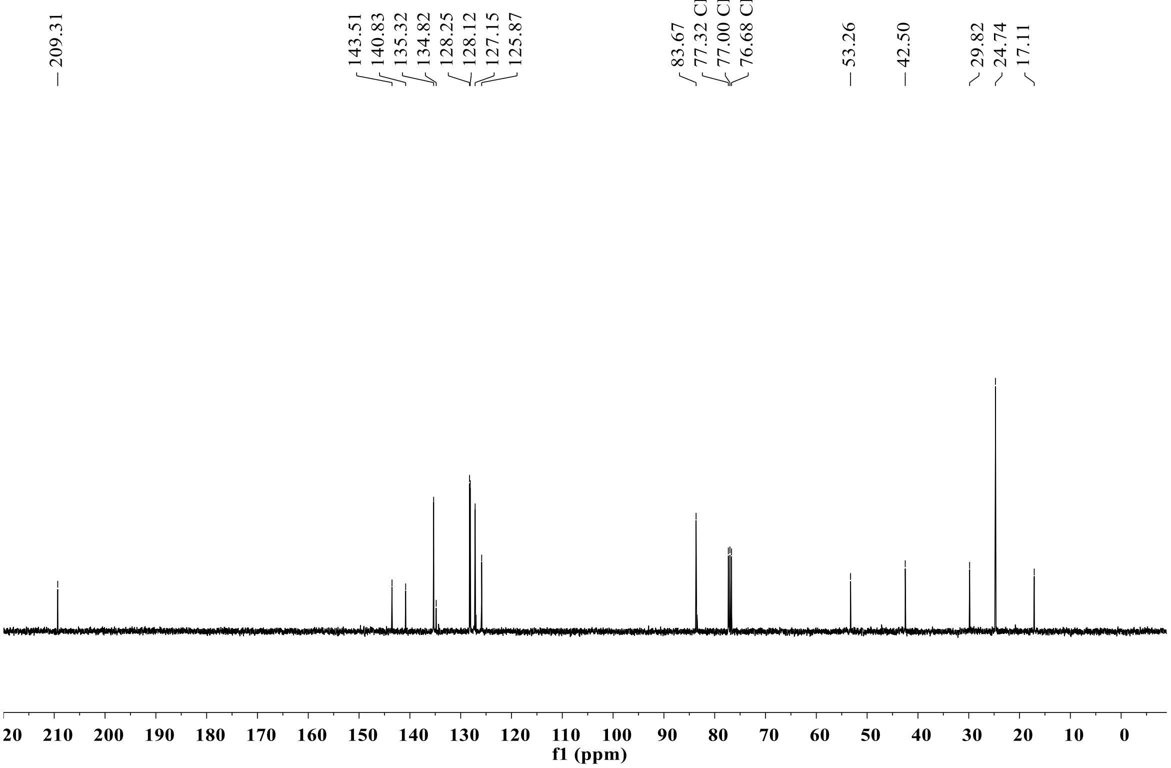

Supplementary Figure 46. ^{1}H NMR and ^{13}C NMR spectrum of **34.**

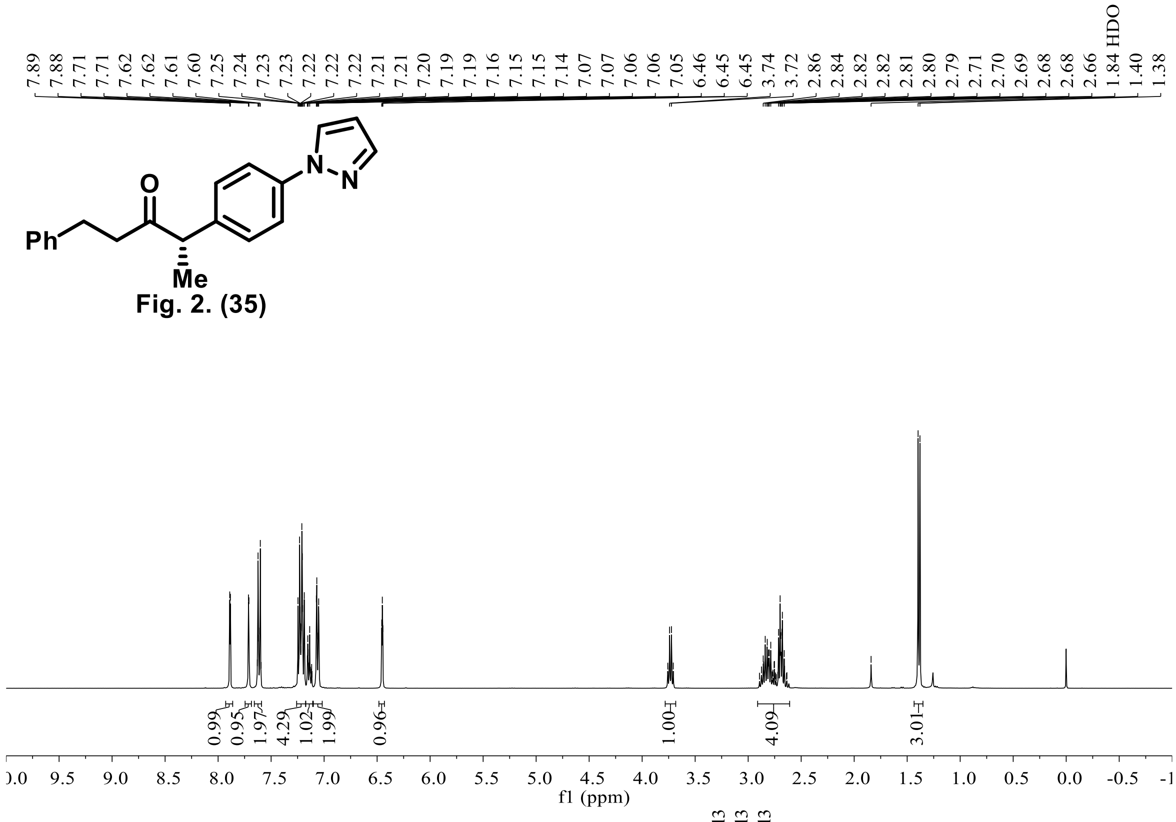

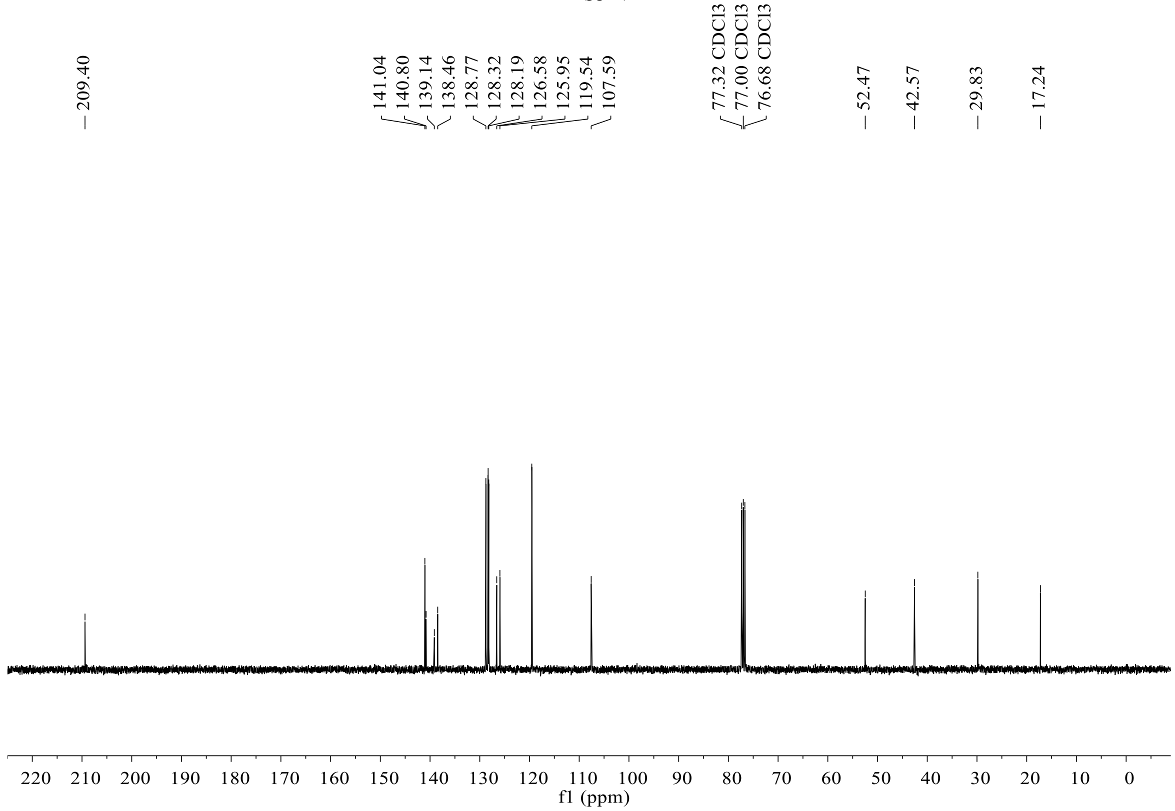

Supplementary Figure 47. ^{1}H NMR and ^{13}C NMR spectrum of **35.**

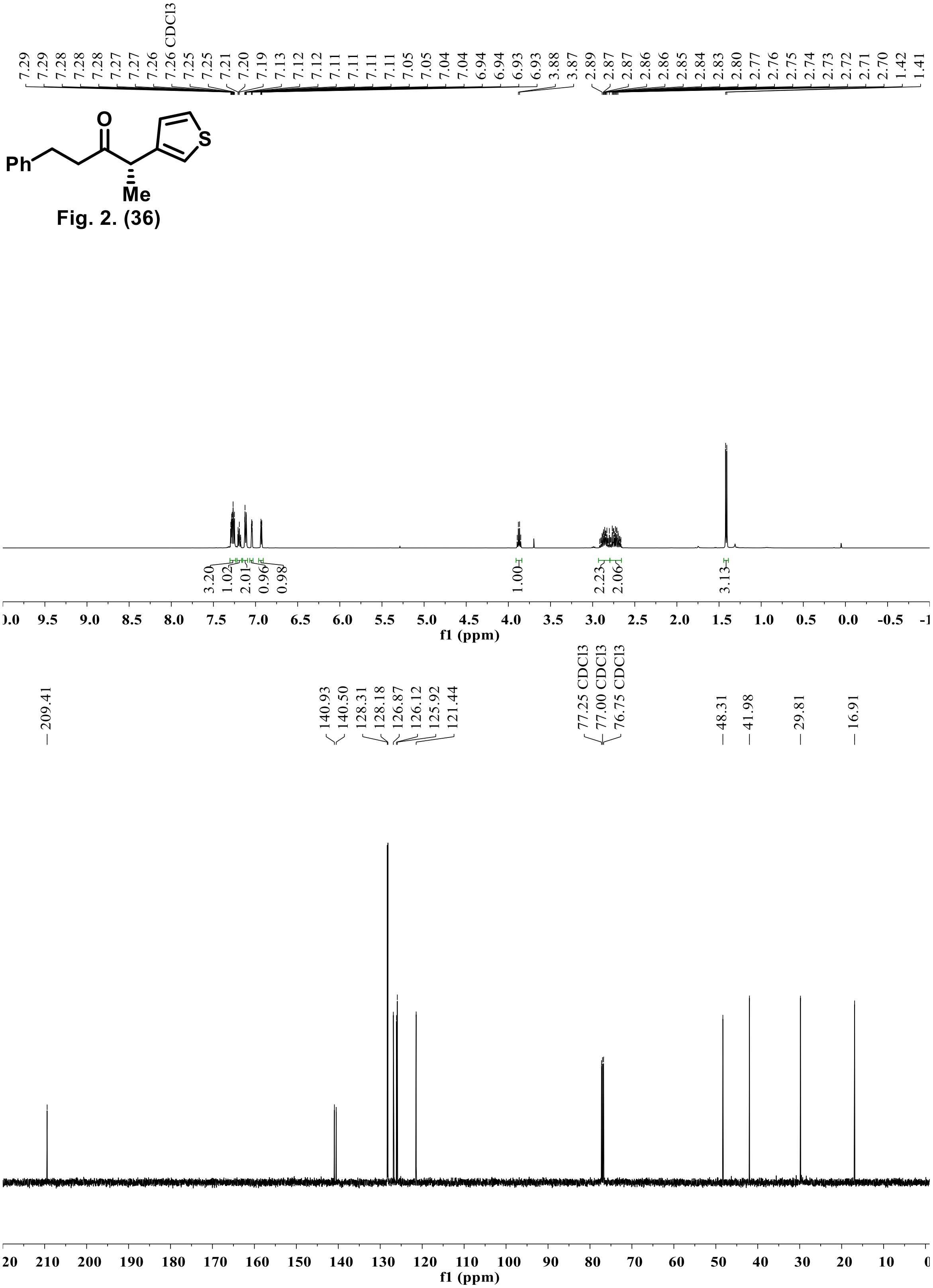

Supplementary Figure 48. ^{1}H NMR and ^{13}C NMR spectrum of **36.**

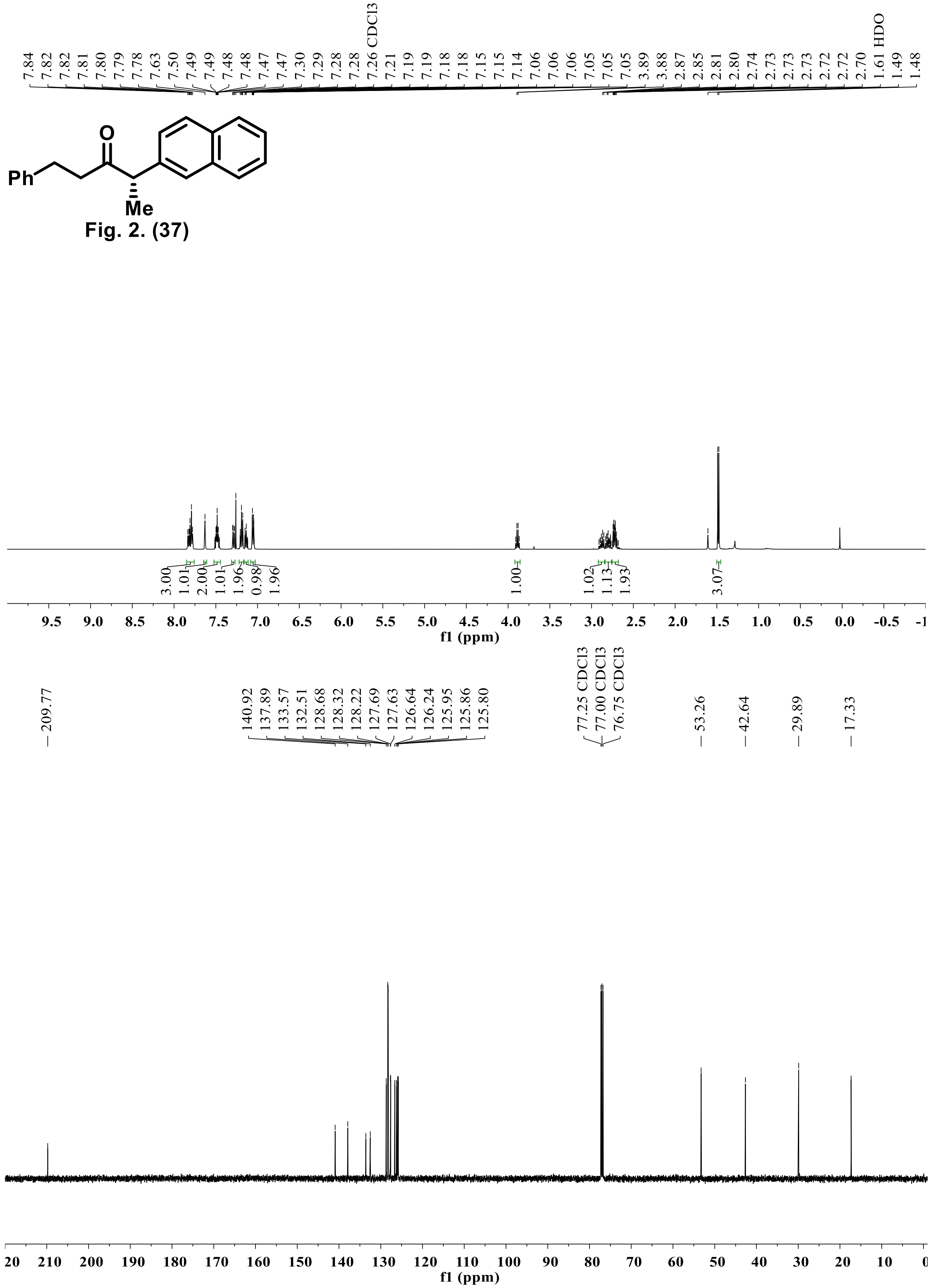

Supplementary Figure 49. ^{1}H NMR and ^{13}C NMR spectrum of **37.**

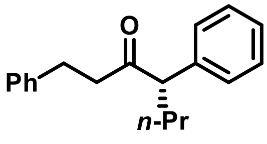

Fig. 2. (38)

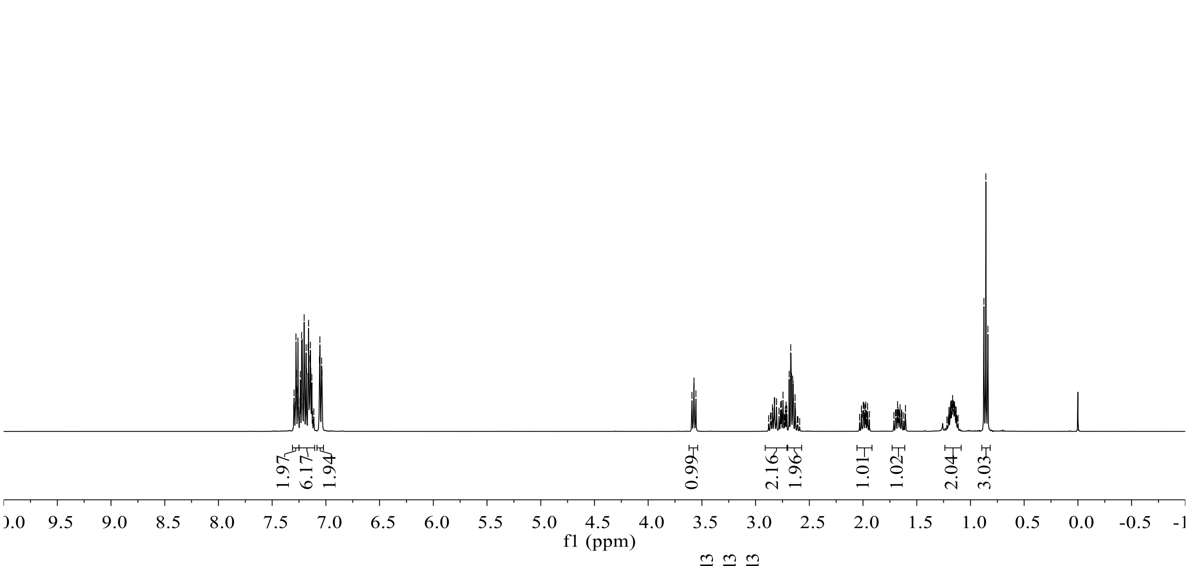

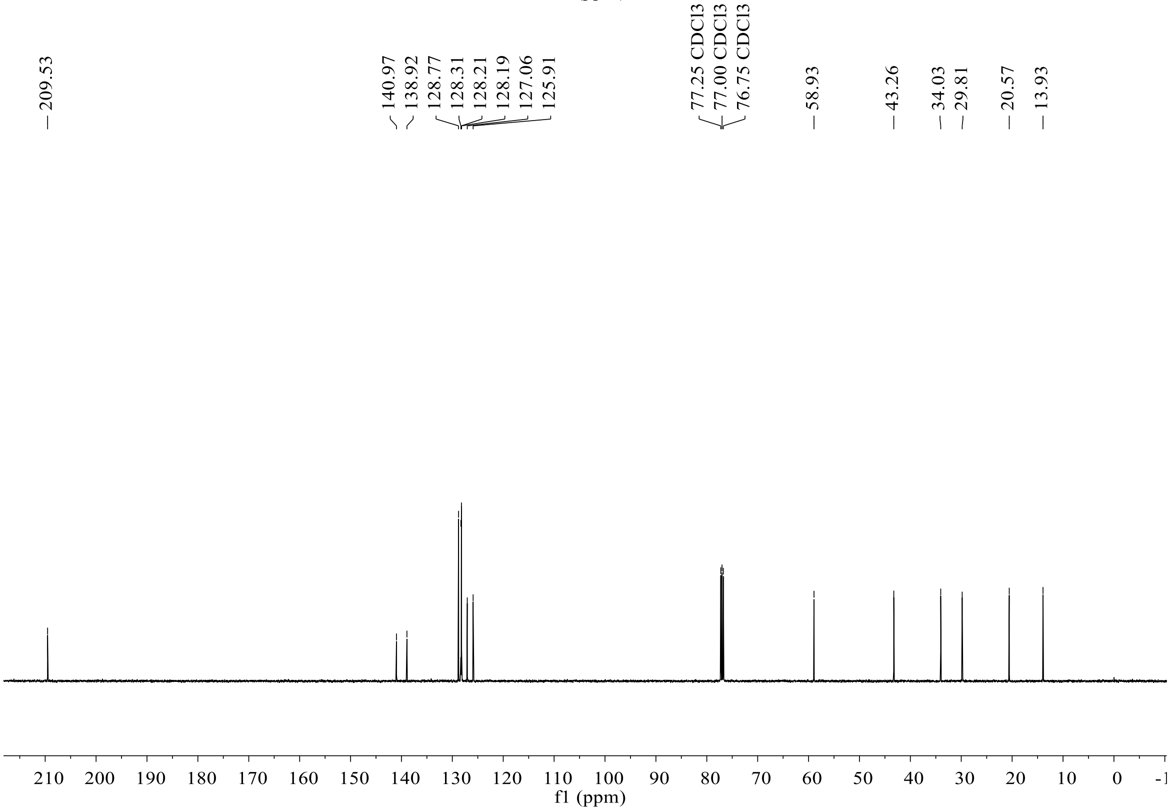

Supplementary Figure 50. ^{1}H NMR and ^{13}C NMR spectrum of **38.**

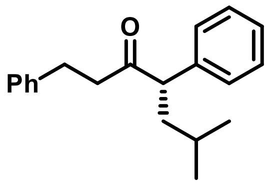

Fig. 2. (39)

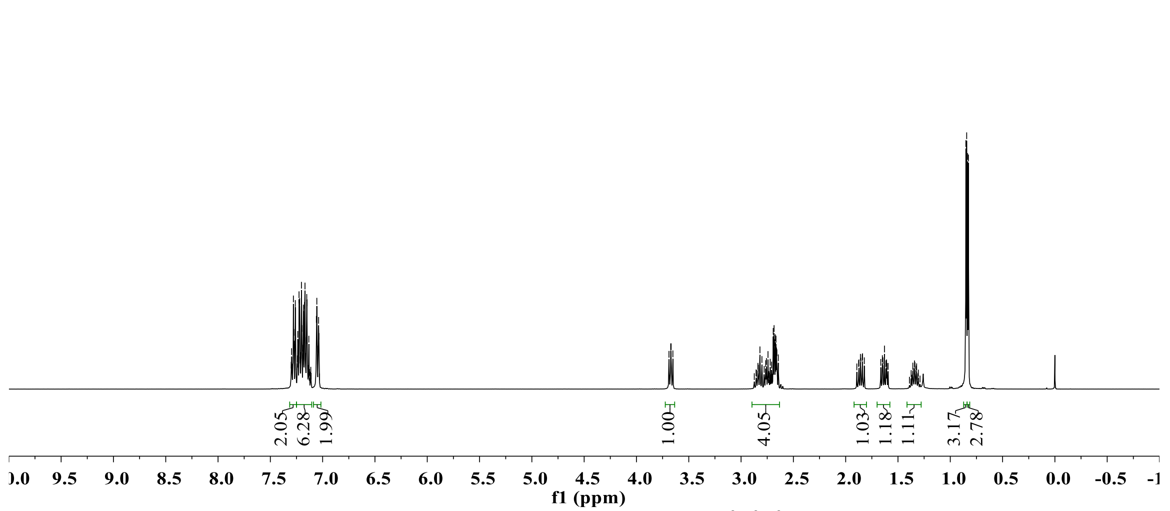

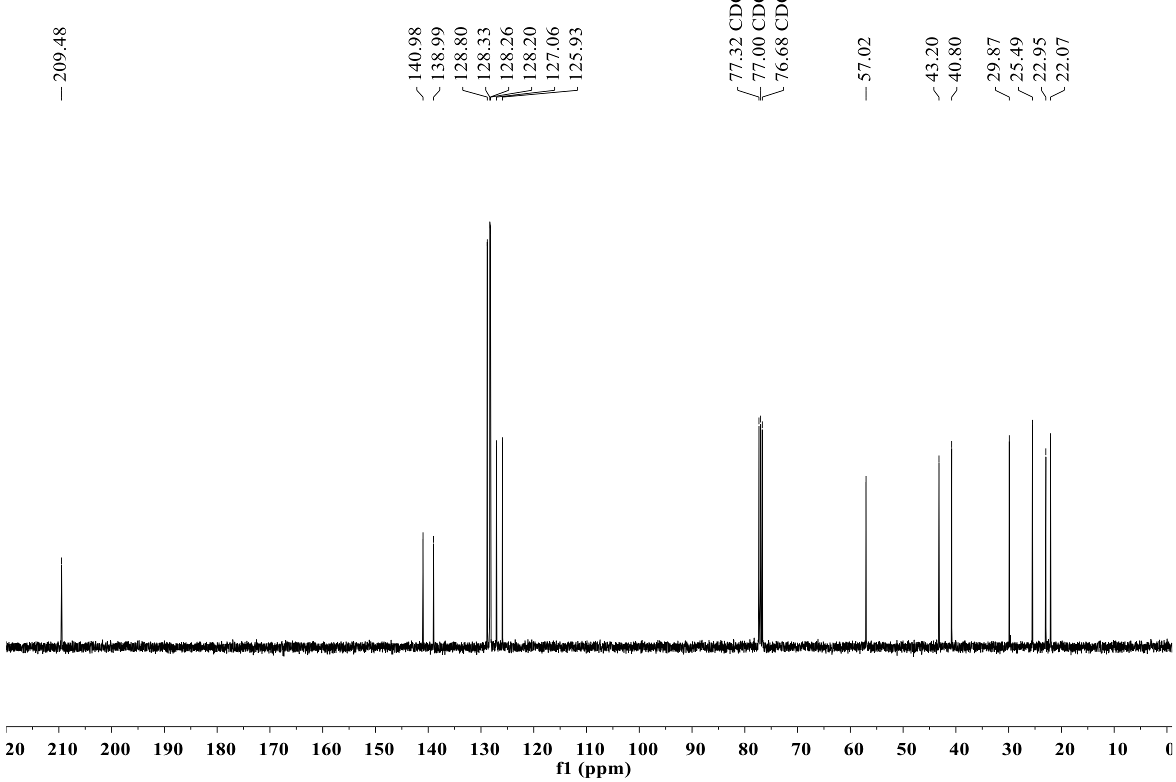

Supplementary Figure 51. ^{1}H NMR and ^{13}C NMR spectrum of **39.**

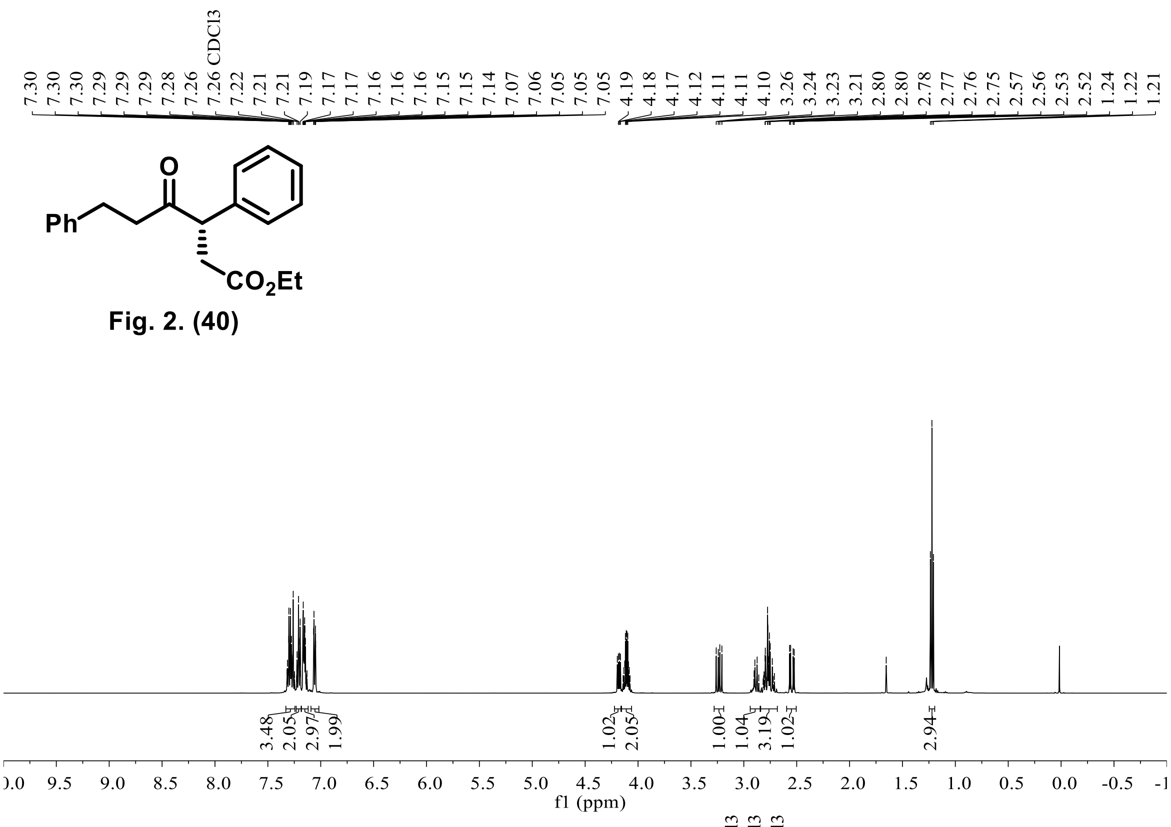

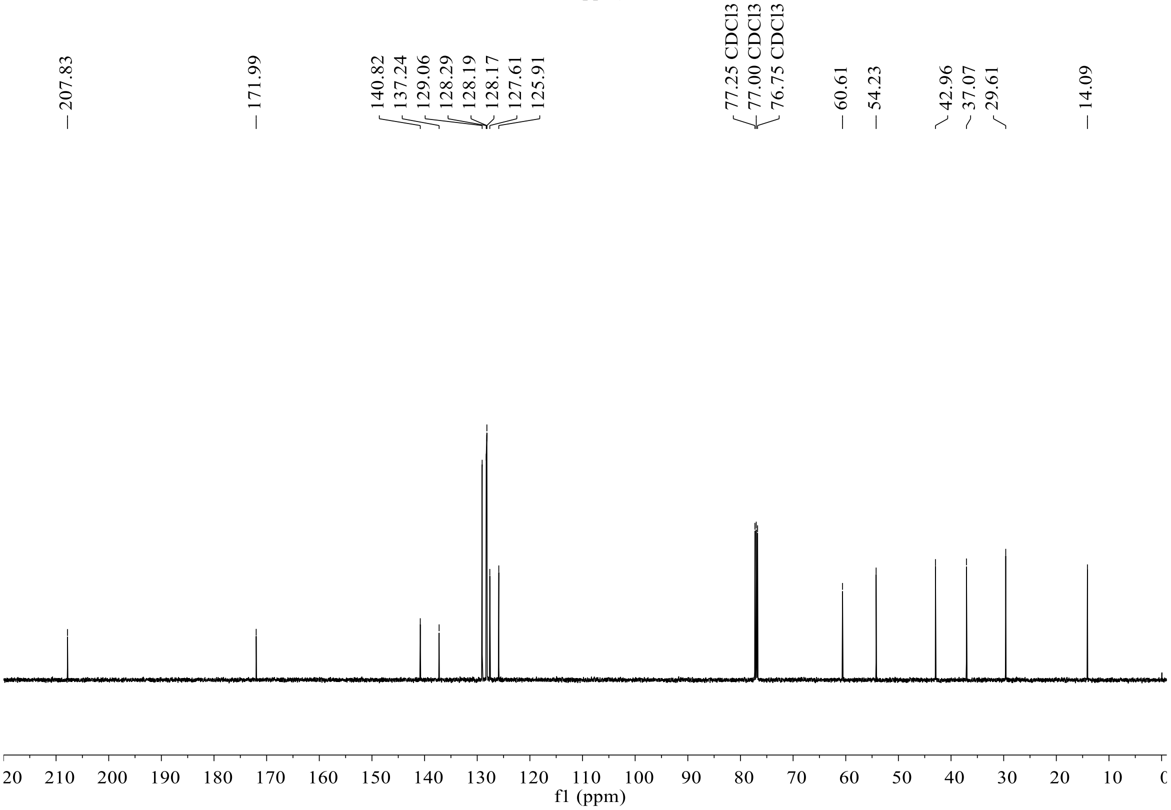

Supplementary Figure 52. ^{1}H NMR and ^{13}C NMR spectrum of **40.**

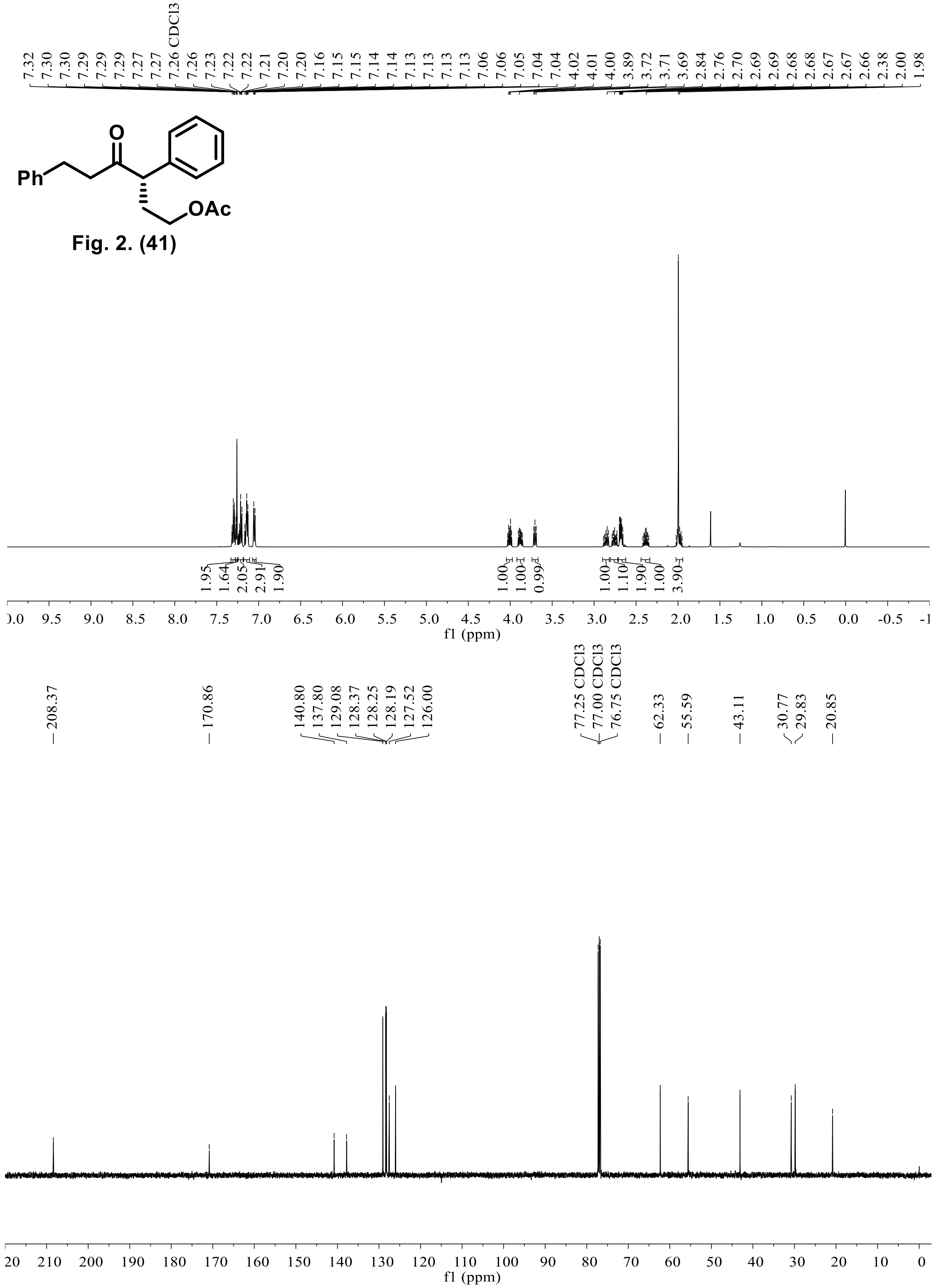

Supplementary Figure 53. ^{1}H NMR and ^{13}C NMR spectrum of **41.**

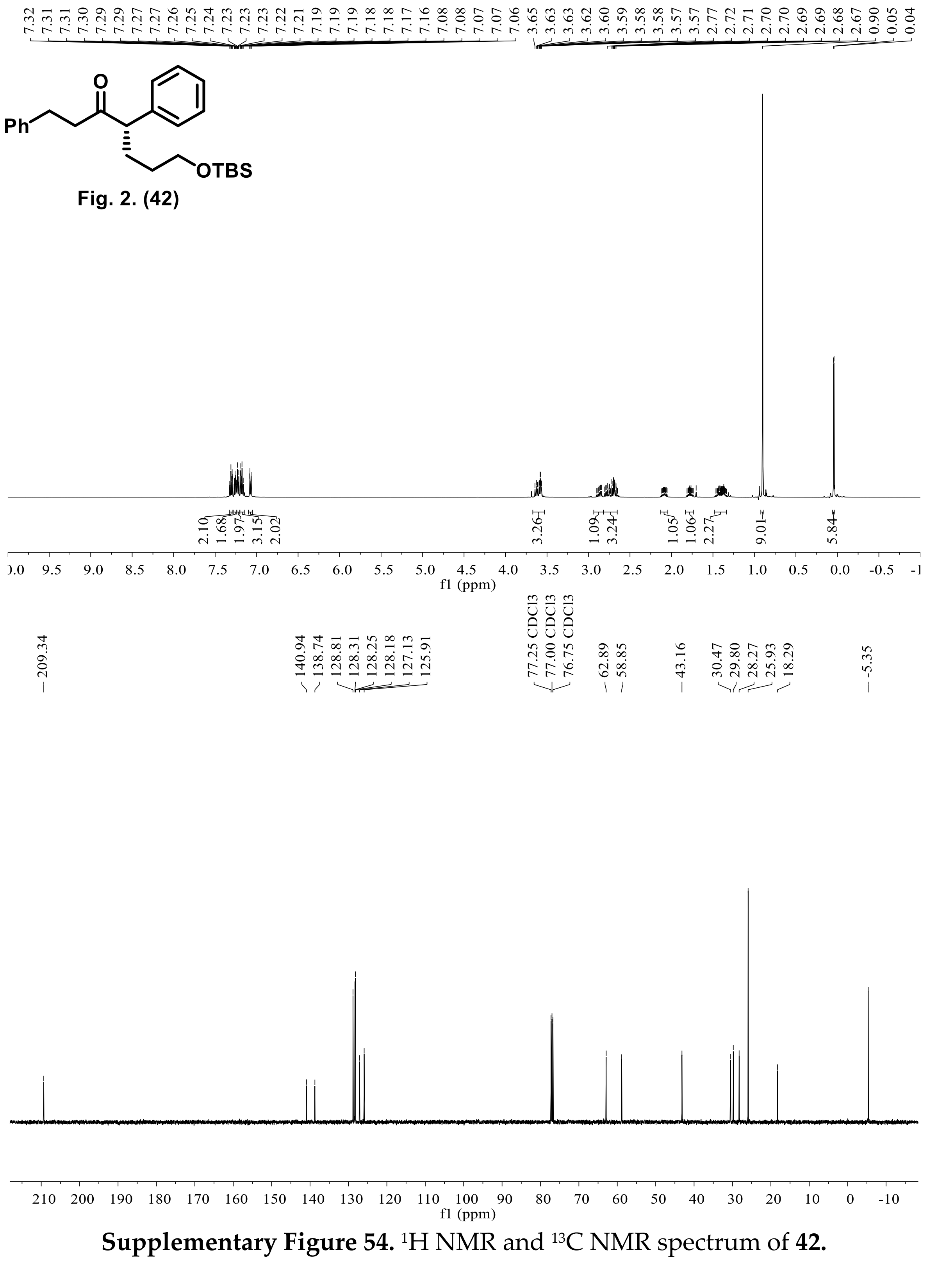

Supplementary Figure 54. ^{1}H NMR and ^{13}C NMR spectrum of **42.**

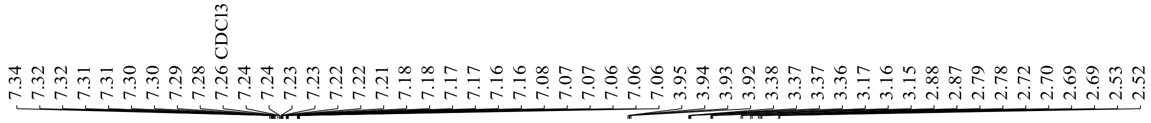

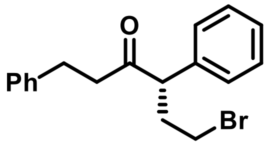

Fig. 2. (43)

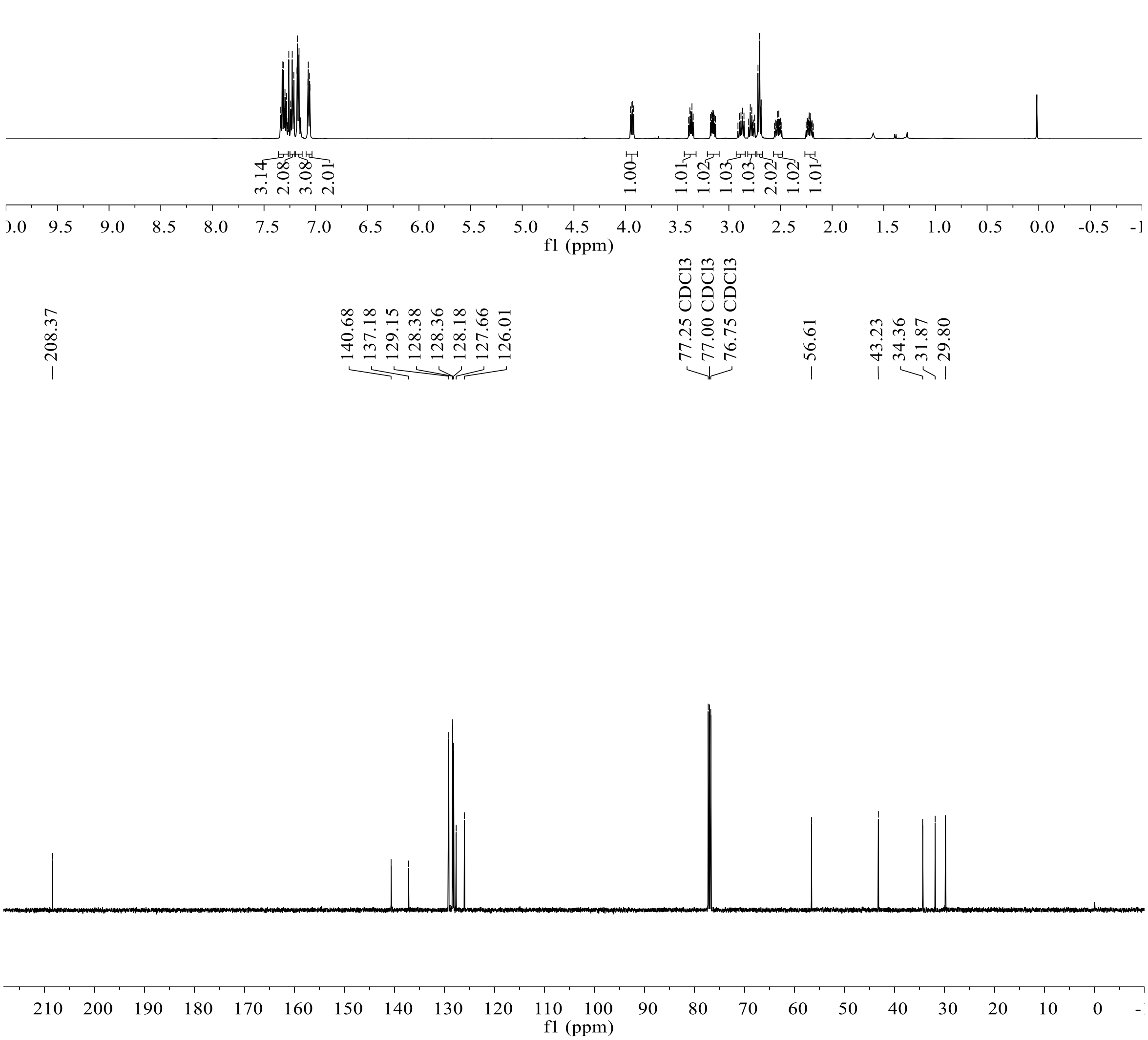

Supplementary Figure 55. ^{1}H NMR and ^{13}C NMR spectrum of **43.**

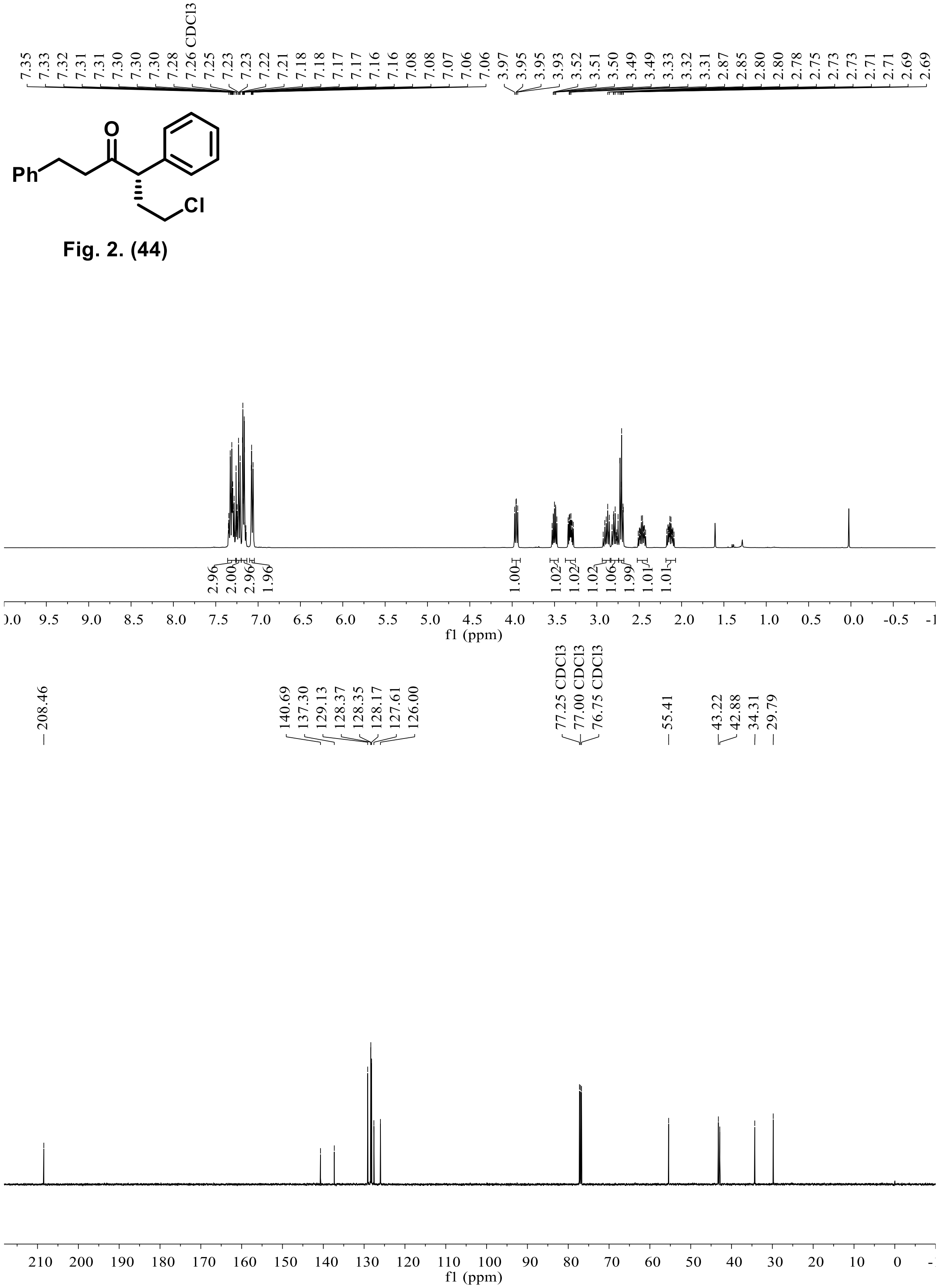

Supplementary Figure 56. ^{1}H NMR and ^{13}C NMR spectrum of **44.**

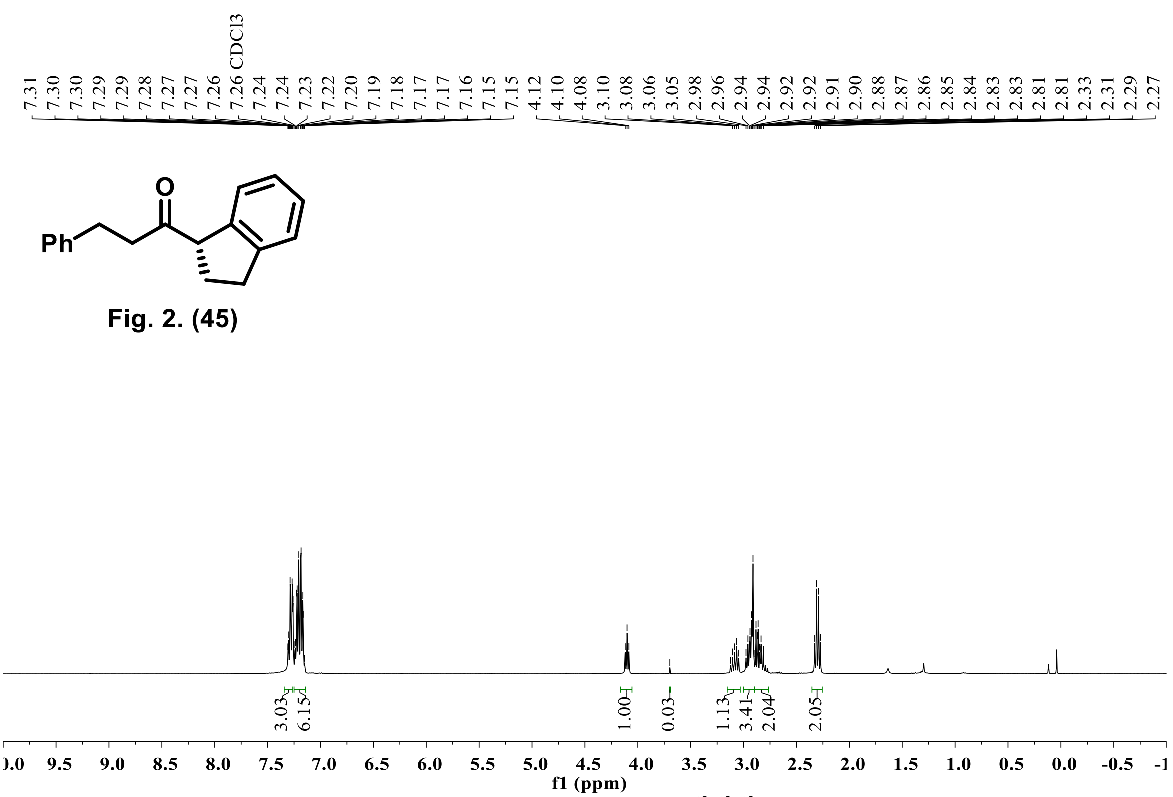

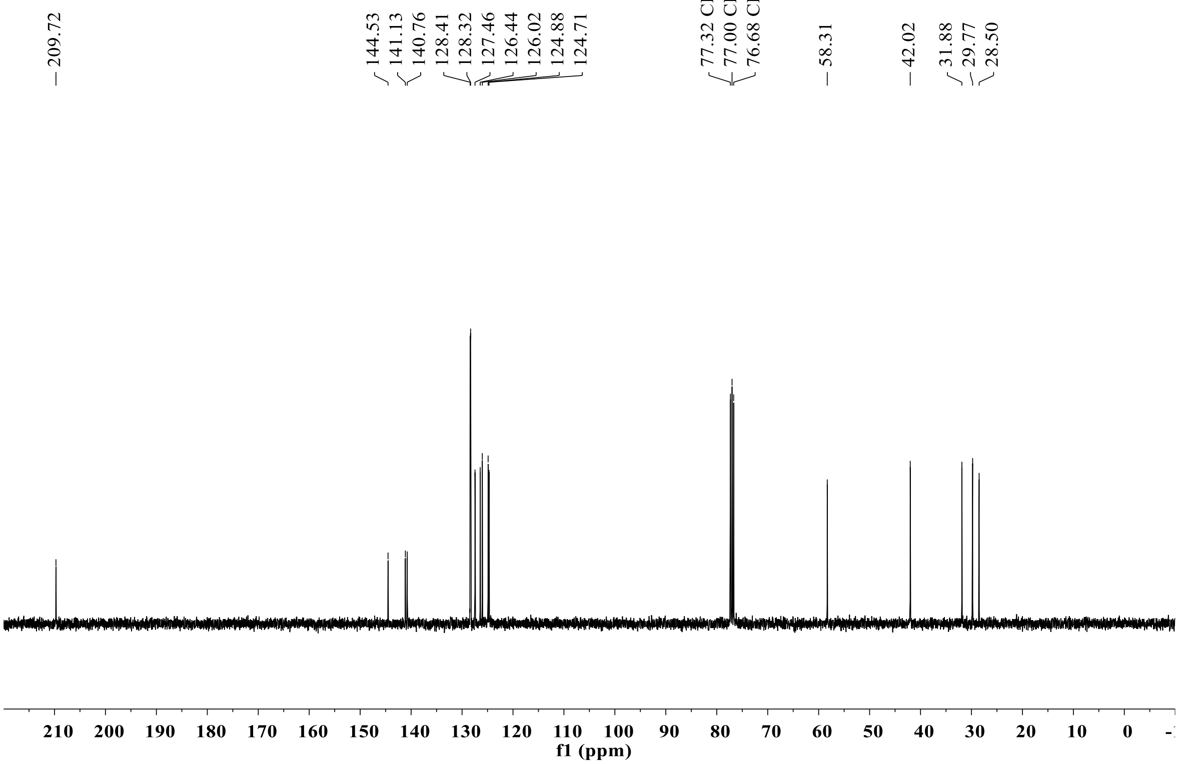

Supplementary Figure 57. ^{1}H NMR and ^{13}C NMR spectrum of **45.**

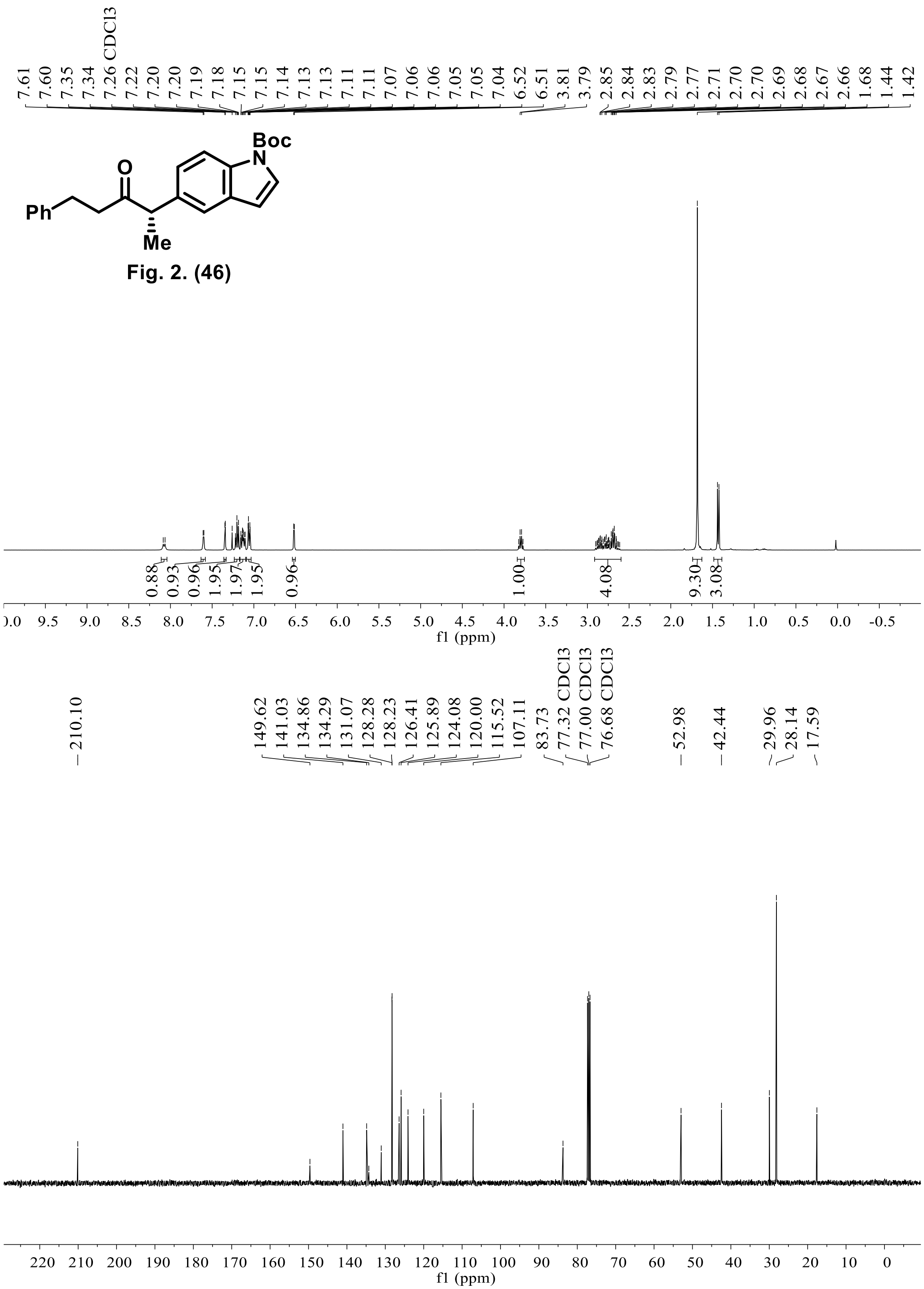

Supplementary Figure 58. ^{1}H NMR and ^{13}C NMR spectrum of **46.**

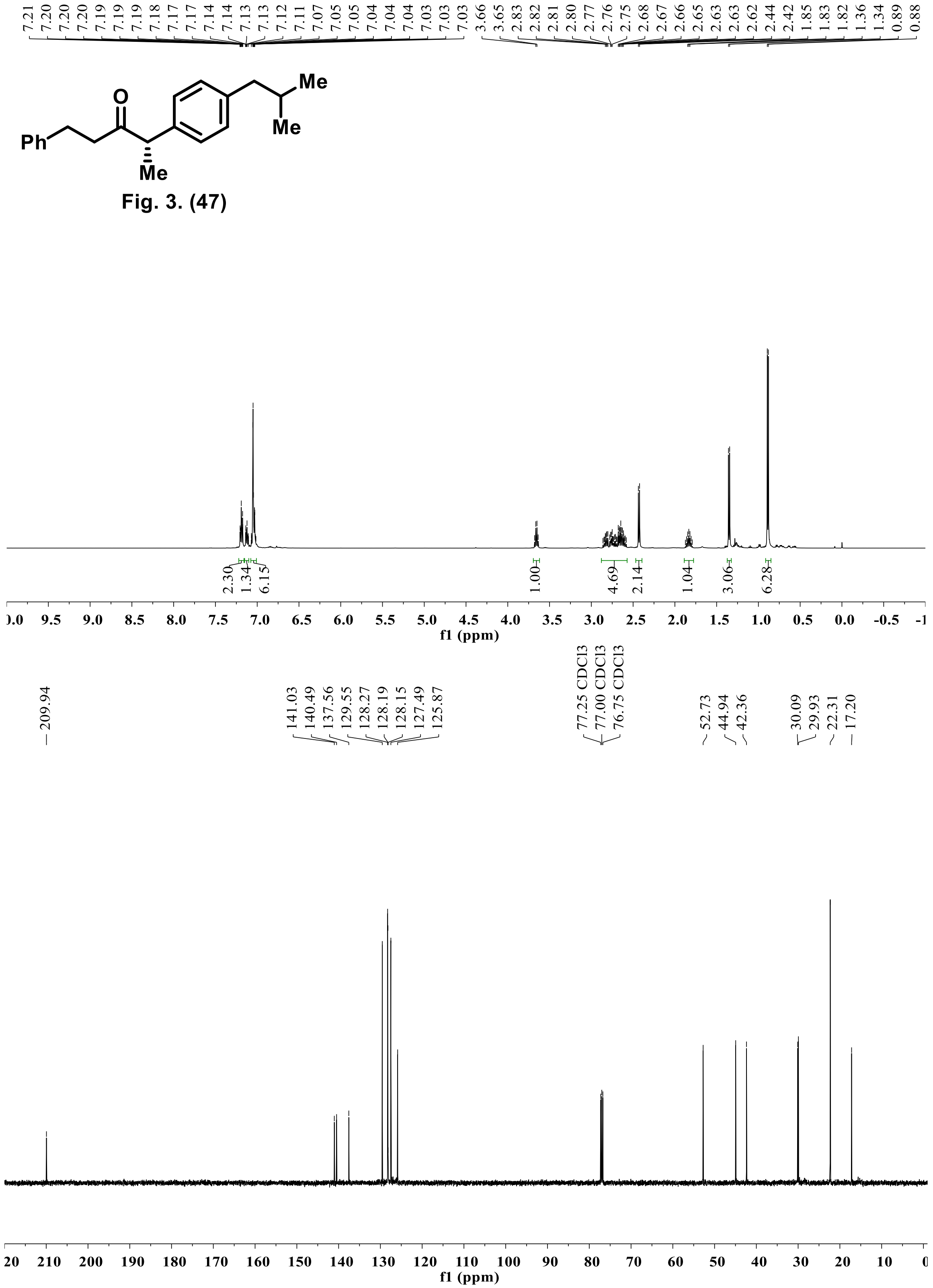

Supplementary Figure 59. ^{1}H NMR and ^{13}C NMR spectrum of **47.**

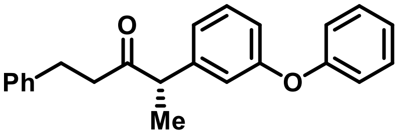

Fig. 3. (48)

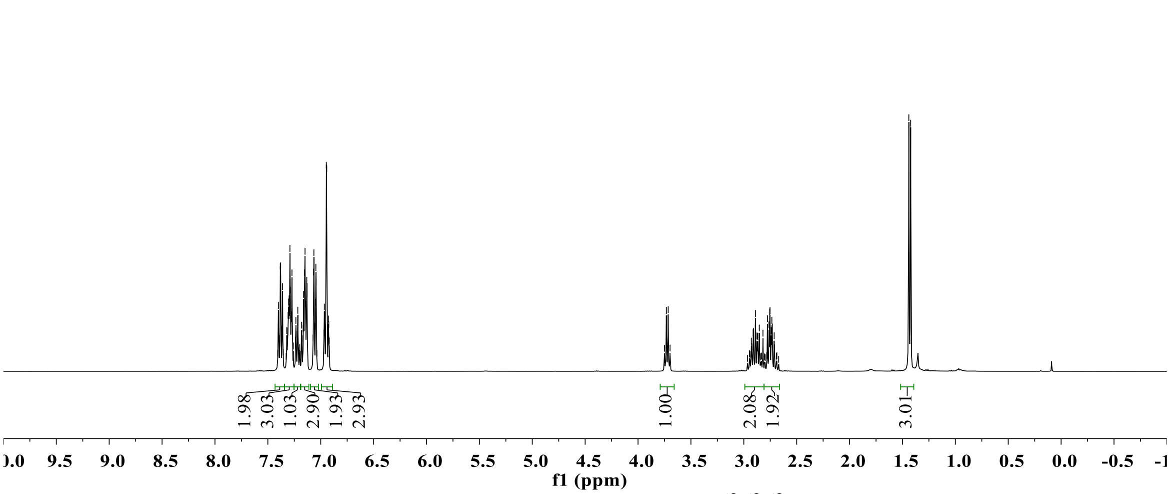

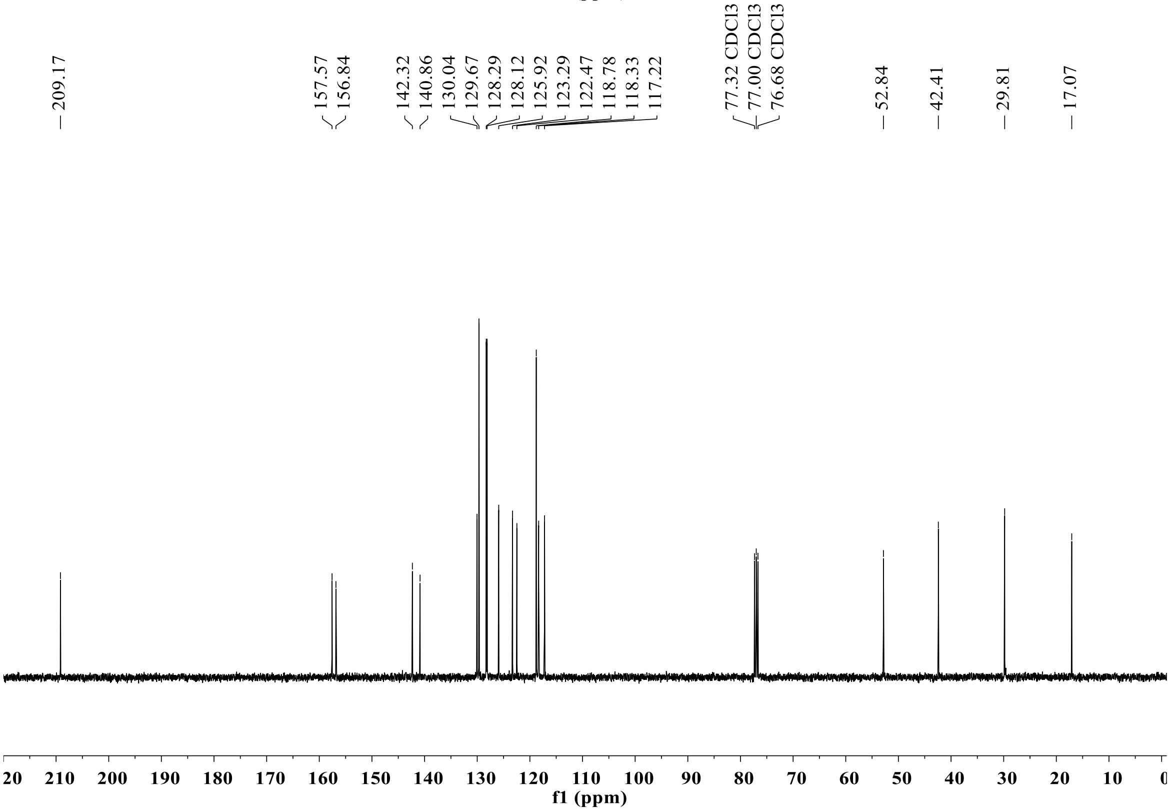

Supplementary Figure 60. ^{1}H NMR and ^{13}C NMR spectrum of **48.**

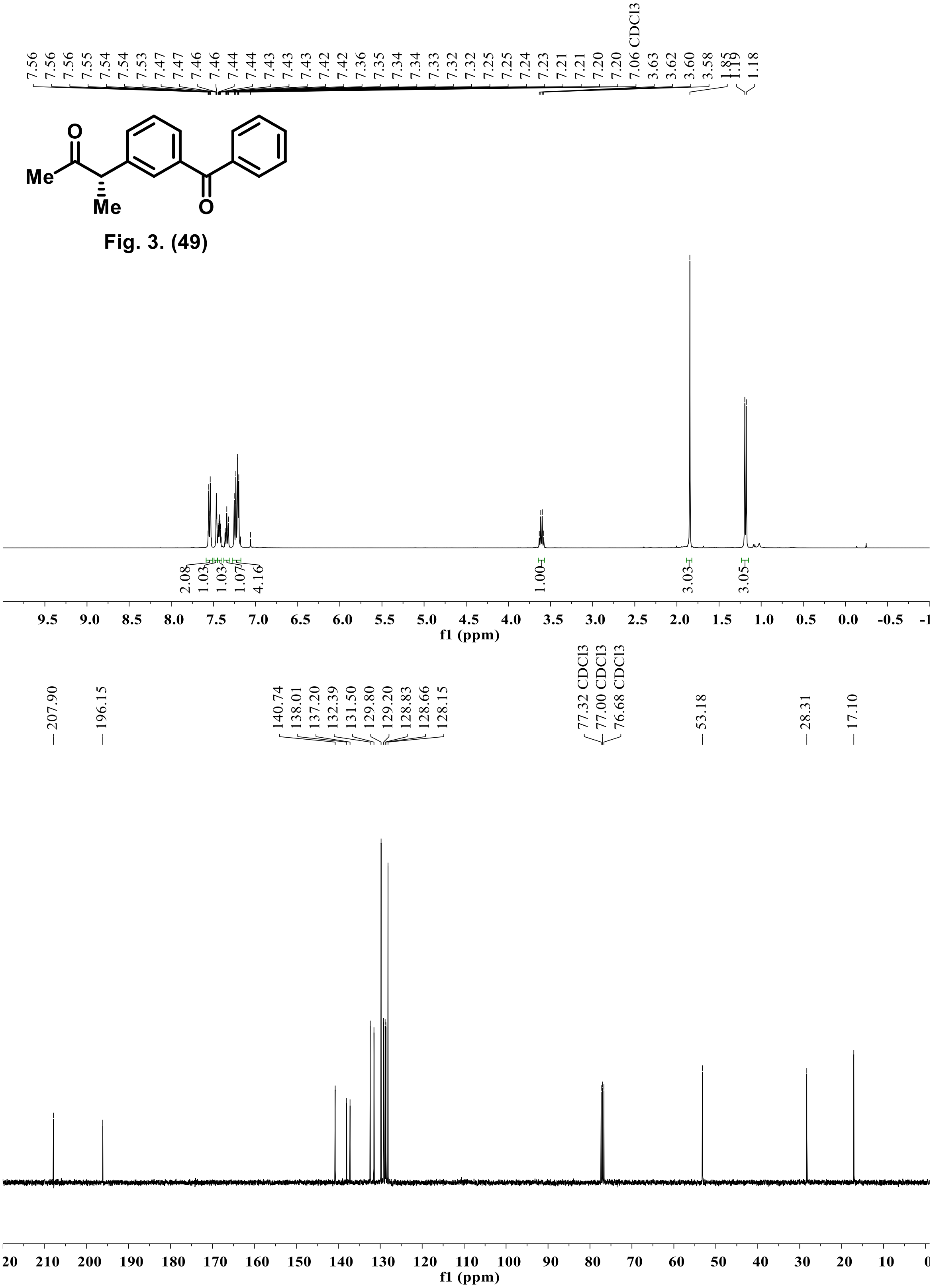

Supplementary Figure 61. ^{1}H NMR and ^{13}C NMR spectrum of **49.**

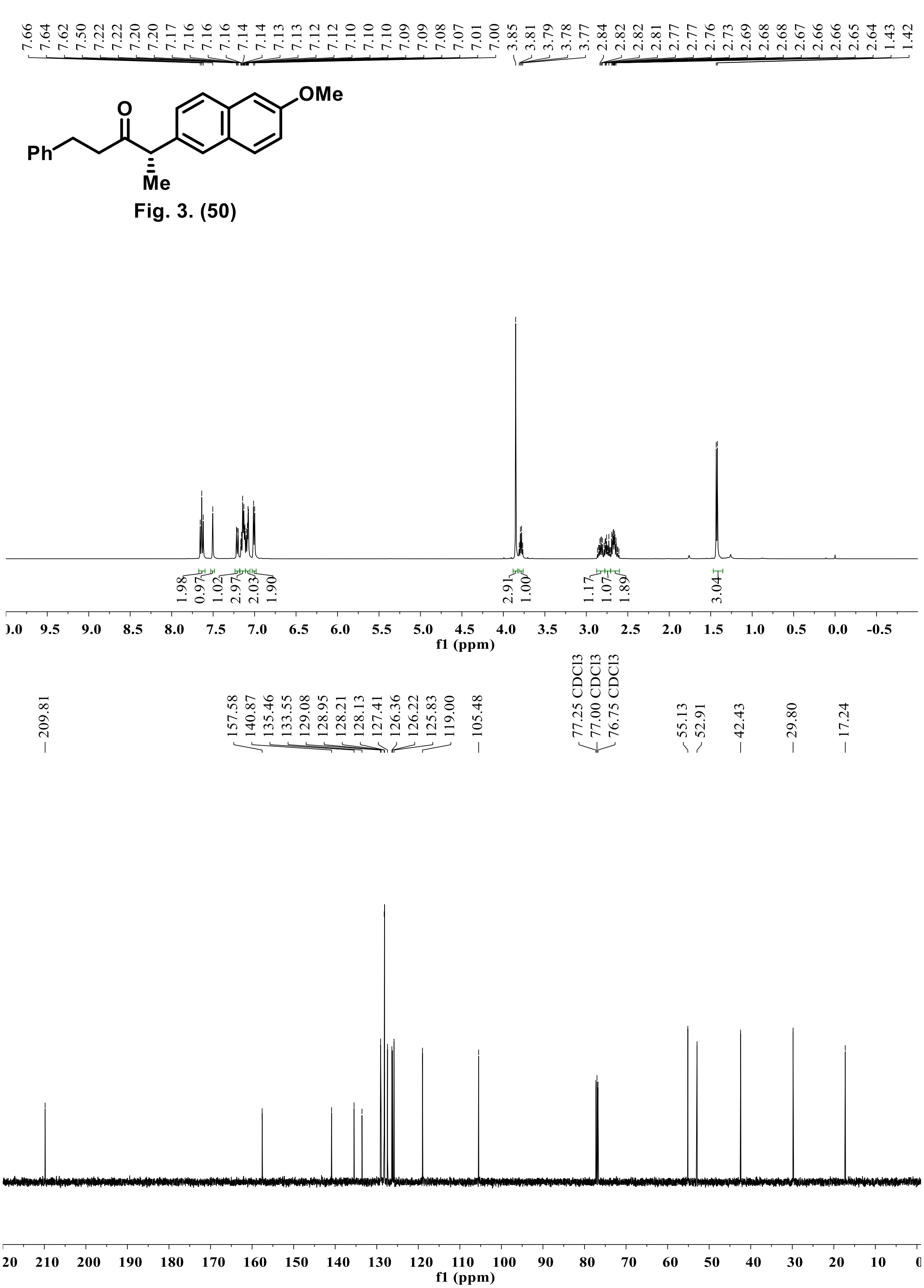

Supplementary Figure 62. ^{1}H NMR and ^{13}C NMR spectrum of **50.**

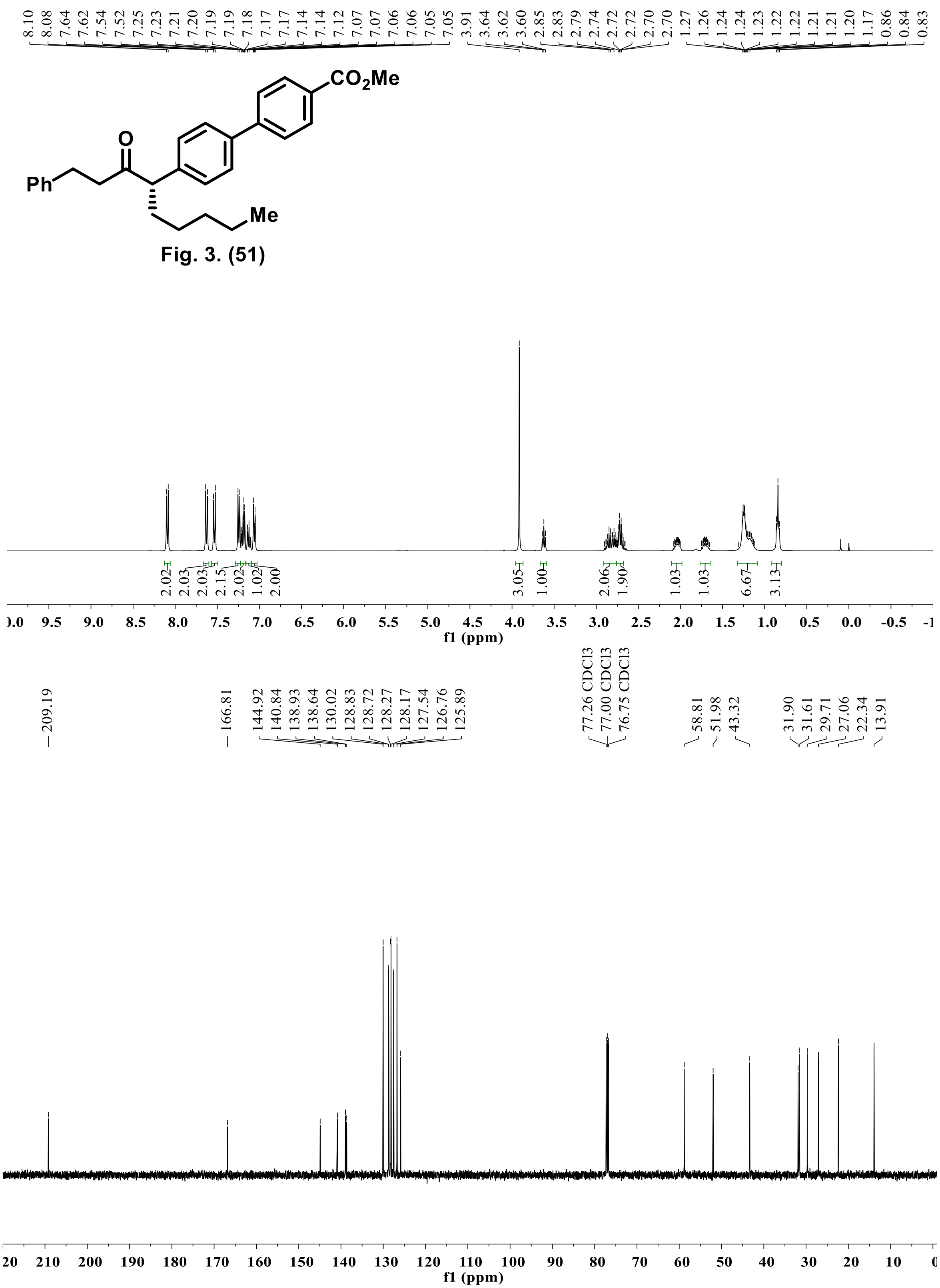

Supplementary Figure 63. ^{1}H NMR and ^{13}C NMR spectrum of **51.**

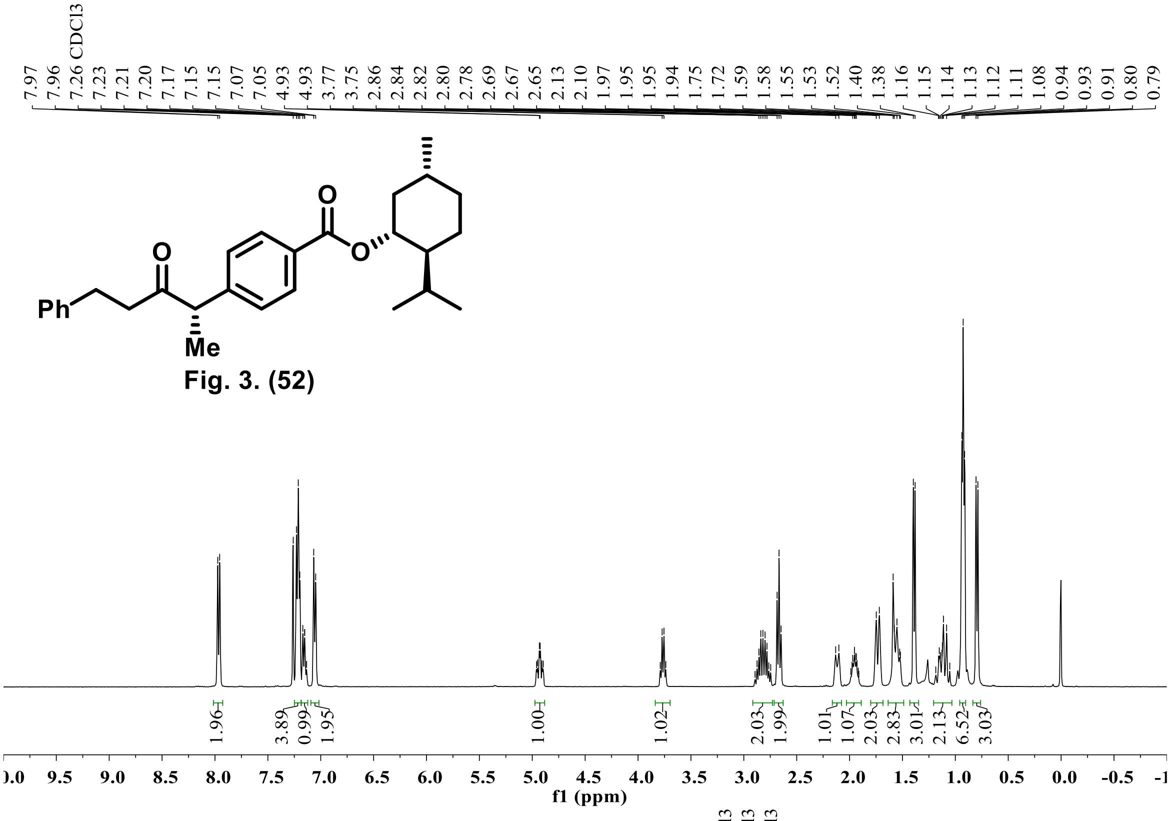

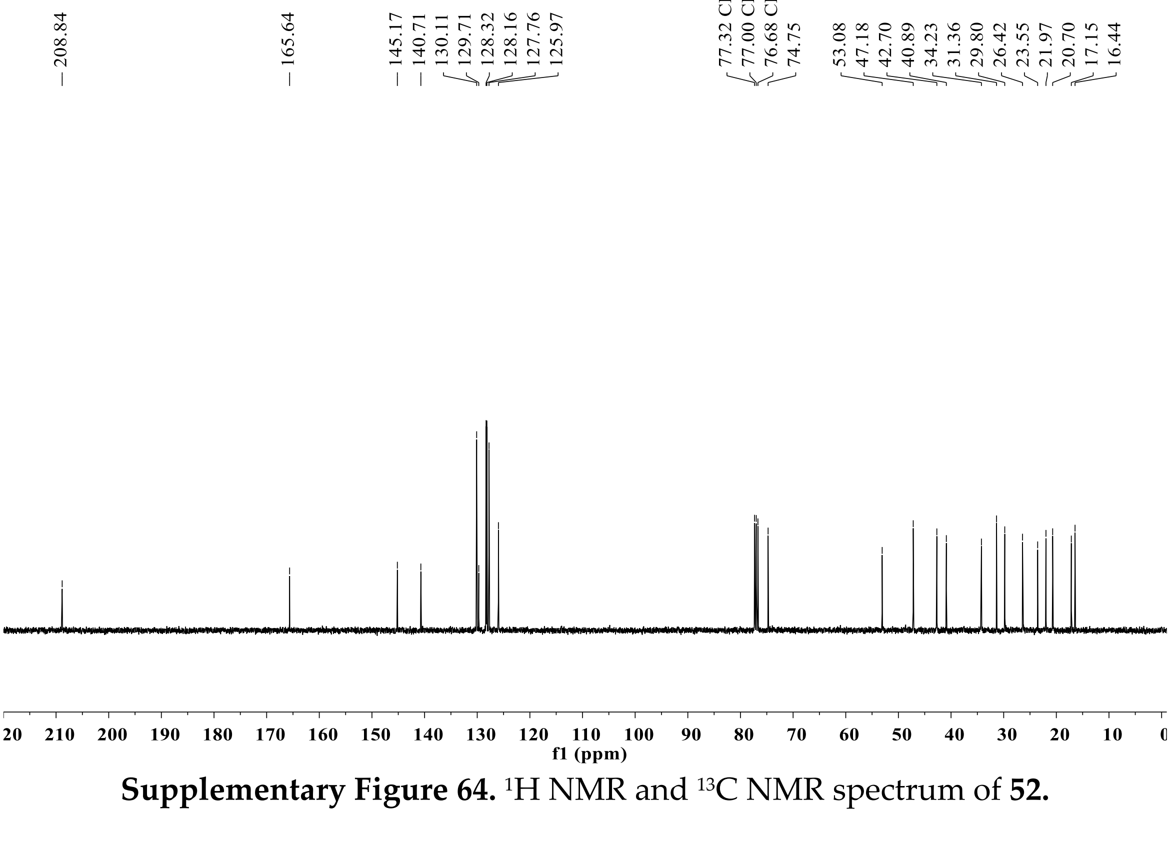

Supplementary Figure 64. ^{1}H NMR and ^{13}C NMR spectrum of **52.**

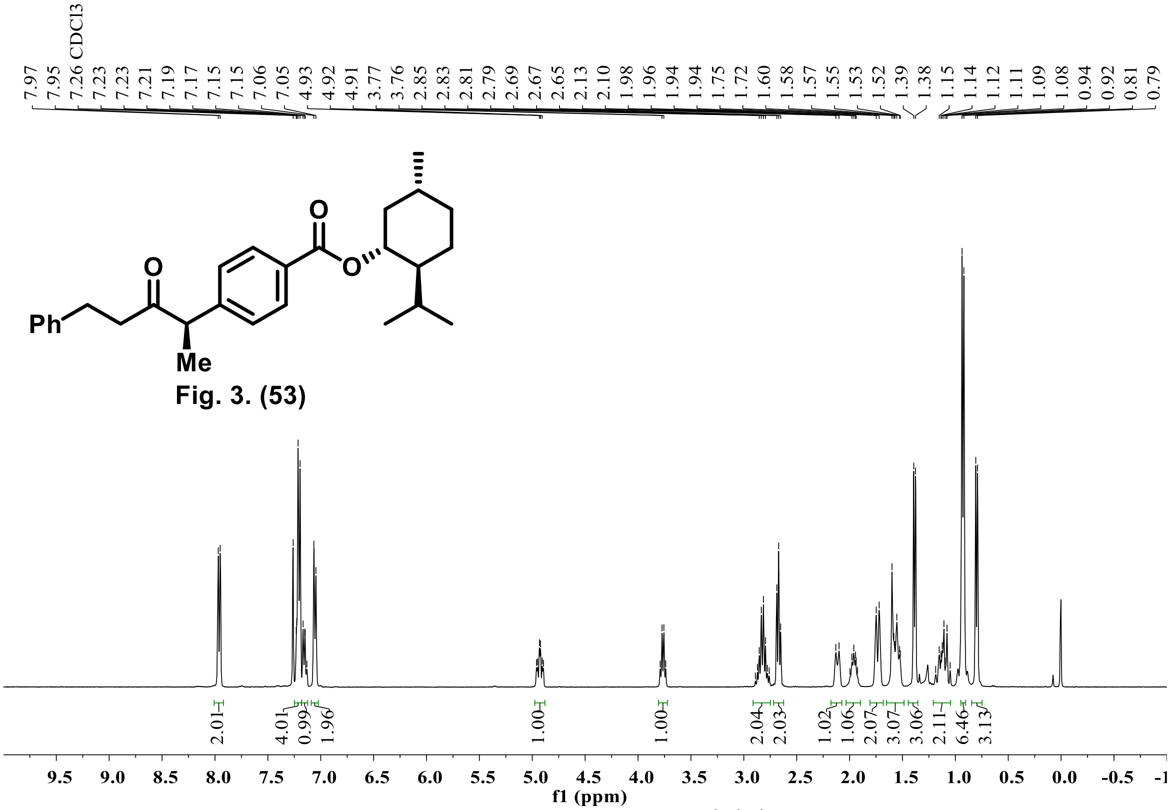

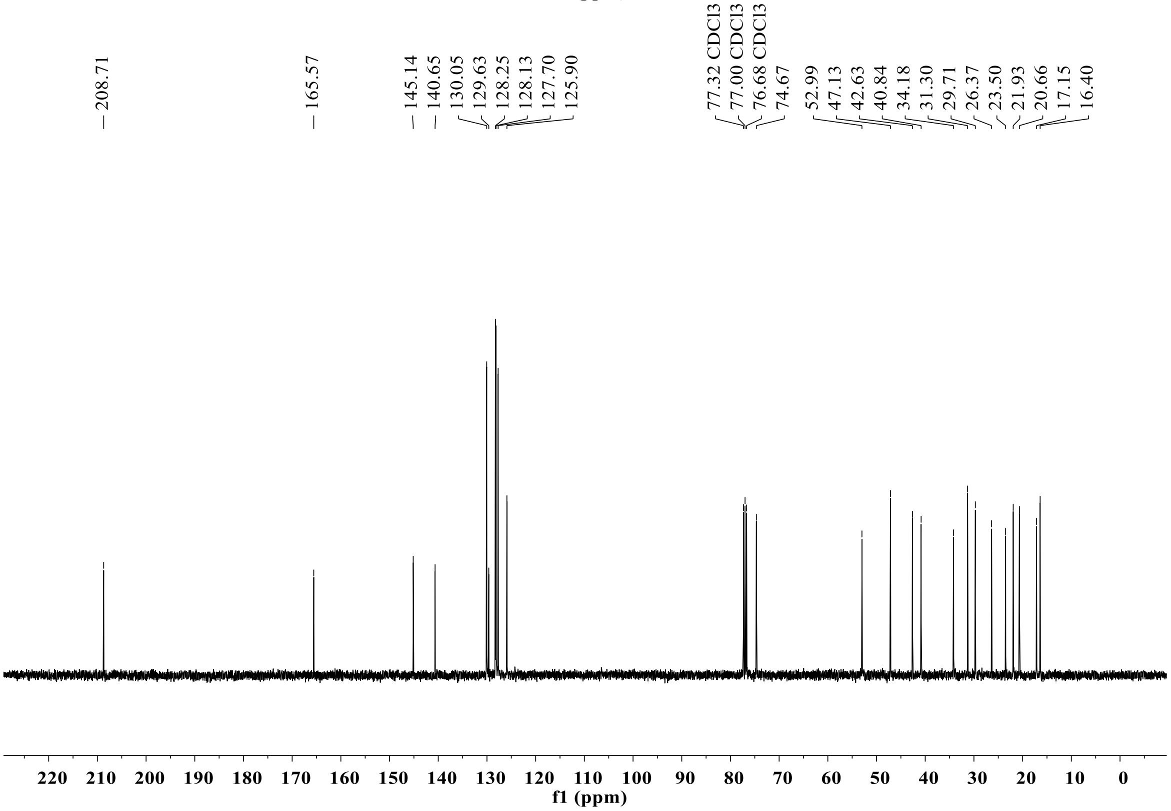

Supplementary Figure 65. ^{1}H NMR and ^{13}C NMR spectrum of **53.**

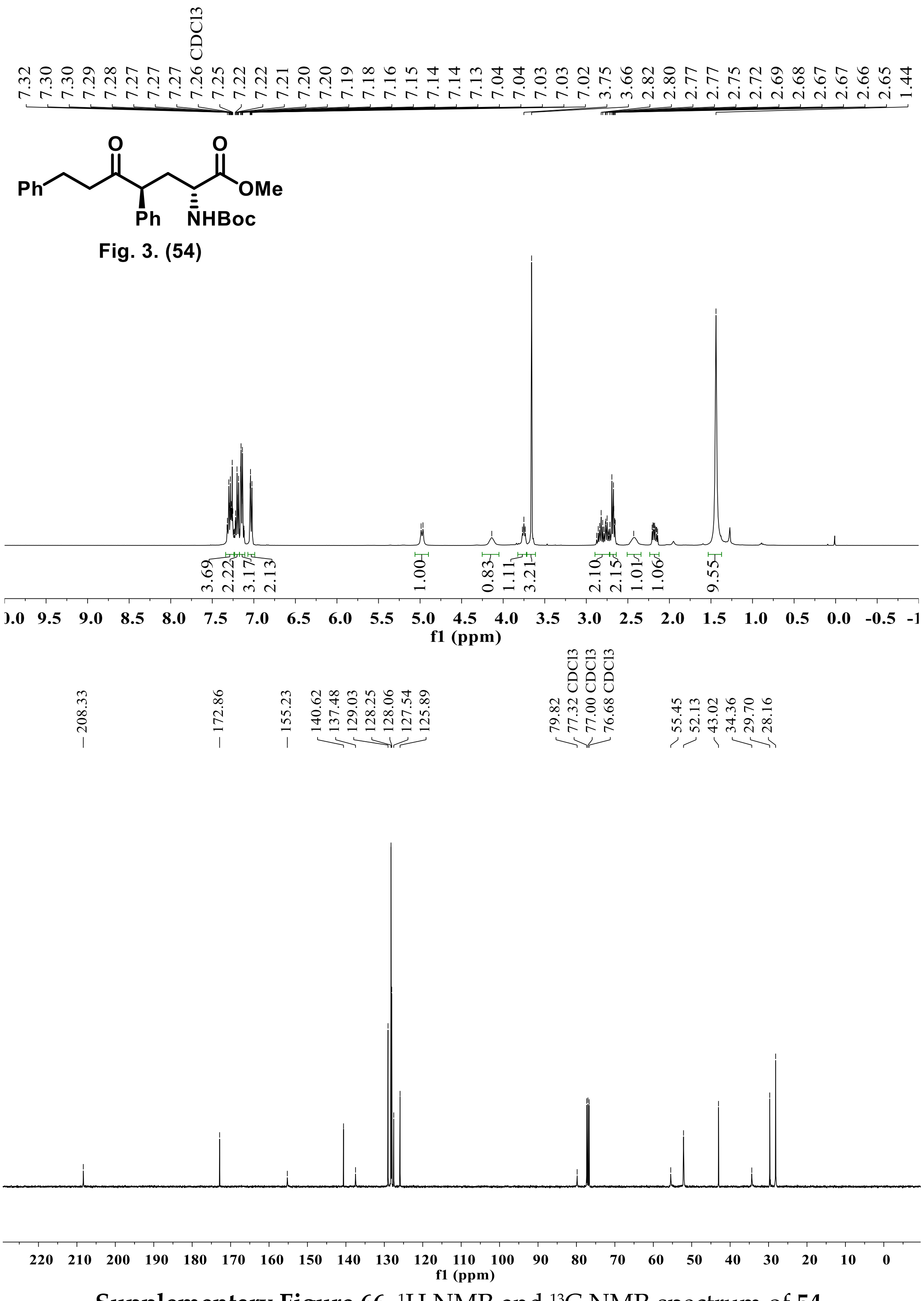

Supplementary Figure 66. ^{1}H NMR and ^{13}C NMR spectrum of **54.**

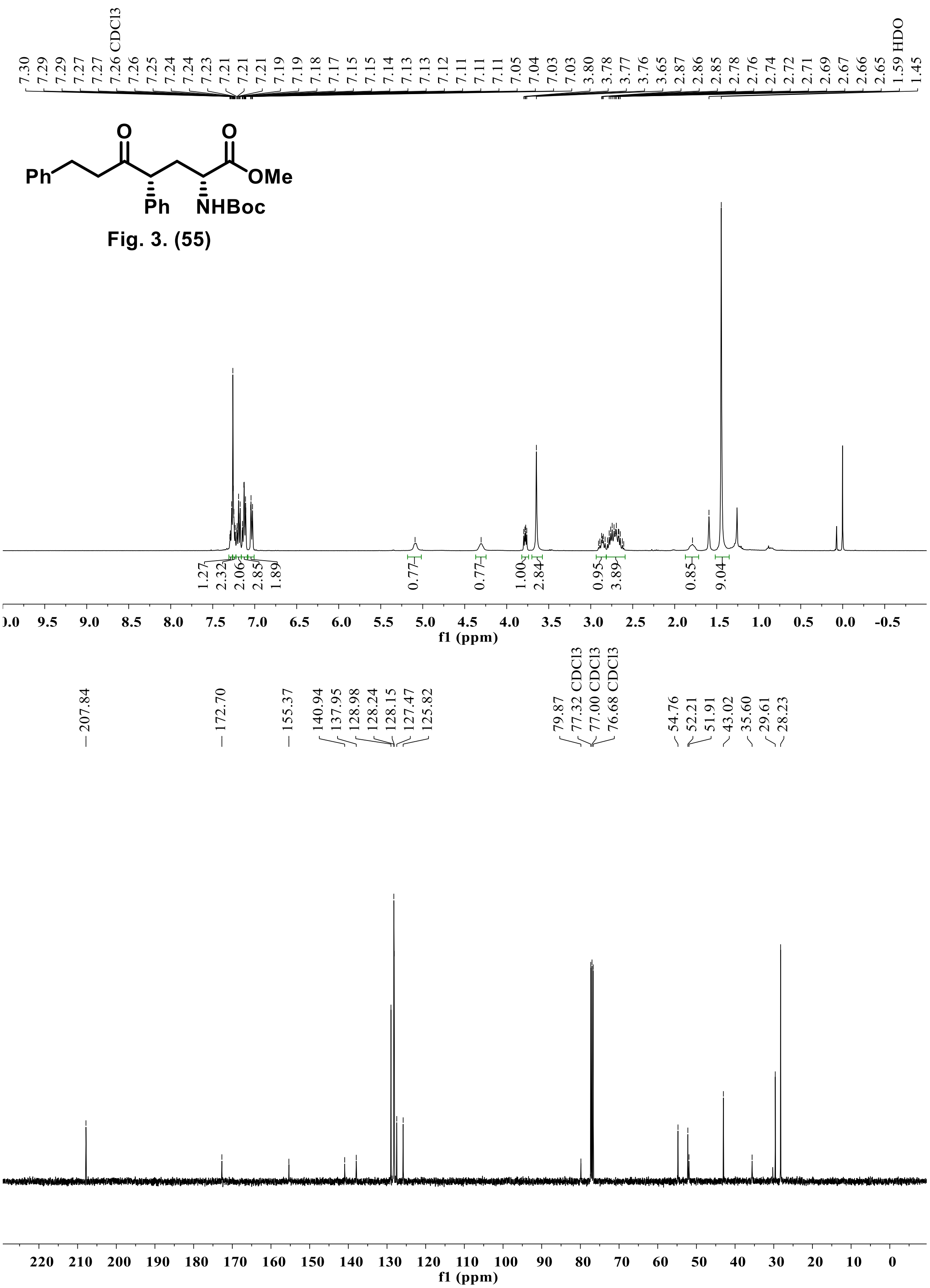

Supplementary Figure 67. ^{1}H NMR and ^{13}C NMR spectrum of **55.**

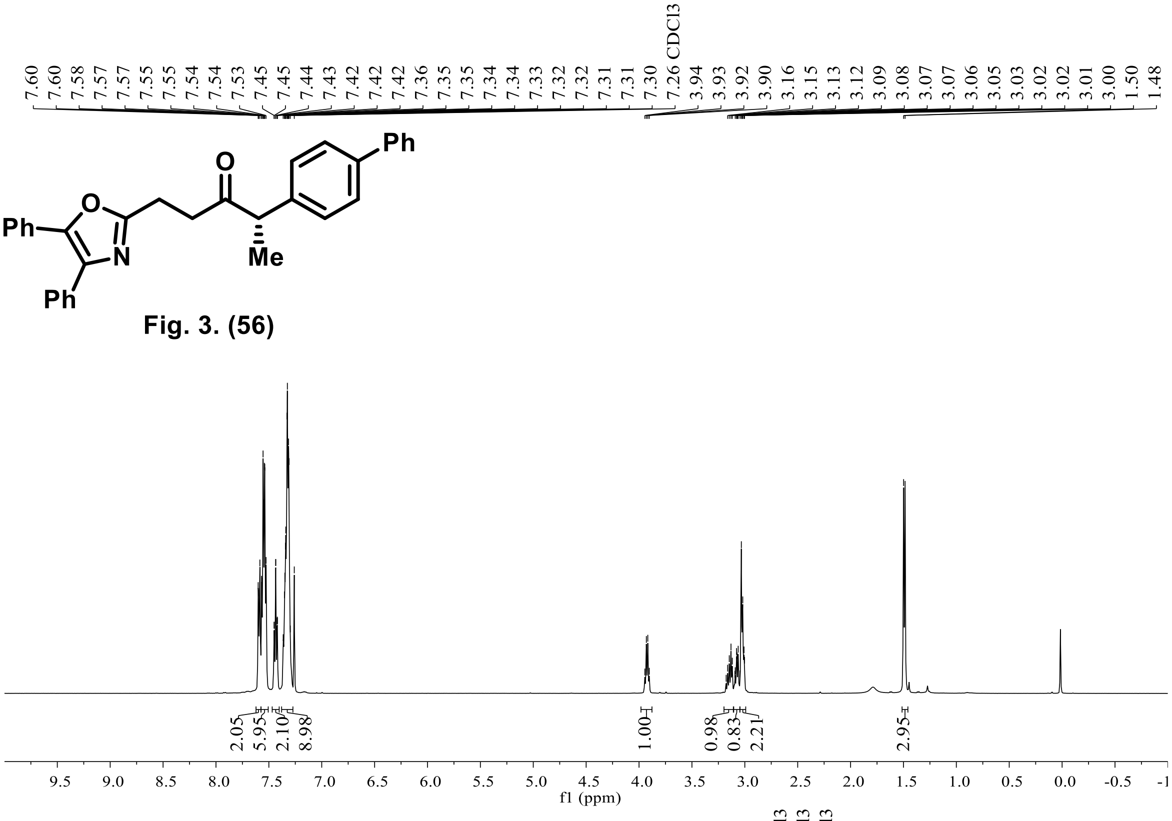

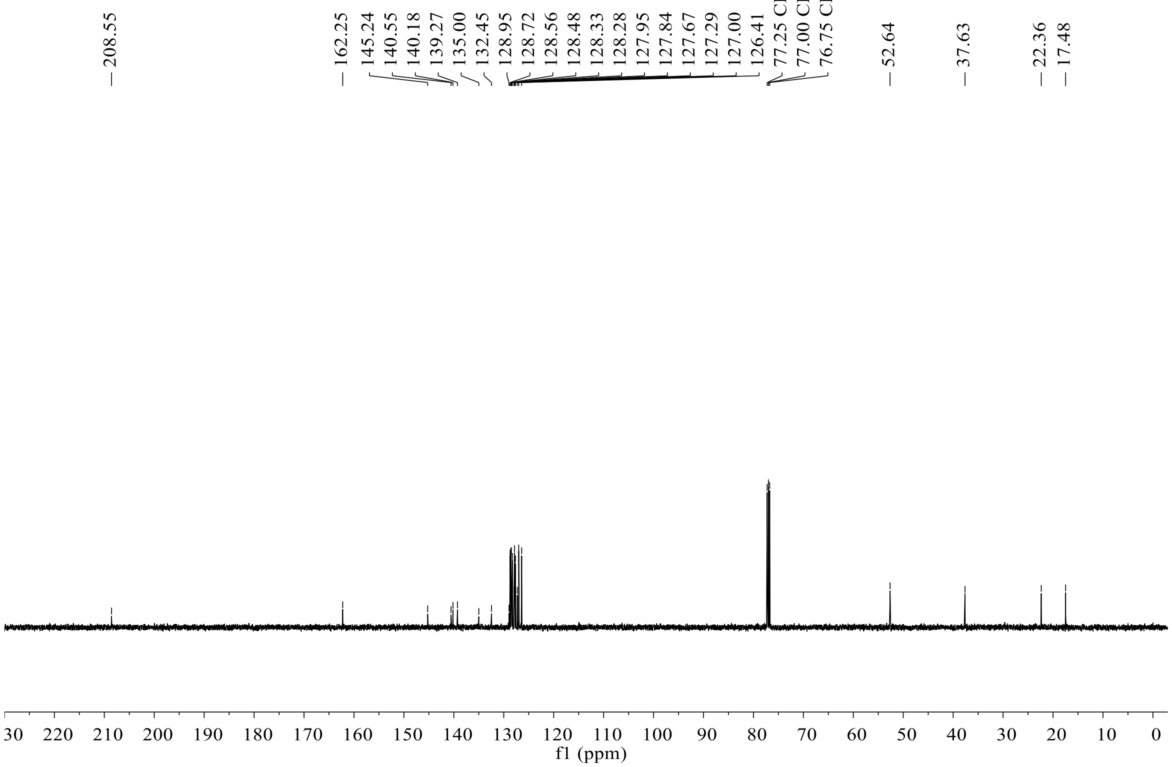

Supplementary Figure 68. ^{1}H NMR and ^{13}C NMR spectrum of **56.**

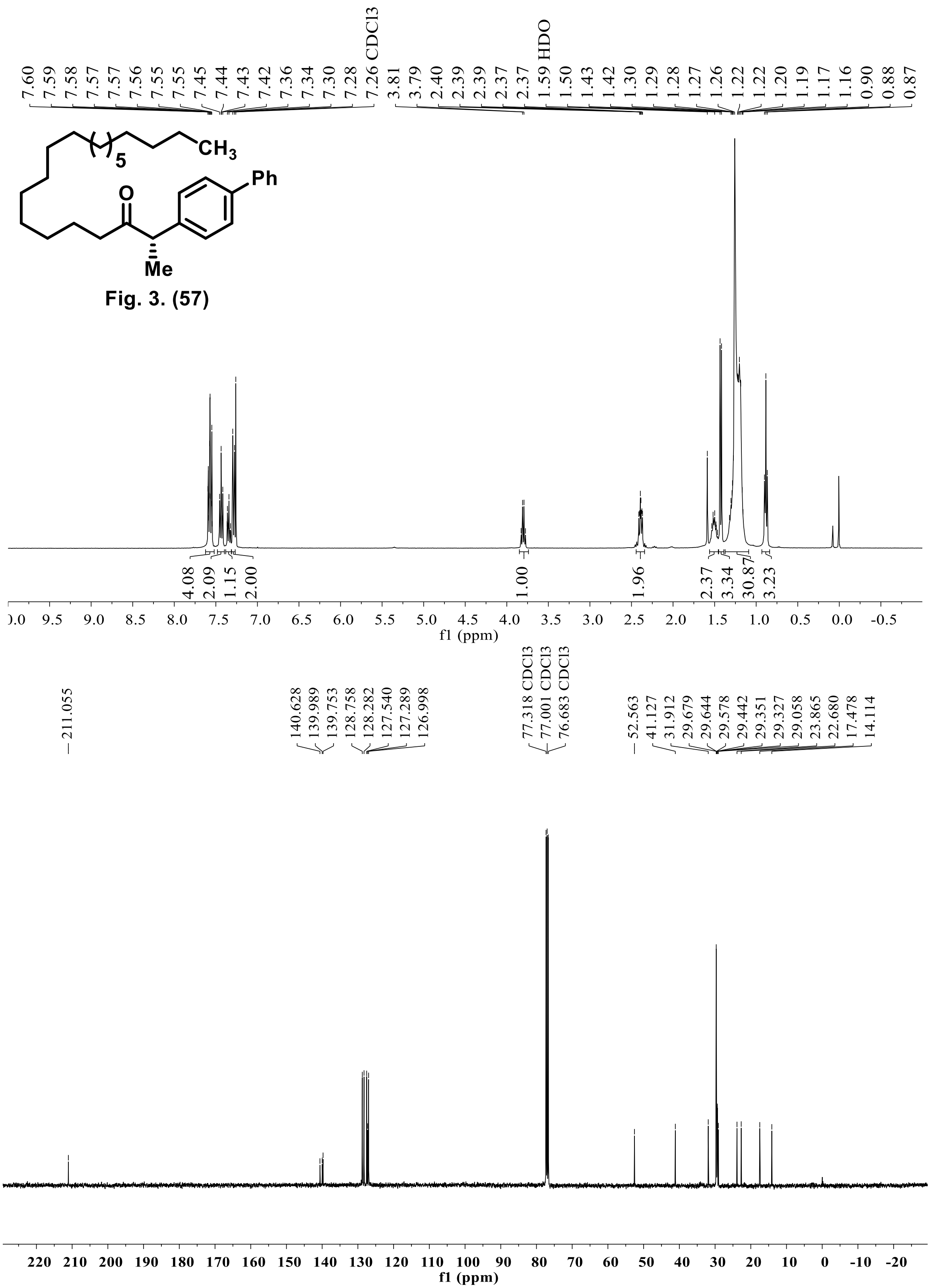

Supplementary Figure 69. ^{1}H NMR and ^{13}C NMR spectrum of **57.**

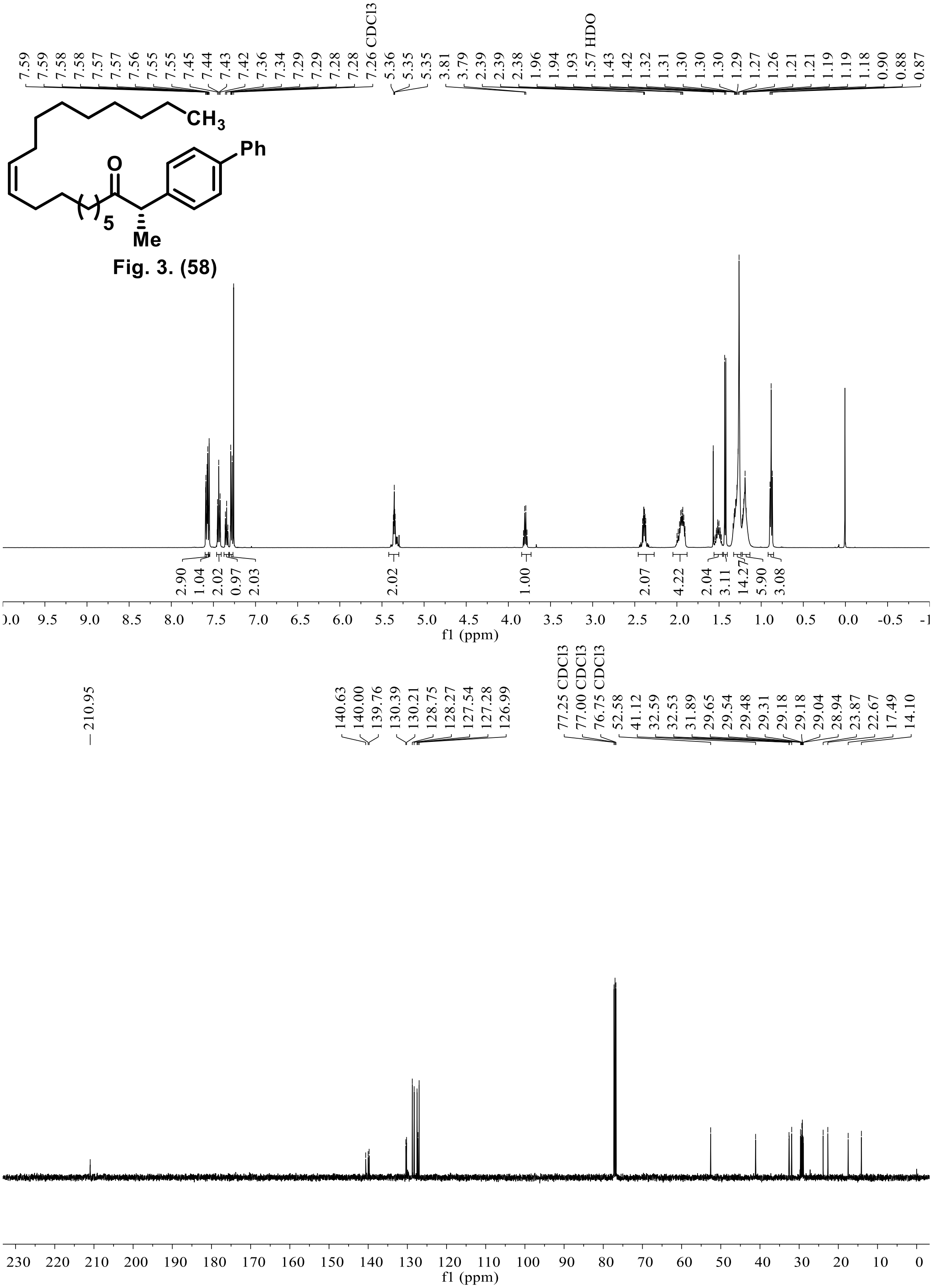

Supplementary Figure 70. ^{1}H NMR and ^{13}C NMR spectrum of **58.**

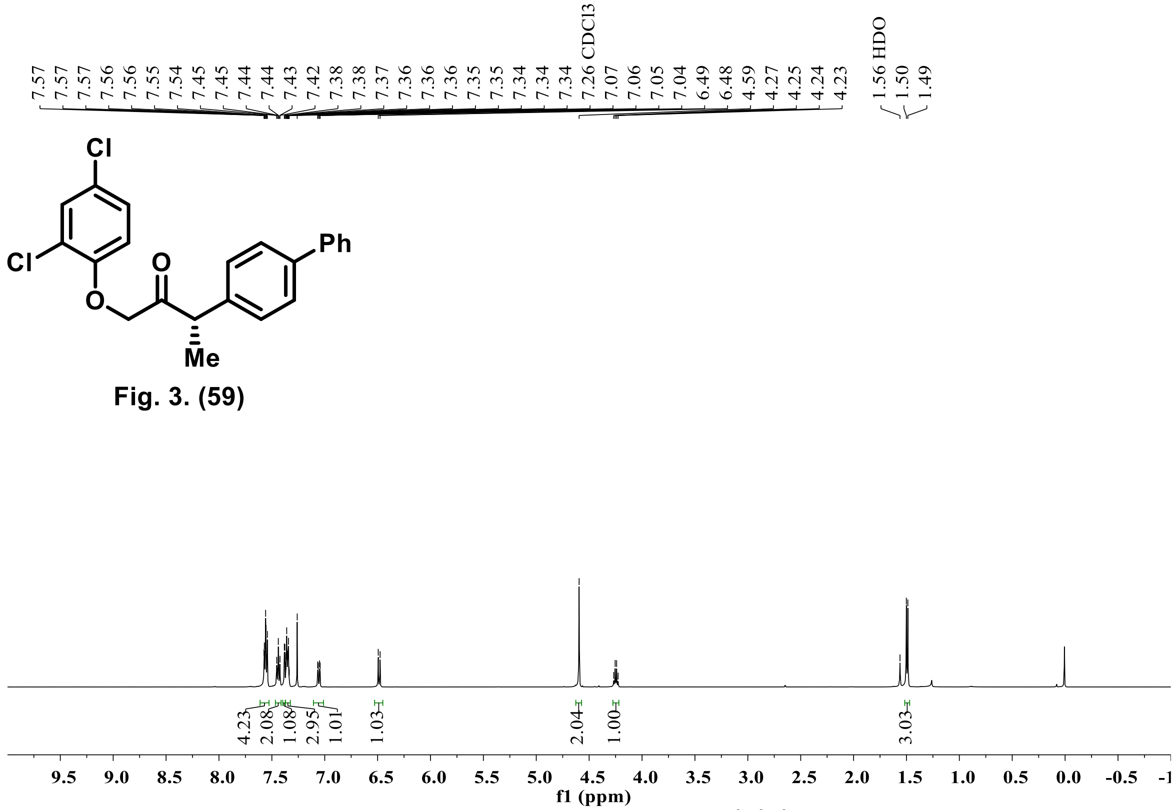

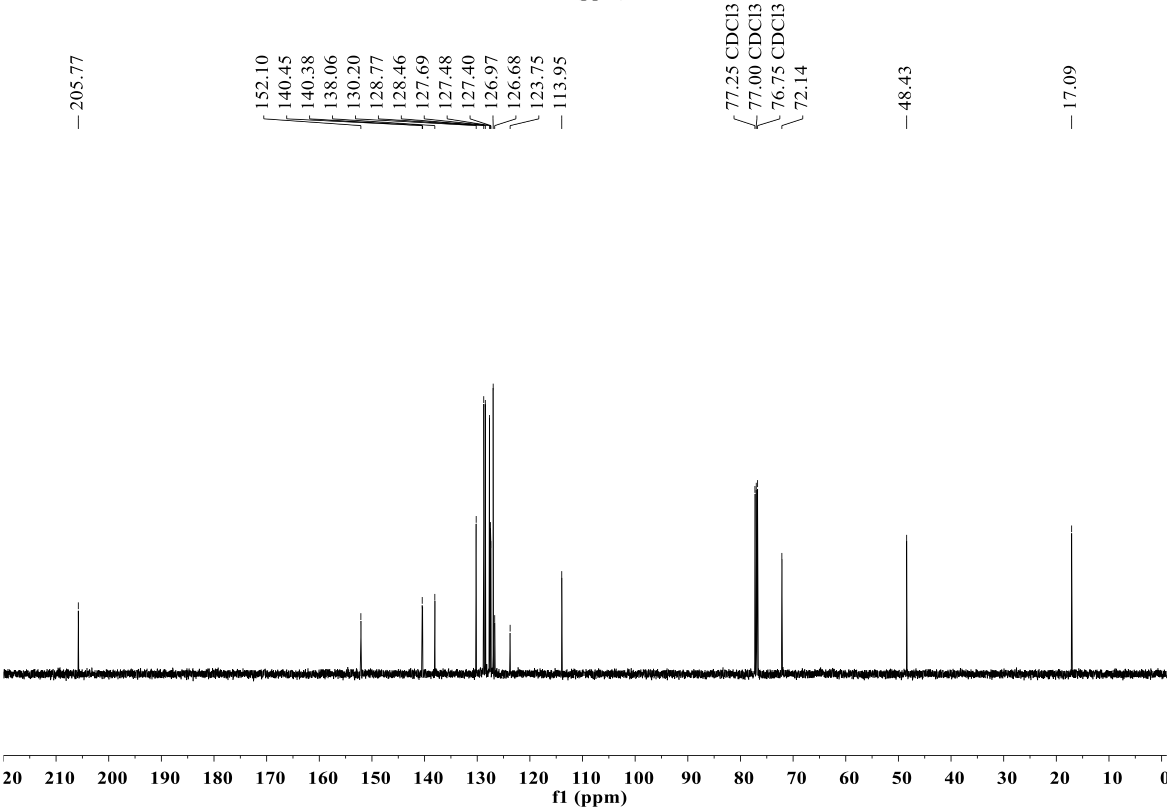

Supplementary Figure 71. ^{1}H NMR and ^{13}C NMR spectrum of **59.**

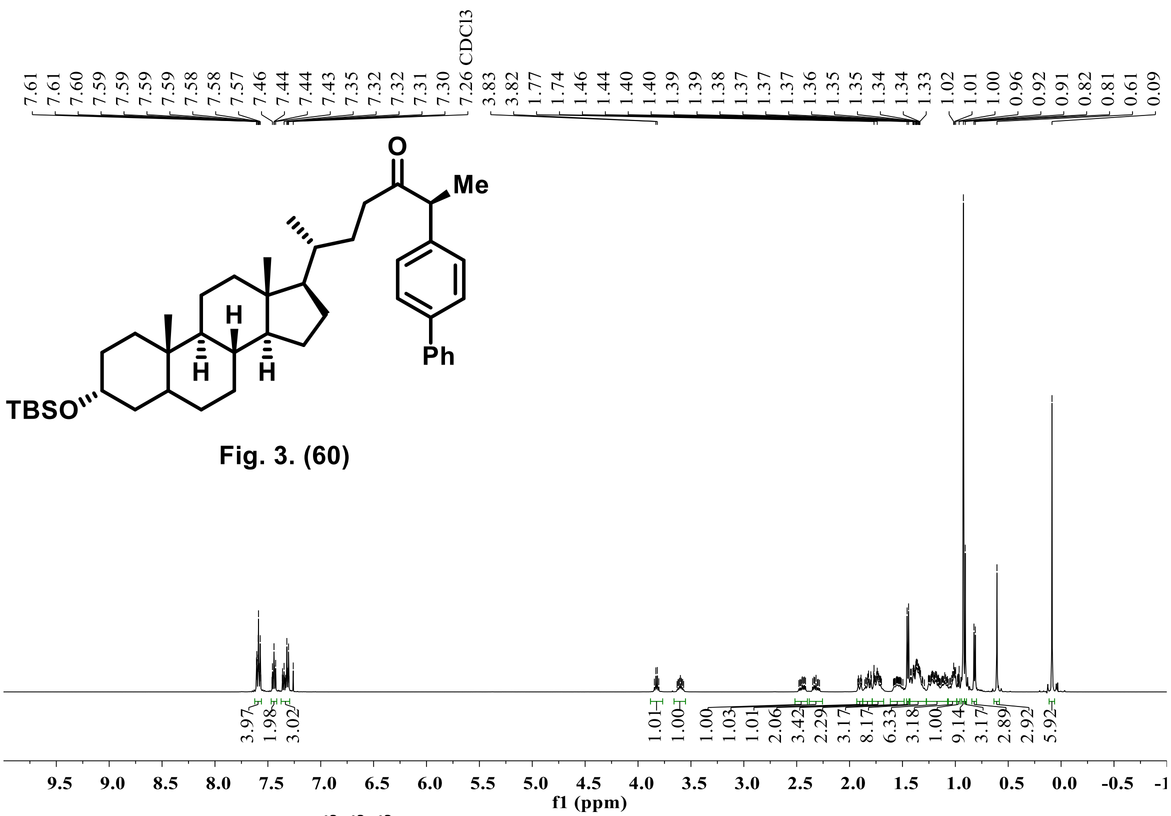

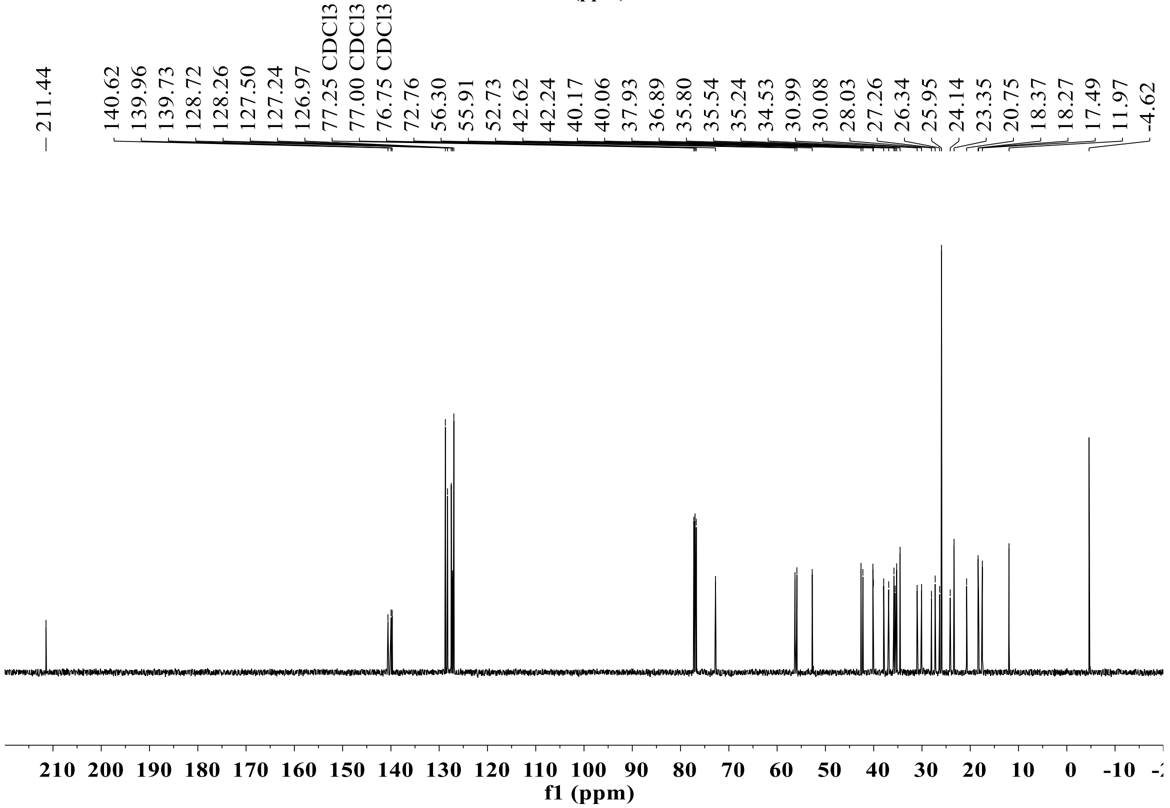

Supplementary Figure 72. ^{1}H NMR and ^{13}C NMR spectrum of **60.**

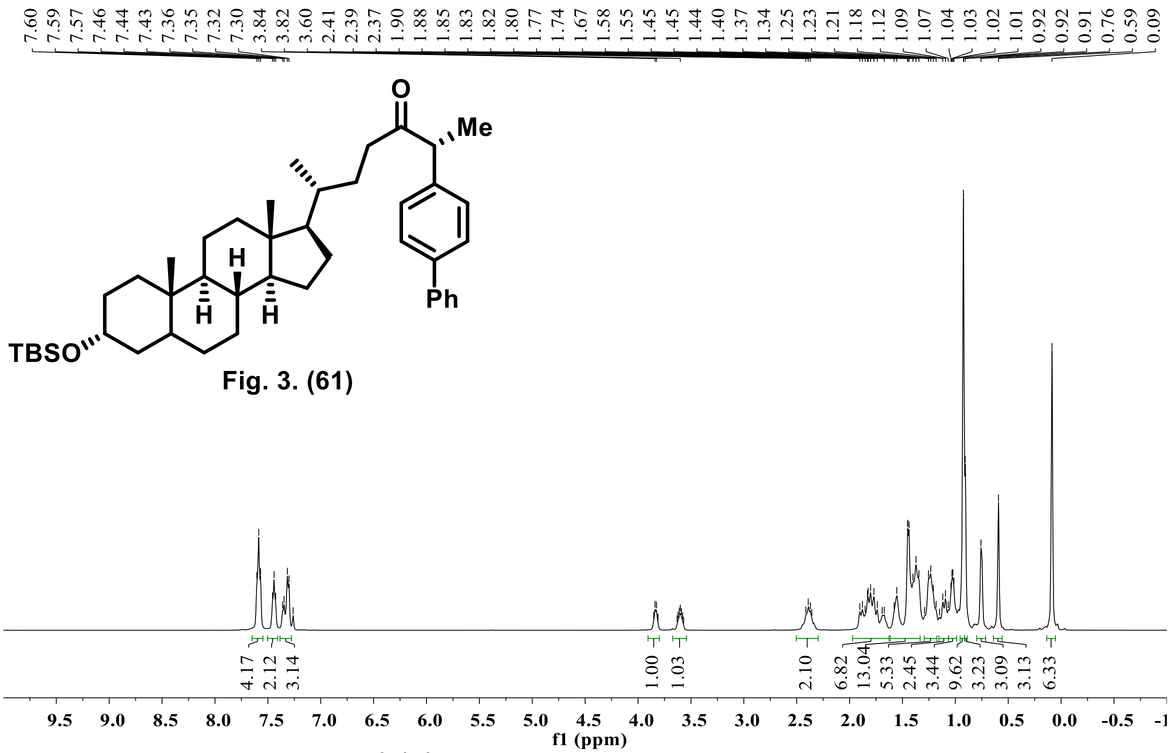

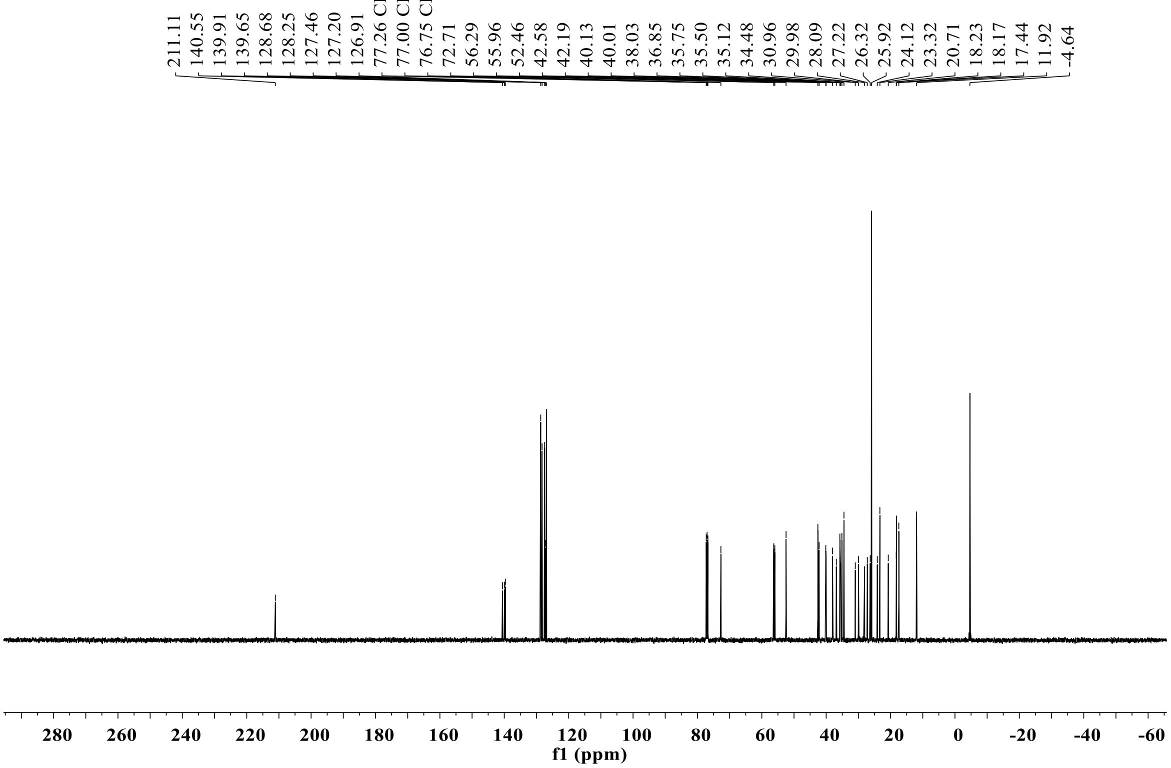

Supplementary Figure 73. ^{1}H NMR and ^{13}C NMR spectrum of **61.**

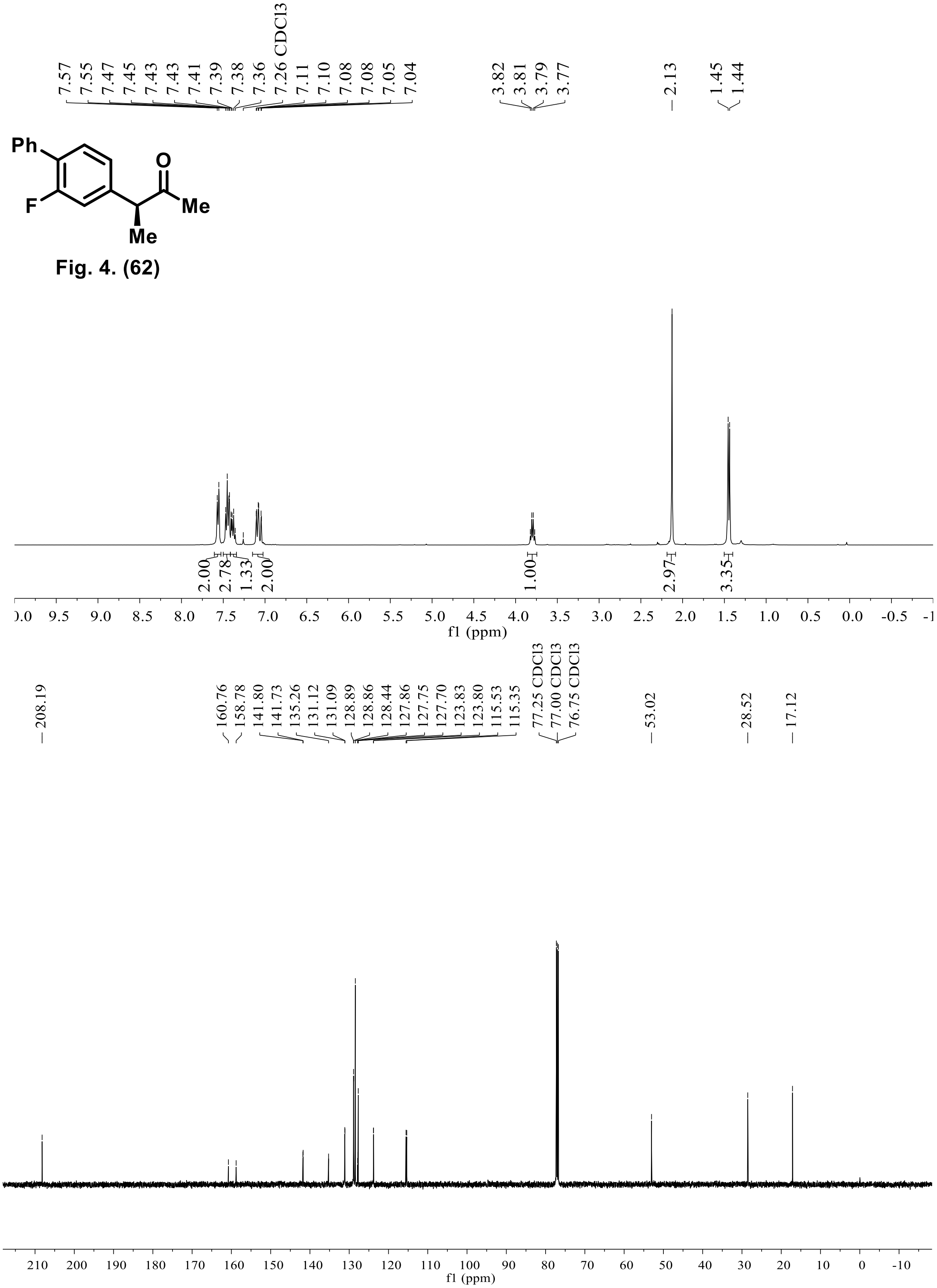

Supplementary Figure 74. ^{1}H NMR and ^{13}C NMR spectrum of **62**.

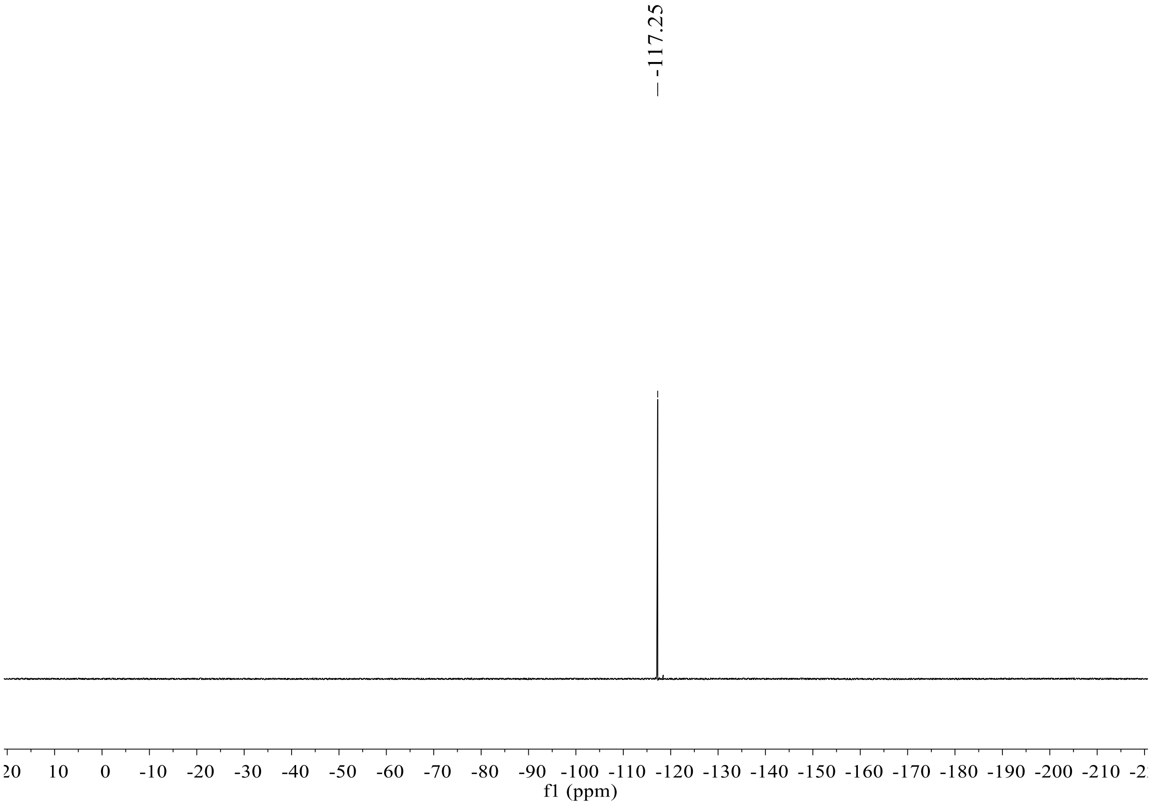

Supplementary Figure 75. ^{19}F NMR spectrum of **62**.

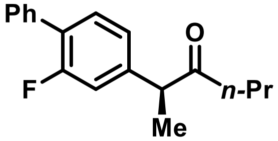

Fig. 4. (63)

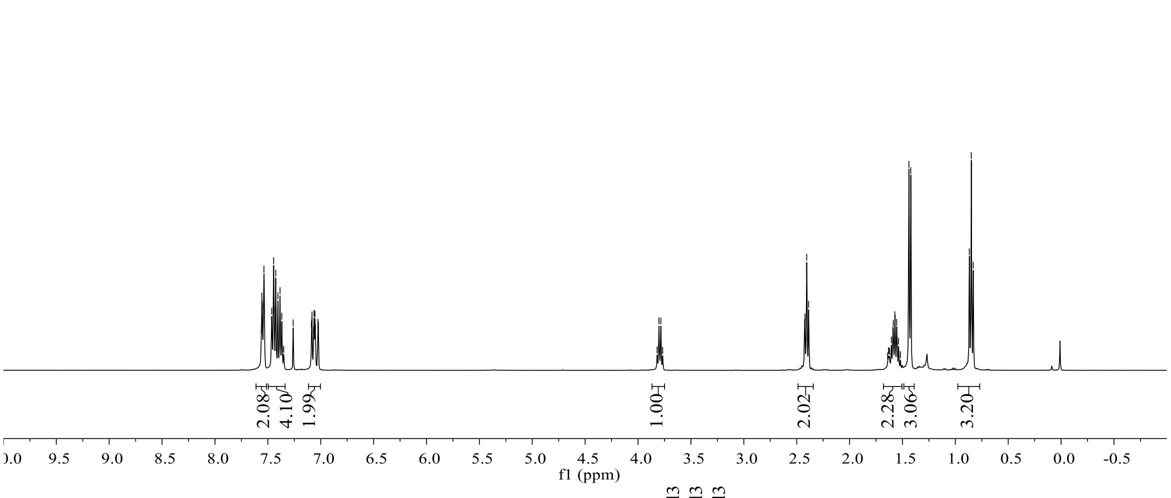

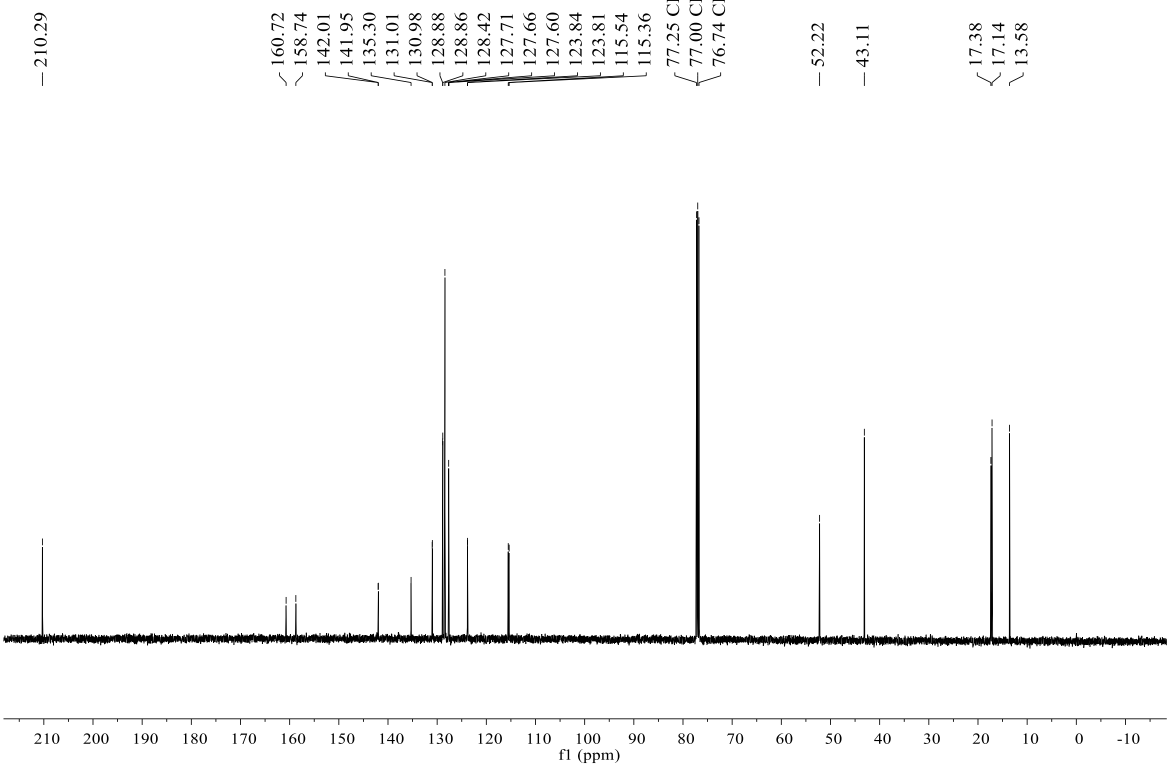

Supplementary Figure 76. ^{1}H NMR and ^{13}C NMR spectrum of **63**.

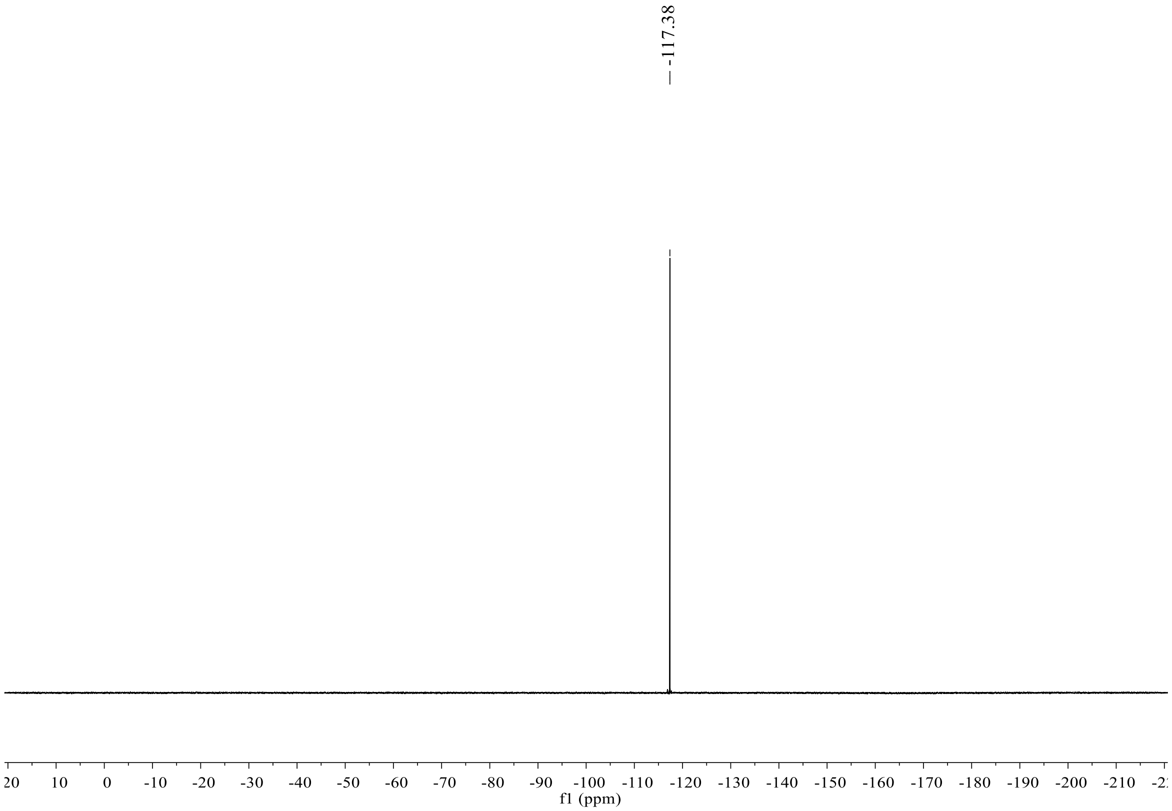

Supplementary Figure 77. ^{19}F NMR spectrum of **63**.

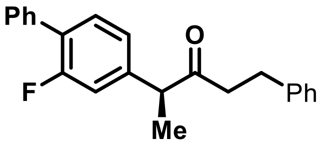

Fig. 4. (64)

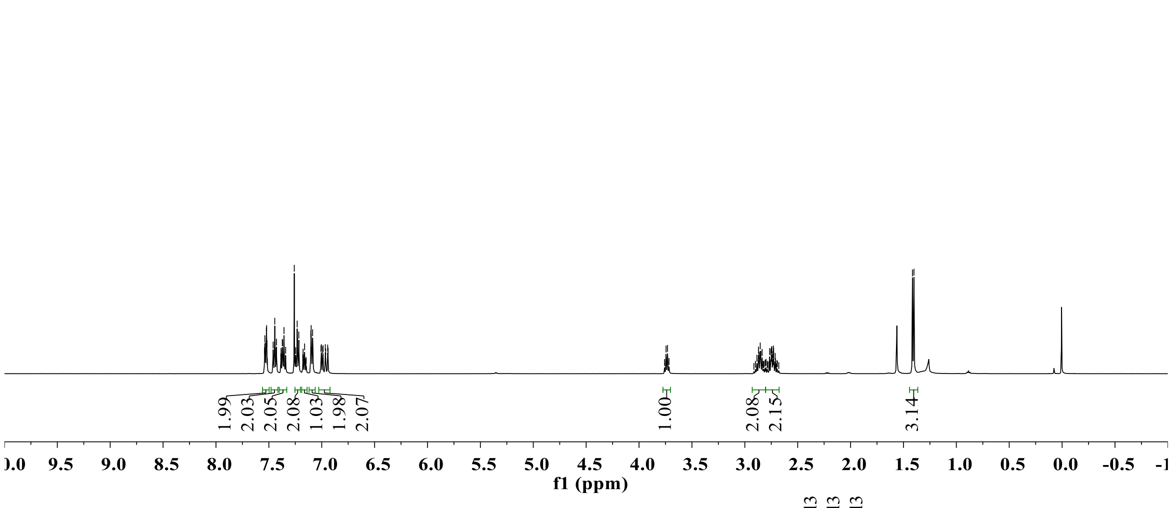

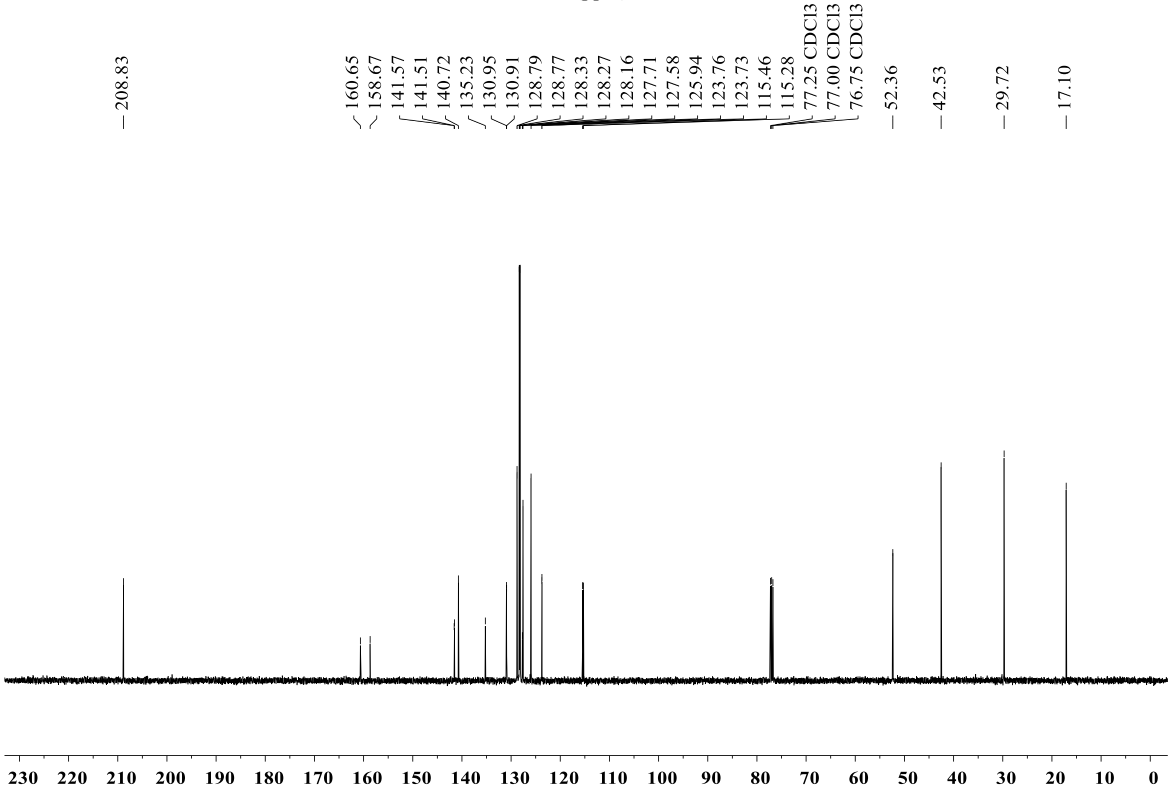

Supplementary Figure 78. ^{1}H NMR and ^{13}C NMR spectrum of **64**.

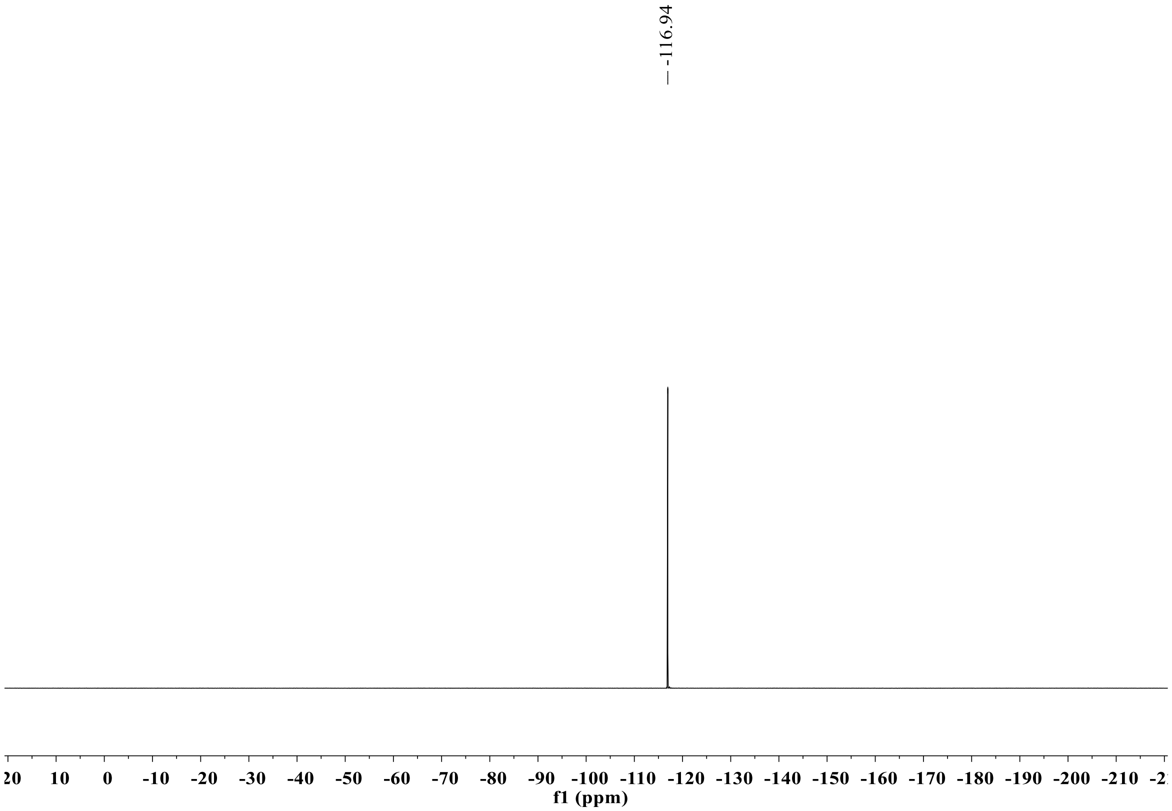

Supplementary Figure 79. ^{19}F NMR spectrum of **64**.

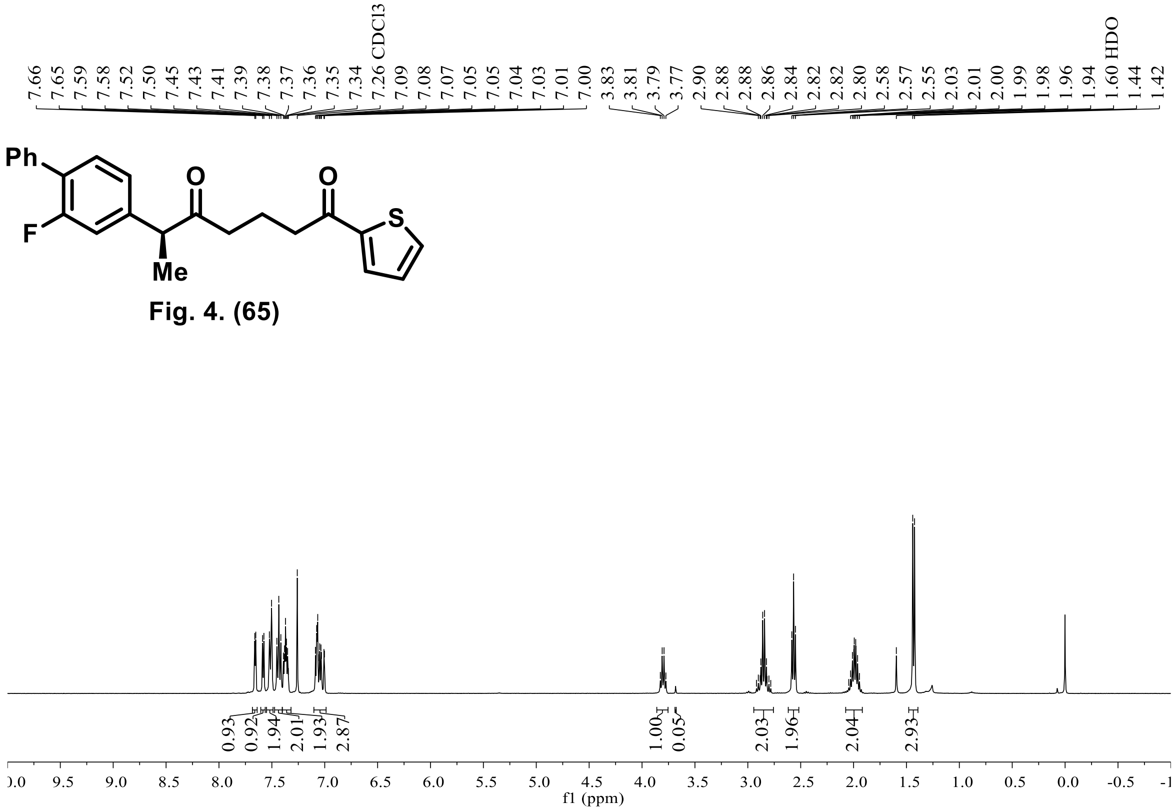

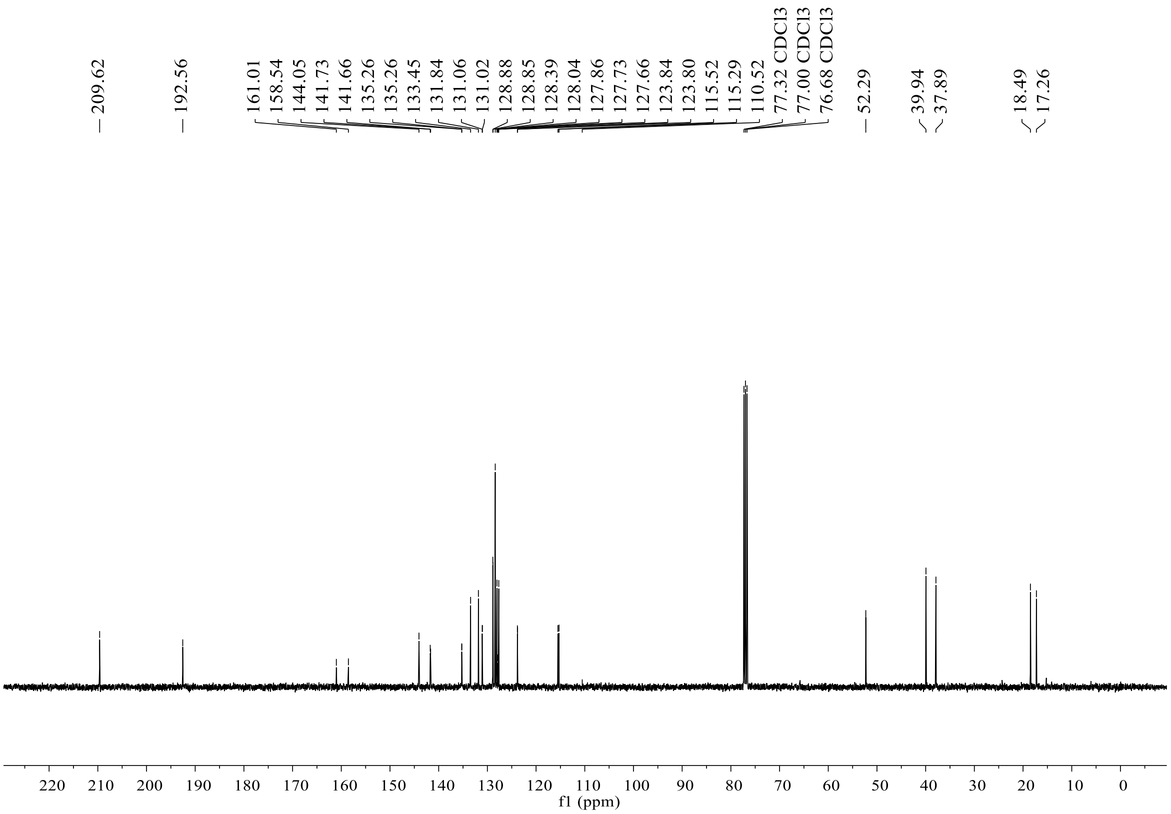

Supplementary Figure 80. ^{1}H NMR and ^{13}C NMR spectrum of **65**.

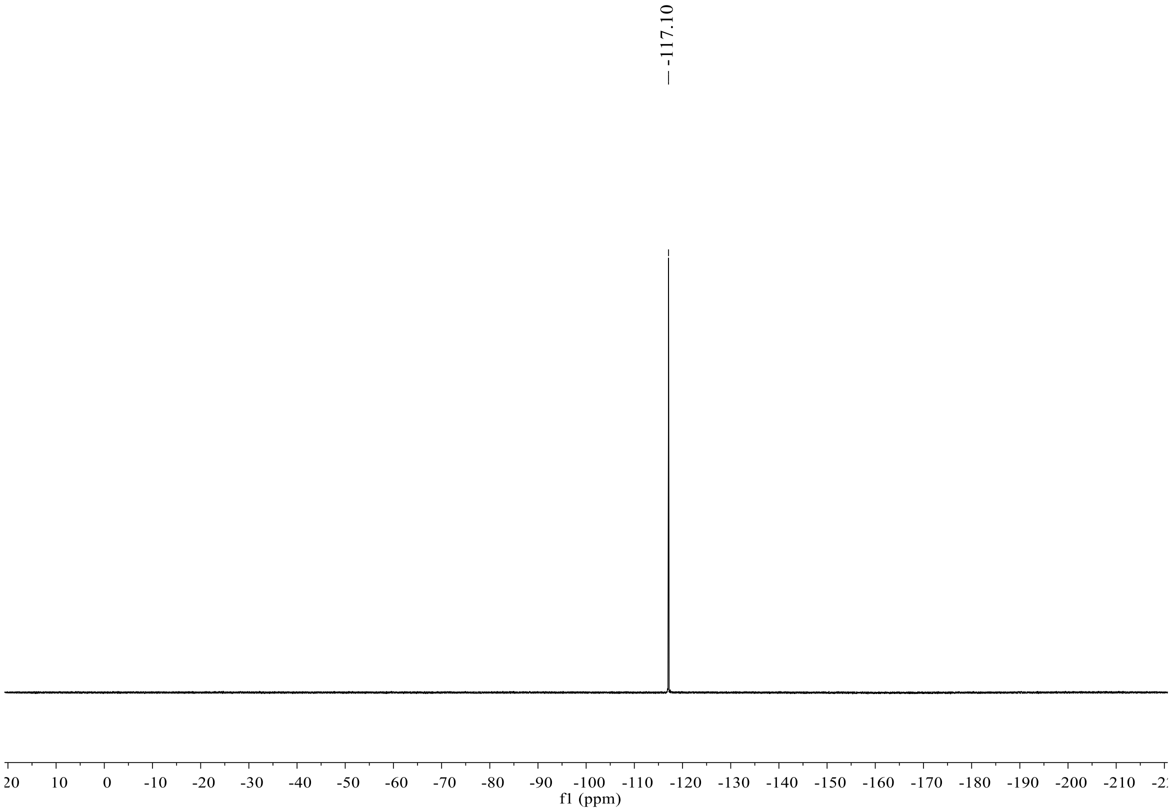

Supplementary Figure 81. ^{19}F NMR spectrum of **65**.

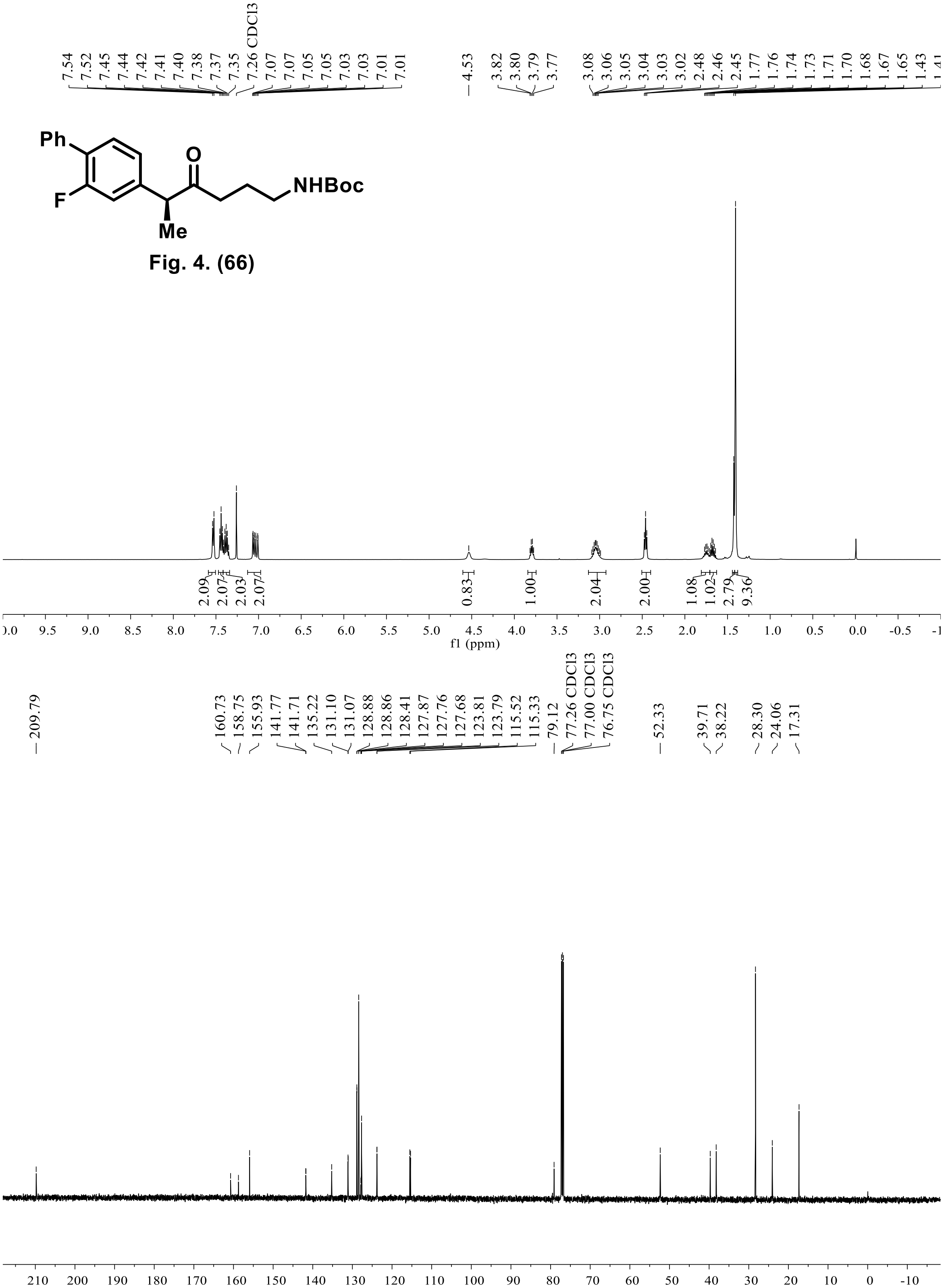

Supplementary Figure 82. ^{1}H NMR and ^{13}C NMR spectrum of **66**.

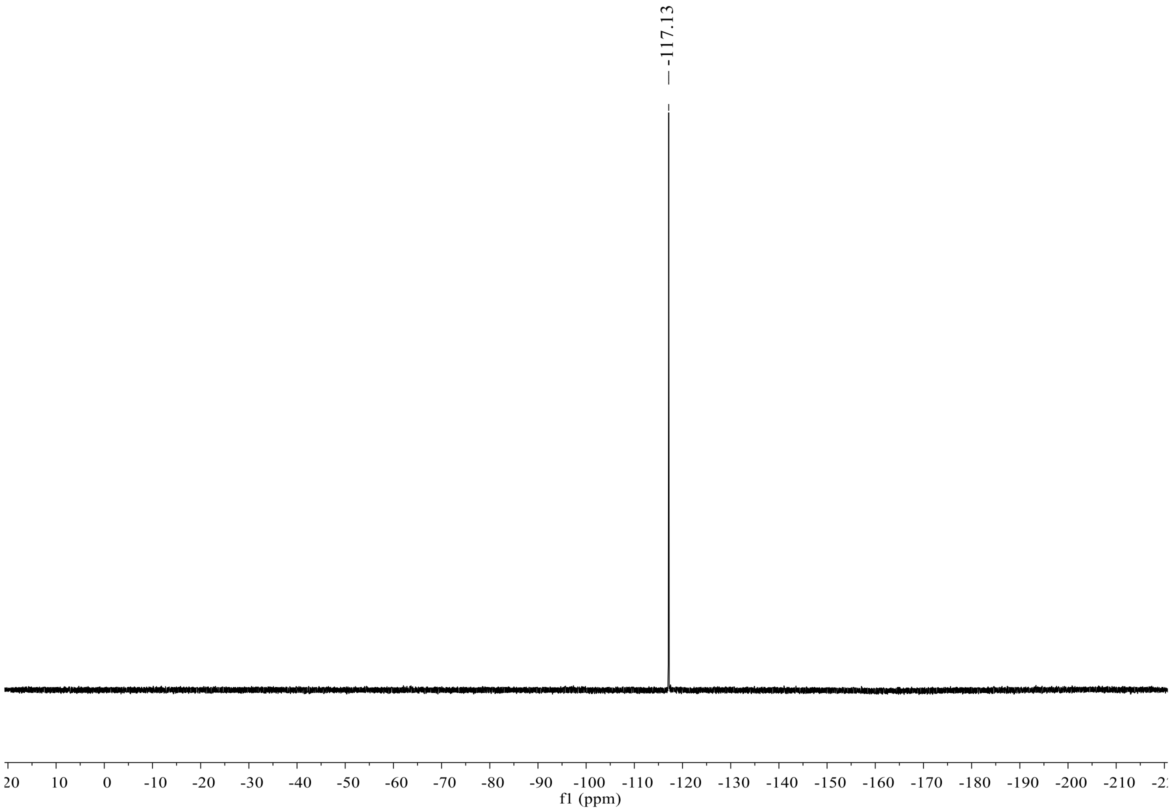

Supplementary Figure 83. ^{19}F NMR spectrum of **66.**

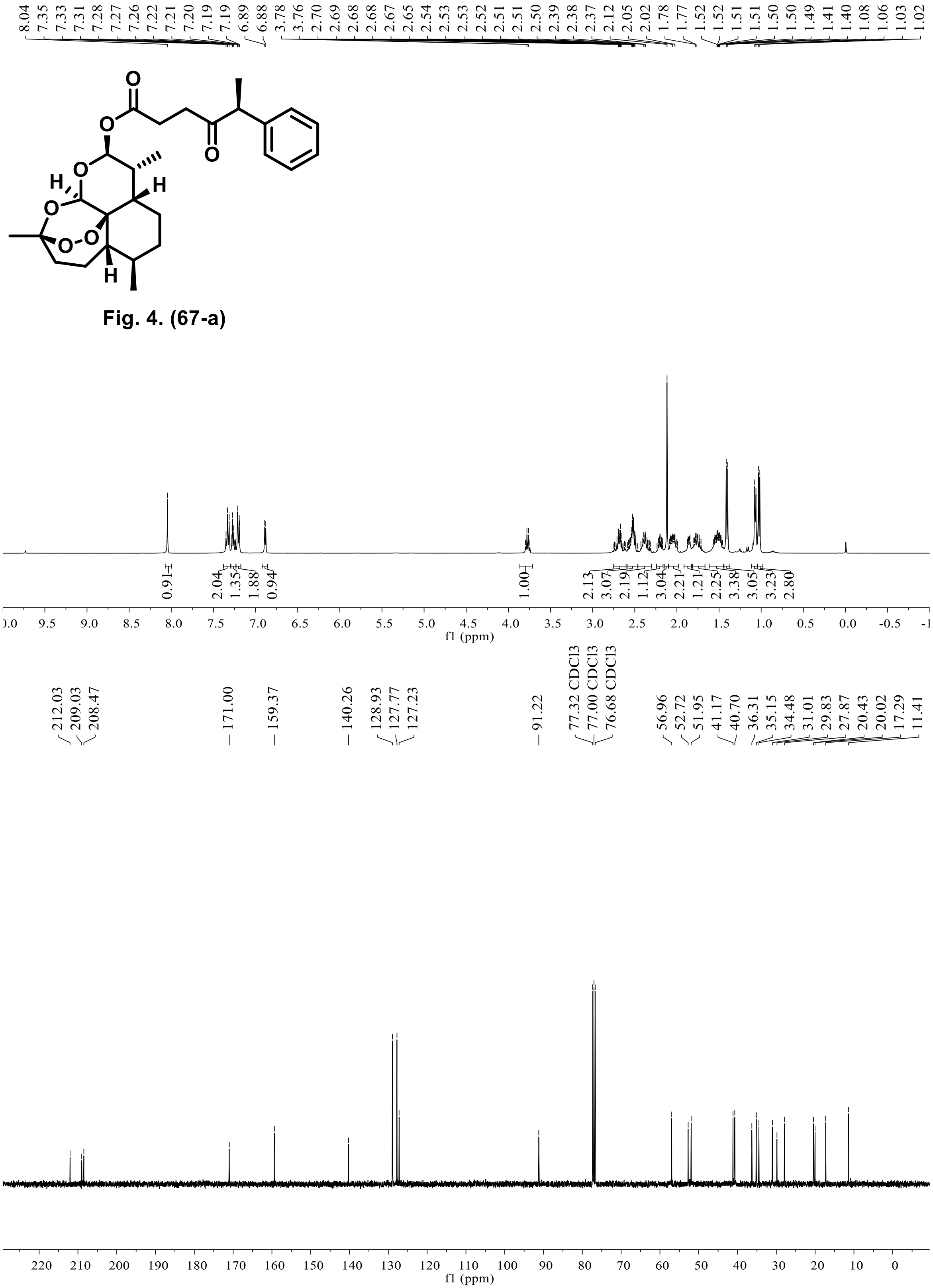

Supplementary Figure 84. ^{1}H NMR and ^{13}C NMR spectrum of **67-a.**

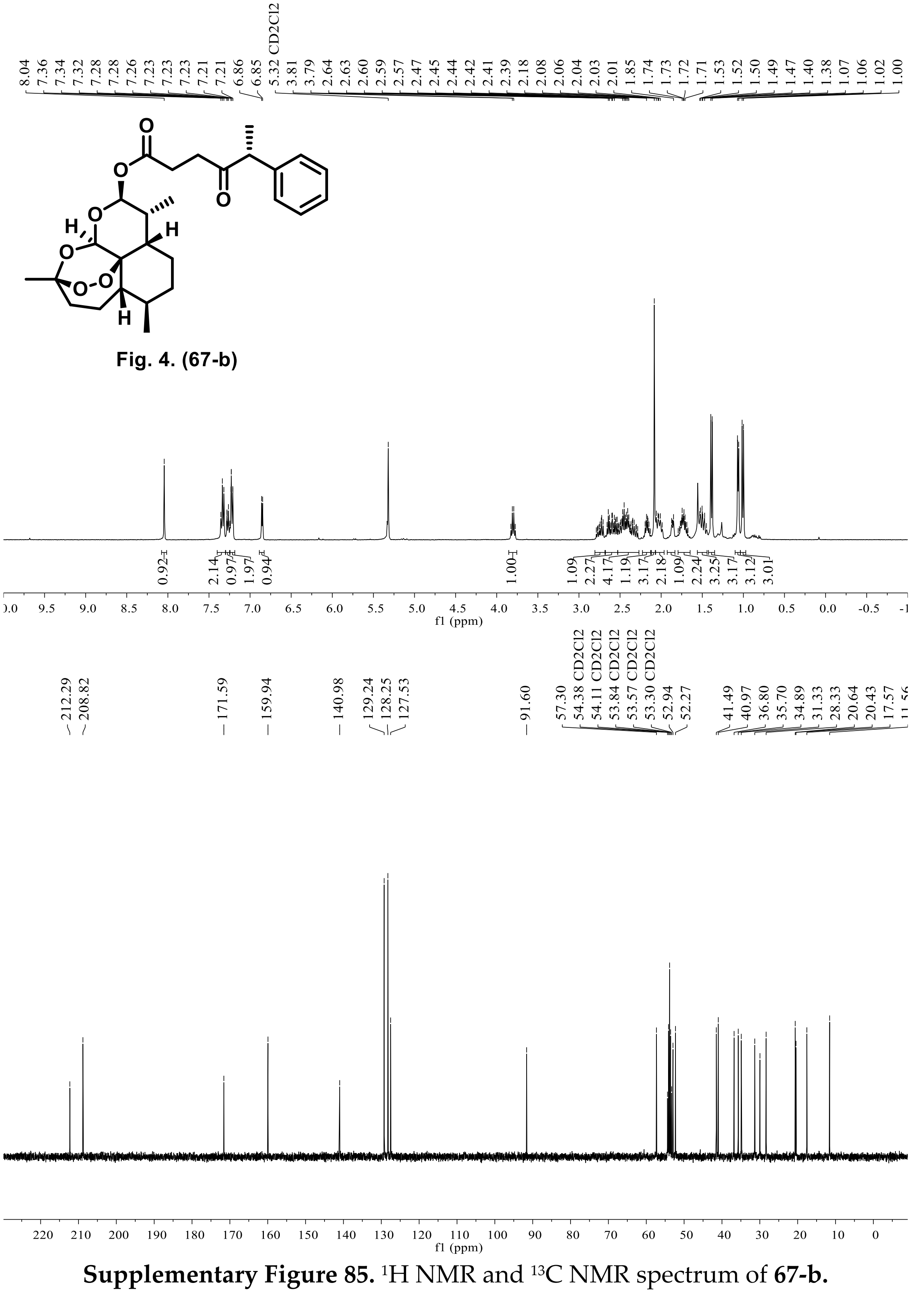

Supplementary Figure 85. ^{1}H NMR and ^{13}C NMR spectrum of **67-b.**

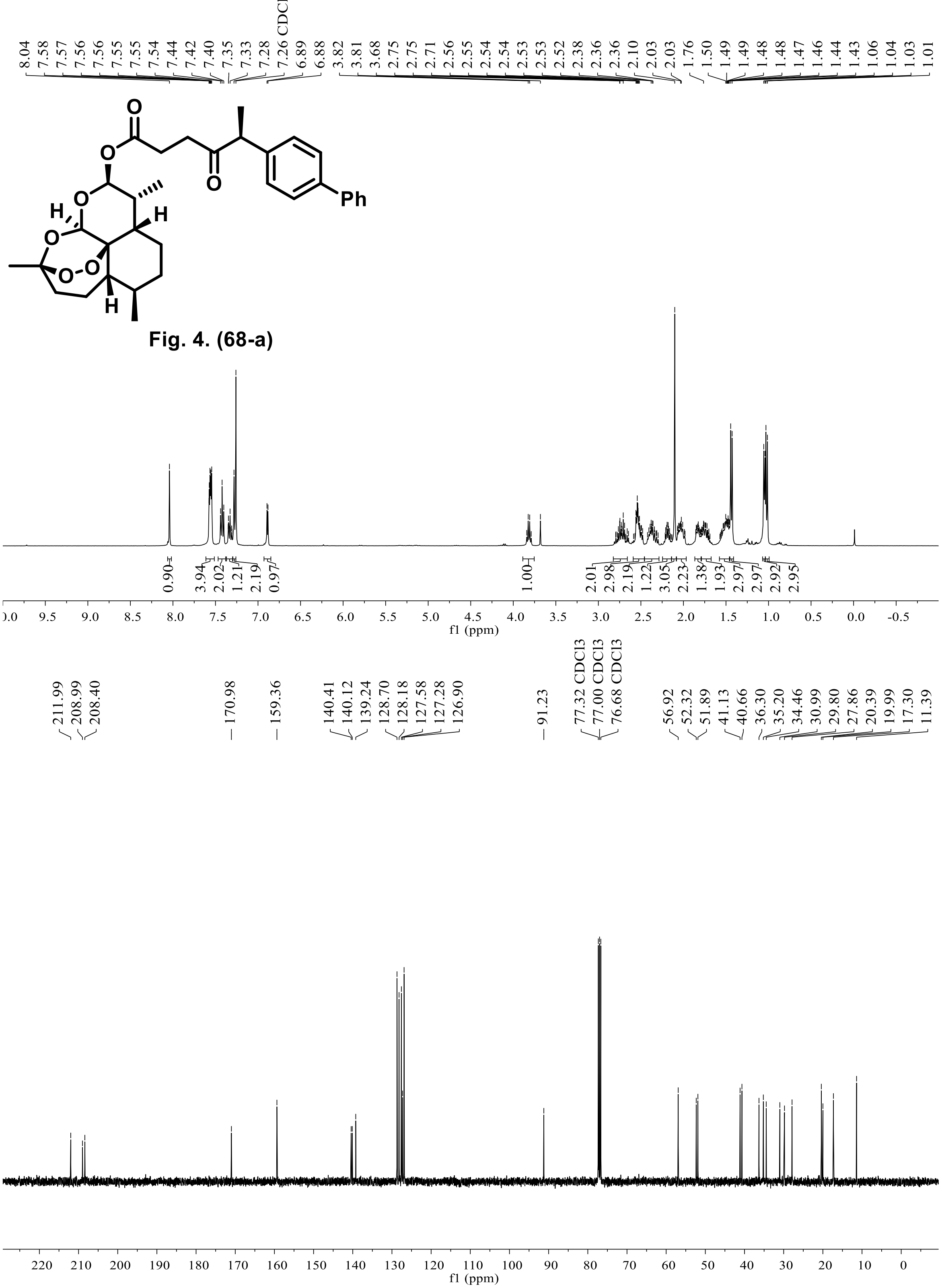

Supplementary Figure 86. ^{1}H NMR and ^{13}C NMR spectrum of **68-a.**

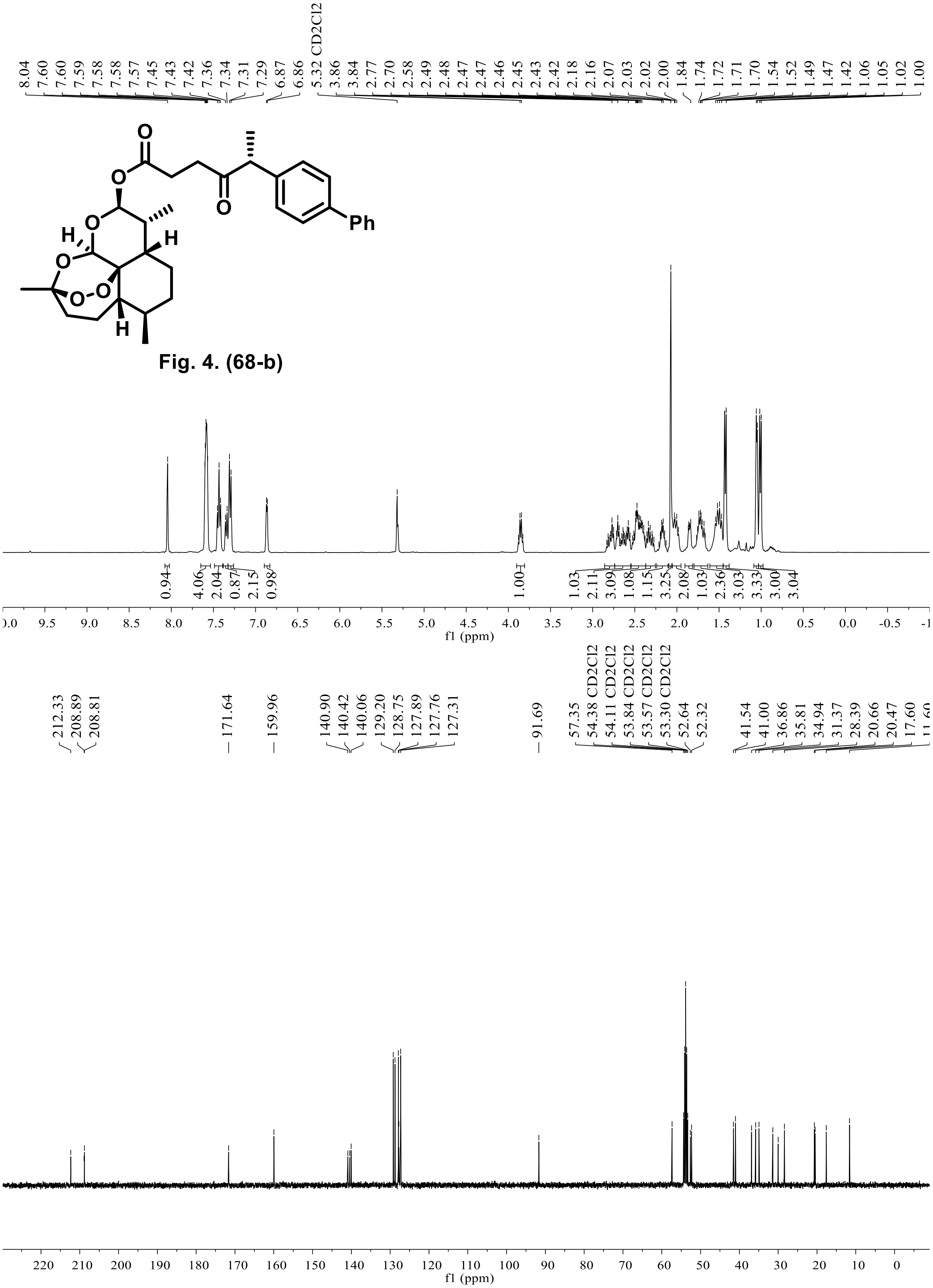

Supplementary Figure 87. ^{1}H NMR and ^{13}C NMR spectrum of **68-b.**

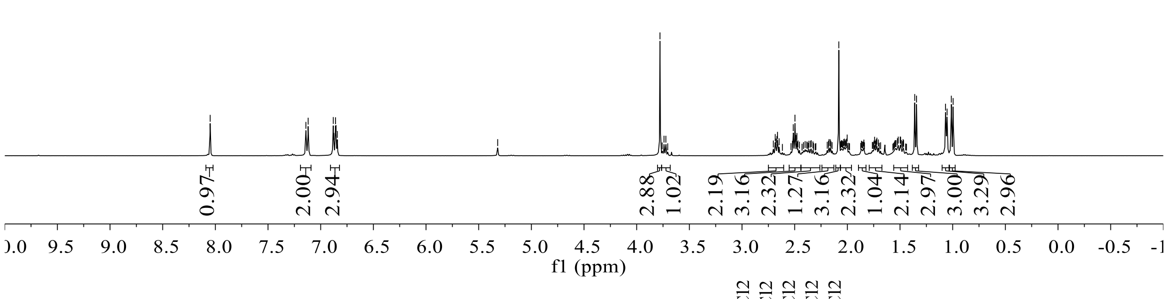

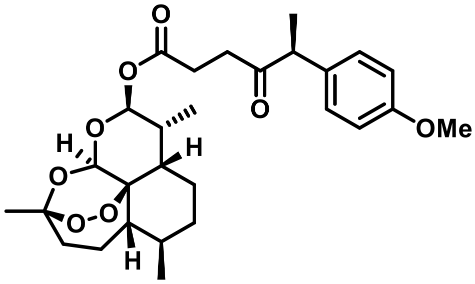

Fig. 4. (69-a)

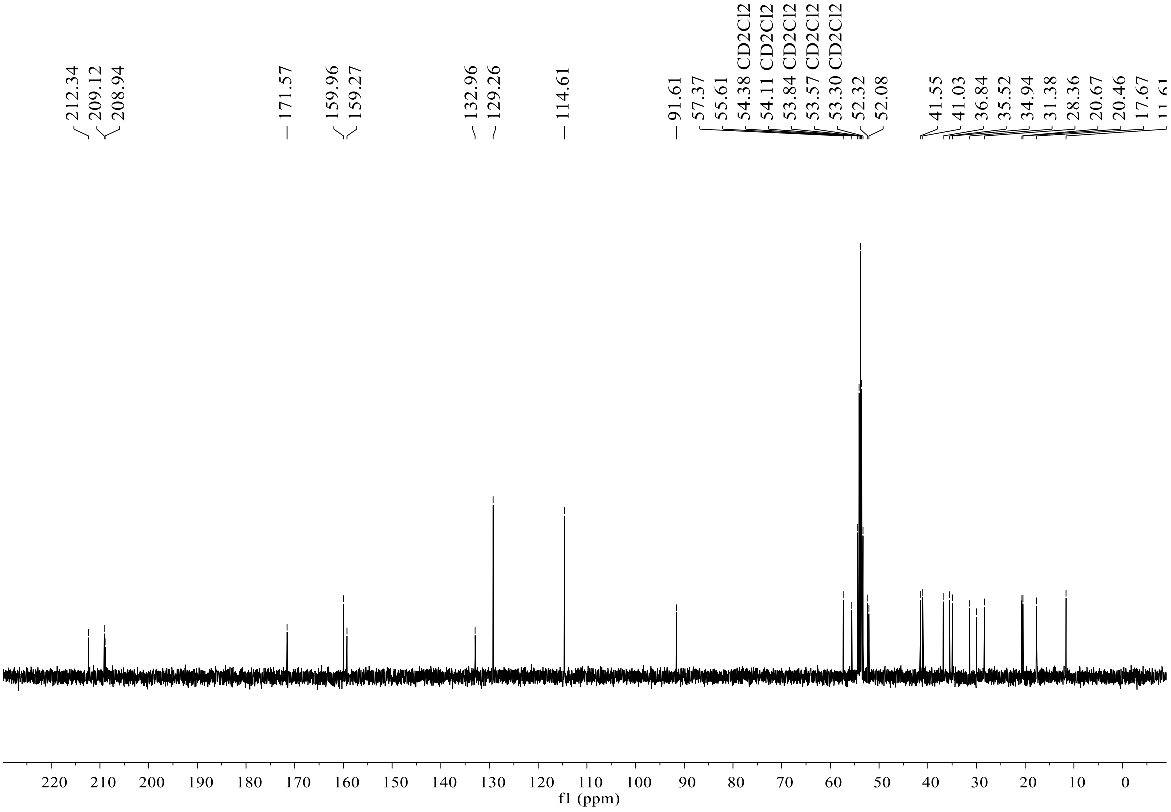

Supplementary Figure 88. ^{1}H NMR and ^{13}C NMR spectrum of **69-a.**

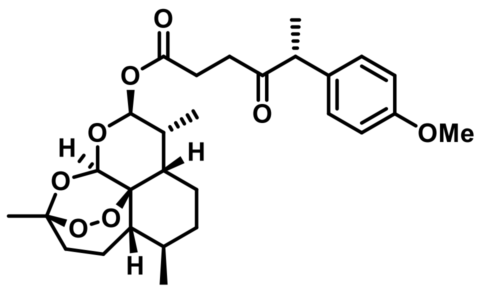

Fig. 4. (69-b)

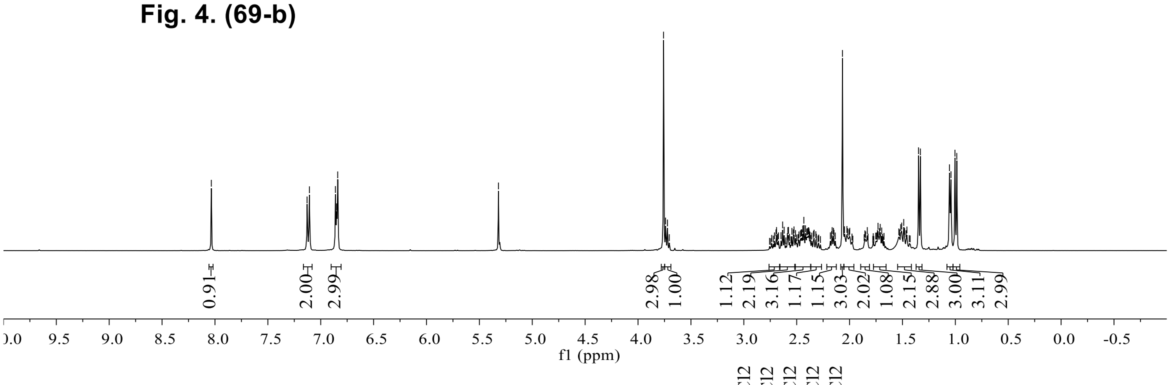

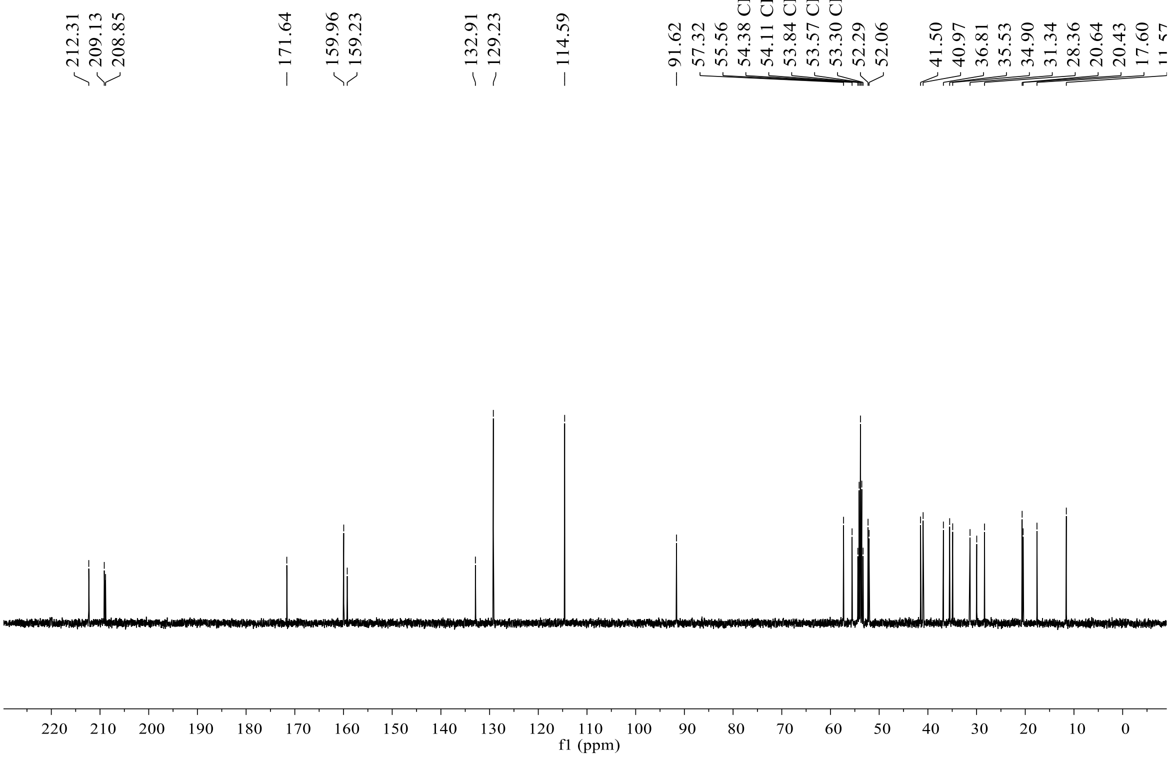

Supplementary Figure 89. ^{1}H NMR and ^{13}C NMR spectrum of **69-b.**

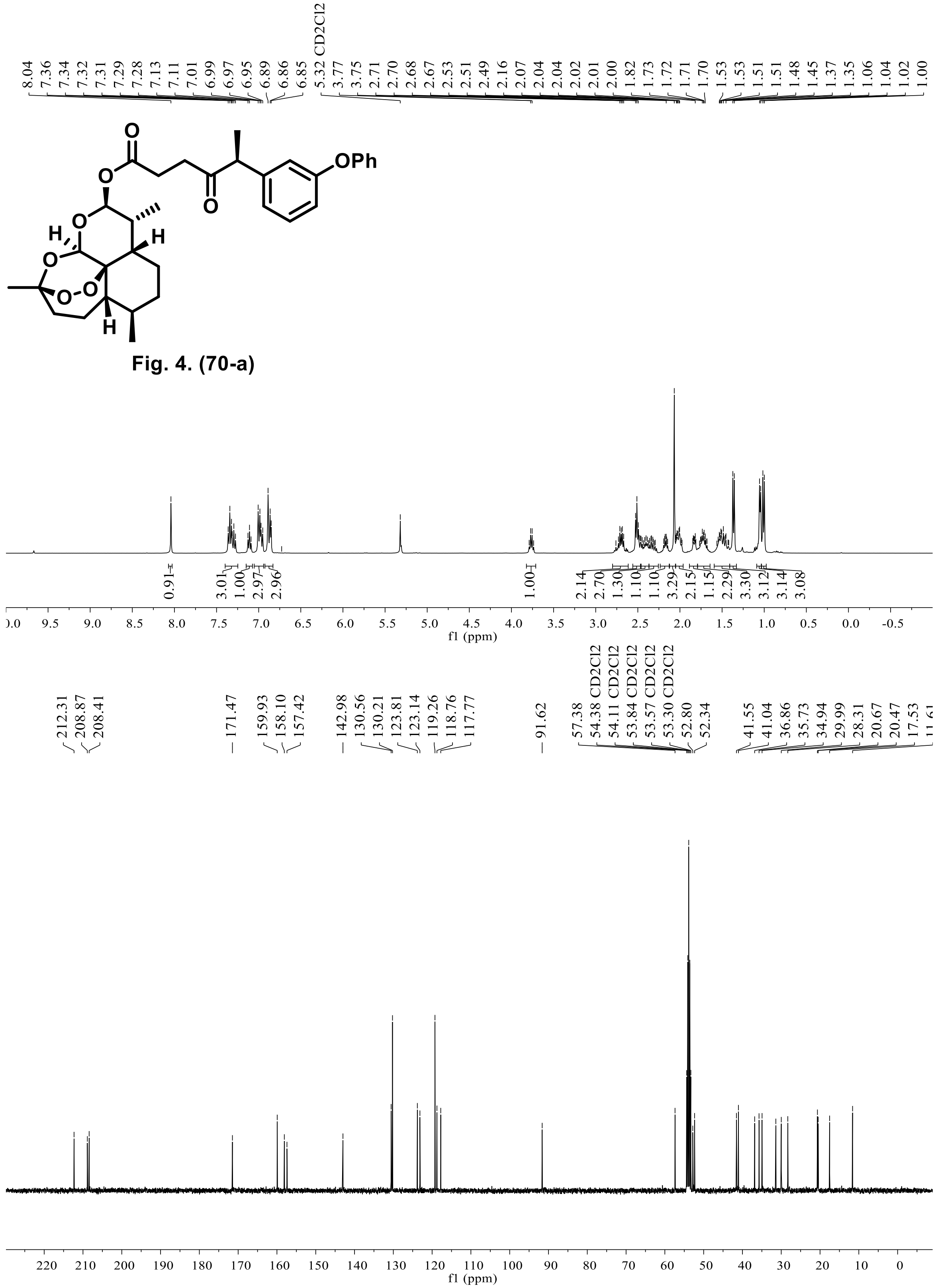

Supplementary Figure 90. ^{1}H NMR and ^{13}C NMR spectrum of **70-a.**

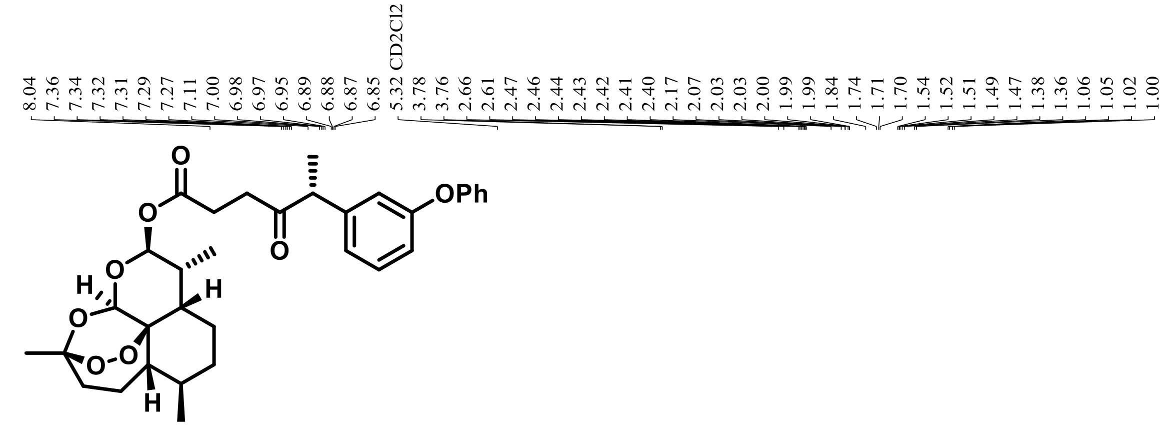

Fig. 4. (70-b)

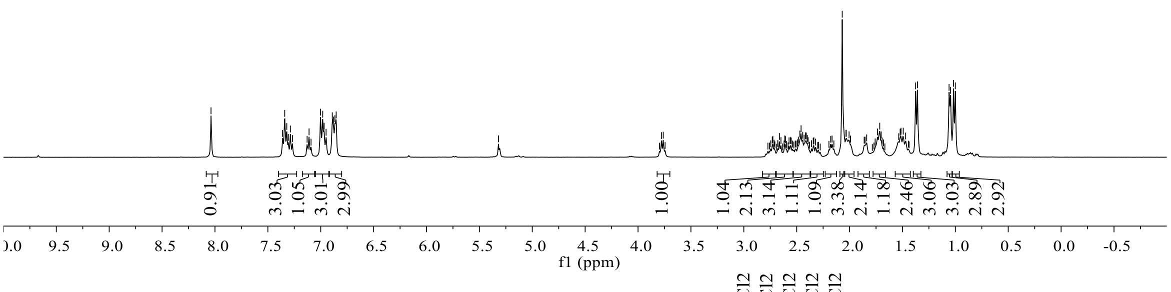

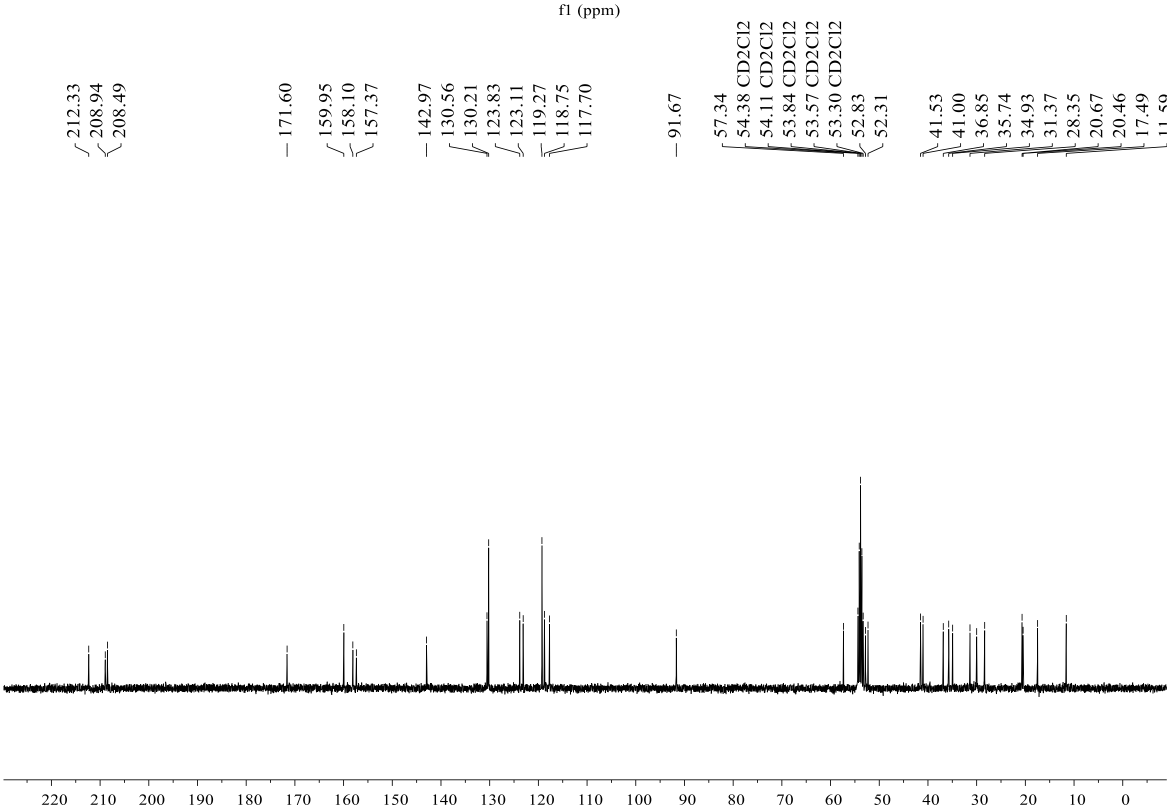

Supplementary Figure 91. ^{1}H NMR and ^{13}C NMR spectrum of **70-b.**

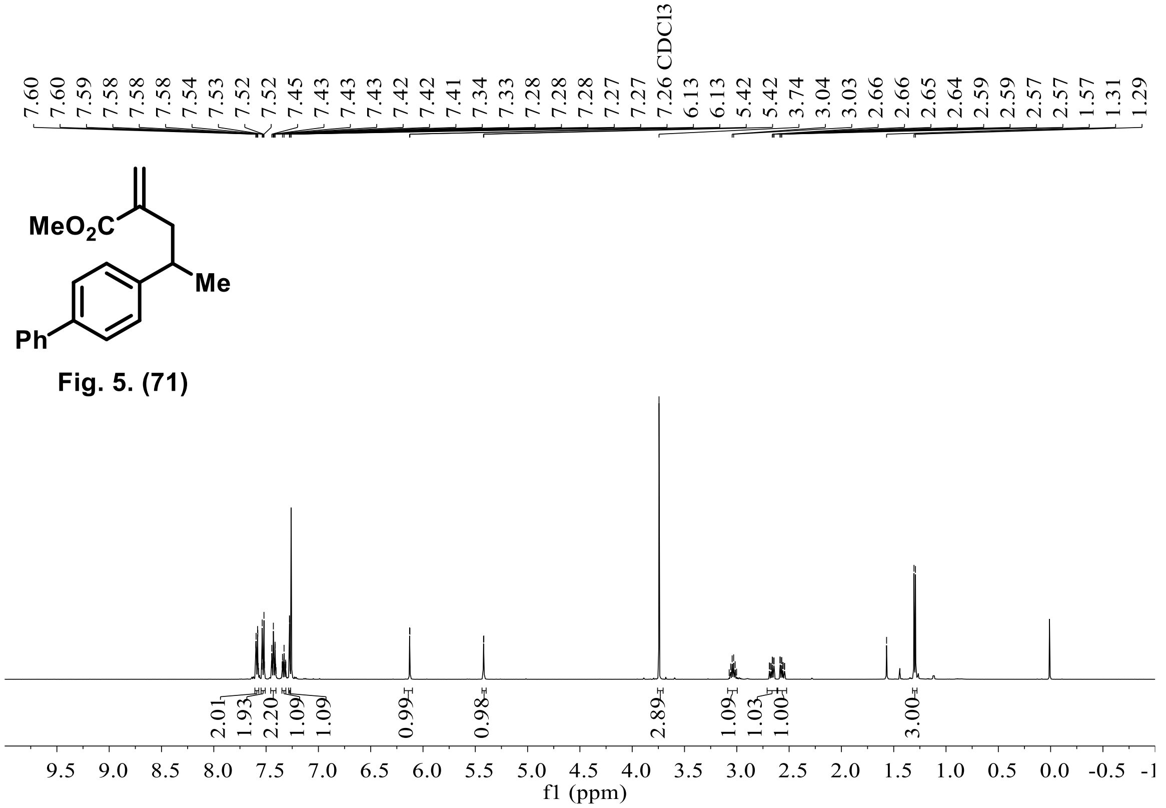

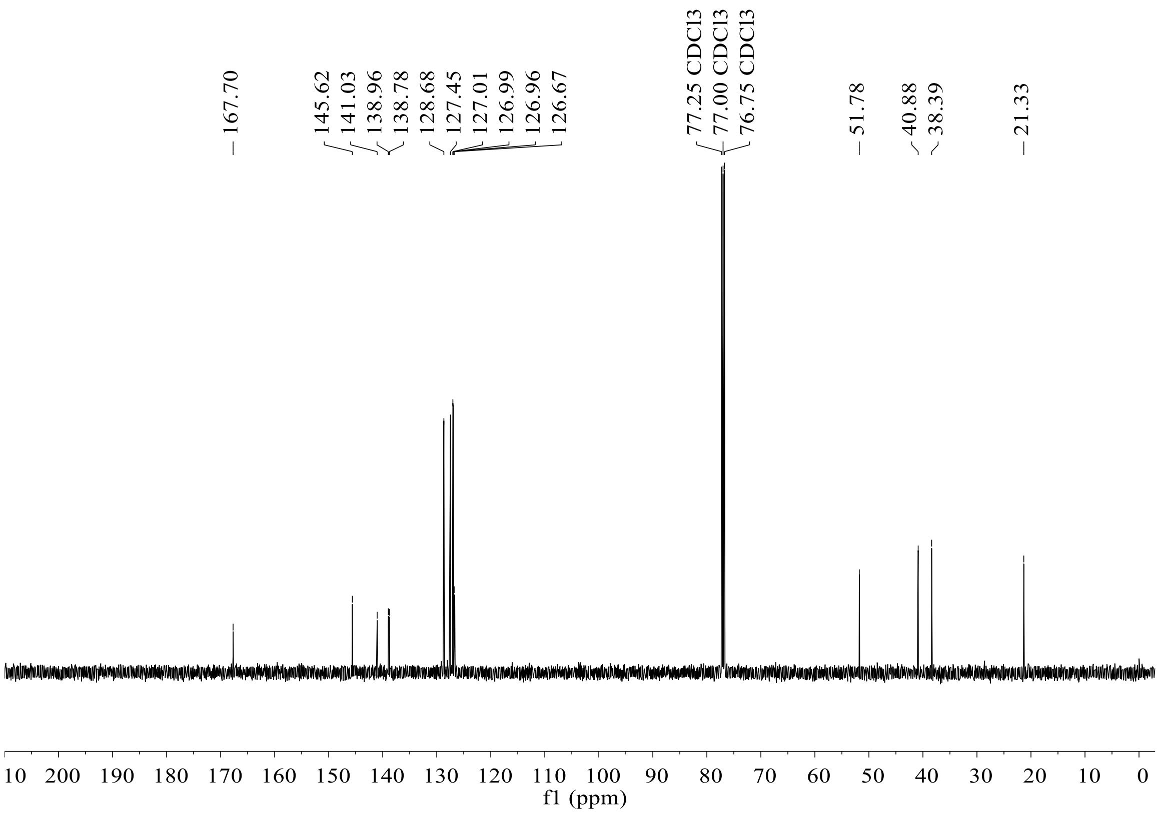

Supplementary Figure 92. ^{1}H NMR and ^{13}C NMR spectrum of **71.**

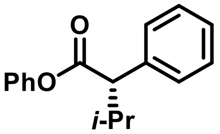

Fig. 6. (72)

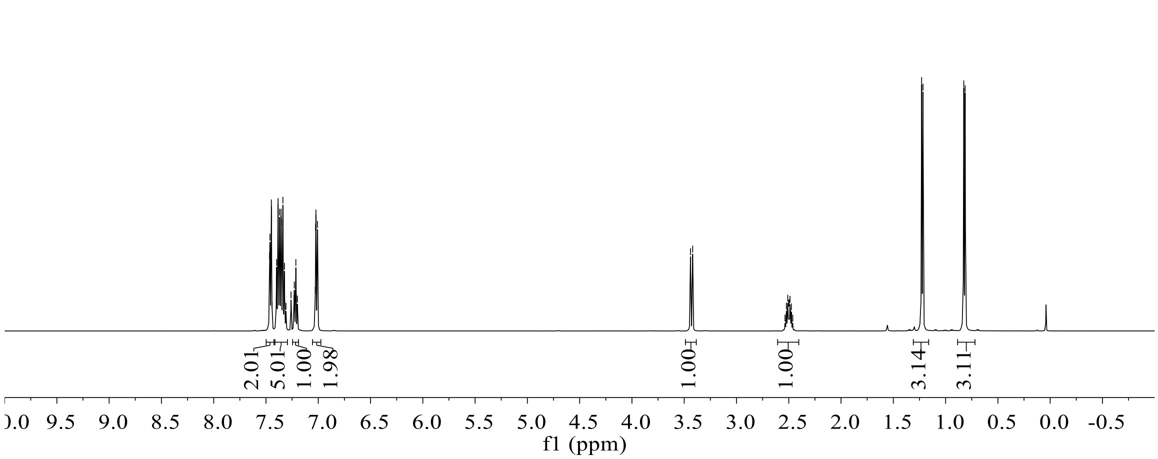

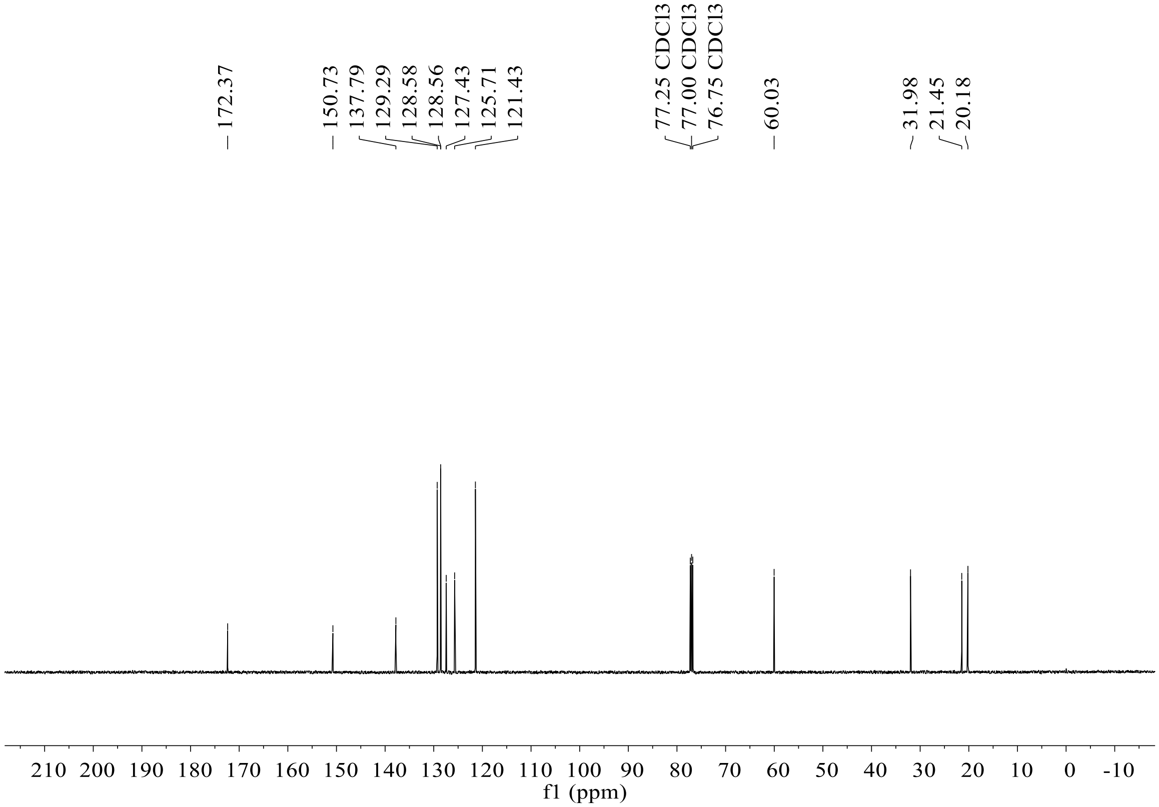

Supplementary Figure 93. ^{1}H NMR and ^{13}C NMR spectrum of **72.**

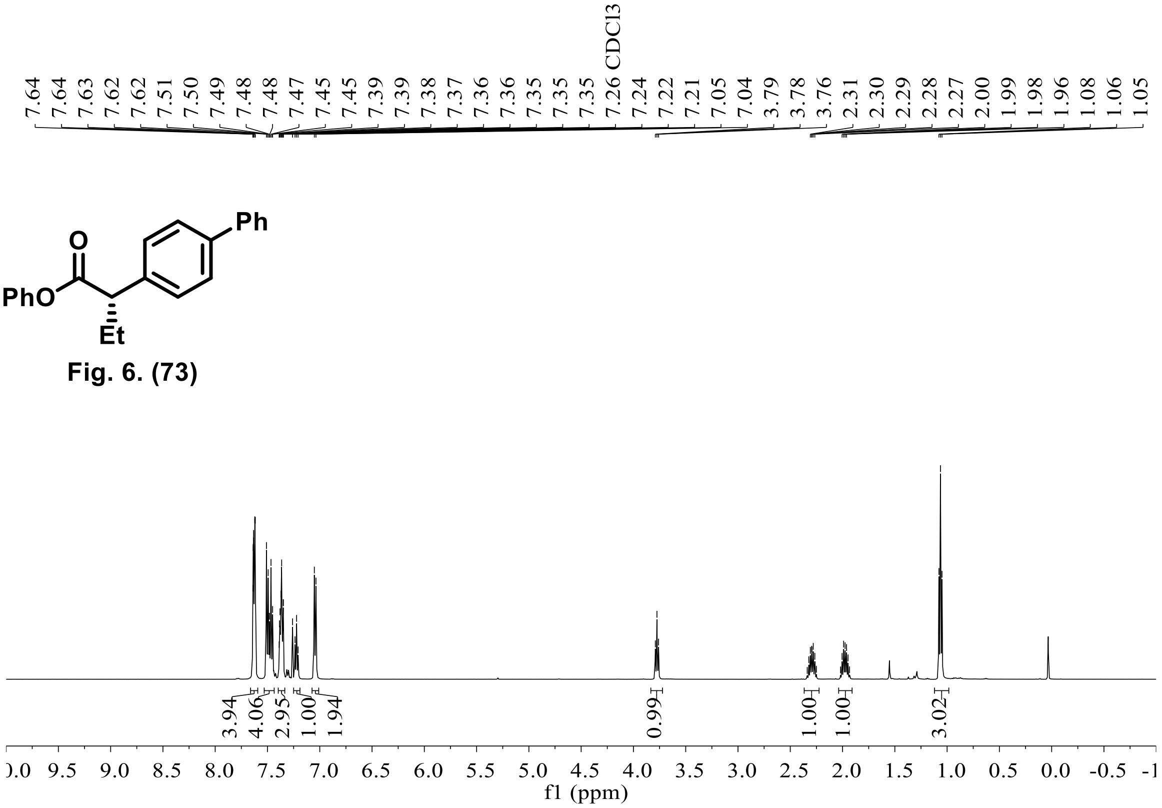

Fig. 6. (73)

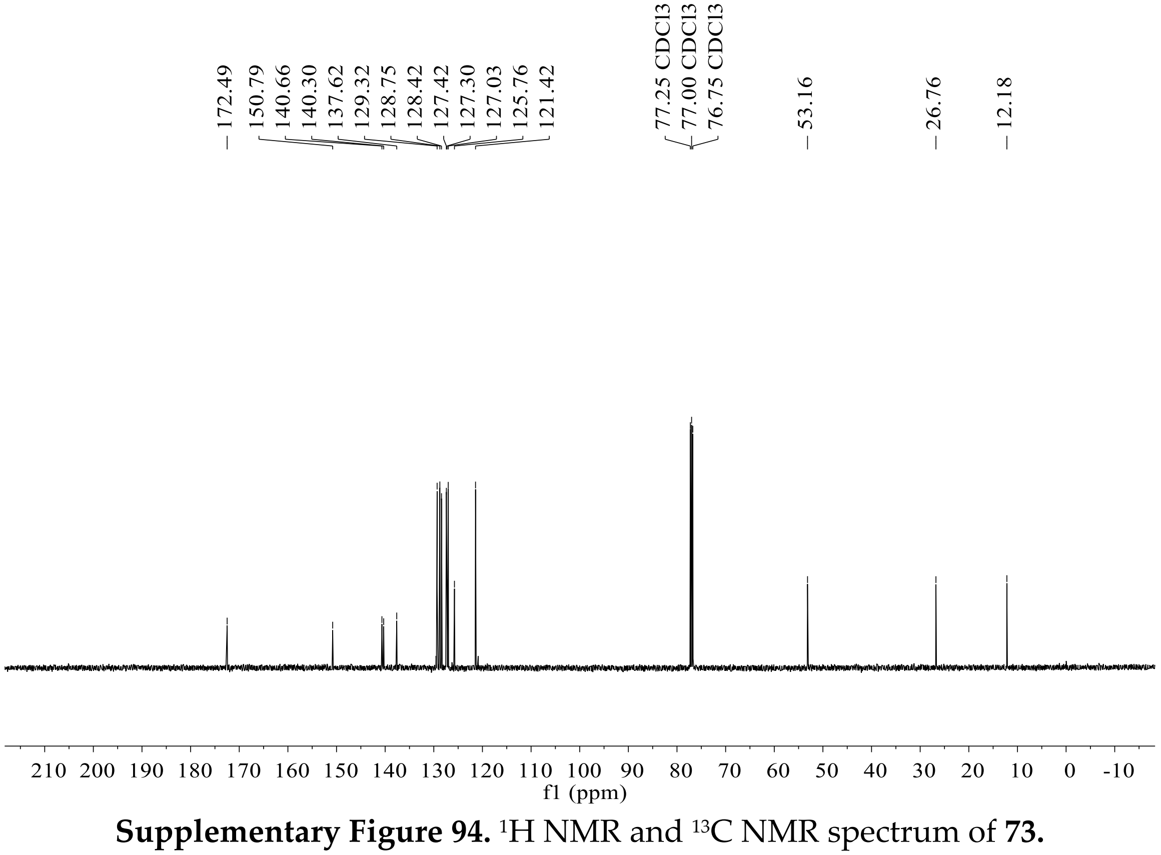

Supplementary Figure 94. ^{1}H NMR and ^{13}C NMR spectrum of **73.**

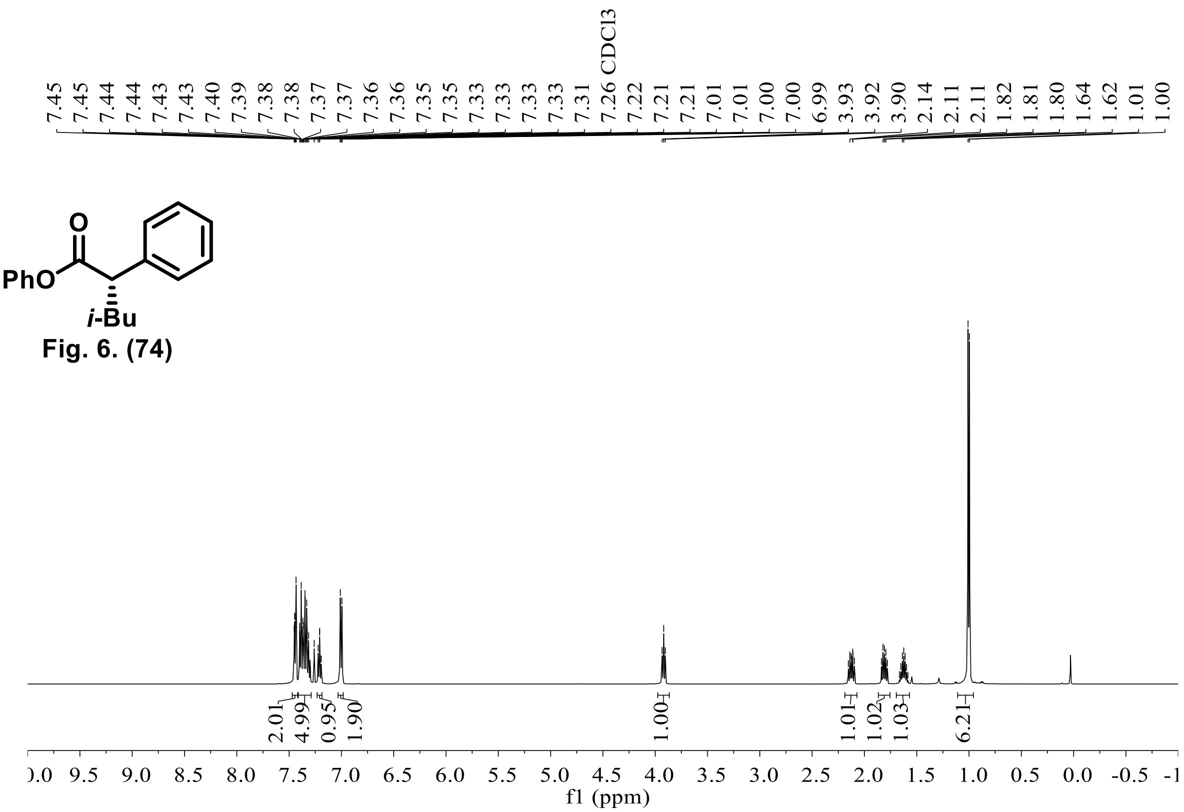

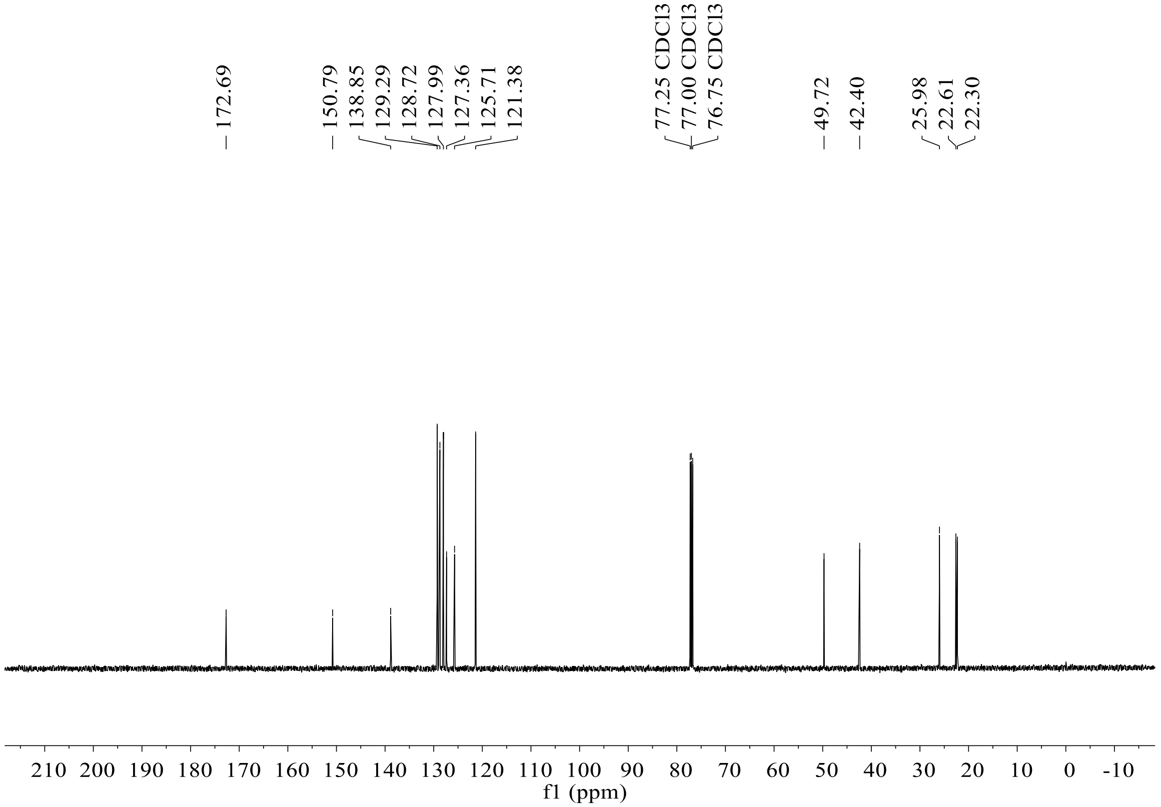

Supplementary Figure 95. ^{1}H NMR and ^{13}C NMR spectrum of **74.**

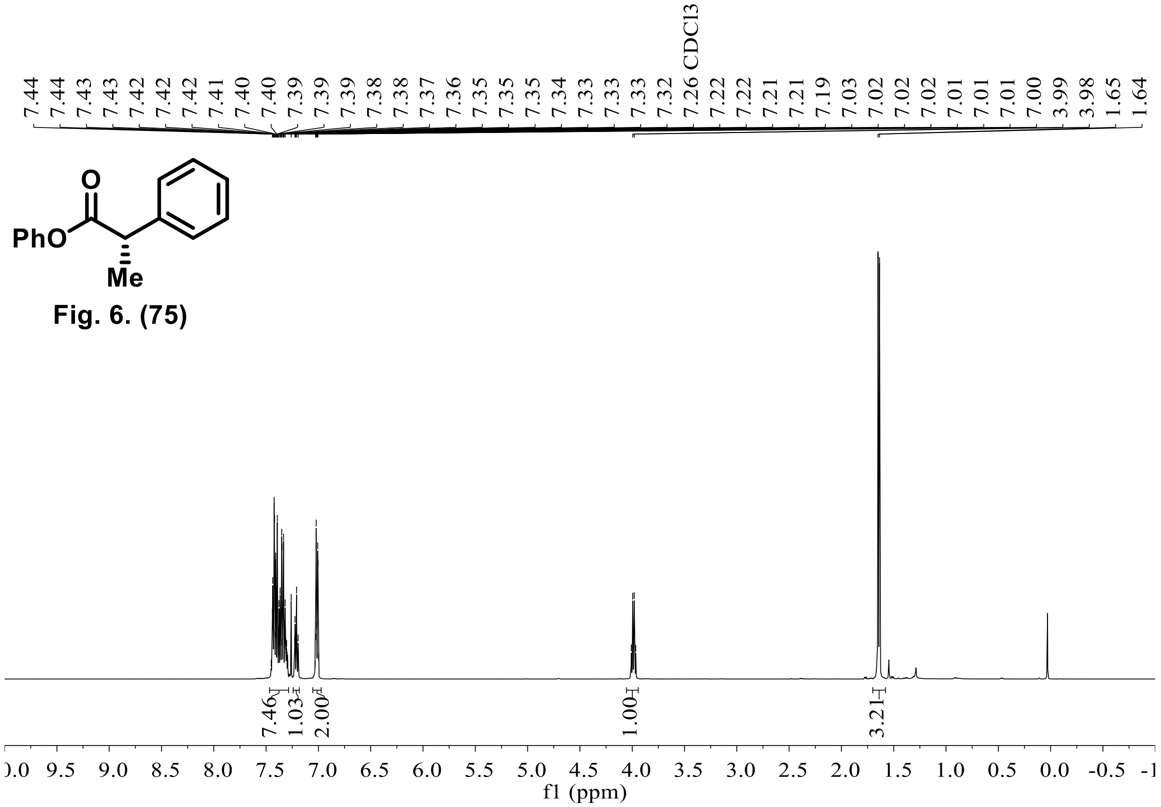

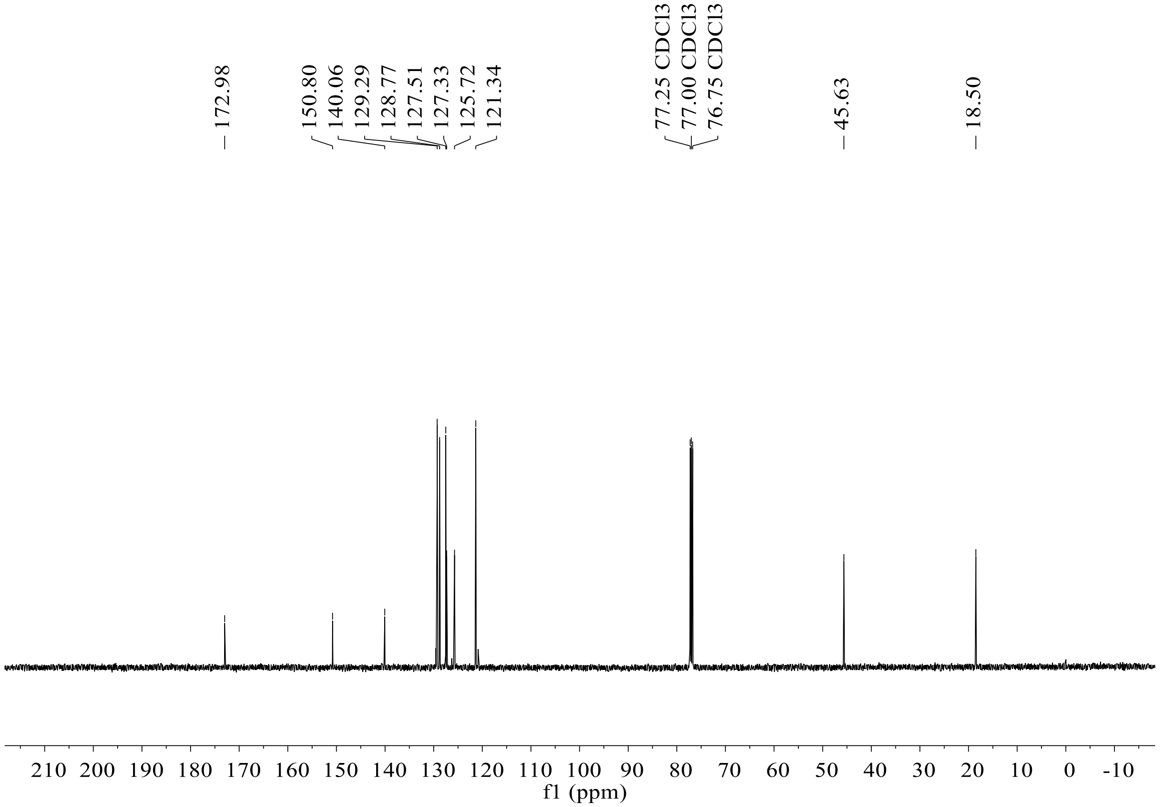

Supplementary Figure 96. ^{1}H NMR and ^{13}C NMR spectrum of **75.**

Stereoselectivity Analysis

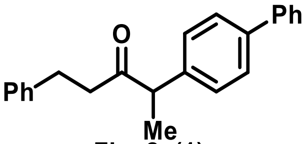

Fig. 2. (1)

(S)-**L**: 94% ee; (R)-**L**: 94% ee.

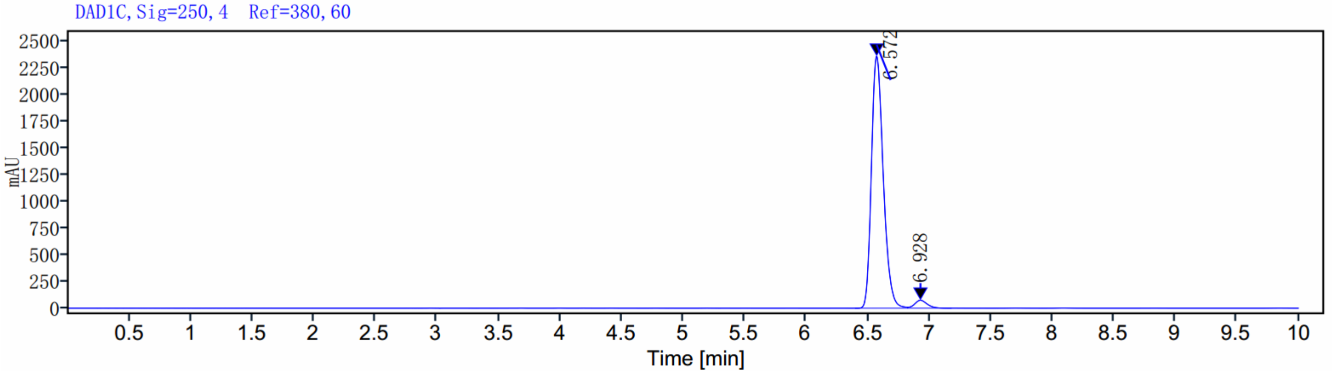

Signal: DAD1C, Sig=250, 4 Ref=380, 60

RetTime [min]	Type	Width [min]	Area [mAU*s]	Height [mAU]	Area %
6.572	BV	0.42727	15848.79806	2366.32082	96.7963
6.928	VV	0.35579	524.55261	74.60272	3.2037
		Totals	16373.35067		

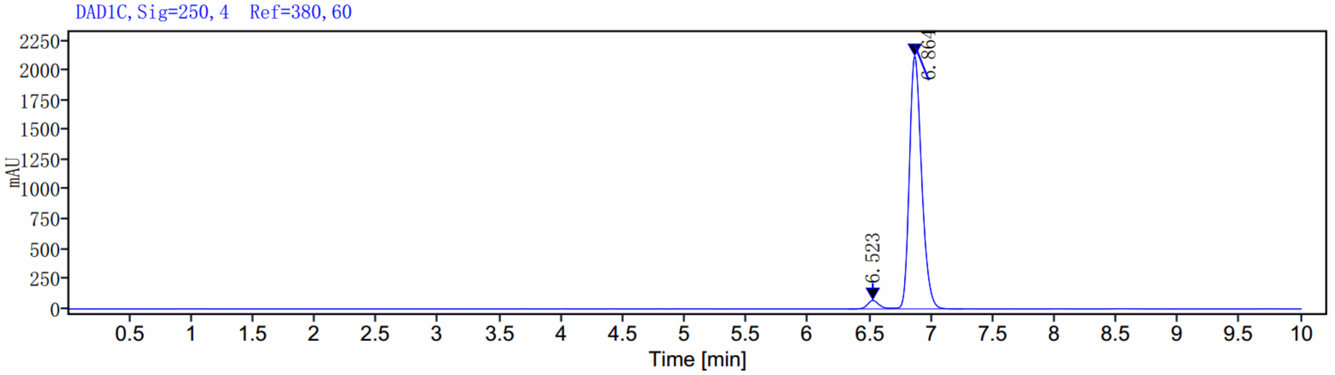

Signal: DAD1C, Sig=250, 4 Ref=380, 60

RetTime [min]	Type	Width [min]	Area [mAU*s]	Height [mAU]	Area %
6.523	BV	0.33393	461.56585	71.43462	3.0547
6.864	VV	0.59199	14648.36121	2118.56355	96.9453
		Totals	15109.92706		

Supplementary Figure 97. HPLC data of **1**.

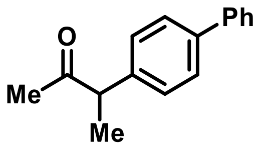

Fig. 2. (2)

(*S*)-**L**: 95% ee; (*R*)-**L**: 95% ee.

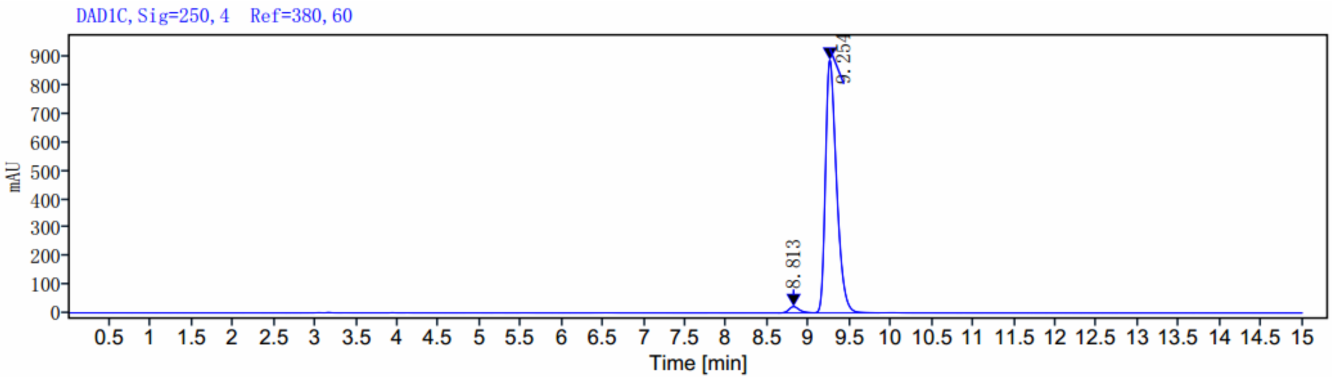

Signal: DAD1C, Sig=250, 4 Ref=380, 60

RetTime [min]	Type	Width [min]	Area [mAU*s]	Height [mAU]	Area %
8.813	BV	0.44025	197.30455	22.32957	2.3092
9.254	VV	0.79954	8347.14787	891.80106	97.6908
		Totals	8544.45242		

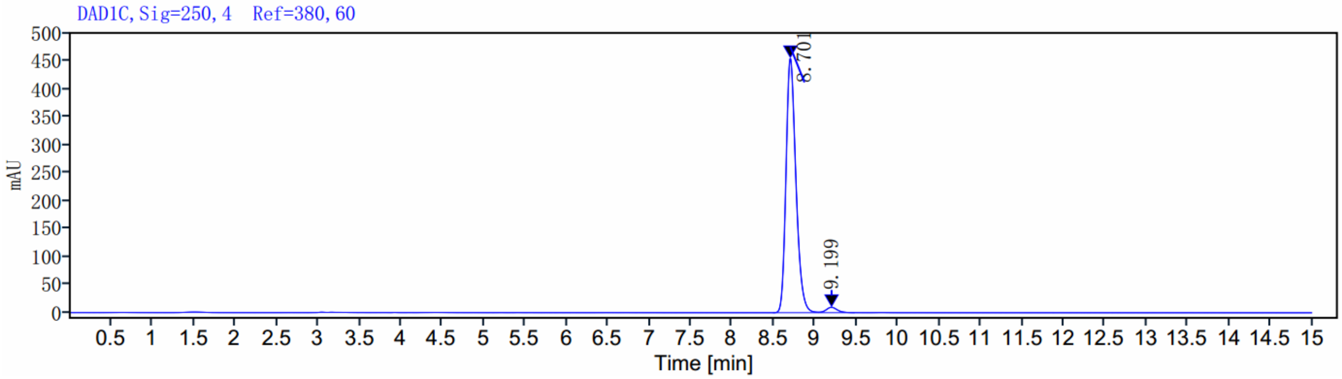

Signal: DAD1C, Sig=250, 4 Ref=380, 60

RetTime [min]	Type	Width [min]	Area [mAU*s]	Height [mAU]	Area %
8.701	BV	0.55026	3789.64316	454.94058	97.6704
9.199	VV	0.43580	90.38730	9.65540	2.3296
		Totals	3880.03046		

Supplementary Figure 98. HPLC data of **2**.

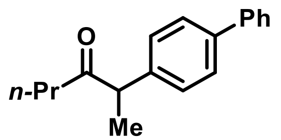

Fig. 2. (3)

(*S*)-**L**: 94% ee; (*R*)-**L**: 94% ee.

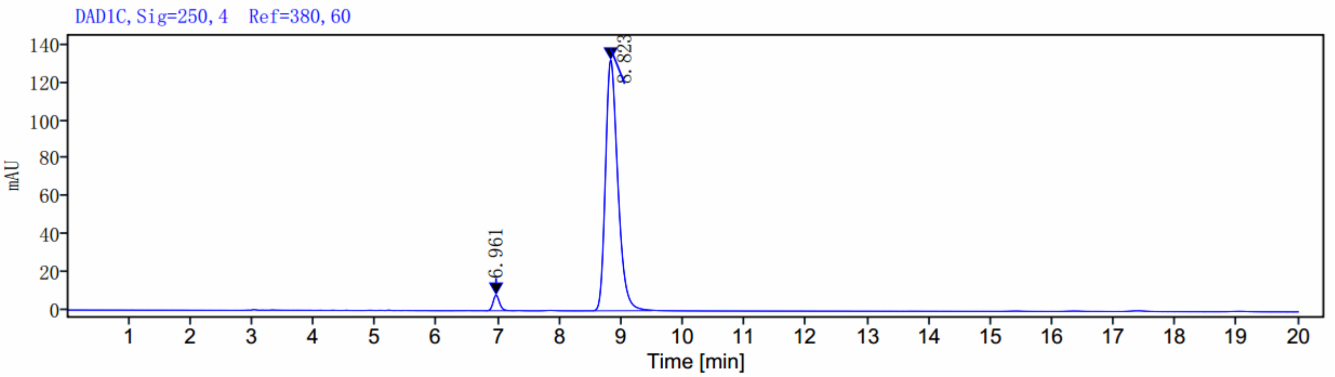

Signal: DAD1C, Sig=250, 4 Ref=380, 60

RetTime [min]	Type	Width [min]	Area [mAU*s]	Height [mAU]	Area %
6.961	BV	0.37413	59.85171	8.19561	3.1344
8.823	VV	0.96162	1849.64040	132.47196	96.8656
		Totals	1909.49211		

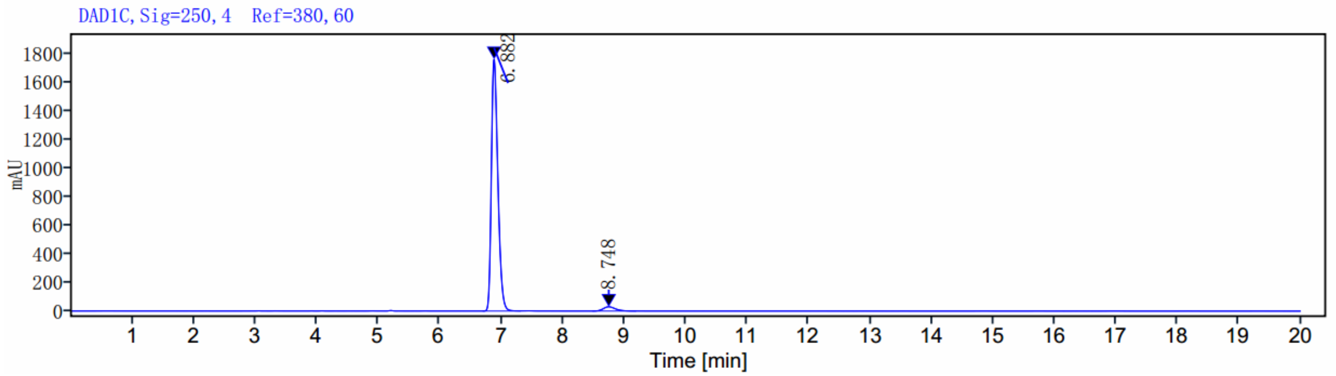

Signal: DAD1C, Sig=250, 4 Ref=380, 60

RetTime [min]	Type	Width [min]	Area [mAU*s]	Height [mAU]	Area %
6.882	BV	0.62051	12881.16120	1757.95650	96.9378
8.748	VV	0.70837	406.90732	29.66519	3.0622
		Totals	13288.06852		

Supplementary Figure 99. HPLC data of **3.**

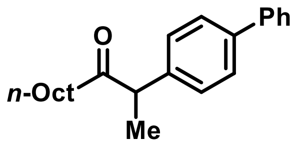

Fig. 2. (4)

(*S*)-**L**: 93% ee; (*R*-**L**: 94% ee.

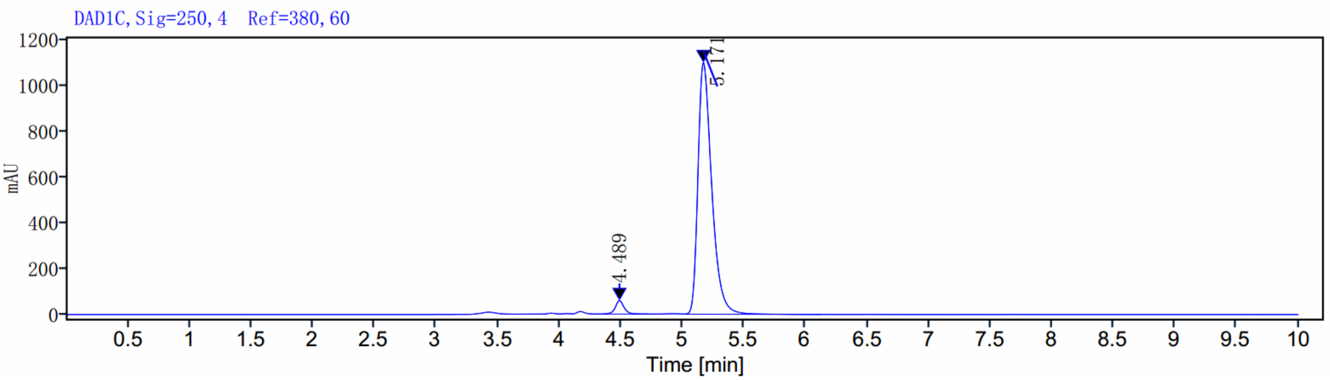

Signal: DAD1C, Sig=250, 4 Ref=380, 60

RetTime [min]	Type	Width [min]	Area [mAU*s]	Height [mAU]	Area %
4.489	VV	0.37407	293.94275	58.72576	3.3222
5.171	VB	1.11393	8553.86014	1098.67495	96.6778
		Totals	8847.80288		

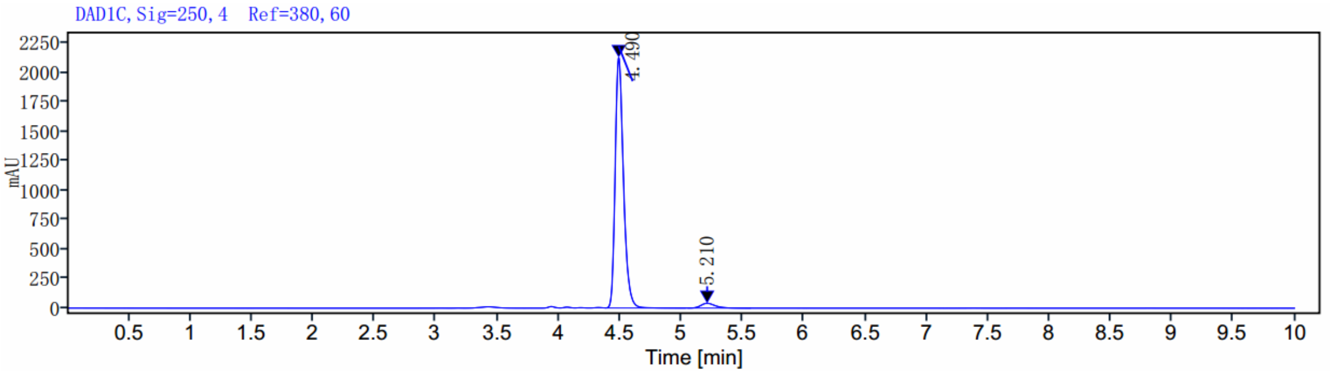

Signal: DAD1C, Sig=250, 4 Ref=380, 60

RetTime [min]	Type	Width [min]	Area [mAU*s]	Height [mAU]	Area %
4.490	VB	0.65494	10059.94192	2133.32331	96.8540
5.210	BB	0.51583	326.76904	40.62984	3.1460
		Totals	10386.71096		

Supplementary Figure 100. HPLC data of **4**.

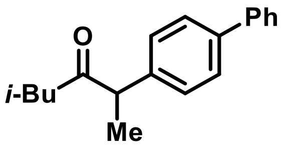

Fig. 2. (5)

(*S*)-**L**: 95% ee; (*R*)-**L**: 95% ee.

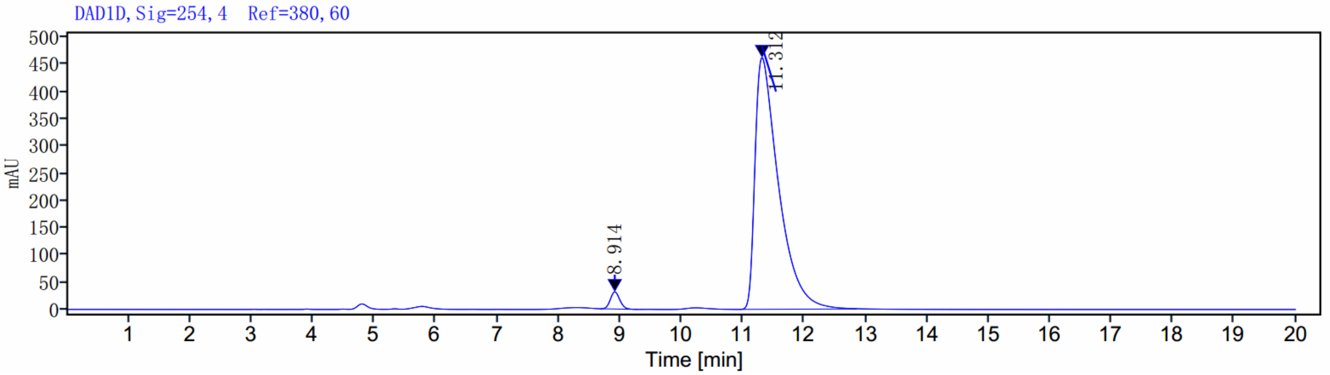

Signal: DAD1D, Sig=254, 4 Ref=380, 60

RetTime [min]	Type	Width [min]	Area [mAU*s]	Height [mAU]	Area %
8.914	VB	0.60463	339.13198	31.59254	2.6732
11.312	VV	1.89927	12347.43523	463.09006	97.3268
		Totals	12686.56721		

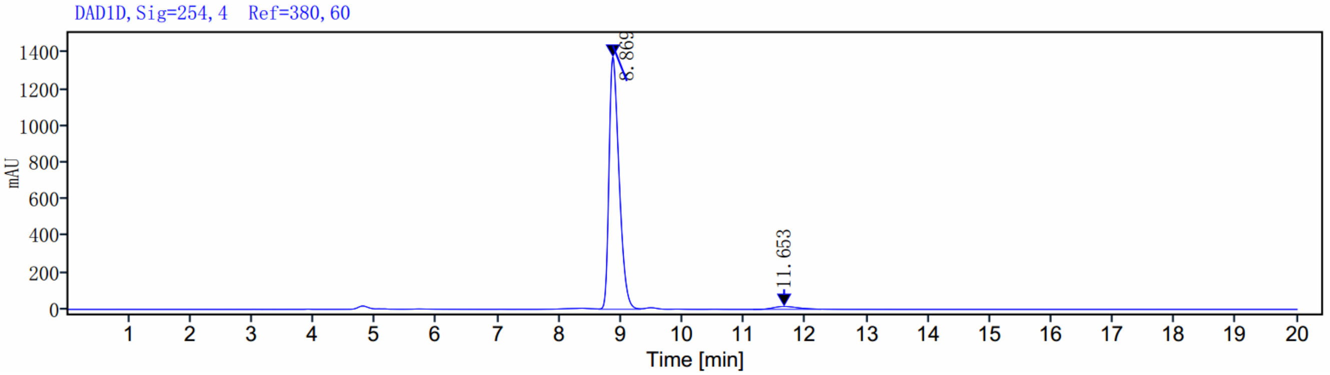

Signal: DAD1D, Sig=254, 4 Ref=380, 60

RetTime [min]	Type	Width [min]	Area [mAU*s]	Height [mAU]	Area %
8.869	VV	0.68739	15629.78987	1376.18337	97.2655
11.653	BV	1.10172	439.41775	15.75778	2.7345
		Totals	16069.20761		

Supplementary Figure 101. HPLC data of **5.**

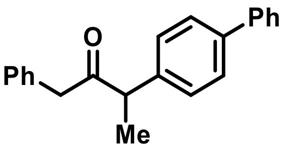

Fig. 2. (6)

(*S*)-**L**: 92% ee; (*R*)-**L**: 93% ee.

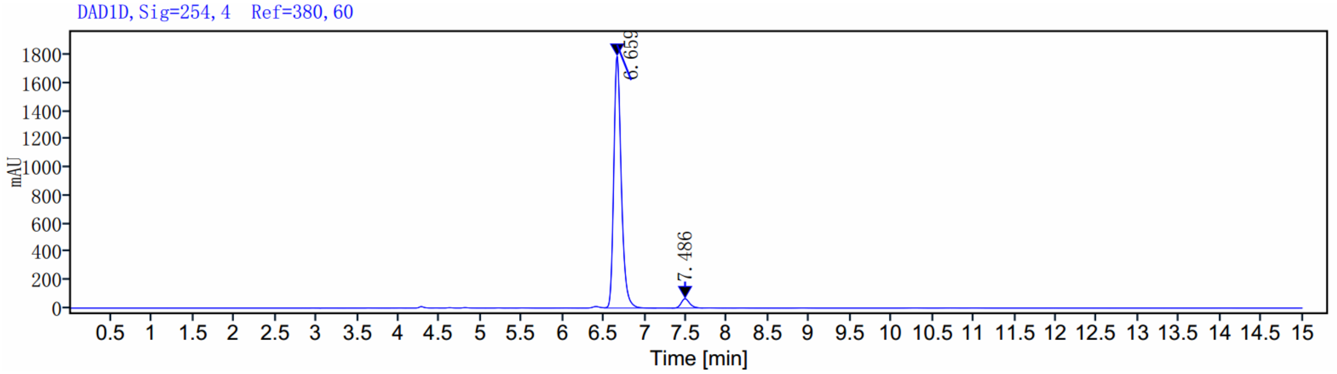

Signal: DAD1D, Sig=254, 4 Ref=380, 60

RetTime [min]	Type	Width [min]	Area [mAU*s]	Height [mAU]	Area %
6.659	VV	0.63954	11409.96467	1794.52450	96.0168
7.486	BV	0.38044	473.33365	66.34417	3.9832
		Totals	11883.29832		

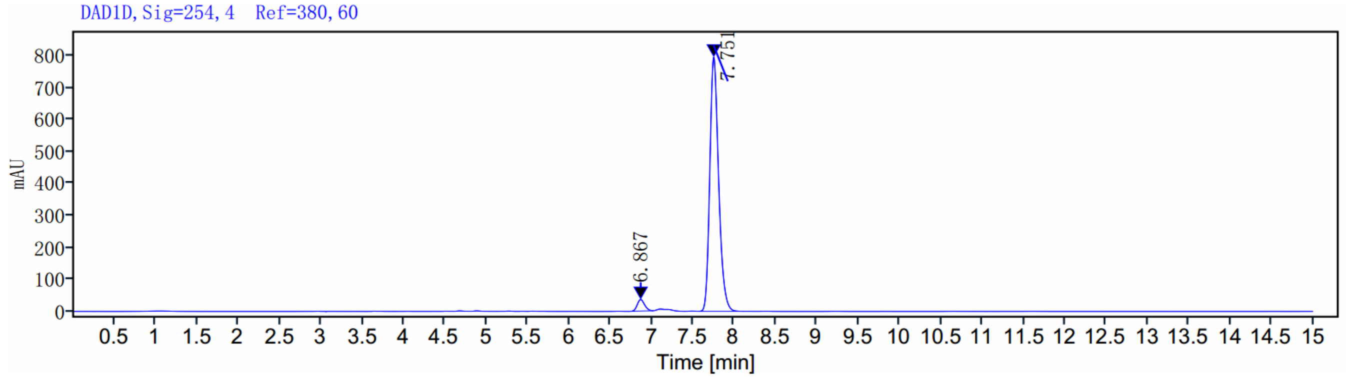

Signal: DAD1D, Sig=254, 4 Ref=380, 60

RetTime [min]	Type	Width [min]	Area [mAU*s]	Height [mAU]	Area %
6.867	BB	0.27189	223.50813	36.18068	3.6421
7.751	VB	0.57746	5913.30891	794.92146	96.3579
		Totals	6136.81703		

Supplementary Figure 102. HPLC data of **6**.

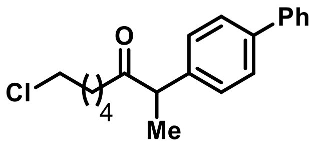

Fig. 2. (7)

(*S*)-**L**: 93% ee; (*R*)-**L**: 93% ee.

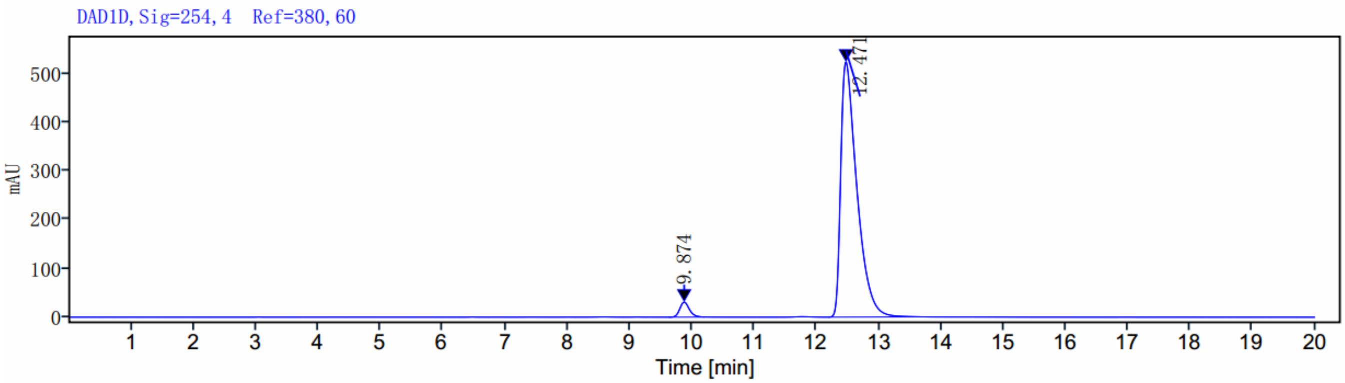

Signal: DAD1D, Sig=254, 4 Ref=380, 60

RetTime [min]	Type	Width [min]	Area [mAU*s]	Height [mAU]	Area %
9.874	BV	0.57467	331.50741	30.86590	3.3960
12.471	VV	1.35102	9430.33297	524.33897	96.6040
		Totals	9761.84038		

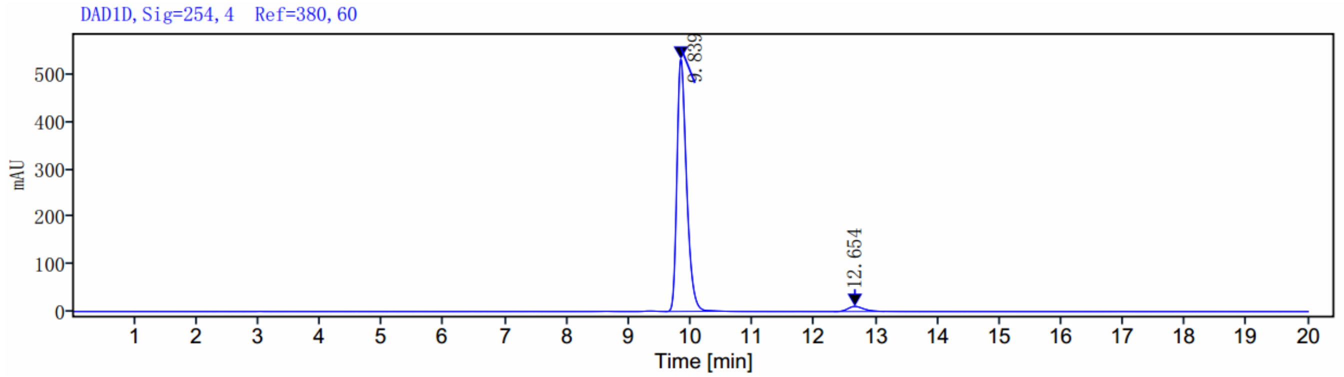

Signal: DAD1D, Sig=254, 4 Ref=380, 60

RetTime [min]	Type	Width [min]	Area [mAU*s]	Height [mAU]	Area %
9.839	BV	0.92160	5768.50387	533.45812	96.7484
12.654	BV	0.81760	193.87002	10.80988	3.2516
		Totals	5962.37389		

Supplementary Figure 103. HPLC data of **7**.

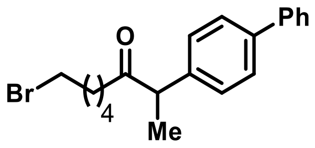

Fig. 2. (8)

(*S*)-**L**: 93% ee; (*R*)-**L**: 93% ee.

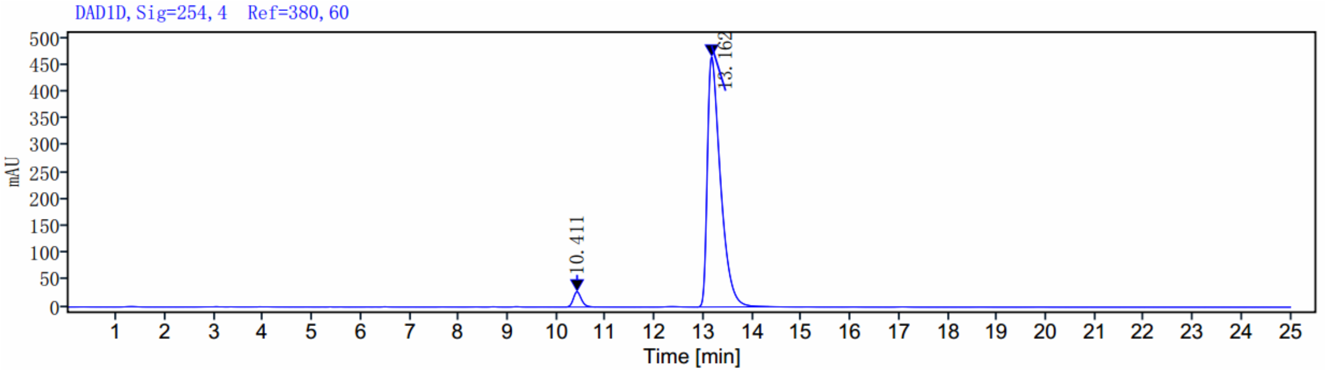

Signal: DAD1D, Sig=254, 4 Ref=380, 60

RetTime [min]	Type	Width [min]	Area [mAU*s]	Height [mAU]	Area %
10.411	VV	0.62306	323.29418	28.16164	3.5962
13.162	VV	1.46902	8666.53163	464.27389	96.4038
		Totals	8989.82580		

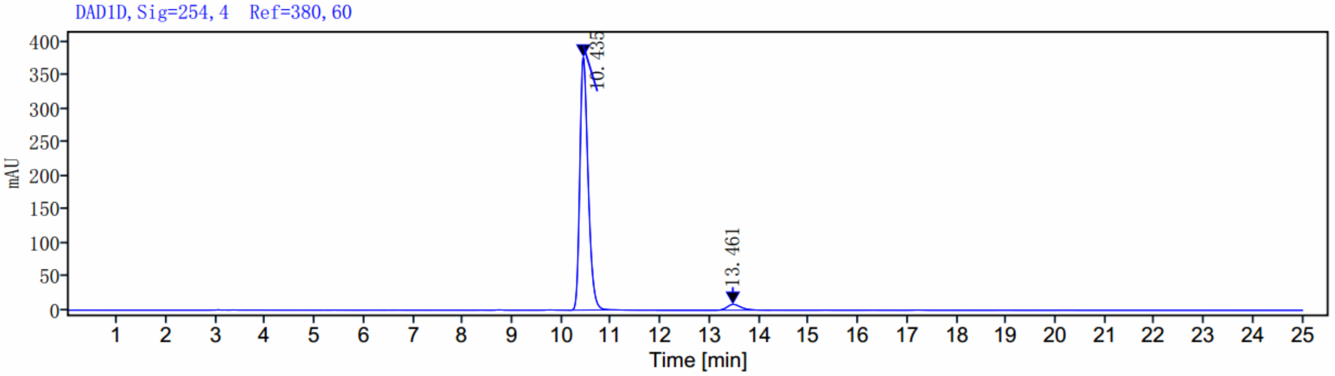

Signal: DAD1D, Sig=254, 4 Ref=380, 60

RetTime [min]	Type	Width [min]	Area [mAU*s]	Height [mAU]	Area %
10.435	VV	0.79186	4370.24603	376.85711	96.3783
13.461	MM m	2.32456	164.22704	8.60744	3.6217
		Totals	4534.47307		

Supplementary Figure 104. HPLC data of **8**.

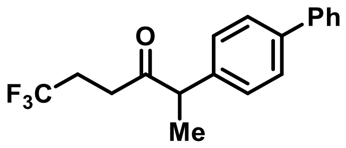

Fig. 2. (9)

(*S*)-**L**: 95% ee; (*R*)-**L**: 97% ee.

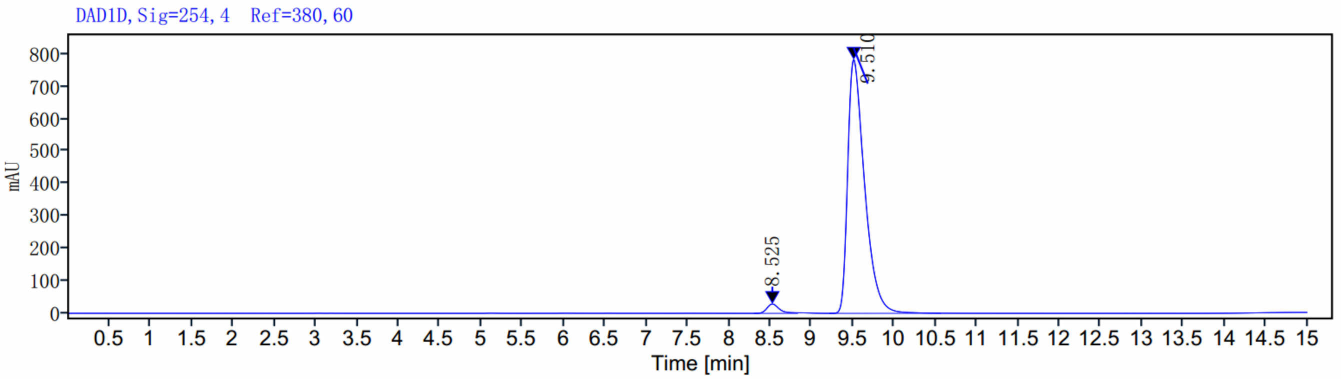

Signal: DAD1D, Sig=254, 4 Ref=380, 60

RetTime [min]	Type	Width [min]	Area [mAU*s]	Height [mAU]	Area %
8.525	BV	0.52979	303.13118	28.96395	2.6500
9.510	BV	1.34812	11135.73658	783.54221	97.3500
		Totals	11438.86776		

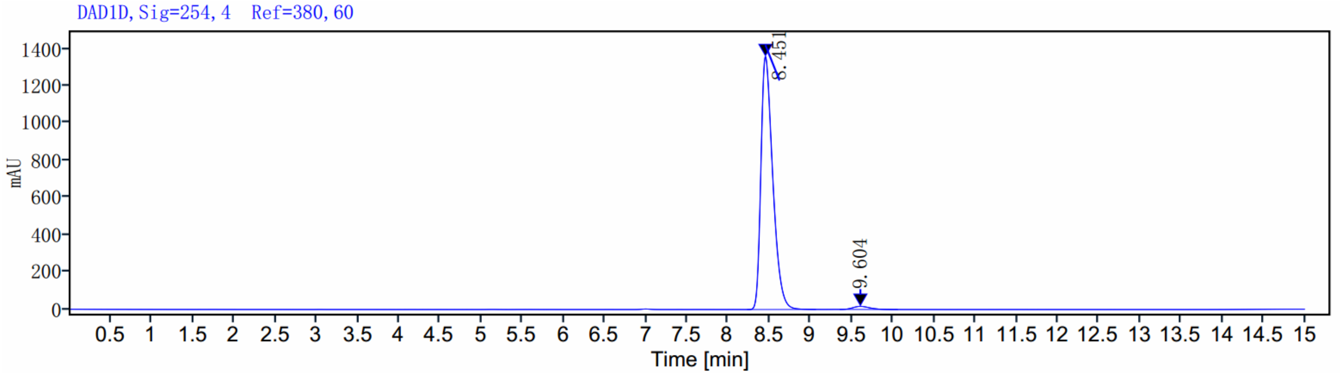

Signal: DAD1D, Sig=254, 4 Ref=380, 60

RetTime [min]	Type	Width [min]	Area [mAU*s]	Height [mAU]	Area %
8.451	BV	0.83535	13611.93977	1361.07175	98.3072
9.604	VV	0.70574	234.39230	16.46830	1.6928
		Totals	13846.33207		

Supplementary Figure 105. HPLC data of **9**.

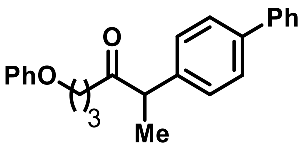

Fig. 2. (10)

(*S*)-**L**: 94% ee; (*R*)-**L**: 93% ee.

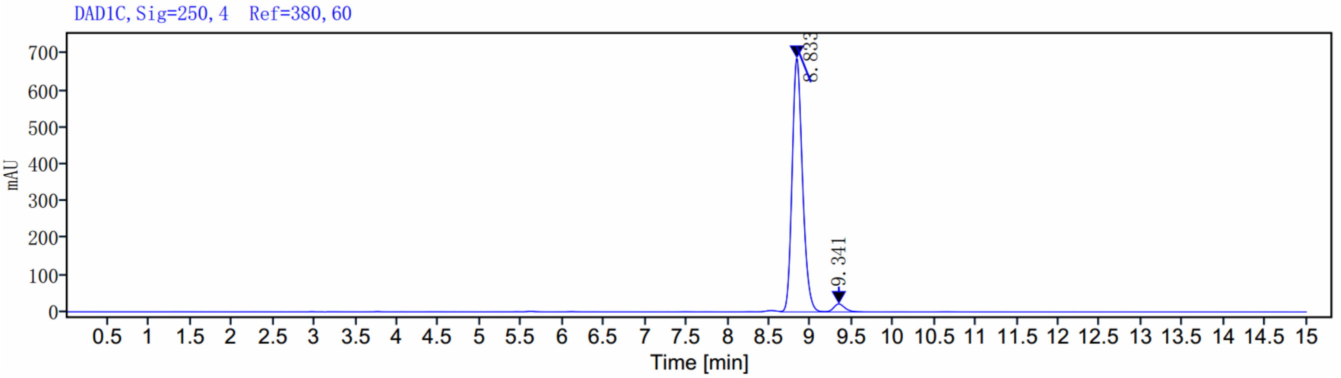

Signal: DAD1C, Sig=250, 4 Ref=380, 60

RetTime [min]	Type	Width [min]	Area [mAU*s]	Height [mAU]	Area %
8.833	VV	0.54059	6011.22598	687.82663	96.7754
9.341	VB	0.45379	200.29528	21.55109	3.2246
		Totals	6211.52125		

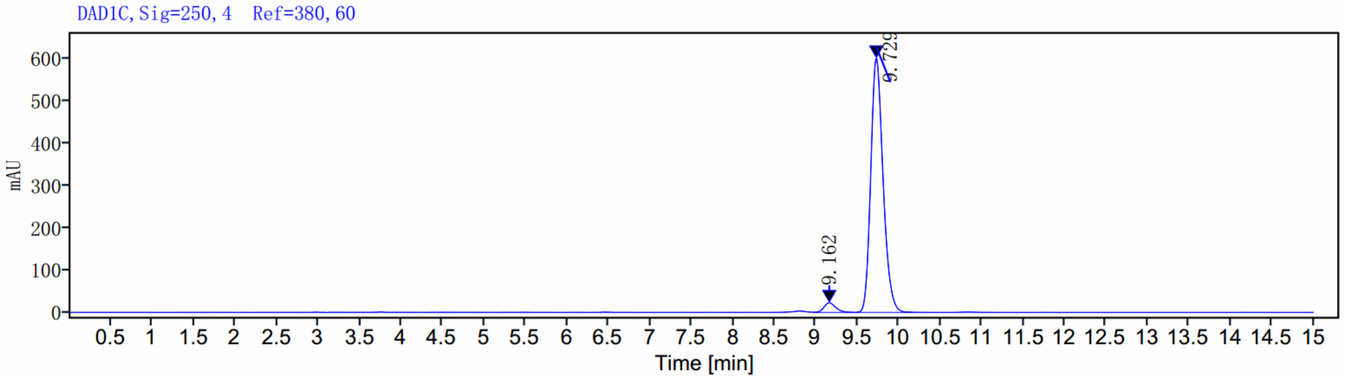

Signal: DAD1C, Sig=250, 4 Ref=380, 60

RetTime [min]	Type	Width [min]	Area [mAU*s]	Height [mAU]	Area %
9.162	VB	0.48618	223.24002	22.77676	3.4565
9.729	BV	0.69897	6235.32641	603.13065	96.5435
		Totals	6458.56643		

Supplementary Figure 106. HPLC data of **10**.

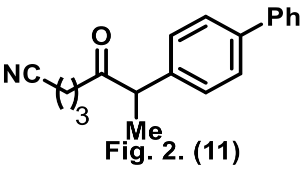

Fig. 2. (11)

(*S*)-**L**: 94% ee; (*R*)-**L**: 94% ee.

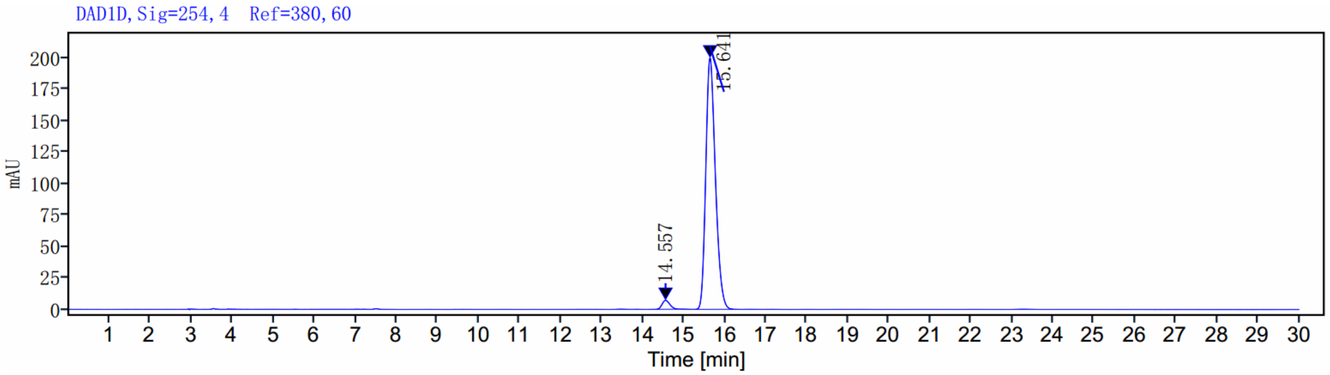

Signal: DAD1D, Sig=254, 4 Ref=380, 60

RetTime [min]	Type	Width [min]	Area [mAU*s]	Height [mAU]	Area %
14.557	VV	0.50003	93.51496	7.11298	2.7948
15.641	VV	0.92224	3252.48375	200.32318	97.2052
		Totals	3345.99872		

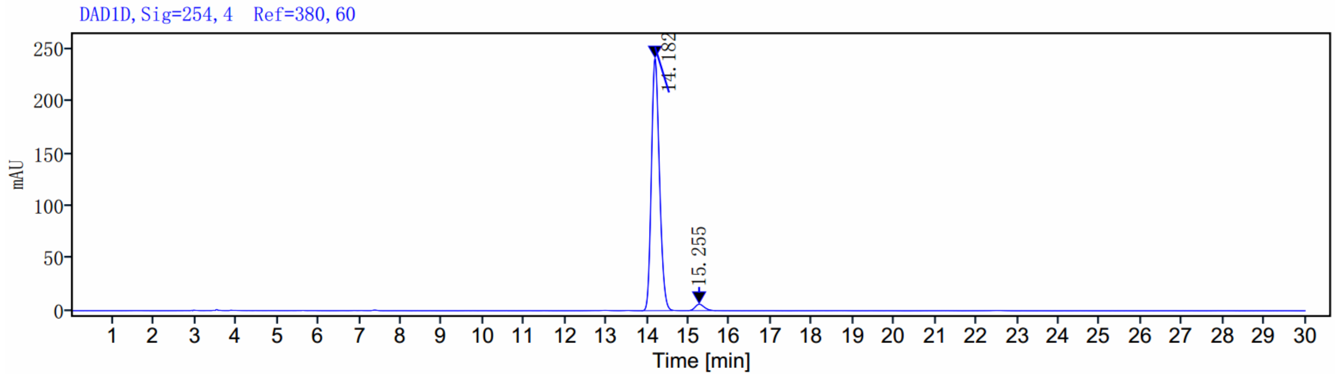

Signal: DAD1D, Sig=254, 4 Ref=380, 60

RetTime [min]	Type	Width [min]	Area [mAU*s]	Height [mAU]	Area %
14.182	VV	0.76059	3296.73383	241.25052	97.1733
15.255	VV	0.68628	95.90095	6.17637	2.8267
		Totals	3392.63478		

Supplementary Figure 107. HPLC data of **11**.

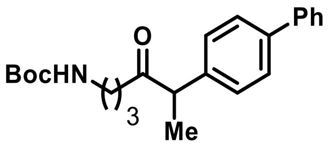

Fig. 2. (12)

(*S*)-**L**: 92% ee; (*R*)-**L**: 93% ee.

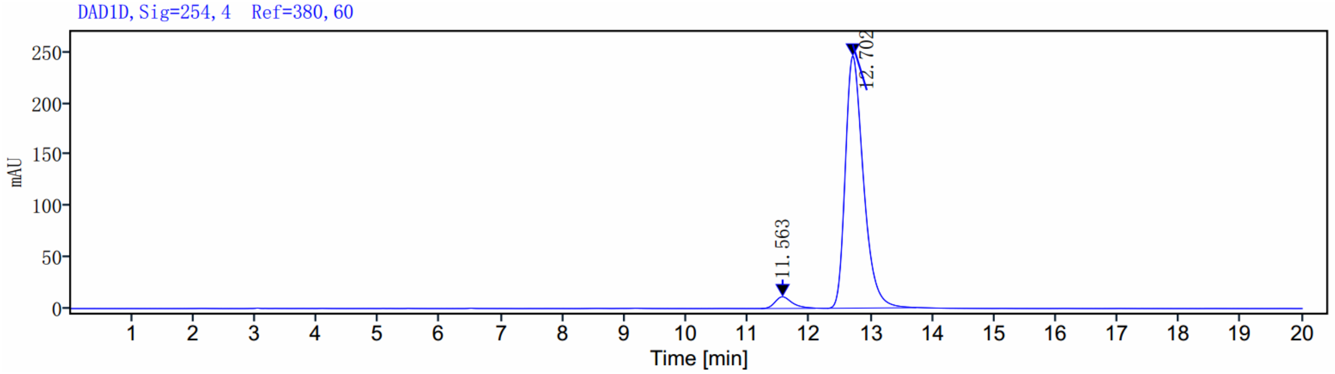

Signal: DAD1D, Sig=254, 4 Ref=380, 60

RetTime [min]	Type	Width [min]	Area [mAU*s]	Height [mAU]	Area %
11.563	VV	0.88214	221.49866	11.42179	4.2107
12.702	BV	1.44114	5038.84223	245.91511	95.7893
		Totals	5260.34089		

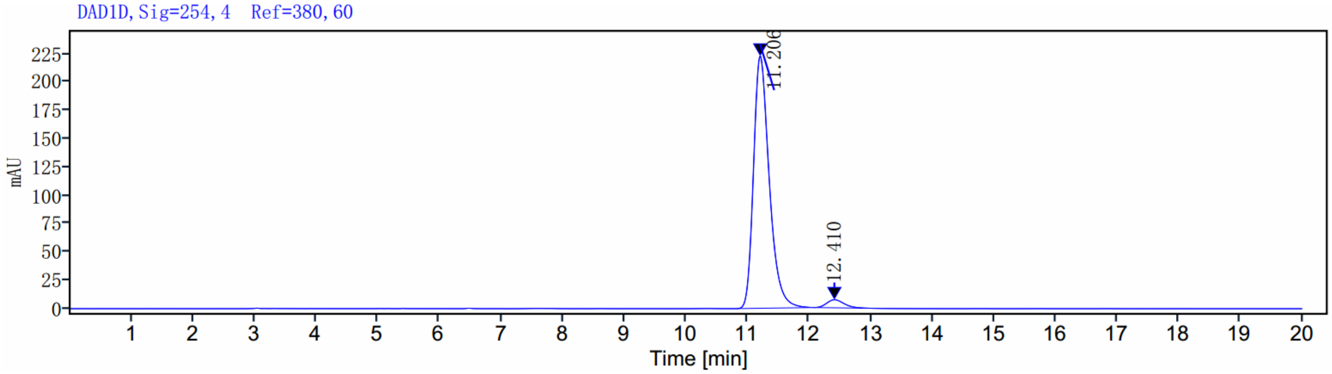

Signal: DAD1D, Sig=254, 4 Ref=380, 60

RetTime [min]	Type	Width [min]	Area [mAU*s]	Height [mAU]	Area %
11.206	VV	1.20058	3958.94350	222.75857	96.5978
12.410	BV	0.80590	139.43437	7.13533	3.4022
		Totals	4098.37787		

Supplementary Figure 108. HPLC data of **12**.

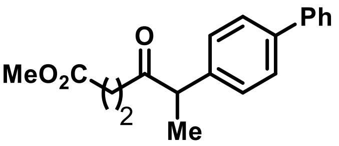

Fig. 2. (13)

(*S*)-**L**: 95% ee; (*R*)-**L**: 95% ee.

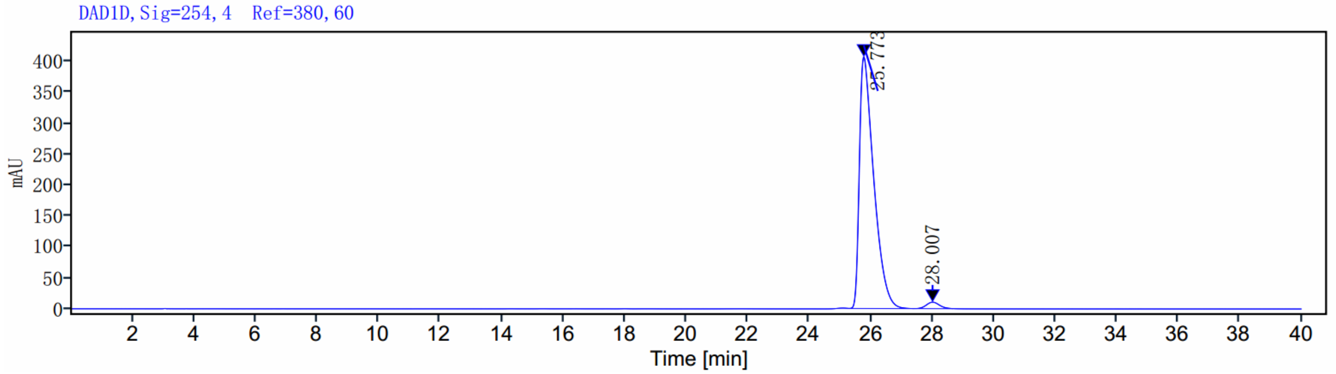

Signal: DAD1D, Sig=254, 4 Ref=380, 60

RetTime [min]	Type	Width [min]	Area [mAU*s]	Height [mAU]	Area %
25.773	BV	1.99056	13196.92733	407.55499	97.7002
28.007	VV	1.18878	310.64275	10.53385	2.2998
		Totals	13507.57008		

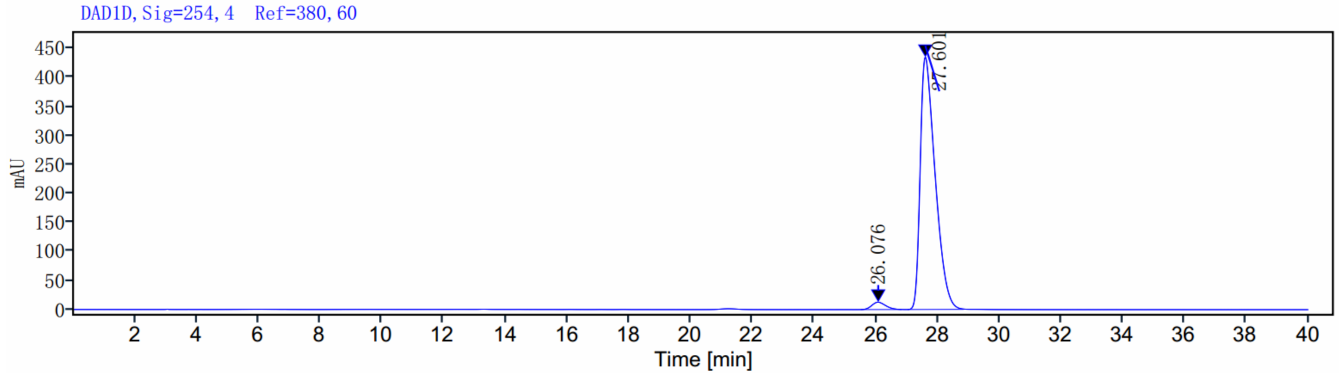

Signal: DAD1D, Sig=254, 4 Ref=380, 60

RetTime [min]	Type	Width [min]	Area [mAU*s]	Height [mAU]	Area %
26.076	VV	1.30343	394.50340	12.68906	2.6266
27.601	VV	1.81169	14625.12662	434.99503	97.3734
		Totals	15019.63002		

Supplementary Figure 109. HPLC data of **13.**

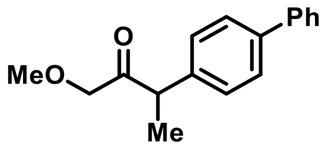

Fig. 2. (14)

(*S*)-**L**: 94% ee; (*R*)-**L**: 93% ee.

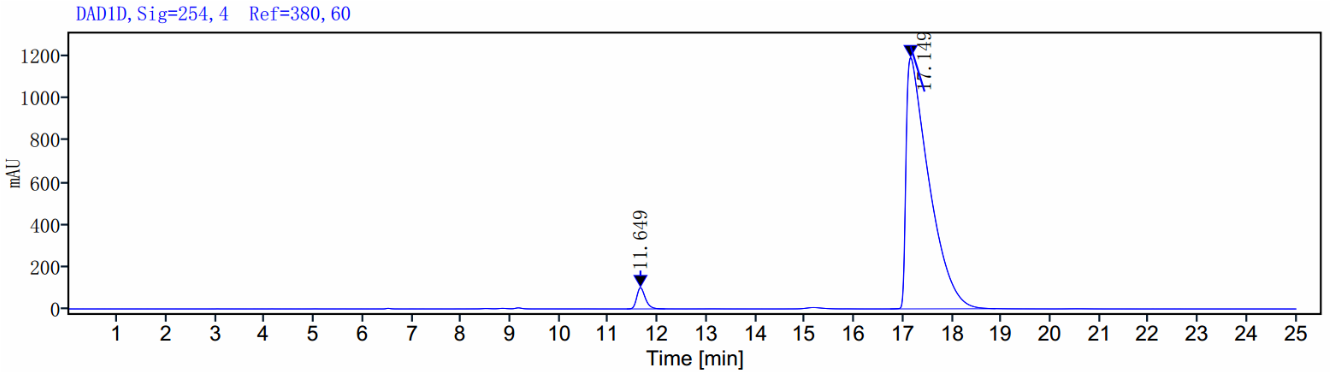

Signal: DAD1D, Sig=254, 4 Ref=380, 60

RetTime [min]	Type	Width [min]	Area [mAU*s]	Height [mAU]	Area %
11.649	VV	0.77692	1232.35697	101.01704	3.0896
17.149	VV	2.13956	38655.46268	1194.86460	96.9104
		Totals	39887.81965		

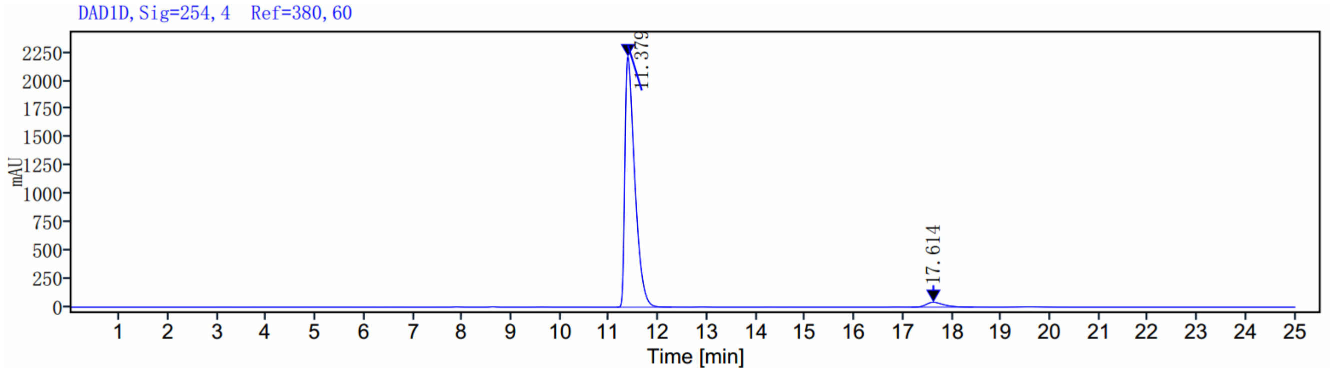

Signal: DAD1D, Sig=254, 4 Ref=380, 60

RetTime [min]	Type	Width [min]	Area [mAU*s]	Height [mAU]	Area %
11.379	BV	1.12362	31001.04425	2214.47420	96.7084
17.614	VV	1.25772	1055.15863	42.38023	3.2916
		Totals	32056.20288		

Supplementary Figure 110. HPLC data of **14.**

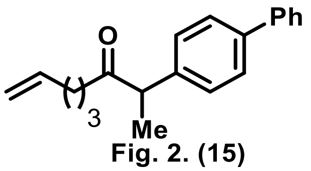

Fig. 2. (15)

(*S*)-**L**: 93% ee; (*R*)-**L**: 93% ee.

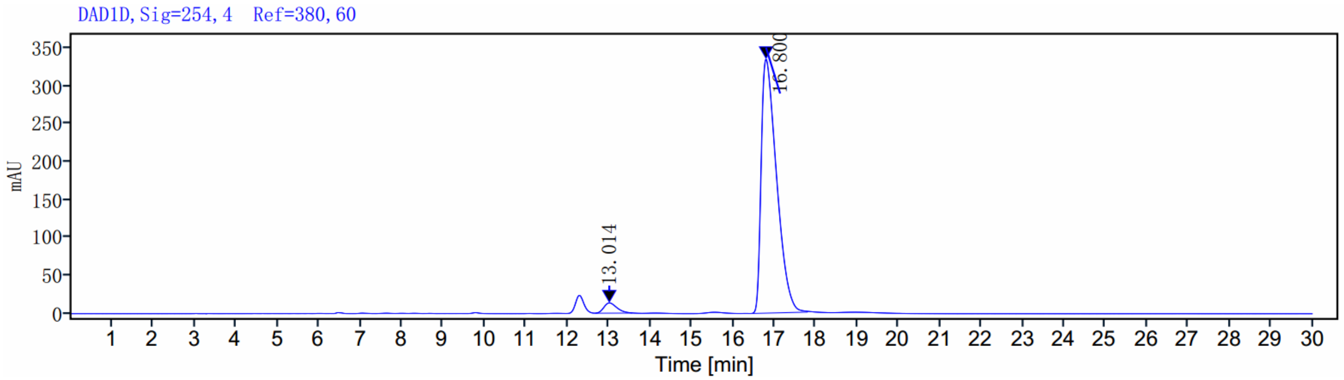

Signal: DAD1D, Sig=254, 4 Ref=380, 60

RetTime [min]	Type	Width [min]	Area [mAU*s]	Height [mAU]	Area %
13.014	VV m	0.88963	295.68666	13.53694	3.3128
16.800	VV	1.35953	8629.88515	334.63943	96.6872
		Totals	8925.57180		

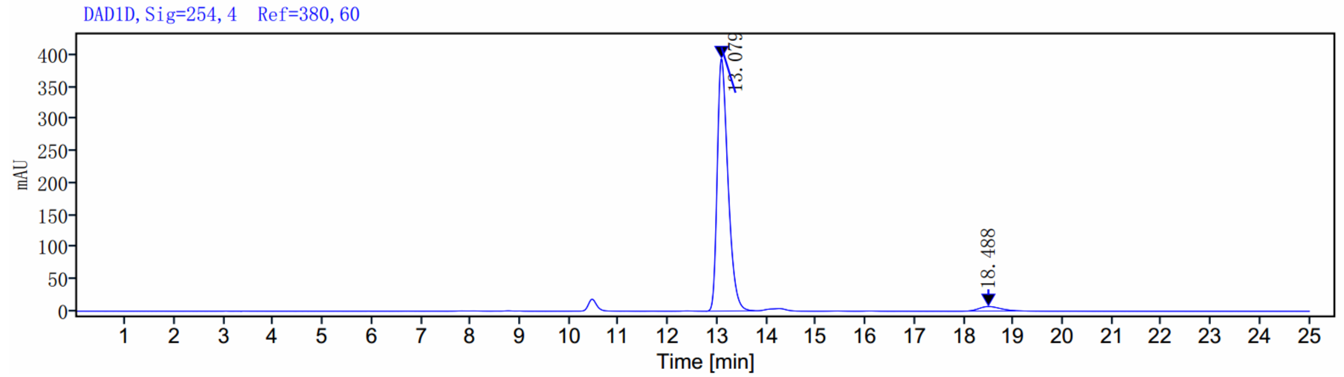

Signal: DAD1D, Sig=254, 4 Ref=380, 60

RetTime [min]	Type	Width [min]	Area [mAU*s]	Height [mAU]	Area %
13.079	VV	0.98635	5976.94748	394.55938	96.6118
18.488	VV	0.98693	209.61057	7.05793	3.3882
		Totals	6186.55805		

Supplementary Figure 111. HPLC data of **15**.

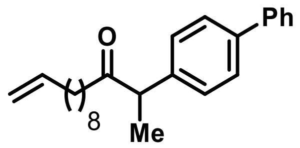

Fig. 2. (16)

(*S*)-**L**: 94% ee; (*R*)-**L**: 93% ee.

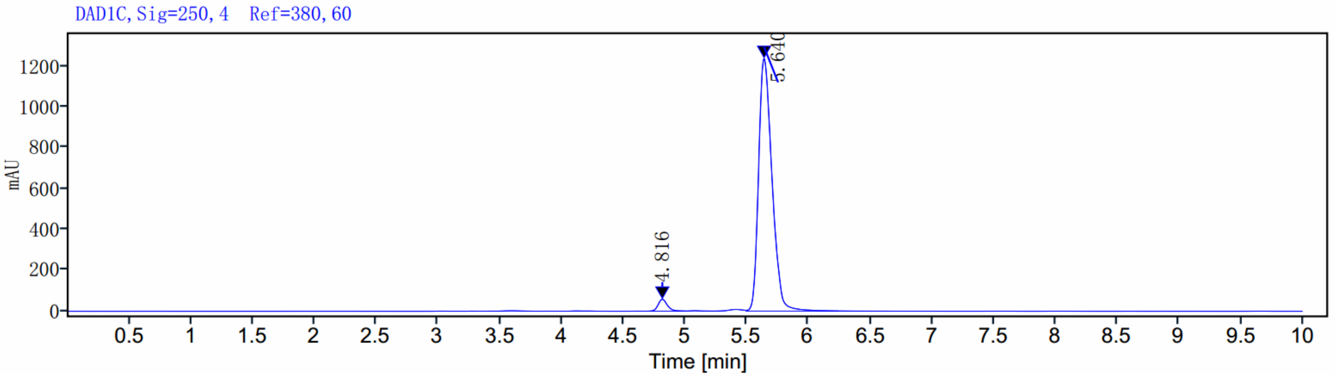

Signal: DAD1C, Sig=250, 4 Ref=380, 60

RetTime [min]	Type	Width [min]	Area [mAU*s]	Height [mAU]	Area %
4.816	BV	0.31168	302.33876	58.28438	3.1299
5.640	VV	0.75301	9357.33515	1236.81966	96.8701
		Totals	9659.67390		

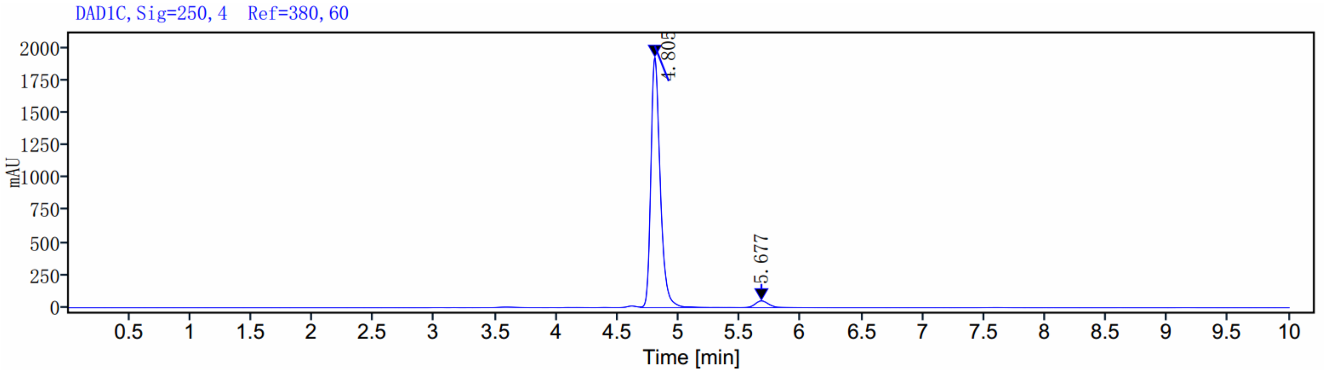

Signal: DAD1C, Sig=250, 4 Ref=380, 60

RetTime [min]	Type	Width [min]	Area [mAU*s]	Height [mAU]	Area %
4.805	VB	0.56387	10272.38487	1927.63251	96.3844
5.677	BV	0.40938	385.34207	51.19446	3.6156
		Totals	10657.72694		

Supplementary Figure 112. HPLC data of **16**.

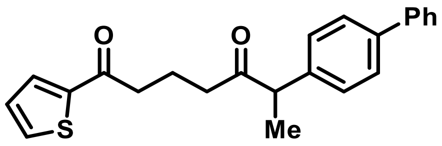

Fig. 2. (17)

(*S*)-**L**: 93% ee; (*R*)-**L**: 94% ee.

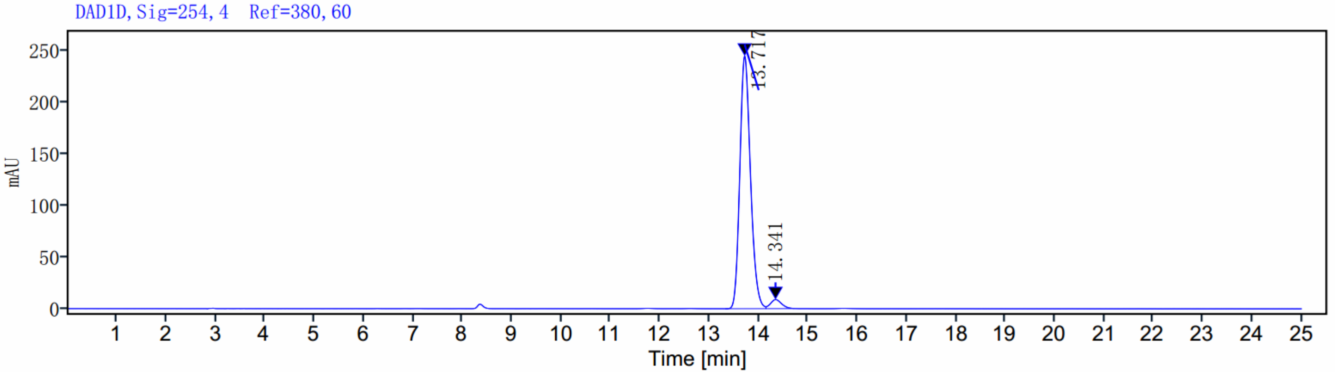

Signal: DAD1D, Sig=254, 4 Ref=380, 60

RetTime [min]	Type	Width [min]	Area [mAU*s]	Height [mAU]	Area %
13.717	BV	0.82269	3672.73286	245.37096	96.3597
14.341	VV	0.57419	138.74872	8.80861	3.6403
		Totals	3811.48158		

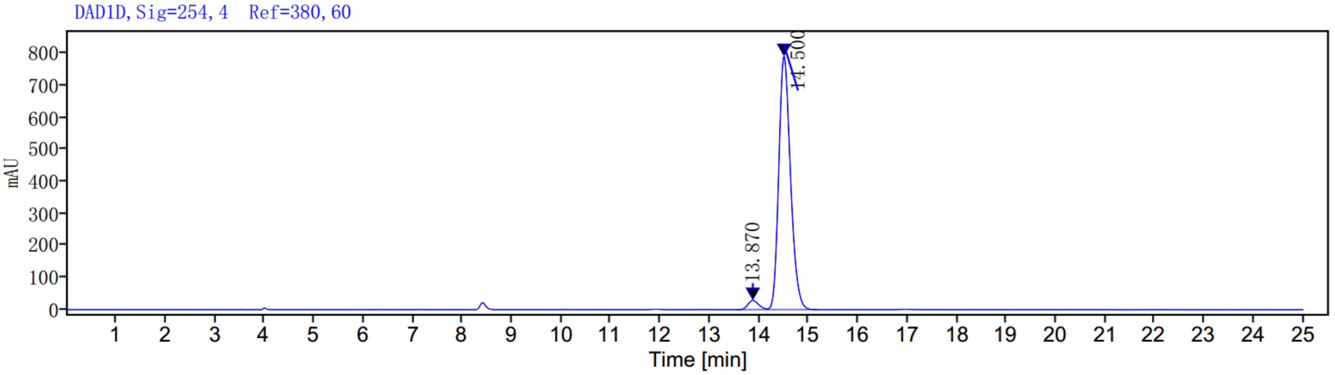

Signal: DAD1D, Sig=254, 4 Ref=380, 60

RetTime [min]	Type	Width [min]	Area [mAU*s]	Height [mAU]	Area %
13.870	VV	0.60673	439.12698	28.92875	3.2485
14.500	VV	1.01430	13078.86749	792.31672	96.7515
		Totals	13517.99447		

Supplementary Figure 113. HPLC data of **17**.

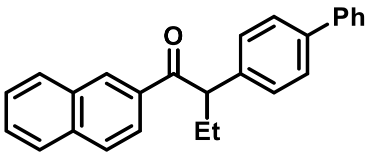

Fig. 2. (18)

(*S*, *R*)-**L**: 69% ee; (*R*, *S*)-**L**: 71% ee.

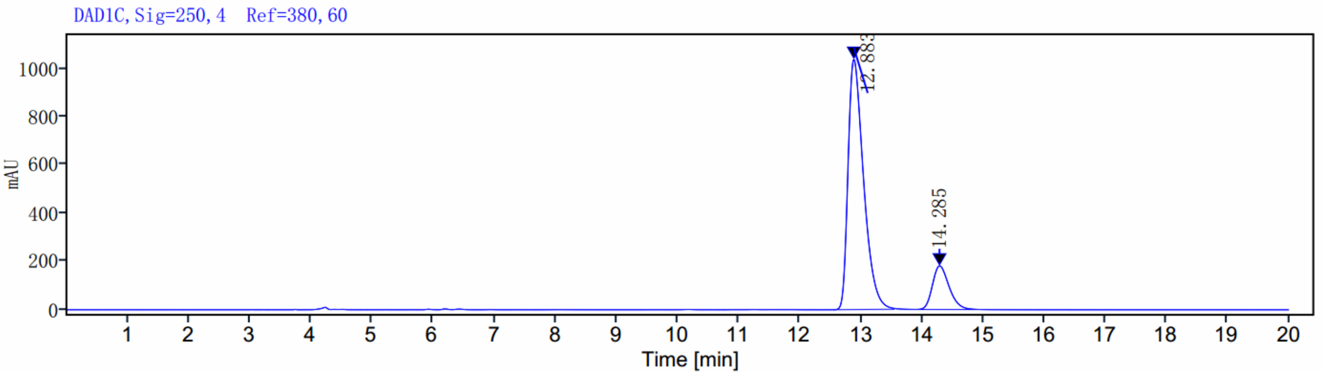

Signal: DAD1C, Sig=250, 4 Ref=380, 60

RetTime [min]	Type	Width [min]	Area [mAU*s]	Height [mAU]	Area %
12.883	BV	0.99417	18303.00141	1037.68877	84.2753
14.285	BV	0.99169	3415.11876	180.79039	15.7247
		Totals	21718.12017		

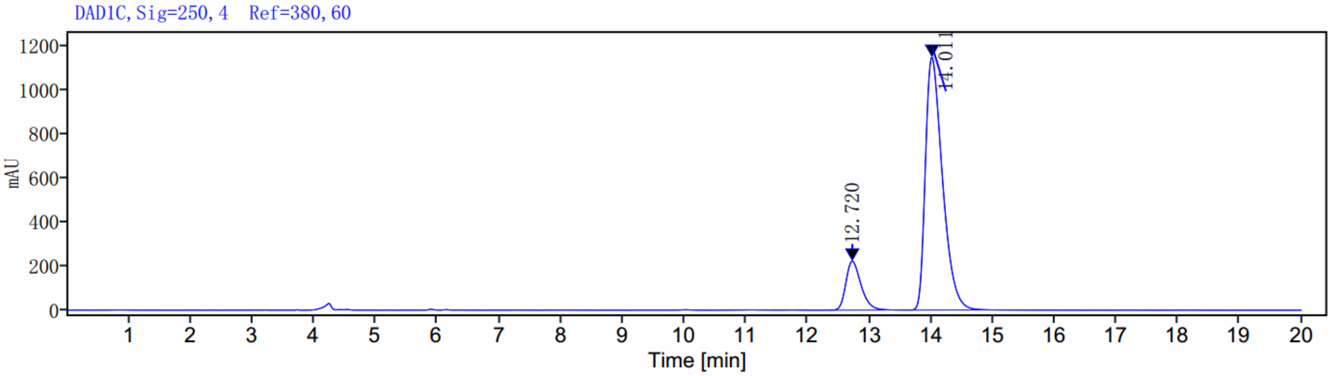

Signal: DAD1C, Sig=250, 4 Ref=380, 60

RetTime [min]	Type	Width [min]	Area [mAU*s]	Height [mAU]	Area %
12.720	VV	0.88811	3787.59935	223.09153	14.5995
14.011	VV	1.16747	22155.80512	1152.53147	85.4005
		Totals	25943.40448		

Supplementary Figure 114. HPLC data of **18**

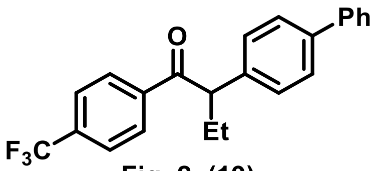

Fig. 2. (19)

(*S*, *R*)-**L**: 76% ee; (*R*, *S*)-**L**: 77% ee

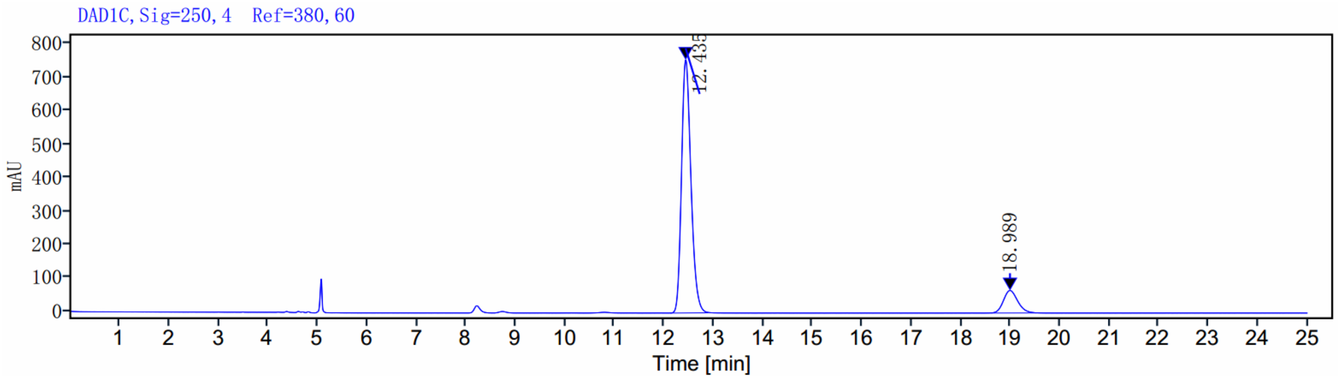

Signal: DAD1C, Sig=250, 4 Ref=380, 60

RetTime [min]	Type	Width [min]	Area [mAU*s]	Height [mAU]	Area %
12.435	VV	0.75368	10026.63624	757.67619	88.1646
18.989	VV	0.91107	1345.99740	67.33378	11.8354
		Totals	11372.63364		

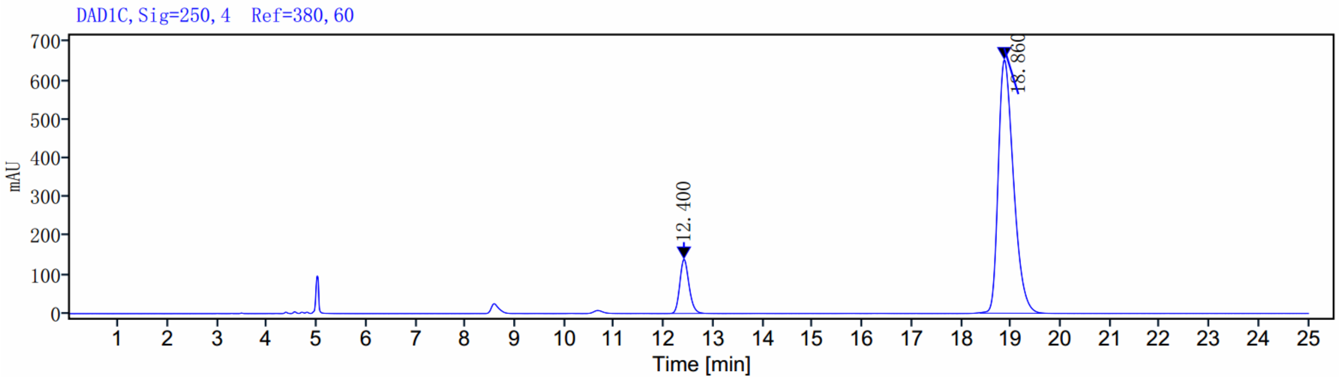

Signal: DAD1C, Sig=250, 4 Ref=380, 60

RetTime [min]	Type	Width [min]	Area [mAU*s]	Height [mAU]	Area %
12.400	VV	0.68082	1806.57269	139.32912	11.4834
18.860	VV	1.30062	13925.51226	652.72295	88.5166
		Totals	15732.08494		

Supplementary Figure 115. HPLC data of **19**

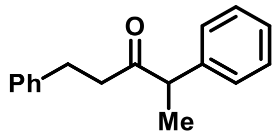

Fig. 2. (20)

(*S*)-**L**: 93% ee; (*R*)-**L**: 93% ee

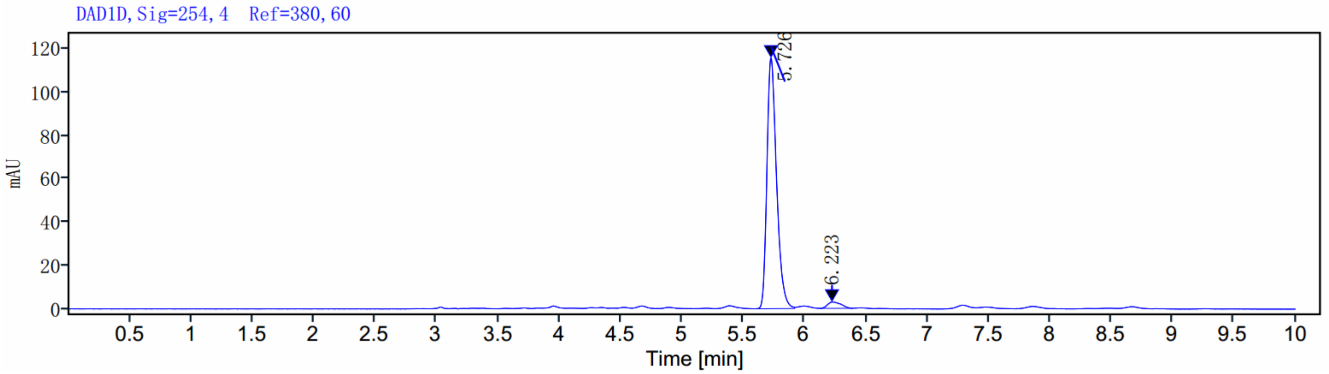

Signal: DAD1D, Sig=254, 4 Ref=380, 60

RetTime [min]	Type	Width [min]	Area [mAU*s]	Height [mAU]	Area %
5.726	BV	0.31259	631.54093	115.92513	96.5809
6.223	BB	0.26140	22.35758	2.93475	3.4191
		Totals	653.89851		

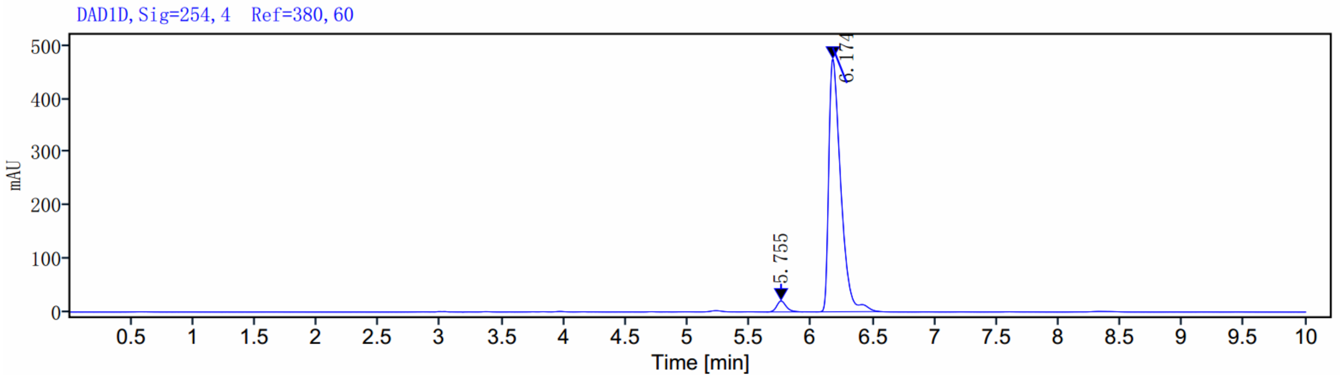

Signal: DAD1D, Sig=254, 4 Ref=380, 60

RetTime [min]	Type	Width [min]	Area [mAU*s]	Height [mAU]	Area %
5.755	BV	0.29312	110.92148	20.62088	3.3579
6.174	VM m	0.51712	3192.39848	474.52039	96.6421
		Totals	3303.31996		

Supplementary Figure 116. HPLC data of **20.**

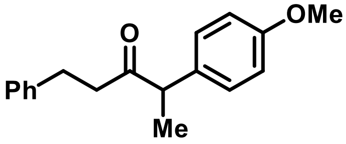

Fig. 2. (21)

(*S*)-**L**: 95% ee; (*R*)-**L**: 95% ee.

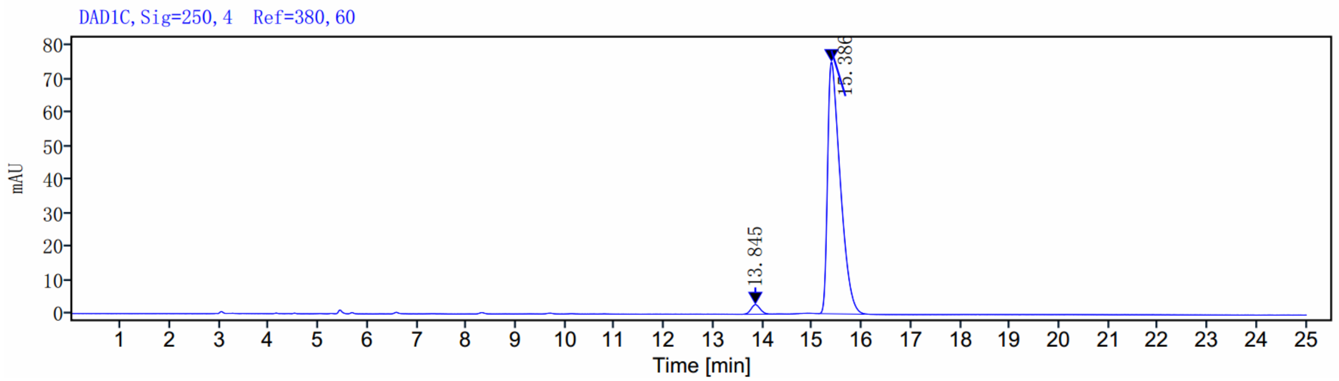

Signal: DAD1C, Sig=250, 4 Ref=380, 60

RetTime [min]	Type	Width [min]	Area [mAU*s]	Height [mAU]	Area %
13.845	VV	0.47363	36.83004	2.86490	2.6658
15.386	BV	0.92294	1344.75027	75.32656	97.3342
		Totals	1381.58032		

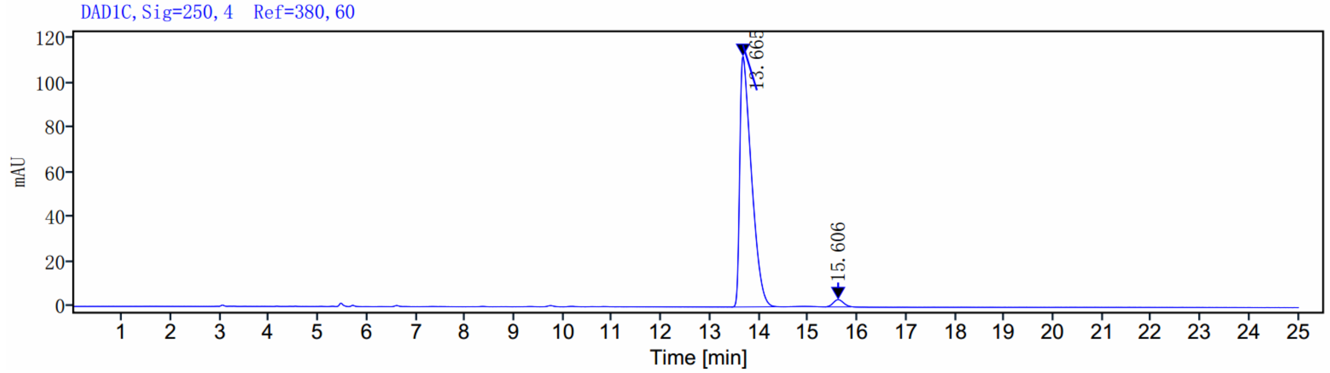

Signal: DAD1C, Sig=250, 4 Ref=380, 60

RetTime [min]	Type	Width [min]	Area [mAU*s]	Height [mAU]	Area %
13.665	BV	0.88325	1857.57167	112.18517	97.5131
15.606	BV m	0.53218	47.37472	3.26013	2.4869
		Totals	1904.94638		

Supplementary Figure 117. HPLC data of **21**.

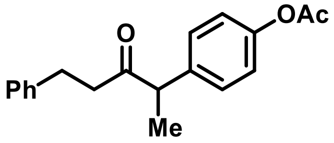

Fig. 2. (22)

(*S*)-**L**: 97% ee; (*R*)-**L**: 97% ee.

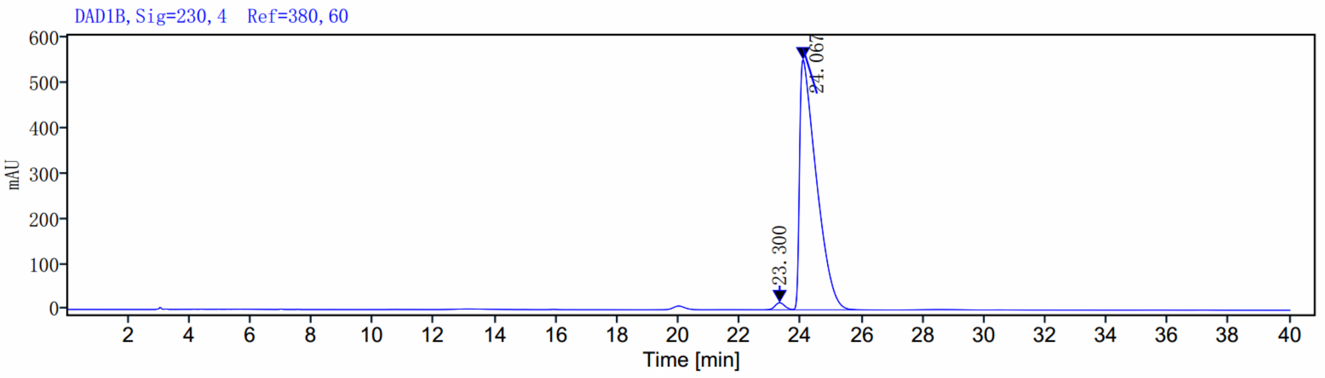

Signal: DAD1B, Sig=230, 4 Ref=380, 60

RetTime [min]	Type	Width [min]	Area [mAU*s]	Height [mAU]	Area %
23.300	BV	0.91491	344.81082	15.66692	1.6171
24.067	VV	2.04337	20977.52117	551.98639	98.3829
		Totals	21322.33199		

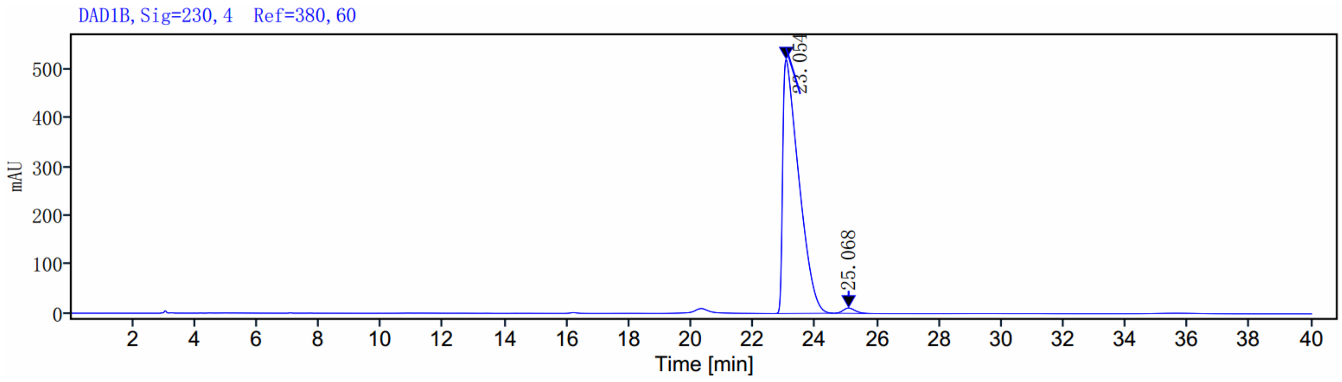

Signal: DAD1B, Sig=230, 4 Ref=380, 60

RetTime [min]	Type	Width [min]	Area [mAU*s]	Height [mAU]	Area %
23.054	VV	1.89838	18730.08638	521.19456	98.4806
25.068	VV	1.02556	288.98318	10.80650	1.5194
		Totals	19019.06956		

Supplementary Figure 118. HPLC data of **22**.

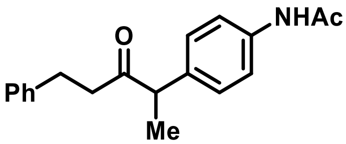

Fig. 2. (23)

(*S*)-**L**: 95% ee; (*R*)-**L**: 95% ee.

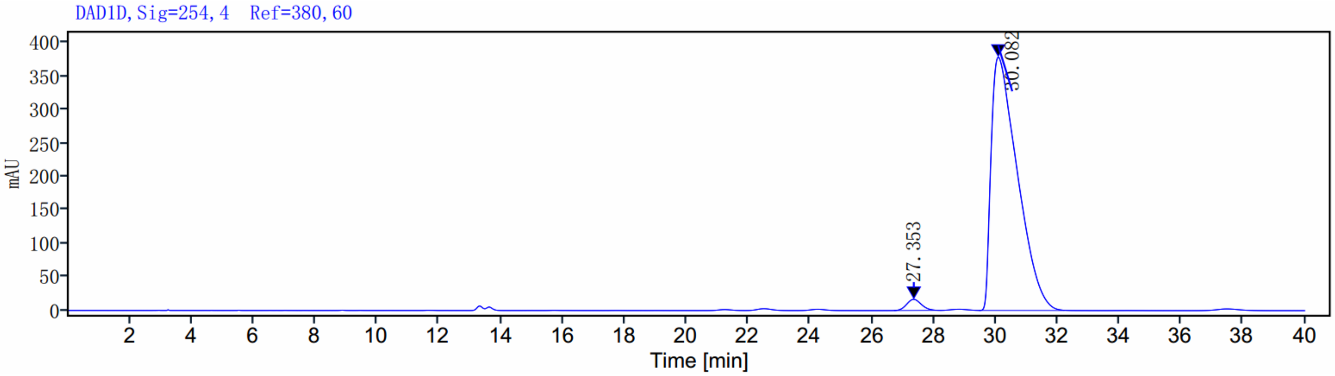

Signal: DAD1D, Sig=254, 4 Ref=380, 60

RetTime [min]	Type	Width [min]	Area [mAU*s]	Height [mAU]	Area %
27.353	BV m	1.35227	543.14601	16.57107	2.3717
30.082	VV	2.75925	22358.38870	378.56023	97.6283
		Totals	22901.53471		

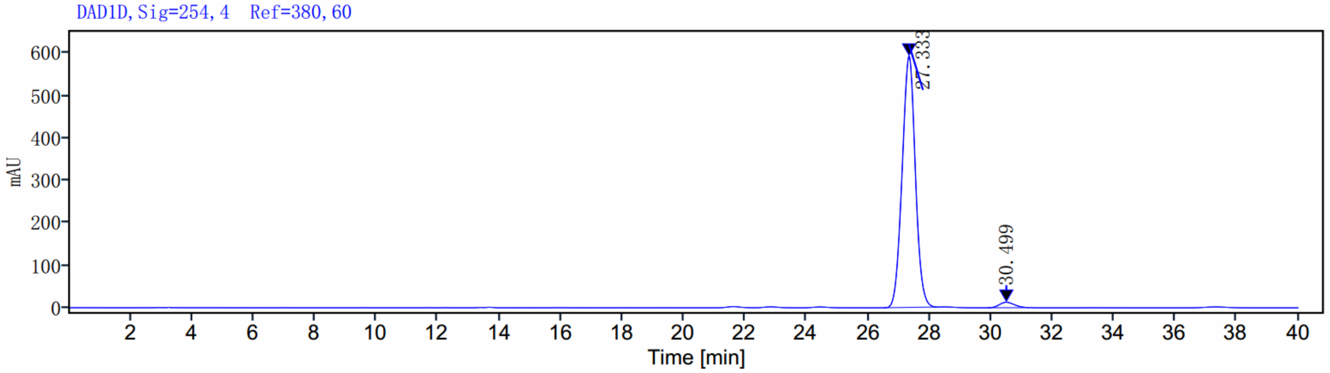

Signal: DAD1D, Sig=254, 4 Ref=380, 60

RetTime [min]	Type	Width [min]	Area [mAU*s]	Height [mAU]	Area %
27.333	VV	1.71729	17722.21358	592.78106	97.5729
30.499	VV m	1.33037	440.83723	12.87626	2.4271
		Totals	18163.05081		

Supplementary Figure 119. HPLC data of **23.**

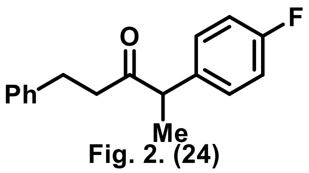

Fig. 2. (24)

(*S*)-**L**: 93% ee; (*R*)-**L**: 93% ee.

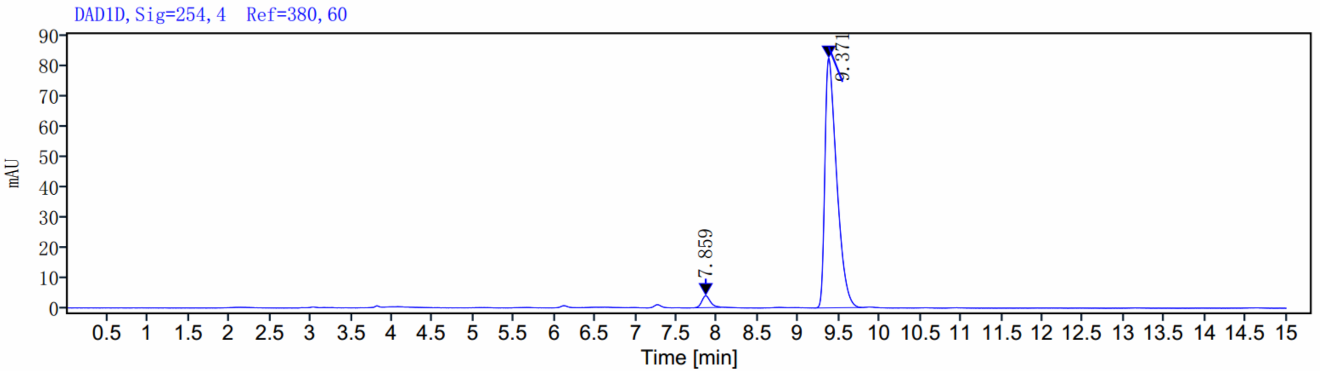

Signal: DAD1D, Sig=254, 4 Ref=380, 60

RetTime [min]	Type	Width [min]	Area [mAU*s]	Height [mAU]	Area %
7.859	BV	0.34306	28.99387	3.94134	3.3676
9.371	VV	0.56878	831.97719	82.39153	96.6324
		Totals	860.97107		

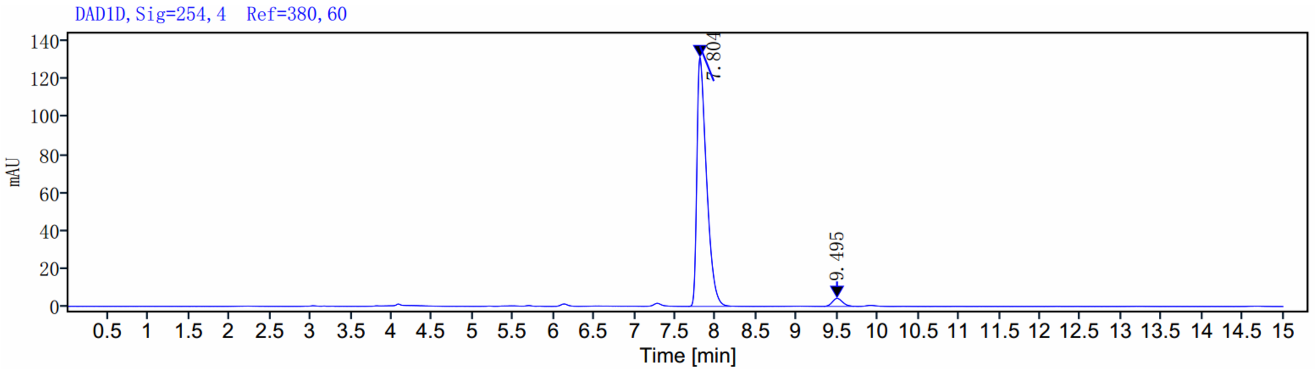

Signal: DAD1D, Sig=254, 4 Ref=380, 60

RetTime [min]	Type	Width [min]	Area [mAU*s]	Height [mAU]	Area %
7.804	VV	0.52531	1090.43056	131.76680	96.6922
9.495	VV	0.36842	37.30279	4.25020	3.3078
		Totals	1127.73335		

Supplementary Figure 120. HPLC data of **24**.

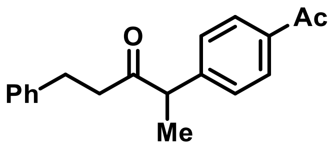

Fig. 2. (25)

(*S*)-**L**: 92% ee; (*R*)-**L**: 90% ee.

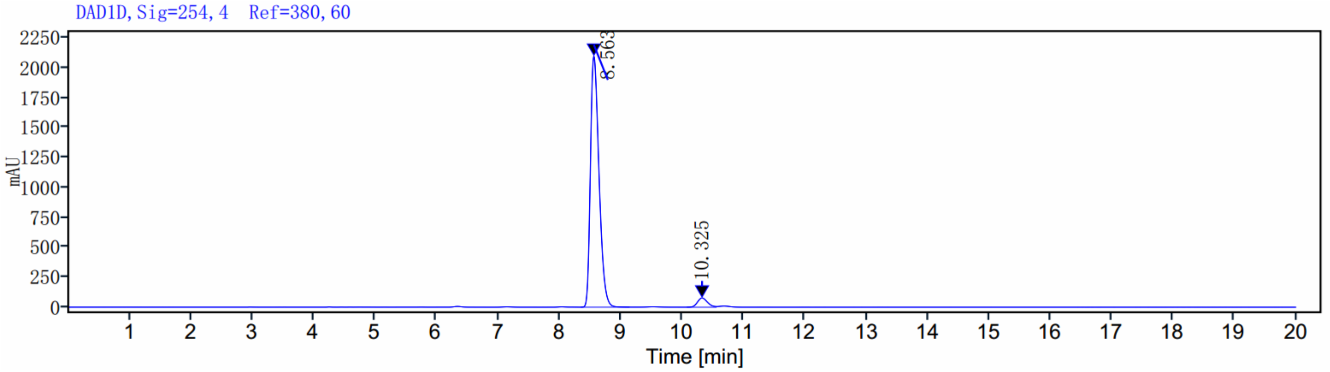

Signal: DAD1D, Sig=254, 4 Ref=380, 60

RetTime [min]	Type	Width [min]	Area [mAU*s]	Height [mAU]	Area %
8.563	BV	0.79444	20367.75509	2096.44682	95.9070
10.325	BV	0.48241	869.23251	76.61677	4.0930
		Totals	21236.98760		

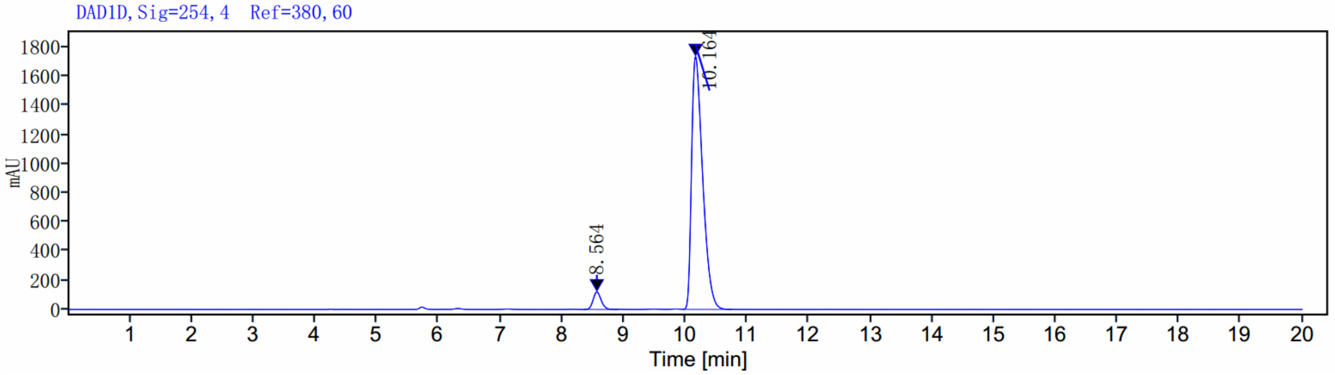

Signal: DAD1D, Sig=254, 4 Ref=380, 60

RetTime [min]	Type	Width [min]	Area [mAU*s]	Height [mAU]	Area %
8.564	BV	0.55445	1100.11379	120.25369	4.8403
10.164	VV	0.79119	21628.21436	1737.51234	95.1597
		Totals	22728.32815		

Supplementary Figure 121. HPLC data of **25.**

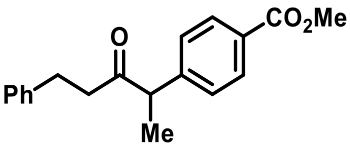

Fig. 2. (26)

(*S*)-**L**: 92% ee; (*R*)-**L**: 90% ee.

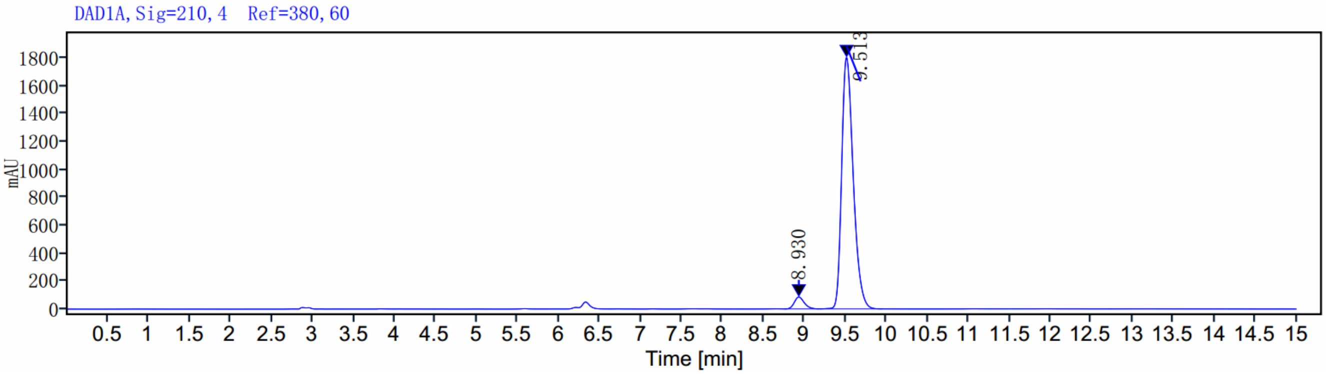

Signal: DAD1A, Sig=210, 4 Ref=380, 60

RetTime [min]	Type	Width [min]	Area [mAU*s]	Height [mAU]	Area %
8.930	BV	0.41680	718.77555	85.37334	3.9148
9.513	VV	0.76365	17641.84136	1800.32278	96.0852
		Totals	18360.61692		

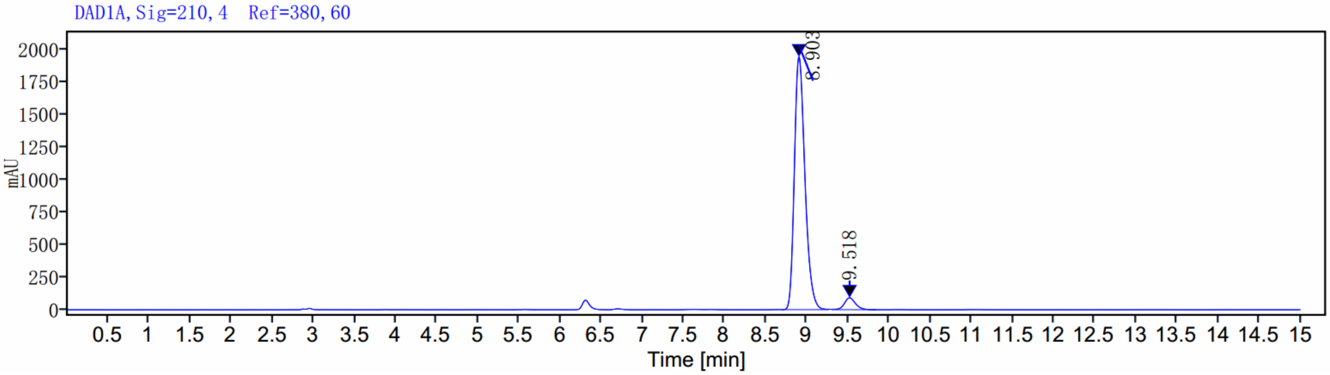

Signal: DAD1A, Sig=210, 4 Ref=380, 60

RetTime [min]	Type	Width [min]	Area [mAU*s]	Height [mAU]	Area %
8.903	BV	0.56810	17344.44760	1939.38161	95.2467
9.518	VB	0.53753	865.58428	91.43625	4.7533
		Totals	18210.03188		

Supplementary Figure 122. HPLC data of **26.**

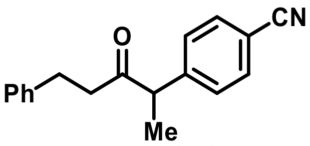

Fig. 2. (27)

(*S*)-**L**: 88% ee; (*R*)-**L**: 88% ee.

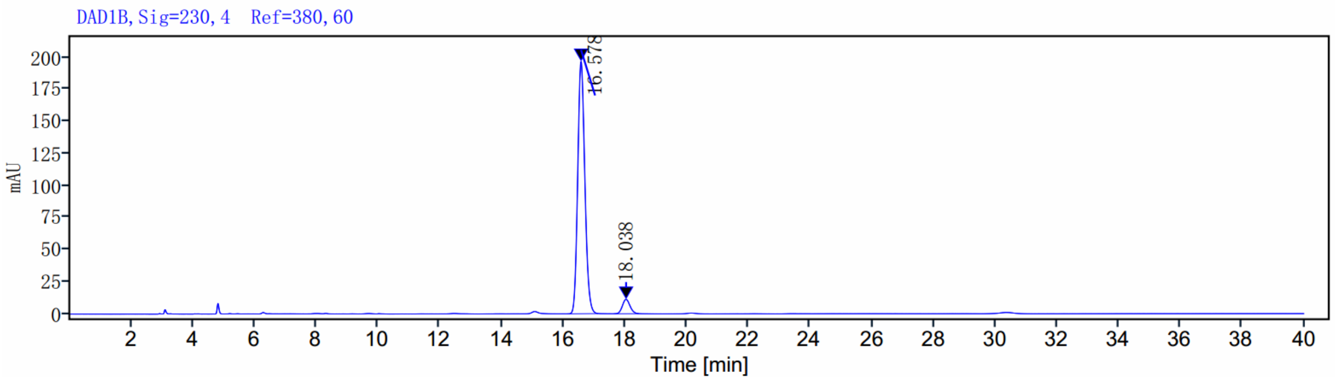

Signal: DAD1B, Sig=230, 4 Ref=380, 60

RetTime [min]	Type	Width [min]	Area [mAU*s]	Height [mAU]	Area %
16.578	VV	0.99053	3149.68937	196.70841	94.2230
18.038	VV	0.80487	193.11255	11.24619	5.7770
		Totals	3342.80192		

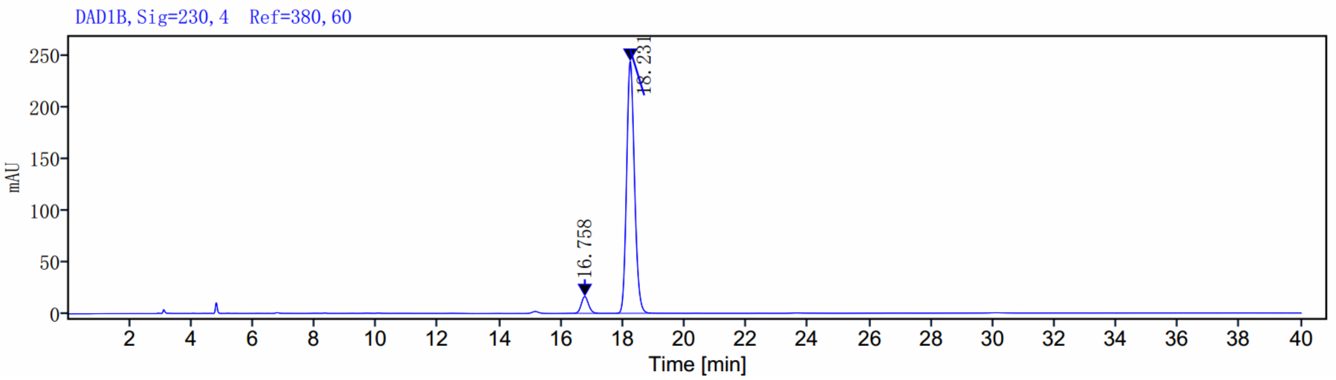

Signal: DAD1B, Sig=230, 4 Ref=380, 60

RetTime [min]	Type	Width [min]	Area [mAU*s]	Height [mAU]	Area %
16.758	VV	0.72922	265.41606	16.21686	5.7834
18.231	VV	1.09205	4323.85429	243.86969	94.2166
		Totals	4589.27035		

Supplementary Figure 123. HPLC data of **27**.

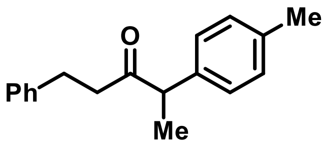

Fig. 2. (28)

(*S*)-**L**: 93% ee; (*R*)-**L**: 95% ee.

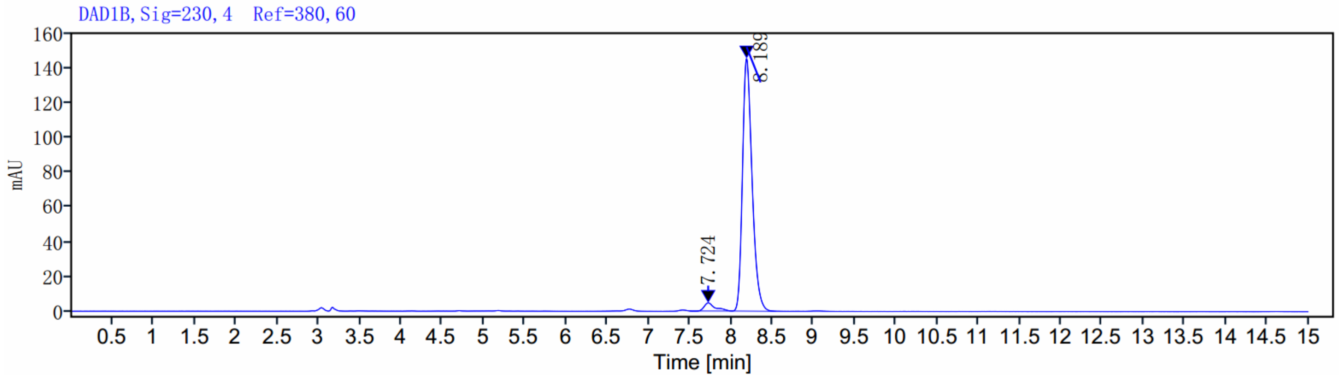

Signal: DAD1B, Sig=230, 4 Ref=380, 60

RetTime [min]	Type	Width [min]	Area [mAU*s]	Height [mAU]	Area %
7.724	MM m	0.51072	42.78207	4.68186	3.4839
8.189	BV	0.53651	1185.22677	146.06134	96.5161
		Totals	1228.00884		

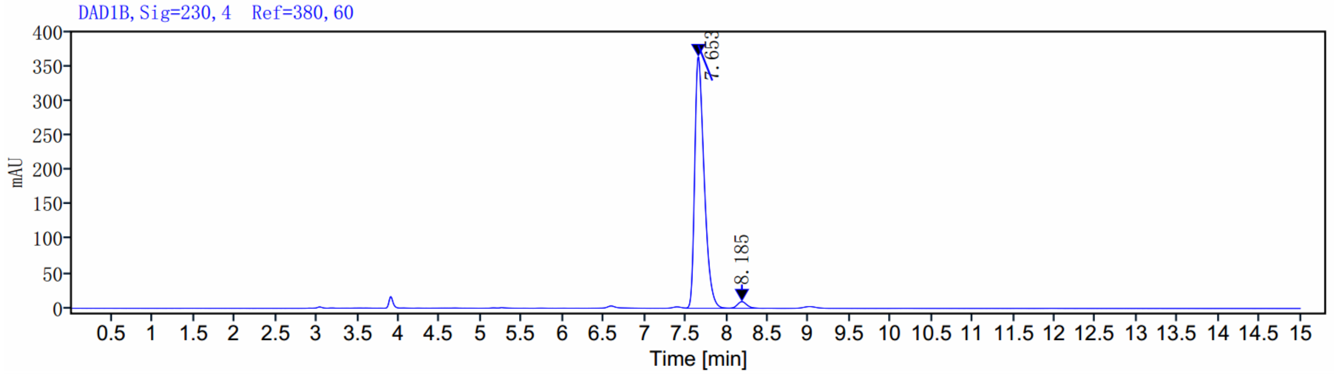

Signal: DAD1B, Sig=230, 4 Ref=380, 60

RetTime [min]	Type	Width [min]	Area [mAU*s]	Height [mAU]	Area %
7.653	VV	0.53193	2923.40062	363.76215	97.4189
8.185	MM m	0.42257	77.45386	9.66313	2.5811
		Totals	3000.85448		

Supplementary Figure 124. HPLC data of **28**.

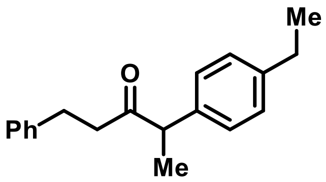

Fig. 2. (29)

(*S*)-**L**: 95% ee; (*R*)-**L**: 94% ee.

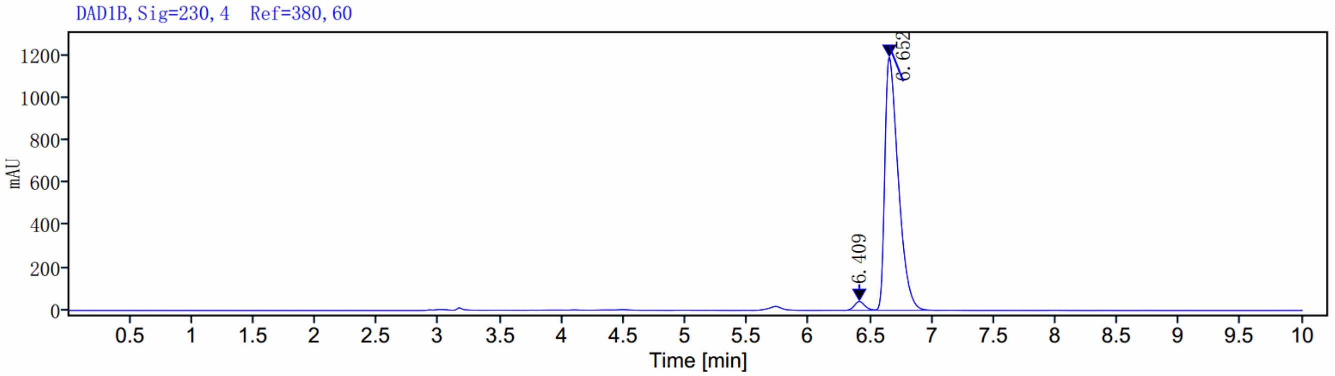

Signal: DAD1B, Sig=230, 4 Ref=380, 60

RetTime [min]	Type	Width [min]	Area [mAU*s]	Height [mAU]	Area %
6.409	BV	0.25410	240.73429	41.14675	2.6070
6.652	VB	0.52424	8993.45955	1189.58128	97.3930
		Totals	9234.19384		

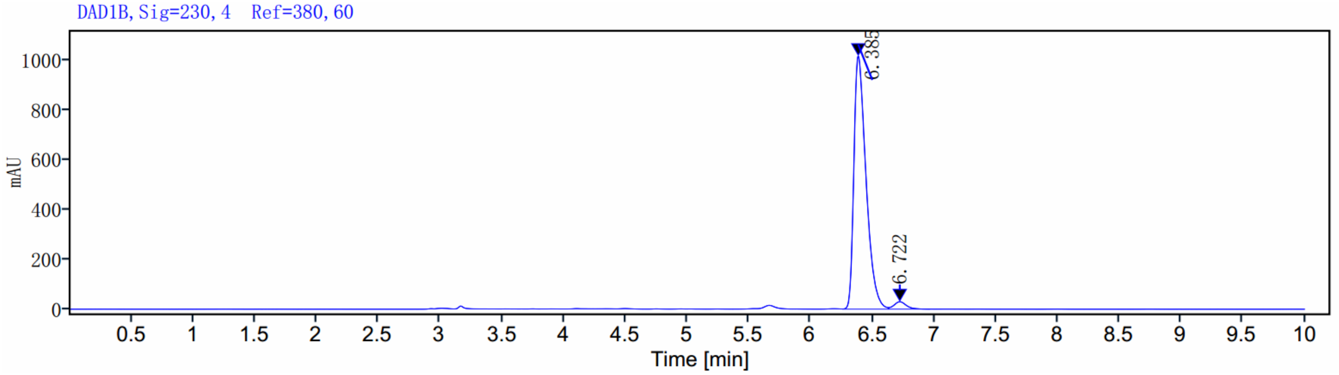

Signal: DAD1B, Sig=230, 4 Ref=380, 60

RetTime [min]	Type	Width [min]	Area [mAU*s]	Height [mAU]	Area %
6.385	VV	0.37262	6964.38498	1020.90612	97.1467
6.722	VB	0.33466	204.55052	29.81346	2.8533
		Totals	7168.93550		

Supplementary Figure 125. HPLC data of **29**.

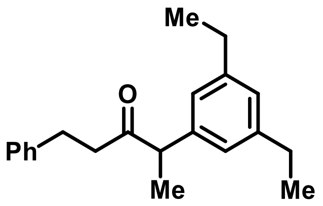

Fig. 2. (30)

(*S*)-**L**: 93% ee; (*R*)-**L**: 93% ee.

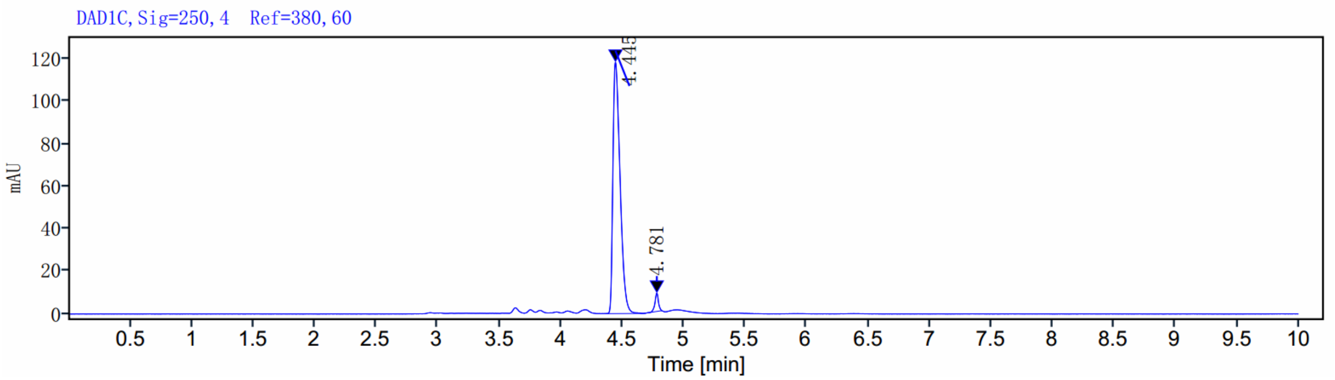

Signal: DAD1C, Sig=250, 4 Ref=380, 60

RetTime [min]	Type	Width [min]	Area [mAU*s]	Height [mAU]	Area %
4.445	VB	0.30592	477.18458	118.21080	96.5862
4.781	MM m	0.15212	16.86599	8.55734	3.4138
		Totals	494.05057		

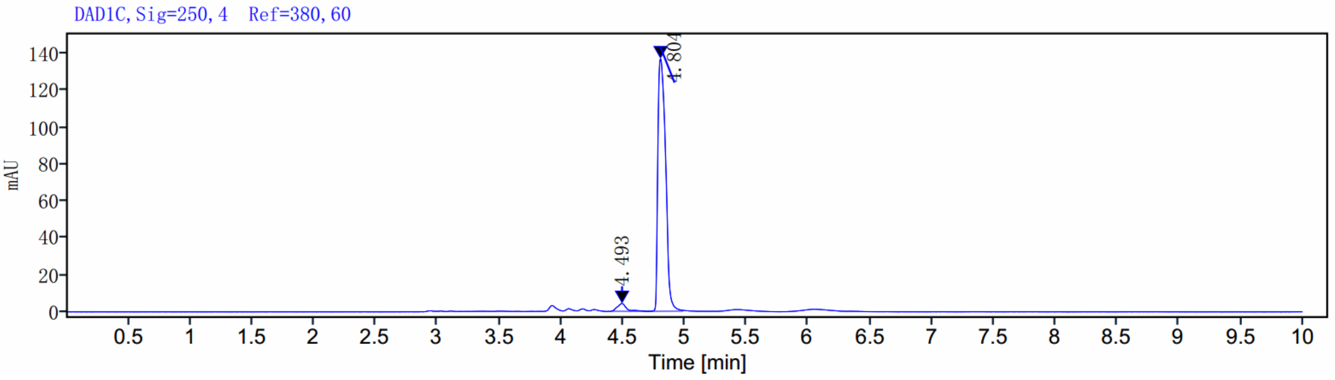

Signal: DAD1C, Sig=250, 4 Ref=380, 60

RetTime [min]	Type	Width [min]	Area [mAU*s]	Height [mAU]	Area %
4.493	MM m	0.31919	21.42219	4.18523	3.4703
4.804	VV	0.25997	595.87364	137.01025	96.5297
		Totals	617.29583		

Supplementary Figure 126. HPLC data of **30**.

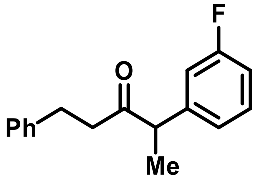

Fig. 2. (31)

(*S*)-**L**: 93% ee; (*R*)-**L**: 93% ee.

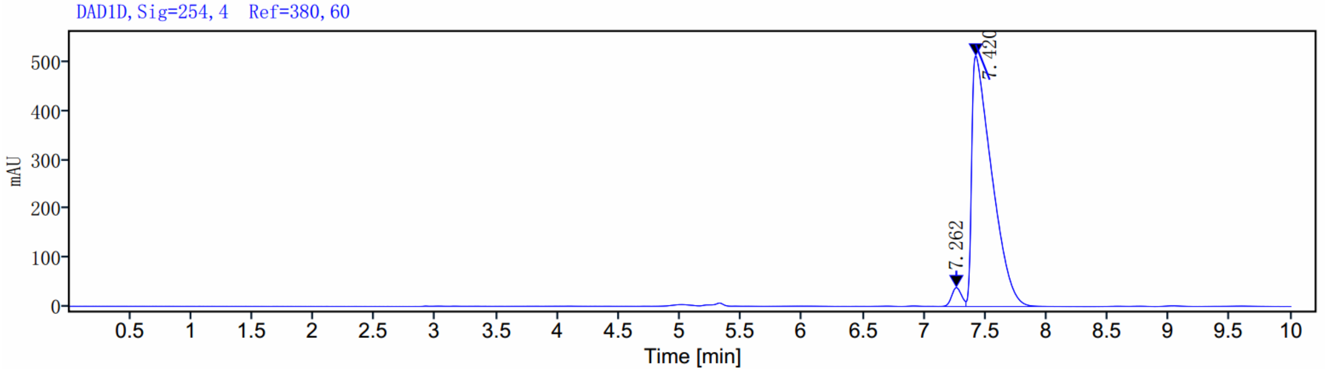

Signal: DAD1D, Sig=254, 4 Ref=380, 60

RetTime [min]	Type	Width [min]	Area [mAU*s]	Height [mAU]	Area %
7.262	MM m	0.27864	217.19625	38.58698	3.5703
7.420	VV	0.62661	5866.26655	514.49555	96.4297
		Totals	6083.46279		

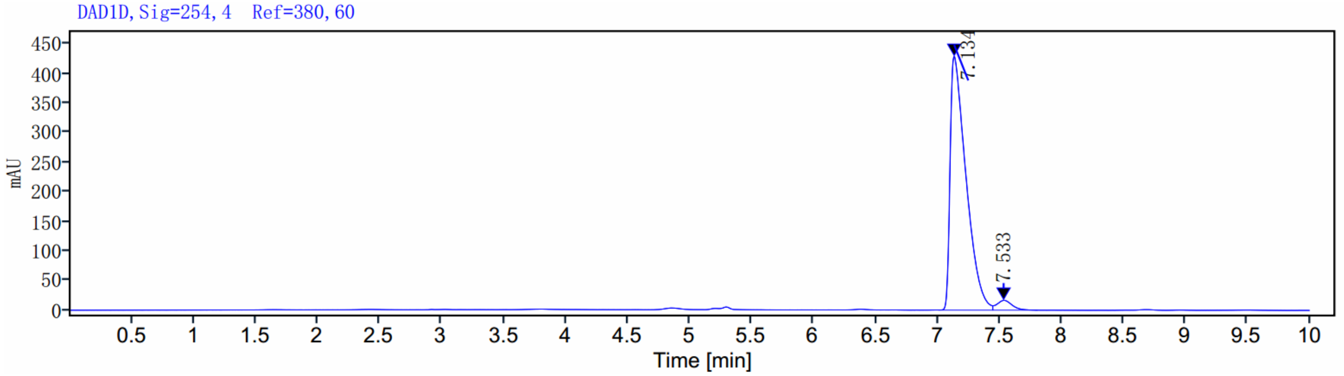

Signal: DAD1D, Sig=254, 4 Ref=380, 60

RetTime [min]	Type	Width [min]	Area [mAU*s]	Height [mAU]	Area %
7.134	VV	0.43864	3818.91360	428.62109	96.4469
7.533	MM m	0.36189	140.68706	16.52736	3.5531
		Totals	3959.60066		

Supplementary Figure 127. HPLC data of **31**.

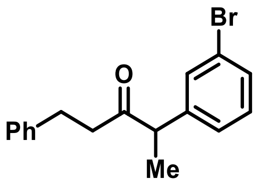

Fig. 2. (32)

(*S*)-**L**: 93% ee; (*R*)-**L**: 94% ee.

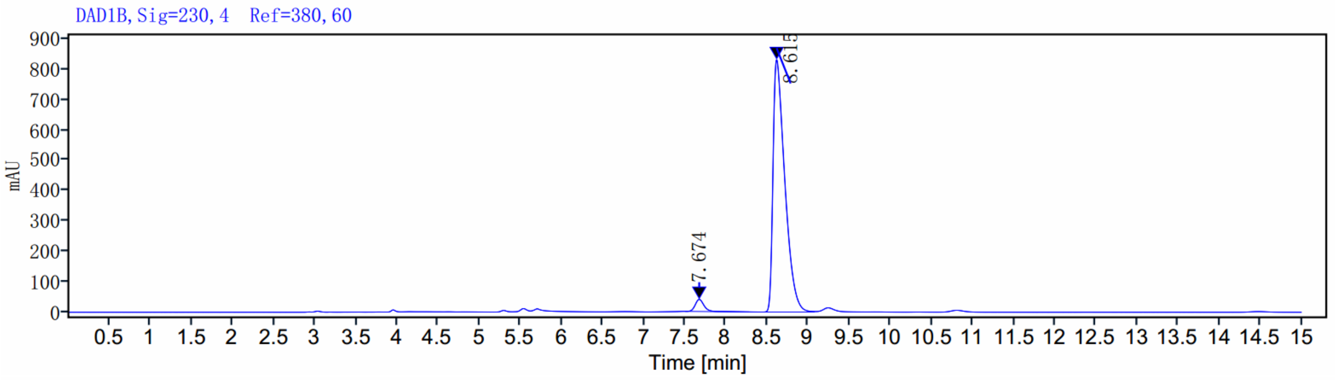

Signal: DAD1B, Sig=230, 4 Ref=380, 60

RetTime [min]	Type	Width [min]	Area [mAU*s]	Height [mAU]	Area %
7.674	BV	0.78047	301.20068	39.81910	3.5097
8.615	BV	0.61845	8280.70751	830.87770	96.4903
		Totals	8581.90819		

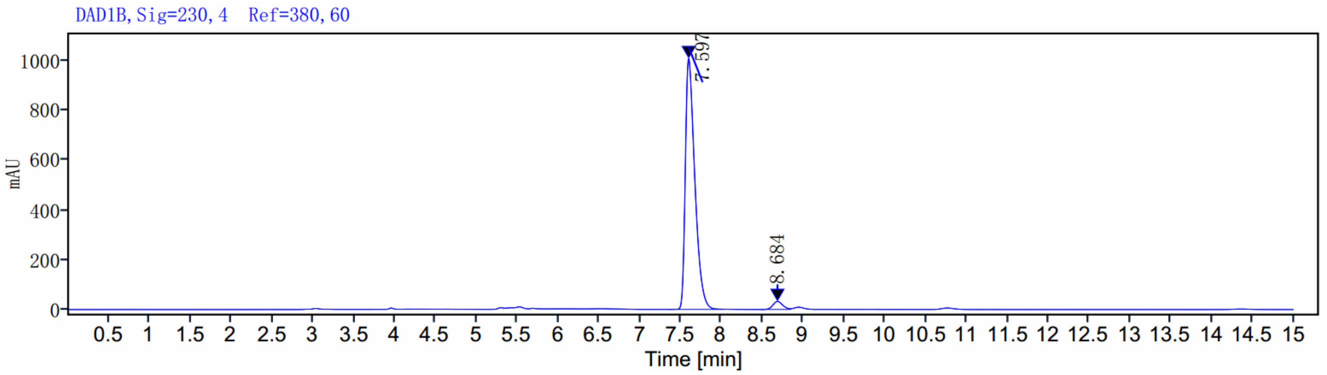

Signal: DAD1B, Sig=230, 4 Ref=380, 60

RetTime [min]	Type	Width [min]	Area [mAU*s]	Height [mAU]	Area %
7.597	VV	0.69747	8180.53697	1010.94768	96.7944
8.684	BV	0.36491	270.92062	32.15198	3.2056
		Totals	8451.45759		

Supplementary Figure 128. HPLC data of **32**.

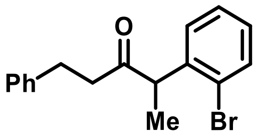

Fig. 2. (33)

(*S*)-**L**: 91% ee; (*R*)-**L**: 90% ee.

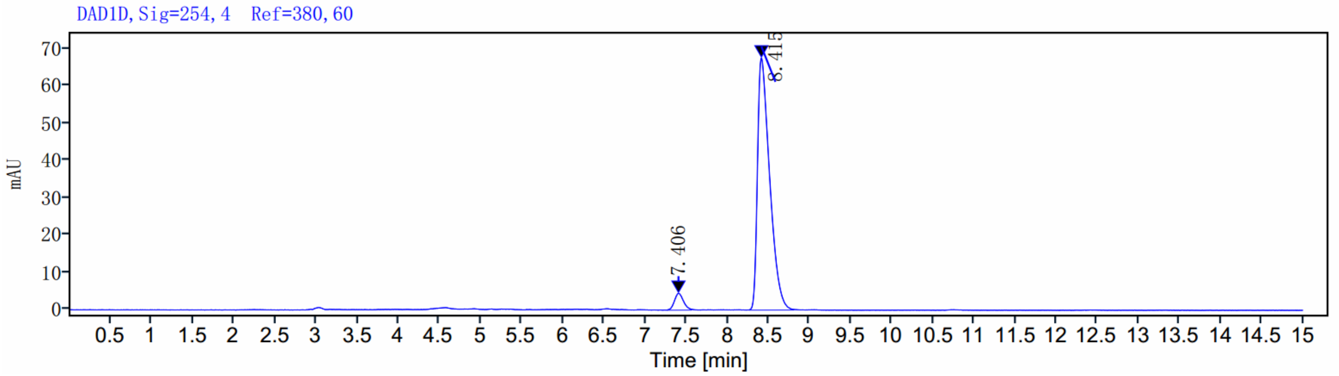

Signal: DAD1D, Sig=254, 4 Ref=380, 60

RetTime [min]	Type	Width [min]	Area [mAU*s]	Height [mAU]	Area %
7.406	VV	0.29934	34.30380	4.48969	4.7115
8.415	VV	0.54692	693.78346	67.53882	95.2885
		Totals	728.08726		

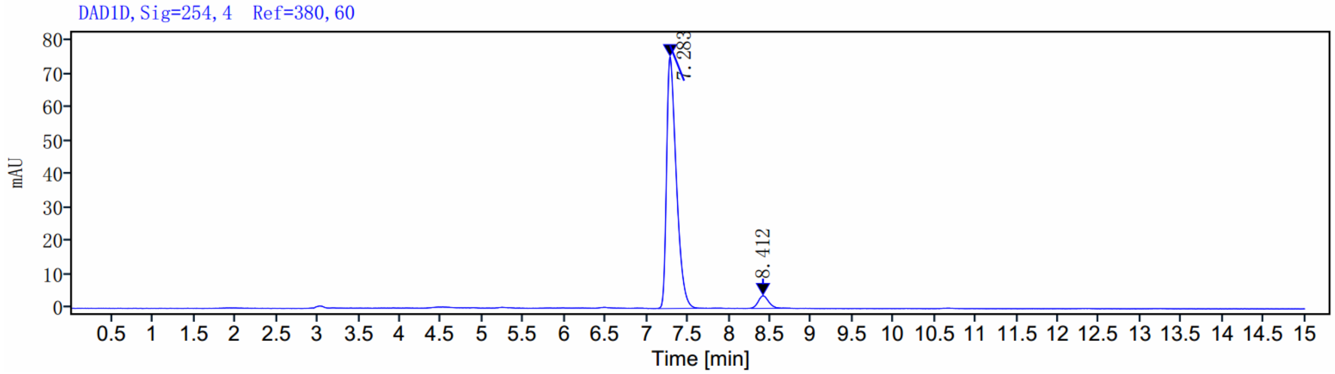

Signal: DAD1D, Sig=254, 4 Ref=380, 60

RetTime [min]	Type	Width [min]	Area [mAU*s]	Height [mAU]	Area %
7.283	VV	0.47467	619.04790	75.31768	94.9154
8.412	VV	0.34837	33.16253	3.78040	5.0846
		Totals	652.21043		

Supplementary Figure 129. HPLC data of **33**.

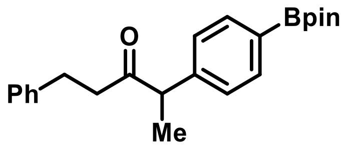

Fig. 2. (34)

(*S*)-**L**: 94% ee; (*R*)-**L**: 94% ee.

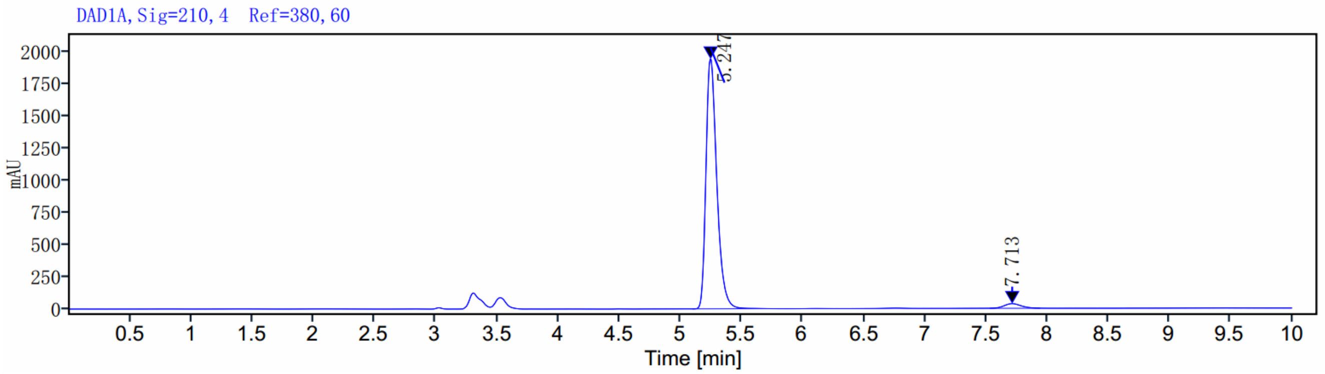

Signal: DAD1A, Sig=210, 4 Ref=380, 60

RetTime [min]	Type	Width [min]	Area [mAU*s]	Height [mAU]	Area %
5.247	BV	0.56535	11997.66736	1946.73808	97.1773
7.713	VV	0.41149	348.49563	35.80139	2.8227
		Totals	12346.16299		

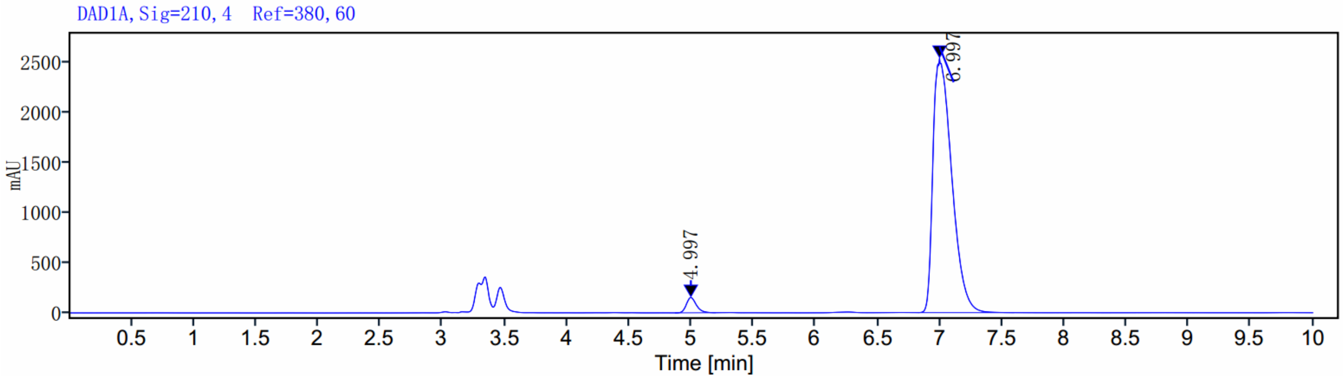

Signal: DAD1A, Sig=210, 4 Ref=380, 60

RetTime [min]	Type	Width [min]	Area [mAU*s]	Height [mAU]	Area %
4.997	BB	0.32765	852.43159	152.36659	3.0941
6.997	BV m	0.77259	26697.99538	2546.84777	96.9059
		Totals	27550.42697		

Supplementary Figure 130. HPLC data of **34**.

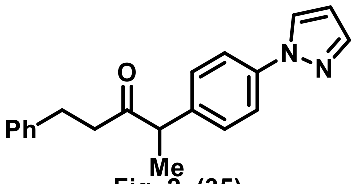

Fig. 2. (35)

(*S*)-**L**: 95% ee; (*R*)-**L**: 94% ee.

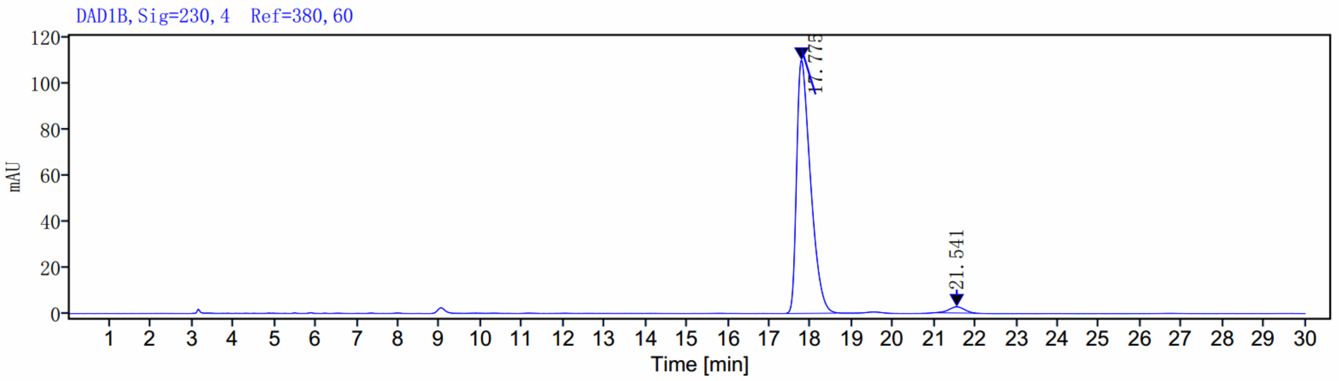

Signal: DAD1B, Sig=230, 4 Ref=380, 60

RetTime [min]	Type	Width [min]	Area [mAU*s]	Height [mAU]	Area %
17.775	VV	1.23949	2590.06163	110.60809	97.3649
21.541	MV m	0.97517	70.09729	2.69055	2.6351
		Totals	2660.15893		

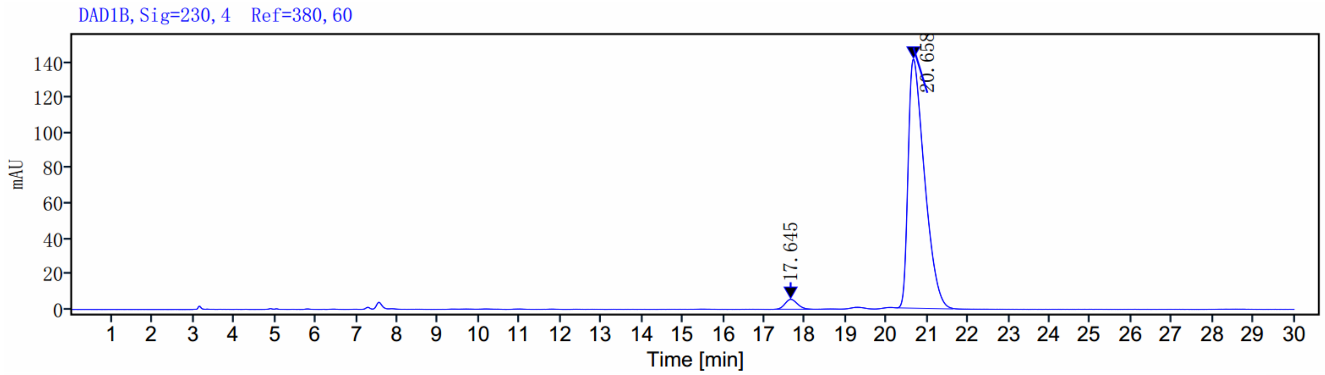

Signal: DAD1B, Sig=230, 4 Ref=380, 60

RetTime [min]	Type	Width [min]	Area [mAU*s]	Height [mAU]	Area %
17.645	VV	0.80318	118.02259	5.64603	2.9077
20.658	BV	1.38593	3940.89969	141.26463	97.0923
		Totals	4058.92229		

Supplementary Figure 131. HPLC data of **35**.

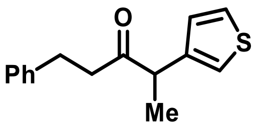

Fig. 2. (36)

(*S*)-**L**: 86% ee; (*R*)-**L**: 86% ee.

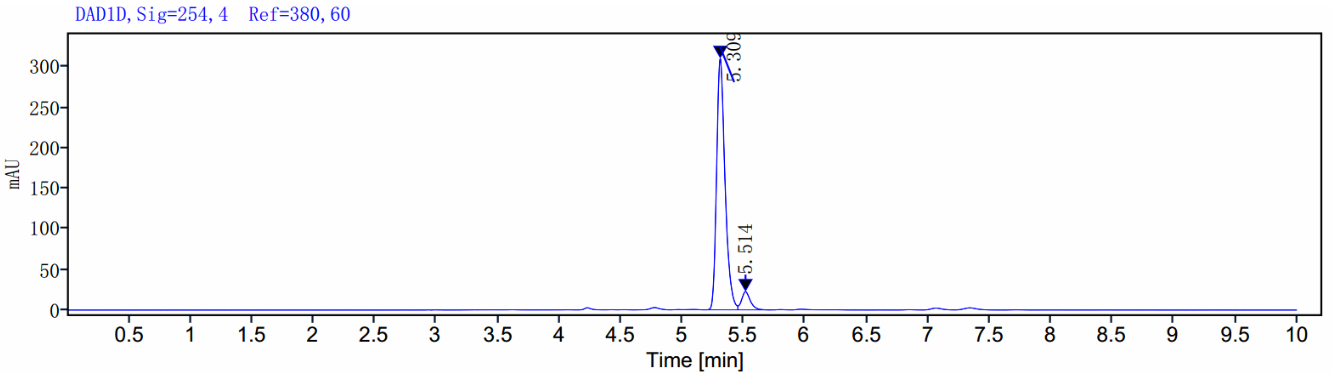

Signal: DAD1D, Sig=254, 4 Ref=380, 60

RetTime [min]	Type	Width [min]	Area [mAU*s]	Height [mAU]	Area %
5.309	BV	0.25697	1486.53032	310.91411	93.0102
5.514	VB	0.26589	111.71333	22.44903	6.9898
		Totals	1598.24365		

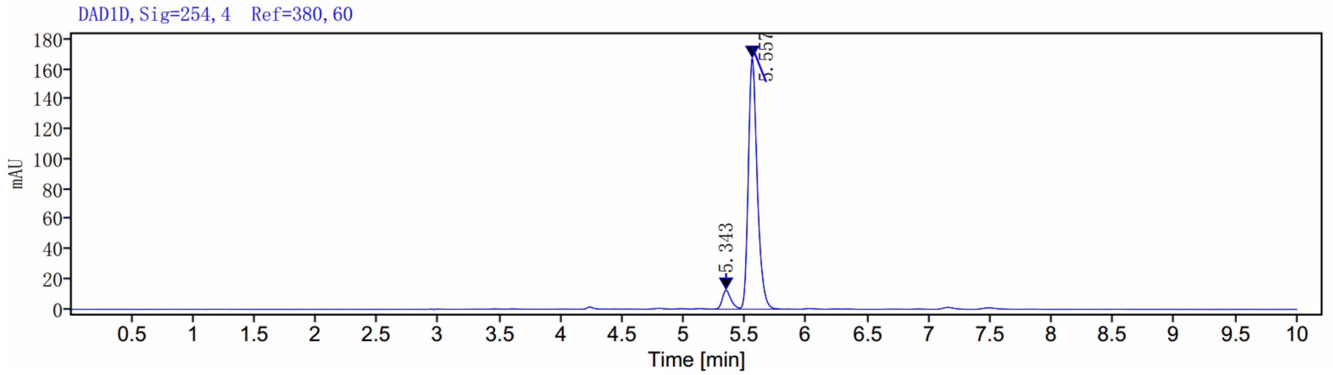

Signal: DAD1D, Sig=254, 4 Ref=380, 60

RetTime [min]	Type	Width [min]	Area [mAU*s]	Height [mAU]	Area %
5.343	BV	0.21181	61.76985	12.60775	6.7804
5.557	VB	0.33619	849.23300	167.52527	93.2196
		Totals	911.00286		

Supplementary Figure 132. HPLC data of **36**.

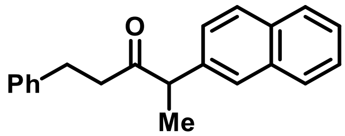

Fig. 2. (37)

(*S*)-**L**: 94% ee; (*R*)-**L**: 94% ee.

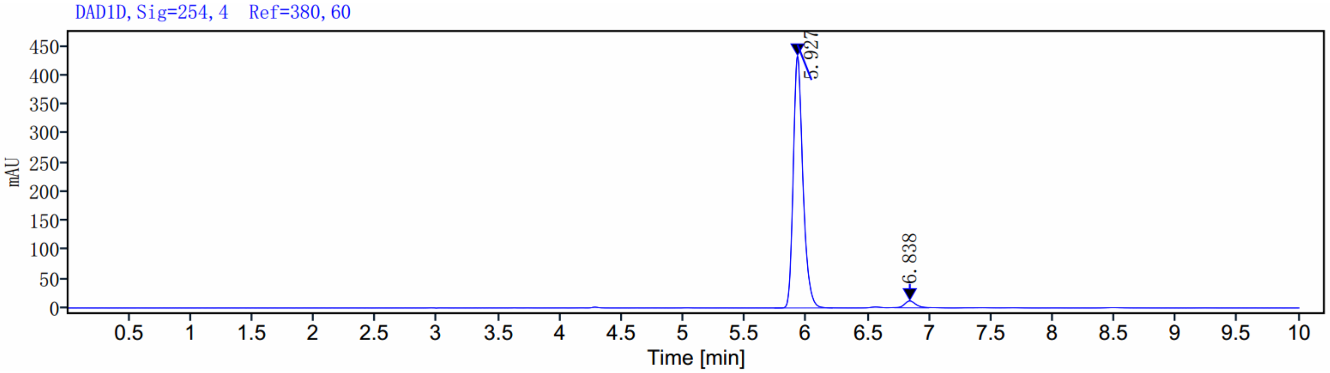

Signal: DAD1D, Sig=254, 4 Ref=380, 60

RetTime [min]	Type	Width [min]	Area [mAU*s]	Height [mAU]	Area %
5.927	BV	0.47216	2363.26570	432.87613	96.9319
6.838	VV	0.28208	74.80164	11.75199	3.0681
		Totals	2438.06734		

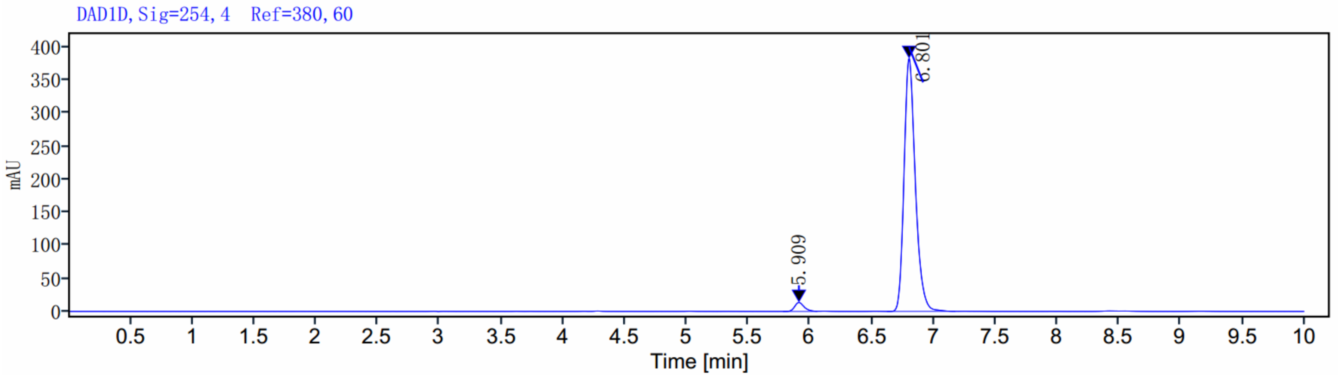

Signal: DAD1D, Sig=254, 4 Ref=380, 60

RetTime [min]	Type	Width [min]	Area [mAU*s]	Height [mAU]	Area %
5.909	BV	0.27513	72.74320	13.57592	2.8958
6.801	BV	0.55250	2439.25192	383.44836	97.1042
		Totals	2511.99512		

Supplementary Figure 133. HPLC data of **37**.

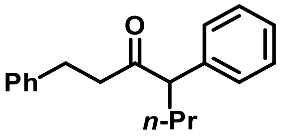

Fig. 2. (38)

(*S*)-**L**: 94% ee; (*R*)-**L**: 94% ee

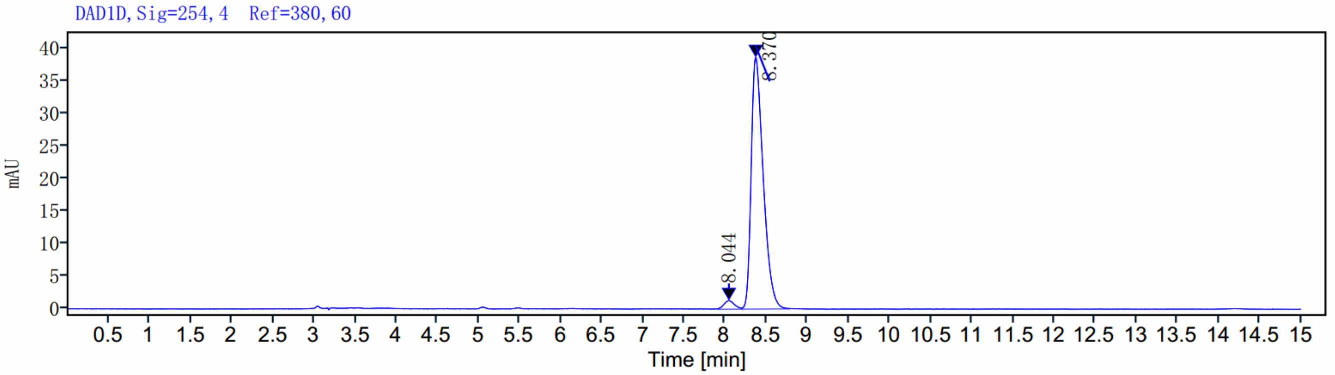

Signal: DAD1D, Sig=254, 4 Ref=380, 60

RetTime [min]	Type	Width [min]	Area [mAU*s]	Height [mAU]	Area %
8.044	VV	0.29634	11.77964	1.32024	2.9050
8.370	VV	0.58374	393.71084	38.66745	97.0950
		Totals	405.49048		

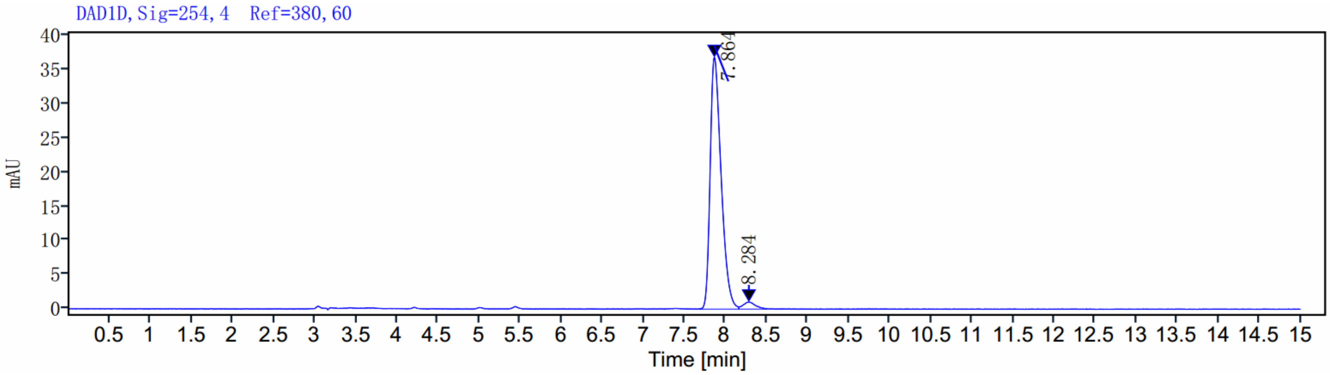

Signal: DAD1D, Sig=254, 4 Ref=380, 60

RetTime [min]	Type	Width [min]	Area [mAU*s]	Height [mAU]	Area %
7.864	VV	0.47855	341.93276	36.78513	96.8934
8.284	VV	0.34373	10.96305	1.02867	3.1066
		Totals	352.89581		

Supplementary Figure 134. HPLC data of **38**.

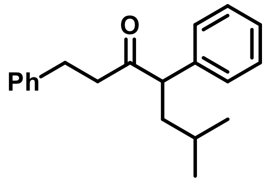

Fig. 2. (39)

(*S*)-**L**: 93% ee; (*R*)-**L**: 93% ee.

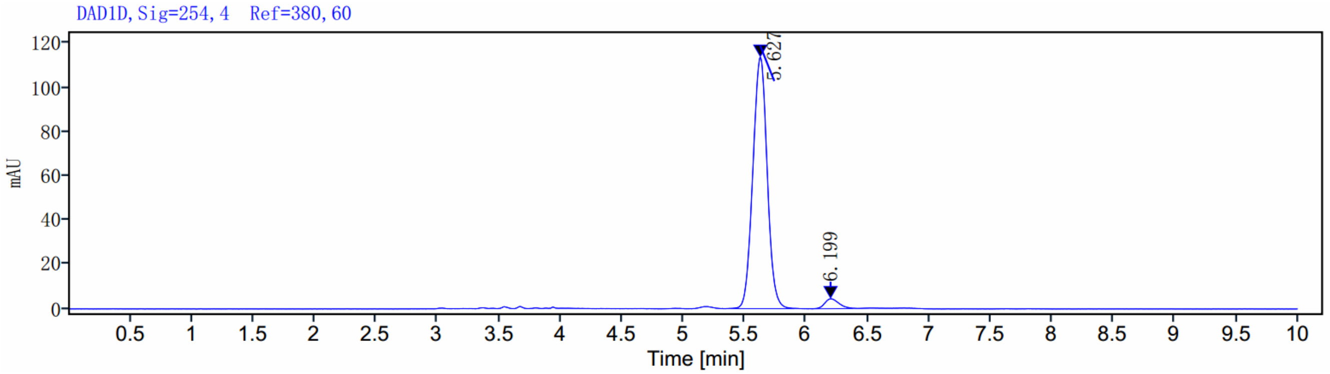

Signal: DAD1D, Sig=254, 4 Ref=380, 60

RetTime [min]	Type	Width [min]	Area [mAU*s]	Height [mAU]	Area %
5.627	VV	0.51360	925.25981	113.59151	96.2884
6.199	BV	0.33185	35.66534	4.45104	3.7116
		Totals	960.92515		

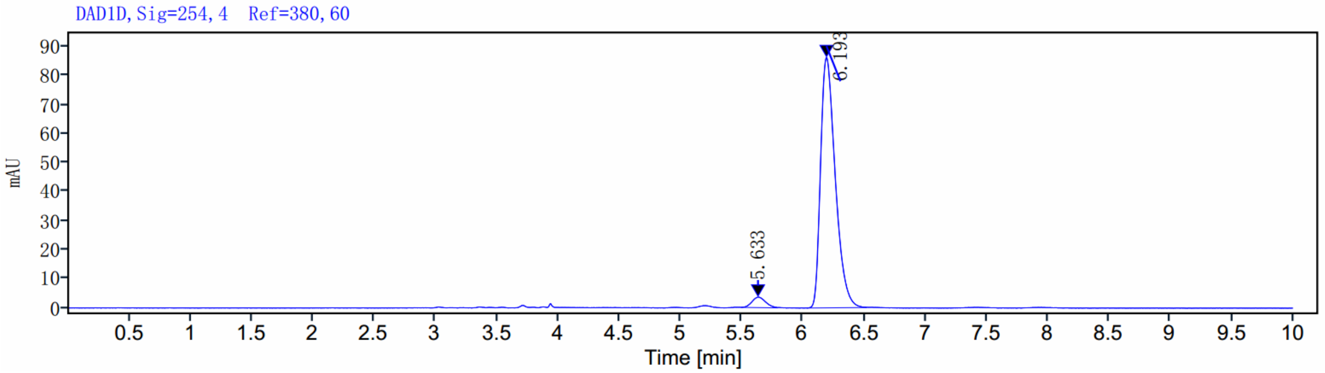

Signal: DAD1D, Sig=254, 4 Ref=380, 60

RetTime [min]	Type	Width [min]	Area [mAU*s]	Height [mAU]	Area %
5.633	BV	0.29789	27.89180	3.62948	3.7369
6.193	VV	0.48597	718.49179	86.19423	96.2631
		Totals	746.38360		

Supplementary Figure 135. HPLC data of **39**.

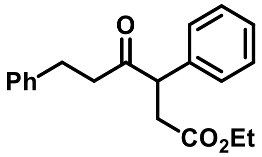

Fig. 2. (40)

(*S*)-**L**: 95% ee; (*R*)-**L**: 94% ee.

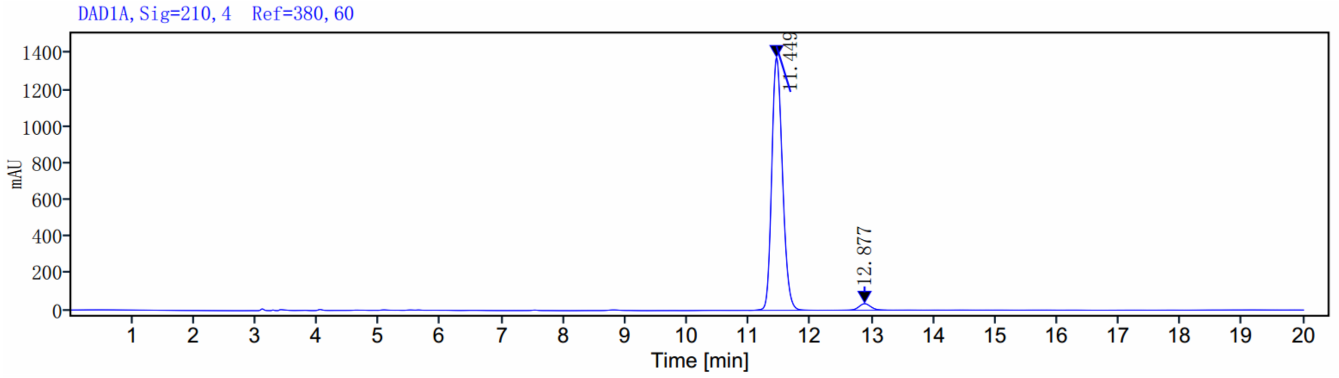

Signal: DAD1A, Sig=210, 4 Ref=380, 60

RetTime [min]	Type	Width [min]	Area [mAU*s]	Height [mAU]	Area %
11.449	VV	1.01074	16675.92004	1377.51217	97.2639
12.877	VV	0.66956	469.10426	35.40592	2.7361
		Totals	17145.02430		

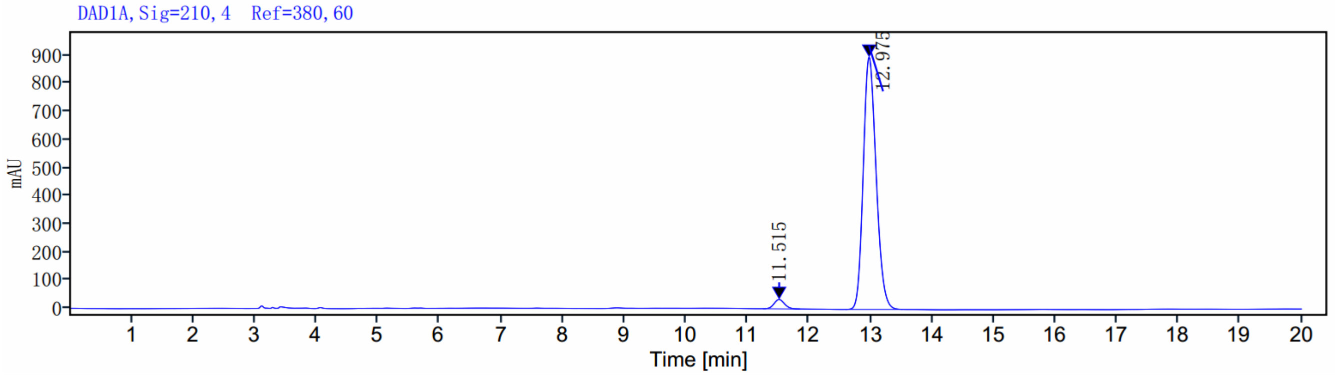

Signal: DAD1A, Sig=210, 4 Ref=380, 60

RetTime [min]	Type	Width [min]	Area [mAU*s]	Height [mAU]	Area %
11.515	VV	0.60182	398.97732	33.28112	2.9670
12.975	VV	0.92441	13048.25556	897.70903	97.0330
		Totals	13447.23288		

Supplementary Figure 136. HPLC data of **40**.

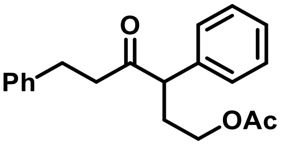

Fig. 2. (41)

(*S*)-**L**: 95% ee; (*R*)-**L**: 95% ee.

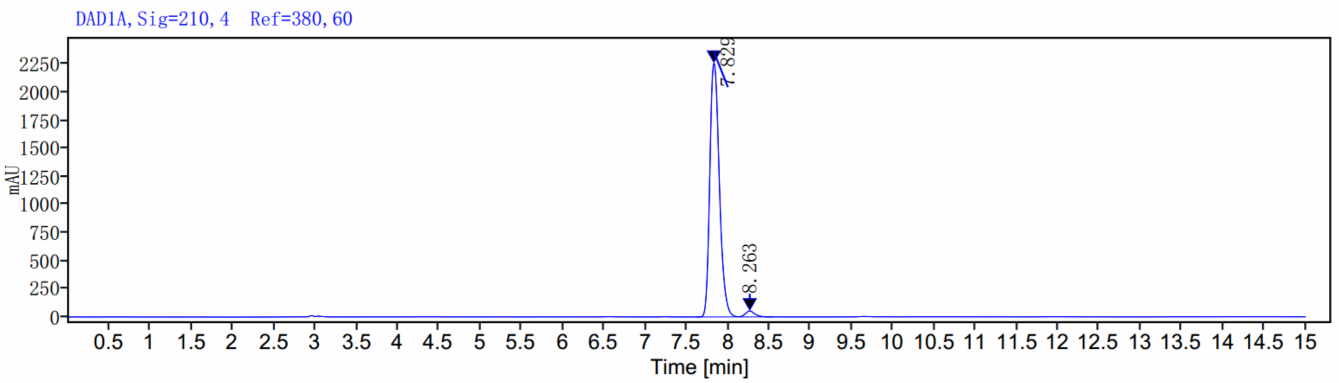

Signal: DAD1A, Sig=210, 4 Ref=380, 60

RetTime [min]	Type	Width [min]	Area [mAU*s]	Height [mAU]	Area %
7.829	BV	0.50526	18157.52437	2258.57081	97.7326
8.263	VV	0.42170	421.24822	52.91244	2.2674
		Totals	18578.77260		

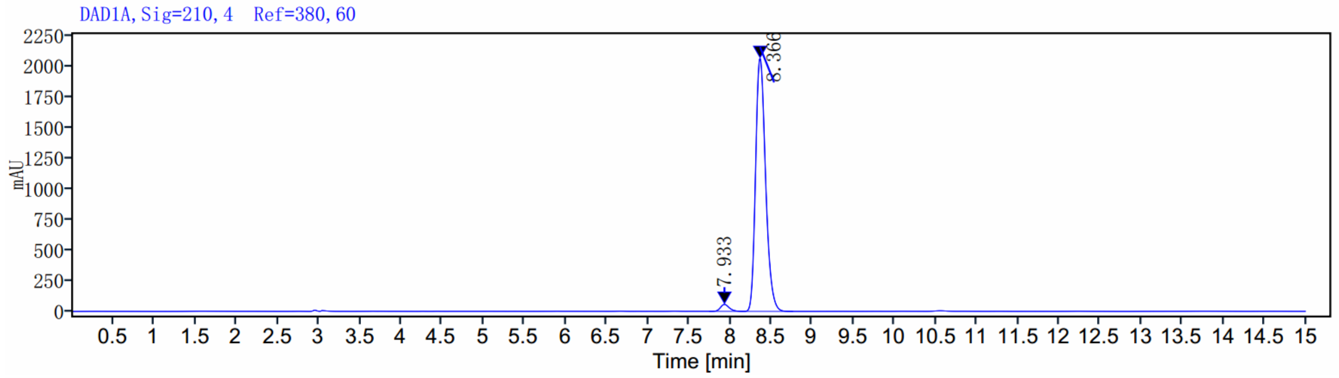

Signal: DAD1A, Sig=210, 4 Ref=380, 60

RetTime [min]	Type	Width [min]	Area [mAU*s]	Height [mAU]	Area %
7.933	VB	0.41179	420.52531	56.75057	2.3437
8.366	BV	0.60668	17522.64847	2068.78715	97.6563
		Totals	17943.17378		

Supplementary Figure 137. HPLC data of **41**.

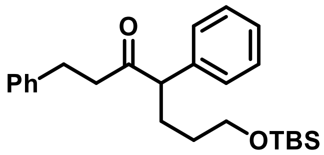

Fig. 2. (42)

(*S*)-**L**: 95% ee; (*R*)-**L**: 93% ee.

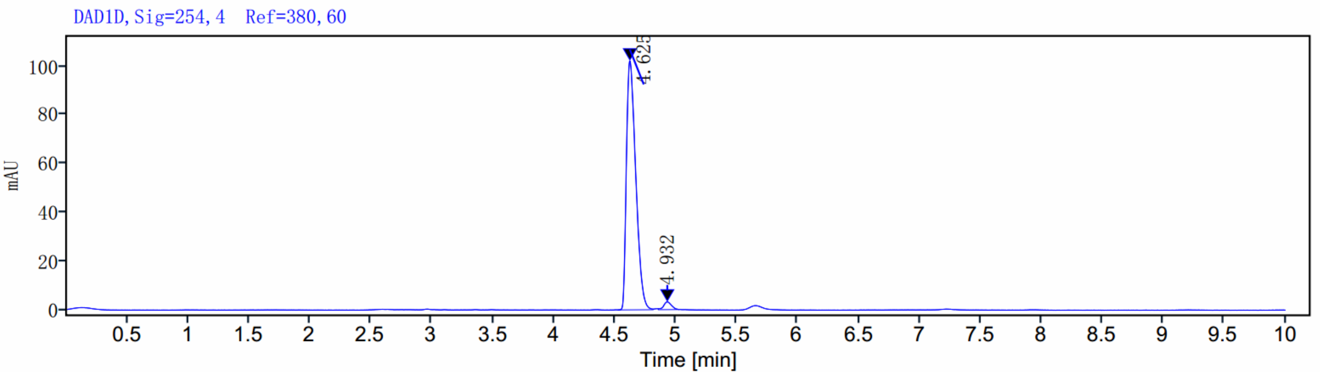

Signal: DAD1D, Sig=254, 4 Ref=380, 60

RetTime [min]	Type	Width [min]	Area [mAU*s]	Height [mAU]	Area %
4.625	BV	0.29190	543.77394	102.09363	97.4517
4.932	VB	0.17194	14.21931	3.18172	2.5483
		Totals	557.99326		

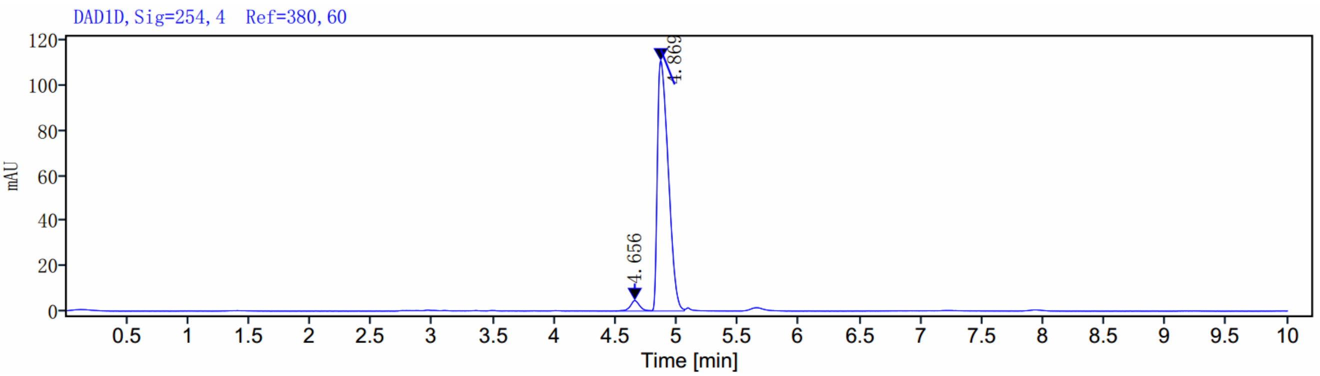

Signal: DAD1D, Sig=254, 4 Ref=380, 60

RetTime [min]	Type	Width [min]	Area [mAU*s]	Height [mAU]	Area %
4.656	VV	0.29225	25.05273	4.68104	3.5149
4.869	VV	0.27125	687.71240	111.23039	96.4851
		Totals	712.76512		

Supplementary Figure 138. HPLC data of **42**.

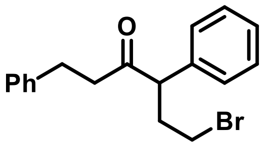

Fig. 2. (43)

(*S*)-**L**: 80% ee; (*R*)-**L**: 83% ee.

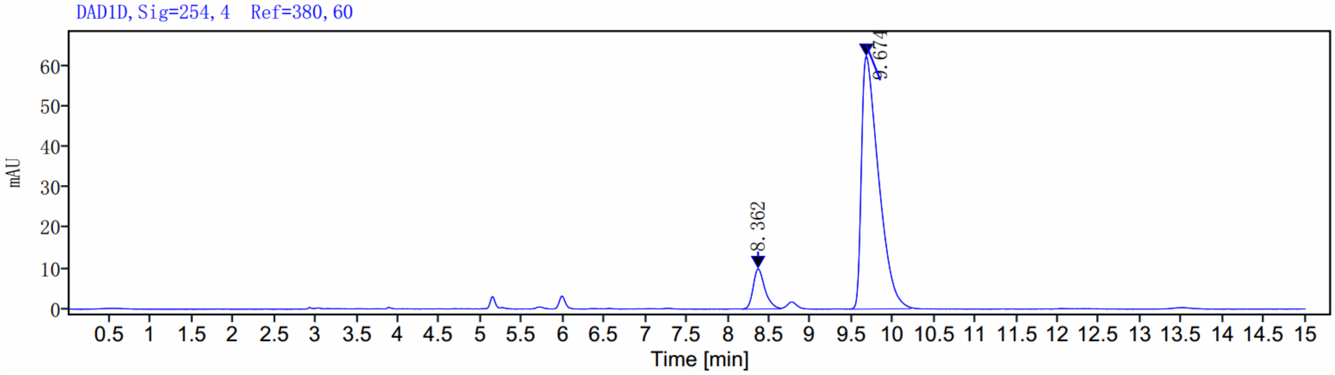

Signal: DAD1D, Sig=254, 4 Ref=380, 60

RetTime [min]	Type	Width [min]	Area [mAU*s]	Height [mAU]	Area %
8.362	BV	0.46889	97.97584	9.94016	9.8197
9.674	BV	0.74064	899.76745	62.28483	90.1803
		Totals	997.74329		

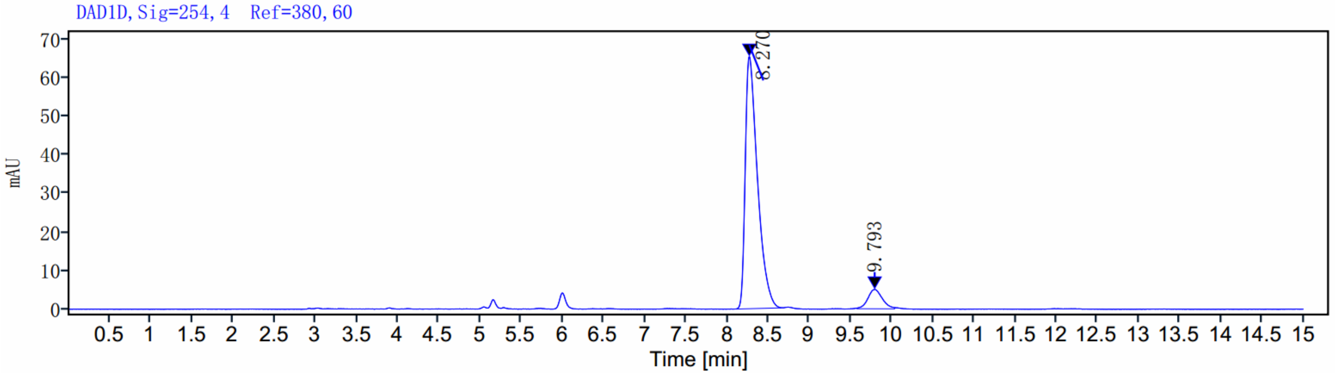

Signal: DAD1D, Sig=254, 4 Ref=380, 60

RetTime [min]	Type	Width [min]	Area [mAU*s]	Height [mAU]	Area %
8.270	VV	0.54851	678.17653	65.68253	91.7324
9.793	VV	0.46094	61.12198	5.07964	8.2676
		Totals	739.29852		

Supplementary Figure 139. HPLC data of **43**.

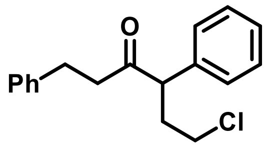

Fig. 2. (44)

(*S*)-**L**: 94% ee; (*R*)-**L**: 95% ee.

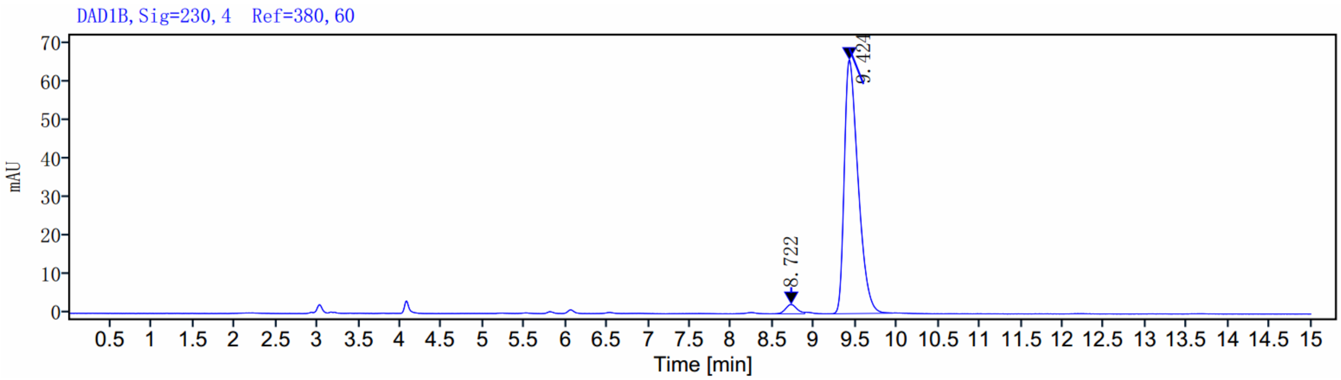

Signal: DAD1B, Sig=230, 4 Ref=380, 60

RetTime [min]	Type	Width [min]	Area [mAU*s]	Height [mAU]	Area %
8.722	BV	0.36280	23.04927	2.41044	2.9399
9.424	BV	0.72826	760.96089	65.59398	97.0601
		Totals	784.01016		

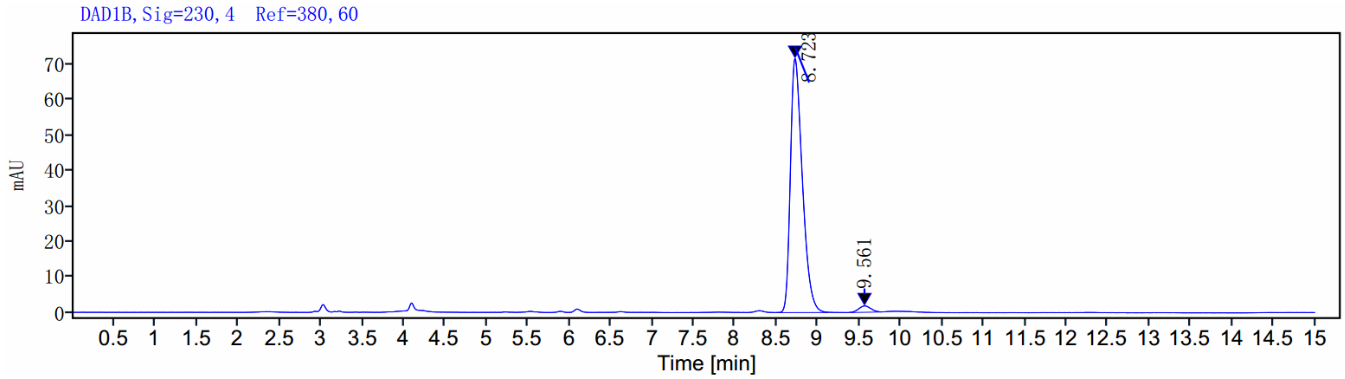

Signal: DAD1B, Sig=230, 4 Ref=380, 60

RetTime [min]	Type	Width [min]	Area [mAU*s]	Height [mAU]	Area %
8.723	VV	0.61852	721.60167	71.91878	97.5121
9.561	VV	0.35839	18.41097	1.83545	2.4879
		Totals	740.01265		

Supplementary Figure 140. HPLC data of **44**.

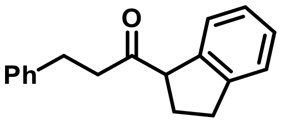

Fig. 2. (45)

(*S*)-**L**: 82% ee; (*R*)-**L**: 81% ee.

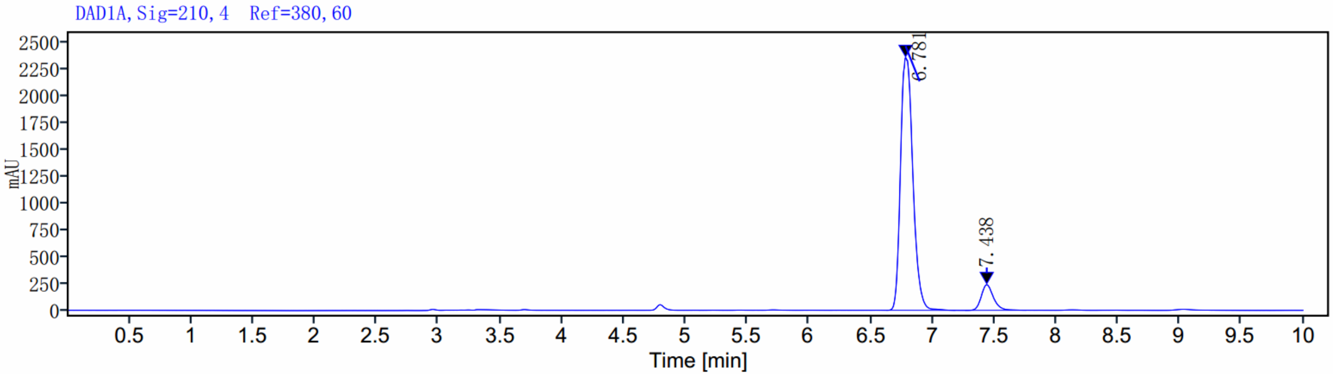

Signal: DAD1A, Sig=210, 4 Ref=380, 60

RetTime [min]	Type	Width [min]	Area [mAU*s]	Height [mAU]	Area %
6.781	VV	0.55738	16387.12471	2352.85468	90.9571
7.438	VV	0.49521	1629.18967	238.26398	9.0429
		Totals	18016.31438		

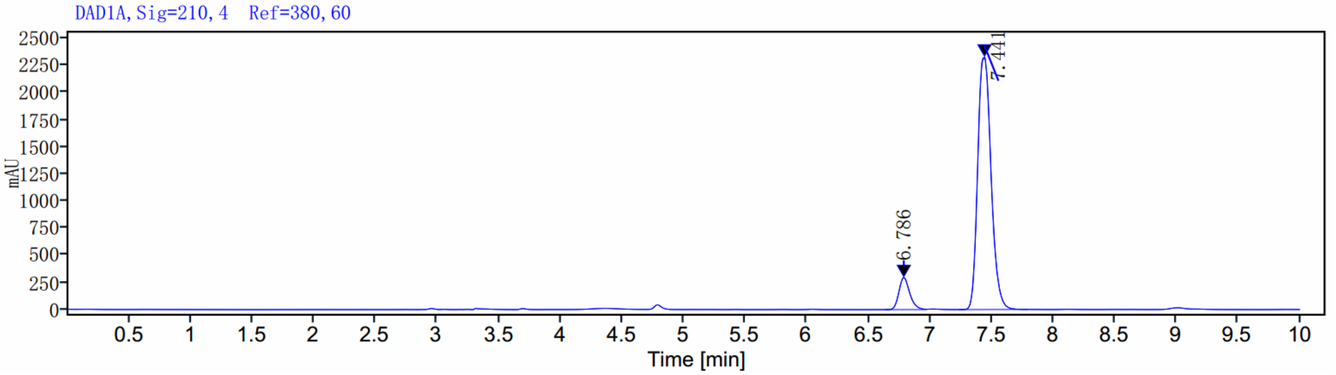

Signal: DAD1A, Sig=210, 4 Ref=380, 60

RetTime [min]	Type	Width [min]	Area [mAU*s]	Height [mAU]	Area %
6.786	BV	0.34038	1812.67886	295.73008	9.3455
7.441	BV	0.55335	17583.62136	2329.21734	90.6545
		Totals	19396.30022		

Supplementary Figure 141. HPLC data of **45**.

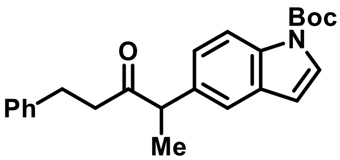

Fig. 2. (46)

(*S*)-**L**: 95% ee; (*R*)-**L**: 95% ee.

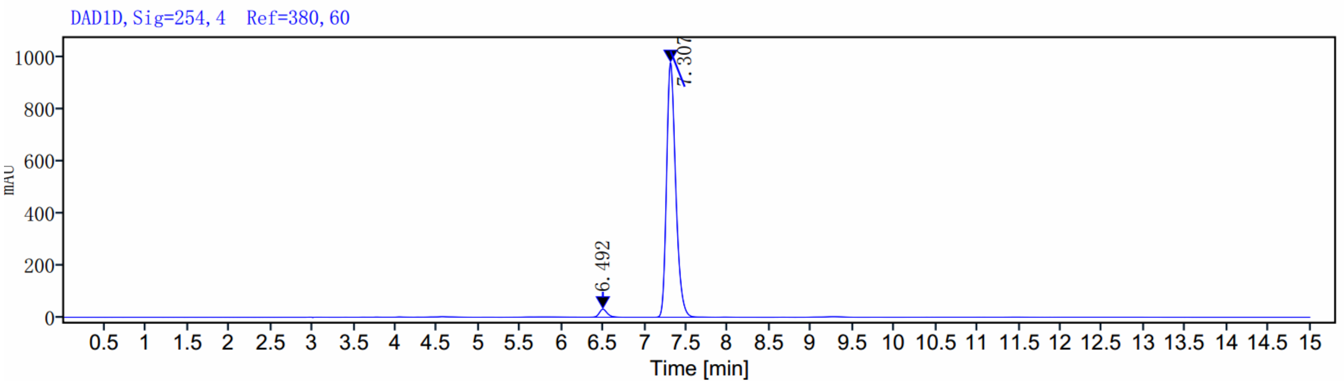

Signal: DAD1D, Sig=254, 4 Ref=380, 60

RetTime [min]	Type	Width [min]	Area [mAU*s]	Height [mAU]	Area %
6.492	VV	0.41766	203.97695	30.83045	2.6617
7.307	VV	0.58288	7459.41422	976.56212	97.3383
		Totals	7663.39117		

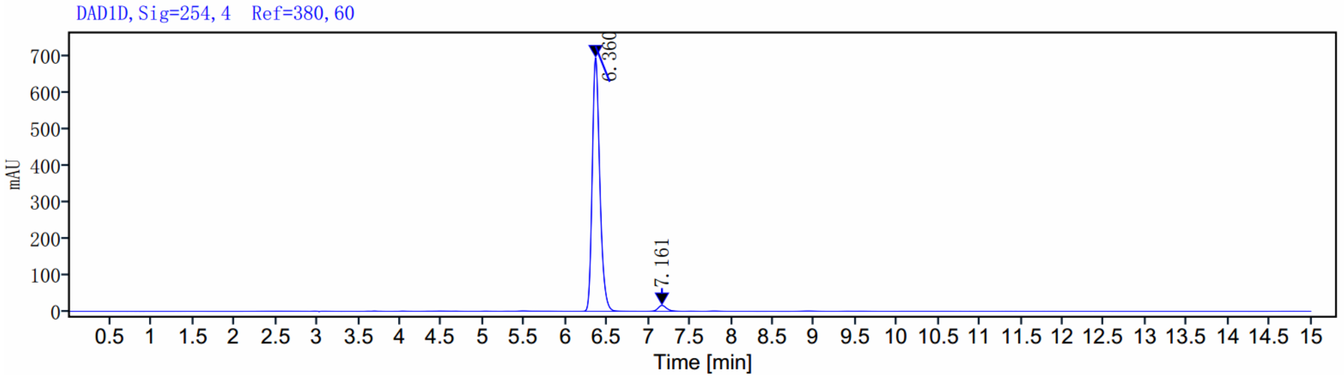

Signal: DAD1D, Sig=254, 4 Ref=380, 60

RetTime [min]	Type	Width [min]	Area [mAU*s]	Height [mAU]	Area %
6.360	BV	0.54631	4422.59907	693.71151	97.3947
7.161	VV	0.46030	118.30590	16.18386	2.6053
		Totals	4540.90498		

Supplementary Figure 142. HPLC data of **46**.

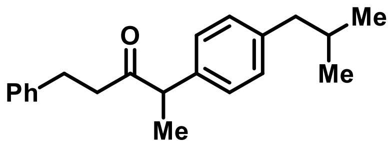

Fig. 3. (47)

(*S*)-**L**: 94% ee; (*R*)-**L**: 95% ee.

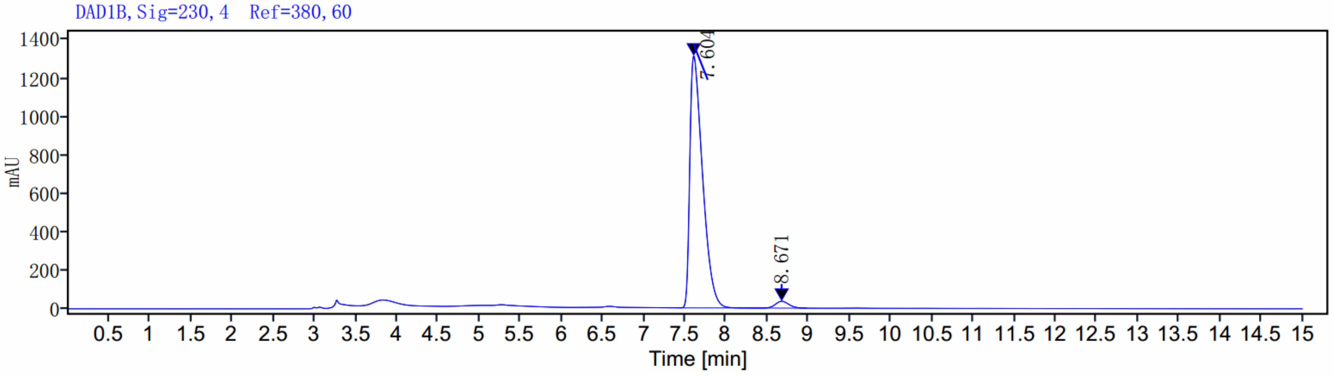

Signal: DAD1B, Sig=230, 4 Ref=380, 60

RetTime [min]	Type	Width [min]	Area [mAU*s]	Height [mAU]	Area %
7.604	BV	0.74109	14090.18864	1313.15360	97.2279
8.671	MM m	0.80376	401.73588	35.71061	2.7721
		Totals	14491.92452		

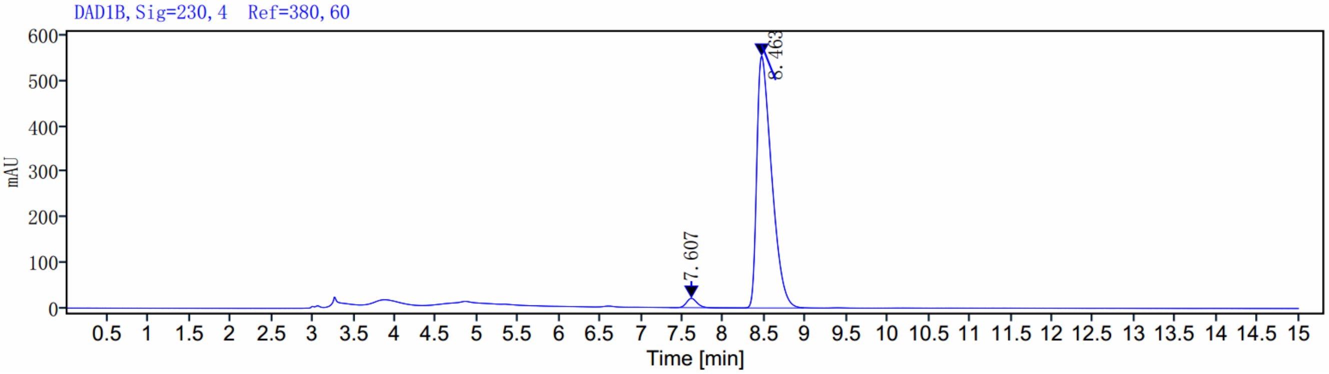

Signal: DAD1B, Sig=230, 4 Ref=380, 60

RetTime [min]	Type	Width [min]	Area [mAU*s]	Height [mAU]	Area %
7.607	MM m	0.91132	183.44043	20.58171	2.5601
8.463	VV	0.72008	6981.96375	555.04071	97.4399
		Totals	7165.40418		

Supplementary Figure 143. HPLC data of **47**.

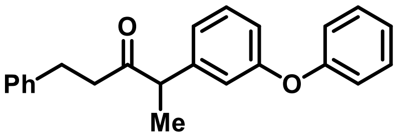

Fig. 3. (48)

(*S*)-**L**: 93% ee; (*R*)-**L**: 94% ee.

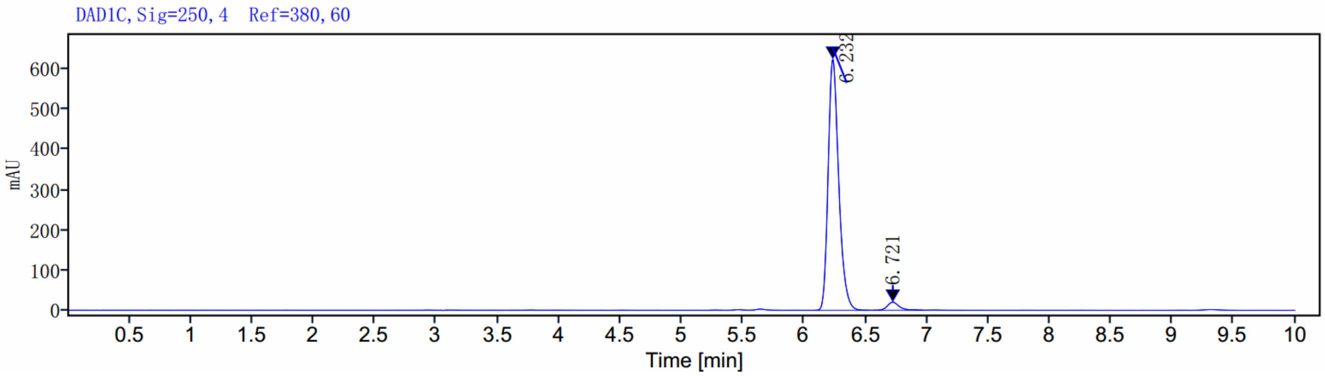

Signal: DAD1C, Sig=250, 4 Ref=380, 60

RetTime [min]	Type	Width [min]	Area [mAU*s]	Height [mAU]	Area %
6.232	VV	0.45442	3758.62630	626.77863	96.4898
6.721	VV	0.40778	136.73549	20.23472	3.5102
		Totals	3895.36179		

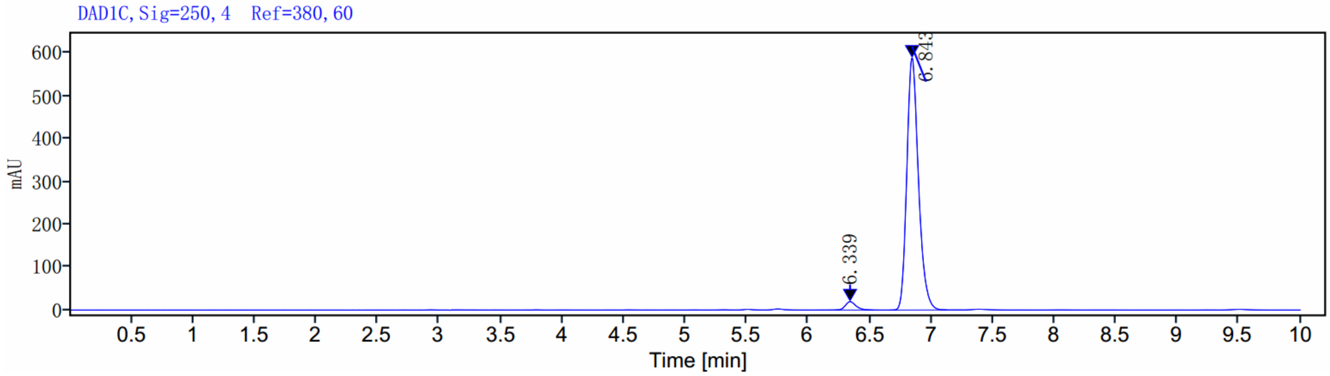

Signal: DAD1C, Sig=250, 4 Ref=380, 60

RetTime [min]	Type	Width [min]	Area [mAU*s]	Height [mAU]	Area %
6.339	VV	0.30787	117.50283	19.51497	2.9365
6.843	VV	0.46618	3883.92255	589.49164	97.0635
		Totals	4001.42538		

Supplementary Figure 144. HPLC data of **48**.

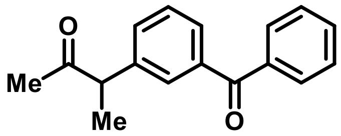

Fig. 3. (49)

(*S*)-**L**: 94% ee; (*R*)-**L**: 93% ee.

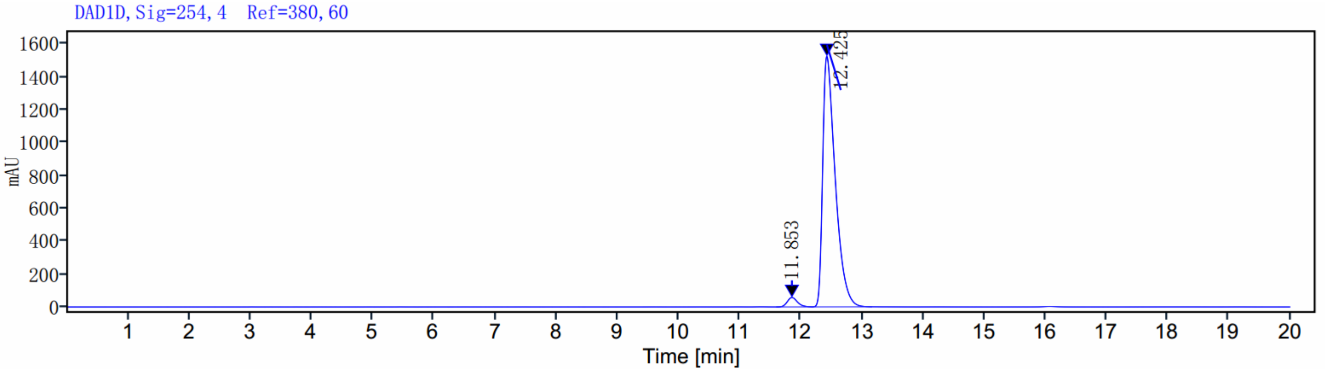

Signal: DAD1D, Sig=254, 4 Ref=380, 60

RetTime [min]	Type	Width [min]	Area [mAU*s]	Height [mAU]	Area %
11.853	VV	0.58903	669.09361	57.87287	2.9858
12.425	VV	0.97692	21740.06229	1526.86494	97.0142
		Totals	22409.15590		

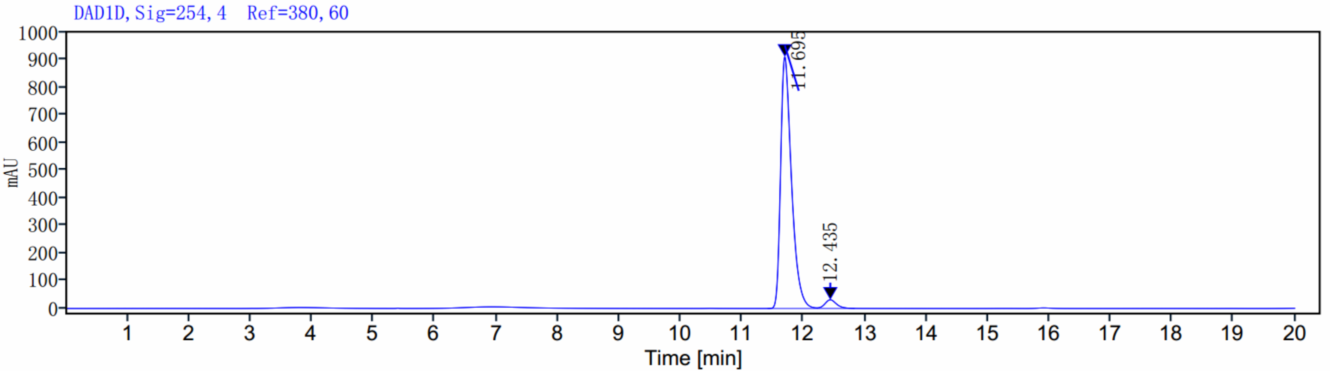

Signal: DAD1D, Sig=254, 4 Ref=380, 60

RetTime [min]	Type	Width [min]	Area [mAU*s]	Height [mAU]	Area %
11.695	BV	0.80603	11204.20468	910.22465	96.4428
12.435	VV	0.61124	413.26094	31.38646	3.5572
		Totals	11617.46562		

Supplementary Figure 145. HPLC data of **49**.

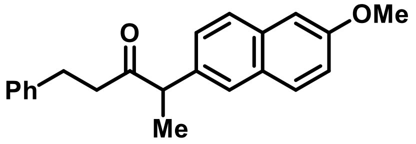

Fig. 3. (50)

(*S*)-**L**: 93% ee; (*R*)-**L**: 92% ee.

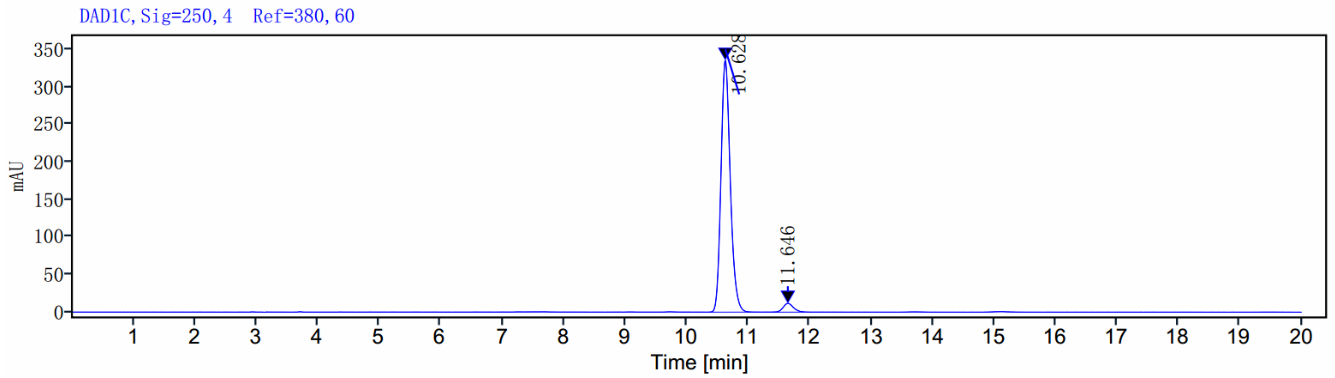

Signal: DAD1C, Sig=250, 4 Ref=380, 60

RetTime [min]	Type	Width [min]	Area [mAU*s]	Height [mAU]	Area %
10.628	VV	0.66310	3531.07668	336.07268	96.2836
11.646	VV	0.52391	136.29267	11.69533	3.7164
		Totals	3667.36935		

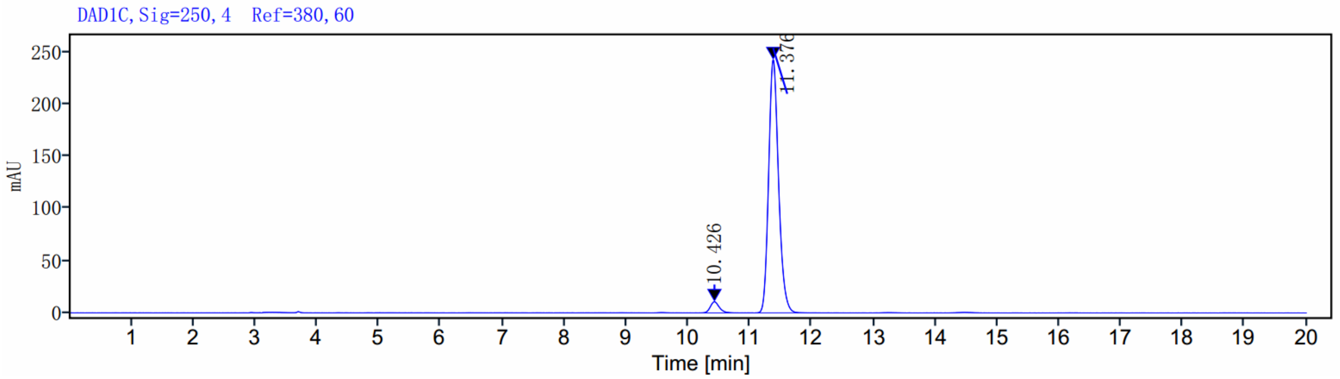

Signal: DAD1C, Sig=250, 4 Ref=380, 60

RetTime [min]	Type	Width [min]	Area [mAU*s]	Height [mAU]	Area %
10.426	VV	0.44461	106.53975	10.65734	3.7984
11.376	VV	0.66901	2698.30099	242.28445	96.2016
		Totals	2804.84075		

Supplementary Figure 146. HPLC data of **50**.

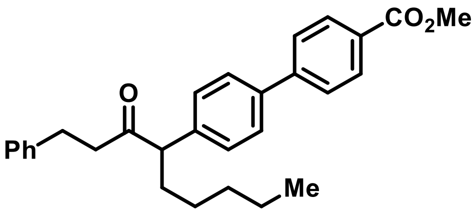

Fig. 3. (51)

(*S*)-**L**: 92% ee; (*R*)-**L**: 92% ee.

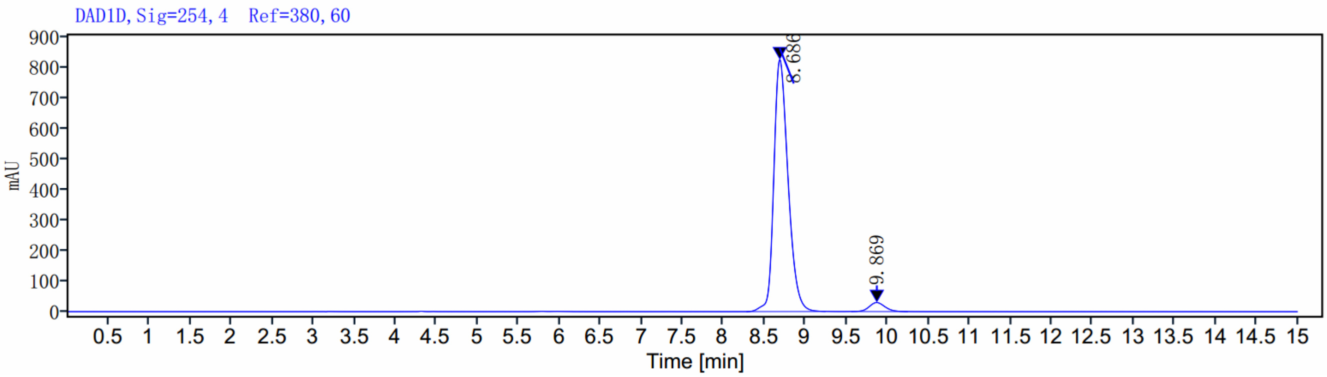

Signal: DAD1D, Sig=254, 4 Ref=380, 60

RetTime [min]	Type	Width [min]	Area [mAU*s]	Height [mAU]	Area %
8.686	VV	0.96416	9977.44128	823.97610	96.1871
9.869	VV	0.68681	395.51054	29.51613	3.8129
		Totals	10372.95182		

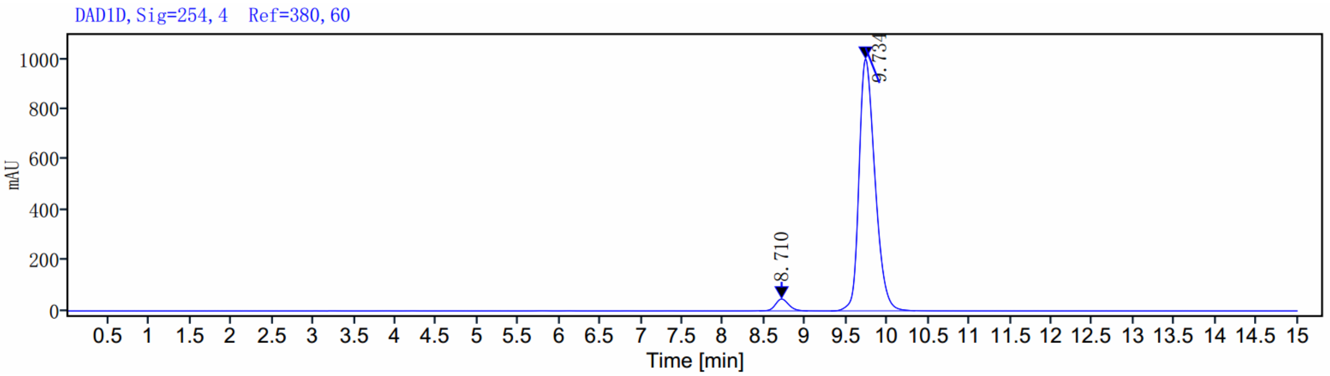

Signal: DAD1D, Sig=254, 4 Ref=380, 60

RetTime [min]	Type	Width [min]	Area [mAU*s]	Height [mAU]	Area %
8.710	VV	0.59022	534.26686	47.18044	3.8072
9.734	BV	1.02801	13498.85705	998.40188	96.1928
		Totals	14033.12390		

Supplementary Figure 147. HPLC data of **51**.

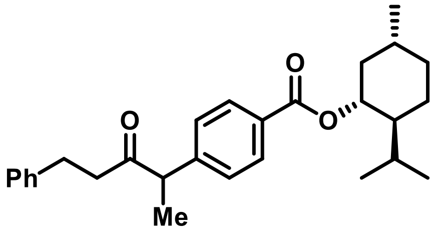

Fig. 3. (52 & 53)

(*S*)-**L**: 4:96 dr; (*R*)-**L**: 96:4 dr.

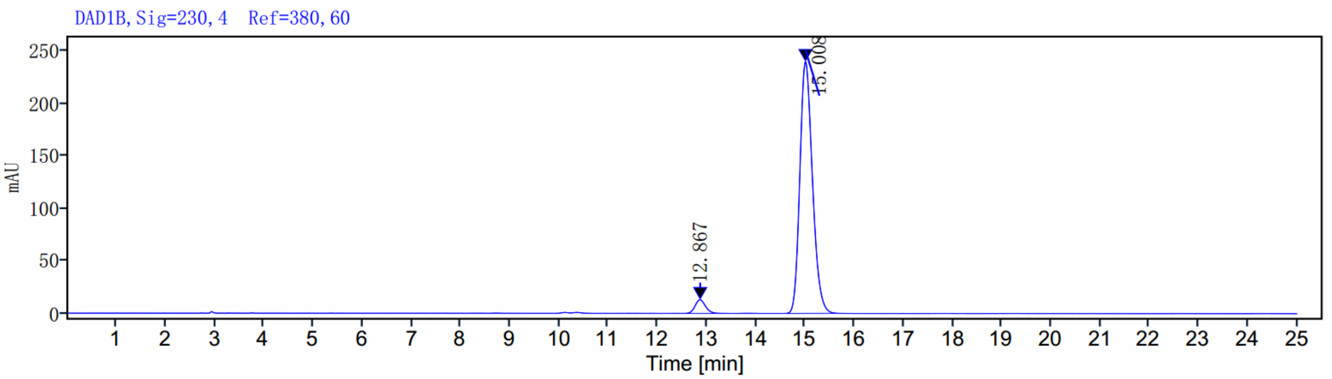

Signal: DAD1B, Sig=230, 4 Ref=380, 60

RetTime [min]	Type	Width [min]	Area [mAU*s]	Height [mAU]	Area %
12.867	VV	0.55671	189.25675	13.00941	4.2451
15.008	VV	1.00042	4268.93762	240.16207	95.7549
		Totals	4458.19437		

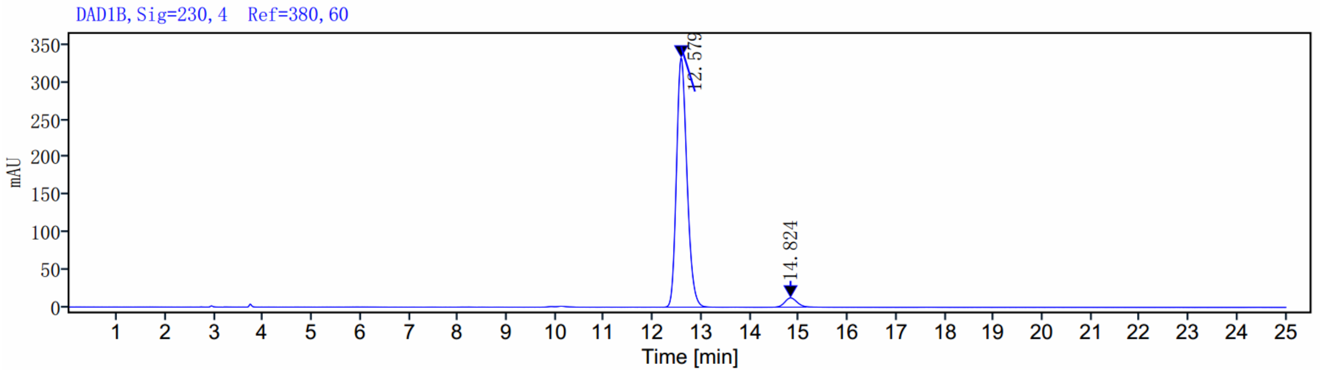

Signal: DAD1B, Sig=230, 4 Ref=380, 60

RetTime [min]	Type	Width [min]	Area [mAU*s]	Height [mAU]	Area %
12.579	BV	0.90872	4981.57814	332.87668	95.9116
14.824	VV	0.64949	212.34673	12.33053	4.0884
		Totals	5193.92487		

Supplementary Figure 148. HPLC data of **52 & 53**.

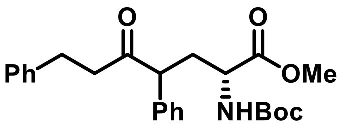

Fig. 3. (54 & 55)

(*S*)-**L**: 3:97 dr; (*R*)-**L**: 99:1 dr.

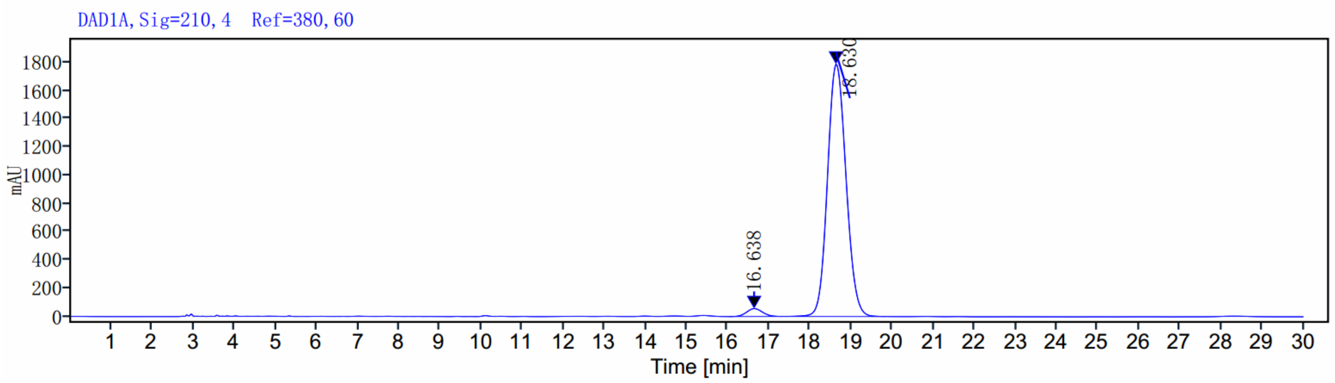

Signal: DAD1A, Sig=210, 4 Ref=380, 60

RetTime [min]	Type	Width [min]	Area [mAU*s]	Height [mAU]	Area %
16.638	BV	1.29447	1498.31576	55.71714	2.6032
18.630	VV	2.40116	56059.38137	1788.16433	97.3968
		Totals	57557.69713		

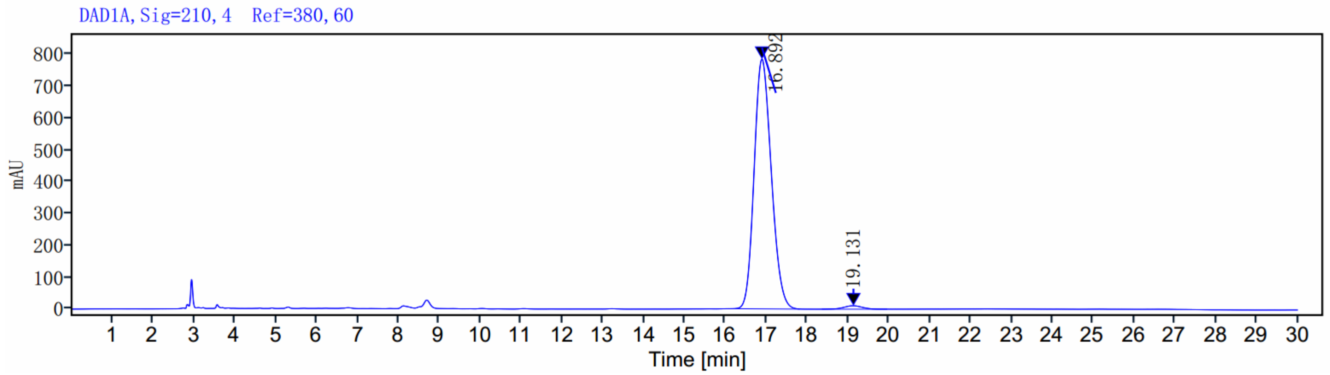

Signal: DAD1A, Sig=210, 4 Ref=380, 60

RetTime [min]	Type	Width [min]	Area [mAU*s]	Height [mAU]	Area %
16.892	VV	1.57060	22938.49999	785.40259	98.5626
19.131	MM m	1.59853	334.53615	10.77086	1.4374
		Totals	23273.03613		

Supplementary Figure 149. HPLC data of **54 & 55**.

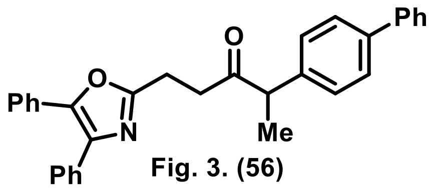

Fig. 3. (56)

(S)-**L**: 87% ee; (R)-**L**: 88% ee.

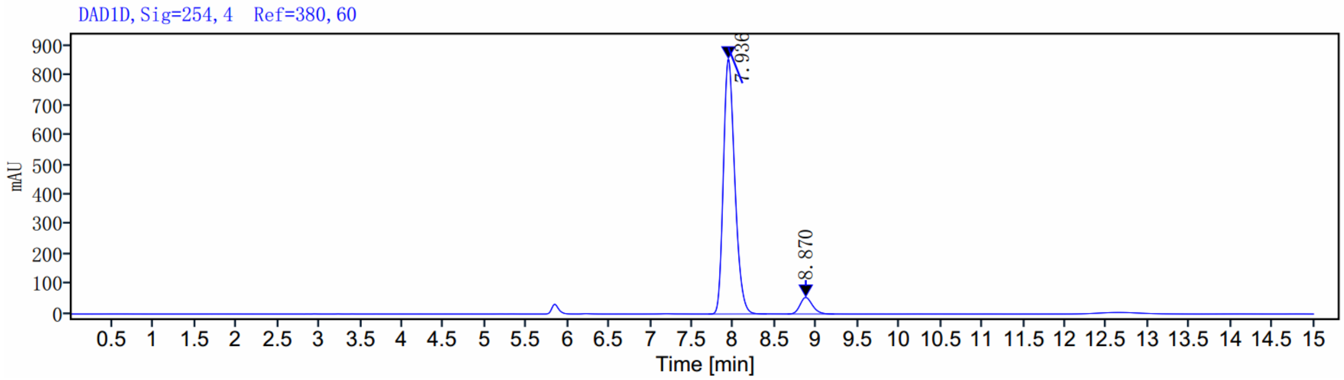

Signal: DAD1D, Sig=254, 4 Ref=380, 60

RetTime [min]	Type	Width [min]	Area [mAU*s]	Height [mAU]	Area %
7.936	BV	0.70258	8193.34379	854.72072	93.4163
8.870	BV	0.56355	577.44311	55.45450	6.5837
		Totals	8770.78690		

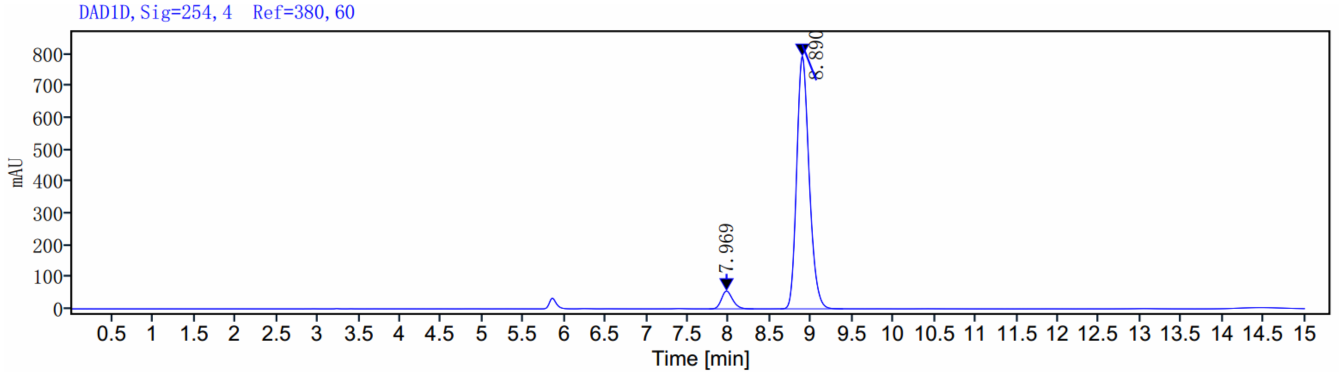

Signal: DAD1D, Sig=254, 4 Ref=380, 60

RetTime [min]	Type	Width [min]	Area [mAU*s]	Height [mAU]	Area %
7.969	BV	0.53833	528.24047	56.03062	5.9224
8.890	VV	0.75963	8391.08335	793.93864	94.0776
		Totals	8919.32382		

Supplementary Figure 150. HPLC data of **56**.

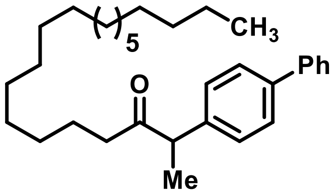

Fig. 3. (57)

(*S*)-**L**: 90% ee; (*R*)-**L**: 90% ee.

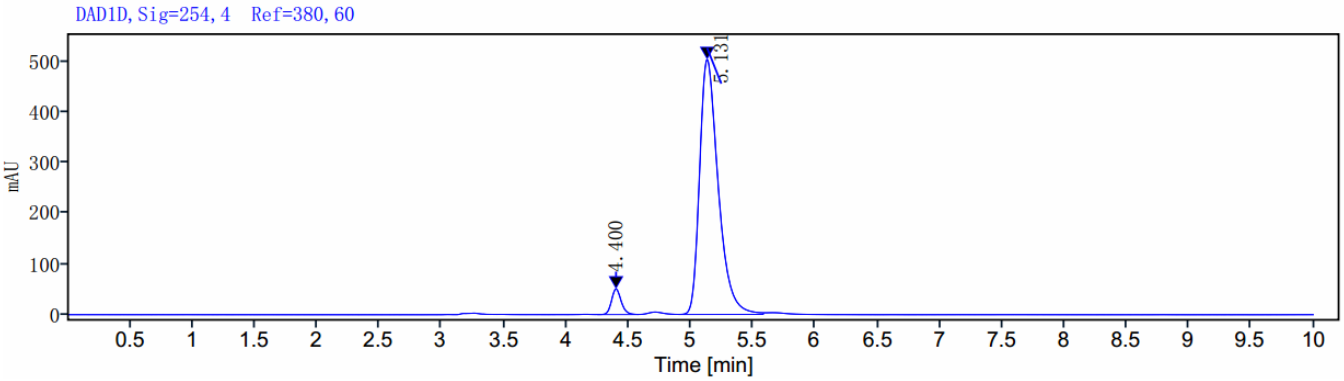

Signal: DAD1D, Sig=254, 4 Ref=380, 60

RetTime [min]	Type	Width [min]	Area [mAU*s]	Height [mAU]	Area %
4.400	VV	0.32836	273.73355	50.51478	4.9554
5.131	VV	0.67939	5250.18077	504.11789	95.0446
		Totals	5523.91433		

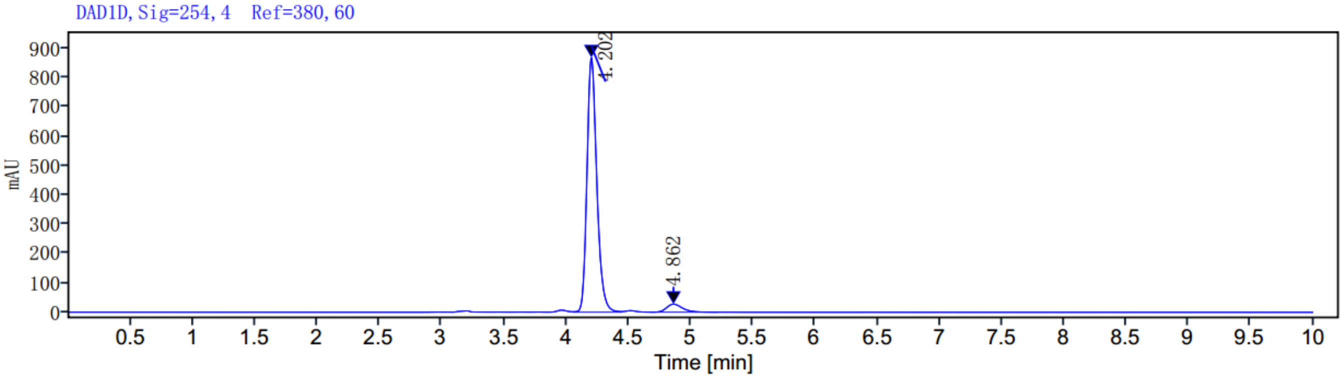

Signal: DAD1D, Sig=254, 4 Ref=380, 60

RetTime [min]	Type	Width [min]	Area [mAU*s]	Height [mAU]	Area %
4.202	VV	0.39197	4778.79044	869.22738	95.1705
4.862	BV	0.53958	242.50323	27.42497	4.8295
		Totals	5021.29368		

Supplementary Figure 151. HPLC data of **57**.

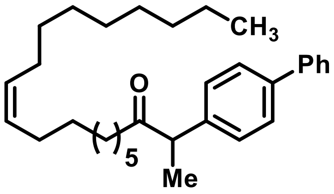

Fig. 3. (58)

(*S*)-**L**: 94% ee; (*R*)-**L**: 94% ee.

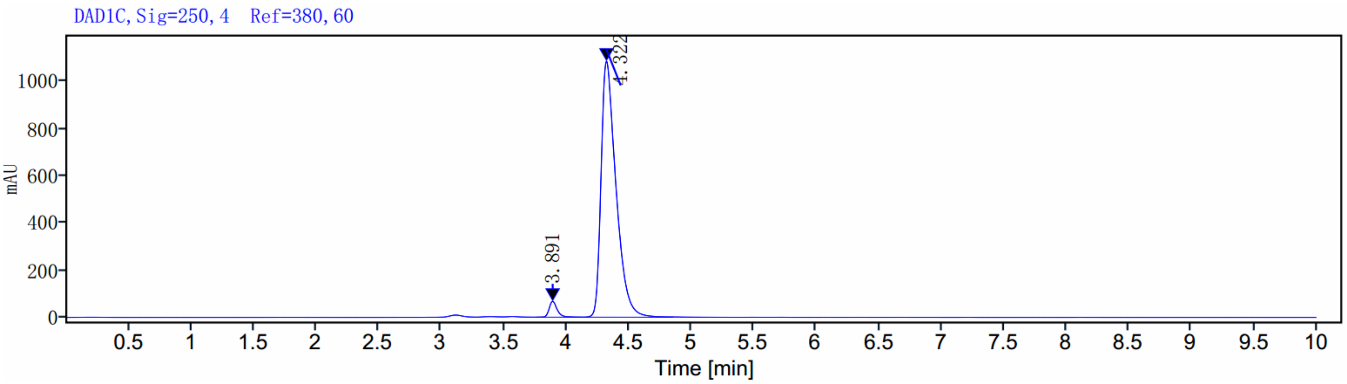

Signal: DAD1C, Sig=250, 4 Ref=380, 60

RetTime [min]	Type	Width [min]	Area [mAU*s]	Height [mAU]	Area %
3.891	BV	0.37527	301.59200	67.30469	3.2424
4.322	VV	0.72264	8999.84119	1085.10844	96.7576
		Totals	9301.43319		

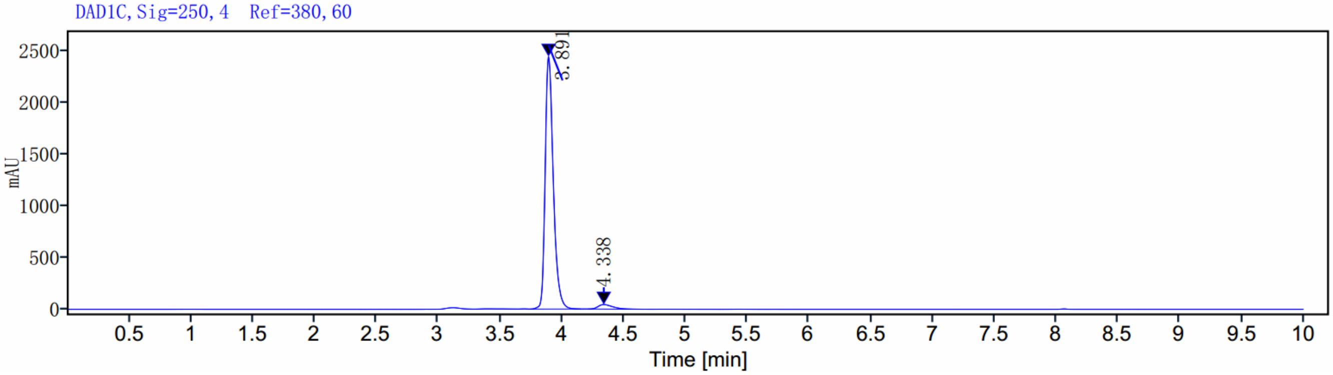

Signal: DAD1C, Sig=250, 4 Ref=380, 60

RetTime [min]	Type	Width [min]	Area [mAU*s]	Height [mAU]	Area %
3.891	BB	0.40917	11208.70681	2451.40031	96.8761
4.338	VV	0.41956	361.44360	44.32724	3.1239
		Totals	11570.15041		

Supplementary Figure 152. HPLC data of **58**.

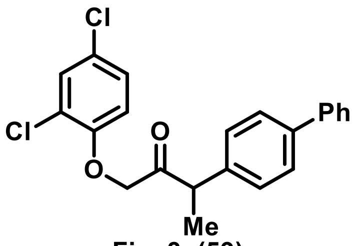

Fig. 3. (59)

(*S*)-**L**: 87% ee; (*R*)-**L**: 86% ee.

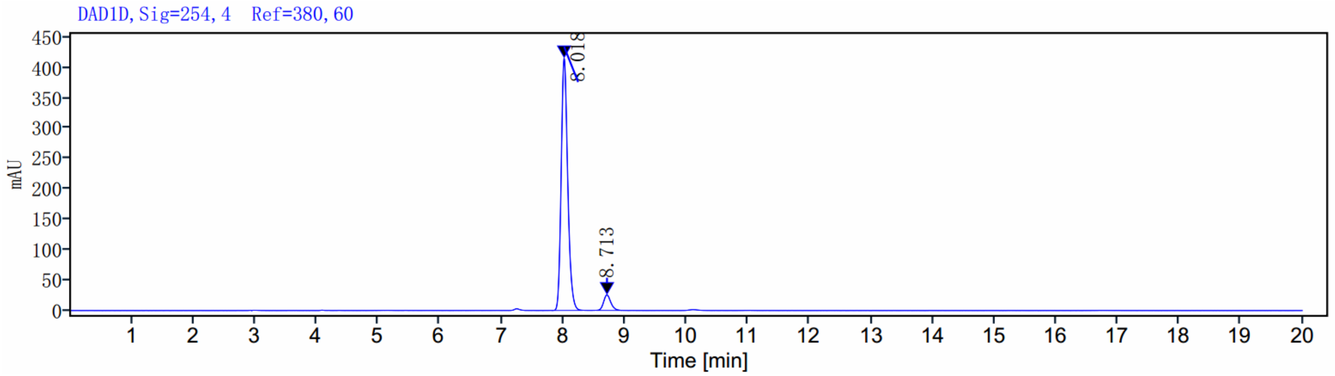

Signal: DAD1D, Sig=254, 4 Ref=380, 60

RetTime [min]	Type	Width [min]	Area [mAU*s]	Height [mAU]	Area %
8.018	BV	0.50103	3059.44142	415.62832	93.5934
8.713	VV	0.41837	209.42241	25.58551	6.4066
		Totals	3268.86383		

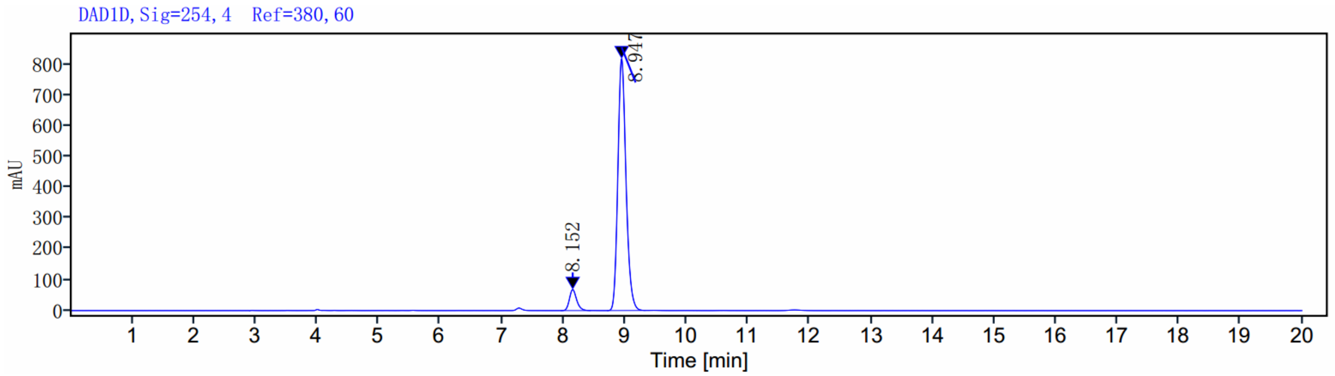

Signal: DAD1D, Sig=254, 4 Ref=380, 60

RetTime [min]	Type	Width [min]	Area [mAU*s]	Height [mAU]	Area %
8.152	BV	0.48692	561.54262	68.16476	6.9333
8.947	VV	0.61802	7537.70896	817.49292	93.0667
		Totals	8099.25157		

Supplementary Figure 153. HPLC data of **59**.

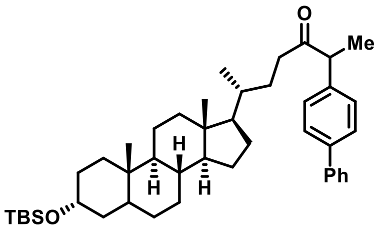

Fig. 3. (60 & 61)

(*S*)-**L**: 4:96 dr; (*R*)-**L**: 96:4 dr.

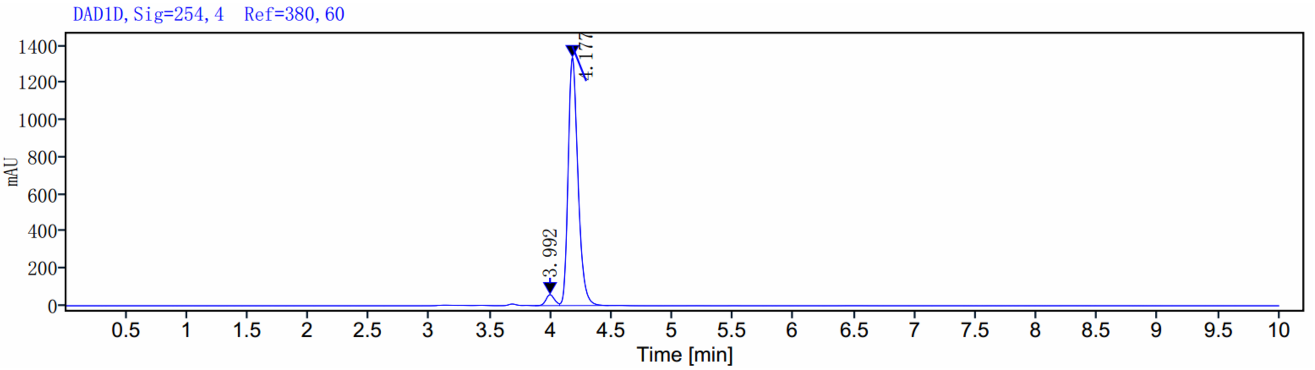

Signal: DAD1D, Sig=254, 4 Ref=380, 60

RetTime [min]	Type	Width [min]	Area [mAU*s]	Height [mAU]	Area %
3.992	BV	0.20363	290.34486	58.82904	3.7120
4.177	VB	0.40439	7531.35236	1335.82966	96.2880
		Totals	7821.69721		

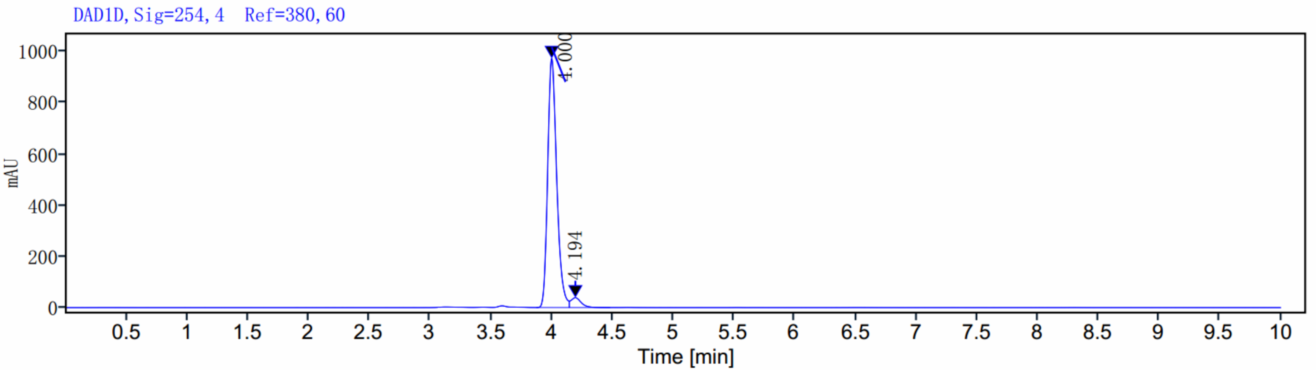

Signal: DAD1D, Sig=254, 4 Ref=380, 60

RetTime [min]	Type	Width [min]	Area [mAU*s]	Height [mAU]	Area %
4.000	BV	0.27157	5059.28145	974.71208	95.7027
4.194	VB	0.33676	227.17471	38.29723	4.2973
		Totals	5286.45616		

Supplementary Figure 154. HPLC data of **60 & 61.**

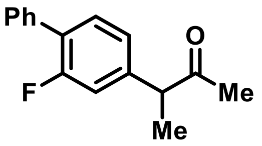

Fig. 4. (62)

(*S*)-**L**: 94% ee; (*R*)-**L**: 94% ee.

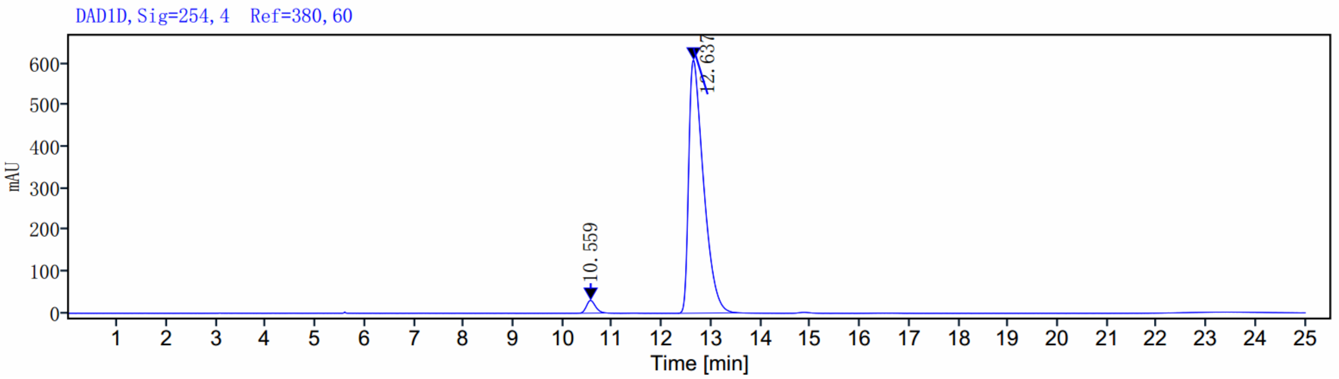

Signal: DAD1D, Sig=254, 4 Ref=380, 60

RetTime [min]	Type	Width [min]	Area [mAU*s]	Height [mAU]	Area %
10.559	VV	0.48771	386.45980	30.54815	2.9659
12.637	BV	1.18664	12643.47780	608.11505	97.0341
		Totals	13029.93760		

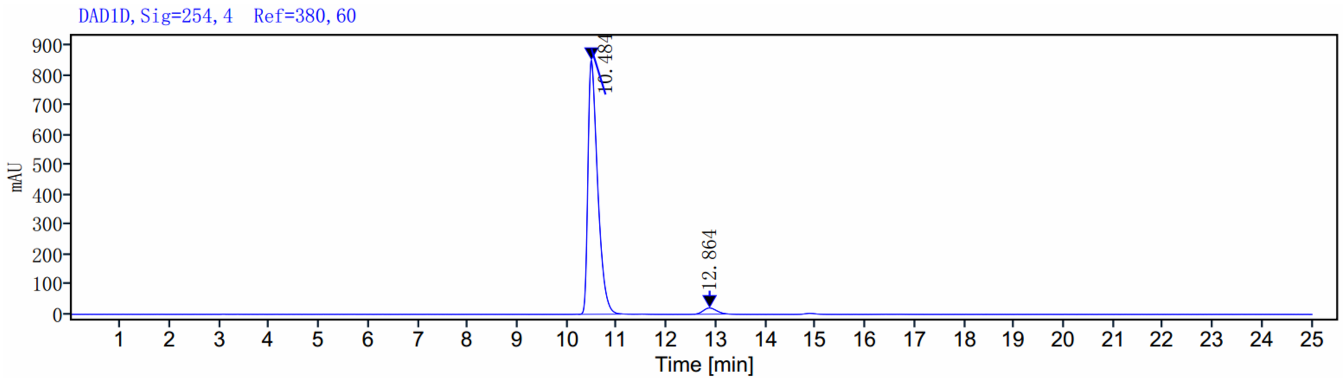

Signal: DAD1D, Sig=254, 4 Ref=380, 60

RetTime [min]	Type	Width [min]	Area [mAU*s]	Height [mAU]	Area %
10.484	BV	0.84977	11579.67032	852.64977	96.7993
12.864	VV	0.62893	382.88748	20.57577	3.2007
		Totals	11962.55780		

Supplementary Figure 155. HPLC data of **62.**

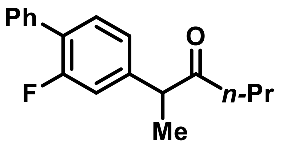

Fig. 4. (63)

(*S*)-**L**: 91% ee; (*R*)-**L**: 92% ee.

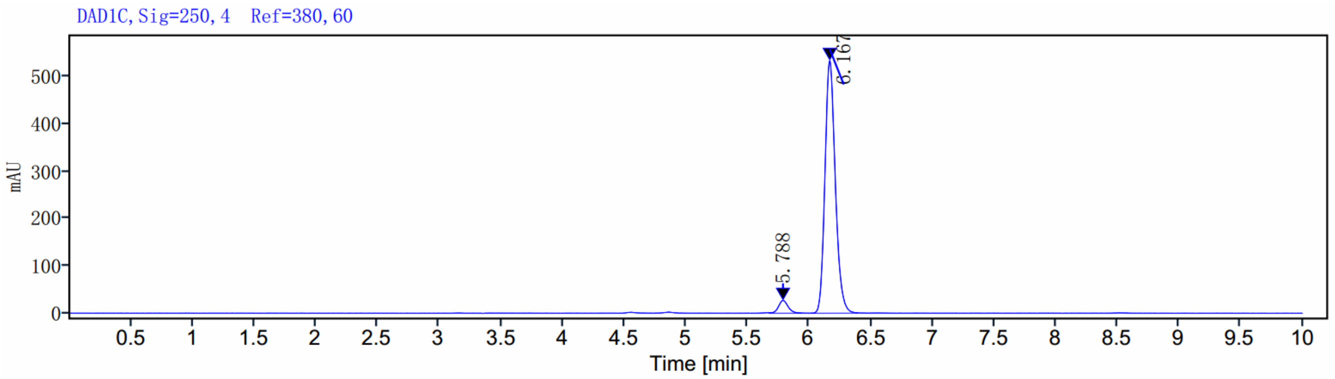

Signal: DAD1C, Sig=250, 4 Ref=380, 60

RetTime [min]	Type	Width [min]	Area [mAU*s]	Height [mAU]	Area %
5.788	VV	0.26494	146.47563	26.79601	4.4821
6.167	BV	0.38862	3121.54587	534.22369	95.5179
		Totals	3268.02150		

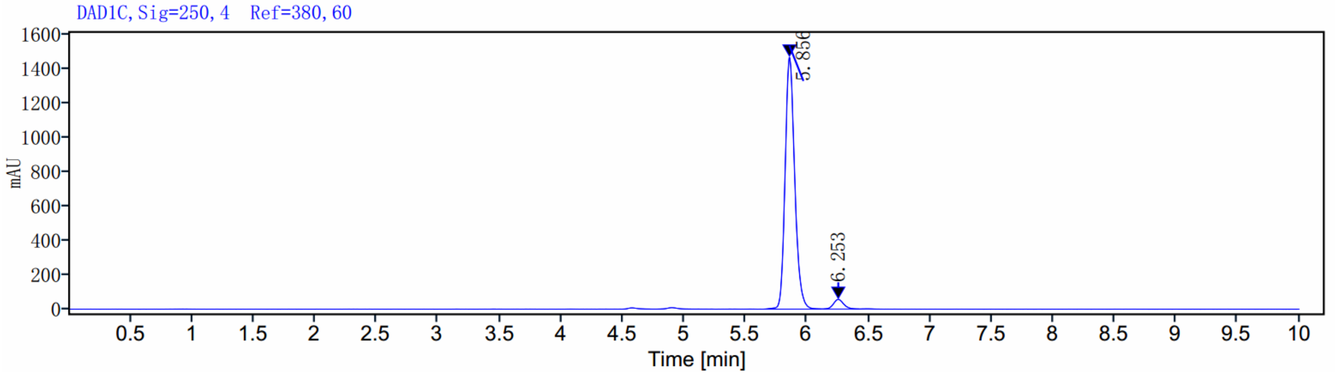

Signal: DAD1C, Sig=250, 4 Ref=380, 60

RetTime [min]	Type	Width [min]	Area [mAU*s]	Height [mAU]	Area %
5.856	VV	0.47219	7966.55706	1467.70929	96.0955
6.253	VB	0.28867	323.69426	56.02722	3.9045
		Totals	8290.25133		

Supplementary Figure 156. HPLC data of **63.**

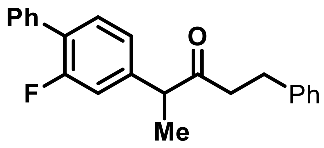

Fig. 4. (64)

(S)-**L**: 91% ee; (R)-**L**: 92% ee.

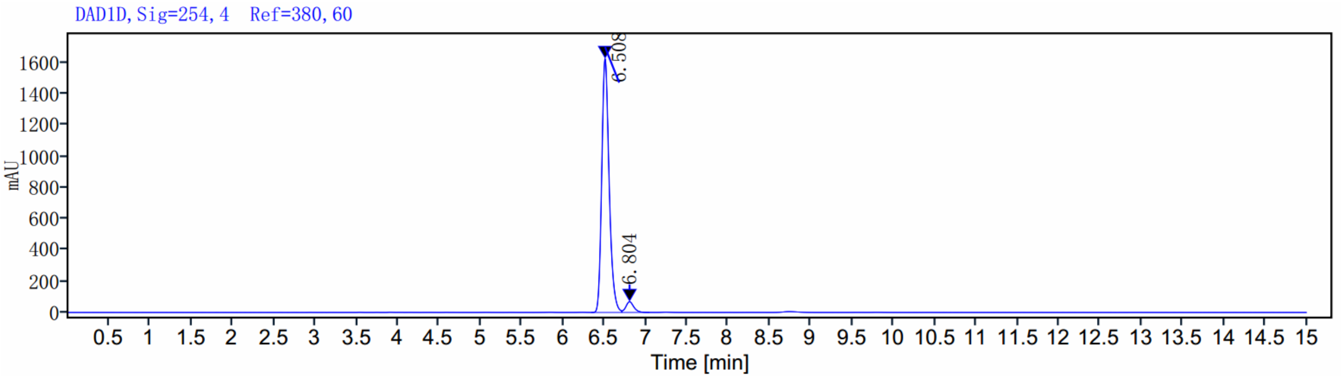

Signal: DAD1D, Sig=254, 4 Ref=380, 60

RetTime [min]	Type	Width [min]	Area [mAU*s]	Height [mAU]	Area %
6.508	BV	0.37112	10138.00950	1625.91727	95.6624
6.804	VV	0.33585	459.68597	68.74743	4.3376
		Totals	10597.69547		

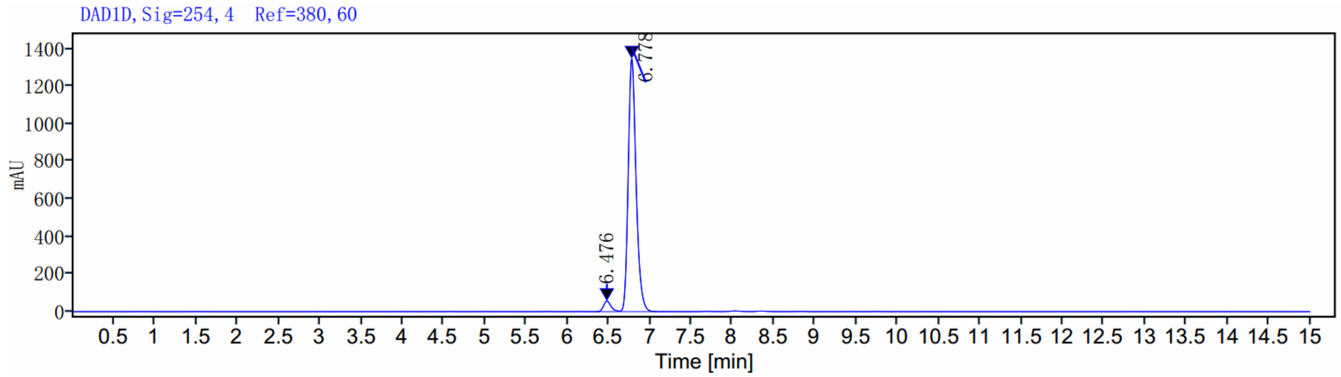

Signal: DAD1D, Sig=254, 4 Ref=380, 60

RetTime [min]	Type	Width [min]	Area [mAU*s]	Height [mAU]	Area %
6.476	BV	0.28158	356.33012	56.53489	3.8407
6.778	VV	0.45007	8921.29237	1347.34139	96.1593
		Totals	9277.62249		

Supplementary Figure 157. HPLC data of **64.**

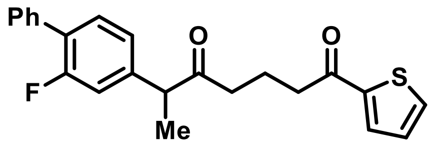

Fig. 4. (65)

(*S*)-**L**: 90% ee; (*R*)-**L**: 90% ee.

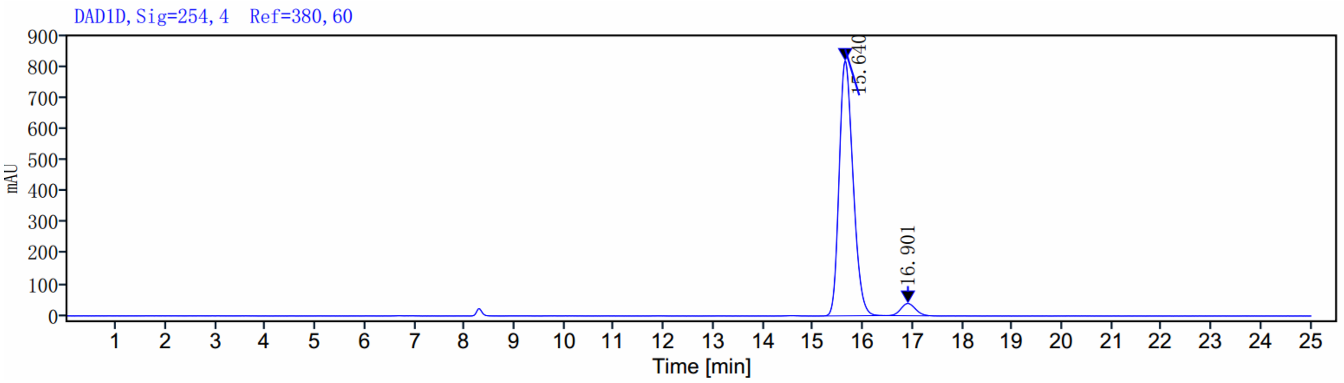

Signal: DAD1D, Sig=254, 4 Ref=380, 60

RetTime [min]	Type	Width [min]	Area [mAU*s]	Height [mAU]	Area %
15.640	VV	1.07422	15872.72633	818.17678	95.1830
16.901	VV	0.78768	803.28646	39.42833	4.8170
		Totals	16676.01280		

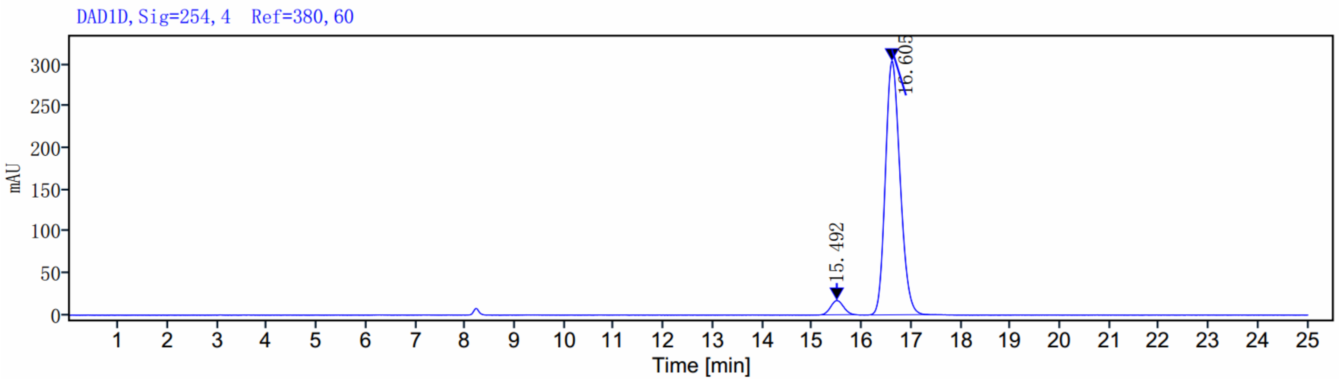

Signal: DAD1D, Sig=254, 4 Ref=380, 60

RetTime [min]	Type	Width [min]	Area [mAU*s]	Height [mAU]	Area %
15.492	MM m	1.11680	317.27913	17.47227	4.8094
16.605	VM m	1.18176	6279.75333	303.72529	95.1906
		Totals	6597.03246		

Supplementary Figure 158. HPLC data of **65.**

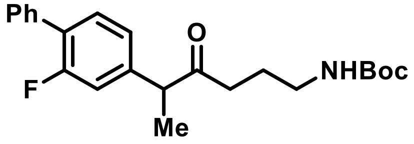

Fig. 4. (66)

(*S*)-**L**: 91% ee; (*R*)-**L**: 92% ee.

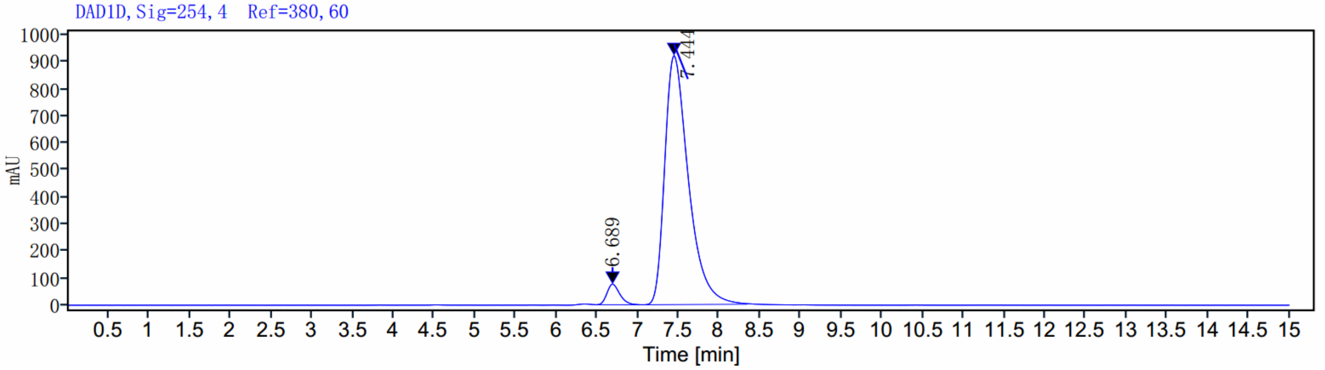

Signal: DAD1D, Sig=254, 4 Ref=380, 60

RetTime [min]	Type	Width [min]	Area [mAU*s]	Height [mAU]	Area %
6.689	VV	0.51793	833.82645	76.17491	4.2689
7.444	BV	1.26219	18698.75231	922.72049	95.7311
		Totals	19532.57876		

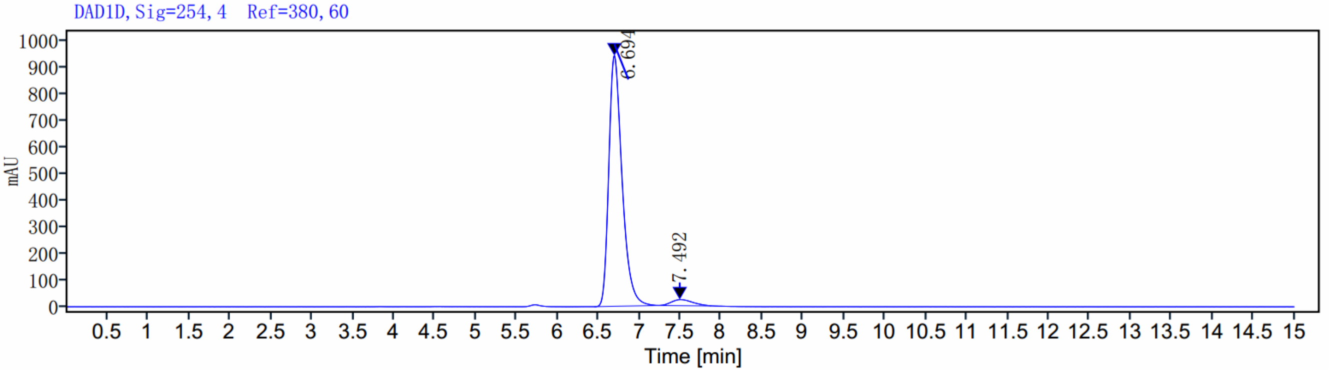

Signal: DAD1D, Sig=254, 4 Ref=380, 60

RetTime [min]	Type	Width [min]	Area [mAU*s]	Height [mAU]	Area %
6.694	BV	0.75645	10649.07533	943.58110	96.2461
7.492	BV m	0.63194	415.34325	22.81320	3.7539
		Totals	11064.41858		

Supplementary Figure 159. HPLC data of **66.**

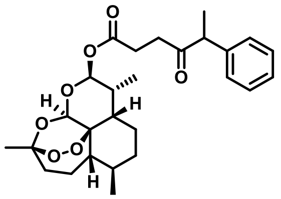

Fig. 4. (67)

(*S*)-**L**: 98:2 dr; (*R*)-**L**: 1:99 dr.

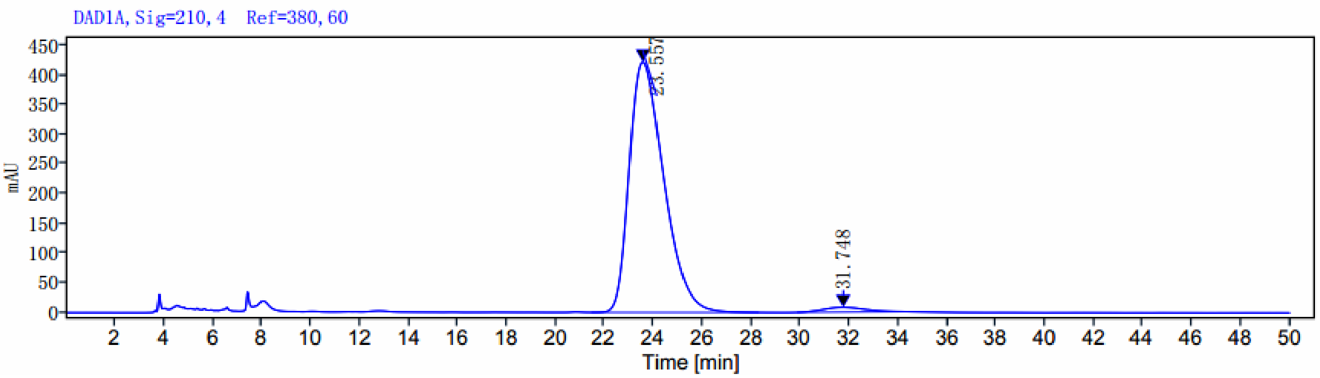

Signal: DAD1A, Sig=210, 4 Ref=380, 60

RetTime [min]	Type	Width [min]	Area [mAU*s]	Height [mAU]	Area %
23.557	MM m	6.86275	41389.16070	421.58195	97.6805
31.748	MM m	4.45261	982.82735	8.25997	2.3195
		Totals	42371.98805		

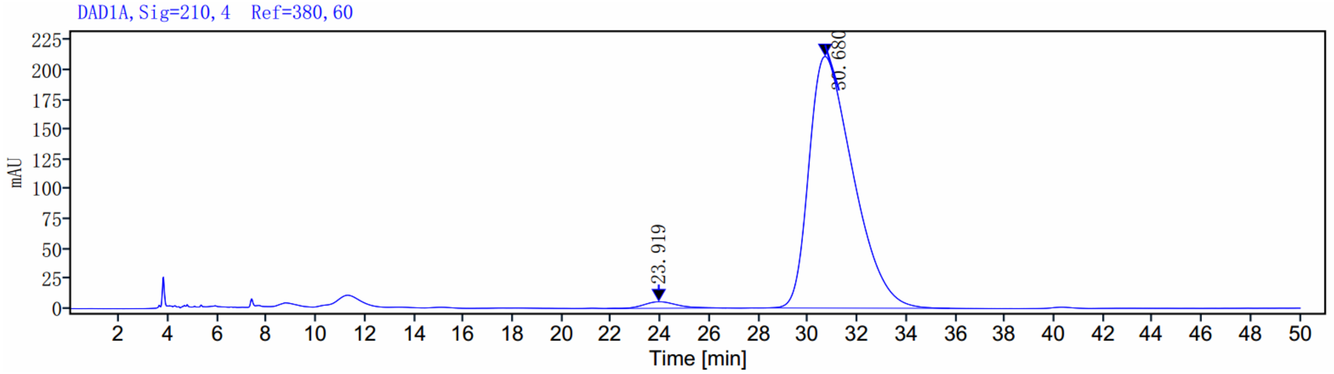

Signal: DAD1A, Sig=210, 4 Ref=380, 60

RetTime [min]	Type	Width [min]	Area [mAU*s]	Height [mAU]	Area %
23.919	MM m	4.86103	513.90158	5.49114	1.9168
30.680	MM m	8.00640	26296.33560	211.03832	98.0832
		Totals	26810.23718		

Supplementary Figure 160. HPLC data of **67.**

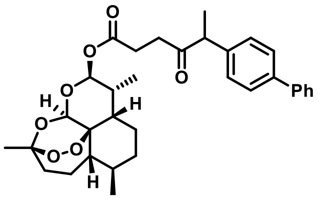

Fig. 4. (68)

(*S*)-**L**: 97:3 dr; (*R*)-**L**: 5:95 dr.

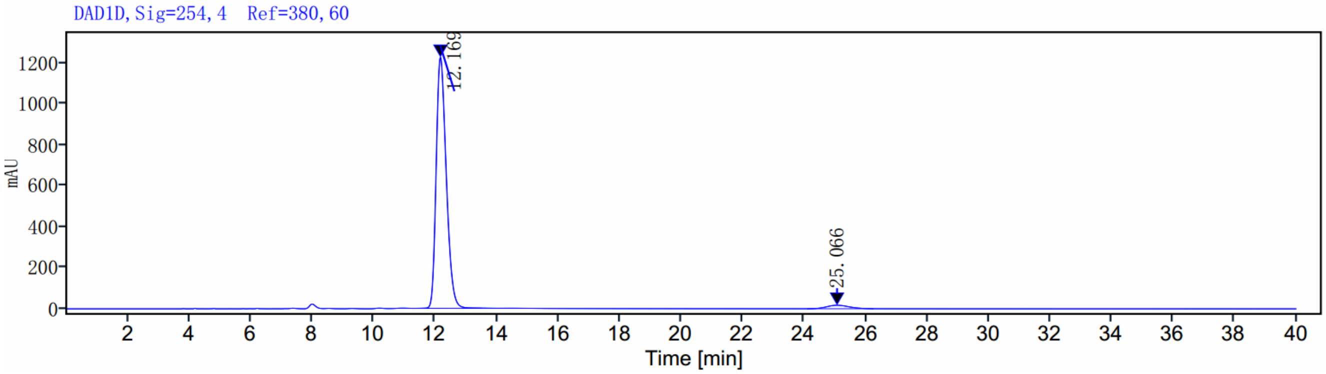

Signal: DAD1D, Sig=254, 4 Ref=380, 60

RetTime [min]	Type	Width [min]	Area [mAU*s]	Height [mAU]	Area %
12.169	BV	2.16557	27983.27050	1228.12992	96.9670
25.066	VV	2.15439	875.29416	16.99626	3.0330
		Totals	28858.56466		

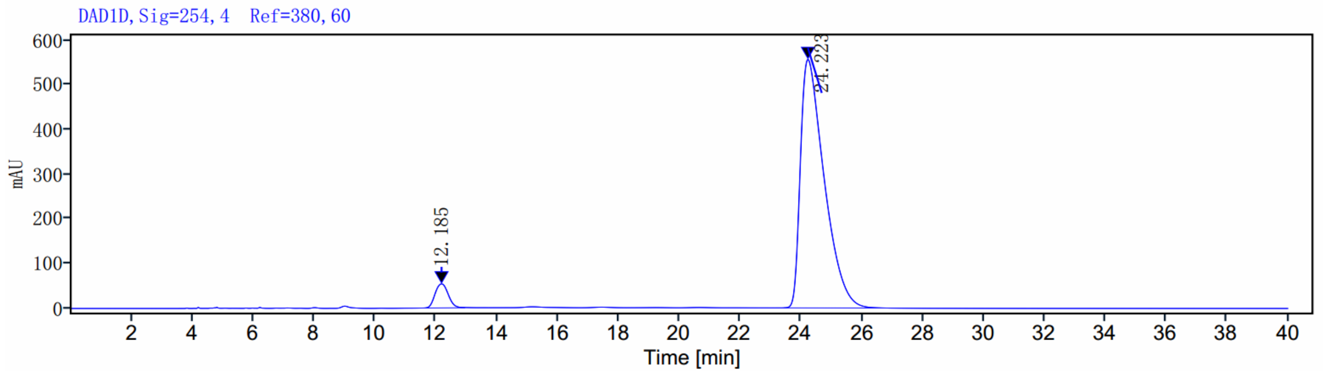

Signal: DAD1D, Sig=254, 4 Ref=380, 60

RetTime [min]	Type	Width [min]	Area [mAU*s]	Height [mAU]	Area %
12.185	VV	1.39010	1613.92705	53.90877	5.0869
24.223	VV	3.16829	30113.42253	556.24601	94.9131
		Totals	31727.34958		

Supplementary Figure 161. HPLC data of **68.**

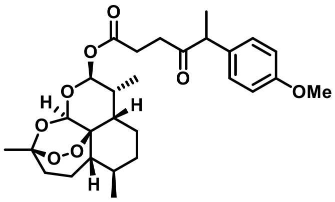

Fig. 4. (69)

(*S*)-**L**: 97:3 dr; (*R*)-**L**: 2:98 dr.

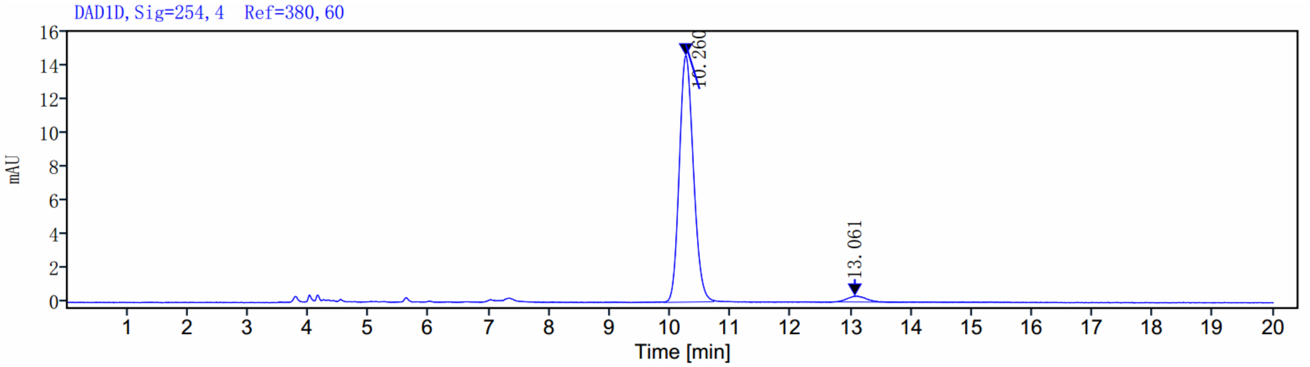

Signal: DAD1D, Sig=254, 4 Ref=380, 60

RetTime [min]	Type	Width [min]	Area [mAU*s]	Height [mAU]	Area %
10.260	BV	0.81812	237.37396	14.61249	96.9023
13.061	MM m	1.05620	7.58821	0.35708	3.0977
		Totals	244.96217		

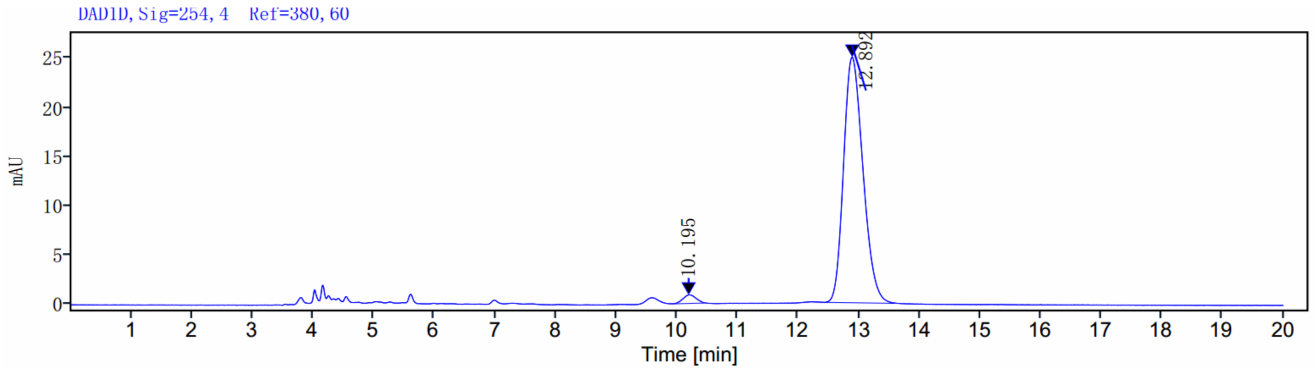

Signal: DAD1D, Sig=254, 4 Ref=380, 60

RetTime [min]	Type	Width [min]	Area [mAU*s]	Height [mAU]	Area %
10.195	VV	0.50653	12.98881	0.87018	2.2901
12.892	BV	1.12178	554.17443	24.90426	97.7099
		Totals	567.16324		

Supplementary Figure 162. HPLC data of **69.**

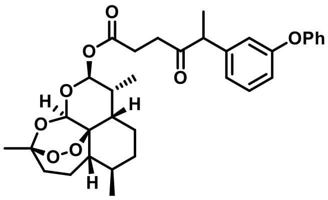

Fig. 4. (70)

(*S*)-**L**: 98:2 dr; (*R*)-**L**: 2:98 dr.

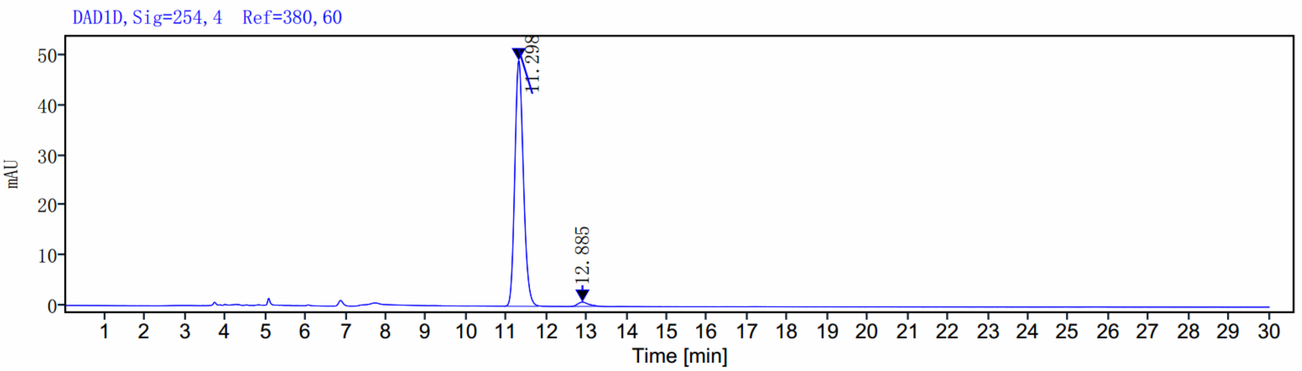

Signal: DAD1D, Sig=254, 4 Ref=380, 60

RetTime [min]	Type	Width [min]	Area [mAU*s]	Height [mAU]	Area %
11.298	VV	0.84998	730.77889	49.20647	97.8615
12.885	VV m	0.66209	15.96894	0.87367	2.1385
		Totals	746.74782		

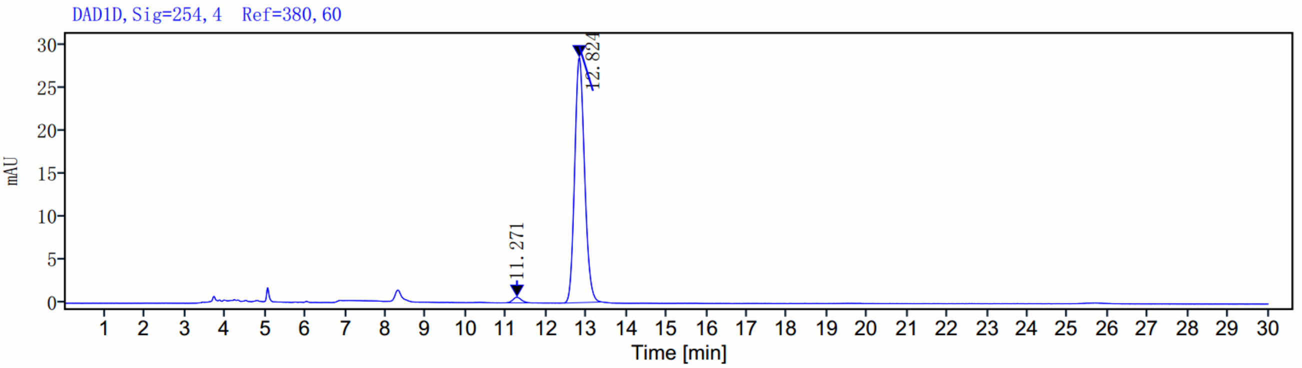

Signal: DAD1D, Sig=254, 4 Ref=380, 60

RetTime [min]	Type	Width [min]	Area [mAU*s]	Height [mAU]	Area %
11.271	VV	0.49514	9.49114	0.65883	1.9118
12.824	VV	0.90540	486.95553	28.41564	98.0882
		Totals	496.44667		

Supplementary Figure 163. HPLC data of **70.**

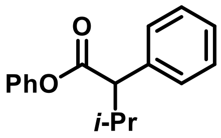

Fig. 6. (72)

(*S, R*)-**L**: 98% ee; (*R, S*)-**L**: 98% ee.

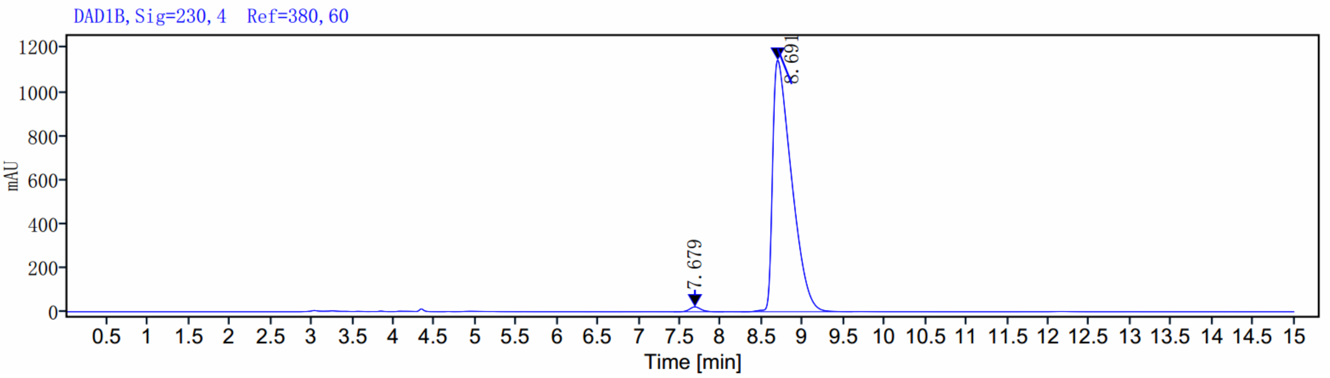

Signal: DAD1B, Sig=230, 4 Ref=380, 60

RetTime [min]	Type	Width [min]	Area [mAU*s]	Height [mAU]	Area %
7.679	BV	0.61006	208.98239	22.09214	1.1240
8.691	BV	1.35997	18384.25796	1145.88593	98.8760
		Totals	18593.24035		

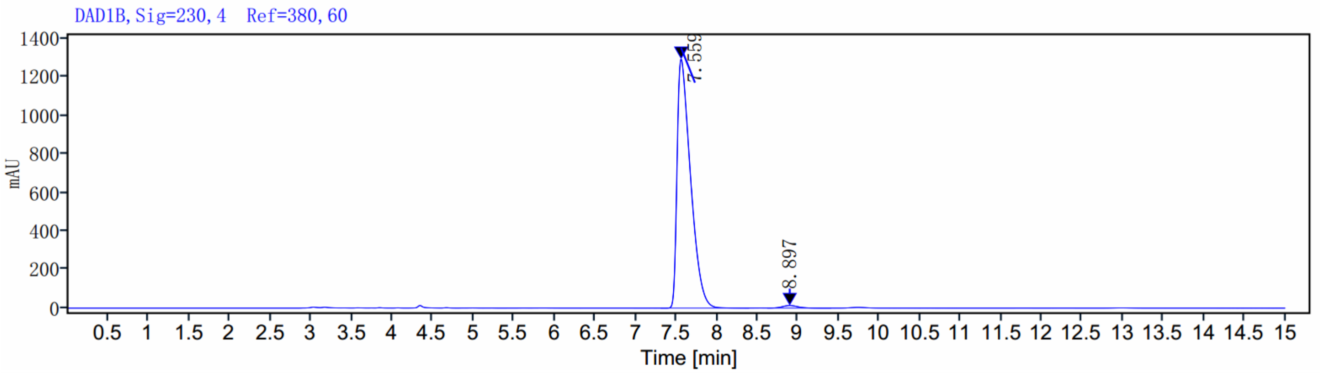

Signal: DAD1B, Sig=230, 4 Ref=380, 60

RetTime [min]	Type	Width [min]	Area [mAU*s]	Height [mAU]	Area %
7.559	BV	1.08843	14884.07834	1292.49864	98.8140
8.897	VB	0.78430	178.64007	13.37091	1.1860
		Totals	15062.71841		

Supplementary Figure 164. HPLC data of **72.**

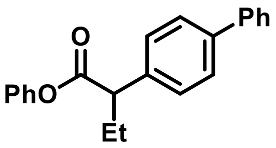

Fig. 6. (73)

(*S*, *R*)-**L**: 91% ee; (*R*, *S*)-**L**: 90% ee.

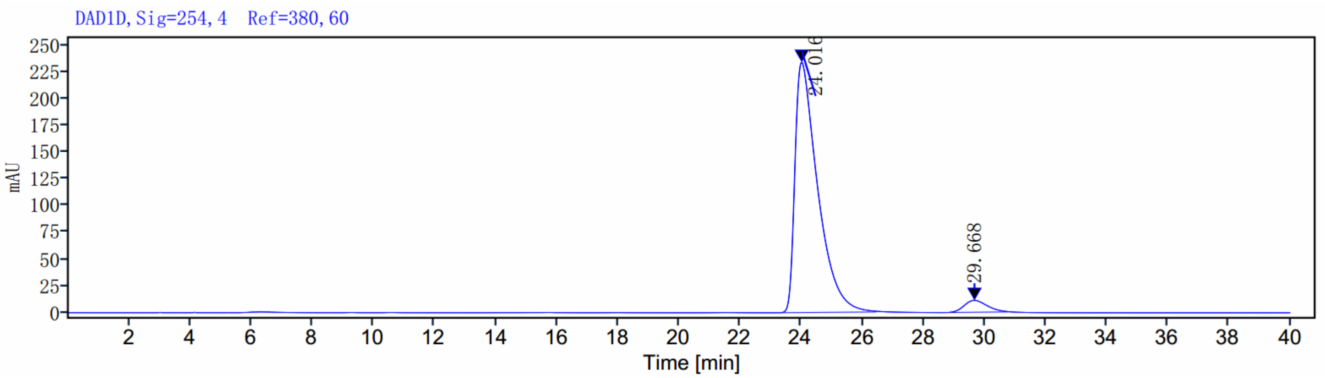

Signal: DAD1D, Sig=254, 4 Ref=380, 60

RetTime [min]	Type	Width [min]	Area [mAU*s]	Height [mAU]	Area %
24.016	VV	3.11685	12048.14961	233.65312	95.4110
29.668	BV m	2.01607	579.47946	11.10916	4.5890
		Totals	12627.62907		

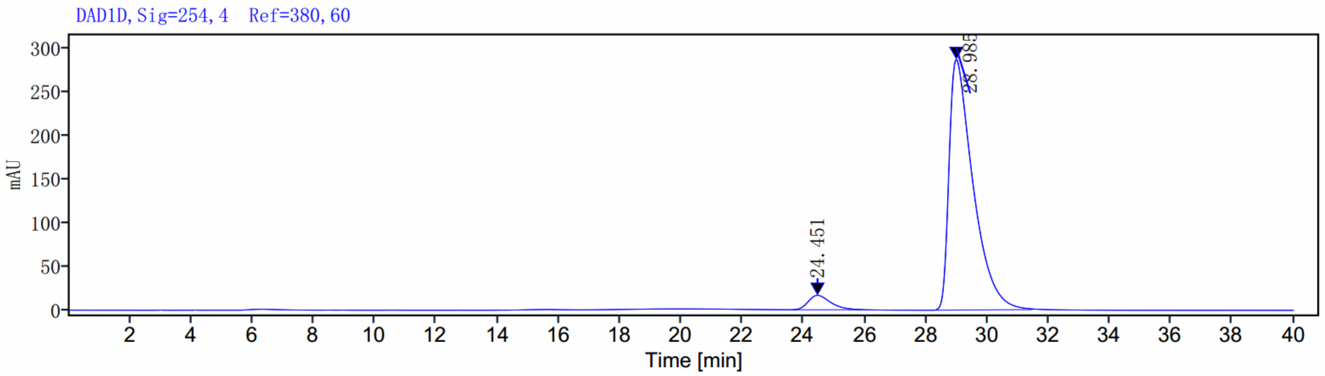

Signal: DAD1D, Sig=254, 4 Ref=380, 60

RetTime [min]	Type	Width [min]	Area [mAU*s]	Height [mAU]	Area %
24.451	VV	2.23862	843.52600	16.59617	5.1119
28.985	VV	3.27521	15657.77250	287.89541	94.8881
		Totals	16501.29850		

Supplementary Figure 165. HPLC data of **73.**

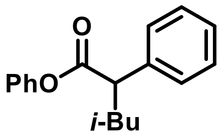

Fig. 6. (74)

(*S*, *R*)-**L**: 83% ee; (*R*, *S*)-**L**: 81% ee.

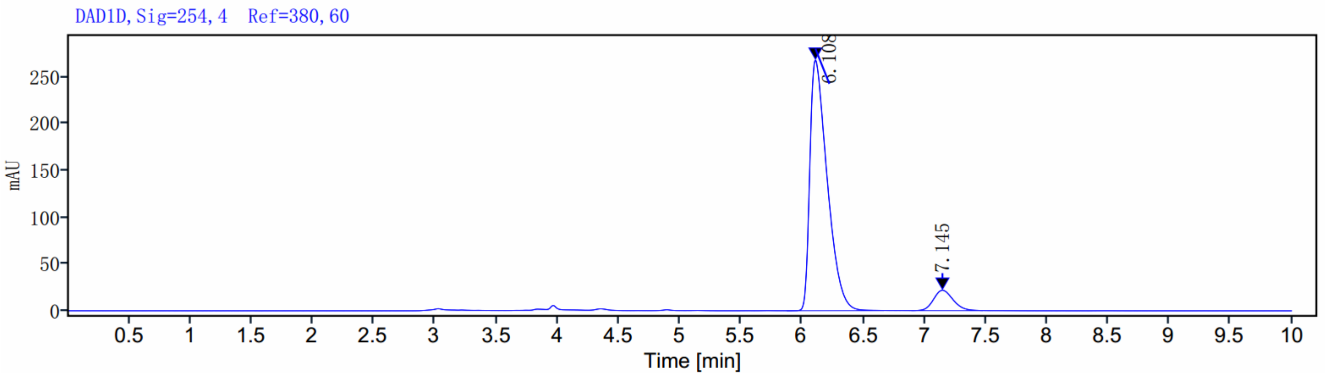

Signal: DAD1D, Sig=254, 4 Ref=380, 60

RetTime [min]	Type	Width [min]	Area [mAU*s]	Height [mAU]	Area %
6.108	BV	0.79184	2641.59114	268.13187	91.6085
7.145	VV	0.58124	241.97575	21.83809	8.3915
		Totals	2883.56689		

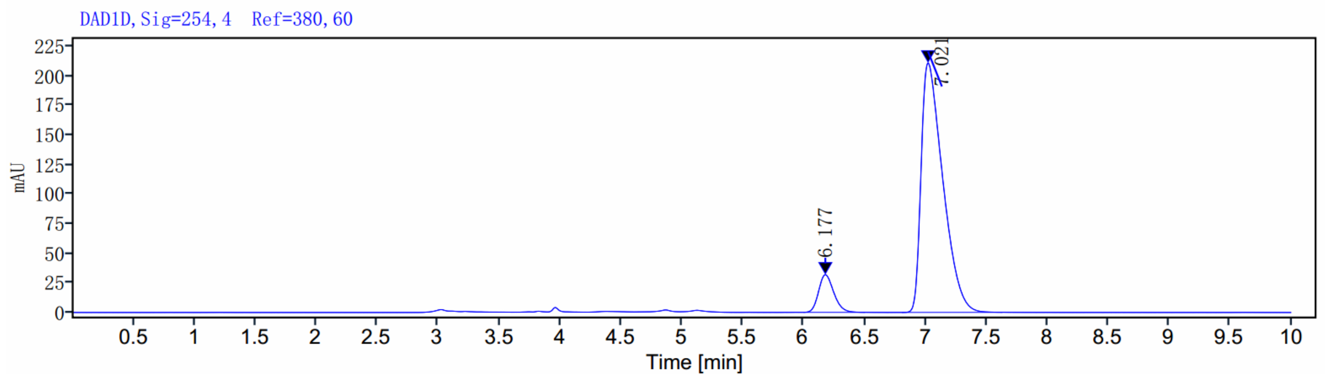

Signal: DAD1D, Sig=254, 4 Ref=380, 60

RetTime [min]	Type	Width [min]	Area [mAU*s]	Height [mAU]	Area %
6.177	VV	0.50357	276.74952	31.90226	9.5629
7.021	VV	0.82275	2617.22780	211.34837	90.4371
		Totals	2893.97732		

Supplementary Figure 166. HPLC data of **74.**

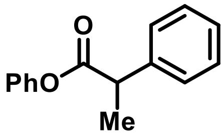

Fig. 6. (75)

(*S, R*)-**L**: 83% ee; (*R, S*)-**L**: 83% ee.

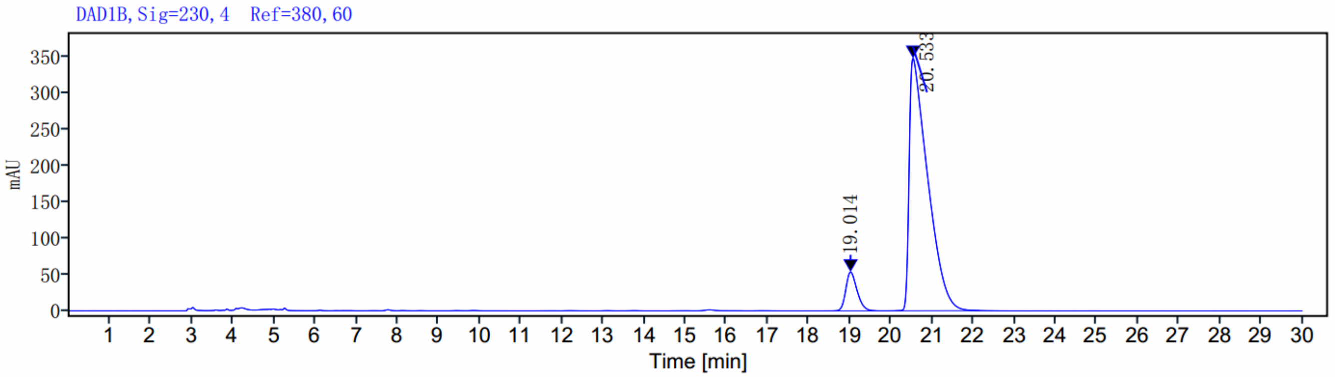

Signal: DAD1B, Sig=230, 4 Ref=380, 60

RetTime [min]	Type	Width [min]	Area [mAU*s]	Height [mAU]	Area %
19.014	VV	1.05424	1016.77705	53.53552	8.5648
20.533	VV	2.05456	10854.78433	348.14530	91.4352
		Totals	11871.56138		

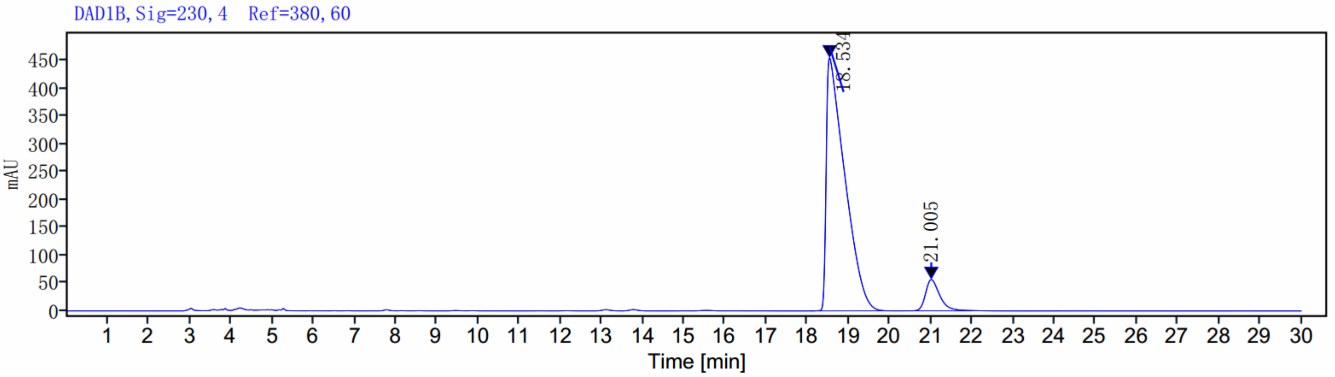

Signal: DAD1B, Sig=230, 4 Ref=380, 60

RetTime [min]	Type	Width [min]	Area [mAU*s]	Height [mAU]	Area %
18.534	VV	1.88497	14180.22071	454.28796	91.2816
21.005	VV	1.48065	1354.35970	56.16635	8.7184
		Totals	15534.58041		

Supplementary Figure 167. HPLC data of **75.**

VIII. Supplementary References

1. Zhang, W. et al. Enantioselective cyanation of benzylic C–H bonds via copper-catalyzed radical relay. *Science* **353,** 1014–1018 (2016).

2. a) Dolomanov, O. V., Bourhis, L. J., Gildea, R. J., Howard, J. A. K. & Puschmann, H. OLEX2: a complete structure solution, refinement and analysis program. *J. Appl. Cryst.* **42,** 339–341 (2009). b) Bourhis, L. J., Dolomanov, O. V., Gildea, R. J., Howard, J, A. K. & Puschmann, H. The anatomy of a comprehensive constrained, restrained refinement program for the modern computing environment-Olex2 dissected. *Acta Cryst.* **A71,** 59–75 (2015).

3. Sheldrick, G. M. SHELXT-Integrated space-group and crystal-structure determination. *Acta Cryst.* **A71,** 3–8 (2015).

4. Gandolfo, E., Tang, X., Raha, R. S. & Melchiorre, P. Photochemical asymmetric nickel-catalyzed acyl cross-coupling. *Angew. Chem. Int. Ed.* **58,** 16854–16858 (2019).

5. Mao, J. et al. Cobalt-bisoxazoline-catalyzed asymmetric Kumada cross-coupling of racemic α-bromo esters with aryl Grignard reagents. *J. Am. Chem. Soc.* **136,** 17662–17668 (2014).
